# The role of water and protein flexibility in the structure-based virtual screening of allosteric GPCR modulators: an mGlu_5_ receptor case study

**DOI:** 10.1007/s10822-019-00224-w

**Published:** 2019-09-21

**Authors:** Zoltán Orgován, György G. Ferenczy, György M. Keserű

**Affiliations:** Medicinal Chemistry Research Group, Research Centre for Natural Sciences, Hungarian Academy of Sciences, Magyar tudósok krt. 2, Budapest, 1117 Hungary

**Keywords:** Metabotropic glutamate receptor 5, Virtual screening, Induced fit binding, Structural water

## Abstract

Stabilizing unique receptor conformations, allosteric modulators of G-protein coupled receptors (GPCRs) might open novel treatment options due to their new pharmacological action, their enhanced specificity and selectivity in both binding and signaling. Ligand binding occurs at intrahelical allosteric sites and involves significant induced fit effects that include conformational changes in the local protein environment and water networks. Based on the analysis of available crystal structures of metabotropic glutamate receptor 5 (mGlu_5_) we investigated these effects in the binding of mGlu_5_ receptor negative allosteric modulators. A large set of retrospective virtual screens revealed that the use of multiple protein structures and the inclusion of selected water molecules improves virtual screening performance compared to conventional docking strategies. The role of water molecules and protein flexibility in ligand binding can be taken into account efficiently by the proposed docking protocol that provided reasonable enrichment of true positives. This protocol is expected to be useful also for identifying intrahelical allosteric modulators for other GPCR targets.

**Electronic supplementary material:**

The online version of this article (10.1007/s10822-019-00224-w) contains supplementary material, which is available to authorized users.

## Introduction

Structure-based virtual screening is an established computational technique for the identification of chemical starting points in drug discovery programs [1]. Virtual screening allows to select compounds for experimental testing that improves the efficiency of hit finding. Structure-based screening requires atomic resolution protein structures that are available either from experiment or as computational models. Owing to the increasing availability of GPCR X-ray structures these important class of proteins are also extensively and successfully used in structure-based virtual screening campaigns [2]. Typically, GPCR virtual screen aims to identify ligands binding in the intrahelical orthosteric binding site.

GPCRs contain a wide variety of allosteric sites and their targeting with small molecules offers unique therapeutic potential [3–5]. As it has been discussed recently [6] allosteric sites are widely distributed intrahelically in the TM bundle, at extrahelical positions within the membrane binding region and at the intracellular signalling protein interface. These sites may or may not be preformed before ligand binding and they are generally sensitive to protein conformational changes and to the presence of and the interactions with water molecules. We selected the metabotropic glutamate receptor 5 (mGlu_5_ receptor) for a detailed investigation because its allosteric site is structurally well characterized owing to the receptor complexes co-crystallized with a variety of allosteric ligands [7–10]. Moreover, a wide range of allosteric ligands with biochemical data have been reported. Therefore, the mGlu_5_ receptor is well suited to investigate how virtual screening can find allosteric ligands and what are the special considerations needed for maximizing its value in allosteric ligand discovery. Metabotropic glutamate receptors belong to the family C of GPCRs, and can influence neurotransmitter release or modulate ion channel activity [11]. Their endogen ligand is l-glutamic acid, a major excitatory neurotransmitter in the central nervous system (CNS) of mammals. Modulation of glutamate transmission has a potential in the treatment of several psychiatric and neurological disorders. Glutamate binds to the extracellular Venus flytrap domain that is linked to the seven transmembrane domain through a cysteine-rich domain. Non-competitive antagonists which bind in the allosteric site are called negative allosteric modulators (NAMs), and could be useful in the treatment of numerous diseases [12].

Early drug discovery efforts targeted the orthosteric binding site, but this site is highly conserved in the mGlu receptor family, making orthosteric modulation by subtype selective ligands highly challenging. However, selective allosteric modulation with ligands targeting the ancestral intrahelical site (whose position resemble to the orthosteric site of most GPCRs) [13] appears to be more attainable. Large number of allosteric modulators have been reported, although few of them have reached clinic to date [14]. This can be attributed to the significant challenges in the medicinal chemistry optimization of allosteric ligands. The complex, multiparametric approach, characteristic to medicinal chemistry optimizations in general, is further complicated by other factors like allosteric coupling and cooperativity, functional activity and effect on signalling that all have to be taken into account [13].

The allosteric binding site of mGlu_5_ receptor can be found within the heptahelical transmembrane domain and this pocket is completely covered by the second extracellular loop (EC2) [7]. The transmembrane helices (TM2, TM5 and TM7) of mGlu_5_ receptor are moved closer to each other and consequently the volume of the pocket is significantly reduced with respect to the typical GPCR class A and class B intrahelical pockets. These latter pockets are the binding sites of their endogenous ligands and they are also the principal target of compounds designed for GPCR activity modulation. In contrast, mGlu_5_ receptor intrahelical pocket is a functional water channel whose water molecules are likely to participate in signalling [9, 15]. This narrow pocket accommodates a well-structured water network and no small molecule is known to bind for any physiological function. Nevertheless, several exogenous compounds binding to this site have been identified. Their optimization into drug candidates, however, is hindered by irregularly steep structure–activity relationship (SAR), functional switches by small structural changes and limited SAR transferability between chemotypes [14].

**Fig. 1:**
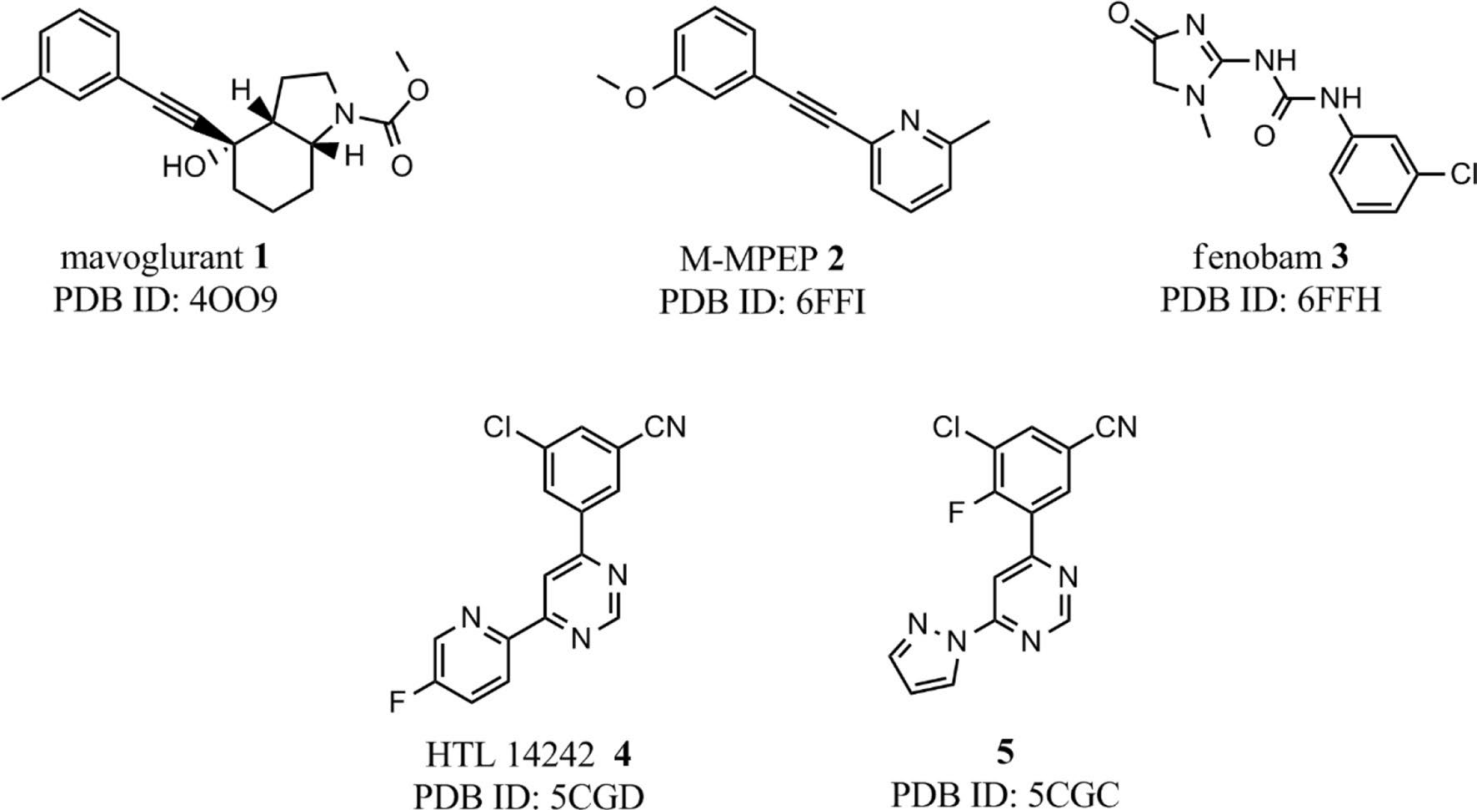
Allosteric ligands of mGlu_5_ receptor X-ray structures available in the PDB

X-ray structures of complexes formed between allosteric ligands (Fig. 1) and mGlu_5_ receptor proved to be indispensable to understand the unconventional features of ligand binding.

The allosteric pocket of mGlu_5_ receptor is made up of a lower and an upper chamber connected by a narrow linker region. The upper chamber is present throughout the mGlu family and its location is similar to the orthosteric binding pocket of class A GPCRs. However, the lower chamber is present only in mGlu_5_ receptor owing to the residues Pro655^3.40^ Gly628^2.49^ and Ala810^7.41^, all are smaller than the corresponding residues in the seven other mGlu receptors. In particular, the small size of Pro655 creates a narrow channel towards the lower chamber allowing the ligands to penetrate deeper into the crevice among the helices.

**Fig. 2:**
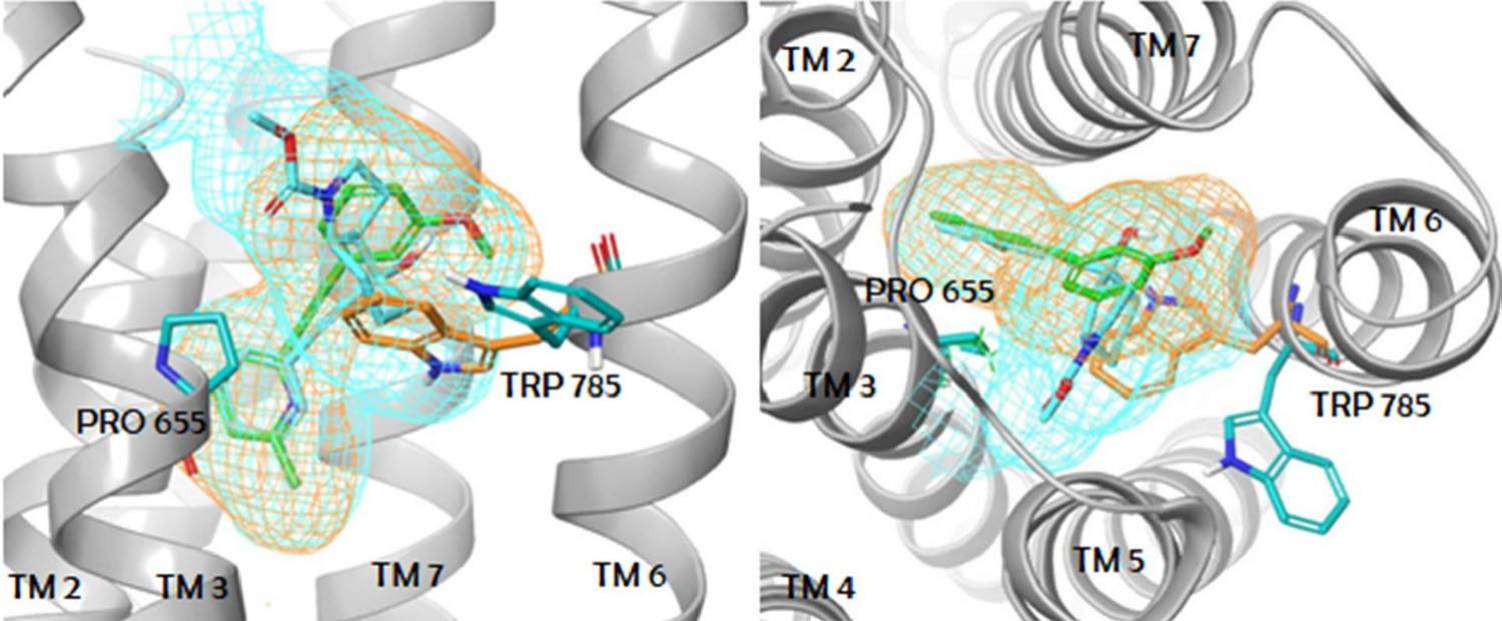
mGlu_5_ binding pocket with mavoglurant (cyan) and with M-MPEP (green) from two perspectives. The shape of the pockets for mavoglurant and for M-MPEP are depicted with cyan and orange meshes, respectively. Trp785 is shown as cyan and orange sticks in its out and in conformations, respectively

Available complex structures of the mGlu_5_ receptor contain ligands with three different linkers. Mavoglurant [16] and M-MPEP [17] both have an acetylenic linker, a urea linker is present in fenobam [18] and pyrimidine in HTL-14242 (**4**) and in **5** [8]. Although mavoglurant and M-MPEP share a common acetylenic linker, this moiety does not perfectly overlay in the two crystal structures and the growing vectors of the headgroups (condensed rings in mavoglurant and methoxyphenyl in M-MPEP) have different orientation for the two ligands [9]. These findings clearly show that modelling of ligand binding is challenging even for a chemotype with available X-ray structures. Indeed, docking of M-MPEP into the protein conformation obtained from the mGlu_5_ receptor–mavoglurant complex results in two distinct cluster of poses, one that corresponds to the X-ray structure, and another in which the methoxyphenyl group is rotated by 180°. The mGlu_5_ receptor binding pocket is significantly different in the mavoglurant and the M-MPEP complexes primarily owing to the different conformations adopted by Trp785^6.50^. The indole ring of Trp785^6.50^ points outward, toward TM5 in the former (χ_1_ = − 170°; χ_2_ = 92°), and it points inward, towards the centre of the helical bundle in the latter (χ_1_ = − 76°; χ_2_ = 63°) (Fig. 2). These two conformations of Trp785^6.50^ result in different shapes of the allosteric binding pocket and warn that protein flexibility and induced fit binding have to be taken into account in the molecular modelling of ligand binding to mGlu_5_ receptor. This is also supported by several structure-based computational studies which were performed previously to map ligand–protein interactions and to understand irregular SAR using pharmacophore modelling, molecular docking and molecular dynamics simulations [15, 19–25]. Moreover, analysis of the X-ray structures coupled with computational water network analysis performed by WaterFLAP showed that many of the ligand–protein interactions are mediated by water molecules and this also has to be taken into account in interpreting structure–activity relationships across chemotypes. We found that self-docking of M-MPEP into empty mGlu_5_ receptor (PDB: 6FFI) results in a pose rotated by 180° with respect to the X-ray pose. In contrast, including the water molecule consistently observed in the X-ray structures results in the X-ray docking pose. This finding confirms that ligand binding is importantly affected by the presence of this water molecule. The sensitivity of the protein conformation, protein–ligand and water mediated interactions to small variations of ligand structures [9] results in unexpected binding modes and irregular structure–activity relationships, and hinders the application of both structure-, and ligand-based modelling methods in a standard manner.

In the present study we evaluate the role of protein flexibility and allosteric binding site water network on structure-based virtual screening on the mGlu_5_ receptor. Similar analysis was performed for the orthosteric site of class A GPCRs [26–28] but the special features of the mGlu_5_ receptor necessitates a separate analysis for this class C receptor. The intrahelical site of mGlu_5_ receptor is the ancestral pocket of GPCRs that is the orthosteric site in class A GPCRs, but appears to be a functional water channel involved in the activation process in mGlu receptors. We selected three mGlu_5_ receptor complexes with available X-ray structures; complexes of mavoglurant, fenobam and HTL-14242 (**4**). The first two ligands share a linear shape in contrast to ligand **4** having a bent shape (Fig. 1). The other two complexes with available X-ray structures (M-MPEP [9], **5** [8]) were also investigated for including them into the study. M-MPEP is similar to mavoglurant in having acetylenic linker, however, it was observed that this protein structure is able to accommodate less than 40% of the acetylenic compounds compiled for docking. This finding shows that the protein conformation is not only well adapted to the thin acetylenic linker but also to the limited extension of the head groups. Indeed, the methoxyphenyl group of M-MPEP is significantly smaller than the corresponding methyl-octahydroindole-carboxylate group in mavoglurant and this results in the inward orientation of Trp785^6.50^ and spatially highly restrictive allosteric site. Therefore, the protein structure of the M-MPEP complex was not used in our study. X-ray structure for compound **5** is also available, however, this compound and the corresponding protein structure is highly similar to that of HTL-14242 and the higher resolution structure of the latter (R(5CGC) = 3.1 Å vs. R(5CGD) = 2.6 Å) [8] was selected for our study.

Virtual screening was performed with compound sets of known active ligands and physically similar decoys. Negative allosteric modulators were only considered as active compounds as all available high resolution mGlu_5_ crystal structures include NAM complexes, and protein conformations in PAM complexes are expected to be significantly different based on experimental [10] and computational analyses [15, 29, 30]. Because of the small number of low affinity NAMs reported for the mGlu_5_ receptor (503 with IC_50_ > 10 µM, 429 with IC_50_ > 5 µM activity) DUD-E [31, 32] was used to generate decoys with physico-chemical properties similar to the actives but with dissimilar fingerprints to minimize topological similarity. Early enrichment factors of protein structures derived from three complexes and containing varying number of water molecules were investigated to explore the effect of protein flexibility and water network on the performance of structure based virtual screening.

Previous docking studies into mGlu receptors explored ligand–protein interactions and their variations depending on receptor subtype [23, 33, 34] and on differences in ligand structures [35] and included the effect of protein flexibility and the interactions with water molecules at various levels. A comprehensive treatment of protein flexibility by induced fit docking and the interactions with explicit water molecules is presented by Harpsøe et al. [23] in a comparative analysis of the allosteric sites of mGlu receptor subtypes and their interactions with various ligands. In contrast, our objective is to devise a virtual screening scheme that takes into account protein flexibility and water mediated interactions found to be crucial in mGlu_5_ receptor–ligand binding. Conventional docking methods treat the protein environment as being rigid, and does not operate with the presence of water molecules in the binding pocket, hence these methods are unable to identify experimental poses, and proper docking scores for mGlu_5_ ligands. Validation by retrospective virtual screening campaigns was used to show that the proposed scheme outperforms conventional docking strategies and could be useful for other systems where these effects are important.

## Methods

Ligand preparation, Protein preparation, Docking: mGlu_5_ receptor negative allosteric modulators were downloaded from the ChEMBL database [36]. Compounds with lower than 1 µM IC_50_ or EC_50_ values on the human mGlu_5_ receptor were selected. The highest activity was considered for compounds with multiple reported activities. An active set with 309 compounds was compiled in this way. These ligands were clustered using Canvas [37, 38], based on fingerprint similarity using Daylight invariant atom types; bonds were distinguished by bond order and cyclic aliphatic bonds were distinguished from acyclic aliphatic bonds. Twelve clusters were obtained this way, out of which six were used in the subsequent study; three clusters with acetylenic linker containing 91 compounds (Table S1), two clusters with fenobam analogues containing 63 compounds (Table S2) and one cluster with HTL-14242 analogues containing 36 compounds (Table S3). Other clusters of actives were not used as they were structurally different from the compounds whose complex structures were used for docking. 50 decoy molecules were generated for each active ligand using DUD-E [31, 32]. The active ligands and decoys were prepared for docking with the default options of Ligprep (Schrödinger Suite, 2018-1 [39] and using Maestro (version: 11.5.011) [40]). The proteins were prepared using default settings of Schrödinger Protein Preparation Wizard [39, 41], that includes the optimization of the hydrogen bond network.

Ligands were removed from the complexes and the empty protein cavities were solvated with WaterFLAP using iterative water hotspot identification with molecular interaction field analysis (GRID) [42, 43] and short molecular dynamics simulation (WaterFLAP version 2.2.1). Waters clashing with the ligands of the complex were removed and the remaining water molecules were optimized by water perturbation analysis within WaterFLAP.

The molecular docking was performed with Glide [44] using standard precision and default settings. Glide’s docking score was used to rank compounds and virtual screening results were evaluated by calculating enrichments factors using KNIME [45]. Enrichment factors of the top scored 1% compounds are calculated as EF1% = [TP1%/(TP1% + FP1%)]/[ACTIVE_TOT_/(ACTIVE_TOT_ + DECOY_TOT_)], where TP1% and FP1% are the number of true positives (actives) and the number of false positives (decoys), respectively, in the top 1% of the ranked ligand set, ACTIVE_TOT_ and DECOY_TOT_ are the total number of actives and decoys, respectively, in the database.

## Results and discussion

**Fig. 3:**
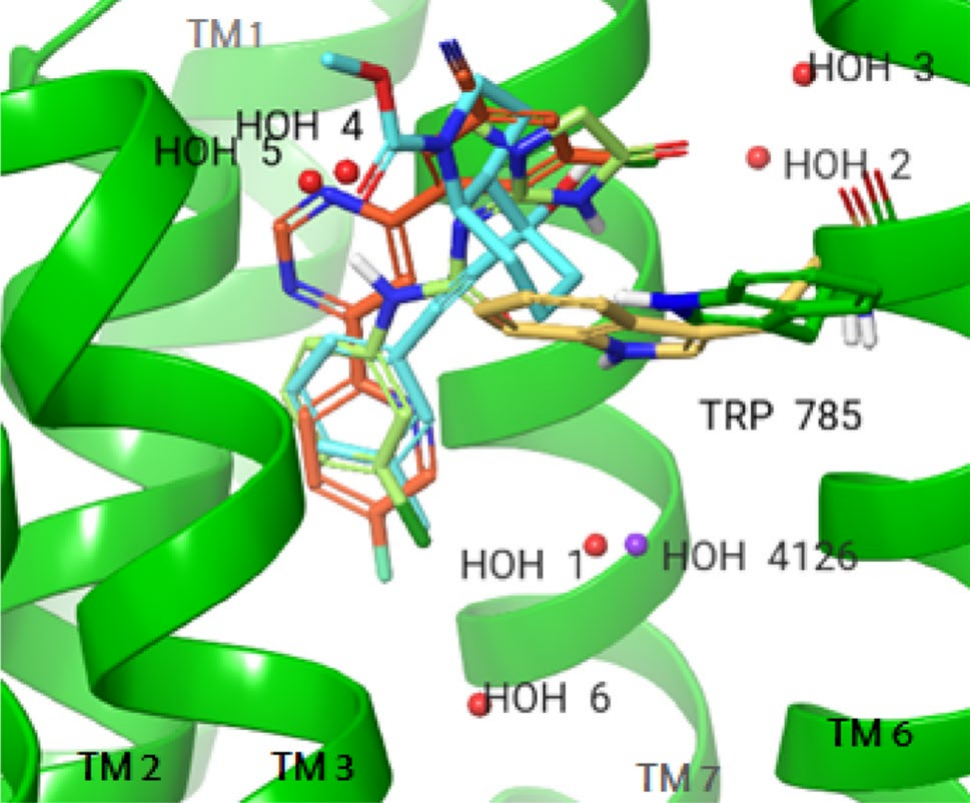
mGlu_5_ receptor binding pocket with mavoglurant (cyan), HTL-14242 (orange) and fenobam (light green). Water molecules generated by WaterFLAP (HOH1-6) and used in the docking studies are shown as red balls and X-ray water (HOH 4126) is shown as purple ball. Trp785^6.50c^ that adopts different conformation in the mavoglurant complex (shown as green sticks) as compared to the HTL-14242 and fenobam complexes (shown as yellow sticks) is also shown

Virtual screening was performed using protein structures both without water molecules and with various sets of water molecules. The protein structures were derived from the mGlu_5_ receptor complexes with mavoglurant (PDB ID: 4OO9, res.: 2.6 Å) [7], HTL-14242 (PDB ID: 5CGD, res.: 2.603 Å) [8] and fenobam (PDB ID: 6FFH, res.: 2.65 Å) [9]. Water molecules were generated by WaterFLAP. It was found that WaterFLAP well reproduces the position of crystallographic waters and, in addition, it predicts the presence of additional water molecules. Some of these waters are close to the ligands and are expected to directly affect ligand binding and to influence virtual screening. Waters used in each complex are shown in Fig. 3. Water HOH 1 generated by WaterFLAP is close to the water molecule that is consistently present in all available X-ray structures (HOH 4126 in 4OO9 crystal structure; see Fig. 3). It is worth noting that WaterFLAP predicts that the free-energy of this water decreases upon ligand binding.

We identified five water molecules (waters 1, 2, 3, 5 and 6) in the mavoglurant-mGlu_5_ receptor complex within 4 Å separation from the ligand out of which four were common in all three crystal structures (waters 1–3, and 6). Water 5 is shared with the fenobam, but not with the HTL-14242 complex since in the latter it is displaced by the pyrimidine ring that occupies a position not filled in the mavoglurant and fenobam complexes. Water 4 is present in the fenobam and HTL-14242 complexes, but it is not found in the mavoglurant complex since it is replaced by the carboxylate substituent. Thus, five water sites are identified in the mavoglurant and HTL-14242, and six water sites are identified in the fenobam complex derived protein.

Virtual screening studies were performed with the protein structures obtained from the three complexes after the removal of the ligands and all crystallographic water molecules. In addition to these structures, further structures were created by adding water molecules generated by WaterFLAP around the ligands in various combinations. In the first step one water molecule was added to the empty structures. Five structures with one water molecules were obtained for the mavoglurant and the HTL-14242, and six structures were obtained for the fenobam complex derived protein structure. In the next step, the three single water containing structures, each derived from different complexes and best performing in docking studies were selected and a further water molecule was added in each possible position shown in Fig. 3. Structures with three water molecules were prepared in the same way. This procedure resulted in one empty and fourteen water containing structures from the mavoglurant complex derived protein. In this case, structures with water pairs 6-1 and 6-5 performed similarly (see below) and both were used to generate structures with three water molecules. Fifteen water containing structures were generated for fenobam where six water positions were identified. Finally, five water containing structures were generated for HTL-14242. Owing to the good performance of the single water containing structures no further water molecules were added. Altogether, 37 virtual screenings were carried out using 3 structures without water and 34 structures with various number of water molecules.

### Virtual screening with mavoglurant–mGlu_5_ receptor complex derived protein structures

Virtual screening was performed for the empty protein and for structures with one, two and three waters. Receiver operating characteristics curves are depicted in Fig. S1, enrichment factors (EF) for the top 1%, 2% and 5% ranked compounds and other characteristic values of the virtual screening are shown in Table 1.

**Table 1:** Number of docked compounds (# docked), enrichment factors (EF), number of false positives (FP), false negatives (FN), true positives (TP) and true negatives (TN) for the mavoglurant–mGlu_5_ receptor complex derived protein structures with varying set of water molecules

Mavoglurant																
Water	# docked	EF			FP			FN			TP			TN		
		1%	2%	5%	1%	2%	5%	1%	2%	5%	1%	2%	5%	1%	2%	5%
–	4573	9^3^	8^2^	4^1^	40	82	224	83	77	73	8	14	18	4710	4668	4526
1	4584	9^3^	7^2^	4^1^	40	83	223	83	78	72	8	13	19	4710	4667	4527
2	4584	10^3^	6^2^	5^1^	39	85	221	82	80	70	9	11	21	4711	4665	4529
3	4586	10^3^	7^2^	4^1^	39	83	223	82	78	72	9	13	19	4711	4667	4527
5	4515	10^3^	6^2^	4^1^	39	85	224	82	80	73	9	11	18	4711	4665	4526
6	4584	11^3^	8^2^	4^1^	38	82	223	81	77	72	10	14	19	4712	4668	4527
6-1	4578	11^3^	7^2^	4^1^	38	83	223	81	78	72	10	13	19	4712	4667	4527
6-2	4582	9^3^	7^2^	5^1^	40	84	219	83	79	68	8	12	23	4710	4666	4531
6-3	4597	10^3^	7^2^	4^1^	39	84	222	82	79	71	9	12	20	4711	4666	4528
6-5	4514	11^3^	7^2^	4^1^	38	83	222	81	78	71	10	13	20	4712	4667	4528
6-5-1	4515	11^3^	7^2^	4^1^	38	84	224	81	79	73	10	12	18	4712	4666	4526
6-5-2	4513	9^3^	7^2^	4^1^	40	84	223	83	79	72	8	12	19	4710	4666	4527
6-5-3	4514	8^2^	7^2^	4^1^	41	83	223	84	78	72	7	13	19	4709	4667	4527
6-1-2	4593	10^3^	6^2^	4^1^	39	85	222	82	80	71	9	11	20	4711	4665	4528
6-1-3	4597	11^3^	8^2^	5^1^	38	82	220	81	77	69	10	14	22	4712	4668	4530

Values for the top scored 1%, 2% and 5% are shown. Standard deviations of enrichment factors are calculated according to Ref. [46] and are shown as superscripts

The mavoglurant structures are able to accommodate over 90% of the compounds in the database. The enrichment factor for the empty mavoglurant structure is 8.9 and this is slightly lower than the enrichment factors obtained with most of the water containing structures. The presence of any single water molecule has a small effect on the enrichments observed for the top-scored 1, 2 and 5% compounds. Water at position 6 was selected for combining it with other water molecules. No significant improvement in the enrichment factors were observed either with two or three water molecules. The structures with two water molecules in the 6-1 and 6-5 positions performed similarly, and both were used in generating structures with three water molecules. Considering EF1%, EF2% and EF5% values, water combination 6-1-3 was found to be the best, slightly outperforming other mavoglurant complex derived structures.

### Virtual screening with fenobam–mGlu_5_ receptor complex derived protein structures

The fenobam complex derived protein structure contains the Trp785^6.50^ sidechain pointing inwards the helical bundle. Therefore, these structures have smaller allosteric site than do mavoglurant derived structures. Receiver operating characteristics curves are depicted in Fig. S2, enrichment factors for the top 1%, 2% and 5% ranked compounds and other characteristic values of the virtual screening are shown in Table 2.

**Table 2:** Number of docked compounds (# docked), enrichment factors (EF), number of false positives (FP), false negatives (FN), true positives (TP) and true negatives (TN) for the fenobam–mGlu_5_ receptor complex derived protein structures with varying set of water molecules

Fenobam																
Water	# docked	EF			FP			FN			TP			TN		
		1%	2%	5%	1%	2%	5%	1%	2%	5%	1%	2%	5%	1%	2%	5%
–	1742	0	2^1^	3^1^	32	62	151	63	61	54	0	2	9	3118	3088	2999
1	1675	2^2^	2^1^	2^1^	31	62	153	62	61	56	1	2	7	3119	3088	2997
2	1759	0	2^1^	3^1^	32	61	151	63	60	54	0	3	9	3118	3089	2999
3	1754	0	2^1^	4^1^	32	61	148	63	60	51	0	3	12	3118	3089	3002
4	1401	0	2^1^	2^1^	32	61	155	63	60	58	0	3	5	3118	3089	2995
5	1155	13^3^	10^2^	5^1^	24	51	144	55	50	47	8	13	16	3126	3099	3006
6	1679	3^2^	2^1^	3^1^	30	61	152	61	60	55	2	3	8	3120	3089	2998
5-1	1124	13^3^	10^2^	5^1^	24	52	143	55	51	46	8	12	17	3126	3098	3007
5-2	1163	13^3^	10^2^	5^1^	24	52	144	55	51	47	8	12	16	3126	3098	3006
5-3	1175	16^3^	10^2^	5^1^	22	51	143	53	50	46	10	13	17	3128	3099	3007
5-4	856	8^3^	4^2^	2^1^	27	59	153	58	58	56	5	5	7	3123	3091	2997
5-6	1148	13^3^	10^2^	5^1^	24	52	144	55	51	47	8	12	16	3126	3098	3006
5-3-1	1141	16^3^	10^2^	5^1^	22	51	144	53	50	47	10	13	16	3128	3099	3006
5-3-2	1172	11^3^	10^2^	5^1^	25	52	143	56	51	46	7	12	17	3125	3098	3007
5-3-4	872	8^3^	4^2^	3^1^	27	59	151	58	58	54	5	5	9	3123	3091	2999
5-3-6	1172	14^3^	12^2^	5^1^	23	49	143	54	48	46	9	15	17	3127	3101	3007

Values for the top scored 1%, 2% and 5% are shown. Standard deviations of enrichment factors are calculated according to ref [46] and are shown as superscripts

The protein is able to accommodate around 50% of the compounds and this is in contrast to the 90% obtained with the mavoglurant complex derived structures. Although both actives and decoys are similar to fenobam [32], the shape and size of the allosteric site and, in particular, the position of Trp785^6.50^ that points inward, limits the number of successfully docked compounds. Docking into the empty protein could not identify any active among the top ranked 1%, however, the presence of water 1 and 6, and especially water 5 improves the performance. The combination of water 5 with other water molecules, except with water 4, gives double digit enrichment. The 5-3 combination gives consistently the highest enrichments at 1, 2 and 5% ranked compounds. Inclusion of a third water molecule also yields good enrichment factors, however, they are not superior to the 5-3 pair, except the 5-3-6 combination at 2% ranked compounds with an enrichment factor of 12.

### Virtual screening with HTL-14242–mGlu_5_ receptor complex derived protein structures

Results obtained for structures derived from the HTL-14242 complex are presented in Table 3 and in Fig. S3.

**Table 3:** Number of docked compounds (# docked), enrichment factors (EF), number of false positives (FP), false negatives (FN), true positives (TP) and true negatives (TN) for the HTL-14242–mGlu_5_ receptor complex derived protein structures with varying set of water molecules

HTL-14242																
Water	# docked	EF			FP			FN			TP			TN		
		1%	2%	5%	1%	2%	5%	1%	2%	5%	1%	2%	5%	1%	2%	5%
–	1484	51	31^2^	16^1^	0	14	63	18	14	8	18	22	28	1800	1786	1737
1	1486	48^1^	33^2^	16^1^	1	13	62	19	13	7	17	23	29	1799	1787	1738
2	1490	48^1^	31^2^	17^1^	1	14	63	19	14	8	17	22	28	1799	1786	1737
3	1494	48^1^	34^2^	16^1^	1	12	61	19	12	6	17	24	30	1799	1788	1739
4	1415	51	35^1^	16^1^	0	11	62	18	11	7	18	25	29	1800	1789	1738
6	1485	48^1^	34^1^	16^1^	1	12	62	19	12	7	17	24	29	1799	1788	1738

Values for the top scored 1%, 2% and 5% are shown. Standard deviations of enrichment factors are calculated according to ref [46] and are shown as superscripts

**Fig. 4:**
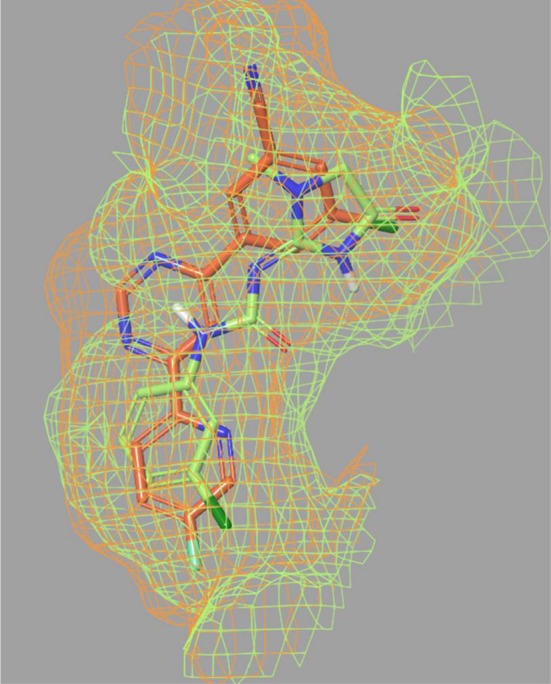
Shape of the binding sites of fenobam (light green) and HTL-14242 (orange) complexes

The total number of successfully docked compounds is around 80% of the total database irrespective of the number of water molecules included. This is smaller than it was seen for mavoglurant and somewhat higher than it was observed for fenobam. The differences can be partly attributed to the different conformation of Trp785^6.50^ in the mavoglurant compared to the fenobam and HTL-14242 complexes. The latter structures share “swung-in” Trp785^6.50^ conformation that yields a significant reduction of the pocket size. The conformational change of this conserved Trp residue contributes to the activation of several class A GPCRs [47, 48] while both conformations appear to be accessible in mGlu_5_ PAM complexes [29, 30], and the conformation is ligand dependent in mGlu_5_ NAM complexes [7–9]. However, Trp785^6.50^ is not the only determinant of the size and the shape of the allosteric pocket. Fenobam and HTL-14242 pockets differ in shape (Fig. 4) owing to the different orientation of Ser654^3.39^ and Val806^7.36^ side chains. Besides the large number of successfully docked molecules, a high enrichment of 51 in the top 1% of the ranked compounds was achieved with the empty HTL-14242 structure. This is the maximum enrichment that can be obtained at 1%, as all compounds in the first 1% are active. Some improvements in the EF2% and EF5% values were observed with the addition of a water molecule. The best early enrichments are achieved with water 4, with a maximal available EF1%, the highest EF2% and a near to highest EF5%. Since the docking results both without water and with a single water molecule are close to the optimum, no further water combinations were tested. It has to be noted, that the very high enrichments in this case can be attributed to the high similarity of the active compounds that all belong to the series investigated in a structure–activity relationship study around HTL-14242. However, the successful docking of 80% of the compounds shows that the outstanding enrichment is not the consequence of having decoys not accommodated in the allosteric pocket, rather it is the consequence of the efficient distinction between active and inactive compounds by the docking-scoring procedure.

The separation of ligands and using an appropriate protein structure with optimally selected water molecules outperforms the conventional approach. Virtual screening campaigns with all ligands using any of the three protein structures give vastly inferior early enrichments (Table S4). Ligands assigned to each protein structure may belong to several clusters as it is the case for mavoglurant (3 clusters) and fenobam analogues (2 clusters). The analysis of virtual screening performances separately for each cluster (Tables S5–7) provides indications on how much similarity to the X-ray ligands is needed for a successful virtual screening. As an example, actives in cluster 12 are more similar to mavoglurant than those of cluster 11 (median similarity 0.532 vs. 0.078), and enrichment factors of cluster 12 are superior to those of cluster 11. This suggests that similarity information is useful in guiding prospective virtual screening applications.

A detailed analysis of the water molecules yielding improved VS performance revealed that they are ligand dependent, and their common feature is the presence of hydrogen bonds to the ligands. Mavoglurant forms no hydrogen bond to any of the water molecules, and a modest increase of enrichment factors was achieved compared to the empty protein structure. HTL-14242 gave high enrichment factors already with the empty protein, and the EF2% enrichment was further improved in the presence of HOH 4, a water molecule hydrogen bonded to HTL-14242 and also to Ile651^3.36^ and Asn747^5.47^. The highest improvement of enrichments for fenobam was observed with the addition of HOH 5 (EF1% increased from 0 to 12.8), and this was further increased when both HOH 5 and HOH 3 were added. HOH 5 is hydrogen bonded to fenobam and to Ile651^3.36^, and HOH 3 forms hydrogen bonds to fenobam and to both Ser805^7.36^ and HOH 2. Other water molecules having smaller effect on enrichments are not hydrogen-bonded to fenobam. The HOH 1 water molecule consistently observed in all mGlu_5_ receptor X-ray structures does not form hydrogen-bond with any of the ligands and its presence was not found to improve virtual screening performance. Although this water molecule appears to contribute to the structural stability of the receptor and even more to its complexes as suggested by WaterFLAP calculations, it does not have direct interactions with the ligands investigated and its presence does affect virtual screening performance. The observations suggest a guideline for selecting water molecules in the virtual screening of the proposed protocol; waters hydrogen-bonded to the ligand are most likely to beneficially affect enrichment factors, and the number and position of these water molecules are ligand dependent.

**Fig. 5:**
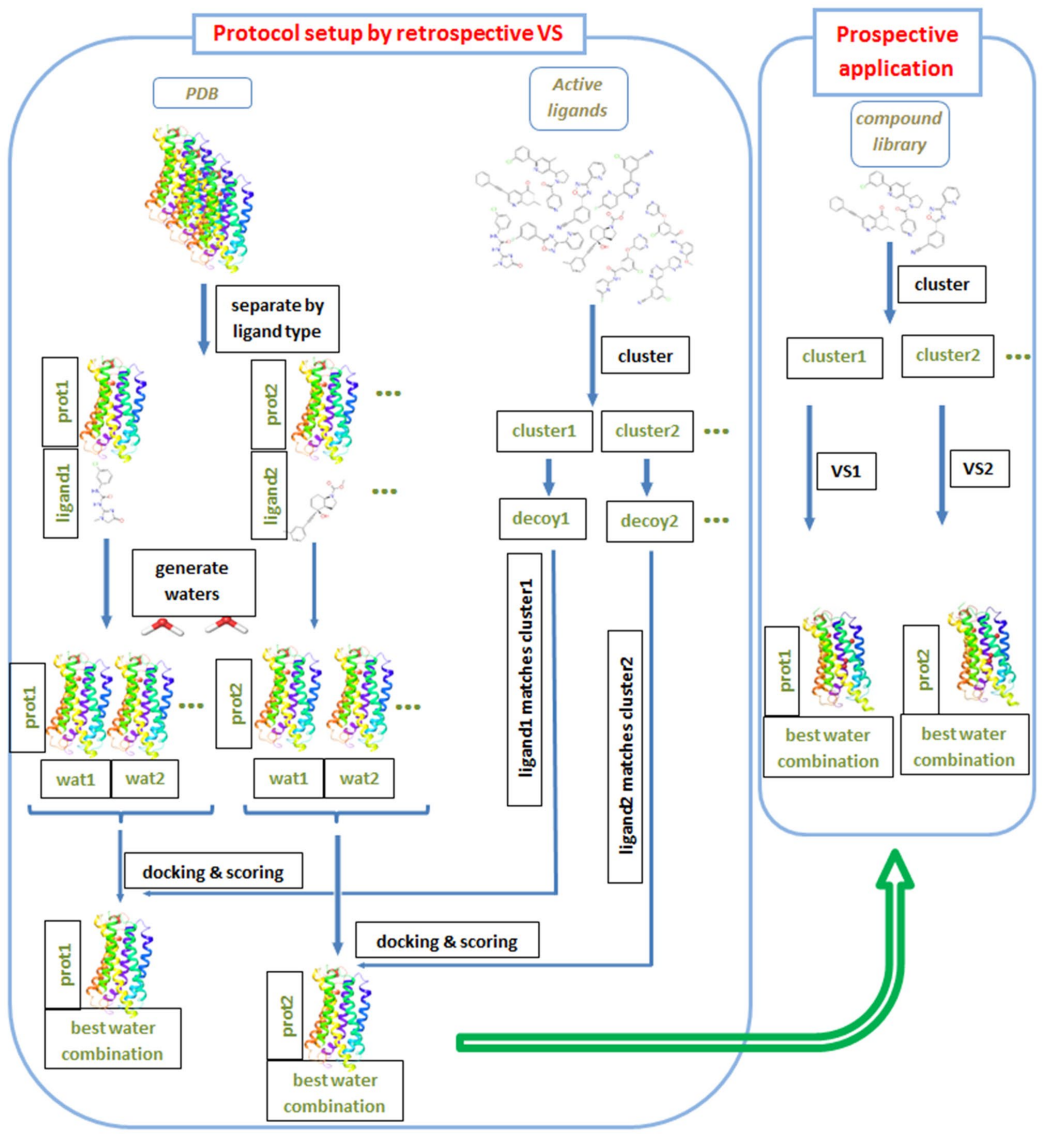
Protocol proposed for virtual screening of allosteric GPCR sites

Based on the above results a virtual screening protocol is proposed that has two key elements to assure that both protein flexibility and the role of water molecules are well accounted for. It includes the separation of the ligands into compound series and docking each series into a distinct protein structure having appropriate conformation to accommodate the particular series of compounds. Restricting compounds to those structurally related to a bound ligand of the protein conformation used for docking decreases false negative rates, while using a single protein structure for a particular compound series decreases false positive rates. The use of an experimental protein structure complexed with a ligand structurally close to the compounds to be docked is reminiscent to self-docking, where a ligand is docked into the experimental protein structure of its complex. In contrast, in cross-docking, a compound is docked into a protein structure extracted from the complex with another ligand. The better performance of self-docking over cross-docking is well-established [49, 50] and it is more pronounced for scoring and ranking than for docking accuracy [51]. Protein flexibility was shown to increase the difficulty of selecting the appropriate protein structure for successful docking [52, 53], and the excessive sensitivity of the mGlu_5_ receptor binding pocket on the bound ligand calls for the judicious selection of mGlu_5_ target structures. The other key element of the procedure is to place computationally generated water molecules into the binding pocket. The evaluation of retrospective virtual screenings performed with various combinations of water molecules informs us on the optimal arrangement of water molecules. The proposed protocol is depicted in Fig. 5.

It was demonstrated above that separating ligands into series and docking them into distinct protein structures with selected water molecules yields good early enrichments. The separation of ligands into structurally similar series is motivated by the induced fit binding to the mGlu_5_ receptor observed in X-ray structures. Although induced fit binding to proteins is frequently observed, mGlu_5_ receptor appears to be extremely sensitive to small structural variations of the ligand as it is exemplified by the binding of mavoglurant and M-MPEP. These two compounds are close analogues (Fig. 1), nevertheless, their cross-docking (the docking of M-MPEP into the protein structure that binds mavoglurant and vice versa) fails to straightforwardly reproduce the experimental complexes. Another feature of mGlu_5_ receptor is the presence of a functional water network in the allosteric site. Although the effect of binding site water molecules on ligand binding is generally observed, this appears to be enhanced in the allosteric site of mGlu_5_ receptor owing to its narrow shape and well structured, functionally relevant water network. Therefore, the presence and the interactions of the water molecules have profound effect on ligand binding, and this has to be taken into account in the structure based virtual screening of the mGlu_5_ receptor.

The effect of protein flexibility and the interaction with water molecules on ligand binding is a general feature of GPCR allosteric sites. Experimental structural data obtained with and without bound allosteric ligands show that these sites are sensitive to protein conformational changes and to interactions with water molecules. Therefore, the proposed protocol that is able to account for these effects is expected to find use in the virtual screening of a wide range of GPCR allosteric sites.

## Conclusion

Our analysis focused on the virtual screening performance of mGlu_5_ receptor protein structures derived from complexes with negative allosteric modulators having varying scaffolds. Important factors in ligand binding to mGlu_5_ receptor is protein flexibility and interactions with the water network in the allosteric pocket. Protein flexibility results in induced fit binding and this necessitates the separation of ligands into structurally similar series and docking each series to a protein conformation derived from a complex with a member of the series. We considered three ligands with available mGlu_5_ receptor X-ray structures. One of the compounds, mavoglurant, contains an acetylenic linker and another, fenobam, contains a urea linker both render the ligands linear. The third ligand, HTL-14242, has a heterocyclic linker that results in a bent shape. Docking compound series into the corresponding protein conformation gave varying enrichments. We found that the inclusion of water molecules in the docking protocol is able to improve the docking performance for all structures studied. This site is a functional water channel and the interaction of the waters with the protein, with the ligand and with each other contribute importantly to the ligand binding. Therefore, the inclusion of waters in the protein structure is beneficial for the performance of the virtual screening.

The experience gained with the retrospective hit identification by virtual screening can be translated to a virtual screening protocol to identify allosteric ligands for the mGlu_5_ receptor. The principal elements of the protocol are the use of several protein structures derived from complexes of ligands with distinct shape or chemotype and to identify water positions by optimizing the performance of small scale retrospective virtual screening calculations. The protocol allows the extension of available set of allosteric ligands and to find novel chemical starting points for mGlu_5_ receptor. The protocol takes into account protein flexibility and the presence of water molecules in the binding site and is expected to be useful for other systems where these effects are important.

## Electronic supplementary material

Below is the link to the electronic supplementary material.

Supplementary material 1 (DOCX 5169 kb)
