# Supplementary material for: The role of water and protein flexibility in the structure-based virtual screening of allosteric GPCR modulators: an mGlu5 receptor case study
Source: J Comput Aided Mol Des. 2019 Sep 21;33(9):787–97. doi: 10.1007/s10822-019-00224-w (PMC6825653; doi:10.1007/s10822-019-00224-w)
Supplement: Supplementary file 1 — Supplementary material 1 (DOCX 5169 kb) [file 10822_2019_224_MOESM1_ESM.docx]

Supplementary Information

The role of water and protein flexibility in the structure-based virtual screening of allosteric GPCR modulators: an mGlu5 receptor case study

Zoltán Orgován, György G. Ferenczy, György M. Keserű*

Table S1 Acetylenic linker containing compounds used in the virtual screening

| Acetylenic linker containing compounds | | |
| --- | --- | --- |
| Structure | Cluster Number | Activite / Inactive |
| CC1=CC=CC(=N1)C#CC1=CC=CC=C1 | 10 | A |
| CC1=NC(=CS1)C#CC1=CC=C(N=C1)C1=CC=CN=C1 | 10 | A |
| CC1=NC(=CS1)C#CC1=CC=CN=C1 | 10 | A |
| CC1=CSC(=N1)C#CC1=CC=CN=C1 | 10 | A |
| O=C1CCCC2=C1C=C(C=N2)C#CC1=CC=CC=C1 | 10 | A |
| O=C1CCCC2=C1C=CC(=N2)C#CC1=CC=CC=C1 | 10 | A |
| O=C(NC1CCCC1)C1=CC=C(N=C1)C#CC1=CC=CC=N1 | 10 | A |
| CC1CC(=O)C2=C(C1)N=CC(=C2)C#CC1=CC=CC=C1 | 10 | A |
| CC1(C)CC(=O)C2=C(C1)N=CC(=C2)C#CC1=CC=CC=C1 | 10 | A |
| CC1(C)CCC2=C(C=CC(=N2)C#CC2=CC=CC=C2)C1=O | 10 | A |
| CN1CCC2=C(C=CC(=N2)C#CC2=CC=CC=C2)C1=O | 10 | A |
| CN1CCC2=C(C=CC(=N2)C#CC2=CC=CC(C)=C2)C1=O | 10 | A |
| CN1CCC2=C(C=CC(=N2)C#CC2=CC=CC=C2C)C1=O | 10 | A |
| CN1CCC2=C(C=CC(=N2)C#CC2=CC=C(Cl)C=C2)C1=O | 10 | A |
| CN1CCC2=C(C=CC(=N2)C#CC2=CC=CC(Cl)=C2)C1=O | 10 | A |
| CN1CCC2=C(C=CC(=N2)C#CC2=CC=CC=C2Cl)C1=O | 10 | A |
| CC(C)N1CCC2=C(C=CC(=N2)C#CC2=CC=CC=C2)C1=O | 10 | A |
| COC1=CC=CC(=C1)C#CC1=CC=C2C(CCN(C)C2=O)=N1 | 10 | A |
| CC(C)N1CCC2=C(C=CC(=N2)C#CC2=CC=CC(C)=C2)C1=O | 10 | A |
| CC(C)N1CCC2=C(C=CC(=N2)C#CC2=CC=CC=C2C)C1=O | 10 | A |
| CC(C)N1CCC2=C(C=CC(=N2)C#CC2=CC=CC(Cl)=C2)C1=O | 10 | A |
| COC1=CC=CC(=C1)C#CC1=CC=C2C(CCN(C(C)C)C2=O)=N1 | 10 | A |
| CN1CCC2=C(C=CC(=N2)C#CC2=CC=CC=N2)C1=O | 10 | A |
| CC(C)N1CCC2=C(C=CC(=N2)C#CC2=CC=CC=N2)C1=O | 10 | A |
| CN1CCC2=C(C=CC(=N2)C#CC2=CC=CS2)C1=O | 10 | A |
| O=C1N(CCC2=C1C=CC(=N2)C#CC1=CC=CC=N1)C1CCCCC1 | 10 | A |
| O=C1N(CCC2=C1C=CC(=N2)C#CC1=CC=CC=N1)C1CCCC1 | 10 | A |
| CC(C)N1CCC2=C(C=CC(=N2)C#CC2=CC=CS2)C1=O | 10 | A |
| CC(C)CN1CCC2=C(C1)SC(=N2)C#CC1=CC=CC=C1 | 10 | A |
| CCN1CCC2=C(C1)SC(=N2)C#CC1=CC=CC(F)=C1 | 10 | A |
| CC(C)S(=O)(=O)N1CC2=C(C1)N=C(S2)C#CC1=CC=CC=C1 | 10 | A |
| CC(=O)N1CCC2=C(C1)SC(=N2)C#CC1=CC=CC=C1 | 10 | A |
| CCC(=O)N1CCC2=C(C1)SC(=N2)C#CC1=CC=CC=C1 | 10 | A |
| CC(C)S(=O)(=O)N1CCC2=C(C1)SC(=N2)C#CC1=CC=CC=C1 | 10 | A |
| CN(C)S(=O)(=O)N1CCC2=C(C1)SC(=N2)C#CC1=CC=CC=C1 | 10 | A |
| O=C1N(CC2=C1SC(=N2)C#CC1=CC=CC=C1)C1CC1 | 10 | A |
| FC1=CC=CC(=C1)C#CC1=NC2=C(CN(CC2)C2CCC2)S1 | 10 | A |
| FC1=CC=CC(=C1)C#CC1=NC2=C(CN(CC2)C2CCCC2)S1 | 10 | A |
| FC1=CC=CC(=C1)C#CC1=NC2=C(CN(CC2)C2CCOC2)S1 | 10 | A |
| O=C(C1CC1)N1CCC2=C(C1)SC(=N2)C#CC1=CC=CC=C1 | 10 | A |
| O=S(=O)(C1CC1)N1CCC2=C(C1)SC(=N2)C#CC1=CC=CC=C1 | 10 | A |
| O=S(=O)(N1CCCC1)N1CCC2=C(C1)SC(=N2)C#CC1=CC=CC=C1 | 10 | A |
| O=S(=O)(N1CCCCC1)N1CCC2=C(C1)SC(=N2)C#CC1=CC=CC=C1 | 10 | A |
| O=C(C1CCC1)N1CCC2=C(C1)SC(=N2)C#CC1=CC=CC=C1 | 10 | A |
| O=S(=O)(N1CCOCC1)N1CCC2=C(C1)SC(=N2)C#CC1=CC=CC=C1 | 10 | A |
| O=S(=O)(C1CCCCC1)N1CCC2=C(C1)SC(=N2)C#CC1=CC=CC=C1 | 10 | A |
| CSC1=CC=C(C=C2C(=O)N(C)C(=S)N(C)C2=O)C=C1 | 10 | I |
| CN1[C@@H]([C@H](C(C)=O)C(=O)C1=O)C1=CC=C(Br)C=C1 | 10 | I |
| CN(C)C(=O)C1=CC=C2CCC3=CC=C(C(=O)N(C)C)C1=C23 | 10 | I |
| CN(C(=O)C1=CC=C(F)C=C1)[C@@]1(C)CCS(=O)(=O)C1 | 10 | I |
| CS(=O)(=O)C1=CC2=CC=C(C=C2C=C1)S(C)(=O)=O | 10 | I |
| CN1C(=S)S\C(=C\C2=CC=C3OCCOC3=C2)C1=O | 10 | I |
| C[C@@H](N1C=C(Br)C=N1)C(=O)N1CCCC1 | 10 | I |
| O=C1[C@H]2[C@H]([C@@H]3C=C[C@H]2C32CC2)C(=O)N1C[C@@H]1CCCO1 | 10 | I |
| CN1CCN(CC1)S(=O)(=O)C1=CC=C(F)C(F)=C1F | 10 | I |
| CCN1C=C(Br)C=C1C(=O)N1CCCC1=O | 10 | I |
| FC1=CC=C(CN2CC(=O)N3CSC[C@H]3C2=O)C=C1 | 10 | I |
| CC1(C)[C@@]2(C)CC[C@@]1(OC2=O)C(=O)N1CCCCCC1 | 10 | I |
| C[C@@H]1CN([C@@H](C)CO1)C(=O)C1=CC(Br)=CN=C1 | 10 | I |
| CN1C2=C(CN(C)CC2)C2=CC(=CC=C12)S(C)(=O)=O | 10 | I |
| CC(C)[C@@H](Br)C(=O)N1CCN2C=CN=C2C1 | 10 | I |
| O=S1(=O)CCN(CN2C=CC(=S)C3=CC=CC=C23)CC1 | 10 | I |
| FC1=C(C=C(C=C1)S(=O)(=O)N1CCSCC1)C#N | 10 | I |
| C[C@@H]1CN([C@H](C)CO1)C(=O)C1=COC(Br)=C1 | 10 | I |
| C[C@H]1C[C@@H](SC2=NC(C)=NC3=CC=CC=C23)C(=O)O1 | 10 | I |
| CN([C@@H]1CCN(C)C1=O)C(=O)[C@]1(C)CC1(Cl)Cl | 10 | I |
| ICCN1N=C2C=CC=CN2C1=O | 10 | I |
| CC1=C(CBr)C=NN1[C@@H]1CCS(=O)(=O)C1 | 10 | I |
| CC(C)[C@H](Br)C(=O)N1CCO[C@H](C1)C#N | 10 | I |
| CCN1C=C(I)C(=O)N(C)C1=O | 10 | I |
| BrC1=CN=CN(CC2=NC=CS2)C1=O | 10 | I |
| CN1N=CN=C1CN1[C@H]2CC[C@@H]1CC(Br)C2 | 10 | I |
| O=C1CCCC[C@]1(C#N)[C@@H]1CS(=O)(=O)C2=CC=CC=C12 | 10 | I |
| C[C@H]1CO[C@@H](CBr)CN1C1=CC=NC=N1 | 10 | I |
| C[C@@H]1CC[C@H](O1)C(=O)N1CCO[C@H](CBr)C1 | 10 | I |
| C[C@H]1[C@@H](C)S(=O)(=O)CCN1C(=O)C1=CSC=C1C | 10 | I |
| CC1(C)CN(CCS1(=O)=O)C(=O)C[C@H]1CCC=C1 | 10 | I |
| C[C@@H](C#N)N1CCN(CC1)C(=O)C1=CC=C(F)C(F)=C1F | 10 | I |
| C[C@]1(CCS(=O)(=O)C1)N1CCN2CCCC[C@@H]2C1 | 10 | I |
| C[C@H]1N(CC2=CC=C(F)C(F)=C2)C(=O)[C@H]2CCCN2C1=O | 10 | I |
| FC1=C(F)C=C(CN2CCC(=O)N3CCC[C@@H]3C2=O)C=C1 | 10 | I |
| CC1=C(C)C=C(CN2CC(=O)N3CCC[C@H]3C2=O)S1 | 10 | I |
| C[C@@]1(CCS(=O)(=O)C1)N1CCCN2CCC[C@@H]2C1 | 10 | I |
| C([C@@H]1CN2CCC[C@@H]2CO1)N1CCCN2CCC[C@@H]2C1 | 10 | I |
| O=C([C@H]1CCO[C@@]2(CCOC2)C1)[C@@H]1CSCCO1 | 10 | I |
| O=C([C@@H]1CCOC2(CCSCC2)C1)[C@@H]1CN2CCN1CC2 | 10 | I |
| FC1=NC(F)=C(F)C(N2CCN3C=CN=C3C2)=C1F | 10 | I |
| BrC1=CN=C(S1)N1CCS(=O)(=O)CC1 | 10 | I |
| CC1=CSC=C1CN1CCC(=O)N2CCCC[C@H]2C1=O | 10 | I |
| CN1CCCN(C)CCN(S)CCCN(C)CC1 | 10 | I |
| COC1=CC(OC)=C2N=NC(I)=C2C1 | 10 | I |
| ClC1=N[C@H]2N=NC(I)=C2C=N1 | 10 | I |
| BrC1=CN2C=NC=N[C@@H]2C(Br)=N1 | 10 | I |
| C[C@H]1C[C@H](C)[C@H](CN2CCS(=O)(=O)CC2)C(=O)C1 | 10 | I |
| CN1C=C(C(=O)N2CCCSCC2)C(=N1)C(F)(F)F | 10 | I |
| BrC1=CN=CC(=C1)S(=O)(=O)N1CCC1 | 10 | I |
| CN(C)C(=O)C1=C(C)C(Cl)=C(C)N=C1Cl | 10 | I |
| BrC1=CC(Br)=NC=N1 | 10 | I |
| FC1=CC=CC=C1CN1C(=O)CSC1=S | 10 | I |
| BrC1=CN=C(Br)C=N1 | 10 | I |
| SC(=S)N1CCN(CC1)C(S)=S | 10 | I |
| COC1=CC=NC(I)=N1 | 10 | I |
| CN1CCN(CC1)C(=O)[C@@]1(C)CC1(Cl)Cl | 10 | I |
| FC1=CC=C(C=C1)C(=O)N1CCSC1=S | 10 | I |
| CC1=NC2=CC(Br)=CC=C2C(=O)O1 | 10 | I |
| BrC1=CN(N=C1)[C@@H]1CCC[C@@H]1C#N | 10 | I |
| BrC1=CC(Br)=NN=C1 | 10 | I |
| O=C1N=C(N=C2S[C@H]3CCCCC3=C12)C1CC1 | 10 | I |
| BrC1=NC(=CN2C=CN=C12)C1CC1 | 10 | I |
| COC1=CC(F)=CC2=C1N=CC(C#N)=C2Cl | 10 | I |
| CC1=CC(=O)N(C1=O)C1=CC(F)=C(F)C=C1F | 10 | I |
| O=C1OCC[C@H]1N1CCC2(CCCCC2)CC1 | 10 | I |
| Cl[C@@H]1CCOC2(CCS(=O)(=O)CC2)C1 | 10 | I |
| C[C@@H](CCl)C(=O)N1C[C@@H](C)S[C@H](C)C1 | 10 | I |
| CC[C@H](C)N1CCC[C@@H](Br)C1=O | 10 | I |
| CC(C)(C)N1CCC[C@@H](Br)C1=O | 10 | I |
| CN(C)C(=O)C1=C(Br)C=CS1 | 10 | I |
| Br[C@H]1CCC[C@H](C1)C1=CN=CN=C1 | 10 | I |
| O=C1O[C@@H]2[C@H]3C[C@H](C[C@@H]13)[C@H]2SC1=CC=CC=C1 | 10 | I |
| BrC1=NN=C(S1)N1CCCC1 | 10 | I |
| CS(=O)(=O)C1=NC=C(Br)S1 | 10 | I |
| BrC1=NC(=CN=C1)C1CCOCC1 | 10 | I |
| BrC1=NC(=CN=C1)[C@H]1CCCOC1 | 10 | I |
| CSC1=CC=C(C=C1)C1CC(=O)CC(=O)C1 | 10 | I |
| CS(=O)(=O)CCI | 10 | I |
| CC1(C)CC2=CC=CC([C@H]3CC(=O)CCO3)=C2O1 | 10 | I |
| C[C@@H]1[C@@H](CCC1=O)[S@](=O)[C@@H]1CCC[C@@H](C)C1 | 10 | I |
| CC1=C(C)N=C(S1)[C@H](CBr)C#N | 10 | I |
| C[C@@H](Cl)C1=NN=C(S1)[C@@H]1CCSC1 | 10 | I |
| C[C@@H](Cl)C1=NN=C(S1)[C@@H]1CCCS1 | 10 | I |
| CSCC1=C(Br)C(C)=NN1C | 10 | I |
| FC1=C(F)C=C2C(Cl)=NN=C(Cl)C2=C1 | 10 | I |
| FC(F)(Br)C(=O)N1CCOCC1 | 10 | I |
| BrC1=CN=CN=C1Br | 10 | I |
| C[C@@H]1[C@H](C)[S@](=O)C2=C(C(C)=CC=C2C)C1=O | 10 | I |
| C[C@@H]1[C@@H](C)[S@](=O)C2=C(C(C)=CC=C2C)C1=O | 10 | I |
| O=C1CCC(=C1)[C@@H]1CCO[C@]2(CCSC2)C1 | 10 | I |
| C[C@H]1CCC(=O)[C@H](CC2=NC(C)=C(C)S2)C1 | 10 | I |
| O=C1C[C@@H]2CCC[C@H](C1)N2C[C@@H]1CCCS1 | 10 | I |
| Br[C@H]1CCN(C[C@H]2CCCO2)C1 | 10 | I |
| BrC[C@H]1C[C@]11CCS(=O)(=O)C1 | 10 | I |
| CN1C(C)=C(Br)C(C)=C(C#N)C1=O | 10 | I |
| F[C@H]1C=C(OC2CC2)C=N[C@@H]1Br | 10 | I |
| CC1=NC(Br)=C(C#N)C(C)=C1C#N | 10 | I |
| CC\N=C1\[C@@H]([O-])[C@H](S)C(C)(C)C(C)(C)C1=O | 10 | I |
| ClCC(=O)N1CCC(Br)CC1 | 10 | I |
| CN1C(=O)N(C)C2=NC(Br)=C(Br)C=C12 | 10 | I |
| CC(=O)[C@@H]1CC[C@H]2[C@@H]3[C@@H](CC(=O)[C@]12C)[C@]1(C)[C@H](CCCC1=O)CC3=O | 10 | I |
| BrC1=CC=C(C=C1)N1C(=O)C=C(N2CCCC2)C1=O | 10 | I |
| CCN1C(=O)C2(OCCCO2)C2=CC(Br)=CC=C12 | 10 | I |
| CC1=CC(=C(C)C=C1Br)S(=O)(=O)N1C=CN=C1 | 10 | I |
| BrC1=CC=C(C=C1)[C@@H]1[C@H](C#N)[C@H](C#N)N2CCCN12 | 10 | I |
| BrC1=CC=C(C=C1)[C@@H]1[C@H](C#N)[C@@H](C#N)N2CCCN12 | 10 | I |
| C[C@H]1CN(C[C@H](C)O1)C(=O)CC1=COC2=C1C=C1CCCC1=C2 | 10 | I |
| FC1=CC=C(N2C(=O)N(CC#C)C3=C(SC=C3)C2=O)C(F)=C1 | 10 | I |
| CN1CCN(CC1)S(=O)(=O)C1=CC(Br)=C(C)C=C1C | 10 | I |
| CCN1C=C(Br)C=C1C(=O)N1C[C@@H](C)O[C@H](C)C1 | 10 | I |
| CN1CCCN(CC1)S(=O)(=O)C1=CC=C(F)C=C1Br | 10 | I |
| CCN1C(=S)N(C)C(C1=O)=C1C=CC2=CC=CC=C2N1CC | 10 | I |
| C[C@@H](Cl)C1=NC2=CC(F)=CC=C2N1[C@]1(C)CCS(=O)(=O)C1 | 10 | I |
| ClC1=NC=NC2=C1C(I)=CN2[C@@H]1CCOC1 | 10 | I |
| FC1=CC=C(C=C1)[C@H]1N(C(=O)CS1(=O)=O)C1=CC=C(F)C=C1 | 10 | I |
| CN1C=CN=C(OC2=CC=C(I)C=C2)C1=O | 10 | I |
| BrC1=CC2=C(OCCCO2)C=C1C(=O)[C@@H]1CCCOC1 | 10 | I |
| CC1(C)CCCN1S(=O)(=O)C1=CC(Br)=CN=C1 | 10 | I |
| C[C@H]1CN(C[C@H](C)S1)S(=O)(=O)C1=CC(Br)=CN=C1 | 10 | I |
| FC1=CC2=C(O[C@H](C2)C(=O)N2CCC[C@@H](C2)C2=NC=CS2)C=C1 | 10 | I |
| CC1=C(CBr)N2N(C1=O)C(=O)C(C)=C2CBr | 10 | I |
| BrC1=CC=CC(=C1)C1=CC=NN1[C@@H]1CCS(=O)(=O)C1 | 10 | I |
| C[C@@]12CC=C3[C@H](CC[C@]45CC6(CC[C@]34O5)OCCO6)[C@@H]1CCC2=O | 10 | I |
| COC1=C(SC(C)=C1Br)C(=O)N1CCOC[C@@H]1C | 10 | I |
| CN(C(=O)C1=CC=C(F)C(Br)=C1)C1=CN(C)N=C1 | 10 | I |
| CN1CCO[C@H](C1)C1=NC=C(I)C(Cl)=N1 | 10 | I |
| Cl[C@H]([C@H]1CCS(=O)(=O)C1)C1=CC2=C(OCCO2)C=C1Cl | 10 | I |
| FC1=C(Br)C=CC(=C1)N1C(=O)C2=CC=NC=C2C1=O | 10 | I |
| CC1=CC=C(C)N1C1=C(SC=C1)C(=O)N1CC[S@@](=O)C(C)(C)C1 | 10 | I |
| CC1=CSC(CN2C=NC3=C(C4=C(CCCC4)S3)C2=O)=N1 | 10 | I |
| ClC1=C(C=C(C=C1)N1CCCC1=O)C(=O)N1C[C@H]2CC=CC[C@H]2C1 | 10 | I |
| FC(F)(F)C1=CC2=C(SCCN2C(=O)C2=CN=CC=N2)C=C1 | 10 | I |
| C[C@H]1SCCN([C@H]1C)C(=O)[C@@H]1CC(=O)N(C)[C@@H]1C1=CC=C(F)C=C1 | 10 | I |
| C[C@@H]1CCN([C@@H](C)C1)C(=O)[C@@H]1CC(=O)N(C)[C@@H]1C1=CC=C(F)C=C1 | 10 | I |
| FC1=CC2=C(OC[C@@H](C2)C(=O)N2CCSC3=C2C=NC=C3)C=C1 | 10 | I |
| ClC1=NC(=NC(C2CC2)=C1Br)C1=NC=CC=N1 | 10 | I |
| CN1N=C(C)C(Cl)=C1CN1C=C(Br)C=CC1=O | 10 | I |
| CCC1=C(Br)C(Cl)=NC(=N1)[C@H]1CN(C)CCO1 | 10 | I |
| BrC1=CN2C(CN3CCOC4(CCC4)C3)=CN=C2C=C1 | 10 | I |
| CC1=CC(=NC(C)=N1)N1N=CC(Br)=C1C(F)(F)F | 10 | I |
| CC1(C)C(=O)N(C2=CC=C(I)C=C2)S1(=O)=O | 10 | I |
| CC1=CN(N=C1I)[C@@H]1CCO[C@@]2(CCOC2)C1 | 10 | I |
| C[C@@H]1N(C(=O)[C@H]2CCCN2C1=O)C1=CC=C(Br)C=C1F | 10 | I |
| C[C@@H]1CN(C[C@@H](C)S1)C1=NC(Br)=CN2C=CN=C12 | 10 | I |
| ClC1=C(Br)C=C(C=C1)N1CC(=O)N2CCC[C@H]2C1=O | 10 | I |
| BrC1=CC2=C(C=C1)C(=O)N(C[C@@H]1CCCCO1)C=N2 | 10 | I |
| CC1=C(Cl)N(N=C1C(F)(F)F)[C@]1(C)CCS(=O)(=O)C1 | 10 | I |
| FC1=C(Cl)C=C2N=C(CCl)N([C@H]3CCCS(=O)(=O)C3)C2=C1 | 10 | I |
| FC1=CC=C(C=C1)[C@@H]1CCCCN(C1)C(=O)C1=COC(=O)C=C1 | 10 | I |
| FC1=CC=C2C(=O)N(C(=O)C2=C1)C1=CC=CC=C1C(F)(F)F | 10 | I |
| CN1C(=O)C2=C(SC3=C2CCCC3)N=C1SCC#C | 10 | I |
| CCN1C(=S)S\C(=C\C2=CC3=CC=CC=C3O[C@@H]2C)C1=O | 10 | I |
| BrC[C@@H]1CSC2=NN=C(N12)C1=CC=CC=C1 | 10 | I |
| CC1=CN=C(S1)N1[C@H](SCC1=O)C1=CC=C(C)C=C1 | 10 | I |
| FC1=CC(\C=C2/CS(=O)(=O)C3=CC=CC=C3C2=O)=CC=C1 | 10 | I |
| C[C@@H]1CN([C@@H](C)CO1)C(=O)C1=CC=C(Br)C=C1F | 10 | I |
| ClC1=C(Cl)C=C(CN2CCS(=O)(=O)CC2)C=C1 | 10 | I |
| BrC1=CN(CC2=CC=C3N=CC=CC3=C2)N=C1 | 10 | I |
| Br[C@H]([C@@H]1CCCO1)C1=CC2=C(OCCO2)C=C1 | 10 | I |
| FC(F)(F)C1=NN(C=C1)C1=C(Br)C=CC=N1 | 10 | I |
| Cl[C@@H]([C@@H]1CCS(=O)(=O)C1)C1=CC2=C(OCC2)C=C1 | 10 | I |
| ClCC1=NOC(=O)\C1=C/C1=CC(Br)=CC=C1 | 10 | I |
| Br[C@H]([C@H]1CCCCO1)C1=CC2=C(OCCO2)C=C1 | 10 | I |
| BrCC1=CN(N=N1)C1=CC=CC=C1Br | 10 | I |
| ClCC1=CN(N=N1)C1=CC=C(I)C=C1 | 10 | I |
| CN1C(=O)COC2=CC=C(I)C=C12 | 10 | I |
| FC1=CC(C=O)=CC(F)=C1N1C=C(Br)C=N1 | 10 | I |
| C[C@@H]1CCC2=C(C1)SC=C2C(=O)N(C)C1(CCC1)C#N | 10 | I |
| ClC1=C(C=C(Br)C=C1)N1C[C@H](CC1=O)C#N | 10 | I |
| CO[C@H]1CCC[C@@H](C1)N1C=C(I)C=N1 | 10 | I |
| CC1=CC=NC(=C1)N1C=C(I)C=N1 | 10 | I |
| CC1=NN=C(Cl)N1C1=CC=C(F)C=C1Br | 10 | I |
| BrC1=CC=C(CN2N=C3CCC[C@@H]3C2=O)C=C1 | 10 | I |
| COC1=C(Br)C=C(Br)C(=N1)C#N | 10 | I |
| C[C@@]12C[C@@H]3O[C@]33[C@H](CCC4=CC(=O)CC[C@]34C)[C@H]1CCC2=O | 10 | I |
| C[C@]12C[C@@H]3O[C@]33[C@@H](CCC4=CC(=O)C=C[C@@]34C)[C@@H]1CCC2=O | 10 | I |
| FC1=C(Br)C=C2N(N=CC2=C1)[C@@H]1CCCCO1 | 10 | I |
| ClC1=NN(C(=O)C=C1)C1=CC=C(Br)C=C1 | 10 | I |
| C[C@@H]1CCC[C@H](C1)N(C)C(=O)C1=COC(Br)=C1 | 10 | I |
| FC1=C(F)C=C(C=C1)S(=O)(=O)[C@H]1CCCC[C@@H]1C#N | 10 | I |
| ClCC1=NC2=C(N=CC(Br)=C2)N1C1CCC1 | 10 | I |
| CCN1CCN(C[C@@H]1C)C1=NC(Br)=CS1 | 10 | I |
| CC1=CN(C[C@@H]2CCOC2)N=C1I | 10 | I |
| CC1=C(Br)C(Cl)=NC(=N1)[C@@]1(C)CCCO1 | 10 | I |
| C[C@H]1CO[C@H](CBr)CN1C1=CC=C(Cl)C=N1 | 10 | I |
| C[C@H]1C[C@@H](CC(=O)N2CC[S@@](=O)C(C)(C)C2)CC(C)(C)C1 | 10 | I |
| C[C@H]1CCCN(CC2=C(Br)C(C)=NN2C)C1 | 10 | I |
| O=C1CC[C@@]2(CCOC2)CN1CC1=CC2=C(CCCC2)C=C1 | 10 | I |
| BrC1=CN(CC2=CC=NC3=CC=CC=C23)N=C1 | 10 | I |
| ClC1=CC(CN2CCC3(C2)OCCO3)=CC(Cl)=C1 | 10 | I |
| C[C@H]1C[C@H](C)[C@H](CN2C[C@@H]3CCCCN3C[C@@H]2C)C(=O)C1 | 10 | I |
| C[C@@H](Cl)C1=NN=C(O1)C12CC3C[C@](C)(C[C@](C)(C3)C1)C2 | 10 | I |
| IC1=CC=C(CN2C=CN=C2C#N)C=C1 | 10 | I |
| CN1C=C(CN2CCC3(CCC4=CC=C(C)C=C34)CC2)C=N1 | 10 | I |
| CCN1C(=O)S\C(=C/C2=CC(Br)=CS2)C1=O | 10 | I |
| BrC1=NC=NC=C1C1=CC=C2C=CC=NC2=C1 | 10 | I |
| CC(C)C1=NN=C2C=C(I)C=CN12 | 10 | I |
| COC1=CC=C2N=CC(F)=C(I)C2=N1 | 10 | I |
| COC1=CC(Br)=CC=C1S(Cl)(=O)=O | 10 | I |
| CC1=NN(C(Cl)=C1C(=O)N1CCCC1)C1=CC=C(F)C=C1 | 10 | I |
| O=C1C[C@@]2(CCC3=CC=CC=C23)C(=O)N1CN1CCSCC1 | 10 | I |
| O=C1CC2(CCCC2)C(=O)N1CN1CCC2=C(C1)C=CS2 | 10 | I |
| C[C@H]1CN(CC(=O)N2CCSC3=C2C=CC=C3)C[C@@H](C)O1 | 10 | I |
| BrC1=C(Br)C(=O)N(N=C1)C1=CC=NC=C1 | 10 | I |
| CC[C@@H]1COCCN1C(=O)C1=CC(Br)=CN1C | 10 | I |
| C[C@@H]1CCC[C@]2(C)C[C@H]3OC(=O)[C@H](CN4CCOCC4)[C@H]3C=C12 | 10 | I |
| FC1=CC=CC=C1C(=O)N1C[C@H]2C[C@@H](C1)C1=CC=CC(=O)N1C2 | 10 | I |
| CC1=CC=C(C=C1C)N1C(=O)C[S@@](=O)[C@H](C1=O)C1=CC=CC=C1 | 10 | I |
| O=S(=O)([C@H]1C[C@H]2C=C[C@@H]1CCC21OCCO1)C1=CC=CC=C1 | 10 | I |
| BrC1=CC=CC(=C1)C1=NO[C@H]2CS(=O)(=O)C[C@H]12 | 10 | I |
| BrC1=CC=CC(=C1)C1=NO[C@@H]2CS(=O)(=O)C[C@@H]12 | 10 | I |
| CN([C@@H]1CCS(=O)(=O)C1)C1=CC=C(Br)C=N1 | 10 | I |
| Br[C@@H]([C@@H]1COCCO1)C1=CC2=C(OCCCO2)C=C1 | 10 | I |
| Br[C@@H]([C@H]1COCCO1)C1=CC2=C(OCCO2)C=C1 | 10 | I |
| CCN1C=C(Br)C=C1C(=O)N1C[C@@H](C)CC1=O | 10 | I |
| COC1=CC=C2[C@@H]3CC4=C5C(O[C@H]1[C@]25CCN3C)=C(OC)C=C4 | 10 | I |
| FC1=C(F)C2=C(C=C1)N=C(CCl)N2[C@H]1CCCS(=O)(=O)C1 | 10 | I |
| FC1=C(F)C=C(C(=O)N2CCC[C@H](C2)N2C=CN=C2)C(Cl)=C1 | 10 | I |
| C[C@@H]1CSCCN1S(=O)(=O)C1=C(C)N=C(Cl)S1 | 10 | I |
| CS(=O)(=O)N1CCC[C@@H]1C1=CC=C(Br)C=N1 | 10 | I |
| CC1CCN(CC1)C(=O)CN1C=C(I)C=N1 | 10 | I |
| FC1=CC(F)=C(C(F)=C1)S(=O)(=O)N1CCC[C@@H](C1)C#N | 10 | I |
| BrC1=CN2C=CN=C2C(=N1)N1CCSCC1 | 10 | I |
| C[C@@H]1CCCN(C1)S(=O)(=O)N1CC[C@H](Br)C1 | 10 | I |
| C[C@H](N1CC[S@@](=O)C(C)(C)C1)C(=O)N1[C@H](C)CC2=CC=CC=C12 | 10 | I |
| CC1(C)CN(CC2=CC(Cl)=CC3=C2OCOC3)CC[S@]1=O | 10 | I |
| CN1N=C(C)C(Br)=C1CC1(CCOCC1)C#N | 10 | I |
| O=C(N1CCS[C@@H]2COCC[C@H]12)C1=C(N=CS1)C1CC1 | 10 | I |
| O=C1CCCC[C@@H]1[C@H]1CCCCN1[C@@H]1CCS(=O)(=O)C1 | 10 | I |
| C[C@@H]1[C@@H](C)S(=O)(=O)CCN1CC1=CC2=C(C=CS2)N=C1 | 10 | I |
| CN1C=CN(CC2=CC(F)=CC(Br)=C2)C(=O)C1=O | 10 | I |
| CS(=O)(=O)[C@H]1CSCCN1C1=C(F)C=C(Cl)C=N1 | 10 | I |
| CN1C=C(CN2N=C(C)C(I)=C2C)C=N1 | 10 | I |
| CCN1C(=O)C2=CC(I)=CC=C2S1(=O)=O | 10 | I |
| CC1(C)C(=O)N(CC2=CC=C(Br)C=C2)S1(=O)=O | 10 | I |
| CC1(C)C(=O)N(C[C@@H]2CSC3=C2C=CC=C3)S1(=O)=O | 10 | I |
| C[C@@H](C#N)N1CCN(CC1)[C@@H]1CCOC2(CCSCC2)C1 | 10 | I |
| CC1=NN([C@@H]2CCO[C@@]3(CCOC3)C2)C(C)=C1Br | 10 | I |
| BrC1=CC=C(CN2CCC(=O)N3CCC[C@H]3C2=O)C=C1 | 10 | I |
| C[C@@H](N1[C@H](C)C(=O)N2CCC[C@@H]2C1=O)C1=C(C)SC(C)=C1 | 10 | I |
| O=C1CCN(C2=CC3=C(SC=C3)C=C2)C(=O)[C@H]2CCCN12 | 10 | I |
| O=C1CCCCC[C@H]1C1=NC(=NO1)[C@H]1CSCCS1 | 10 | I |
| CC1=C(Br)SC(=C1)S(=O)(=O)N1CCCC1=O | 10 | I |
| CS(=O)(=O)[C@@H]1CCC[C@@H](C1)N1C=C(Br)C=N1 | 10 | I |
| CC(C)[C@@H](Br)C(=O)N1CCN(C)C(=O)C1(C)C | 10 | I |
| COC(=O)C1=C[C@@H]2N=NC(I)=C2C=C1 | 10 | I |
| COC(=O)C1=C2C=NN=C2C=C(I)C1 | 10 | I |
| CC1=CSC(=O)N1CC(=O)N1CCSC2=C1C=CC=C2 | 10 | I |
| CN([C@H]1CCS(=O)(=O)C1)C1=C(Br)C=C(C)C=N1 | 10 | I |
| CN1C(=S)S\C(=C\C2=CC=C(O2)C2=CC=C(F)C=C2)C1=O | 10 | I |
| FC1=C(F)C(F)=C(N2C(=O)C(Cl)=C(Cl)C2=O)C(F)=C1F | 10 | I |
| C[C@@]12CC=CC[C@@H]1C(=O)N(C2=O)C1=CC=C(Br)C=C1 | 10 | I |
| C[C@]12CC=CC[C@H]1C(=O)N(C2=O)C1=CC=C(Br)C=C1 | 10 | I |
| FC1=C(F)C=C2N3C(SC4=C(F)C(F)=C(F)C=C4C3=O)=NC2=C1 | 10 | I |
| ClN(Cl)S(=O)(=O)C1=CC=C(Br)C=C1 | 10 | I |
| CN(C)C(=O)CC1=C(Br)OC2=C1C=C(Cl)C(C)=C2 | 10 | I |
| C[C@H]1C[C@@H]2[C@@H]3CC[C@H](C(C)=O)[C@@]3(C)CC(=O)[C@H]2[C@@]2(C)CCC(=O)C=C12 | 10 | I |
| COC1=C(OC)C=C2C(S[C@@H](C)C3=C2CC[C@@]2(C)C(=O)CC=C32)=C1 | 10 | I |
| C[C@@H](Cl)C1=NN=C(O1)C1=CC=C(Br)C=C1F | 10 | I |
| C[C@@H](Cl)C(=O)C1=CC2=C(CCCN2C(C)=O)C=C1Br | 10 | I |
| CC1=C(C=CO1)[C@H](Br)C1=CC=C2OCCCOC2=C1 | 10 | I |
| CC1=C(C=CO1)[C@@H](Br)C1=CC=C2OCCCOC2=C1 | 10 | I |
| CC1=CC([C@H](Br)C2=CC=C3OCCOC3=C2)=C(C)O1 | 10 | I |
| ClC1=CC2=C(OCCO2)C=C1[C@@H](Br)[C@H]1CCCO1 | 10 | I |
| C[C@H]1CCN(C1)S(=O)(=O)C1=C(F)C=C(F)C=C1Br | 10 | I |
| BrC1=C(CN2C(=O)COC3=CC=CC=C23)SC=C1 | 10 | I |
| FC1=CC2=C(C=C1)C(=O)C(=O)N2CC1=CC(Br)=CS1 | 10 | I |
| C[C@@]12CCCC(=C)[C@H]1C[C@H]1[C@@H](C2)OC(=O)[C@H]1CN1CCCCC1 | 10 | I |
| Br[C@@H]([C@@H]1CCCCO1)C1=CC2=C(OCCCO2)C=C1 | 10 | I |
| COC1=CC=C(C=C(C#N)C#N)C=C1I | 10 | I |
| ClS(=O)(=O)C1=CC2=C(OC=C2)C(I)=C1 | 10 | I |
| C[C@H]1CN(CC2=NC3=C(C=CS3)C(Cl)=N2)C[C@@H](C)S1 | 10 | I |
| BrC1=CC(N2C[C@@H](CC2=O)C#N)=C(Br)C=C1 | 10 | I |
| CC(=O)C1=CC=C(C=C1)N1C=C(I)C=N1 | 10 | I |
| CC1(C)CC2=CC=CC([C@H](Cl)[C@H]3CCS(=O)(=O)C3)=C2O1 | 10 | I |
| ClC1=NC(=NC(Cl)=C1Br)[C@@H]1C[C@H]2CC[C@@H]1O2 | 10 | I |
| ClC1=NC(C[C@@H]2CCCO2)=NC(Cl)=C1Br | 10 | I |
| ClC1=CN(C[C@@H]2CCOC2)N=C1I | 10 | I |
| FC1=C(N=CC(Cl)=C1)N1CCO[C@H](CBr)C1 | 10 | I |
| CCN1C=C(Br)C=C1C(=O)N1C[C@H](C)CC[C@H]1C | 10 | I |
| C[C@@H]1CC[C@@H](C)N(C1)C(=O)C1=CC(Br)=CN1C1CC1 | 10 | I |
| CCC1=C(Br)C(Cl)=NC(=N1)[C@@H]1C[C@H]2CC[C@@H]1O2 | 10 | I |
| CCC1CCN(CC1)C1=C(I)C=NC=N1 | 10 | I |
| IC1=CN=CN=C1N1CCC[C@@H]2CCC[C@H]12 | 10 | I |
| FC(F)(F)C1=C(C=CC(Br)=C1)N1CCO[C@@H](C1)C#N | 10 | I |
| FC1=CC2=C(O[C@@H](CN3C=C(Br)C=CC3=O)C2)C=C1 | 10 | I |
| BrC1=C2C(=O)C(=O)N(C[C@@H]3CC4=CC=CC=C34)C2=CC=C1 | 10 | I |
| CC1=NC2=C(S1)C=C(O[C@@H]1CCCC(=O)C1)C1=C2SC=C1 | 10 | I |
| CC1=NC2=C(S1)C=C(O[C@H]1CCCC(=O)C1)C1=C2SC=C1 | 10 | I |
| IC1=CC2=C(C=NN2C[C@H]2CCCO2)C=C1 | 10 | I |
| FC(F)CN1C(=O)C2=C(C=C(I)C=C2)C1=O | 10 | I |
| IC1=CC2=C(OCC(=O)N2CC2CCC2)C=C1 | 10 | I |
| CCCN1C(=O)COC2=CC=C(I)C=C12 | 10 | I |
| BrC1=CN=CC(O[C@H]2CCOC3(CCSCC3)C2)=C1 | 10 | I |
| C[C@@H]1CN2CCC[C@@H]2CN1C1=C(CCl)C=C(Br)C=N1 | 10 | I |
| CC1=C(C=C(Br)S1)S(=O)(=O)N1CCC(C)(C)C1 | 10 | I |
| C[C@H]1C[C@@]11[C@H](C)C2=C1C1=C(C[C@@H](C)OS1(=O)=O)C1=C2C[C@H]1C | 10 | I |
| C[C@@H]1[C@@H](C)C(=O)N(CC2=CC=C(F)C(Br)=C2)C1=O | 10 | I |
| CN(C(=O)C1=CC(Br)=CN1C)C1=CC=C(F)C(F)=C1 | 10 | I |
| CCN1C(SC2=CC=CC=C12)=C1N(C)C(=S)N(C)C1=O | 10 | I |
| CC1=CC(C=C2C(=O)C3=CC=CC=C3C2=O)=C(C)N1C1=CC=NC=C1 | 10 | I |
| C[C@@]12O[C@@H](C=C1)[C@H]1[C@@H]2C(=O)N(C1=O)C1=CC=C(F)C(Cl)=C1 | 10 | I |
| BrC1=CC=CC=C1\C=C1\SC2=NC=NN2C1=O | 10 | I |
| FC1=CC=C(C=C1)C(=O)CN1N=CC(Cl)=C(Cl)C1=O | 10 | I |
| CC1=NN(C(C)=C1Cl)S(=O)(=O)C1=CC=C(F)C(F)=C1 | 10 | I |
| CC1=NC=CN1S(=O)(=O)C1=CC(Cl)=CC=C1Br | 10 | I |
| C[C@@H]1CN(C[C@@H](C)O1)C(=O)C1=CC2=C(C=C1)N(C)C(C)=C2C | 10 | I |
| CC1=CC=C(C=C1Br)N1C(=O)[C@H]2[C@H]3O[C@H](C=C3)[C@@H]2C1=O | 10 | I |
| CN1CCN(CC1)C1=NC=NC2=CC=C(Br)C=C12 | 10 | I |
| CN1C[C@H]2SC3=NC(=S)N(CC=C)C(=O)C3=C2CC1(C)C | 10 | I |
| BrCC1(OCCO1)C1=CC2=C(OCCO2)C=C1 | 10 | I |
| C[C@H]1CCC2=C3C(S[C@@H]2C1)=NC(CN1CCCC1)=NC3=O | 10 | I |
| FC1=C(F)C2=C(C=C1)N=C(CCl)N2[C@H]1CCS(=O)(=O)C1 | 10 | I |
| CC(C)=CC(=O)N1CCN(CC2=CC=C3OCCC3=C2)CC1 | 10 | I |
| CN(CC1=CC=C(Br)O1)C(=O)C1=CSC=N1 | 10 | I |
| CN(CC1=CC=C(F)C=C1)C(=O)C1=CC=C2OCCOC2=C1 | 10 | I |
| FC1=CC=C(C=C1)N1CCN(CC1)C(=O)[C@@H]1C[C@H]2CC[C@@H]1O2 | 10 | I |
| C[C@@H]1C[C@H](CN1C(=O)C1=CC(=O)N(C)C=C1)C1=CC=C(F)C=C1 | 10 | I |
| C[C@H](C(=O)N1CCCS1(=O)=O)C1=CC=C(Br)S1 | 10 | I |
| CC(C)N1C=C(Br)C=C1C(=O)N1[C@@H](C)COC[C@@H]1C | 10 | I |
| CN1C=C(C=CC1=O)C(=O)N1CCCC2=C1C(F)=CC(F)=C2 | 10 | I |
| C[C@@H]1CN(C(=O)CN1C(=O)C1=CSC=C1)C1=CC=C(C)C=C1 | 10 | I |
| FC1=CC2=C(OC=C(\C=C3\COC4=CC=C(F)C=C4C3=O)C2=O)C=C1 | 10 | I |
| CCN1C=NC=C1C1=NC(C)=C(Br)C(Cl)=N1 | 10 | I |
| CC(C)N1C=NC=C1C1=NC=C(Br)C(Cl)=N1 | 10 | I |
| ClCC1=NC(=CN=C1)N1C=C(I)C=N1 | 10 | I |
| ClC1=NC(SC2=NC=NC=C2)=C(Br)C=N1 | 10 | I |
| C[C@H]1CN(CCN1C)C1=C(Br)C=NC(Cl)=N1 | 10 | I |
| CC[C@@H]1CN(CCN1C(C)C)C(=O)C1=CC=C2OC=CC2=C1 | 10 | I |
| C[C@H](Cl)C1=NC2=CN=CC=C2N1[C@@H]1CCN2CCCC[C@H]12 | 10 | I |
| C[C@@H](Cl)C1=NC2=C(N=CC=C2C)N1CC1=CC=C(C)N=C1 | 10 | I |
| C[C@H](Cl)C1=NC2=CC(C)=CN=C2N1CC1=CC=C(C)N=C1 | 10 | I |
| CN1[C@H]2CC[C@@H]1CN(CC1=C(Cl)N=C3SC=CN13)CC2 | 10 | I |
| CN1CCO[C@@H]2CN(CC3=C4OC(C)(C)CC4=CC=C3)C[C@H]12 | 10 | I |
| ClC1=NC=C(CN2CCN(CC2)C2=NC=CS2)S1 | 10 | I |
| O=C1COC2=C1C=CC(O[C@H]1CCO[C@]3(CCSC3)C1)=C2 | 10 | I |
| O=C1COC2=C1C=CC(O[C@H]1CCO[C@@]3(CCSC3)C1)=C2 | 10 | I |
| CSC1=CC=C(C=C1)N1[C@H](C)C(=O)N2CCCC[C@@H]2C1=O | 10 | I |
| C[C@@H]1N(C(=O)[C@@H]2CCCCN2C1=O)C1=CC=C(Cl)C(F)=C1 | 10 | I |
| C[C@@H]1N(CC2=CC(C)=C(C)S2)C(=O)[C@@H]2CCCCN2C1=O | 10 | I |
| CC[C@H]1N(C(=O)[C@H]2CCCN2C1=O)C1=CC=C(F)C(Cl)=C1 | 10 | I |
| CC[C@H]1N(C(=O)[C@@H]2CCCN2C1=O)C1=CC(Cl)=CC=C1F | 10 | I |
| BrC1=CC2=C(C=C1)C=NN(C1=CC=NC=C1)C2=O | 10 | I |
| CN1C=CC(=CC1=O)C(=O)N1CCCC2=C1C(F)=CC=C2F | 10 | I |
| CN(C)S(=O)(=O)C1=CC(Br)=CC2=C1OCC2 | 10 | I |
| C[C@@]1(CCS(=O)(=O)C1)N1N=C(C=C1Cl)C(F)(F)F | 10 | I |
| CC1=CC=C(O1)C1=CC=C(S1)C(=O)N1CC[S@](=O)C(C)(C)C1 | 10 | I |
| CN(C(=O)[C@@H]1CN(C)C(=O)C1)C1=CC=C(Br)C=C1C | 10 | I |
| C[C@H]1CN(C)CCN1C(=O)\C=C/C1=CC=C(Br)O1 | 10 | I |
| O=C1OCC[C@H]1SC1=CC2=C(OCCO2)C=C1 | 10 | I |
| CC1=CC(=CC=C1F)C1=C[N+]([O-])=C2CCCCC2=[N+]1[O-] | 10 | I |
| CC1=CC=C(CN2C(=O)CSCC2=O)C=C1 | 10 | I |
| CC(=O)S[C@@H]1CS(=O)(=O)C[C@H]1Br | 10 | I |
| CC1(C)[C@@H]([C@H](Cl)S1(=O)=O)N1CCOCC1 | 10 | I |
| O=S1(=O)C[C@@]2(CCC[C@H]12)N1CCOCC1 | 10 | I |
| CN1CCS\C1=C/C=C1\OC(=S)N(C)C1=O | 10 | I |
| O=C(CN1CCSCC1)N1CCOCC1 | 10 | I |
| C[C@H]1CN(CCC1=O)[C@]1(C)CCS(=O)(=O)C1 | 10 | I |
| CN1CCCN(CCC1)S(=O)(=O)CCl | 10 | I |
| CN(C)C1=CC=C(C=C1)C1=[N+]([O-])C(C)(C)C(C)=N1 | 10 | I |
| CN1CCN(C)CCN(C)CCN(C)CC1 | 10 | I |
| C[C@H]1CC(=O)CCN1C[C@@H]1CN(C)CCN1C | 10 | I |
| FC1=C(F)C=C(C=C1)C(=O)N1CCCS1(=O)=O | 10 | I |
| CN1C=C(CN2CCC[C@H](Br)C2=O)C=N1 | 10 | I |
| CC1(C)OCC2(CN(C2)C(=O)CCl)CO1 | 10 | I |
| C[C@H]1CN(CCN1C)C(=O)CBr | 10 | I |
| C[C@]1(CCS(=O)(=O)C1)N1CC[C@H](Br)C1=O | 10 | I |
| FC1=CC=C(C=C1)[S@@](=O)[C@H]1CCS(=O)(=O)C1 | 10 | I |
| C[C@@H]1[C@H](CCC1=O)[S@@](=O)C1CCOCC1 | 10 | I |
| CS(=O)(=O)[C@@H]1CCC[C@]11CC(=O)CCO1 | 10 | I |
| CSC1(CCN(CC1)S(C)(=O)=O)C#N | 10 | I |
| BrC1=CC=C(CN2CC(=O)CC2=O)C=C1 | 10 | I |
| CN1N=CC([C@H](Cl)[C@@H]2CCS(=O)(=O)C2)=C1C | 10 | I |
| CC[C@H](Br)C(=O)N1CCO[C@H](C1)C#N | 10 | I |
| CC1=NN(CC2=CN=C(Cl)S2)C(C)=N1 | 10 | I |
| CC1=C(C)N=C(C[C@]2(CCCC2=O)C#N)S1 | 10 | I |
| O=C([C@@H]1CCS(=O)(=O)C1)[C@@H]1CCCCS1 | 10 | I |
| C[C@@H]1OCC[C@@H]1C(=O)N1CCO[C@H](CCl)C1 | 10 | I |
| CS(=O)(=O)N1CCO[C@@H](CBr)C1 | 10 | I |
| C[C@@H]1CO[C@H](CBr)CN1S(C)(=O)=O | 10 | I |
| C[C@H]1CO[C@@H](CBr)CN1S(C)(=O)=O | 10 | I |
| C[C@@H]1C[C@H]1C(=O)N1CC[S@@](=O)C(C)(C)C1 | 10 | I |
| CC1(C)CN(C[C@@H]2CCCCO2)CC[S@@]1=O | 10 | I |
| C[C@@H]1CN(C)CCN1CC1=NC=C(Cl)N1C | 10 | I |
| CN1N=C(C)C(CN2[C@H]3CC[C@@H]2CC(=O)C3)=C1C | 10 | I |
| C[C@H]1[C@H](C)C(=O)CCN1C1CCS(=O)(=O)CC1 | 10 | I |
| C[C@@H]1[C@H](C)C(=O)CCN1C[C@@H]1CN(C)CCN1C | 10 | I |
| CC1(C)OC(C)(C)C(=O)[C@H]1CN1CCOCC1 | 10 | I |
| CC(C)[C@H]1N(CC#C)C(=O)[C@H]2CCCN2C1=O | 10 | I |
| CC(C)[C@@H]1N(CC#C)C(=O)[C@H]2CCCN2C1=O | 10 | I |
| C([C@@H]1CN2CCC[C@@H]2CO1)N1CCN2CCC[C@H]2C1 | 10 | I |
| CC1=NN(C(C)=N1)C1=C(F)C(F)=NC(F)=C1F | 10 | I |
| C[C@H]1COCC[C@H]1S(=O)(=O)N1CCCC1 | 10 | I |
| BrC1=NO[C@@H]2CS(=O)(=O)C[C@H]12 | 10 | I |
| CN1N=NC(=N1)[C@@H](Br)CBr | 10 | I |
| COC1=C[C@H]2N=C(N=C2C=C1)C1=CC=C(F)C=C1 | 10 | I |
| BrC1=NC(=CN=C1)N1CCCCC1=O | 10 | I |
| CN1C=CC(=O)N(CC2=CC(F)=CC(F)=C2)C1=O | 10 | I |
| O=C1C=CO[C@@H]2CO[C@H](O[C@@H]12)C1=CC=CC=C1 | 10 | I |
| ClC1(Cl)[C@@H]2O[C@](C=C2)([C@@H]2CO2)C(Cl)(Cl)C1=O | 10 | I |
| FC1=CC=CC=C1C(=O)N1C=CC2=CC=CC=C2[C@H]1C#N | 10 | I |
| BrC1=CC=C(CN2C(=O)CSC2=O)C=C1 | 10 | I |
| CN1C(=S)S[C@H](\[N+]([O-])=C/C2=CC=CC=C2F)C1(C)C | 10 | I |
| BrCCC(=O)N1C=CC2=CC=CC=C2[C@H]1C#N | 10 | I |
| CN(C1=CC=CC=C1)C1=C(C)N(C)C(=S)N(C)C1=S | 10 | I |
| CC1=CC=C(C=C1)S(=O)(=O)C(C#N)=C1SCCS1 | 10 | I |
| FC1=CC=CC=C1N1C(=O)[C@H]2[C@H]([C@@H]3C=C[C@H]2[C@H]2C[C@@H]32)C1=O | 10 | I |
| BrC1=CC=C(\C=N\[C@@H]2CS(=O)(=O)C=C2)C=C1 | 10 | I |
| BrC1=CC=CC(\C=N\[C@@H]2CCS(=O)(=O)C2)=C1 | 10 | I |
| CC1(C)CC2(CCS1)N1CC3(C)CN2CC(C)(C1)C3=O | 10 | I |
| CC(=O)C1=C(C)N(N=C1)C1=CC=C(Br)C=C1 | 10 | I |
| CC(C)[C@@H](Br)C(=O)N1C[C@H](C)OC[C@@H]1C | 10 | I |
| CCN1N=CC(Br)=C(Br)C1=O | 10 | I |
| BrC1=CC=CC(CN2CCCS2(=O)=O)=C1 | 10 | I |
| CC1=NN(CC2=CC=C(F)C(Br)=C2)C(C)=N1 | 10 | I |
| BrC1=C(CN2C(=O)CCCC2=O)SC=C1 | 10 | I |
| BrC1=CSC(CN2CCCS2(=O)=O)=C1 | 10 | I |
| C[C@H]1CCC2=C(C(=O)CC2)[C@@]1(C)[C@H]1C[C@@](C)(C=C)C(=O)O1 | 10 | I |
| C[C@H](C#N)N(C)C(=O)C1=CC(F)=CC(Br)=C1 | 10 | I |
| FC1=CC(=CC(F)=C1F)[C@H](Cl)C(=O)N1CCOCC1 | 10 | I |
| BrC[C@H](C#N)[C@H]1CS(=O)(=O)C2=CC=CC=C12 | 10 | I |
| BrC1=CC=C(CN2C[C@H](CC2=O)C#N)S1 | 10 | I |
| BrC1=CSC(CN2C[C@H](CC2=O)C#N)=C1 | 10 | I |
| CC1=CC(=O)[C@@H](O1)C(=O)C1=CC=C(Br)C=C1 | 10 | I |
| C[C@H]1N(CC2=NC=C(Cl)N2C)CCC2=C1C=CS2 | 10 | I |
| FC1=CC(=C(Cl)C=C1)S(=O)(=O)N1CCSC1 | 10 | I |
| CS(=O)(=O)C1=CC=C2C(Br)=CC=NC2=C1 | 10 | I |
| CC1(C)CC(=O)N(C1=O)C1=CC=C(Br)C=C1 | 10 | I |
| Br[C@H]1CCN(C1=O)C1=NC(=CS1)C1CC1 | 10 | I |
| ICC1=NN=C(O1)C1=CC=CS1 | 10 | I |
| C[C@@H]1CC(=O)N(CC2=CC=C(Br)S2)C1=O | 10 | I |
| CC1=CC(Br)=C(S1)C(=O)[C@@H]1COCCO1 | 10 | I |
| CC1=CC(Br)=C(S1)C(=O)[C@H]1COCCO1 | 10 | I |
| ClC1=NC(C[C@H]2CCCO2)=NC=C1Br | 10 | I |
| Cl[C@@H]1CCCN(C1)C1=NC=NC=C1Br | 10 | I |
| C[C@@H]1[C@H](C)S(=O)(=O)C2=C(C=CC=C2Br)C1=O | 10 | I |
| FC(F)(F)CN1C=NC(I)=N1 | 10 | I |
| C[C@H]1CC[C@H](O1)C(=O)N1CCC[C@H](CBr)C1 | 10 | I |
| C[C@@H]1CCC[C@@H](CC(=O)N2CC[S@@](=O)C(C)(C)C2)C1 | 10 | I |
| CN1N=C(C)C(Br)=C1CN1CCCSCC1 | 10 | I |
| C[C@H]1[C@@H](C)S(=O)(=O)CCN1CC1=CC(F)=C(F)C=C1F | 10 | I |
| CN1C=CC(CN2CCO[C@]3(CCC4=C3C=CC=C4)C2)=C1 | 10 | I |
| CSC1(CCN(CC1)[C@@H]1CCOC2(CCC2)C1)C#N | 10 | I |
| O=C[C@@H]1CCCCN1[C@@H]1CCOC2(CCSCC2)C1 | 10 | I |
| C[C@@H]1C[C@H](C)[C@H](CN2CCN3CCCC[C@@H]3C2)C(=O)C1 | 10 | I |
| CC1(C)CCC(=O)[C@H](CN2CCN3CCCC[C@@H]3C2)C1 | 10 | I |
| O=C([C@H]1CSCCS1)C1=CC2=C(OCCO2)C=C1 | 10 | I |
| CN1C(=O)CCC2=CC(=CC=C12)[C@H](Cl)[C@]1(C)CCCO1 | 10 | I |
| FC(F)(F)C1(OCCO1)C1=NC=C(Br)C=C1 | 10 | I |
| BrC1=CC=C(C=C1)C(=O)\C=C\C1=CC=CO1 | 10 | I |
| CCC1=CC(Br)=CC=C1N1C=CC(C=O)=C1 | 10 | I |
| CC(=O)[C@@H]1CC[C@@H]2[C@H]3CCC4=CC(=O)CC[C@H]4[C@@H]3CC[C@]12C | 10 | I |
| CN1C=CC=C1\C=C(/C#N)C1=CC=C(Br)C=C1 | 10 | I |
| O=S1(=O)CC[C@H](SC2=CC=CC=C2)C2=C1SC=C2 | 10 | I |
| CN1C(C)=CC(\C=C(\C#N)C2=CC=C(Br)C=C2)=C1C | 10 | I |
| FC1=CC=CC=C1N1N=CC(Br)=C1C(F)(F)F | 10 | I |
| CSC1=N\C(=C\C2=CC=C(Br)C=C2)C(=O)S1 | 10 | I |
| FC1=CC=C(C=C1)[C@@H]1SCC(=O)N1C1=CC=CC=C1F | 10 | I |
| FC1=CC=C(C=C1)[C@H](Cl)C1=CC=C2OCCOC2=C1 | 10 | I |
| CC1=CC([C@@H](Cl)C2=CC=C3OCCOC3=C2)=C(C)S1 | 10 | I |
| Cl[C@@H](C1=CSC=C1)C1=CC2=C(OCCO2)C=C1Cl | 10 | I |
| C[C@H]1CC2=CC(=CC=C2O1)[C@H](Cl)C1=C(C)OC(C)=C1 | 10 | I |
| Br[C@H](C1=CC=CO1)C1=CC=C2OCCC2=C1 | 10 | I |
| FC(F)(F)C1=CC=CC=C1C1=CN=C(Br)C=N1 | 10 | I |
| COC(=O)C1=C(C)C(Br)=C(Br)S1 | 10 | I |
| FC1=CN=CC(=C1)C(=O)C1=CC=C(Br)C=C1 | 10 | I |
| CC1=NN(CC2=CC=CC(Br)=C2)C(C)=C1Cl | 10 | I |
| CC1(C)CC2=CC(=CC=C2O1)[C@H](Br)[C@H]1CCCO1 | 10 | I |
| CC1(C)CC2=CC(=CC=C2O1)[C@@H](Br)[C@H]1CCCO1 | 10 | I |
| CC1=CC(C)=C(C)C([C@@H](Br)[C@@H]2COCCO2)=C1C | 10 | I |
| CC(C)(C)[C@H](Br)C1=CC=C2OCCOC2=C1 | 10 | I |
| ClC1=NC=CC(CN2CCC3(CCCCC3)CC2)=C1 | 10 | I |
| C[C@H]1CC2=CC(=CC=C2O1)[C@@H](Br)C1CCOCC1 | 10 | I |
| COC1=C(OC)C=C2[C@@H](Br)C[C@H](C)CCC2=C1 | 10 | I |
| C[C@@H]1CC2=CC(=CC=C2O1)[C@@H](Br)[C@@H]1CCCOC1 | 10 | I |
| FC1=CC=C([C@H](Cl)[C@@H]2COCCO2)C2=CC=CC=C12 | 10 | I |
| C[C@H]1CN(C[C@@H](C)S1)C1=CC(Br)=CC=C1C=O | 10 | I |
| CC1=C(Cl)C=NN1CC1=CC=C(Br)S1 | 10 | I |
| Br[C@@H](CC1=NN=CC2=CC=CC=C12)C1CC1 | 10 | I |
| CC1=CC=C(S1)C1=NN=C(I)S1 | 10 | I |
| BrC1=C(Br)N=C(S1)C1=CC=CO1 | 10 | I |
| FC(F)(F)C1=CC(Cl)=C(C=C1)S(Cl)(=O)=O | 10 | I |
| CC1=CC=C([C@H](Cl)[C@@H]2COCCO2)C2=C1C=CC=C2 | 10 | I |
| ClCC1=NC2=C(C=CC(Br)=C2)N1C1CCC1 | 10 | I |
| CC(C)[C@H](Cl)C1=CC2=C(OCCO2)C=C1Br | 10 | I |
| CC1=C(CBr)C=NN1C1=CC=C(C)C=C1C | 10 | I |
| CCC1=CC=C(S1)[C@@H](Cl)[C@H]1CCS(=O)(=O)C1 | 10 | I |
| CC1=CC=C(O1)C(=O)C1=C(C)SC(Br)=C1 | 10 | I |
| FC1=CC=C([C@@H](Cl)[C@H]2CCS(=O)(=O)C2)C2=CC=CC=C12 | 10 | I |
| CC1=CC([C@@H](Cl)[C@@H]2CCS(=O)(=O)C2)=C(C)C=C1Cl | 10 | I |
| ClC1=CC(\C=C\CBr)=CC2=C1OCCO2 | 10 | I |
| ClC1=C(O[C@H]2CCCC(=O)C2)C=C(Br)C=C1 | 10 | I |
| C[C@@H]1CC[C@@H](C[C@@H]1C)N1C=CC(I)=N1 | 10 | I |
| BrC1=CSC(CC2=NC3=CC=CC=C3S2)=N1 | 10 | I |
| CC1(C)CCC[C@@H](CN2CCC3(CCCC3)CC2)C1=O | 10 | I |
| ClC1=NC(=NC(=C1)C1=CC=CC=C1)[C@H]1CSCCS1 | 10 | I |
| C[C@@H]1[C@@H](Cl)CC[C@H]1[C@@H]1CCC[C@@H](C1)S(C)(=O)=O | 10 | I |
| CC(=O)OC1=CC(C)=C(I)C(C)=C1 | 10 | I |
| COC1=CC=C(OC)C(I)=C1Cl | 10 | I |
| C[NH+](C)C=C1CCCC2=C1OC(=CC2=S)C1=CC=CC=C1 | 10 | I |
| CC1CCC(CC1)N1CC[NH+](C[C@@H]2C[C@H]3C[C@@H]2C=C3)CC1 | 10 | I |
| CC1=CC=C(OCC2=CC=C[NH+]=C2)C(Br)=C1 | 10 | I |
| N#C[C@H]1CC[C@@H](C[C@H]1[NH+]1CCSCC1)C1=CC=CC=C1 | 10 | I |
| IC1=CC=CC=C1OCC1=CC=C[NH+]=C1 | 10 | I |
| CN1C=C[NH+]=C1SCC1=C(Br)C=CS1 | 10 | I |
| CC[C@@H]1CO[C@@H](C)C[NH+]1CC1=C(Br)C=CS1 | 10 | I |
| C[C@@H]1C[NH+](CC2=CC=CC=C2Br)CC(C)(C)O1 | 10 | I |
| C[C@H]1C[NH+](CC2=CC=CC=C2Br)CC(C)(C)O1 | 10 | I |
| C[C@H]1C[NH+](CC2=CC=C(Br)C=C2)CC(C)(C)O1 | 10 | I |
| CC[C@H]1COCC[NH+]1CC1=CC=C(Br)C=C1 | 10 | I |
| CC1=CC(C)=C(C=C1C)C(=O)C[NH+]1CCCSCC1 | 10 | I |
| C[C@H](Cl)C1=[NH+]C2=CC=C(F)C(F)=C2N1[C@@H]1CC1(C)C | 10 | I |
| FC1=CC=CC=C1\C=C1\CCC2=C3C=CC=CC3=[NH+][C@@H]2C1=O | 10 | I |
| CCC1(C)CCN(CC1)C1=[NH+]C[C@H](CBr)S1 | 10 | I |
| C[C@H]1CCC[C@H](C1)N(C)C1=[NH+]C[C@H](CBr)S1 | 10 | I |
| CC1=C[NH+]=CC=C1OC1=CC=C(F)C=C1Br | 10 | I |
| C[C@@H]1COC[C@@H](C)[NH+]1CC1=CC(Br)=CC=C1F | 10 | I |
| FC(F)(F)[C@@H](Br)CN1C=[NH+]C2=CC=CC=C12 | 10 | I |
| C[C@H]1C[NH+](CC2(CBr)CCCCC2)CCO1 | 10 | I |
| CCC1=[NH+]N(C)C(C[C@H]2CC[C@H](Br)[C@@H]2C)=C1 | 10 | I |
| C[C@H](N1C=[NH+]C=C1CCl)C1=CC=C(Br)C=C1 | 10 | I |
| Cl[C@@H](CC1=CC=CC=[NH+]1)C1=CC=C(Br)O1 | 10 | I |
| C[C@H]([C@H](Cl)C1=CC=C(Br)O1)C1=CC=CC=[NH+]1 | 10 | I |
| C[C@H]([C@@H](Cl)C1=CC=C(Br)O1)C1=CC=CC=[NH+]1 | 10 | I |
| C[C@@H]([C@@H](Br)[C@]1(C)CCCO1)C1=CC=[NH+]C=C1 | 10 | I |
| CN(CCBr)C1=CC=[NH+]C2=C1C=CC(Cl)=C2 | 10 | I |
| ClC1=C(C=C[NH+]=C1)N1CCC[C@@H](CCBr)C1 | 10 | I |
| CC1=C(C)[NH+]=C(S1)N1CCCC[C@H]1CBr | 10 | I |
| CC1=C(C)[NH+]=C(S1)N1CCC[C@@H](CCBr)C1 | 10 | I |
| C[C@H]1CN(CC2=CC=C(F)C(Br)=C2)C[C@@H]1[NH+](C)C | 10 | I |
| BrC1=CC=C(C[NH+]2CCOC[C@@H]2C2CC2)C=C1 | 10 | I |
| BrCC1(C[NH+]2C[C@@H]3CC[C@H](C2)O3)CCCCCC1 | 10 | I |
| C[C@@H](C(=O)C1=CC(Br)=CC=C1F)C1=CC=[NH+]C=C1 | 10 | I |
| Br[C@H](CC1=[NH+]C2=CC=CC=C2C=C1)C1=CC=CO1 | 10 | I |
| C[C@H]([C@@H](Br)[C@H]1[C@@H](C)O[C@H](C)[C@@H]1C)C1=CC=CC=[NH+]1 | 10 | I |
| C[C@@H]1O[C@H](C)[C@H]([C@@H](Br)CC2=CC=C(C)C=[NH+]2)[C@@H]1C | 10 | I |
| C[C@@H]1C[NH+](CC2=CC=C(F)C(=C2)C(F)(F)F)[C@H](C)CO1 | 10 | I |
| C[C@H]1C[NH+](CC2=CC(=CC(F)=C2)C(F)(F)F)[C@H](C)CO1 | 10 | I |
| C[C@@H]1C[C@@H]2CCCC[C@@H]2[NH+]1CC1=CC=C(C=C1)[S@](C)=O | 10 | I |
| Cl\C=C(\Cl)C[NH+]1CCCC[C@@H]1[C@@H]1CCCCC1=O | 10 | I |
| BrC1=CC=C(CC(=O)[C@H]2CCC3=C2[NH+]=CC=C3)C=C1 | 10 | I |
| CN1C(C[C@@H]2CC[C@@H](Br)C2)=[NH+]C2=CC=CC=C12 | 10 | I |
| C[C@@]12CC3C[C@@](C)(C1)CC(C3)(C2)[NH+]1CCN2CCC[C@@H]2C1 | 10 | I |
| SCC1(C[NH+]2CCC[C@H]3CCCC[C@@H]23)CCOCC1 | 10 | I |
| C[C@H]1C[NH+]2CCC[C@@H]2CN1CC1(CS)CCCCCC1 | 10 | I |
| CN1C2=CC=CC=C2C(Br)=C1C1=CC=[NH+]C=C1 | 10 | I |
| C1C[C@@H]2C[C@H]1[C@@H]1O[C@]([C@H]21)(C1=CC=CC=C1)C1=CC=C[NH+]=C1 | 10 | I |
| CC1=CC=C(F)C(C[NH+]2CCCC3(CCOCC3)C2)=C1F | 10 | I |
| CN(CC1=CC=CC=C1F)C1=C(Br)C=C(C)C=[NH+]1 | 10 | I |
| BrC1=CC2=CN=NC=C2C=C1 | 10 | I |
| IC1=CN=CN=C1 | 10 | I |
| COC(=O)[C@@H](C)Br | 10 | I |
| CCC1=CC=C(C=C1)S(C)(=O)=O | 10 | I |
| BrCCN1C=CC=C1 | 10 | I |
| C[C@]1(Cl)CS(=O)(=O)C[C@@H]1Cl | 10 | I |
| CC(C)[C@@H](Br)C(C)=O | 10 | I |
| FC1(F)OS(=O)(=O)C1(F)F | 10 | I |
| N#CC1=C2OCCOC2=CS1 | 10 | I |
| CC1(C)OC[C@@H](CBr)O1 | 10 | I |
| CC1=NC=NC(C)=C1Br | 10 | I |
| C[N+]([O-])=CC1=CC=C(Br)C=C1 | 10 | I |
| CC(=C)[C@H]1C[C@@H](C(C)=O)C(=O)C1 | 10 | I |
| IC1=CC=COC1=O | 10 | I |
| C[C@@H]1CCC[C@@H](C)N1C[C@@H]1CO1 | 10 | I |
| O=C1SC(=O)[C@@H]2[C@H]3CC[C@H](O3)[C@H]12 | 10 | I |
| ClC1=C[C@@H]2N=CN=C2C=C1Cl | 10 | I |
| FCC(=O)[C@@]1(F)CCCCC1=O | 10 | I |
| CSC1(SC)[C@H](C)CCC1=O | 10 | I |
| C[C@H]1[C@@H](C)N(C)[C@H](C)[C@@H](C)N1C | 10 | I |
| CC1(C)[C@@]2(C)CC[C@@]1(OC2=O)C=C | 10 | I |
| CCN1C=CN=C1Br | 10 | I |
| BrCC(=O)CBr | 10 | I |
| C[C@H]1SC2=NC=NC(=S)C2=C1C | 10 | I |
| C[C@H](CN1CCSC[C@H]1C)C#N | 10 | I |
| C[C@H]1CN(CCC#N)C[C@H](C)S1 | 10 | I |
| O=S1(=O)C[C@H]2CC=CC[C@H]2C1 | 10 | I |
| CCCN1C=NC(Br)=C1 | 10 | I |
| FC(F)N1C=CC(CCl)=N1 | 10 | I |
| O=C1CCO[C@]2(CCSC2)C1 | 10 | I |
| IC1=NC=CO1 | 10 | I |
| CC(=O)[C@H]1C[C@@H]1[C@@H]1CCCOC1 | 10 | I |
| CC1=NC=CN1CC(Br)=C | 10 | I |
| CC[S@](=O)[C@@H]1CCCC[C@@H]1C#N | 10 | I |
| CC[S@](=O)[C@H]1CCCCC[C@@H]1C#N | 10 | I |
| C[S@](=O)[C@H]1CCCCC[C@@H]1C#N | 10 | I |
| O=C1CC[C@@H](C1)SC1CCOCC1 | 10 | I |
| C[C@H](Br)[C@H](C)C1=NC=NS1 | 10 | I |
| COC[C@@H](C)I | 10 | I |
| CC1(C)C[C@@H]1N1C=NC=C1CCl | 10 | I |
| CC1=CN=C(CBr)O1 | 10 | I |
| CCCN1CCC(=O)[C@H](C)[C@@H]1C | 10 | I |
| C[C@H]1CN2CCCC[C@@H]2CN1CCS | 10 | I |
| C[C@H](N1CCC(C)(C)C1)C(C)=O | 10 | I |
| Br[C@@H]1CCCO[C@H]1C#N | 10 | I |
| ClC[C@@H]1CC(Br)=NO1 | 10 | I |
| BrC1=C2C=NCC=C2N=C1 | 10 | I |
| FC(F)(F)CS(Cl)(=O)=O | 10 | I |
| FC(F)(F)C1=CN=C(N=C1)C#C | 10 | I |
| O=C1OC(=O)[C@H]2CC[C@@H]1C21CC1 | 10 | I |
| O=C1SCCC2=C1N1CCCC3=C1C2=CC=C3 | 10 | I |
| IC1=CC=CC(C=C(C#N)C#N)=C1 | 10 | I |
| CC1=C(Br)C=C(S1)C=C(C#N)C#N | 10 | I |
| C[C@@H]1CSC=NN1C1=CC=C(Br)C=C1 | 10 | I |
| ClC1=C(Cl)C(=O)C(=O)C(Cl)=C1Cl | 10 | I |
| CC1=CC=C(C)N1C1=CC=C(Br)C=N1 | 10 | I |
| CC1=NN(C(C)=C1)C1=CC=C(Br)C=C1 | 10 | I |
| O=C1C2=CC=CC3=CC=CC(=C23)[C@@]11OCCS1 | 10 | I |
| ClC1=NC(=NC2=C1C1=C(CCC1)S2)C1CC1 | 10 | I |
| O=S1(=O)C2=CC=CC=C2C=C1C1=CC=CC=C1 | 10 | I |
| ClCC1=CC2=C(OCCCO2)C(Br)=C1 | 10 | I |
| FC1=CC(Cl)=CC(=C1)C1=CC(Cl)=NC=N1 | 10 | I |
| C[C@H](Cl)C1=CC2=C(OCCO2)C=C1Br | 10 | I |
| C[C@@H](Cl)C1=CC2=C(OCCO2)C=C1Br | 10 | I |
| C[C@H](Cl)C1=CC2=C(OCCCO2)C=C1Cl | 10 | I |
| C[C@@H](Cl)C1=CC2=C(OCCCO2)C=C1Cl | 10 | I |
| [O-]C1=C(Cl)C(Cl)=C(Br)C=C1C=O | 10 | I |
| CC1=NN(C2CCCC2)C(C)=C1Br | 10 | I |
| Br[C@@H]1CCCC[C@@H]1[C@@H]1CCS(=O)(=O)C1 | 10 | I |
| FC1=CC=CC=C1C1=CC=C(Br)N=N1 | 10 | I |
| CC1(C)C[C@@]23CS(=O)(=O)[C@@H](Cl)[C@@]2(C1)CC=CC3 | 10 | I |
| CC1=CC(C(F)F)=C2C(Br)=NSC2=N1 | 10 | I |
| FC1=C(Br)C=CC(=C1)S(Cl)(=O)=O | 10 | I |
| BrC1=CC(N=C=O)=C(Br)C=C1 | 10 | I |
| FC(F)(F)C1=NC2=C(C=N1)C=C(Br)C=C2 | 10 | I |
| C[C@H]1CCC2=C3CC[C@H](C)C[C@@H]3C3=C([C@@H]2C1)C(=O)C3=O | 10 | I |
| CN1N=CC=C1[C@@H]1CCCC[C@@H](Br)C1 | 10 | I |
| C[C@@H]1CCC[C@H](C1)N1N=C(C)C(Br)=C1C | 10 | I |
| C[C@H](Cl)C1=NC2=C(C=C(C)S2)C(Cl)=N1 | 10 | I |
| FC1=C(Br)C=C(C=C1)C1=CC=NO1 | 10 | I |
| C[C@H](Cl)C1=NC2=CC(Cl)=C(F)C=C2N1C | 10 | I |
| C[C@@]12CC[C@@H]3[C@@H](CCC4=CC(=O)CC[C@@H]34)[C@@H]1CCC2=O | 10 | I |
| CC1=CN(N=C1I)C1CCCC1 | 10 | I |
| Cl[C@H]1CCC[C@H]1[C@@H]1CCO[C@]2(CCOC2)C1 | 10 | I |
| C[C@H]1CCC2=C(ON=C2CBr)[C@@H]1C | 10 | I |
| C[C@H]1CCC2=C(ON=C2CBr)[C@H]1C | 10 | I |
| CC1(C)CCC2=C(C1)C(CBr)=NO2 | 10 | I |
| ClC1=C(N=CC=N1)C1=C(Br)C=CS1 | 10 | I |
| CC1=C(Cl)N=C(N=C1Cl)[C@@H]1CCCS1 | 10 | I |
| CON1C(I)=CC2=CC=CC=C12 | 10 | I |
| FC1(F)OC2=CC=CC(CBr)=C2O1 | 10 | I |
| O=C1CCCCC[C@@H]1[C@@H]1CCO[C@]2(CCSC2)C1 | 10 | I |
| O=C1CCCCC[C@H]1[C@@H]1CCO[C@]2(CCSC2)C1 | 10 | I |
| C[C@H]1CCC[C@H](C1)N1CCCN2CCCC[C@@H]2C1 | 10 | I |
| C[C@H]1CC[C@H]([C@@H]1C)N1CCCN2CCCC[C@@H]2C1 | 10 | I |
| CC1(C)CSC[C@@](C1)(C#N)N1CCCCCC1 | 10 | I |
| IC1=CC=C(C=C1)C1=CN=CO1 | 10 | I |
| ClC1=CC(=C(Cl)S1)S(Cl)(=O)=O | 10 | I |
| C[C@H]1OC2=C(Cl)C=CC(Br)=C2C1=O | 10 | I |
| ClS(=O)(=O)C1C2=CC=CC=C2C2=CC=CC=C12 | 10 | I |
| BrCC1OCCCO1 | 10 | I |
| CN1C(C)=C(C)C(=S)N(C)C1=S | 10 | I |
| CO[C@H]1OCCC[C@@H]1Br | 10 | I |
| C[C@H](Br)C(=O)C(F)(F)F | 10 | I |
| CC1=CC(=S)N2C=CC=CC2=N1 | 10 | I |
| [N-]=[N+]=C1[C@@H]2C[C@H]3C[C@@H](C2)[C@H](C3)C1=O | 10 | I |
| CC(C)[C@@H](Br)C(=O)N(C)C | 10 | I |
| FC1(F)C[C@H](CC#N)C1(F)F | 10 | I |
| C\C(Cl)=C\CN1CCOCC1 | 10 | I |
| FC(F)(F)C(=O)C1=C[C@@H]2CC[C@H]1C=C2 | 10 | I |
| CO[C@@H]1C=CC[C@H]1Br | 10 | I |
| Br[C@@H]1C2CC(C2)C1=O | 10 | I |
| FC(F)C(=O)CBr | 10 | I |
| ClC1(CC1)S(=O)(=O)C1(Cl)CC1 | 10 | I |
| CN(CC#C)[C@@H]1CCCN(C)C1 | 10 | I |
| CO[C@@H]1C[C@@H](C)SC(SC)=N1 | 10 | I |
| [O-][N+]1=CC=C(C=C1)C1=CC=C(F)C=C1 | 10 | I |
| C[C@@H](Br)[C@H](C)C1=NC=CN1C | 10 | I |
| CC1=CN=C(I)N=C1 | 10 | I |
| BrCCC1=COC=C1 | 10 | I |
| Cl[C@H]1CCO[C@@H](C1)[C@@H]1CCOC1 | 10 | I |
| C[C@H]1CN(CC(C)=O)C[C@H](C)S1 | 10 | I |
| C[C@H](N1C[C@H](C)S[C@H](C)C1)C(C)=O | 10 | I |
| C[C@@H](CC#N)N1CCC[C@H](C)CC1 | 10 | I |
| CN1C=C(C=N1)[C@H]1CC[C@H](Cl)C1 | 10 | I |
| O=C1CCC2(CC1)SCCS2 | 10 | I |
| CC1=CC=C[C@H]2CS(=O)(=O)C[C@@]12C | 10 | I |
| CCN1C=CC(CBr)=N1 | 10 | I |
| SCC1=CC2=C(OCCO2)C=C1 | 10 | I |
| F[C@@H]1CCCN(CCCl)C1 | 10 | I |
| C[C@@H](Cl)C[C@H]1CCS(=O)(=O)C1 | 10 | I |
| BrC(=C)CN1C=CC=N1 | 10 | I |
| CC(C)[S@@](=O)[C@H]1CCC[C@@H]1C#N | 10 | I |
| CN1C=C(CI)C=N1 | 10 | I |
| C\C=C(\C)CN1CCN(C)CC1 | 10 | I |
| CC1=C(C)N=C(CBr)O1 | 10 | I |
| C[C@H]1C[C@@H]1N1C=NC=C1CCl | 10 | I |
| O=C1[C@H]2C=C[C@@H]3C2C2[C@@H]1C=C[C@H]2C3=O | 10 | I |
| N#CCCN1CC2(C1)CCCCC2 | 10 | I |
| CN(CC(F)(F)F)C(Cl)=O | 10 | I |
| CCO[C@]1(CCCS[C@@H]1C)C#N | 10 | I |
| O=C1CCO[C@H](C1)C1=CC=C(C=C1)C1CC1 | 10 | I |
| SCCN1CCO[C@@H]2CCC[C@@H]12 | 10 | I |
| C[C@@H](Cl)[C@H](C)C1=CN=CN=C1 | 10 | I |
| BrCC(=O)[C@@H]1CCCCS1 | 10 | I |
| BrC1=CCC2=C(C=O)C=NC2=C1 | 10 | I |
| FC1=CCC2=C(Cl)N=NC2=C1 | 10 | I |
| FCC1=NO[C@@H]2CC(F)(F)C[C@@H]12 | 10 | I |
| BrC1(CCOCC1)C=O | 10 | I |
| FC1=CN=C(N=C1)C(F)(F)F | 10 | I |
| CSC1=C(C#N)C2(CCC(C)CC2)[C@H](C#N)C(=O)N1 | 10 | I |
| OC1=CC=C(C=C1)N1[C@@H]2CS(=O)(=O)C[C@@H]2SC1=S | 10 | I |
| BrC1=CC=CC=C1NC(=O)[C@@H]1COCCO1 | 10 | I |
| CN1CC(=O)N[C@H](C1=O)C1=CC=C(Br)C=C1 | 10 | I |
| CC[C@]1(C)CC2=C3C(S[C@H]2CO1)=NC(=S)NC3=O | 10 | I |
| CCNC(=O)C1=CC2=C(OCCO2)C=C1Br | 10 | I |
| BrC1=C(SC2=NC=CC(=O)N2)N=CC=C1 | 10 | I |
| CN1N=CC=C1NC(=O)C1=CSC(Br)=C1 | 10 | I |
| CC1=CC=C2N(C(=S)NC2=C1)[C@]1(C)CCS(=O)(=O)C1 | 10 | I |
| C[C@]1(CCS(=O)(=O)C1)N1C(=S)NC2=C(F)C=CC=C12 | 10 | I |
| O=C1N(C[C@H]2CCCOC2)C(=S)NC2=C1SC=C2 | 10 | I |
| CC1=NN(C(C)=C1Br)C1=CC=NC(CO)=C1 | 10 | I |
| CC1=NN([C@@H]2CCCCNC2=O)C(C)=C1Br | 10 | I |
| CNC(=O)[C@H](Cl)C1=CC2=C(OCCO2)C(Cl)=C1 | 10 | I |
| C[C@@H]1CN([C@H](C)CN1)S(=O)(=O)C1=CC=C(F)C=C1 | 10 | I |
| O[C@H](C1=CC2=C(OCCCO2)C=C1)C1=CC(F)=CN=C1 | 10 | I |
| C[C@@H]1CN(C[C@@H](C)S1)C1=C(CO)N2C=CSC2=N1 | 10 | I |
| O[C@@H]1[C@H](COC2=CC=CC=C12)N1C=C(C=N1)C(F)(F)F | 10 | I |
| BrC1=CSC(CNC(=O)N2C=CN=C2)=C1 | 10 | I |
| CN1C=CN=C1CN1C(=S)NC2=CC(Cl)=CC=C12 | 10 | I |
| CN1C(=CC2=CC(F)=CC=C12)C(=O)NC1=NC=CS1 | 10 | I |
| BrC1=CC=CC(CN2CC(=O)NC(=O)C2)=C1 | 10 | I |
| CN1N=C(C)C(Br)=C1C[C@]1(O)CCCOC1 | 10 | I |
| BrC1=C(N=C(NC1=O)[C@H]1CCCO1)C1CC1 | 10 | I |
| IC1=CN=CN=C1O[C@@H]1CCNC1 | 10 | I |
| CC1(C)[C@@H](N[C@@H]2CCCS(=O)(=O)C2)[C@H]2CCCO[C@H]12 | 10 | I |
| C[C@@H]1NC(=O)[C@@H](C)N(CC2=CC=C(Cl)S2)C1=O | 10 | I |
| C[C@@H]1NC(=O)C2(CCCC2)N(CC2=CSC=C2)C1=O | 10 | I |
| C[C@@H]1NC(=O)C2(CCCCC2)N(CC(F)(F)F)C1=O | 10 | I |
| C[C@@H]1CC2=CC(=CC=C2O1)[C@@H](O)[C@@H]1CCS(=O)(=O)C1 | 10 | I |
| C[C@@]1(CCS(=O)(=O)C1)N1CCCN[C@](C)(C1)C1CC1 | 10 | I |
| C[C@@H]1NC(=O)[C@@H](C)N(C1=O)C1=CC=C(Br)C=C1 | 10 | I |
| C[C@H](N1[C@@H](C)C(=O)N[C@@H](C)C1=O)C1=CC=C(Cl)S1 | 10 | I |
| CC[C@@H]1NC(=O)[C@@H](C)N(C1=O)C1=CC(F)=C(F)C=C1F | 10 | I |
| CC[C@H]1NC(=O)CCN(C2=CC(F)=C(F)C(F)=C2)C1=O | 10 | I |
| C[C@@H]1N(C(=O)CNC1=O)C1=CC(=CC=C1F)C(F)(F)F | 10 | I |
| C[C@H]1N(C2CCC(C)(C)CC2)C(=O)[C@H](NC1=O)C1CC1 | 10 | I |
| C[C@H]1CC[C@H](C[C@@H]1C)N1[C@H](C)C(=O)N[C@H](C2CC2)C1=O | 10 | I |
| C[C@@H]1CN([C@@H](C)CN1)S(=O)(=O)C1=C(F)C=CC=C1F | 10 | I |
| C[C@@H]1CN(CCN1)S(=O)(=O)C1=C(C)SC(C)=C1 | 10 | I |
| COC1=CC=C(C=C1F)[C@H]1CS(=O)(=O)C[C@@H](C)CN1 | 10 | I |
| CC1=CC(N[C@]2(CCCS(=O)(=O)C2)C#N)=CC(F)=C1 | 10 | I |
| CN1CCO[C@@H](CN2C(=S)NC3=C(F)C=CC=C23)C1 | 10 | I |
| CN1C=C(Br)C=C(NC(=O)C(C)(C)F)C1=O | 10 | I |
| FC1=CC=C(C=C1)[C@@H]1C(=O)NC(=O)CC11CCOCC1 | 10 | I |
| O[C@H](C1=CC2=C(OCCCO2)C=C1)C1=CC=C(F)C=N1 | 10 | I |
| C[C@H]1CCC[C@@H](N1)C(=O)N1C[C@@H]2CCCCN2C[C@@H]1C | 10 | I |
| O[C@H]([C@H]1CSCCO1)C1=CC2=C(OCCCO2)C=C1 | 10 | I |
| CS(=O)(=O)[C@H]1CCC[C@@H](C1)[NH+]1CCCC\C1=C/[O-] | 10 | I |
| CC1(C)NCC(=O)N(CC2=CC=C3CCCC3=C2)C1=O | 10 | I |
| CC(=O)[C@H]1CC[C@H]2[C@@H]3CC[C@H]4CC(=O)CC[C@]4(C)[C@@H]3C(=O)C[C@]12C | 10 | I |
| CC(=O)[C@H]1CC[C@H]2[C@H]3CC(=O)[C@H]4CC(=O)CC[C@]4(C)[C@@H]3CC[C@]12C | 10 | I |
| BrC1=CC=C(C=C1)S(=O)(=O)N1CCSCC1 | 10 | I |
| C[C@H]1SCCN1S(=O)(=O)C1=CC=C(Br)C=C1 | 10 | I |
| BrC1=CC=C(CN2N=CC(=O)C3=CC=CC=C23)C=C1 | 10 | I |
| CC(=O)[C@@H]1CC[C@H]2[C@@H]3CC(=O)C4=CC(=O)CC[C@@]4(C)[C@@H]3CC[C@]12C | 10 | I |
| FC1=CC=C(C=C1)N1C(=O)C2=C(C1=O)C(F)=C(F)C(F)=C2F | 10 | I |
| CN1CCN(CC1)C(=O)C12C[C@H]3C[C@H](CC(Br)(C3)C1)C2 | 10 | I |
| CC(C)[C@@]1(C)CC(=O)N(C1=O)C1=CC=C(Br)C=C1 | 10 | I |
| CC[C@H]1CO[C@H](C)CN1C(=O)C1=CSC(Br)=C1 | 10 | I |
| BrC1=CC=CC(=C1)N1C(=O)[C@H]2[C@H]([C@@H]3CC[C@H]2C=C3)C1=O | 10 | I |
| ClC1=CC=CC=C1[C@H]1CS\C(S1)=C(\C#N)N1C=CN=C1 | 10 | I |
| CN(C)\C=C1/SC(=S)N(C1=O)C1=CC=C(Br)C=C1 | 10 | I |
| CN1C(=O)CC2=CC(=CC=C12)[C@H](Br)[C@@H]1CCCO1 | 10 | I |
| FC1=C(CN2C(=O)CC3(CCCC3)C2=O)C=C(Br)C=C1 | 10 | I |
| Br[C@@H]([C@@H]1CCS(=O)(=O)C1)C1=CC2=C(OCC2)C=C1 | 10 | I |
| CN1C(=O)CC2=CC(=CC=C12)[C@H](Br)[C@H]1CCCCO1 | 10 | I |
| CN1C(=O)CC2=CC(=CC=C12)[C@@H](Br)[C@H]1CCCCO1 | 10 | I |
| FC1=C(F)C2=C(C=C1)N=C(CCl)N2[C@@H]1CCN2CCC[C@H]12 | 10 | I |
| ClC1=CC=CC=C1[C@@H]1CS\C(S1)=C(/C#N)N1C=CN=C1 | 10 | I |
| C[C@@H]1CC2=CC(=CC=C2O1)[C@H](Br)[C@@H]1COCCO1 | 10 | I |
| BrC1=CC=C(C=C1)N1C(=O)C[C@]2(C[C@H]3C[C@@H]2C=C3)C1=O | 10 | I |
| CN1C(=O)CCC2=CC(=CC=C12)[C@H](Br)[C@H]1CCCOC1 | 10 | I |
| IC1=NN=C(S1)[C@@H]1COC2=CC=CC=C12 | 10 | I |
| CC1=CN(C=N1)C1=NC(Br)=C(Br)S1 | 10 | I |
| FCCN1C=NC2=C(C=C(I)C=C2)C1=O | 10 | I |
| CC1(C)[C@H]2[C@@H]1C(=O)N(CC1=CC=C(Br)C=C1F)C2=O | 10 | I |
| C[C@@H]1CC2=CC(=CC=C2O1)[C@@H](Br)[C@@H]1CCS(=O)(=O)C1 | 10 | I |
| C[C@H]1CC2=CC(=CC=C2O1)[C@@H](Br)[C@@H]1CCS(=O)(=O)C1 | 10 | I |
| FC1=CC(I)=C(C=C1)N1C(=O)C=CC1=O | 10 | I |
| CC1=NN(CC2=CC=C(I)C=C2)C(C)=N1 | 10 | I |
| ClC1=C(CN2C(=O)[C@H]3C[C@H]3C2=O)C=CC(Br)=C1 | 10 | I |
| CC(C)[C@@H]1CCCN1C1=C(I)C=NC=N1 | 10 | I |
| CN1CCO[C@@H]2CN(CC3=CC(Cl)=CC=C3Br)C[C@H]12 | 10 | I |
| IC1=CN(C[C@H]2CCCCO2)C(=O)C=C1 | 10 | I |
| CO[C@H]1CCC[C@H](C1)N1N=C(C)C(I)=C1C | 10 | I |
| CSC1(CCN(CC1)[C@H]1CCO[C@]2(CCSC2)C1)C#N | 10 | I |
| CSC1(CCN(CC1)[C@H]1CCO[C@@]2(CCSC2)C1)C#N | 10 | I |
| ClC1=NC(=NC(C2CC2)=C1Br)[C@H]1CSCCO1 | 10 | I |
| ClC1=C(Br)C=C(C=C1)N1CCCS1(=O)=O | 10 | I |
| CCN1CCN(C[C@H]1C)C(=O)C1=C(F)C=C(Br)C=C1F | 10 | I |
| C[C@]1(CCCO1)[C@H](Br)C1=CC=C2OCCOC2=C1 | 10 | I |
| FC(F)(F)[C@@H]1N(CCC2=C1N=C1C=CC=C[C@@H]21)C(=O)CCl | 10 | I |
| IC1=CC=CC(=C1)C1=N[C@H]2C=NN=C2S1 | 10 | I |
| BrC1=CC=C(CN2C(=O)[C@H]3CC=CC[C@@H]3C2=O)C=C1 | 10 | I |
| Cl[C@@H]1CS(=O)(=O)C[C@@H]1\N=C(\Cl)C(Cl)(Cl)Cl | 10 | I |
| Cl[C@H]1CS(=O)(=O)C[C@@H]1\N=C(\Cl)C(Cl)(Cl)Cl | 10 | I |
| Cl[C@H]1CS(=O)(=O)C[C@@H]1\N=C(/Cl)C(Cl)(Cl)Cl | 10 | I |
| C[C@@H]1[C@@H](C)C(=O)N(CC2=CC=C(I)C=C2)C1=O | 10 | I |
| CC(C)\N=C1\S[C@H]2CS(=O)(=O)[C@H](Br)[C@]2(C)S1 | 10 | I |
| C[NH+]1CCC(CC1)C1=CC=C2C(CSC3=C(C=CS3)C2=O)=C1 | 10 | I |
| C1C[C@@H]2C[C@H]1C[C@@H]2[NH+]1CCN(CC1)C1C2CC3CC(C2)CC1C3 | 10 | I |
| BrC1=CC=CC=C1OCC1=CC=C2[NH+]=CC=CC2=C1 | 10 | I |
| O=C1C2=C(C=CS2)C2=C(SC=C2)C=C1CC1=C[NH+]=CC=C1 | 10 | I |
| FC1=CC=CC2=C1[NH+]=C(CCCl)N2C1=C(F)C=CC=C1F | 10 | I |
| BrC1=CC(CN2C(=[NH+]C3=CC=CC=C23)C2CC2)=CC=C1 | 10 | I |
| BrC1=CC2=C(C=C1)C=C(OCC1=CC=[NH+]C=C1)C=C2 | 10 | I |
| CC1=CC=C2N(CC3=CC=C(F)C(F)=C3)C(CCl)=[NH+]C2=C1 | 10 | I |
| C[C@H]1C[C@H]1N1C(CCCl)=[NH+]C2=CC(Br)=CC=C12 | 10 | I |
| ClC1=CC=C(S1)C(=O)C[NH+]1CCC2(CCCC2)CC1 | 10 | I |
| Br[C@H](CC1=C2C=CSC2=CC=[NH+]1)C1=CC=CC=C1 | 10 | I |
| C[C@H]1CC[C@H]([C@H](C)C1)N1C(CCCl)=[NH+]C2=CC(F)=CC=C12 | 10 | I |
| C[C@@H]1CC[C@@H]([C@H](C)C1)N1C(CCCl)=[NH+]C2=CC=C(F)C=C12 | 10 | I |
| C[C@@H](Cl)C1=[NH+]C2=CC=C(F)C(F)=C2N1[C@@H]1CCC[C@H]1C | 10 | I |
| FC1=CC=C(C[NH+]2CCO[C@H](C2)C2=CC=C(F)C(Cl)=C2)C=C1 | 10 | I |
| ClC1=CC=CC=C1C[NH+]1CCO[C@H](C1)C1=CC=CC=C1Cl | 10 | I |
| C[C@H](Br)CC(C)(C)C1=CC2=CC=CC=C2[NH+]=C1 | 10 | I |
| C[C@H]([C@H](Cl)C1=CC=C(Br)O1)C1=CC=[NH+]C=C1 | 10 | I |
| Cl[C@@H](CC1=CC=CC=[NH+]1)C1=CC=CC=C1I | 10 | I |
| C[C@@H]([C@@H](Br)C1=CC=C(C)S1)C1=CC=[NH+]C=C1 | 10 | I |
| C[C@H]1CC[C@@H]([C@@H]1C)N1C(CCCl)=[NH+]C2=C(F)C=CC=C12 | 10 | I |
| C[C@H]1CC[C@H]([C@@H]1C)N1C(CCCl)=[NH+]C2=C(F)C=CC=C12 | 10 | I |
| CC(C)(CBr)C[NH+]1CCC2(CCCCC2)CC1 | 10 | I |
| C[C@H]1CSCC[NH+]1CC1(CBr)CCCCCC1 | 10 | I |
| FC1=CC=CC=C1N1C(CCl)=[NH+]C2=CC=C(Br)C=C12 | 10 | I |
| CC1CC[NH+](CC2=CC=C(I)C=C2)CC1 | 10 | I |
| C[C@H]1C[C@H](C)C[NH+](CC2=CC=C(I)C=C2)C1 | 10 | I |
| CC1=[NH+]C(N2CCC[C@@H](C2)C(F)(F)F)=C(CCl)C(C)=C1 | 10 | I |
| C[C@@H]1CC[C@H](C)[NH+](CC2(CBr)CCCCC2)C1 | 10 | I |
| ClCC1=[NH+]C2=C(C=C(Br)C=C2)N1C1=CSC=C1 | 10 | I |
| C[C@@H](Cl)C1=[NH+]C2=CC=CC=C2N1C[C@@H]1CC2=C1C=CC=C2 | 10 | I |
| C[C@H]1O[C@H](C)[C@H]([C@H](Cl)CC2=CC=C3C=CC=CC3=[NH+]2)[C@@H]1C | 10 | I |
| FC1=CC=CC(F)=C1[C@@H](Cl)CC1=[NH+]C2=CC=CC=C2C=C1 | 10 | I |
| Cl[C@H](CC1=CC=[NH+]C=C1)C1=C(Cl)C=C(Br)C=C1 | 10 | I |
| C[C@H]1O[C@H](C)[C@H]([C@H](Br)CC2=CC=C3C=CC=CC3=[NH+]2)[C@@H]1C | 10 | I |
| Br[C@H](CC1=[NH+]C2=CC=CC=C2C=C1)[C@@H]1CC2=C1C=CC=C2 | 10 | I |
| CC1=C(OC=C1)[C@H](Br)CC1=CC=C2C=CC=CC2=[NH+]1 | 10 | I |
| CC1=C(C=CC=C1Br)[C@H](Br)CC1=CC=[NH+]C=C1 | 10 | I |
| CC1=CC=C(C[C@@H](Br)C2=CC=C(F)C=C2Cl)[NH+]=C1 | 10 | I |
| COC1=C(Br)C=C(C[NH+]2C[C@@H]3CCC[C@@H]3C2)S1 | 10 | I |
| C[NH+](CC1=CC=C(Cl)C(F)=C1)[C@@H]1[C@@H]2CCO[C@@H]2C1(C)C | 10 | I |
| CC1CCC(CC1)[NH+](C)CC1=CC(F)=CC(Br)=C1 | 10 | I |
| FC1=CC(Br)=CC(C[NH+]2CCC[C@H](CCl)C2)=C1 | 10 | I |
| CN1C(C[C@H]2CCCC[C@H]2Br)=[NH+]C2=CC=CC=C12 | 10 | I |
| C[C@@]12CC3C[C@@](C)(C1)CC(C3)(C2)[C@H](Cl)CC1=CC=CC=[NH+]1 | 10 | I |
| CC1=CC=C2[NH+]=C(CCl)N(C2=C1)C1=CC=C(Br)C=C1 | 10 | I |
| C[C@@H](Cl)C1=[NH+]C2=CC=C(F)C(F)=C2N1CC1=C(C)C=CS1 | 10 | I |
| CN(C)C1=CC=C(\C=[NH+]/C2=CC=C(I)C=C2)C=C1 | 10 | I |
| CC1(C)C[C@@H]1N1C(CCCl)=[NH+]C2=CC(Cl)=C(F)C=C12 | 10 | I |
| CCC1CCC(CC1)[NH+](C)C1=C(Br)C=C(C)C=N1 | 10 | I |
| CC(=O)[C@@H]1CC[C@@]2(C)[C@H](CC[C@H]3[C@H]4CCC(=O)[C@]4(C)CC[C@H]23)C1 | 10 | I |
| CC1=C(C)C(\C(C#N)=C(\C#N)C2=C(C)SC(C)=C2C)=C(C)S1 | 10 | I |
| FC1=CC=C(\C=C2\COC\C(=C/C3=CC=C(F)C=C3)C2=O)C=C1 | 10 | I |
| BrC1=CSC(\C=C2/COC3=CC=CC=C3C2=O)=C1 | 10 | I |
| CC(=O)[C@H]1CC[C@H]2[C@@H]3CC[C@@H]4CC(=O)CC[C@]4(C)[C@@H]3CC[C@]12C | 10 | I |
| CC1=CC(C(=O)C2=CC=C(I)C=C2)=C(C)O1 | 10 | I |
| ClC1=CN2C(Br)=CN=C2C(Br)=C1 | 10 | I |
| FC1=CC=C(\C=C2/OC3=CC=C(Br)C=C3C2=O)C=C1 | 10 | I |
| FC(F)(F)[C@@H](Cl)C1=CC2=C(OCCO2)C=C1Br | 10 | I |
| FC1=CC=C(C=C1)[C@H](Br)C1=CC=C2OCCOC2=C1 | 10 | I |
| FC1=CC(=CC(F)=C1)[C@H](Br)C1=CC2=C(OCCO2)C=C1 | 10 | I |
| FC1=CC=C(C=C1F)[C@H](Br)C1=CC2=C(OCCO2)C=C1 | 10 | I |
| BrC1=C(SC(=C1)C1=CC=NC=C1)C1=CC=NC=C1 | 10 | I |
| ClC1=C(C=NC2=C1C=C(Br)C=C2Br)C#N | 10 | I |
| CC1=C(C)C(C)=C([C@@H](Br)[C@H]2COCCO2)C(C)=C1C | 10 | I |
| C[C@H]1CN(C[C@@H](C)O1)C1=CC(Br)=CC=C1CCl | 10 | I |
| BrC1=CC=C(S1)C(=O)C1=CC2=C(COC2)C=C1 | 10 | I |
| BrC1=CC=CC=C1CN1C(=O)C=CC2=CC=CC=C12 | 10 | I |
| Br[C@H]([C@H]1COC2=CC=CC=C12)C1=CC=C2COCC2=C1 | 10 | I |
| CC1=CC([C@@H](Br)[C@@H]2COCCO2)=C(C)C=C1Cl | 10 | I |
| FC1=C(F)C(F)=C(OC2=CC(Br)=CN=C2)C(F)=C1F | 10 | I |
| BrC1=NN=C(S1)C1=CC(Br)=CC=C1 | 10 | I |
| Br[C@@H]1CCC[C@@H](C1)C1=CC2=C(OCCCO2)C=C1 | 10 | I |
| C[C@H]1C[C@H]1C1=NC(C2CC2)=C(I)C(Cl)=N1 | 10 | I |
| ClC[C@H]1CCO[C@H]1C1=CC(Br)=C(Br)O1 | 10 | I |
| BrC1=CSC(C(=O)C2=COC=C2)=C1Br | 10 | I |
| CC[C@H](C)N1C=C(Br)C(I)=N1 | 10 | I |
| CC1=C(C)C([C@@H](Br)C2=CC=C3COCC3=C2)=C(C)O1 | 10 | I |
| BrCC1=NOC(=C1)C1=CC=CC=C1Br | 10 | I |
| ClC1=NC(CC2CC2)=NC(Cl)=C1I | 10 | I |
| CC1=CC(Br)=C(S1)C(=O)C1=C(C)OC(C)=C1C | 10 | I |
| BrC1=C(C[C@@]2(COC3=CC=CC=C3C2)C#N)SC=C1 | 10 | I |
| C[C@@H]1CC[C@H](Cl)[C@@H](CC2=C(Br)C(C)=NN2C)C1 | 10 | I |
| ClC1=NC(=NC(C2CC2)=C1Br)[C@H]1CC2=CC=CC=C12 | 10 | I |
| FC1=CC=C(SC2=C(I)C=NC=N2)C=C1 | 10 | I |
| Cl[C@@H]([C@H]1CC2=CC=CC=C12)C1=CC2=C(OCCO2)C=C1Cl | 10 | I |
| FC1=C(F)C(F)=C(C2=C(CBr)N=CO2)C(F)=C1F | 10 | I |
| C[C@H](Cl)C1=NC2=CC=C(F)C(F)=C2N1C1C(C)(C)C1(C)C | 10 | I |
| CC1=NN(CC2=CC(F)=CC(Br)=C2)C(C)=C1Cl | 10 | I |
| IC1=CC2=C(C=NN2C2CCCC2)C=C1 | 10 | I |
| Br[C@@H]1CCCC[C@@H](C1)[C@@H]1CCOC2(CCOCC2)C1 | 10 | I |
| CC1=C(OC2=CC=C(F)C=C12)C(=O)C1=CC=C(F)C=C1Cl | 10 | I |
| C[C@@]1(CCCO1)[C@H](Br)C1=CC=C2OCCCC2=C1 | 10 | I |
| C[C@]1(CCCO1)[C@@H](Br)C1=CC=C2OCCCC2=C1 | 10 | I |
| CC1(C)CC2=CC(=CC=C2O1)[C@H](Br)[C@]1(C)CCCO1 | 10 | I |
| CC1(CCCC1)[C@H](Br)C1=CC=C2OCCOC2=C1 | 10 | I |
| FC1=C(C=CC(Br)=C1)C1=NC=C(Br)C=N1 | 10 | I |
| BrC1=CC=C(SC2(CC3(C2)CCOCC3)C#N)C=C1 | 10 | I |
| C[C@@H](Cl)C1=NC2=CC(Br)=C(F)C=C2N1C1CC1 | 10 | I |
| IC1=CC=C(O[C@@H]2CC(=O)C22CCCC2)C=C1 | 10 | I |
| FC1=C(F)C=C(C=C1)N1C(=O)C(Cl)=C(Cl)C1=O | 10 | I |
| FC(F)(F)C1=C(C=CC(Cl)=C1)N1C(=O)CSCC1=O | 10 | I |
| CC(=O)N1C=CC2=C1N=CC=C2I | 10 | I |
| CC(=C)CN1C(=O)C(=O)C2=C(Br)C=CC=C12 | 10 | I |
| CC1=C(C)C2=C(S1)N=CN(CCBr)C2=O | 10 | I |
| C[C@H]1CN(CCO1)C1=CC(Br)=CC=C1C=O | 10 | I |
| CC1(C)[C@@]2(C)CC[C@@]1(CC2=O)C(=O)N1CCCCCC1 | 10 | I |
| FC1=CC(F)=C2C(=O)C(=O)N(CC3=CSC=C3)C2=C1 | 10 | I |
| IC1=CC=C(C=C1)N1C(=O)[C@H]2C[C@H]2C1=O | 10 | I |
| ClC1=CC(Cl)=C(CN2CCCS2(=O)=O)C=C1 | 10 | I |
| ClC12C[C@@H]3C[C@H](C1)CC(C3)(C2)C(=O)N1CCOCC1 | 10 | I |
| CC#CCN1C(=O)C(=O)C2=C(Br)C=CC=C12 | 10 | I |
| CN(C)S(=O)(=O)C1=CC(Br)=CC=C1F | 10 | I |
| CC[C@H](Br)C(=O)N1C[C@@H](C)OC(C)(C)C1 | 10 | I |
| CN1C(=S)N=C2[C@H](SC=C2C2=CC=C(C)C=C2)C1=O | 10 | I |
| COC1=CC=C(Br)C=C1[C@H]1CC(=O)C=CO1 | 10 | I |
| FC1=CC(Br)=C(C=C1)N1C=NN=C1Cl | 10 | I |
| BrC1=CC=C(CN2C(=O)[C@H]3C[C@H]3C2=O)S1 | 10 | I |
| C[C@H]1CN(CCO1)C(=O)C1=C(Cl)SC(Cl)=C1 | 10 | I |
| CC(C)N1CCO[C@@H](C1)C1=CC=C(Br)C=N1 | 10 | I |
| C[C@@H]1COCCN1C(=O)[C@H]1CCCC[C@@H]1C(F)(F)F | 10 | I |
| CN([C@H]1CCSC1)C1=NN=C(Br)S1 | 10 | I |
| CC[C@@H](Br)C1=CC=C2N(C)C(=O)N(C)C2=C1 | 10 | I |
| ClCC1=NN=C(S1)C1=CC=C(Br)O1 | 10 | I |
| C[C@@H]1C[C@@H](C)[C@H](CN2CCCN3CCC[C@@H]3C2)C(=O)C1 | 10 | I |
| CON(C)C(=O)C1=C(F)C=C(Br)C=C1F | 10 | I |
| FC1=C(N=C(Cl)N=C1)C1=C(Br)C=CO1 | 10 | I |
| CC1=CC(C)=C(C)C(C(=O)[C@@H]2CCS(=O)(=O)C2)=C1C | 10 | I |
| CC1=CC(C)=C(C)C(C(=O)[C@H]2CCS(=O)(=O)C2)=C1C | 10 | I |
| C[C@H]1OC2=CC(I)=CC=C2N(C)C1=O | 10 | I |
| FC1=CN=CC(=C1)C(=O)C1=CC(Br)=CN=C1 | 10 | I |
| CC(C)C1=CC(=NC=N1)N1CCC[C@@H](Br)C1 | 10 | I |
| CN1N=C(C)C(Cl)=C1CN1CCC(Br)CC1 | 10 | I |
| C[C@H](Cl)C1=NN=C(S1)[C@@H]1CCOC2=C1C=CC=C2 | 10 | I |
| C[C@@H](N1CCCSCC1)C(=O)N1[C@H](C)CCC[C@H]1C | 10 | I |
| CC1=CC(C)=C2C(=O)C(=O)N(C\C(Cl)=C\Cl)C2=C1 | 10 | I |
| CN1C=CC=C1CN1CCO[C@@]2(CCC3=C2C=CC=C3)C1 | 10 | I |
| C[C@H]1OCC[C@H]1[C@H](Cl)C1=CC=C2N(C)C(=O)CC2=C1 | 10 | I |
| CC1CCC(CC1)C(=O)[C@H]1CCOC2(CCOCC2)C1 | 10 | I |
| CC1(C)CCC(CC1)C(=O)[C@@H]1CCO[C@@]2(CCOC2)C1 | 10 | I |
| FC1=CC=CC=C1S(=O)(=O)[C@H]1CCOC2(CCC2)C1 | 10 | I |
| O=C[C@H]1CCCN(C1)[C@@H]1CCOC2(CCSCC2)C1 | 10 | I |
| CC(C)[C@@H]1CN2CCCC[C@@H]2CN1[C@@H]1CCCN(C)C1 | 10 | I |
| O=C1CCCC[C@H]1SC1=CC2=C(OCCCO2)C=C1 | 10 | I |
| CCCN1C(C)=NC2=C1C(Br)=CC(=C2)C#N | 10 | I |
| CN1CCC[C@]2(C1)CN(CC1=CC=C(F)C=C1)CCO2 | 10 | I |
| FC1=CC(=C(F)C=C1)C1=CC(=CN=C1)C(=O)N1CCCC1 | 10 | I |
| CN1C=CC=C1\C=C1\CS\C(=C\C2=CC=CN2C)C1=O | 10 | I |
| ClC1=CC=C(CN2C(=O)[C@H]3CC=CC[C@@H]3C2=O)S1 | 10 | I |
| C[C@@H]1[C@@H](C)C(=O)N(CC2=C(Br)C=CS2)C1=O | 10 | I |
| BrC1=CC2=C(OC=C(C=O)C2=O)C=C1 | 10 | I |
| Br[C@H]1CS(=O)(=O)C[C@@H]1Br | 10 | I |
| CN1CCN(CC1)C(=O)C1=C(F)C(F)=C(F)C(F)=C1F | 10 | I |
| BrC1=CSC(CN2CCOCC2)=C1 | 10 | I |
| CC1=NN=C(O1)C1=CC(Br)=CC=C1F | 10 | I |
| BrC1=CN(N=C1)C1=CC=C(C=C1)C#N | 10 | I |
| CC1=NN([C@H]2CCCCO2)C(C)=C1Br | 10 | I |
| BrC1=CSC(=N1)N1C=CC=CC1=O | 10 | I |
| BrC1=CC(=CC=N1)N1C=CC=CC1=O | 10 | I |
| CN(C)C(=O)[C@H](Cl)C1=CC(Br)=CC=C1F | 10 | I |
| CC1=C(C)N(C=N1)C1=CC=C(Br)C=N1 | 10 | I |
| CN(C)C(=O)[C@H](Cl)C1=CSC2=C1C=CC=C2 | 10 | I |
| CN(C)C(=O)[C@@H](Cl)C1=CSC2=C1C=CC=C2 | 10 | I |
| C[C@@H]1CC[C@H](C#N)[C@@H](C1)N1CCCN2CCC[C@@H]2C1 | 10 | I |
| ClC[C@@H]1CCCCN1C(=O)[C@@H]1CCSC1 | 10 | I |
| BrC1=NN=C(S1)C1CCOCC1 | 10 | I |
| CC1=CC=C(C=C1)S(=O)(=O)C(\Cl)=C\Cl | 10 | I |
| C[C@@H]1COCCN1C(=O)[C@@]1(C)CC1(Cl)Cl | 10 | I |
| O=C(C[C@@H]1C[C@H]2CC[C@@H]1C2)N1CCCSCC1 | 10 | I |
| CC1(C)CN(CCS1)C(=O)[C@H]1C[C@H]2CC[C@@H]1O2 | 10 | I |
| CC1=NC(N2CCCSCC2)=C(C)C(Cl)=N1 | 10 | I |
| C[C@@H](C#N)[S@@](=O)C1=CC=CC=C1Br | 10 | I |
| C[C@@H](I)C(=O)N1CCCC1 | 10 | I |
| CC1=C(C)C=C(CN2CC[C@@H](Br)C2=O)S1 | 10 | I |
| C[C@H]1[C@@H](CCC1=O)[S@@](=O)C1=CC=C2CCCC2=C1 | 10 | I |
| C[C@H]1[C@H](CCC1=O)[S@@](=O)C1=CC=C2CCCC2=C1 | 10 | I |
| IC1=CC=CC(=C1)N1CC(=O)CC1=O | 10 | I |
| C[C@H]1O[C@H](C)[C@@H]([C@@H]1C)C(=O)N1CCC[C@H](Cl)C1 | 10 | I |
| BrC[C@@H]1CCCCN1C(=O)[C@@H]1CCSC1 | 10 | I |
| BrC[C@H]1CCCN1C(=O)[C@H]1CCCCS1 | 10 | I |
| CC1=NSC(=N1)N1CC[C@@H](Br)C1 | 10 | I |
| CC1=C(C)N=C(S1)N1CC[C@@H](Br)C1 | 10 | I |
| CN(C)C(=O)[C@@H](Cl)C1=CC=C(F)C(Cl)=C1 | 10 | I |
| CC1=CC2=NC(CBr)=CC(=O)N2C=C1 | 10 | I |
| C[C@H]1CN(C[C@@H](C)S1)C1=CC=NC=C1CCl | 10 | I |
| CC1(C)C(C(=O)N2CC[S@](=O)C(C)(C)C2)C1(C)C | 10 | I |
| C[C@H]1[C@@H](CCC1=O)[S@@](=O)C1=CC=C(Cl)C(Cl)=C1 | 10 | I |
| C[C@H]1CN(CC2=NC(C)=C(C)S2)C[C@H](C)S1 | 10 | I |
| C[C@@H]1CN(CC2=C(C)N=C(C)S2)C[C@@H](C)S1 | 10 | I |
| O=C1CCN(CC1)[C@@H]1CCO[C@]2(CCSC2)C1 | 10 | I |
| O=C1CCC[C@@H](C1)[C@H]1CCOC2(CCOCC2)C1 | 10 | I |
| FC1=CC=CC=C1[S@@](=O)[C@H]1CCOC2(CCSCC2)C1 | 10 | I |
| FC1=CC=CC=C1[S@](=O)[C@H]1CCOC2(CCSCC2)C1 | 10 | I |
| C[C@@H]1CN2CCCC[C@@H]2CN1[C@@H]1CCCN(C)C1 | 10 | I |
| C[C@H]1CN2CCCC[C@@H]2CN1[C@@H]1CCC[C@@H]1C#N | 10 | I |
| C1C[C@H]2CN(CCCN2C1)[C@@H]1CCN2CCC[C@H]12 | 10 | I |
| C[C@H]1CN2CCC[C@@H]2CN1[C@@H]1CCCC[C@@H]1C#N | 10 | I |
| C[C@H]1CC[C@H](C#N)[C@@H](C1)N1CCN2CCC[C@@H]2C1 | 10 | I |
| C[C@@H]1CC[C@@H](C#N)[C@@H](C1)N1CCN2CCC[C@@H]2C1 | 10 | I |
| C[C@@H](F)C1=NC=NC(=O)[C@H]1I | 10 | I |
| CN(C)C(=O)CSC1=NC(C)=NC2=C1C1=C(CCCC1)S2 | 10 | I |
| CC1=C(Br)C=C(C(=O)N(CC#N)CC#N)C(C)=C1C | 10 | I |
| CCC1=C(C2=CC=CC=C2)[N+]([O-])=C(C(C)=[N+]1[O-])C1=CC=C(C)C=C1 | 10 | I |
| CCOC(=O)C1=CC(=O)C2=CC(F)=CC(Br)=C2O1 | 10 | I |
| ClC1=CC(C(=O)CN2CCS(=O)(=O)CC2)=C(Cl)S1 | 10 | I |
| BrC1=CC(C=O)=C(O[C@H]2CCS(=O)(=O)C2)C=C1 | 10 | I |
| CN(CC1=CC=C(Br)O1)C(=O)C1=CC=C(C=C1)C#N | 10 | I |
| CN(CC1=CC(Br)=CS1)C(=O)C1=COCCO1 | 10 | I |
| FC1=CC=C(C=C1Cl)S(=O)(=O)N1CCN(CC#C)CC1 | 10 | I |
| CN(CC1=CSC(Br)=C1)C(=O)C1=CN=NS1 | 10 | I |
| CN(CC1=NN=C(C)N1C)C1=CC=C(Br)C=C1F | 10 | I |
| CN1C(Cl)=C(Cl)C=C1C(=O)N1CCN(CCC#C)CC1 | 10 | I |
| COC1=CC=C(Br)C=C1N1C(=O)CC2(CCCC2)CC1=O | 10 | I |
| CC1=NC(=O)N(CC(=O)C2=CC=CC(Br)=C2)C(C)=C1 | 10 | I |
| C[C@H](Cl)C1=NC2=CC=C(F)C(F)=C2N1[C@H](C)C(=O)N(C)C | 10 | I |
| CN(CC1=CC=C(F)C=C1)C(=O)[C@H]1CS[C@@]2(C)CCC(=O)N12 | 10 | I |
| FC(F)C1=NN([C@H]2CCS(=O)(=O)C2)C(Cl)=C1CCl | 10 | I |
| CN1N=C(C[C@H]1C1=CSC=C1)C1=CC=C(C=C1)S(C)(=O)=O | 10 | I |
| CN(CC1=C(C)C=CS1)C(=O)[C@@H]1CS[C@@]2(C)CCC(=O)N12 | 10 | I |
| CN1N=C(C)C(CCl)=C1N1C=C(I)C=N1 | 10 | I |
| CC1=CC=C(C=C1C)N([C@@H]1CS(=O)(=O)C=C1)C(=O)CCl | 10 | I |
| CN(CC1=CC=C(Cl)N=C1)C(=O)C1=COC(Br)=C1 | 10 | I |
| CS(=O)(=O)[C@@H]1CSCCN1C1=NC(Br)=CS1 | 10 | I |
| CC1=NSC(SC2=C(I)C=NC=N2)=N1 | 10 | I |
| C[C@@H]1C[C@H]1C1=CC=C(O1)\C=C\C(=O)N1CC[S@@](=O)C(C)(C)C1 | 10 | I |
| ClCC1=C(C=CS1)S(=O)(=O)N1CCO[C@H]2CCC[C@H]12 | 10 | I |
| CN1N=C(C)C(Br)=C1CSC1=NN=C(C)S1 | 10 | I |
| CN1N=C(C)C(Br)=C1CN1CCC[C@H](C1)C(C)=O | 10 | I |
| CC(C)N1CCN(CC2=C(Br)C(C)=NN2C)CC1 | 10 | I |
| CCN1N=C(C)C(Br)=C1CN1C[C@H](C)OC[C@@H]1C | 10 | I |
| CCN1N=C(C)C(Br)=C1CN1CCCO[C@H](C)C1 | 10 | I |
| CCN1N=C(C)C(Br)=C1CN1CCCO[C@@H](C)C1 | 10 | I |
| CC1=CC2=C(S1)N=CN(CC(=O)C1=CC=C(F)C=C1F)C2=O | 10 | I |
| C[C@H]1CSC2=CC=CC=C2N1C(=O)CCC1=C(C)N(C)N=C1 | 10 | I |
| CN1N=CC(CCC(=O)N2CCCC3=C2C(F)=CC=C3F)=C1C | 10 | I |
| O=C(CN1CCOC[C@H]1C1CC1)N1CCSC2=CC=CC=C12 | 10 | I |
| COCC1=C(Br)C(Cl)=NC(=N1)[C@@H]1CCCOC1 | 10 | I |
| CC[C@@H]1CC[C@H](C#N)[C@@H](C1)N1CCSC[C@@H]1S(C)(=O)=O | 10 | I |
| CN1CCCN(CC1)S(=O)(=O)C1=CC=C(CCl)C=C1F | 10 | I |
| C[C@@H]1[C@@H](C)S(=O)(=O)CCN1CC1=CN=C(S1)C1CCCC1 | 10 | I |
| CCN1C=CC(=O)N(CC2=CC(F)=CC(Br)=C2)C1=O | 10 | I |
| CC[C@@H]1CN(C(=O)C2=CC(Br)=CN2CC)C(=O)C1 | 10 | I |
| O=C1N(CC[C@]11CCCN(CC2=CC=NC=C2)C1)C1=CSC=C1 | 10 | I |
| O=C1N(CC[C@@]11CCCN(CC2=CC=NC=C2)C1)C1=CSC=C1 | 10 | I |
| C([C@@H]1CCCO1)N1CCC2(CCCN(C2)C2=CN=CC=C2)CC1 | 10 | I |
| C[C@@H]1CN(CC2=CC=NC=C2)C[C@]11CCN(C2=CSC=C2)C1=O | 10 | I |
| C(N1CCOC[C@@]2(CCN(C2)C2=CC=CC=N2)C1)C1=CSC=C1 | 10 | I |
| CN(C)\C=C(/C#N)S(=O)(=O)C1=CC=C(Br)S1 | 10 | I |
| C[C@H]1[C@H](C)S(=O)(=O)CCN1CC1=CC(Cl)=CC2=C1OCC2 | 10 | I |
| BrC\C=C/CN1C(=O)C2=CC=CC=C2S1(=O)=O | 10 | I |
| CC1=CC(C=O)=C(C)N1C1=CC=C(Br)C=C1 | 10 | I |
| O=C1C=C2CCCC[C@@]22[C@H]1[C@H]1C(=O)[C@@H]2C2=C1CCCC2 | 10 | I |
| C[C@]12CC[C@H]3[C@@H](CC[C@@]45C[C@@]34C=CC(=O)C5)[C@@H]1CCC2=O | 10 | I |
| SCCC1=CC2=C(OC1=O)C=CC(Br)=C2 | 10 | I |
| O=S1(=O)C=C(C(=C1)C1=CC=CC=C1)C1=CC=CC=C1 | 10 | I |
| IC1=C2C=CC=NC2=C2N=CC=CC2=C1 | 10 | I |
| SC1=NC(=O)\C(S1)=C\C1=CC(Br)=CS1 | 10 | I |
| ClC[C@@H]1CO[C@@H](O1)C1=CC=C(Br)C=C1 | 10 | I |
| CN1C(CCCl)=NC2=CC(Br)=CC=C12 | 10 | I |
| FC(F)(F)C1=C(C#N)C(=S)OC(=C1)C1=CC=CS1 | 10 | I |
| C[C@]12CC[C@H]3[C@H](CC[C@H]4CC(=O)CC[C@@]34C)[C@H]1CCC2=O | 10 | I |
| BrC1=CC=CC2=C(SCC#N)C=CN=C12 | 10 | I |
| C[C@@H]1CN(CC2=CC=C(F)C(Br)=C2)[C@H](C)CO1 | 10 | I |
| CC1=CC(=CC(C)=C1)[C@H](Cl)[C@@H]1CCS(=O)(=O)C1 | 10 | I |
| CC1=CC(=CC(C)=C1)[C@@H](Cl)[C@H]1CCS(=O)(=O)C1 | 10 | I |
| C[C@H]1CN(CC2=CC(Br)=CS2)CCO1 | 10 | I |
| FC(F)(F)C1=CN=CC(I)=N1 | 10 | I |
| C[C@@H](Br)[C@H](C)C1=C2C=CSC2=NC=N1 | 10 | I |
| C[C@H](Br)[C@H](C)C1=C2C=CSC2=NC=N1 | 10 | I |
| CC(C)[C@@H](C)C1=NN=C(I)S1 | 10 | I |
| C[C@H]1CN(C[C@@H](C)S1)C1=C(F)C=C(C=O)C=C1F | 10 | I |
| Br[C@@H]1C=C(C#N)[C@]23CCC[C@]12C(=CC3)C#N | 10 | I |
| ClC1=NC=NC2=C1C(I)=CS2 | 10 | I |
| Br[C@@H]1CCN(C1=O)C1=CC2=C(SC=C2)C=C1 | 10 | I |
| C[C@H]1CCC[C@H](C1)[S@@](=O)[C@@H]1C[C@H](C)CC[C@@H]1C#N | 10 | I |
| CN1N=C(C)C(Br)=C1CC1=C[C@@H](Cl)CC1 | 10 | I |
| C[C@@H](Cl)CC1=C(I)C=NC=N1 | 10 | I |
| Br[C@H]1C[C@H](O[C@@H]2CCOC2)C11CCCCC1 | 10 | I |
| FC1=C(O[C@@H]2CCCC(=O)C2)C=CC(Br)=C1 | 10 | I |
| FC1=C(Br)C=CC(O[C@@H]2CCCC(=O)C2)=C1 | 10 | I |
| CC1=CC(=CC=C1F)[C@H](Br)[C@H]1COCCO1 | 10 | I |
| CCC1=C(C)C(Cl)=NC(=N1)[C@H]1CSCCS1 | 10 | I |
| C[C@H](OC1=CC=C(I)C=C1)C#N | 10 | I |
| C[C@@H](OC1=CC=C(I)C=C1)C#N | 10 | I |
| FC1=CN=C(C=C1)C(=O)C1=C(Br)C=CS1 | 10 | I |
| CC1=C(C)N=C(S1)N1CCC[C@H]1CBr | 10 | I |
| CO[C@H]1CCC[C@@H](C1)N1CCC[C@H](Br)C1 | 10 | I |
| CS(=O)(=O)C1=CC=C(Br)C=C1Cl | 10 | I |
| BrC1=CC=C(CN2C[C@@H]3CC[C@H](C2)O3)S1 | 10 | I |
| C[C@H]1OCC[C@@H]1[C@H](Br)C1=CC=C2COCC2=C1 | 10 | I |
| ClC1=NC(=NC2=C1CCCC2)[C@@H]1CSCCS1 | 10 | I |
| CC(C)C1=C(C)N=C(N=C1Cl)[C@@H]1CSCCS1 | 10 | I |
| IC1=CC2=CC(=CCC2=N1)C#N | 10 | I |
| BrC1=CC(OC2CC2)=C(Br)N=C1 | 10 | I |
| CC(C)(C)N1N=CC(CBr)=C1Br | 10 | I |
| COC1=C2C=CN=C2C(Br)=C(Br)C1 | 10 | I |
| IC1=CON=C1C1=CC=CC=C1 | 10 | I |
| C[C@@H]1OC2=C(Br)C=C(Br)C=C2C1=O | 10 | I |
| C[C@H]1SCCN([C@@H]1C)C1=C(F)C(CCl)=CC=N1 | 10 | I |
| C[C@@]12CC[C@H]3[C@H](CC[C@H]4CC(=O)CC[C@]34C)[C@H]1C=CC2=O | 10 | I |
| BrC1=[NH+]C2=C(C=C1)N=CC1=C2C=CS1 | 10 | I |
| CC(C)(C)C1=CC(=CC(=[O+]1)C(C)(C)C)C1=CC=CN1 | 10 | I |
| BrCCC[NH+]1CCC[C@H]2CCCC[C@H]12 | 10 | I |
| CCC1=[NH+]C=CN1C1=CC=CC(Cl)=C1CCl | 10 | I |
| CC[C@H]1CC[C@@H](C#N)[C@@H](C1)[NH+]1CCCSCC1 | 10 | I |
| CC1CCC(CC1)N(C)C1=C(CCl)C=CC=[NH+]1 | 10 | I |
| O=C(CC1=CC=CC=[NH+]1)C1=CSC2=CC=CC=C12 | 10 | I |
| C[C@@H]1CC[NH+](CC2=C(Br)C=CS2)C1 | 10 | I |
| C[NH+](CC#N)CC1=CC=C(Br)C=C1F | 10 | I |
| ClC1=NC(C[NH+]2C[C@H]3CC[C@@H]2C3)=C(Cl)C=C1 | 10 | I |
| BrCC[NH+]1CCC2(CCCCC2)CC1 | 10 | I |
| CC1=CC=C(C[C@H](Br)C2=CC=[NH+]C=C2)C=C1 | 10 | I |
| CN1C=C[NH+]=C1[C@H]1CCCCC[C@@H]1Br | 10 | I |
| CC1CCN(CC1)C1=[NH+]C[C@@H](CBr)S1 | 10 | I |
| C[C@@H]1CCN(C1)C1=[NH+]C[C@@H](CBr)S1 | 10 | I |
| CC1=[NH+]C=CN1CC1=CC(Br)=CC=C1F | 10 | I |
| C[C@H]1C[NH+](C[C@@H]2CCCCCC2=O)C[C@@H](C)S1 | 10 | I |
| FC1=CC=C(CN2C=[NH+]C3=C2C=C(F)C(F)=C3)C=C1 | 10 | I |
| C[NH+]1CCCC[C@H]1CCI | 10 | I |
| C[C@@H]1C[NH+](CC(C)(C)CBr)C[C@@H](C)O1 | 10 | I |
| CC1(C)CC[NH+](CC2(CBr)CCC2)CC1 | 10 | I |
| BrCC1(CN2C=[NH+]C3=CC=CC=C23)CC1 | 10 | I |
| Br[C@@H]1CCC[C@@H](CC2=CC=[NH+]C=C2)C1 | 10 | I |
| CCC1CC[NH+](C[C@H]2[C@H](C)C[C@H](C)CC2=O)CC1 | 10 | I |
| C[C@H]([C@H](Br)C1=CC=CO1)C1=CC=[NH+]C=C1 | 10 | I |
| C[C@@H]([C@H](Br)C(F)(F)F)C1=CC=[NH+]C=C1 | 10 | I |
| Br[C@H](CC1=CC=CC=[NH+]1)C1=CSC=C1 | 10 | I |
| BrC\C=C\C1=CC=C(C=C1)N1C=C[NH+]=C1 | 10 | I |
| C\C(=C\CCBr)C1=CC2=CC=CC=C2[NH+]=C1 | 10 | I |
| BrCC\C=C\[C@H]1CCCC2=C1[NH+]=CC=C2 | 10 | I |
| CC1=CC=C(CC(=O)C2=C(Cl)C=CS2)[NH+]=C1 | 10 | I |
| BrC1C[C@@H]2CC[C@H](C1)[NH+]2C[C@H]1CCCO1 | 10 | I |
| BrC1C[C@@H]2CC[C@H](C1)[NH+]2CCC1CC1 | 10 | I |
| BrC[C@@H]1CCCN(C1)C1=CC=[NH+]C=C1 | 10 | I |
| BrC[C@H]1CCCC[NH+]1C[C@@H]1CCCO1 | 10 | I |
| CC1=C(C)[NH+]=C(S1)N1CC[C@H](Br)C1 | 10 | I |
| Cl[C@H]1CC[NH+](CCC2=CC3=C(OCC3)C=C2)C1 | 10 | I |
| C[C@@H]1CC[C@H](C)[NH+](C[C@@]2(CCC[C@H](C)C2)C=O)C1 | 10 | I |
| Cl[C@H]1CCC[NH+](C[C@@H]2CSC3=CC=CC=C23)C1 | 10 | I |
| CC(C)C1=[NH+]C=CN1C[C@H]1CSC2=C1C=CC=C2 | 10 | I |
| C[C@H]1CC[C@H](C)[NH+](C[C@@H]2CSC3=C2C=CC=C3)C1 | 10 | I |
| ClC[C@H]1CCCO[C@@H]1C1=CC=[NH+]C2=CC=CC=C12 | 10 | I |
| CCCC1CCC(CC1)[NH+]1CCC(=O)[C@H](C)[C@@H]1C | 10 | I |
| C[S@](=O)C1=CC=C(C[NH+]2C[C@@H]3CCC[C@@H]3C2)C=C1 | 10 | I |
| CC1=CC(C)=C(C[NH+]2CC[C@H]3OCCC[C@@H]3C2)C=C1C | 10 | I |
| C[S@](=O)C1=CC=C(C[NH+]2CC(C)(C)C2(C)C)C=C1 | 10 | I |
| C[C@H]1CCC[C@@H](C1)[NH+]1CCCCC[C@@H]1CC(C)=O | 10 | I |
| CC(C)[NH+]1[C@H]2CC[C@@H]1CN(C[C@@H]1CCC=CC1)CC2 | 10 | I |
| CC1CCC(C[NH+](C)[C@H]2CCN(C)[C@H](C)C2)CC1 | 10 | I |
| C([NH+]1CCS[C@H]2CCCC[C@@H]12)C1=CCCOC1 | 10 | I |
| C[C@@H]1CC2=C(C)[NH+]=C3C=CC(Br)=CC3=C2O1 | 10 | I |
| COC1=CC=C(C[NH+]2CCSCC2)C=C1Br | 10 | I |
| IC1=CC=C(C[NH+]2CCOCC2)C=C1 | 10 | I |
| C[NH+]1CCC2(CC1)O[C@@H]1CCCC[C@@H]1C1=C2C=CS1 | 10 | I |
| FC(F)(F)COC1=C(I)C=CC=[NH+]1 | 10 | I |
| FC1=CC2=C(C=C1F)N(CC1=CC=CC=C1Cl)C=[NH+]2 | 10 | I |
| BrC1=CC=C(C=C1)[C@H](C#N)[NH+]1CCCCCC1 | 10 | I |
| C[C@H]1C[NH+](CC2=C(Br)C=CS2)C[C@@H](C)O1 | 10 | I |
| CCC1=C(C)N(CC2=C(Br)C=CS2)[NH+]=C1C | 10 | I |
| BrC1=CSC(C[NH+]2CCSCC2)=C1 | 10 | I |
| CC(C)(C)[C@@H]1CC[C@H](C#N)[C@@H](C1)[NH+]1CCCSCC1 | 10 | I |
| C[C@@H]1C[C@@H]1N1C(CCl)=[NH+]C2=CC(Br)=CC=C12 | 10 | I |
| ClCC1CC[NH+](CC2=CC(Br)=CS2)CC1 | 10 | I |
| C[C@@H](Br)C[C@H](C)C1=CC=CC2=C1C=C[NH+]=C2 | 10 | I |
| CC1(C)CC[NH+](C[C@@H](Br)C(F)(F)F)CC1 | 10 | I |
| FC(F)(F)[C@@H](Br)C[NH+]1CCO[C@H]2CCC[C@H]12 | 10 | I |
| C[C@H]1CN(CC2(CBr)CCCC2)CC[NH+]1C | 10 | I |
| C[C@@H]1CSCC[NH+]1CC1(CBr)CCCCC1 | 10 | I |
| C[C@H]1C[C@H]1N1C(CCl)=[NH+]C2=CC=C(Br)C=C12 | 10 | I |
| BrC1CC(C1)OC1=CC=CC2=CC=C[NH+]=C12 | 10 | I |
| FC1=CC=C(C=C1)[C@H](Br)CC1=CC=[NH+]C=C1 | 10 | I |
| Br[C@@H](CC1=CC=[NH+]C=C1)C1=C(Br)C=CS1 | 10 | I |
| C[C@H]1C[NH+](CC2(CBr)CCOCC2)C[C@H](C)S1 | 10 | I |
| BrCC1(C[NH+]2CCCC3=CC=CC=C23)CCOCC1 | 10 | I |
| BrCC1(CN2CCC[NH+]3CCC[C@H]3C2)CCC1 | 10 | I |
| BrCC1(C[NH+]2CCC3(CCCC3)CC2)CCC1 | 10 | I |
| BrC1=CSC=C1C(=O)CC1=CC=CC=[NH+]1 | 10 | I |
| ClC1=CC=C(C[NH+]2CC[C@H](CBr)C2)S1 | 10 | I |
| BrC[C@H]1CC[NH+](CC2=CC(Br)=CS2)C1 | 10 | I |
| CC1=CC=C(C[NH+]2CCO[C@@H](CBr)C2)C=C1 | 10 | I |
| C[C@H]1C[NH+](CC2=CC=C(I)C=C2)CCCO1 | 10 | I |
| C[C@@H]1CSCC[NH+]1CC1=CC=C(I)C=C1 | 10 | I |
| C[C@H]1CC[C@@H](C)[NH+](CC2=C(Br)C=CS2)C1 | 10 | I |
| BrC1=CSC(C[NH+]2C[C@@H]3CC[C@H](C2)O3)=C1 | 10 | I |
| FC1=C(F)C(F)=C([C@@H](Cl)CC2=CC=CC=[NH+]2)C(F)=C1F | 10 | I |
| C[NH+](C)[C@@H]1CCCN(CC2=CC=C(I)C=C2)C1 | 10 | I |
| FC1=CC([C@H](Cl)CC2=CC=[NH+]C=C2)=C(Br)C=C1 | 10 | I |
| Br[C@H](CC1=[NH+]C2=CC=CC=C2C=C1)C1CCOCC1 | 10 | I |
| Br[C@@H](CC1=[NH+]C2=CC=CC=C2C=C1)[C@H]1CCCCO1 | 10 | I |
| FC1=CC=CC(F)=C1[C@H](Br)CC1=CC=CC=[NH+]1 | 10 | I |
| FC1=CC(=CC(F)=C1F)[C@@H](Br)CC1=CC=[NH+]C=C1 | 10 | I |
| CC1=CC=C(C[C@@H](Br)C2=CC=C3OCCC3=C2)[NH+]=C1 | 10 | I |
| C[C@@H]1C[C@@H](C)CC(C1)C(=O)C1(CCCC1)[NH+]1CCCC1 | 10 | I |
| CN1[C@H]2CC[C@@H]1C[NH+](CC1(CBr)CCCCC1)CC2 | 10 | I |
| BrC1=CC=C(C[NH+]2CCC[C@@H]2[C@@H]2CCCC2=O)C=C1 | 10 | I |
| C[C@@H]1C[C@@H](CC[NH+]1C)N(C)CC1=CC(C)=C(Br)S1 | 10 | I |
| CC1=CC=C([C@H](Br)CC2=CC=CC=[NH+]2)C(F)=C1 | 10 | I |
| CSC1=C[NH+]=CC(I)=C1OC1CC1 | 10 | I |
| CN(C)C1=C(Br)C=[NH+]C=C1Br | 10 | I |
| IC1=CN2C=C([NH+]=C2C=C1)C1CC1 | 10 | I |
| C[C@@]12CC[C@H]3[C@H](CC[C@]4(C)CC(=O)CC[C@@]34C)[C@@H]1CCC2=O | 10 | I |
| COC1=CC(Br)=C(OC)C=C1Br | 10 | I |
| CCN1C(=S)S\C(=C\C2=CC=CC3=C2C=CC=C3)C1=O | 10 | I |
| C[C@@]12CC(=O)[C@H]3[C@@H](CC[C@H]4C[C@H](F)CC[C@@]34C)[C@H]1CCC2=O | 10 | I |
| CN1C=C(Br)C=C1C(=O)C(Cl)(Cl)Cl | 10 | I |
| O=S1(=O)CC[C@]11CC2C3=CC=CC=C3C1C1=C2C=CC=C1 | 10 | I |
| BrC1=C(Br)C2=C(CCO2)C2=C1CCC2=O | 10 | I |
| CC1=CC=C(C=C1)[C@@H]1C[C@@H]1C1=NN=C(Br)S1 | 10 | I |
| CC1=CC=C(C=C1)[C@H]1C[C@@H]1C1=NN=C(Br)S1 | 10 | I |
| CC1=CC=C(C=C1)[C@@H]1C[C@H]1C1=NN=C(Br)S1 | 10 | I |
| FC1=C(F)C(F)=C(N2C=C(Br)C=N2)C(F)=C1F | 10 | I |
| CC1=C2[C@@H](S[C@@H]1Br)SC([C@@H]1C=CN=N1)=C2C | 10 | I |
| CC1=C(I)[C@@H]2CC[C@H]1CC21OCCO1 | 10 | I |
| Cl[C@@H]([C@H]1CCS(=O)(=O)C1)C1=CC(Br)=CC=C1 | 10 | I |
| CC1=CC(=CC(C)=C1)[C@@H](Br)[C@H]1CCS(=O)(=O)C1 | 10 | I |
| ClC1=C(Cl)C=C(C=C1)[C@@H](Br)[C@@H]1COCCO1 | 10 | I |
| C1C[C@@H]2[C@@H](C3CCCCC3)N(CCN2C1)[C@@H]1CCSC1 | 10 | I |
| Br[C@H]([C@@H]1CCCOC1)C1=CC2=C(COC2)C=C1 | 10 | I |
| Br[C@@H]([C@H]1CCCOC1)C1=CC2=C(COC2)C=C1 | 10 | I |
| FC1=CC(Br)=C(F)C=C1[C@@H](Cl)[C@H]1COCCO1 | 10 | I |
| BrC1=CN(CC2=CC3=C(CCC3)C=C2)C(=O)C=C1 | 10 | I |
| C[C@H]1CC[C@@H](C[C@@H]1C)N1C=C(I)C=N1 | 10 | I |
| Cl[C@@H]([C@@H]1CCS(=O)(=O)C1)C1=CC=C(Br)S1 | 10 | I |
| BrC1=CC=C(C[C@]2(CCCCC2=O)C#N)S1 | 10 | I |
| IC1=CC=C(C[C@@]2(CCCC2=O)C#N)C=C1 | 10 | I |
| CC(C)C1=NC(C)=C(I)C(Cl)=N1 | 10 | I |
| FC1=C(Br)C=C2N=C(CCl)N(C3CC3)C2=C1 | 10 | I |
| CCCC1=C(I)C(Cl)=NC(C)=N1 | 10 | I |
| CC[C@@]12CC(=C)[C@H]3[C@@H](CCC4=CC(=O)CC[C@H]34)[C@H]1CCC2=O | 10 | I |
| FC(F)CN1N=CC2=C1C=C(I)C=C2 | 10 | I |
| FC1=CC=C(CN2C=CC(I)=N2)C=C1 | 10 | I |
| CC1=C(Br)SC(=C1)[C@H](Cl)[C@@H]1COCCO1 | 10 | I |
| ClCC1=NC(Cl)=C(I)C(Cl)=N1 | 10 | I |
| CC1=C(Br)C(Cl)=NC(=N1)[C@@H]1CCCCS1 | 10 | I |
| CCC1=C(Br)C(Cl)=NC(=N1)[C@@H]1CCCS1 | 10 | I |
| FC1=C(N=CC(Cl)=C1)N1CC[C@@H](CBr)C1 | 10 | I |
| IC1=CC=C(CN2CCC[C@H]2C#N)C=C1 | 10 | I |
| C[C@@H]1CN(CC2=CC=C(I)C=C2)C[C@@H](C)O1 | 10 | I |
| Br[C@H]1C[C@H](OC2CCOCC2)C11CCCCC1 | 10 | I |
| O=C1C=CC2=CC=CC=C2N1C[C@@H]1CSC2=C1C=CC=C2 | 10 | I |
| C[C@@H]1CN(CC2=CC(F)=CC(Br)=C2)CCN1C | 10 | I |
| CN1C(C[C@@H]2CC[C@@H](Br)C2)=NC2=CC=CC=C12 | 10 | I |
| IC1=NN(C[C@@H]2CC3=CC=CC=C23)C=C1 | 10 | I |
| Br[C@@H]1CCC[C@H](C1)[C@@H]1CCO[C@]2(CCOC2)C1 | 10 | I |
| FC(F)(F)C1=CCC2=NN=C(I)C2=C1 | 10 | I |
| ClC1=C(I)C=C(OC2CC2)C=N1 | 10 | I |
| ClC1=C(Br)C=C(S1)S(Cl)(=O)=O | 10 | I |
| FC1=C(CN2C=CC(=O)C=C2Cl)C=CC(Br)=C1 | 10 | I |
| ClC[C@]1(CCCC2=CC=CC=C12)[C@H]1CCS(=O)(=O)C1 | 10 | I |
| COC1=CC2=CC(I)=CN=C2C=C1F | 10 | I |
| CC1(C)OC(=O)O[C@@]1(Br)CBr | 10 | I |
| CCN1C(=O)\C(=C\C2=CC=C(F)C=C2)N(C)C1=S | 10 | I |
| FC1=CC=C(C=C1)N1C(=O)[C@H]2CSCN2C1=S | 10 | I |
| CN1C(=S)S\C(=C/C2=C(C)N(C3CC3)C(C)=C2)C1=O | 10 | I |
| CS(=O)(=O)OC1=C(F)C(F)=C(F)C(F)=C1F | 10 | I |
| CC(C)[C@@H](Br)C(=O)N1CCO[C@@H](C)C1 | 10 | I |
| CN1C(=S)S\C(C1=O)=C1\C=CC=CN1CC=C | 10 | I |
| CN1C(CCl)=NC2=C1C(Br)=CN=C2 | 10 | I |
| CC(C)[C@H](Br)C(=O)N1CCOC[C@H]1C | 10 | I |
| CN(C)\C=C1/SC(=S)N(C1=O)C1=CC=C(F)C=C1 | 10 | I |
| CN1C(=O)CC2=CC(=CC=C12)[C@H](Cl)[C@H]1CCCO1 | 10 | I |
| CCC1=C(C)SC=C1C(=O)N1C[C@H](C)O[C@H](C)C1 | 10 | I |
| FC1=C(Br)C=C2C(=O)C(=O)N(CC#C)C2=C1 | 10 | I |
| BrC1=C(CN2CCOC2=O)SC=C1 | 10 | I |
| C[C@@H](C#N)N(C)C(=O)C1=CC=C(Br)C=C1 | 10 | I |
| C[C@@H](C#N)N(C)C(=O)C1=C(F)C(F)=C(F)C(F)=C1F | 10 | I |
| C[C@@H]1C[C@H](SC2=NC3=CC=CC=C3CS2)C(=O)O1 | 10 | I |
| CC[C@@H](Br)C(=O)N1CCOCC1(C)C | 10 | I |
| C[C@H]1[C@@H]2CC[C@]3(C)C=C(Cl)C(=O)C(C)=C3[C@@H]2OC1=O | 10 | I |
| BrC[C@@H](C#N)C1=NC=NC2=CC=CC=C12 | 10 | I |
| BrC[C@H](C#N)C1=NC=NC2=CC=CC=C12 | 10 | I |
| O=C(CN1CCC[C@@]2(CCC=CC2)C1)N1CCCC1 | 10 | I |
| COC1=CC=C(Br)C=C1S(C)(=O)=O | 10 | I |
| CN(C)\C=C1\SC(=S)N(C1=O)C1=CC=C(F)C=C1 | 10 | I |
| COCC1=C(I)C(Cl)=NC(C)=N1 | 10 | I |
| BrC(=C)CN1C=NC=C(Br)C1=O | 10 | I |
| CN(C1CC1)C1=C(I)C=NC=N1 | 10 | I |
| Cl[C@@H]1CCN(C1)C1=NC=NC=C1Br | 10 | I |
| ClC1=C(C=C(Br)C=C1)N1CC(=O)CC1=O | 10 | I |
| IC1=CN(N=C1)[C@@H]1CCCC(=O)C1 | 10 | I |
| BrC1=CC=C(CN2C=CN=C2C#N)S1 | 10 | I |
| BrC1=CSC(CN2C=CN=C2C#N)=C1 | 10 | I |
| CC1=C(C)N=C(CN2C=C(Br)C=N2)S1 | 10 | I |
| BrCC1CCN(CC1)C(=O)[C@@H]1CCCCO1 | 10 | I |
| C[C@H]1CO[C@H](CCl)CN1C1=NC(C)=C(C)S1 | 10 | I |
| C[C@@H]1C[C@@H]1C(=O)N1CCO[C@H](CBr)C1 | 10 | I |
| C[C@H](N1CCCSCC1)C(=O)N1CCC(C)CC1 | 10 | I |
| C[C@H]1C[C@@H](C)CC(C1)C(=O)[C@@H]1CN2CCC[C@@H]2CO1 | 10 | I |
| C[C@H]1CN(CC2=CC3=CC(F)=CC=C3O2)[C@H](C)CO1 | 10 | I |
| C[C@H]1CN(CC2=CC3=C(C=CS3)N=C2)[C@H](C)CO1 | 10 | I |
| FC(F)(F)C1=CC(Cl)=C(C=C1)N1CC(=O)CC1=O | 10 | I |
| C[C@H]1OCCN(CC2=CC3=C(C=CS3)N=C2)[C@@H]1C | 10 | I |
| O=C([C@H]1CCOC1)[C@H]1CCOC2(CCSCC2)C1 | 10 | I |
| C[C@H]1CCC(=O)[C@H](CN2C[C@@H]3CCCN3C[C@@H]2C)C1 | 10 | I |
| FC1=NC(F)=C(F)C(SC2=NC=CC=N2)=C1F | 10 | I |
| ClC1=CC2=C(I)N=N[C@H]2C=N1 | 10 | I |
| CN1CCO[C@@H]2CN(CC3=C(C)C=CS3)C[C@@H]2C1 | 10 | I |
| Cl[C@H]1CC(=O)N(C1=O)C1=CC=C(Br)C=C1 | 10 | I |
| CC1=CC=C2C=C(C=CC2=N1)[C@@H]1OC[C@H](CCl)O1 | 10 | I |
| CC(=O)C1=CC(Br)=NN=C1Br | 10 | I |
| O=C1N(C(=S)S\C1=C/C1=CN=CC=C1)C1=CC=C2OCOC2=C1 | 10 | I |
| COC1=CC=CC=C1N1C(=O)[C@@H]2[C@H]([C@@H]3C(=O)C[C@H]2C2=C3C=CC=C2)C1=O | 10 | I |
| CN1C=C2N(C)C(=O)N(C)C(=O)C2=C1C1=CC=C(Br)C=C1 | 10 | I |
| C[C@@H]1C[C@H]2OC(=O)C(=C)[C@H]2[C@@H](OC(=O)C(C)=C)[C@@]2(C)[C@H]1C=CC2=O | 10 | I |
| FC1=CC=CC=C1N1[C@@H]2CS(=O)(=O)C[C@H]2S\C1=N/C(=O)CCl | 10 | I |
| ClC1=CC(Cl)=C2OC(=O)C(=CC2=C1)C(=O)N1CCOCC1 | 10 | I |
| CCN([C@H]1CCS(=O)(=O)C1)C(=O)C1=CC=C(Br)O1 | 10 | I |
| CC(C)C(=O)N1CCN(CC1)C(=O)C1=CC=C(Br)O1 | 10 | I |
| CCN1CCN(CC1)S(=O)(=O)C1=CC(Br)=CN=C1 | 10 | I |
| C[C@@H](C(=O)N1C[C@@H](C)C[C@H](C)C1)S(=O)(=O)C1=NC(C)=CS1 | 10 | I |
| CN1C=C(C(=O)CC2=CC=CC(Br)=C2)C(=O)N(C)C1=O | 10 | I |
| BrC1=CC=CC2=C1N(CC(=O)C1=CC=CO1)C(=O)C2=O | 10 | I |
| FC1=CC(C(=O)C2CCN(CC2)C(=O)C2=CN=CC=N2)=C(F)C=C1 | 10 | I |
| FC1=CC(Br)=C(C=C1)S(=O)(=O)N1CCN2C=CN=C2C1 | 10 | I |
| CC1=NN=C(CN2CCO[C@@H](C2)C2=CC=C(Cl)C(Cl)=C2)O1 | 10 | I |
| CCC1=CC=C(C=C1)S(=O)(=O)N1CC[C@]2(C1)CCCN(C)C2=O | 10 | I |
| CC1=CC(C(=O)N2CCCS2(=O)=O)=C(C)N1C1=CC=C(F)C=C1 | 10 | I |
| CN1C(=O)C2=CC=C(C=C2C1=O)N1CCC[C@@H](Br)C1=O | 10 | I |
| CN1C=C(C=N1)[C@H]1CCCN1C(=O)C1=C(C)N=C2C=C(F)C=CC2=C1 | 10 | I |
| ClC1=CC=CC=C1[C@H]1CN(CCO1)C(=O)CN1CSCC1=O | 10 | I |
| O=C1CCCN1CC1=CC(=CC=C1)N1C(=O)[C@H]2[C@@H]3C[C@@H](C=C3)[C@H]2C1=O | 10 | I |
| CN1N=C(C)C([C@H]2CCCN2C(=O)C2=CSC(=N2)C2CC2)=C1C | 10 | I |
| C[C@@H]1C[C@@H](C)CN(C1)S(=O)(=O)C1=CC=C2N(C)C(=O)CCC2=C1 | 10 | I |
| C[C@@H](C1CCN(CC1)C(=O)C1=CC=C(Cl)C=N1)N1CCOCC1 | 10 | I |
| C[C@H]1[C@@H](C)[S@@](=O)CCN1C(=O)C1=NN(C(=C1)C1CC1)C(C)(C)C | 10 | I |
| C[C@@H]1CN(C[C@@H]1N1CCOCC1)C(=O)C1=CC=C(Br)O1 | 10 | I |
| C[C@@H]1CN(C[C@H]1N1CCOCC1)C(=O)C1=CC=C(Br)O1 | 10 | I |
| C[C@@H]1[C@@H](C)S(=O)(=O)CCN1S(=O)(=O)C1=CC=C(Cl)S1 | 10 | I |
| C[C@@H]1CN(C(=O)CCN2C=NC3=C(C=CS3)C2=O)C2=C1C=CC=C2 | 10 | I |
| C[C@H]1C[C@H](N2CCN(CC2)C(=O)C23CC4CC(CC(C4)C2)C3)C(=O)O1 | 10 | I |
| CC[C@H]1CN(C[C@H]1N1CCOCC1)C(=O)C1=COC2=C1C=CC=C2 | 10 | I |
| C[C@@H](C(=O)N1CC[S@](=O)C(C)(C)C1)C1=CC=C2OCCOC2=C1 | 10 | I |
| CN1N=CC(CCC(=O)N2C[C@@H]3CCCN3CC3=C2C=CC=C3)=C1C | 10 | I |
| CCN([C@H]1CCS(=O)(=O)C1)C(=O)[C@@H]1COC2=CC=C(F)C=C2C1 | 10 | I |
| CN1CCC[C@]2(CCN(CC3=CN=CN3C3CCCCC3)C2)C1=O | 10 | I |
| CN1CCO[C@H](CN2C(=O)C(=O)C3=CC=C(Br)C=C23)C1 | 10 | I |
| C[C@H](Cl)C1=NC2=C(N=CC=C2C)N1[C@@]1(C)CCS(=O)(=O)C1 | 10 | I |
| CC1=CN2C(S1)=NC=C(C(=O)N1CCC[C@@]3(CCC=CC3)C1)C2=O | 10 | I |
| FC1=CC2=C(OC(CN3CCC[C@H](C3)C3=NN=C4CCCN34)=C2)C=C1 | 10 | I |
| CN1CCC2(CN(CC3=CN=CN3C3CCCCC3)C(=O)C2)CC1 | 10 | I |
| CN1C=C(CN2CC[C@H]3C[C@H](O[C@@H]3C2)C2=NC(C)=C(C)S2)C=N1 | 10 | I |
| C(N1CCC[C@@]2(C1)COCCN(C2)C1=CC=CN=C1)C1=CN=CC=C1 | 10 | I |
| FC1=CN=C(N=C1)N1CCC[C@]2(CCN(CC3=NC=CS3)C2)C1 | 10 | I |
| CC1=CC(C(=O)N2CCC3=C(CC2)N(CC2CC2)C(=O)C=C3)=C(C)O1 | 10 | I |
| FC(F)(F)CC(=O)N1CC[C@]2(C1)CCCN(C2)C1=NN=CS1 | 10 | I |
| FC1=C(F)C=C(C=C1)N1C[C@@]2(CCN(C2)C2=NN=CS2)CC1=O | 10 | I |
| CCN1CCO[C@@H]2CN(CC[C@@H]2C1)S(=O)(=O)C1=CC=CS1 | 10 | I |
| C[C@H]1CS(=O)(=O)CCN1C(=O)C1=C(C)N=C(S1)C1CCCC1 | 10 | I |
| CN1C=CC=C1[C@H]1COCCN1C(=O)C1=COC(Br)=C1 | 10 | I |
| CC1=CC(OC2CCN(CC2)C(=O)[C@H]2C[C@H]3C[C@@H]2C=C3)=CC(=O)O1 | 10 | I |
| CSC1=CC(=CC=C1)N1C[C@H](CC1=O)C1=NC2=CC=CC=C2N1C | 10 | I |
| BrC1=CC=C(C=C1)N1C(=O)C=C(N2CCCCC2)C1=O | 10 | I |
| COC1=CC=C(C=C1)N1C(=S)S\C(=C/C2=CC=C(C)O2)C1=O | 10 | I |
| IC1=CC=C(C=C1)N1CN=C2SCC(=O)N2C1 | 10 | I |
| CC(=O)O[C@@H]1C[C@@]2(C)[C@H]3CC[C@]4(C)[C@H](CCC4=O)[C@@H]3CCC2=CC1=O | 10 | I |
| CC(=O)O[C@H]1C[C@@H]2[C@@H]3CCC(=O)[C@]3(C)CC[C@@H]2[C@@]2(C)CCC(=O)C=C12 | 10 | I |
| BrC1=CC=C(C2=CC=CC=C12)S(=O)(=O)N1C=CN=C1 | 10 | I |
| CC1=CC(=CC=C1F)S(=O)(=O)C1=CC2=CC(F)=CC(F)=C2OC1=O | 10 | I |
| CN(CC1=NC2=CC=CC=C2S1)C(=O)C1=CN(C)C2=C1C=CC=C2 | 10 | I |
| CCC1=CC=C(C=C1)S(=O)(=O)C1=CN(C)C2=CC=CC=C2C1=O | 10 | I |
| BrC1=CC=C(C=C1)N1C(=O)C[C@H](N2CCCCC2)C1=O | 10 | I |
| C[C@H]1CCCN(CN2C(=O)C(=O)C3=C(Br)C=CC=C23)C1 | 10 | I |
| BrC1=CC=C(Br)C(=C1)S(=O)(=O)N1C=CC=N1 | 10 | I |
| C[C@H]1CN(C[C@@H]2[C@H]3C[C@H]4C(=C)CCC[C@]4(C)C[C@H]3OC2=O)C[C@H](C)O1 | 10 | I |
| O=C1[C@H]2[C@H](C3C4=CC=CC=C4C2C2=CC=CC=C32)C(=O)N1C1=CC=NC=C1 | 10 | I |
| C[C@@H]1CN(C[C@@H]2[C@H]3C=C4[C@@H](C)CCC[C@@]4(C)C[C@H]3OC2=O)C[C@@H](C)O1 | 10 | I |
| CS(=O)(=O)N1CCN2C=CC=C2[C@H]1C1=CC=C(Br)C=C1 | 10 | I |
| CC1=NOC(=N1)[C@@H]1CCCN(CC2=CC=CC=C2Br)C1 | 10 | I |
| CC1=NN(CC2=CN(N=C2)C2=CC=CC=C2)C(C)=C1Br | 10 | I |
| ClC1=CC2=C(OCCCO2)C=C1[C@H](Br)[C@H]1COCCO1 | 10 | I |
| C[C@@H]1CN(C(=O)CN2C=C(C=CC2=O)C(F)(F)F)C2=C1C=CC=C2 | 10 | I |
| CN1C=C(C=CC1=O)C(=O)N1CCC(CC2=CC=C(F)C=C2)CC1 | 10 | I |
| CS(=O)(=O)C1=CC=C(C=C1)C1=NN=C(I)S1 | 10 | I |
| BrC1=CC2=C(C=C1)N(CN1CCCSCC1)C(=O)C2=O | 10 | I |
| BrC1=NN(CCN2CCCCC2)C(Br)=N1 | 10 | I |
| FC1=C(F)C(F)=C(OS(=O)(=O)C2=NC=CS2)C(F)=C1F | 10 | I |
| O=C1OCC[C@H]1N1CCN(CC2=CC=CC=C2)C2(CCCCC2)C1 | 10 | I |
| ClCC1=CC(=CS1)S(=O)(=O)N1CCCN2CCC[C@@H]2C1 | 10 | I |
| C[C@H]1CN(C[C@H](C)S1)S(=O)(=O)N1CCC(CCl)CC1 | 10 | I |
| CCC1=NN(C)C(CN2C(=O)SC(C)=C2C)=C1Br | 10 | I |
| COCC1=C(Br)C(Cl)=NC(=N1)[C@@H]1C[C@H]2CC[C@@H]1O2 | 10 | I |
| CC1=C(Br)C(=O)N(CCC2=CC=C3OCCC3=C2)C=N1 | 10 | I |
| CC1=CC=C(C=C1)S(=O)(=O)N1C[C@@H](Br)C(=O)C(C)(C)C1 | 10 | I |
| CC1=NC2=C(SC=C2)C(=N1)N1CCN(CC1)C1CCSCC1 | 10 | I |
| CN1[C@H]2CC[C@@H]1CN(CC2)S(=O)(=O)C1=CSC(CCl)=C1 | 10 | I |
| IC1=CC2=C(OCC(=O)N2C[C@H]2CCCO2)C=C1 | 10 | I |
| CN1N=C(C)C(Br)=C1C[C@]1(CCCC(C)(C)C1=O)C#N | 10 | I |
| CC[C@@H]1N(CC2=CC=C(Cl)S2)C(=O)[C@H]2CCCCN2C1=O | 10 | I |
| C[C@H]1N(C(=O)[C@@H]2CCCCN2C1=O)C1=CC=C(Br)C(F)=C1 | 10 | I |
| C[C@@H]1N(C(=O)[C@@H]2CCCCN2C1=O)C1=CC=C(Br)C(F)=C1 | 10 | I |
| CC1=CC(Br)=CC(C)=C1N1CCC(=O)N2CCC[C@@H]2C1=O | 10 | I |
| FC1=CC(Br)=CC(F)=C1N1CCC(=O)N2CCC[C@H]2C1=O | 10 | I |
| CS(=O)(=O)[C@@H]1CCC[C@H](C1)OC1=CC(=CN=C1)C#CCCl | 10 | I |
| CC[C@@H]1CN(C(=O)C1)S(=O)(=O)C1=CC=C(Br)S1 | 10 | I |
| CN1N=C(C)C(Cl)=C1CC(=O)C1=CC(Br)=CN=C1 | 10 | I |
| BrC1=CN=CC(=C1)C(=O)[C@@H]1CCOC2(CCOCC2)C1 | 10 | I |
| C[C@@H]1CN(CCO1)S(=O)(=O)C1=CC=C(F)C(Br)=C1 | 10 | I |
| FC1=C(Br)C=CC(CN2C=CN=C2C2=CN=CC=N2)=C1 | 10 | I |
| C[C@@H](Cl)C1=NC2=CC(Cl)=C(F)C=C2N1[C@H](C)C(=O)N(C)C | 10 | I |
| CC1=C(C=NO1)C(=O)N1CCS[C@H]1C1=CC=C(Cl)C(Cl)=C1 | 10 | I |
| C[C@@]12CC[C@H]3[C@H]([C@H]1CCC2=O)C(=O)C[C@@H]1CCCC[C@@]31C | 10 | I |
| CC1=C(Br)C(C)=C(C#N)C(Br)=N1 | 10 | I |
| FC1=CC=C(C=C1)[C@H]1OC(=O)C2=CC=CC=C2S1 | 10 | I |
| C1=CC=C2N=C3C4=CC=CC5=CC=C[C@H]([C@@H]45)C3=NC2=C1 | 10 | I |
| CSC1=NC(C)=NC2=C1C1=C(C[C@H](C)CC1)S2 | 10 | I |
| C1OC2=C(O1)C=C(C=C2)C1SCSCS1 | 10 | I |
| C[C@@]12CC[C@H]3[C@H](CC(=O)[C@H]4CCCC[C@@]34C)[C@H]1CCC2=O | 10 | I |
| C[C@H]1CCC2=CC=CC3=C2N1C1=C3CCSC1=O | 10 | I |
| CC1=CC(C)=C2C[C@@H]3C=CC=C[C@H]3S(=O)(=O)C2=C1C | 10 | I |
| CC1(C)OC(=O)C(=C1)C1=CC=C(Br)C=C1 | 10 | I |
| CC1=C(C)C2=C(O1)C=C1OC(=S)C3=C(CCC3)C1=C2 | 10 | I |
| FC(F)(F)[C@@]1(F)OC2=CC=CC=C2C(F)(F)O1 | 10 | I |
| FC(F)(F)[C@]1(F)OC2=CC=CC=C2C(F)(F)O1 | 10 | I |
| BrC1=CC=C(C=C1)N1N=CC2=CC=CC=C12 | 10 | I |
| BrC1=CC2=C(C=C1)C=C1CCCOC1=N2 | 10 | I |
| C[C@H]1[C@H]2C[C@H]([C@H](Br)C22OCCO2)C1(C)C | 10 | I |
| CC1=CN2C(Br)=CN=C2C(Br)=C1 | 10 | I |
| ClC1=NSN=C1C1=CC(Br)=CC=C1 | 10 | I |
| Cl[C@H]1[C@]23CCCCC[C@@]2(CS1(=O)=O)C=CC=C3 | 10 | I |
| Cl[C@H]1CCCC2=C(Cl)C=C3OCCCOC3=C12 | 10 | I |
| CC1=NN(C(S)=C1)C1=CC=C(Cl)C(Cl)=C1 | 10 | I |
| C[C@]1(CC(=O)C=CO1)C1=CC(F)=C(Cl)C=C1Cl | 10 | I |
| FC1=C(Br)C=C(C=C1)[C@@H]1OCCC[C@H]1C#N | 10 | I |
| FC1=C(C=CC(Br)=C1)[C@@H]1OCCC[C@H]1C#N | 10 | I |
| FC1=C(C=CC(Br)=C1)[C@@H]1OCCC[C@@H]1C#N | 10 | I |
| FC1=CC(=CC(F)=C1F)C1=NN=C(Br)S1 | 10 | I |
| C[C@H]1[C@H]2O[C@]22CC[C@H](C2)C(C)(C)C(=O)[C@@H]2CCC[C@H]12 | 10 | I |
| ClC1=NC(Cl)=C(Br)C(Cl)=N1 | 10 | I |
| C[C@]12CC[C@H]3[C@@H](CC[C@H]4C[C@@H]5O[C@@H]5C[C@]34C)[C@@H]1CCC2=O | 10 | I |
| CN1N=CC2=C1C[C@@H](C[C@H]2Cl)C1=CC=C(F)C=C1 | 10 | I |
| C[C@H]1C[C@@H](C)CC(C1)N1C=C(Br)C=N1 | 10 | I |
| ClC1=C2OC(C=O)=CC2=CC(Br)=C1 | 10 | I |
| ClC1=CC(Br)=C2OC(C=O)=CC2=C1 | 10 | I |
| C[C@H]1C[C@@H](C)CC(C1)N1N=C(C)C(Br)=C1C | 10 | I |
| CN1C=C(C=N1)C1=CC=C(I)C=C1 | 10 | I |
| CC1=CC(F)=C(Br)C2=C1N=C(C=O)C=C2 | 10 | I |
| C[C@H]1CCC[C@@H](C1)C1=NC=C(Br)C(Cl)=N1 | 10 | I |
| ClC1=NC(=NC=C1Br)[C@H]1CCCCS1 | 10 | I |
| ClC1=CC(I)=CC2=NC=CN12 | 10 | I |
| CC(=O)N1C=C(Br)C2=C1C=CC=C2Cl | 10 | I |
| Cl[C@H]1CCC[C@H]1[C@@H]1CCOC2(CCOCC2)C1 | 10 | I |
| O=C1CCCC[C@H](C1)[C@H]1CCOC2(CCSCC2)C1 | 10 | I |
| CC1(C)CC(=O)C=C(C1)[C@H]1CCOC2(CCSCC2)C1 | 10 | I |
| Cl[C@@H]1CCCC[C@H](C1)[C@@H]1CCO[C@]2(CCOC2)C1 | 10 | I |
| C[C@@H]1[C@@H](Cl)CC[C@@H]1[C@@H]1CCO[C@]2(CCOC2)C1 | 10 | I |
| CC1(C)CCN(CC1)C1=C(F)C(F)=NC(F)=C1F | 10 | I |
| FC1=C(Br)C=C2C(Cl)=NC=NC2=C1 | 10 | I |
| F[C@H]1OC2=C(OC1(F)F)C=C(C=C2)C(Cl)Cl | 10 | I |
| F[C@H]1OC2=C(C=CC=C2OC1(F)F)C(Cl)Cl | 10 | I |
| FC1=CC=C(C=C1)N1C=CC2=C1C=NC=C2Br | 10 | I |
| BrC1=CC=C(C=C1)C1=CSN=N1 | 10 | I |
| BrC1=CSC(=C1)C1OCCO1 | 10 | I |
| BrC1=CC2=C(CCO[C@@H]2C#N)C=C1 | 10 | I |
| CC1(C)CCC(C2=CC=C(Cl)C(Cl)=C2)=[N+]1[O-] | 10 | I |
| C[C@H]1OCC[C@]11S[C@@H]2CCO[C@]2(C)S1 | 10 | I |
| BrC1=CN=C(S1)N1C=CC=C1 | 10 | I |
| C[C@@H]1[C@H]2C[C@H]3C(=C)CCC[C@]3(C)C[C@H]2OC1=O | 10 | I |
| C[C@@H]1CC[C@@H]2[C@H](C(=O)[C@H](C)CCC1=O)C2(C)C | 10 | I |
| C[C@@H]1CC[C@H]2[C@H](C(=O)[C@H](C)CCC1=O)C2(C)C | 10 | I |
| BrC1=CC=C(C=C(C#N)C#N)C=C1 | 10 | I |
| CN1C=C(C=O)C2=C1C=CC(Br)=C2 | 10 | I |
| CC1=CC=C(\C=C2\SC(S)=NC2=O)C=C1 | 10 | I |
| C[C@H]1SC2=C(SC(=O)S2)S[C@@H](C)C1=O | 10 | I |
| CN1CCN(CC1)C1C2CC3CC(C2)CC1C3 | 10 | I |
| CC1=C(C)N2C=C(Br)C=C(C)C2=N1 | 10 | I |
| ClCC1=CC2=C(OCCCO2)C=C1Cl | 10 | I |
| C[C@@H]1CCC[C@]2(C)C[C@H]3OC(=O)C(=C)[C@H]3C=C12 | 10 | I |
| CC[C@@H]1C=CC(C)=C2N=C3SC(C)=NC3=C12 | 10 | I |
| CC1=CC=C2OCCN3C(=S)C=C(C)C1=C23 | 10 | I |
| CN1C=CN=C1C1=CC=C(Br)C=C1 | 10 | I |
| CC1(C)[C@@H]2CC[C@](C2)(C=C)[C@@]2(CCCO2)C1=O | 10 | I |
| BrC1=CN=C(S1)C1=COC=C1 | 10 | I |
| IC1=CN(N=C1)C1CCCC1 | 10 | I |
| CN1C=C(C=N1)C1=CC=C(Br)C=C1 | 10 | I |
| ClC1=C2OCC(=O)C2=C(Br)C=C1 | 10 | I |
| Cl[C@H]1CCO[C@H](C1)C1=CC2=C(OCC2)C=C1 | 10 | I |
| CC(C)[C@H]1C[C@@]2(CCO1)C[C@H](Cl)CCO2 | 10 | I |
| FC1=C(F)C(F)=C(N2C=CN=C2)C(F)=C1F | 10 | I |
| BrC1=CN=CC(=C1)C1=CC=NC=C1 | 10 | I |
| CC1(C)O[C@@H]2CCC=C(Br)[C@@H]2O1 | 10 | I |
| FC1=CC(=CC(F)=C1F)[C@H]1OCCC[C@H]1C#N | 10 | I |
| C[C@@H]1C[C@H](Cl)C2=C1C=C1OCCCOC1=C2 | 10 | I |
| C[C@@H]1C[C@@H](Cl)C2=C1C=C1OCCCOC1=C2 | 10 | I |
| FC(F)(F)[C@H]1CCC[C@]2(C1)CC(=O)CCO2 | 10 | I |
| C[C@@H]1OC2=C(F)C=C(Br)C=C2C1=O | 10 | I |
| BrC1=CC2=C(C=C1)C=CC(=O)N2CC#C | 10 | I |
| FC1=C2C=CC(C=O)=NC2=C(Br)C=C1 | 10 | I |
| ClC1=CSC(=C1)[C@H]1OCCC[C@H]1C#N | 10 | I |
| O=C1CCC[C@H]1[C@H]1CCO[C@]2(CCSC2)C1 | 10 | I |
| Cl[C@@H]1CCC(=C1)[C@H]1CCO[C@]2(CCOC2)C1 | 10 | I |
| CC1=CC(Cl)=NC(=N1)[C@H]1CSCCS1 | 10 | I |
| CC1=CC(Cl)=NC(=N1)[C@H]1CCCCS1 | 10 | I |
| ClC1=NC=CC2=C1N=C(Br)S2 | 10 | I |
| O=C1CCC[C@H](C1)[C@H]1CCO[C@]2(CCSC2)C1 | 10 | I |
| FC1=CC(Cl)=C(C=C1)S(Cl)(=O)=O | 10 | I |
| ClCC1=NC(=CC(Br)=C1)C#N | 10 | I |
| FC(F)(F)C1=CC2=C(N=C1)C(Cl)=NC=C2 | 10 | I |
| IC1=CC2=C(OC=N2)C=C1 | 10 | I |
| BrC1=CN=CC(=C1)N1C[C@H]2CC[C@@H]1C2 | 10 | I |
| Cl[S@](=O)C1=NC=C(Br)C=C1 | 10 | I |
| C=CC[C@@]1(C[C@@H]2C[C@H]1C=C2)C#N | 10 | I |
| S=C1SC2=C(S1)SC=CS2 | 10 | I |
| CC1(C)CCCC(C)(C)S1=O | 10 | I |
| CC1=CSC2=CC(C)=C(C=S)N12 | 10 | I |
| Cl[C@]12CCCC(Cl)(Cl)[C@H]1O2 | 10 | I |
| CC(C)(C)SC(S)=S | 10 | I |
| BrC1=CC(\C=C\C#N)=CS1 | 10 | I |
| FC1=C(F)C(F)=C(C=O)C=C1 | 10 | I |
| FC(F)=C(F)OC(F)(F)F | 10 | I |
| S1C=CC(=C1)C1=CSC=C1 | 10 | I |
| CC1(C)CCCC(C)(C)N1F | 10 | I |
| BrC1CCSCC1 | 10 | I |
| Br\C=C\C=C\C=O | 10 | I |
| ClCC1=CC=C(CC#N)C=C1 | 10 | I |
| ClC(=O)[C@H]1C[C@@H]2CC=C[C@@H](C2)C1 | 10 | I |
| ClC(=O)[C@H]1C[C@H]2CC=C[C@H](C2)C1 | 10 | I |
| FC(=O)C12CCC(Cl)(CC1)CC2 | 10 | I |
| O=C1C[C@H]2[C@@H]3C[C@@H](CCC3)C2=C1 | 10 | I |
| Br[C@H]1CCCC=C1 | 10 | I |
| C[C@@H](C=C)[C@@H]1C[C@@H](C)CCC1=O | 10 | I |
| FC1=C(F)C(CBr)=C(F)C=C1 | 10 | I |
| FC(F)=CCCBr | 10 | I |
| BrC1=C[C@@H]2C[C@H](C1)C=C2 | 10 | I |
| C[C@H](Br)CC(F)(F)F | 10 | I |
| FC1(F)C[C@@H]1CBr | 10 | I |
| Br[C@H]1CC[C@@H](CCC#C)C1 | 10 | I |
| C[C@]1(CCS)OCCS1 | 10 | I |
| FC1=CC(F)=C(F)C(F)=C1CCl | 10 | I |
| C\C(F)=C(\Br)C(F)(F)F | 10 | I |
| C[C@H]1CC[C@H](Br)CS1 | 10 | I |
| F\C(=C\C(F)(F)F)C(F)(F)F | 10 | I |
| Cl[C@@H]1CCN(CC2CC2)C1 | 10 | I |
| C[C@H]1CC[C@H]([C@@H]1C)N1C=CC=C1 | 10 | I |
| C[C@@H](Br)[C@H]1CCCOCC1 | 10 | I |
| C[C@@H]1C[C@@H](C)CC(CC(C)=O)C1 | 10 | I |
| FCC(F)(Cl)Br | 10 | I |
| CC1(C)C[C@@H](CCl)C(C)(C)O1 | 10 | I |
| CC1(C)CSC[C@@H](S)C1 | 10 | I |
| ClC[C@H]1C[C@@]11CCCOC1 | 10 | I |
| C[C@H]1C[C@@]2(C[C@@H]2CBr)CCO1 | 10 | I |
| BrC[C@H]1C[C@H]1C1CC1 | 10 | I |
| BrC[C@@H]1C[C@@H]1C=O | 10 | I |
| CC1(C)C[C@@H](CBr)O1 | 10 | I |
| FC(F)(F)I | 10 | I |
| FC1=CC=C(Br)C2=NC=C[C@@H]12 | 10 | I |
| FC1=C(Cl)C=NC=C1CCl | 10 | I |
| FC1=C(Cl)C2=CC=NC2=CC1 | 10 | I |
| C[C@@]1(Br)C[C@@H]1C=C | 10 | I |
| C[C@]1(Br)C[C@H]1C=C | 10 | I |
| C\C=C\C(F)(F)C(F)(F)F | 10 | I |
| ClC1=CC=C(CN2CC(=O)N3CC4=CC=CC=C4C[C@H]3C2=O)C=C1 | 10 | I |
| CN(C)C1=CC=C(\C=C2/SC(=S)N(C2=O)C2=CC=CN=C2)C=C1 | 10 | I |
| CN1CCN(CC1)S(=O)(=O)CC1=CC=C(Br)C=C1 | 10 | I |
| CN(CC1=CC=C(Cl)C(Cl)=C1)C(=O)C1=C(C)N(C)N=C1C | 10 | I |
| BrC1=CC(C(=O)CN2C=NC=N2)=C(Br)S1 | 10 | I |
| COC1=CC=C(Br)C=C1[C@H]1SCCN1S(C)(=O)=O | 10 | I |
| CCC1=CC=C(C=C1)S(=O)(=O)C1=CN(C)C2=CC(F)=C(F)C=C2C1=O | 10 | I |
| COCCN1C(=O)[C@H]2[C@@H](C3C4=CC=CC=C4C2C2=C3C=CC=C2)C1=O | 10 | I |
| CC1=N[C@@H](SCC2=NC(=O)C3=C4CCC[C@H]4SC3=N2)SC1 | 10 | I |
| BrC1=CC2=C(C=C1)C(=O)C(=O)N2CCC1=CC=NC=C1 | 10 | I |
| C[C@H]1C[C@H](SC2=NC=CN2C2=CC=C(Br)C=C2)C(=O)O1 | 10 | I |
| COC1=CC=C(Br)C=C1CN1CCS(=O)(=O)CC1 | 10 | I |
| CN(CC1=C(C)OC=C1)C(=O)C1=CC(Br)=CN=C1Cl | 10 | I |
| CCS(=O)(=O)N1CCN(CC1)C1=CC=C(Br)C=C1 | 10 | I |
| O=C1CC2(CCN(CC3=CC=CC=C3)CC2)C(=O)N1C1=CC=CC=C1 | 10 | I |
| FC1=CC=CC2=C1N=C(CCCl)N2[C@@H]1CCCS(=O)(=O)C1 | 10 | I |
| CS(=O)(=O)C[C@H](Cl)C1=CC2=C(OCCO2)C=C1Br | 10 | I |
| CCN([C@@H]1CCS(=O)(=O)C1)C(=O)C1=C(Br)C=CS1 | 10 | I |
| C[C@@H]1[C@@H](C)S(=O)(=O)CCN1C(=O)CC1=CC=C(C=C1)C(F)(F)F | 10 | I |
| CN(CC#N)C(=O)C1=C(C)N(C(C)=C1)C1=CC=CC(Br)=C1 | 10 | I |
| O=C(N(C1CC1)[C@H]1CCS(=O)(=O)C1)C1=CC2=C(CCCC2)C=C1 | 10 | I |
| CC[C@@H]1CN(C[C@@H]1N1CCOCC1)C(=O)[C@@H]1CSC2=C1C=CC=C2 | 10 | I |
| CC1=NC=C(I)C(=O)N1CC(=O)C(C)(C)C | 10 | I |
| BrC1=CC=C(C=C1)[C@@H](C#N)C(=O)[C@@H]1CCS(=O)(=O)C1 | 10 | I |
| BrCC1(CN2C(=O)C(=O)C3=CC=CC=C23)CCOCC1 | 10 | I |
| C[C@@H](N1C(CCl)=NC2=CC=C(Br)C=C12)C(=O)N(C)C | 10 | I |
| CCO[C@H]1CCCN(C1)C1=C(I)C=NC=N1 | 10 | I |
| C[C@H]1CN(C(=O)CCN2C(=O)SC(C)=C2C)C2=C(S1)C=CC=C2 | 10 | I |
| CN1CCN(CC1)S(=O)(=O)C1=CC=C(CBr)C=C1 | 10 | I |
| CC1=CC(Br)=CC2=C1N(CC1(CC#N)CC1)C(=O)C2=O | 10 | I |
| C[C@H]1CN(CCN1C)S(=O)(=O)C1=CC=C(F)C(CCl)=C1F | 10 | I |
| CN1N=C(C)C(Br)=C1CN1C(=O)CCC2=CC=C(F)C=C12 | 10 | I |
| CCC1=NN(C)C(CN2C=CC(=O)C3=C2C=CC=C3)=C1Br | 10 | I |
| CC(C)N(C)C(=O)[C@H]1CSCN1C(=O)C1=CSC2=C1C=CC=C2 | 10 | I |
| CN([C@@H]1CCS(=O)(=O)C1)C(=O)C1=C(Cl)C=CC(Cl)=C1Cl | 10 | I |
| COC1=CC=C(CN2C(C)=NC=C(Br)C2=O)C=C1F | 10 | I |
| CC1=CN2C(S1)=NC(C)=C2CN1CCC[C@H](C1)C1=NC(C)=CS1 | 10 | I |
| C[C@@H]1[C@@H](C)S(=O)(=O)CCN1CC1=CN=C(S1)C1=CSC=C1 | 10 | I |
| IC1=CC=C(C=C1)C(=O)CN1CCO[C@H](C1)C#N | 10 | I |
| C[C@H]1OCC[C@@H]1C(=O)C1=CC2=C(CCN2C(C)=O)C=C1Br | 10 | I |
| FC1=CC(F)=C(N2CC(=O)N3CCCC[C@@H]3C2=O)C(Br)=C1 | 10 | I |
| C[C@H]1CN2CCC[C@@H]2CN1S(=O)(=O)C1=CSC(CCl)=C1 | 10 | I |
| CS(=O)(=O)[C@H]1CCC[C@@H](C1)N1C=C(I)C=N1 | 10 | I |
| CC1=NC(=NN1CCC#N)C1=CC=C(I)C=C1 | 10 | I |
| O=C1COC2(CCN(CC3=CSC=C3)CC2)CN1C1=CSC=C1 | 10 | I |
| FC1=CC=C(C=C1)S(=O)(=O)C1CN(C1)C(=O)C1=CC=C(Cl)C=C1 | 10 | I |
| CC1(C)CN(CCS1(=O)=O)C(=O)[C@@H]1C[C@@H]1C1=CC=C(F)C=C1 | 10 | I |
| CN1C(C)=CC(\C=C2\SC(=S)N(CC3=CC=CO3)C2=O)=C1C | 10 | I |
| FC1=CC=C(C=C1)C(=O)N1CCN(CN2CCSC2=S)CC1 | 10 | I |
| CC1=C(C)N(CC(=O)N2CCC[C@H]2C2=CC=C(F)C=C2)C(=O)S1 | 10 | I |
| FC1=CC=C(C=C1)N1C(=S)S\C(=C\C2=CC=CO2)C1=O | 10 | I |
| BrC1=CC=C(S1)S(=O)(=O)N1C=CC=C1 | 10 | I |
| FC1=CC=C(CN2C(=O)[C@@H]3[C@H]([C@@H]4C=C[C@H]3[C@@H]3C[C@H]43)C2=O)C=C1 | 10 | I |
| CC(=O)[C@H]1CC[C@H]2[C@H]3CCC4=CC(=O)C=C[C@]4(C)[C@@H]3C(=O)C[C@]12C | 10 | I |
| CC1=CN=C(S1)N1[C@H](SCC1=O)C1=CC=C(F)C=C1 | 10 | I |
| FC1=C(C=CC(Br)=C1)C1=NN=C(CCl)O1 | 10 | I |
| C[C@H]1CCC2=C(C1)SC=C2C(=O)N1C[C@H](C)O[C@H](C)C1 | 10 | I |
| C[C@@H]1CN([C@@H](C)CO1)C(=O)C1=CC=C(Br)C=C1 | 10 | I |
| C[C@@H]1CN([C@H](C)CO1)C(=O)C1=CC(Br)=CC=C1F | 10 | I |
| CN([C@H]1CCSC1)C(=O)C1=C(Cl)C=CC(Cl)=N1 | 10 | I |
| CC[C@@H]1CO[C@@H](C)CN1C(=O)C1=CC=C(Br)C=C1 | 10 | I |
| C[C@]12CC[C@H]3[C@@H](CCC4=CC(=O)CC[C@]34C=O)[C@H]1CCC2=O | 10 | I |
| CC[C@@H]1SC2=NC(=S)N(C3=CC=CC=C3)C(=O)C2=C1C | 10 | I |
| BrC1=CC=C2O\C(=C/C3=CN=CC=C3)C(=O)C2=C1 | 10 | I |
| CN1C(=O)CC2=CC(=CC=C12)[C@@H](Br)C1=C(C)OC=C1 | 10 | I |
| FC1=C(CN2CCCS2(=O)=O)C=C(Br)C=C1 | 10 | I |
| C[C@@H]1CN(CCO1)[C@@H](C#N)C1=CC=C(Br)C=C1 | 10 | I |
| FC1=C2N(CCBr)C(=O)C(=O)C2=C(Cl)C=C1 | 10 | I |
| CN(C)C(=O)C1=C(C)N(C(C)=C1)C1=CC=C(Br)C=C1 | 10 | I |
| FC1=C(C=CC(Br)=C1)C(=O)[C@@H]1CCS(=O)(=O)C1 | 10 | I |
| FC1=C(CN2C(=O)CCCCC2=O)C=CC(Br)=C1 | 10 | I |
| CN(C)[C@H]1COC2=C(C=C(Br)C(C)=C2C)C1=O | 10 | I |
| C[C@@H]1CC(=O)N(C1=O)C1=CC=C(I)C=C1 | 10 | I |
| CC1=CN(CC2=CC=C(F)C=C2Br)C(=O)N=C1 | 10 | I |
| CC1=CC(C)=NC(SC2=CC(=O)[C@H]3[C@H]4C[C@H](C=C4)[C@@H]3C2=O)=C1 | 10 | I |
| C[C@@H](C#N)N1CCN(CC1)C1=C(F)C(F)=C(F)C(F)=C1F | 10 | I |
| FC1=CC=CC(=N1)N1C=C(I)C=N1 | 10 | I |
| IC1=CN(C[C@@H]2CCCCO2)N=C1 | 10 | I |
| FC1=C(F)C=C(CN2C(=O)CC3(CCCCC3)C2=O)C=C1 | 10 | I |
| C[C@@H](Cl)C1=NN=C(O1)C1=C(Br)C=CS1 | 10 | I |
| CC1=CC2=NC(CI)=CC(=O)N2C=C1 | 10 | I |
| CC1=CSC2=NC(CI)=CC(=O)N12 | 10 | I |
| O=S(=O)([C@H]1CCCC[C@@H]1C#N)C1=CC2=C(CCC2)C=C1 | 10 | I |
| ClC1=CC(=C(Cl)C=C1)S(=O)(=O)[C@H]1CCCC(=O)C1 | 10 | I |
| CC1=C(Br)C(Cl)=NC(C[C@H]2CCCO2)=N1 | 10 | I |
| C[C@H]1CCN(C1)C1=C(I)C=NC=N1 | 10 | I |
| BrC1=CC=C(C=C1)S(=O)(=O)C1(CC2(CC2)C1)C#N | 10 | I |
| FC1=CC(Br)=CC(F)=C1N1C[C@H](CC1=O)C#N | 10 | I |
| CC1=NC=C2N1C(I)=CN=C2Cl | 10 | I |
| C[C@@H]1CCN(CC2=C(Br)C(C)=NN2C)[C@@H](C)C1 | 10 | I |
| FC1=CC(CN2C(=O)[C@H]3C[C@H]3C2=O)=C(Br)C=C1 | 10 | I |
| CC1=NC2=C(S1)C=C(OC1CC(=O)C1)C1=C2SC=C1 | 10 | I |
| FC1=CC(Br)=CC(CN2C(=O)[C@H]3C[C@H]3C2=O)=C1 | 10 | I |
| IC1=CC=CN(C[C@@H]2CCCCO2)C1=O | 10 | I |
| C[C@@H]1C[C@@H](C)[C@H](CN2C[C@@H]3CCCCN3C[C@@H]2C)C(=O)C1 | 10 | I |
| C[C@H]1CN2CCCC[C@@H]2CN1C[C@@H]1CC(C)(C)CCC1=O | 10 | I |
| CCC1=NN=C(O1)C1=C(F)C=C(Br)C=C1F | 10 | I |
| CN1C(=O)CC2=CC(=CC=C12)[C@@H](Br)[C@]1(C)CCCO1 | 10 | I |
| C[N+]1([O-])CCN(CC1)[C@H]1CC2=CC=CC=C2SC2=C1C=CC=C2 | 10 | I |
| C1CC2=C(C1)C1=C(N=CN=C1S2)N1CCCSCC1 | 10 | I |
| COC(=O)[C@H]1CC2=CC(=O)CC[C@@]2(C)C2=CC[C@]3(C)[C@H](CC[C@@]33CCC(=O)O3)[C@H]12 | 10 | I |
| FC1=CC=C(C=C1)C(=O)N1CCN(CC1)S(=O)(=O)C1=CC=C(Cl)C=C1 | 10 | I |
| CC1=C(SC=C1)C(=O)N1CCN(CC1)S(=O)(=O)C1=CC=CC=C1F | 10 | I |
| FC1=CC(=CC(F)=C1)[C@H]1[C@@H]2C(=O)CCCC2=NC2=NC(=NN12)C(F)(F)F | 10 | I |
| CC(=O)N1N=C(O[C@@H]1C1=CC=CO1)C1=CC=CC(I)=C1 | 10 | I |
| ClC1=CC=C(C(=O)\N=C2/S[C@H]3CS(=O)(=O)C[C@@H]3N2CC=C)C(Cl)=C1 | 10 | I |
| CN1C(=O)S\C(=C/C2=C(C)N(C(C)=C2)C2=C(C#N)C(C)=C(C)S2)C1=O | 10 | I |
| COC1=CC=C(Br)C=C1\C=C1/SC(=NC1=O)N1CCOCC1 | 10 | I |
| CCC12CN3CC(C)(CN(C1)C31C(=O)N(C)C3=CC=C(Br)C=C13)C2=O | 10 | I |
| ClC1=CC2=C(OC[C@@](CN3CCOCC3)(SC#N)C2=O)C(Cl)=C1 | 10 | I |
| C[C@]1(CC1(Cl)Cl)C(=O)N1CCN(CC2=CC=C3OCOC3=C2)CC1 | 10 | I |
| CC[C@@H]1CN(CCO1)S(=O)(=O)C1=CC(Br)=CN=C1Cl | 10 | I |
| CC[C@@H]1CO[C@H](C)CN1S(=O)(=O)C1=CC(Br)=CN=C1Cl | 10 | I |
| C[C@H]1CN(CC(C)(C)O1)S(=O)(=O)C1=CC(Br)=CN=C1Cl | 10 | I |
| ClC1=CC(C(=O)N2CCC[C@H]2C2=NN=C3CCCCCN23)=C(Cl)S1 | 10 | I |
| IC1=CC=C(C=C1)C(=O)CN1N=C2CCCCCN2C1=O | 10 | I |
| C[C@@H](Cl)C1=NC2=CC(Br)=CC=C2N1[C@H](C)C1=NN=CN1C | 10 | I |
| CCN([C@@H]1CCS(=O)(=O)C1)C(=O)C1=CC2=C(S1)C1=CC=CC=C1OC2 | 10 | I |
| BrC1=CC=C(C=C1)N1C=CC=C1\C=C(\C#N)C(=O)N1CCOCC1 | 10 | I |
| CN1[C@H](SCC2=CC=C(Br)C=C2F)N=C2C1=NC=NC2=O | 10 | I |
| CC1CCN(CC1)C(=O)C1=CSC(=C1)S(=O)(=O)N1CCCCCC1 | 10 | I |
| CC(C)(C)C(=O)\N=C1\S[C@H]2CS(=O)(=O)C[C@@H]2N1CC1=CC=CC=C1Cl | 10 | I |
| FC1=CC=CC=C1N1[C@@H]2CS(=O)(=O)C[C@H]2S\C1=N\C(=O)C1=CC=CC=C1 | 10 | I |
| CN1CCN(C)C2(CCN(CC3=C(Cl)N=C(S3)N3CCCC3)CC2)C1 | 10 | I |
| CN1[C@@H]2C=C[C@@H]([C@@H]3[C@@H]2C(=O)N(C3=O)C2=CC=C(CC3=CC=CC=C3)C=C2)C1=O | 10 | I |
| O=C([C@H]1[C@H]2[C@H]3CO[C@H](O3)C(=O)[C@H]2[C@H]2N1C=CC1=C2C=CC=C1)C1=CC=CC=C1 | 10 | I |
| COC1=C(Br)C=C(Cl)C=C1S(=O)(=O)N1CCN(C)CC1 | 10 | I |
| CC(=O)[C@@H]1[C@H]2[C@@H]([C@H]3N1C=CC1=C3C=CC=C1)C(=O)N(C2=O)C1=CC=C(Cl)C=C1 | 10 | I |
| FC1=CC=C(Cl)C(=C1)C(=O)N1CCN(CC2=CC3=C(OCO3)C=C2)CC1 | 10 | I |
| CN1C(=CC2=CC(F)=CC=C12)C(=O)N1CCN(C(=O)C1)C1=CC(C)=CC(C)=C1 | 10 | I |
| CN1C(=O)\C(S\C1=C(/C#N)C(=O)C(C)(C)C)=C\C1=CC=C2OCCC2=C1 | 10 | I |
| COC1=CC=C(C(=O)N2CC(=O)N(C[C@@H]2C)C2=CC=C(C)C=C2)C(F)=C1F | 10 | I |
| C[C@H]1CN(CC(C)(C)O1)C(=O)C1=CC=CC=C1N(C)C(=O)C1=CC=CS1 | 10 | I |
| C[C@H](N1CCN(CC1)C(=O)C1=CC2=C(CCCC2)S1)C(=O)N1CCCC1 | 10 | I |
| CC(C)(C)OC(=O)N1CCN2C(Br)=NC(Br)=C2C1 | 10 | I |
| O=C(N1CCCCC[C@H]1[C@@H]1CCCCC1=O)C1=CN=C(S1)[C@@H]1CCCO1 | 10 | I |
| BrC1=CC(=CC=C1)C(=O)N1CCN(CC2=CN=C3C=CC=CN23)CC1 | 10 | I |
| C[C@@H]1OC2=CC=CC=C2O[C@@H]1C(=O)N1CCCSC[C@@H]1CN1CCCC1 | 10 | I |
| C[C@@H]1CN(CC(C)(C)O1)S(=O)(=O)C1=C(Br)OC(CCl)=C1 | 10 | I |
| C[C@@H]1CN(CC2=CN(C)N=C2C2=CC=C3OCCCOC3=C2)C[C@@H](C)S1 | 10 | I |
| CN(C)C(=O)C1=CC=C(Cl)C(=C1)S(=O)(=O)N1CCC2=CC(F)=CC=C2C1 | 10 | I |
| CCN1C(=O)CCC[C@@]11CCCN(C1)S(=O)(=O)C1=CC=C(F)C(F)=C1 | 10 | I |
| FC1=CC=C(C=C1)N1[C@H]2CS(=O)(=O)C[C@H]2S\C1=N/C(=O)C1=CC=CC=C1 | 10 | I |
| FC1=CC=CC=C1N1[C@H]2CS(=O)(=O)C[C@H]2S\C1=N/C(=O)CC1=CC=CC=C1 | 10 | I |
| FC1=CC(CS(=O)(=O)N2CC[C@@]3(C2)CC(=O)C2=CC=CC=C2O3)=CC=C1 | 10 | I |
| CC1=NOC(CN2C(CCl)=NC3=CC(I)=CC=C23)=N1 | 10 | I |
| C[C@@H](Cl)C1=NC2=CC(Br)=C(F)C=C2N1CC1=NN=CN1C | 10 | I |
| C[C@@H]1C[C@@H]1C(=O)N1CCN(CC1)S(=O)(=O)C1=CC=C(Br)C=C1 | 10 | I |
| BrC1=CC=C2OCC(=O)N(CC3=CC4=C(OCO4)C=C3)CC2=C1 | 10 | I |
| FC1=CC=C(C=C1)N1C(=O)[C@@H]2[C@H]3CCCN3[C@H]([C@@H]2C1=O)C(=O)C1=CC=CC=C1 | 10 | I |
| CSC1=NCCCN1C(=O)C1=C(C)ON=C1C1=C(F)C=CC=C1Cl | 10 | I |
| BrC1=C(I)OC(\C=N\N2C=NN=C2)=C1 | 10 | I |
| BrC1=CN(N=C1)C(=O)CN1C=C(I)C=N1 | 10 | I |
| C[C@H]1CN(C[C@H](C)O1)C(=O)C1=C(Br)C(=NN1C)C(F)(F)F | 10 | I |
| FC1=C(Cl)C=C(C=C1)N1C(=O)C[C@H](N2CCC(CC2)C2=CC=NC=C2)C1=O | 10 | I |
| FC1=CC=C(\C=C2/SC(N(C[C@@H]3CCCO3)C2=O)=C(C#N)C#N)C=C1 | 10 | I |
| C[C@@H]1N(CCN2C=CC=C12)C(=O)CN1C2=CC=CC=C2C(=O)C2=C1C=CC=C2 | 10 | I |
| COC1=CC(=CC(OC)=C1Br)C(=O)N1C[C@H](C)OC[C@@H]1C | 10 | I |
| CN1C(Cl)=CN=C1CN1C(=O)C(=O)C2=C(Br)C=CC=C12 | 10 | I |
| ClC1=C(N2CCOCC2)C(=O)N(C1=O)C1=CC=C(Br)C=C1 | 10 | I |
| CSC1=NCCN1C(=O)C1=C(C)ON=C1C1=C(F)C=CC=C1Cl | 10 | I |
| C[C@@H]1CN(CC2=NC(=O)C3=C(CSC3=N2)C2=CC=CC=C2)C[C@@H](C)O1 | 10 | I |
| ClC1=C(Cl)C=C(C=C1)C(=O)N1CCC(CC1)C1=NN=C2CCCN12 | 10 | I |
| CN1CCN([C@H](C1)C1=CC=CC=C1)C(=O)CN1C=C(Br)C=CC1=O | 10 | I |
| CN1CCC(CC1)N1N=C(C(C(=O)N2CCCCC2)=C1C)C(F)(F)F | 10 | I |
| CN1C(=O)N(C)C2=CC([C@@H](Cl)[C@@H]3COCCO3)=C(Br)C=C12 | 10 | I |
| CN1C(=O)N(C)C2=CC([C@@H](Cl)[C@H]3COCCO3)=C(Br)C=C12 | 10 | I |
| C[C@H]1C[C@@H](C)CN(C1)C(=O)C1=CC(=CC=C1Cl)S(=O)(=O)N(C)C | 10 | I |
| FC1=CC=C(C=C1)C(=O)N1CCN(CC1)S(=O)(=O)C1=CC(F)=CC=C1F | 10 | I |
| C[C@H]1C[C@@H](C)CN(C1)C(=O)CN1CCN(CC1)C(=O)C1=CC(F)=CC(F)=C1 | 10 | I |
| CC(C)C(=O)N1CCN(CC1)C(=O)C1(CCOCC1)C1=CC=C(F)C=C1 | 10 | I |
| O=C1C[C@@H](C2=CC=CO2)S(=O)(=O)C2=CC=CC=C2N1CC1=CC=CC=C1 | 10 | I |
| C[C@H]1C[C@@H](SC2=NN=C(O2)C2=CC=CC=C2Br)C(=O)O1 | 10 | I |
| COC1=CC=C(C=C1Br)S(=O)(=O)N1CCCO[C@@H](C)C1 | 10 | I |
| C[C@H]1SCC(=N1)C1=CC=C2N(CCC2=C1)C(=O)C1=C[C@@H](Cl)C(=O)N=C1 | 10 | I |
| COC1=CC=CC=C1[C@@H]1CC(=O)N(C2=C1C(=O)OC2)C1=CC=C(Cl)C=C1 | 10 | I |
| COC1=CC=CC=C1[C@@H]1CC(=O)N(C2=C1C(=O)OC2)C1=CC(C)=CC(C)=C1 | 10 | I |
| ClC1=C(Cl)C=C(C=C1)[C@H]1CN(CCO1)C(=O)CN1CCCC1=O | 10 | I |
| CN1C=CC=C(C(=O)N2CCO[C@@H](C2)C2=CC=C(Cl)C(Cl)=C2)C1=O | 10 | I |
| CC1=CC(CN2CCN(CC2)C(=O)C2=C(Cl)SC(Cl)=C2)=NO1 | 10 | I |
| C[C@@H]1CN(C[C@@H]2CCCN2C(=O)[C@@H]2CC3=CC(F)=CC=C3O2)C[C@@H](C)O1 | 10 | I |
| CN1C=CC=C1C(=O)N1CCN(CC1)C(=O)C1=CC=CC(Cl)=C1Cl | 10 | I |
| IC1=CN(CC(=O)C2=CC3=C(OCO3)C=C2)N=C1 | 10 | I |
| FC1=CC=CC(CC(=O)N2CCC[C@@H]2C2=NN=C3CCCCCN23)=C1F | 10 | I |
| C[C@H]1CN(C(=O)CCN2C(=O)OC3=CC=CC=C23)C2=CC=CC=C2S1 | 10 | I |
| C[C@H]1CN(CCN1C)S(=O)(=O)C1=C(Br)OC(CCl)=C1 | 10 | I |
| FC1=C(Br)C=C2C(=O)C(=O)N(CCC3OCCCO3)C2=C1 | 10 | I |
| CN1CCC[C@@]2(CCN(CC3=CC=C4COC(=O)C5=CC=CC3=C45)C2)C1=O | 10 | I |
| FC1=CC(CN2CC3(CC2=O)CCN(CC3)C2=NC=CN=C2)=CC(F)=C1 | 10 | I |
| CN(C1CCN(CC2=CN=C3C=CC(Br)=CN23)CC1)C(C)=O | 10 | I |
| C[C@@]1(CCS(=O)(=O)C1)N1C=C(CN2CCS[C@H]3CCCC[C@H]23)C=N1 | 10 | I |
| C[C@@H]1CN(CC(C)(C)O1)C(=O)C1=C(C)N(N=C1C)C1=CC=C(F)C=C1F | 10 | I |
| CN1CCN2C(=CN=C2C11CCN(CC2=NC=CS2)CC1)C1=CC=CC=C1 | 10 | I |
| C(N1CCC2(CN(C3=CC=CC=C23)C2=NC=CC=N2)CC1)C1=CC=CN=C1 | 10 | I |
| CN1CC(=O)N(C[C@@]11CCN(C1)C(=O)C1=CC(F)=CC(F)=C1)C1=CSC=C1 | 10 | I |
| FC1=CC=C(C=C1)N1C[C@@]2(CCN(C2)S(=O)(=O)C2=CC=CC=C2)CC1=O | 10 | I |
| BrC1=C(I)OC(\C=N/N2C=NN=C2)=C1 | 10 | I |
| CN(CC1=CC2=C(OCO2)C(Br)=C1)[C@H]1CCS(=O)(=O)C1 | 10 | I |
| O=C(N1CCS[C@@H](CC1)C1=CC=CO1)C1=CC2=CC=CC=C2OC1=O | 10 | I |
| O=C(N1CCS[C@H](CC1)C1=CC=CO1)C1=CC=C(C=C1)N1CCOCC1 | 10 | I |
| O=C(N1CCN(CC1)C(=O)C1=CC=C2OCCC2=C1)C1=CC2=C(OCC2)C=C1 | 10 | I |
| CN(C)C1=CC=C(C=C1)N1[C@@H]2CS(=O)(=O)C[C@H]2S\C1=N/C(=O)CCl | 10 | I |
| O=C(CCC(=O)N1CCCCCC1)N1C[C@H]2C[C@@H](C1)C1=CC=CC(=O)N1C2 | 10 | I |
| FC1=CC=C2OC3=C([C@@H](N(C4=NC=CS4)C3=O)C3=CN=CC=C3)C(=O)C2=C1 | 10 | I |
| C[C@H]1CC2=C(S1)C(=O)N(C)C(SCC(=O)N1C[C@@H](C)O[C@H](C)C1)=N2 | 10 | I |
| O=C(C[C@H]1CCS(=O)(=O)C1)N1CC2=CC=CC=C2OC2(CCOCC2)C1 | 10 | I |
| FC1=CC=C(C=C1)C1=CSC2=C1C(=O)N(CC(=O)N1CCCC1=O)C=N2 | 10 | I |
| FC1=C(F)C=C(CN2CCC[C@]3(CCN(C3)C(=O)C3=CSN=N3)C2=O)C=C1 | 10 | I |
| CN(CC1=C(C)N(C)N=C1C)C(=O)C1=C(I)C=NN1C | 10 | I |
| O=C1\C(OC2=C1C=CC1=C2CN(CC2=CC=NC=C2)CO1)=C\C1=CC=NC=C1 | 10 | I |
| CC1=NN2C(=C1)N=CC(C(=O)N1CCN(CC3=CC=C(Cl)C=C3)CC1)=C2C | 10 | I |
| CC1=CC2=C(C=C1C)C(CN1CCN(CC1)[C@H]1CCS(=O)(=O)C1)=CC(=O)O2 | 10 | I |
| O=C([C@@H]1CCCO1)N1CCN(CC2=CC(=O)OC3=C2C=C2CCCC2=C3)CC1 | 10 | I |
| FC1=CC=CC=C1C(=O)N1CCN(CC2=CC(=O)N3C=CSC3=N2)CC1 | 10 | I |
| BrC1=CC2=C(O[P@](=O)([C@H]2N2CCOCC2)N2CCOCC2)C=C1 | 10 | I |
| CN1CCN(CC1)S(=O)(=O)C1=CC2=CC(Br)=CC=C2OC1=O | 10 | I |
| FC1=CC=C(C=C1)C(=O)N1CCN(CC1)C(=O)[C@H]1COC2=CC=CC=C2O1 | 10 | I |
| C[C@H]1CN(C[C@@H](C)O1)C(=O)C1=C(C)N(N=C1C(F)(F)F)[C@H]1CCCN(C)C1 | 10 | I |
| C[C@H](N1CCN(CC1)C(=O)C1=CC=CC=C1)C1=NC(=O)C2=C(C)[C@@H](C)SC2=N1 | 10 | I |
| C[C@H](N1CCN(CC1)C(=O)C1=CC=CC=C1)C1=NC(=O)C2=C(C)[C@H](C)SC2=N1 | 10 | I |
| CC(C)C(=O)N1CCN(CC1)S(=O)(=O)C1=CC(Br)=CN=C1 | 10 | I |
| C[C@H]1C[C@H](C)CN(C1)C(=O)C1=CC(=CC=C1F)S(=O)(=O)N1CCOCC1 | 10 | I |
| CN1C(=O)N(C(=O)\C(=C/C2=CC=C(Br)O2)C1=O)C1=CC=CC=C1 | 10 | I |
| FC(F)(F)C(=O)N1CCC(CC1)C1=CC2=C(C=NN2C=C1)C(=O)N1CCCC1 | 10 | I |
| CC1=NC2=C(C=NN2C(=C1)C(F)(F)F)C(=O)N1CCO[C@H](C1)C(F)(F)F | 10 | I |
| CN1[C@@H]2CN(C[C@]2(C)C(=O)C2=CC=CC=C2C1=O)C(=O)COC1=CC=CC=C1F | 10 | I |
| ClC1=CC=CC2=C1C=CN2CC(=O)N1CCN(CC1)[C@@H]1CCS(=O)(=O)C1 | 10 | I |
| FC(F)(F)C1=NN=C(O1)[C@@H]1CCCN(C1)C(=O)C1=CC=C(Br)O1 | 10 | I |
| CCN1CCN([C@H]2CS(=O)(=O)C[C@@H]12)C(=O)C1=CC2=CC=CC=C2OC=C1 | 10 | I |
| CN1CCN(CC1)S(=O)(=O)C1=CC(Br)=CC2=C1N(CC2)C(C)=O | 10 | I |
| O=C(CN1C[C@H]2C[C@@H](C1)C1=CC=CC(=O)N1C2)C1=CC=C2OCCCOC2=C1 | 10 | I |
| CO[C@H]1C[C@H]2C(C)=C(OC)C(=O)[C@H]3[C@@]2(C)[C@@H](C[C@@H]2[C@@H](C)C=C(OC)C(=O)[C@@]32C)O1 | 10 | I |
| CC(C)(C)N1C[C@]23O[C@H](C=C2)[C@@H]([C@@H]3C1=O)C(=O)N1CC(C1)N1CCCCC1 | 10 | I |
| CCN1CC[C@@]2(CN(CC3=CC=C4OCCOC4=C3)CCN2C)CCC1=O | 10 | I |
| CN1C=NN=C1C1CCN(CC2=CN=C3C=CC(Br)=CN23)CC1 | 10 | I |
| CN1CCC[C@@]2(CCN(C2)C(=O)CC2=CC3=C(OCCO3)C=C2Cl)C1=O | 10 | I |
| ClC1=C(C=CC(Br)=C1)C(=O)N1CCOC[C@@H]1C1=NOC=N1 | 10 | I |
| CCN1C[C@H]2C3(CC[C@]2(C1)C(=O)N1CCCC1)CCN(CC3)C(=O)N(C)C | 10 | I |
| O=C([C@H]1CN2C=CN=C2C2(CCN(CC3=CC=CS3)CC2)O1)N1CCCC1 | 10 | I |
| FC1=CC=C(C=C1)N1C[C@]2(COCCN(CC3=NC=CS3)C2)OCC1=O | 10 | I |
| CN(C)C(=O)C[C@@H]1CC2(CCN(CC2)C(=O)C2=CN=CC=N2)C2=C1C=CC=C2 | 10 | I |
| O=C(CC1=CC=CS1)N1C[C@H](C(=O)N2CCOCC2)C2(C1)CCOCC2 | 10 | I |
| CC1=NC(=CN1C1=NC=CS1)C1=CC(C(=O)N2CCCC2)=C2C=CC=CN12 | 10 | I |
| ClC1=CC=C(C=C1)C(=O)N1C[C@@H](C(=O)N2CCOCC2)C2(C1)CCOCC2 | 10 | I |
| CC(C)N1CCN(CC1)C(=O)[C@@H]1CN(CC11CCCCC1)C(=O)C1=CC=NC=C1 | 10 | I |
| FC(F)(F)C1=NN=C(O1)C1CCN(CC1)C(=O)C1=CC=C(Br)O1 | 10 | I |
| FC(F)(F)C1=NN=C2CN(CCN12)C1=NC=NC=C1I | 10 | I |
| FC1=C(F)C=C(C=C1)S(=O)(=O)N1CC(C1)N1C(=O)[C@@H]2CC=CC[C@H]2C1=O | 10 | I |
| ClC1=CC(Cl)=C(C=C1)C(=O)N1CCN(CC1)C(=O)C1=C2CCCCN2N=C1 | 10 | I |
| FC1=CC(\C=C\C(=O)N2CCN(CC2)C(=O)C2=C3CCCCN3N=C2)=C(F)C=C1 | 10 | I |
| CC1=C(C)C2=C(S1)N1\C(CSC1=NC2=O)=C\Br | 10 | I |
| O=S1OCCSC2=C(SC(=S)S2)SCCO1 | 10 | I |
| Cl[C@H]1CS(=O)(=O)C[C@@H]1\N=C\C1=CC=C(Br)C=C1 | 10 | I |
| Cl[C@@H]1CS(=O)(=O)C[C@H]1\N=C\C1=CC=C(Br)C=C1 | 10 | I |
| CN1C=CC=C1\C=C1\SC(=S)N(C1=O)C1=CC=CC=C1F | 10 | I |
| BrC1=CC=C(S1)\C=N\N1C(=O)CSC1=S | 10 | I |
| CC(=O)[C@H]1CC[C@@H]2[C@@H]3CC(=O)C4=CC(=O)CC[C@@]4(C)[C@@H]3CC[C@]12C | 10 | I |
| FC1=C(CN2C(=O)CC3(CCCC3)C2=O)C=CC(Br)=C1 | 10 | I |
| CC1=CC=C(C=C1)C1CC(=O)N(C(=O)C1)C1=CC=C(F)C(C)=C1 | 10 | I |
| CC(=O)OC1=CC(Br)=CN=C1I | 10 | I |
| CC(=O)[C@@]12O[C@@H]1C[C@H]1[C@H]3CCC4=CC(=O)CC[C@@]4(C)[C@@H]3CC[C@]21C | 10 | I |
| C[C@@H]1CCC2=C3C(S[C@@H]2C1)=NC(=S)N(CC(C)=C)C3=O | 10 | I |
| CN1C(=O)CC2=CC(=CC=C12)[C@H](Br)C1=C(C)OC(C)=C1 | 10 | I |
| BrC1=CC=CC=C1CN1C(=O)CC2(CCCC2)C1=O | 10 | I |
| BrC1=CC=CC=C1CN1C(=O)[C@H]2[C@H]3C[C@H](C=C3)[C@@H]2C1=O | 10 | I |
| C[C@H]1CN(CCO1)C1=C(CCl)C=C(Br)C=N1 | 10 | I |
| CC1(C)CC2=CC(=CC=C2O1)[C@H](Br)[C@H]1COCCO1 | 10 | I |
| C[C@H]1CC(=O)[C@@]2(C)[C@@H]3CC[C@@H]4CC=CC[C@@H]4C3=CC[C@@H]2S1(=O)=O | 10 | I |
| FC1=C(C=C(Br)C=C1)S(=O)(=O)N1CCSCC1 | 10 | I |
| IC1=NN=C(S1)[C@H]1COC2=CC=CC=C12 | 10 | I |
| C[C@@H]1CSCCN1S(=O)(=O)C1=CC=C(Br)S1 | 10 | I |
| CCN1C=C(Br)C=C1C(=O)N1C[C@@H](C)S[C@H](C)C1 | 10 | I |
| FC(F)(F)C1=C(C=CC(Br)=C1)N1C[C@@H](CC1=O)C#N | 10 | I |
| ClC1=NC=CC(=C1)N1C=C(I)C=N1 | 10 | I |
| BrCCN1C2=CC=CC3=CC=CC(=C23)S1(=O)=O | 10 | I |
| FC1=CC=CC=C1N1C(=S)S\C(=C/C2=CC=CO2)C1=O | 10 | I |
| CCC1=C(I)C(Cl)=NC(=N1)[C@@H]1CCOC1 | 10 | I |
| ClC[C@@H]1CCCN1C1=NC=NC=C1I | 10 | I |
| BrC1=CC=C(C=C1)N1C(=O)[C@H]2CC(=C)C[C@H]2C1=O | 10 | I |
| ClC1=NC=NC(C2=CSC=C2Br)=C1C=O | 10 | I |
| CC1=NN(C[C@H]2CCOC2)C(C)=C1I | 10 | I |
| CCN1C(=O)COC2=CC(I)=CC=C12 | 10 | I |
| C[C@@H]1CC[C@@H](C[C@@H]1C)N1C=NC(I)=N1 | 10 | I |
| CCC1=C(Br)C(Cl)=NC(=N1)[C@@H]1CSCCO1 | 10 | I |
| CN1CCN(CC1)[C@H](C#N)C1=CC(Br)=CC=C1F | 10 | I |
| CN1N=C(C)C(Br)=C1CN1CCC[C@H]2CCC[C@H]12 | 10 | I |
| C[C@@H]1CCC[C@@H](C)N1C1=C(I)C=NC=N1 | 10 | I |
| CC1(C)CCCN(C1)C1=C(I)C=NC=N1 | 10 | I |
| C[C@H]1CN(C2=CC=C(I)C=C2)S(=O)(=O)C1 | 10 | I |
| FC1=CC(Br)=CC(CN2CCC3(CC2)OCCO3)=C1 | 10 | I |
| C[C@H]1OCC[C@@H]1[C@H](Br)C1=CC=C2N(C)C(=O)CC2=C1 | 10 | I |
| CC1=CC(=O)N(C[C@H]2CCCO2)C=C1I | 10 | I |
| ClCC#CC1=CC(O[C@@H]2CCO[C@@]3(CCSC3)C2)=CN=C1 | 10 | I |
| ClC1=NC(=NC(C2CC2)=C1Br)[C@@H]1CSCCO1 | 10 | I |
| CN1C(=O)CCC2=CC(=CC=C12)[C@@H](Br)[C@@]1(C)CCCO1 | 10 | I |
| ClCC1=CN2C=C(Br)N=C(Br)C2=N1 | 10 | I |
| BrC1=CC=CC=C1N1C(=O)[C@@H]2CC=CC[C@H]2C1=O | 10 | I |
| CSC1=N\C(=C\C2=CC=C(Br)O2)C(=O)S1 | 10 | I |
| BrC1=CC=CC=C1\C=C1/SC(=O)N(CC#C)C1=O | 10 | I |
| CC[C@H]1SCC2=C(C1=O)C1=C(OCCO1)C=C2Br | 10 | I |
| CC(=O)\N=C1/SC=CN1CC(=O)C1=CC=C(I)C=C1 | 10 | I |
| CC1=CC=C(C=C1)N1[C@H](C2=CSC=C2)C(=O)N(CC1=O)C1=CC=CC=C1F | 10 | I |
| BrC1=CC=C(C=C1)N([C@H]1CS(=O)(=O)C=C1)C(=O)C1=CC=CC=C1 | 10 | I |
| BrC1=CC=C(C=C1)C(=O)CN1C=NC2=C(C3=C(CCC3)S2)C1=O | 10 | I |
| IC1=CC=C(C=C1)S(=O)(=O)N1C=NC2=CC=CC=C12 | 10 | I |
| C[C@H]1C[C@H]2[C@@H]3CC[C@](OC(C)=O)(C(C)=O)[C@]3(C)CC[C@@H]2[C@@]2(C)C=CC(=O)C=C12 | 10 | I |
| C[C@H]1CCC2=CC(F)=CC=C2N1C(=O)CN1N=C(C(F)F)C(Cl)=C1C | 10 | I |
| C[C@@H]1N(CCN2C(C)=CC=C12)S(=O)(=O)C1=CC=C(Br)S1 | 10 | I |
| C[C@@H]1CN(C[C@@H](C)O1)S(=O)(=O)C1=CC=C(Br)C=C1Cl | 10 | I |
| FC(F)(F)C1=CN(CC2=CC3=C(OCCO3)C=C2Br)C(=O)C=C1 | 10 | I |
| BrC1=CC=CC=C1CN1C(=O)S\C(=C\C2=CC=C(C=C2)C#N)C1=O | 10 | I |
| FC1=CC=C(C=C1F)N1[C@H]2C[C@H](N(C2)C(=O)CC2=CC=CC=C2Cl)C1=O | 10 | I |
| CC1=NC(SCC2=CC3=C(OCCO3)C=C2Br)=C(C#N)C(C)=C1 | 10 | I |
| BrC1=CC=C(C=C1)C1=NC=NN1C1=CC=C(Br)N=C1 | 10 | I |
| C[C@H]1CN(C[C@@H](C)O1)C1=NC(=O)\C(S1)=C/C1=CC=C(Br)C=C1 | 10 | I |
| CC1=NN(C2=NC(=CC(=C12)C(F)(F)F)C1=CC=NC=C1)C1=CC=C(F)C=C1 | 10 | I |
| ClC1=CC(=CC(Cl)=C1)N1C(=O)[C@@H]2[C@H]([C@@H]3C(=O)C[C@H]2C2=CC=CC=C32)C1=O | 10 | I |
| O=C1[C@H]2[C@@H]3CSCN3[C@@H](\C=C\C3=CC=CC=C3)[C@@H]2C(=O)N1C1=CC=CC=C1 | 10 | I |
| CC(C)CN1C(=O)OC(=O)C2=C1C(Br)=CC(Br)=C2 | 10 | I |
| COC1=C(C#N)C(C2=CC=CO2)=C2[C@H](Br)C3=CC=CC=C3C2=N1 | 10 | I |
| CCC1=CC=C(C=C1)N1CCN([C@H](C)C1=O)C(=O)C1=CC=C(Br)C=C1 | 10 | I |
| C[C@H](N1C=C(C=C(Cl)C1=O)C(F)(F)F)C(=O)N1[C@@H](C)CC2=CC=CC=C12 | 10 | I |
| FC1=C([C@H]2[C@@H]3N=C4C=CC=C[C@H]4[C@@H]3CCN2CC2N=CC=N2)C(Cl)=CC=C1 | 10 | I |
| FC1=CC=C2OC3=C([C@H](N(C3=O)C3=CC=CC=C3)C3=CC=CC=C3)C(=O)C2=C1 | 10 | I |
| CC1=CC=CC2=NC(COC3=CC=CC=C3I)=CC(=O)N12 | 10 | I |
| CN1C(=O)N(C)C2=C1C=C(Br)C(=C2)[C@H](Cl)C1=C(C)OC=C1 | 10 | I |
| CC1=CC=C(C=C1)S(=O)(=O)\N=C1/[C@H](Cl)[C@](C)(Cl)C(=O)C(C)=C1Cl | 10 | I |
| FC1=CC(=CC(F)=C1)S(=O)(=O)N1CCO[C@H](C1)C1=CC=C(Cl)C=C1 | 10 | I |
| C[C@@]12O[C@@](C)(C=C1)[C@@H]1[C@@H]2C(=O)N(C1=O)C1=CC=C(Cl)C(=C1)C(F)(F)F | 10 | I |
| ClC1=CC(Cl)=C(C=C1)N1C(=S)S[C@@H](CN2CCOCC2)C1=O | 10 | I |
| CCC1(C)CCN(CC1)S(=O)(=O)C1=CC(Br)=CN=C1Cl | 10 | I |
| C[C@H]1CN(C(=O)C1)S(=O)(=O)C1=C(Cl)C=C(Br)C=C1Cl | 10 | I |
| CC[C@@]1(C)CC2=C3C(S[C@H]2CO1)=NC(=S)N(C3=O)C1=CC=C(C)C=C1 | 10 | I |
| CC12CN3CC(C)(CN(C1)C3C1=CC=CC=C1)C2\N=C\C1=C[C@H]2N=CC=C2C=C1 | 10 | I |
| CN1C(C)=C(C2N3CC4(C)CN2CC(C3)(C2=CC=CC=C2)C4=O)C2=CC=CC=C12 | 10 | I |
| CN1N=C(C)C(CN2CCO[C@H](C2)C2=CC=C(Br)C=C2)=C1Cl | 10 | I |
| ClC1=CC=C(C=C1)C1=CC=C(S1)\C=C\C(=O)N1CCCS1(=O)=O | 10 | I |
| BrC1=CC=C(C=C1)N1C(=O)C2=C(C=CS2)N=C1SCC#N | 10 | I |
| C[C@H]1CN(C[C@@H](C)O1)S(=O)(=O)C1=CC=C(Cl)C(CCl)=C1Cl | 10 | I |
| C[C@H]1CN(C[C@@H](C)O1)S(=O)(=O)C1=CC(Cl)=CC(CCl)=C1Cl | 10 | I |
| C[C@H]1CN(CCN1C)S(=O)(=O)C1=CC(CCl)=C(Cl)C=C1Cl | 10 | I |
| C[C@H]1CN2CCC[C@@H]2CN1S(=O)(=O)C1=CC=C(Br)C=C1Cl | 10 | I |
| BrCC1(CN2C=NC3=CC=CC=C3S2(=O)=O)CCCCC1 | 10 | I |
| CN1N=CC=C1CN1C(CCl)=NC2=CC(I)=C(F)C=C12 | 10 | I |
| CN1CCO[C@H](C1)C1=NC(=C(I)C(Cl)=N1)C(C)(C)C | 10 | I |
| C[C@@H](N1C(CCl)=NC2=C1N(C)N=C2C)C1=CC=CC(Br)=C1 | 10 | I |
| CCN1N=C(C)C2=C1N(C(CCl)=N2)C1=CC=C(Br)C=C1F | 10 | I |
| FC(F)(F)C1=CC(=CC=C1)N1C(=O)\C(SC1=C(C#N)C#N)=C/C1=CSC=C1 | 10 | I |
| FC1=CC=C(C=C1)\N=C1/[C@@H](Cl)C(=O)\C(=N\C2=CC=C(F)C=C2)[C@@H](Cl)C1=O | 10 | I |
| O=S(=O)(N1CCS[C@H](CC1)C1=CC=CO1)C1=CC2=CC=CC=C2C=C1 | 10 | I |
| CC1CCC2(CC1)[C@H](C#N)C(=O)N1CCSC1=C2C#N | 10 | I |
| CC1=CC=C(C=C1)N1[C@H]2CS(=O)(=O)C[C@@H]2SC1=O | 10 | I |
| O=S1(=O)CC[C@@H](C1)SC1=NN=CC2=CC=CC=C12 | 10 | I |
| CCN1C(=O)N(C)C2=CC=C(Br)C=C2C1=O | 10 | I |
| CC1=CC=C2N(CCCC2=C1)C(=O)CN1CSCC1=O | 10 | I |
| O=C1S[C@H]2[C@@H]3[C@@H](CSC2=N1)C(=O)OC1=CC=CC=C31 | 10 | I |
| FC1=CC2=C(C=C1)N=C1C2=N[C@H]2SCCCN2C1=O | 10 | I |
| BrC1=CN=C(N=C1)N1CCN(CC#C)CC1 | 10 | I |
| CC1=C(OC2=C1C(=O)CCC2)C(=O)N1CCSCC1 | 10 | I |
| C[C@H]1CN(C[C@@H](C)O1)C(=O)C1=CC=C(N(C)C)C(F)=C1 | 10 | I |
| C[C@@H]1CN(C[C@@H](C)O1)C(=O)C1=CC=C(N(C)C)C(F)=C1 | 10 | I |
| CCN1CCCN(C)CCN(C)CCCN(CC)CC1 | 10 | I |
| ClCC1=CC(Cl)=C(N=C1)N1CCN2C=CN=C2C1 | 10 | I |
| CN(C(=O)[C@H]1[C@H]2C[C@H]3OC(=O)[C@@H]1[C@H]3C2)C1=CC=C(C)C=C1 | 10 | I |
| CC[C@H]1CCCCN1C(=O)[C@@H]1CS[C@@]2(C)CCC(=O)N12 | 10 | I |
| CN1C=C(C=N1)C1=NN=C(I)S1 | 10 | I |
| C[C@H]1CN([C@H](C)CO1)S(=O)(=O)C1=C(F)C=CC=C1F | 10 | I |
| CS(=O)(=O)N1C=CC2=C1N=CC(Br)=C2 | 10 | I |
| CN1C[C@H](CC1=O)C(=O)N1CCC[C@H]1C1=CC=C(F)C=C1 | 10 | I |
| CC1=CC=C(C=C1)S(=O)(=O)N1C=CC2=C1N=CC(F)=C2 | 10 | I |
| IC1=CN=CN=C1O[C@@H]1CCOC1 | 10 | I |
| C[C@@H]1CC2=CC(=CC=C2O1)C(=O)[C@H]1CCS(=O)(=O)C1 | 10 | I |
| CC1=NN=C(O1)SC1=NC(Br)=CS1 | 10 | I |
| BrC1=CN2C=CN=C2C(SC2COC2)=N1 | 10 | I |
| FC1=CC(Br)=CC2=C1N=CN(CC#N)C2=O | 10 | I |
| C[C@@H]1COCCN1CC1=C(Br)C(C)=NN1C | 10 | I |
| CN1N=C(C)C(Br)=C1CN1C(C)=CC=CC1=O | 10 | I |
| CN1N=C(C)C(Br)=C1CN1CCC[C@H](C1)C#N | 10 | I |
| CC(C)C1=NC(C)=C(S1)C(=O)N1CCN(C)C[C@@H]1C | 10 | I |
| C[C@@H]1CCN([C@@H]1C)C(=O)C1=CN=C(S1)[C@@H]1CCCO1 | 10 | I |
| CC1=NC=C(Br)C(=O)N1C[C@@H]1CCCCO1 | 10 | I |
| CS(=O)(=O)[C@H]1CSCCN1[C@@H]1CCCC[C@@H]1C#N | 10 | I |
| CN1[C@@H]2CC[C@H]1CN(CC2)C1=C(CCl)C(C)=NN1C | 10 | I |
| ClC1=CC(=CC2=C1OCCO2)C(=O)[C@H]1CCC(=O)C1 | 10 | I |
| CSC1(CCN(CC1)[C@H]1CCO[C@@]2(CCOC2)C1)C#N | 10 | I |
| CC(=O)C1=C(C)N(N=C1C)[C@@H]1CCO[C@]2(CCSC2)C1 | 10 | I |
| CC1=NN([C@H]2CCO[C@]3(CCOC3)C2)C(C)=C1CCl | 10 | I |
| CC1=C(C)C=C(CN2CC(=O)N3CCCC[C@H]3C2=O)S1 | 10 | I |
| C[C@H]1N(C2C(C)(C)C2(C)C)C(=O)[C@H]2CCCCN2C1=O | 10 | I |
| CC[C@@H]1N([C@@H]2CCC[C@H](C)C2)C(=O)[C@@H]2CCCN2C1=O | 10 | I |
| CSC1=CC=C(C=C1)N1[C@@H](C)C(=O)N2CCC[C@H]2C1=O | 10 | I |
| CCC1CCC(CC1)N1[C@@H](C)C(=O)N2CCC[C@H]2C1=O | 10 | I |
| C[C@@H](N1CC(=O)N2CCC[C@@H]2C1=O)C1=C(C)SC(C)=C1 | 10 | I |
| C[C@@H](N1CC(=O)N2CCC[C@H]2C1=O)C1=C(C)SC(C)=C1 | 10 | I |
| C[C@H]1N(C(=O)[C@H]2CCCN2C1=O)C1=CC=C(F)C(C)=C1 | 10 | I |
| CC1CCC(CN2CCC(=O)N3CCC[C@H]3C2=O)CC1 | 10 | I |
| CC[C@@H]1N([C@@H]2CC[C@H](C)[C@@H]2C)C(=O)[C@H]2CCCN2C1=O | 10 | I |
| CC[C@@H]1CC[C@H]([C@@H]1C)N1CCC(=O)N2CCC[C@@H]2C1=O | 10 | I |
| CC1=CC(CN2CC(=O)N3CCC[C@@H]3C2=O)=CC(C)=C1F | 10 | I |
| CN1C=CC=C1[C@H]1COCCN1C(=O)C1=CSC=C1C | 10 | I |
| O=C1C(=O)C2=C1OC1=CC=CC=C1C21SCCCS1 | 10 | I |
| CCN1C(=S)S\C(=C\C2=CC3=CC=CC=C3O[C@H]2C)C1=O | 10 | I |
| CC1=C(CN2[C@H]3CC[C@@H]2C2=CN=C(C)N=C2C3)SC=C1 | 10 | I |
| C[C@H](Br)C(=O)C1=CC=C2OCCCOC2=C1 | 10 | I |
| CSC1=N[P@@](=S)(SC)N2CCCC2=C1C#N | 10 | I |
| C[C@H]1SC2=NC(=S)N(C3=CC=C(F)C=C3)C(=O)C2=C1C | 10 | I |
| FC1=CC=C(C=C1)N1C(=O)S\C(=C/C2=CC=CC=C2F)C1=O | 10 | I |
| C[C@H]1CN(C[C@@H](C)O1)C(=O)C1=CC=C(F)C(=C1)C(F)(F)F | 10 | I |
| CC1=NN(CN2CC3C[C@@H]4C[C@H](C3)CC2C4)C(=S)S1 | 10 | I |
| CC(C)C(=O)C1=CC2=C(OCCO2)C=C1Br | 10 | I |
| Br[C@H](C1CCOCC1)C1=CC2=C(OCCO2)C=C1 | 10 | I |
| CC(=O)C1=CC=C(N2C=NC(C)=C2C)C(Br)=C1 | 10 | I |
| CC1=NN(CC2=CC=C(Br)C=C2F)C(=O)C=C1 | 10 | I |
| CCC1=CSC(=N1)N1N=C(C)C(Br)=C1C | 10 | I |
| C[C@H]1CN(CCCO1)C1=C(CCl)C=C(Br)C=N1 | 10 | I |
| CN1C=C(C=O)C(=N1)C1=CC=CC=C1I | 10 | I |
| CC1=C(C)C2=C(S1)N=CN=C2[C@@H](CBr)C#N | 10 | I |
| C[C@H]1CSCCN1CC1=NC2=C(C=CS2)C(Cl)=N1 | 10 | I |
| ClCC1=CN=NN1C1=CC=C(I)C=C1 | 10 | I |
| Br[C@@H]1CCCN(C1=O)C1=CC=C(CC#N)C=C1 | 10 | I |
| IC1=CN(N=C1)[C@H]1CCC[C@@H]1C#N | 10 | I |
| CO[C@@H]1CCC[C@@H](C1)N1C=C(I)C=N1 | 10 | I |
| CC1=NN=C(Cl)N1C1=CC=C(Br)C=C1F | 10 | I |
| CN1CCC2=C(C1)C1=CC=CC=C1N2\C=C/C1=CC=CN=C1 | 10 | I |
| O=S(=O)([C@@H]1CCCC[C@H]1C#N)C1=CC2=C(CCC2)C=C1 | 10 | I |
| FC1=C(F)C=C(C=C1)S(=O)(=O)[C@@H]1CCCC[C@@H]1C#N | 10 | I |
| CC1=C(Br)C(Cl)=NC(CC2=CC=NC=C2)=N1 | 10 | I |
| ClC1=NC(CC2=CC=NC=C2)=NC=C1Br | 10 | I |
| ClC1=NC=C(Br)C(=N1)N1C[C@H]2CC[C@@H]1C2 | 10 | I |
| CCN1C(=NC2=C1N=CC(Br)=C2)[C@H](C)Cl | 10 | I |
| CC[C@H]1CO[C@@H](C)CN1C1=NC(Br)=CS1 | 10 | I |
| CCN1C(=O)C2=CC=CC(I)=C2C1=O | 10 | I |
| CC1=NN=C(Cl)N1CC1=C(Br)C=CS1 | 10 | I |
| C[C@@H](Cl)C1=C(C)N=C(N=C1)[C@H]1CCOC2=C1C=CC=C2 | 10 | I |
| CC#CCN1C=NC2=C(C=C(Br)C=C2F)C1=O | 10 | I |
| CN1N=CN=C1C[C@H]1CCC2=CC=CC=C2[C@@H]1Br | 10 | I |
| CN1N=C(C)C(Br)=C1CN1CCCC1(C)C | 10 | I |
| CC1CCC(CC1)OC1=C(I)C=NC=N1 | 10 | I |
| C[C@@H]1[C@@H](C)S(=O)(=O)CCN1CC1CCC(C)(C)CC1 | 10 | I |
| IC1=CC2=C(C=NN2[C@@H]2CCOC2)C=C1 | 10 | I |
| C[C@H](Cl)C1=CN(N=C1)[C@@H]1CCOC2(CCSCC2)C1 | 10 | I |
| CC[C@@H]1CN2CCCC[C@@H]2CN1[C@@H]1CCN2CCCC[C@H]12 | 10 | I |
| C[C@@H]1C[C@H](C)[C@H](CN2C[C@@H]3CCCCN3C[C@@H]2C)C(=O)C1 | 10 | I |
| C[C@@H](C#N)N(C)C(=O)C1=CC(C)=C(Br)S1 | 10 | I |
| ClC1=C(C=O)C=CC(=C1)N1C=C(Br)C=N1 | 10 | I |
| BrC[C@@H]1CN2C(S1)=NN=C2C1=CC=CC=C1 | 10 | I |
| CC1=NC=CN1CC1=CC=C(Br)C2=C1N=CC=C2 | 10 | I |
| ClC1=CC(CN2CCCS(=O)(=O)CC2)=C(Cl)C=C1 | 10 | I |
| BrC1=CC=CC=C1CN1C(=O)[C@H]2CC=CC[C@@H]2C1=O | 10 | I |
| FC1=CC(OC2=C(Br)C=NC=N2)=CC(F)=C1 | 10 | I |
| C[C@@]12CCC(=O)C[C@@H]1CCC1=C2CC[C@@H]2[C@@H]1CCC2=O | 10 | I |
| C[C@@]12CC[C@H]3[C@H](CCC4=CC(=O)C=C[C@@]34C)[C@@H]1CCC2=O | 10 | I |
| ClC1=NC=C(I)C(Cl)=N1 | 10 | I |
| Br[C@@H]1C=C(C#N)[C@H]2C[C@@H]1C(=C[C@H]2Br)C#N | 10 | I |
| CC(C)(C)S(=O)(=O)C1=C(Br)SC=C1 | 10 | I |
| C[C@@H]1C\C(CC(C)(C)C1)=N/N1C(=O)CSC1=S | 10 | I |
| CC1=N\C(=C\C2=CC=C(Br)S2)C(=O)O1 | 10 | I |
| C[C@]12CC=C3[C@H](CCC4=CC(=O)CC[C@]34C)[C@@H]1CCC2=O | 10 | I |
| ClCC1=CC2=C(OCCCO2)C=C1Br | 10 | I |
| CC1=NN2C3=C(CCC3)C(C)=NC2=C1Br | 10 | I |
| C[C@H]1CC[C@H](C#N)[C@@H](C1)N1C=C(Br)C=N1 | 10 | I |
| CN1CCN(CC2=CC=C(Br)C=C2)CC1 | 10 | I |
| IC1=CC=CC=C1C1OCCCO1 | 10 | I |
| CC(=O)[C@@H]1C[C@@H]1C1=CC(Br)=C(Br)O1 | 10 | I |
| CC(=O)[C@H]1C[C@H]1C1=CC(Br)=C(Br)O1 | 10 | I |
| CC1=NN(CCBr)C(C)=C1Br | 10 | I |
| Br[C@H]1CCCC2=CC3=C(OCCO3)C=C12 | 10 | I |
| CC1=NN(C(=O)C1)C1=C(F)C(F)=C(F)C(F)=C1F | 10 | I |
| CN(C)C1=C(Br)C=C(C=N1)C(F)(F)F | 10 | I |
| BrCC1=CC2=C(OCCO2)C=C1Br | 10 | I |
| ClC1=CC2=C(OCCCO2)C=C1CBr | 10 | I |
| C[C@H]1CN(C[C@H](C)S1)C1=NC(CCl)=CS1 | 10 | I |
| C[C@@H]1CC[C@H]([C@H](C)C1)N1CCC[C@H](Br)C1=O | 10 | I |
| CC1=C(SC=N1)C1=NN=C(Br)S1 | 10 | I |
| C[S@@](=O)C1=CN=C2C=C(Br)C=CC2=N1 | 10 | I |
| ClC1=NC(=CC=N1)C1=NC(Br)=CC=C1 | 10 | I |
| ClC1=CC=CC(=N1)C1=CC=NC(Br)=N1 | 10 | I |
| FC(F)S(=O)(=O)C1=CC(Br)=CC=C1 | 10 | I |
| FC1=NC(F)=C(F)C(SC2=NC=CS2)=C1F | 10 | I |
| C[C@@H]1CCC[C@H](C1)[S@@](=O)[C@@H]1C[C@H](C)CC[C@@H]1C#N | 10 | I |
| CCN1N=C(C)C(Br)=C1CI | 10 | I |
| FC(F)C(F)(F)C1=NN=C(Br)S1 | 10 | I |
| C[C@H]1CN(CC2=CN=C(Cl)S2)C[C@H](C)S1 | 10 | I |
| BrN1CCC(=O)C1(Br)Br | 10 | I |
| CC1=C(Cl)N=C(N=C1Cl)[C@H]1CSCCO1 | 10 | I |
| BrC1=CSC=C1C(=O)[C@@H]1CSCCO1 | 10 | I |
| BrC1C[C@@H]2CC[C@H](C1)N2CC1CCOCC1 | 10 | I |
| CCC1=NN(C)C(CBr)=C1Br | 10 | I |
| BrC[C@@H]1C[C@H]1C1=CC2=C(OCCCO2)C=C1 | 10 | I |
| FC1=CC=C(C=C1)[C@@H](Br)[C@H]1CCS(=O)(=O)C1 | 10 | I |
| FC1=CC(CCl)=CC(F)=C1N1C[C@@H]2CC[C@H](C1)O2 | 10 | I |
| CC1(C)CC(=O)C=C(C1)[C@H]1CCO[C@]2(CCOC2)C1 | 10 | I |
| CC(=O)C1=CC(Br)=CC(Br)=N1 | 10 | I |
| BrC1=CCC2=C(I)N=NC2=C1 | 10 | I |
| Br[C@H]1C=NC2=NC=C(I)C2=C1 | 10 | I |
| Br[C@H]1CC(OC2CC2)=CC(Br)=N1 | 10 | I |
| BrC1=C2CC=C(I)C=C2N=N1 | 10 | I |
| ClC1=CN2C=NC(I)=C2C=C1 | 10 | I |
| ICC(=O)N1CC2CCC(CC2)C1 | 10 | I |
| FC(F)(F)C1=N[C@H]2C(C=C(Cl)C(Cl)=C2Cl)=N1 | 10 | I |
| BrCC(=O)C1=CC2=C(OCCCO2)C=C1 | 10 | I |
| ClC1=CC=C(S1)\C=N\N1C(=O)CSC1=S | 10 | I |
| C[C@H](Br)C(=O)C1=CC=C2OCCOC2=C1 | 10 | I |
| COCC1=C(C#N)C(Cl)=NC(C)=C1Br | 10 | I |
| CS(=O)(=O)C(=C\C1=CC=C(Cl)C=C1Cl)\C#N | 10 | I |
| CS(=O)(=O)C(=C\C1=CC=CC(Cl)=C1Cl)\C#N | 10 | I |
| O=S1(=O)CC[C@H](C1)\N=C\C1=CC=CC2=CC=CC=C12 | 10 | I |
| CN1C(=O)C2=C(SC3=C2CCC3)N=C1SCC#C | 10 | I |
| CN1C=C(C=O)C(=N1)C1=CC=C(Br)S1 | 10 | I |
| ClC1=NC=C(CN2C=C(Br)C=N2)C=C1 | 10 | I |
| O=C1[C@@H]2[C@H]3C[C@H](C=C3)[C@@H]2S(=O)(=O)[C@H]2[C@H]3C[C@H](C=C3)[C@@H]12 | 10 | I |
| C[C@H](C#N)N(C)C(=O)C1=CC=C(Br)S1 | 10 | I |
| C[C@@H]1CN([C@H](C)CO1)C(=O)\C=C(\C)C1=CC=C(F)C=C1 | 10 | I |
| C[C@@H]1CN([C@@H](C)CO1)C(=O)\C=C(\C)C1=CC=C(F)C=C1 | 10 | I |
| FC1=C(C=CC(Br)=C1)C1=NN=C(O1)C1CC1 | 10 | I |
| BrC1=CC=C(CN2N=CC=CC2=O)S1 | 10 | I |
| C[C@H]1CSCCN1C1=NC=C(Br)C=N1 | 10 | I |
| IC1=CN(CC2=CC=CO2)N=C1 | 10 | I |
| CC1=NN=C(Cl)N1C1=CC=C(Br)C=C1 | 10 | I |
| BrC1=NN(CC2CC2)C(Br)=N1 | 10 | I |
| CSC1=C(SC=C1)C(=O)N1[C@H](C)COC[C@H]1C | 10 | I |
| C[C@H](C#N)N(C)C(=O)C1=C(Br)C=CS1 | 10 | I |
| COC[C@H]1CN(C)[C@@H]2CC3=CN(C)C4=C3C(=CC=C4)C2=C1 | 10 | I |
| C(N1CCC2(CC1)OCCO2)C1=CC2=C(CCC2)C=C1 | 10 | I |
| Cl[C@H]([C@@H]1CCS(=O)(=O)C1)C1=CC2=C(COC2)C=C1 | 10 | I |
| CN1C=C(SC2=NC(Br)=CS2)C=N1 | 10 | I |
| CC1=NSC(=N1)N1CCCC[C@H]1CBr | 10 | I |
| CC(C)(C)S(=O)(=O)N1CCC[C@H](Br)C1 | 10 | I |
| CCC1=NSC(=N1)N1CCC[C@H](Br)C1 | 10 | I |
| FCCN1C(=O)C(=O)C2=C1C=CC(Br)=C2 | 10 | I |
| O=S1(=O)CC[C@H](C1)[C@]1(CCCC2=CC=CC=C12)C#N | 10 | I |
| CN1CCN(CC1)[C@@]1(CCC[C@@H](C1)C(F)(F)F)C#N | 10 | I |
| CN1CCN(CC1)C1(CCC(CC1)C(F)(F)F)C#N | 10 | I |
| CC1=C(C)N=C(S1)N1CCO[C@@H](CBr)C1 | 10 | I |
| ClC1=CN2C(=O)C=C(CBr)N=C2C=C1 | 10 | I |
| CC1(C)CN(CC[S@@]1=O)C(=O)CC1CCCCC1 | 10 | I |
| C\C(=C/C(=O)N1CC[S@@](=O)C(C)(C)C1)C(C)(C)C | 10 | I |
| CC1(C)CC(=O)N(CC2=CC=C(Br)C=C2)C1=O | 10 | I |
| C[C@H]1CCC[C@@H](C)N1C(=O)CN1CCCSCC1 | 10 | I |
| C[C@@H](N1CCCSCC1)C(=O)N1CCCCCC1 | 10 | I |
| FC1=C(Cl)C=CC(CN2CCC3(C2)OCCO3)=C1 | 10 | I |
| C[C@H]1[C@@H](C)S(=O)(=O)CCN1CC1=C(C)C=CS1 | 10 | I |
| C(N1CCOC2(CCC2)C1)C1=CC2=C(C=CS2)N=C1 | 10 | I |
| C[C@@H]1CN(C)C2=CC=CC=C2CN1CC1=CCCOC1 | 10 | I |
| CC1=NN([C@@H]2CCO[C@]3(CCSC3)C2)C(C)=C1CCl | 10 | I |
| CC[C@@H]1CN2CCCC[C@@H]2CN1[C@@H]1CN2CCC1CC2 | 10 | I |
| BrC1=CN2N=C(Br)C=CC2=N1 | 10 | I |
| CS(=O)(=O)C(=C/C1=CC=CC(Cl)=C1Cl)\C#N | 10 | I |
| CC[C@@H]1CN(CCO1)C1=CC(Br)=CC=N1 | 10 | I |
| CC1=CC(Br)=C(OC2CCOCC2)N=C1 | 10 | I |
| C[C@]12CCC(=O)C[C@@H]1CCC1=C2CC[C@@H]2[C@@H]1CCC2=O | 10 | I |
| CC1=N\C(=C/C2=CC=CC=C2Br)C(=O)O1 | 10 | I |
| CC1=NC=CN1C1=CC=C(I)C=C1 | 10 | I |
| COC1=CC(Br)=CN=C1I | 10 | I |
| C[C@@H]1C=C[C@@H](C)[C@H]2[C@H]1C(=O)[C@H]1[C@H](C)C=C[C@@H](C)[C@@H]1C2=O | 10 | I |
| ClC[C@@H]1CO[C@H](O1)C1=CC(Br)=CC=C1 | 10 | I |
| C[C@H](Br)C(=O)C1=CC=C2O[C@H](C)CC2=C1 | 10 | I |
| BrC1=CC2=C(O[C@H](C2)[C@H]2C=CN=N2)C=C1 | 10 | I |
| FC(F)(F)[C@H](Br)C1=CC2=C(OCCO2)C=C1 | 10 | I |
| BrC1=CSC(CN2C=CC=CC2=O)=C1 | 10 | I |
| CC1(C)[C@H]2CC[C@]1(C)[C@H](SC1=NCCS1)C2=O | 10 | I |
| CC1=CC=C(C=C1C)[C@H](Br)[C@@H]1COCCO1 | 10 | I |
| CC1=CC=C(C=C1C)[C@@H](Br)[C@@H]1COCCO1 | 10 | I |
| CC1=CC(=O)N(CC2=CC=CC=C2Br)C=C1 | 10 | I |
| FC1=C(C=CC(Br)=C1)[C@@H]1CC(=O)C=CO1 | 10 | I |
| BrC1=CC2=C(C[C@@]3(CC2)CC(=O)C=CO3)C=C1 | 10 | I |
| FC1=C(Br)C=C(C=C1)[C@H]1OCC[C@@H]1C#N | 10 | I |
| BrC1=CSC(=C1)[C@@H]1OCCC[C@@H]1C#N | 10 | I |
| FC1=C(Br)C=C(CN2C=CSC2=O)C=C1 | 10 | I |
| CCN(C)C(=O)C1=CC=C(I)C=C1Cl | 10 | I |
| C[C@@H]1COC[C@@H](C)N1CC1=CC=CC=C1Br | 10 | I |
| BrC1=C(CN2CCC[C@H](C2)C#N)SC=C1 | 10 | I |
| CC(C)N(C)C(=O)C1=CSC(I)=C1 | 10 | I |
| CC[C@@H](Br)C(=O)N1C[C@H](C)CC[C@@H]1C | 10 | I |
| C[C@@H]1C[C@@H](C)CC(C1)N1CC[C@H](Br)C1=O | 10 | I |
| BrC[C@]1(C[C@@H]2CC[C@H]1C2)[C@@H]1CCS(=O)(=O)C1 | 10 | I |
| BrCC1(CCCCC1)[C@H]1CCS(=O)(=O)C1 | 10 | I |
| C[C@H](Br)CC1=C(I)C=NC=N1 | 10 | I |
| IC1=CC=C(O[C@H]2CCC(=O)C2)C=C1 | 10 | I |
| CCCN1C=C(Cl)C(I)=N1 | 10 | I |
| CC1=CC(=O)N(CC(F)F)C=C1I | 10 | I |
| CCC1=C(C)N=C(N=C1Cl)[C@H]1CSCCS1 | 10 | I |
| BrC1=CN=C(SC2=CC=NC=C2)C=C1 | 10 | I |
| C[C@@H](Br)[C@H](C)[C@H]1CCO[C@]2(CCOC2)C1 | 10 | I |
| BrC1=CSC=C1C(=O)C1=CSC=N1 | 10 | I |
| Br[C@H]1CCCN(C[C@@H]2CCCCS2)C1=O | 10 | I |
| BrC1=CN=C2C=C(Br)C=CN12 | 10 | I |
| FC1=C(CN2CCOCC2)C(F)=C(Br)C=C1 | 10 | I |
| FC1=C(Cl)C=C(CN2CCOC3(CCC3)C2)C=C1 | 10 | I |
| O=C1CCCC[C@H]1[C@H]1CCOC2(CCSCC2)C1 | 10 | I |
| CC1=C(Br)N=CC(I)=N1 | 10 | I |
| C[C@H]1C=C(OC2CC2)C=N[C@H]1I | 10 | I |
| FC1=NC=C(OC2CC2)C(I)=C1 | 10 | I |
| BrC1=CN=CC(Br)=C1OC1CC1 | 10 | I |
| F[C@@H]1C=C2N=NC(I)=C2C(F)=C1 | 10 | I |
| CC(=O)C1=CC(Br)=CN=C1C(F)(F)F | 10 | I |
| FC1=CC(Br)=CN2C(Br)=CN=C12 | 10 | I |
| FC1=CC(Br)=C(Cl)C=C1C(=O)CC#N | 10 | I |
| BrCC1=NC2=C(S1)N=CC(Br)=C2 | 10 | I |
| CC1=C(Br)C(=O)C(Br)=C(Cl)C1=O | 10 | I |
| Br[C@H]1C=C(C#N)[C@]23CC12C(=C[C@H]3Br)C#N | 10 | I |
| C=CCN1C(=S)S\C(=C2\CC3=CC=CC=C3S2)C1=O | 10 | I |
| S=C1SC2=C(S1)S[C@H]1OCCO[C@@H]1S2 | 10 | I |
| FC(F)(F)C1=CC=C(C=C1)N1C(=O)CSC1=S | 10 | I |
| CC1=CC(=O)N(C1=O)C1=CC=C(Br)C=C1 | 10 | I |
| C[C@@]1(Br)CCS(=O)(=O)[C@@H]1Br | 10 | I |
| CN(C)C(=O)C1=CC(Br)=C(Br)S1 | 10 | I |
| C[C@]12CC[C@@H]3[C@@H](CCC4=CC(=O)C=C[C@]34C)[C@H]1CCC2=O | 10 | I |
| C[C@]12CC[C@H]3[C@@H](CCC4=CC(=O)C=C[C@@]34C)[C@@H]1CCC2=O | 10 | I |
| CCN1C(=O)C=CC2=CC(CBr)=CC=C12 | 10 | I |
| Br[C@@H]1N=NC(Br)=C1Br | 10 | I |
| CC1=NC=CN1CC1=CC=C(Br)C=C1F | 10 | I |
| C[C@@H]1COCCN1CC1=CC=C(Br)C=C1 | 10 | I |
| C1COC2(CC[C@@H]3C=C[C@H]2CC32SCCS2)O1 | 10 | I |
| Br[C@@H](C1CCCC1)[C@H]1CCS(=O)(=O)C1 | 10 | I |
| CC1=NN([C@@H]2CCC[C@@H]2C#N)C(C)=C1Br | 10 | I |
| CC1=C(C)N(CC2=CC=C(Br)C=C2)C=N1 | 10 | I |
| CC1=CC=C(C=C1)[C@@H](Br)[C@H]1COCCO1 | 10 | I |
| FC1=CC=C(\C=C2\C(=O)OC3=CC=CC=C3C2=O)C=C1 | 10 | I |
| ClC1=NN2C(I)=CN=C2C=C1 | 10 | I |
| C[C@H]1CSCCN1C1=CC=C(Br)C=N1 | 10 | I |
| CC[C@H](C)C1=NN=C(I)S1 | 10 | I |
| FC(F)(F)C1=NC2=C(O1)C=C(Br)C=N2 | 10 | I |
| CN(C)C1=CN=C(Br)C=C1Br | 10 | I |
| COC1=CC(Cl)=C(I)C=N1 | 10 | I |
| FC1=CC(=CC(F)=C1F)N1CC[C@H](Br)C1=O | 10 | I |
| Br[C@H]1CCN(C[C@H]2C[C@H]3CC[C@@H]2C3)C1=O | 10 | I |
| CC(C)C(=O)C1=CC2=CC(F)=CC(Br)=C2O1 | 10 | I |
| CCN1N=C(C)C(Cl)=C1CI | 10 | I |
| CC1(C)C(=O)[C@@]2(Br)CC[C@]1(C=O)[C@@H]2Br | 10 | I |
| CCC1=NC(C)=C(I)C(Cl)=N1 | 10 | I |
| CSCC1=NC=C(I)C(Cl)=N1 | 10 | I |
| C[C@]12CC[C@@H]3[C@H](CC=C4CC(=O)CC[C@]34C)[C@@H]1CCC2=O | 10 | I |
| CC1(C)C(CN2CCC[C@@H](Br)C2=O)C1(C)C | 10 | I |
| CC1=CC(Br)=CN=C1[C@@H](CBr)C#N | 10 | I |
| I[C@H]1CCO[C@@]2(CCOC2)C1 | 10 | I |
| BrC1=CN=CC(=C1)C(=O)C1=CSC=C1 | 10 | I |
| ClCC1=CSC(=N1)[C@@H]1COC2=CC=CC=C2C1 | 10 | I |
| C[C@@H]1CCN([C@H](C)C1)C1=C(Br)C=NC=N1 | 10 | I |
| FC(F)(F)C1=NC=CN=C1I | 10 | I |
| C[C@H]1CN(C[C@@H]2CSC3=C2C=CC=C3)C[C@@H](C)O1 | 10 | I |
| CC1=NN(C[C@@H]2CSC3=C2C=CC=C3)C=C1Cl | 10 | I |
| CC1=CC([C@@H](Cl)[C@@H]2COCCO2)=C(F)C=C1F | 10 | I |
| O=C1CCCC[C@@H]1[C@H]1CCOC2(CCSCC2)C1 | 10 | I |
| CN([C@H]1CCSC1)C1=C(F)C(F)=NC(F)=C1F | 10 | I |
| CCN1C(C)=NC2=C1C(Br)=CC(=C2)C#N | 10 | I |
| Br[C@@H]1C=NC2=NN=C(I)C2=C1 | 10 | I |
| BrC1=CCC2=NN=C(I)C2=C1 | 10 | I |
| IC1=NC=C[C@H](C1)OC1CC1 | 10 | I |
| BrC1=NC=CC(OC2CC2)=C1Br | 10 | I |
| CSC1=NC=NC(SC)=C1Br | 10 | I |
| CC1=C(Br)C=C(Br)C(C)=[N+]1[O-] | 10 | I |
| BrC1=CC2=CC=CC=C2S1(=O)=O | 10 | I |
| C[C@H]1[C@@H]2CCC(C)=C3[C@@H]([C@@H]2OC1=O)C(C)=CC3=O | 10 | I |
| CS(=O)(=O)C1=CC(Br)=CC=C1F | 10 | I |
| [O-][N+]1=CC=CC=C1S[C@H]1[C@H]2CC[C@H](C2)[C@@H]1Cl | 10 | I |
| CN1N=C(I)C2=CC(F)=CC=C12 | 10 | I |
| C[C@H]1[C@H]2CC[C@]3(C)CCC(=O)[C@H](C)[C@@H]3[C@@H]2OC1=O | 10 | I |
| BrCC1=CC=C(C=C1)C1=NC=CC=N1 | 10 | I |
| BrC1=CC=C(C=C1)C1=CCS(=O)(=O)C1 | 10 | I |
| FC(F)(F)C1=NC(Br)=C(C=O)C=C1 | 10 | I |
| Br[C@H](C1CC1)[C@@H]1CCS(=O)(=O)C1 | 10 | I |
| CC(C)=C1CC[C@@H]2CCC3=CC(=O)CC[C@@]3(C1)C2=O | 10 | I |
| CC1=NN(CC(Cl)=C)C(C)=C1Br | 10 | I |
| Cl[C@@H]1COCCC2=CC3=C(OCCCO3)C=C12 | 10 | I |
| BrCCN1CCCN2CCC[C@@H]2C1 | 10 | I |
| C[C@H](Br)CC1=C2C=CSC2=NC=N1 | 10 | I |
| CC1=[O+]C2=C3C(OC4=CC=CC=C34)=CC=C(C)C2=C1 | 10 | I |
| CCN1C=CN=C1[C@H]1CCC[C@H]1Br | 10 | I |
| FC1=CC=C(SC2=C(Cl)C(=O)OC2)C=C1 | 10 | I |
| CCN1C=CN=C1[C@H]1CCC[C@H](Br)C1 | 10 | I |
| C[C@]12O[C@H]1C[C@@H]1[C@@H]2[C@H]2OC(=O)C(=C)[C@@H]2CCC1=C | 10 | I |
| ClC1=NC=C(CBr)C(Cl)=N1 | 10 | I |
| C[C@H]1C[C@@H](SC2=CC=C(F)C(F)=C2)C(=O)O1 | 10 | I |
| CC1(C)CCC(=O)[C@H](CN2CCCSCC2)C1 | 10 | I |
| Br[C@H]1CCN(C[C@H]2CCC=CC2)C1=O | 10 | I |
| CCN1C=CN=C1[C@@H]1CC[C@@H](Br)[C@@H]1C | 10 | I |
| O=[S@]([C@H]1CCC[C@@H]1C#N)C1=CC2=C(CCC2)C=C1 | 10 | I |
| CC1(C)C(=O)[C@@]2(Br)CC[C@]1(CC#N)C2 | 10 | I |
| CSCC1=NC=C(Br)C(Cl)=N1 | 10 | I |
| C[C@@H](Cl)[C@H](C)C1=C(Br)C=NC=N1 | 10 | I |
| C[C@@H]1[C@H]2CC[C@]3(C)C(=O)CCC(C)=C3[C@@H]2OC1=O | 10 | I |
| C[C@H]1[C@H](C)[S@@](=O)C2=CC(F)=C(F)C=C2C1=O | 10 | I |
| C[C@H]1O[C@H](C)[C@@H]([C@@H]1C)C(=O)[C@@H]1CCCCS1 | 10 | I |
| C[C@H](Cl)C1=C(C)N=C(N=C1)[C@@H]1CCCS1 | 10 | I |
| C[C@H]1C[C@@H]1C(=O)N1CCC[C@@H]1CBr | 10 | I |
| BrC1CCN(C[C@H]2CCCO2)CC1 | 10 | I |
| BrC[C@H]1C[C@@]11CCCS(=O)(=O)C1 | 10 | I |
| FC(F)(F)C1=NC=C(Br)C(C=O)=C1 | 10 | I |
| N#C[C@]1(C[C@@H]2CSC3=CC=CC=C23)CCOC1 | 10 | I |
| C[C@H]1CN(C[C@@H]2CSC3=C2C=CC=C3)CCO1 | 10 | I |
| C([C@H]1CSC2=CC=CC=C12)N1C[C@@H]2CC[C@H](C1)O2 | 10 | I |
| C[C@@H]1[C@@H](C)S(=O)(=O)C2=C(C=C(C)C(C)=C2)C1=O | 10 | I |
| C[C@@H]1OCCN(CC2=CC(F)=C(F)C=C2F)[C@H]1C | 10 | I |
| CC(C)N1C=CN=C1[C@H](C)I | 10 | I |
| CC1=NN(CCF)C=C1I | 10 | I |
| CC1=CN=C2N1C=C(Cl)N=C2Br | 10 | I |
| C[C@@]12C[C@@H](N3CCSC3=N1)C1=CC=CC=C1O2 | 10 | I |
| ClC1=CC(=O)C2=C3[C@@H]1C=CC1=C3[C@H](C=C2)C(Cl)=CC1=O | 10 | I |
| CN(C)C1=CC=NC(I)=C1 | 10 | I |
| CC1=NC2=C(C=C(C)C=C2Br)C(=O)O1 | 10 | I |
| BrC1=C(Br)C=C(O1)C=O | 10 | I |
| COC1(OC)[C@@H]2CC[C@@]1(Cl)C(Cl)=C2Cl | 10 | I |
| Cl[C@@]12C[C@H]3C[C@@H]1[C@@H](OC2=O)[C@@H]3I | 10 | I |
| CC1=COC2=C1C[C@H]1O[C@@]1(C)CC[C@H]1O[C@]1(C)C2 | 10 | I |
| C[C@@H]1[C@H]2CCC(C)=C3[C@H]([C@@H]2OC1=O)C(C)=CC3=O | 10 | I |
| CCN1C(=S)S\C(=C\[C@@H]2CCC=CC2)C1=O | 10 | I |
| BrC1=C2OCCOC2=C(S1)C#N | 10 | I |
| CC1=COC2=C1C[C@H]1O[C@]1(C)CC[C@@H]1O[C@]1(C)C2 | 10 | I |
| C[C@@]12O[C@@H]1C[C@@H]1[C@H]2[C@H]2OC(=O)C(=C)[C@@H]2CCC1=C | 10 | I |
| C[C@H]1CC(=O)C2=C(C)CC[C@H]3[C@H](C)C(=O)O[C@@H]3[C@@H]12 | 10 | I |
| CS(=O)(=O)C1=CC=C(Cl)C(=C1)C(F)(F)F | 10 | I |
| [O-][N+]1=CC=CC=C1S[C@H]1[C@H]2CC[C@H](C2)[C@H]1Cl | 10 | I |
| CN1C=C2C[C@@H]3[C@H](OCCN3C)C3=CC=CC1=C23 | 10 | I |
| C[C@@H]1[C@@H]2CC[C@@]3(C)O[C@]33CC=C(C)[C@@H]3[C@H]2OC1=O | 10 | I |
| C[C@H]1[C@H]2[C@H](OC1=O)[C@@H]1C(C)=CC(=O)C1=C(C)C[C@@H]2C | 10 | I |
| IC1=CC=CC=C1OCC#N | 10 | I |
| C1COC2(CC[C@H]3C=C[C@@H]2CC32SCCS2)O1 | 10 | I |
| CN(C)C(=O)[C@]12CC[C@](C)([C@H]1Br)C2(C)C | 10 | I |
| CC1=NN(CC(F)(F)F)C(C)=C1Br | 10 | I |
| C[C@@H]1CC(=O)C2=C(C)CC[C@H]3[C@H](C)C(=O)O[C@@H]3[C@H]12 | 10 | I |
| I[C@@H]1C[C@@H](C=C)[C@H]2CC(=O)O[C@@H]12 | 10 | I |
| ClC1=CC(=CN2C(Br)=CN=C12)C#N | 10 | I |
| BrC1=NC=C(C=C1)[C@H]1CCCOC1 | 10 | I |
| O=C1C[C@@H]2[C@@H]3CC(=O)[C@@H]4[C@@H]5CC[C@@H]6C5C([C@H]34)[C@@H]2[C@@H]16 | 10 | I |
| BrC1=CC=C(CN2CCS(=O)CC2)C=C1 | 10 | I |
| BrC1=CC(=O)C(Br)=CC1=O | 10 | I |
| C[C@@H]1CCC(=O)[C@H](CN2CCCSCC2)C1 | 10 | I |
| Br[C@H]1[C@@H]2[C@@H]3CC(=O)[C@H]4[C@@H]3C[C@@H]2[C@H]4C1=O | 10 | I |
| CCN1C=C(C=N1)[C@@H]1CCC[C@@H]1Br | 10 | I |
| CCC(C)(C)N1CCC[C@H](Br)C1=O | 10 | I |
| C[C@@H]1CCC(=O)[C@H](CN2C[C@H](C)S[C@H](C)C2)C1 | 10 | I |
| CN1C=C(C[C@H]2CCC[C@H]2Br)C=N1 | 10 | I |
| CCN1N=C(C)C=C1CI | 10 | I |
| CO[C@@H](C#N)C1=CC=C(F)C(Br)=C1 | 10 | I |
| ICC1=CN=CN1C1CC1 | 10 | I |
| CC1(C)C(=O)[C@]2(Br)CC[C@]1(C2)C=O | 10 | I |
| CC[C@H](Br)[C@H]1CCCS(=O)(=O)C1 | 10 | I |
| CN1C=CN=C1C[C@H]1CCC[C@@H](Br)C1 | 10 | I |
| CC(=O)C1=CSC(=N1)[C@H]1CSCCS1 | 10 | I |
| C[C@@H](Cl)C1=C(C)N=C(N=C1)[C@H]1CSCCS1 | 10 | I |
| BrC1CCN(CC2=NC=CS2)CC1 | 10 | I |
| C[C@H](I)C1=NC=CN1C1CC1 | 10 | I |
| CCN1C=CN=C1[C@@H](C)I | 10 | I |
| CN1C(Br)=CC2=C1N=CN=C2Cl | 10 | I |
| IC1=CCC2=NN=CC2=C1 | 10 | I |
| IC1=NN=C2CC=CN=C12 | 10 | I |
| CN1C=C(Br)C2=C1C(Cl)=NC=N2 | 10 | I |
| FC(F)(F)C1=NC(=NC=C1Br)C#C | 10 | I |
| IC1=CC=C(CN=O)C=C1 | 10 | I |
| C[C@@]12CC(=O)C[C@H]1[C@H]1CC[C@H]3CC(=O)CC[C@]3(C)[C@@H]1CC2 | 10 | I |
| BrC1=CC2=C(C=CC=N2)N=C1Br | 10 | I |
| C\C1=C\C=C/C(=O)C#CC(=O)\C=C/C=C(C)\C#CC#C1 | 10 | I |
| BrCC1=CSC2=NC3=CC=CC=C3N12 | 10 | I |
| BrC1=CC=C(C=C1)C1=CN2C=CN=C2S1 | 10 | I |
| C[C@@]12CC[C@@H]3[C@H](CC[C@H]4CCC(=O)C[C@]34C)[C@@H]1CCC2=O | 10 | I |
| C[C@]12CC[C@@H]3[C@H](CC[C@H]4CCC(=O)C[C@]34C)[C@@H]1CCC2=O | 10 | I |
| ClC1=CC(Cl)=C(C=C1)C1=CN2C=CSC2=N1 | 10 | I |
| C1CC2=C(N=C3C=C[C@@H]4C=CC=C[C@@H]4C3=C2C1)C1=CC=CO1 | 10 | I |
| C1CC2=C(N=C3C=C[C@@H]4C=CC=C[C@H]4C3=C2C1)C1=CC=CO1 | 10 | I |
| CC1=NC2=CC=C(Br)C=C2C(Cl)=N1 | 10 | I |
| CC1=CN=C(Cl)N1C1=CC=C(Cl)C(Cl)=C1 | 10 | I |
| ClC1=CC=C(N=N1)C1=CC(Br)=CC=C1 | 10 | I |
| BrC1=CC=C(C=C1)C1=CN2CCCCCC2=N1 | 10 | I |
| ClC1=NC(=NC=C1)C1=CC=C(Br)C=C1 | 10 | I |
| CC1=CC(C)=C2C(C)=C(Br)C(=O)OC2=C1 | 10 | I |
| CC1(C)O[C@H]2[C@@H](O1)C1C3=CC=CC=C3C2C2=C1C=CC=C2 | 10 | I |
| Cl[C@@H]1CCCC2=C(Cl)C=C3OCCCOC3=C12 | 10 | I |
| C[C@H]1CC(C)(C)O[C@H](O1)C1=CC=C(Br)C=C1 | 10 | I |
| C[C@@H]1CCC[C@@H](C1)N1C=C(I)C=N1 | 10 | I |
| FC1=C(F)C=C(C=C1)C1=CC(Cl)=NC(Cl)=N1 | 10 | I |
| CC1(C)COC(=N1)C1=CC(F)=C(F)C(Cl)=C1F | 10 | I |
| CC1=CSC(=O)N1C1=CC(Cl)=CC(Cl)=C1 | 10 | I |
| BrC1=CC2=C(C=C1)N=CC(Br)=N2 | 10 | I |
| CC1(C)CC2=CC=CC([C@H]3C[C@@H](Cl)CCO3)=C2O1 | 10 | I |
| ClC1=CC=CC(=N1)C1=C(Br)C=CO1 | 10 | I |
| C[C@H]1CC(C)=C[C@@H](C)C11COC(CBr)OC1 | 10 | I |
| C[C@H]1C[C@@H](C)CN(C1)C1=NC(Br)=CS1 | 10 | I |
| C[C@@H]1C[C@@H](C)CN(C1)C1=NC(Br)=CS1 | 10 | I |
| ClC1=NC(=NC2=C1C=CS2)[C@@H]1CC2=CC=CC=C12 | 10 | I |
| CC1=C(Br)SC(=C1)C(=O)N=C=S | 10 | I |
| C[C@H](Cl)C1=NC2=CC(Br)=CC=C2O1 | 10 | I |
| CC1(C)CCN(C1)C1=NC(Br)=CS1 | 10 | I |
| CC1=NC(C2=CC=C(Cl)S2)=C(C)C(Cl)=N1 | 10 | I |
| ClC1=NSN=C1C1=C(Br)C=CS1 | 10 | I |
| C[C@H]1CCC[C@H](C1)C1=NC(Cl)=C(F)C(Cl)=N1 | 10 | I |
| C[C@@H]1CC[C@H](C1)C1=NC2=C(C=C(C)S2)C(Cl)=N1 | 10 | I |
| C[C@@H]1C[C@H]1C1=NC2=C(C3=C(CCC3)S2)C(Cl)=N1 | 10 | I |
| ClC1=NC2=CC=CC=C2N=C1CBr | 10 | I |
| C[C@@H]1[C@@H](C)C(=O)CCN1C12CC3CC(CC(C3)C1)C2 | 10 | I |
| Cl[C@H]1CCCC[C@H]1[C@@H]1CCOC2(CCOCC2)C1 | 10 | I |
| C[C@@H]1CC[C@@H]([C@H](C)C1)N1CCCN2CCCC[C@@H]2C1 | 10 | I |
| C[C@H]1CC[C@H](C[C@@H]1C)N1CCCN2CCCC[C@@H]2C1 | 10 | I |
| CC1=CC(Br)=CC2=C1N=C(Cl)C(C=O)=C2 | 10 | I |
| COC1=CC=C(Br)C2=C1N=C(Cl)S2 | 10 | I |
| ClC1=C(Cl)OC(Cl)(Cl)C(Cl)(Cl)O1 | 10 | I |
| ClC1=CC=C(C=C1)C1=NOC(Br)=C1 | 10 | I |
| CCC1=C2[C@H](CC(=O)C2=O)[C@@H]2CC[C@@H]3CCCC[C@@H]3[C@@H]2C1 | 10 | I |
| CCC1=C2[C@H](CC(=O)C2=O)[C@H]2CC[C@@H]3CCCC[C@@H]3[C@@H]2C1 | 10 | I |
| CC1=CC(Cl)=C(C)C(=C1Cl)S(Cl)(=O)=O | 10 | I |
| BrC1=CC2=C(OCO2)C=C1C=O | 10 | I |
| CCN1C(=O)OC2=CC(Br)=CC=C12 | 10 | I |
| CN(C(C)=O)C1=CC=C(Br)C=C1 | 10 | I |
| CN1C=NN=C1C1=CC=CC=C1Br | 10 | I |
| CC1=CN(N=C1)C1=C(F)C(F)=NC(F)=C1F | 10 | I |
| CN1C(=O)C2=C(C1=O)C(F)=C(F)C(F)=C2F | 10 | I |
| FC(F)(F)[C@@]1(F)C(F)(F)OS1(=O)=O | 10 | I |
| CN(C)C(=O)C1=CC(Br)=CN1C | 10 | I |
| CC1=CC=C(C)N1C1CCS(=O)(=O)CC1 | 10 | I |
| CC1=CN(N=C1)C1=CC(Br)=CC=N1 | 10 | I |
| ClC1=NC=NN2C=C(Br)C=C12 | 10 | I |
| CC1=CC=C(C)N1[C@@]1(C)CCS(=O)(=O)C1 | 10 | I |
| C1CC11CCC23CC[C@@]4(CO4)C12[C@]1(CO1)CC3 | 10 | I |
| CN(CC1=CC=C(Br)C=C1)C=O | 10 | I |
| BrC1=C(OC=C1)[C@@H]1CCC(=O)C1 | 10 | I |
| CN1N=CC=C1[C@H]1CC[C@H](Br)C1 | 10 | I |
| CN1N=CC=C1[C@H]1CC[C@@H](Br)C1 | 10 | I |
| CN1C=C(C=N1)[C@@H]1CC[C@H](Br)C1 | 10 | I |
| BrC1=NN(C=C1)[C@H]1CCCCO1 | 10 | I |
| CC12CN3CC(C)(CN(C1)C3C1=CC=CO1)C2 | 10 | I |
| CN1N=CC(I)=C1C#N | 10 | I |
| CN1N=CC2=C1CCO[C@@H]2CBr | 10 | I |
| ClC1=CC(=C(Cl)C=C1)[S@](=O)CC#N | 10 | I |
| FC1=C(F)C=C(C=C1)[S@](=O)[C@H]1CCC(=O)C1 | 10 | I |
| C[C@@H]1[C@@H](CCC1=O)[S@@](=O)C1=CC=C(C)C=C1 | 10 | I |
| C[C@@H]1[C@@H](Br)CC[C@H]1C1=CN=CN=C1 | 10 | I |
| C[C@H](Cl)C1=NN=C(S1)[C@@H]1CCCS1 | 10 | I |
| C[C@H]1CC[C@]2(C)C[C@H](C([O-])=O)C(=O)C[C@H]2C1=C | 10 | I |
| CC#CCN1C=NC(C)=C(Br)C1=O | 10 | I |
| C[C@@H](Cl)C(=O)N1C[C@H]2CCCN2C[C@H]1C | 10 | I |
| BrCCN1CCN2CCC[C@H]2C1 | 10 | I |
| CC1=CC(Cl)=NC(=N1)[C@H]1CSCCO1 | 10 | I |
| CC1=C(C)C(C(=O)CBr)=C(C)O1 | 10 | I |
| C([C@@H]1CCCS1)N1CCCN2CCC[C@@H]2C1 | 10 | I |
| ClCC1=CN=C(N=C1)[C@H]1CCCCS1 | 10 | I |
| Br[C@@H]1CCN(CC2=CC=NC=C2)C1 | 10 | I |
| C[C@H]1CC(=O)CCN1C[C@]1(C)CCCS1 | 10 | I |
| BrC[C@H]1C[C@@]11CCS(=O)(=O)C1 | 10 | I |
| CC1(C)CN(CC2=CCCOC2)CCS1 | 10 | I |
| N#C[C@H]1CCCN(C1)[C@@H]1CCOC2(CCC2)C1 | 10 | I |
| N#C[C@@H]1CCCN(C1)[C@@H]1CCOC2(CCC2)C1 | 10 | I |
| O=C1CCCC[C@@H]1[C@H]1CCO[C@]2(CCOC2)C1 | 10 | I |
| C[C@H]1[C@H](CCC1=O)[C@@H]1CCO[C@]2(CCOC2)C1 | 10 | I |
| C1C[C@@H]2CN(CCN2C1)[C@@H]1CN2CCC1CC2 | 10 | I |
| ClC1=CC=C(Br)C2=NN=C[C@H]12 | 10 | I |
| BrC1(CCOCC1)C1OCCO1 | 10 | I |
| CC1=NN=C2SC(=N[C@H]12)C1=CC=CC=C1F | 10 | I |
| CN1N=C(Br)N=C1C(F)(F)F | 10 | I |
| N#C[C@@]1(CCN2CC[C@@H]1C2)N1CCCCCC1 | 10 | I |
| FC1=CC=C(C=C1)[C@@H]1C[C@H]1S(Cl)(=O)=O | 10 | I |
| CN1N=C(C)C(Cl)=C1I | 10 | I |
| FC1=CC(N2C(=O)CSC2=S)=C(F)C=C1 | 10 | I |
| BrC1=CN=CC(Br)=C1C#N | 10 | I |
| CCN1C(C)=CC(=O)C2=C1C=CC(Br)=C2 | 10 | I |
| CC1=CC=C(\C=C2/SC(S)=NC2=O)C=C1 | 10 | I |
| C[C@H](Br)C1=C(F)C(Cl)=NC=N1 | 10 | I |
| BrC1=CC2=C(OC(=C2)[C@H]2CO2)C=C1 | 10 | I |
| BrC1=CC2=C(C=C1)N=C1OCCC1=C2 | 10 | I |
| CC1(C)CC2(OCCO2)C(Br)=C1 | 10 | I |
| SC1=C(Br)C=C2OCCOC2=C1 | 10 | I |
| Cl\C(=C/C#N)C1=CC=C(Br)O1 | 10 | I |
| ClC1=C(Cl)C=C(C=C1)N1CSCC1=O | 10 | I |
| BrCC1=C2OCCOC2=CC=C1 | 10 | I |
| C[C@@H](Br)C(=O)N1C[C@@H](C)C[C@H](C)C1 | 10 | I |
| BrC1=CC=C(O1)C1=NC=CS1 | 10 | I |
| ClS(=O)(=O)C1=CC=C(Br)S1 | 10 | I |
| BrC1=CC(CC#N)=CC2=C1OCC=C2 | 10 | I |
| ClC1=CN=C2C=CC=C(Br)N12 | 10 | I |
| ClC1=NC=CC(=N1)C(Cl)(Cl)Cl | 10 | I |
| CC[C@@]1(C)C[C@@]2(CCO1)C[C@H](Cl)CCO2 | 10 | I |
| C[C@@H]1CC[C@H](C#N)[C@@H](C1)N1CCSC[C@@H]1C | 10 | I |
| C[C@@H]1CC[C@@H](C#N)[C@@H](C1)N1CCSC[C@@H]1C | 10 | I |
| C[C@H]1CC[C@@H](C#N)[C@@H](C1)N1C[C@H](C)S[C@H](C)C1 | 10 | I |
| CN1N=CC=C1[C@H]1CCCC[C@H](Br)C1 | 10 | I |
| C[C@H]1[C@H]2OC(C)(C)O[C@@H]2[C@H](C)P1C1CCCC1 | 10 | I |
| CCN1C(Br)=NC(Cl)=C1Cl | 10 | I |
| ClC1=CC=C(C=C1)N1C=C(Br)C=N1 | 10 | I |
| CC(C)N1C=CC(I)=N1 | 10 | I |
| CC1=C(Br)C=CC2=C1OCCO2 | 10 | I |
| FC(F)(F)[C@H]1CCC[C@]2(C1)CC(=O)C=CO2 | 10 | I |
| C[C@@H]1OC2=C(Br)C=C(F)C=C2C1=O | 10 | I |
| Cl[C@H]1CCO[C@@H](C1)C1=NC2=CC=CC=C2C=C1 | 10 | I |
| CC1=C(C)C(Cl)=NC(=N1)[C@@H]1CSCCS1 | 10 | I |
| C[C@@H]1CCN(C1)C1=C(F)C(F)=NC(F)=C1F | 10 | I |
| C[C@H]1CC[C@H](C)C2=C1ON=C2CBr | 10 | I |
| FC(F)(F)C1=CN2C=CN=C2C(Br)=C1 | 10 | I |
| COC1=CC(Br)=NC=C1Br | 10 | I |
| CN1C=C2C=CC(I)=CC2=N1 | 10 | I |
| C[C@@H]1[C@@H](C)C(=O)CCN1C1CCC(C)(C)CC1 | 10 | I |
| O=C1CCC[C@@H]1[C@@H]1CCOC2(CCSCC2)C1 | 10 | I |
| C[C@@H]1[C@@H](CCC1=O)[C@@H]1CCO[C@]2(CCSC2)C1 | 10 | I |
| O=C1CCCC(=C1)[C@@H]1CCOC2(CCCC2)C1 | 10 | I |
| O=C1CCCC(=C1)[C@@H]1CCOC2(CCSCC2)C1 | 10 | I |
| O=C1CCC(=C1)[C@@H]1CCOC2(CCCCC2)C1 | 10 | I |
| CC1(C)[C@@H]2C[C@H]1C1=CC[C@@H]3[C@H]([C@H]1C2)C(=O)C=CC3=O | 10 | I |
| FC(F)(F)C1=NC2=CC(Br)=CCC2=N1 | 10 | I |
| CC1=C2N=NC(I)=C2C=CC1 | 10 | I |
| COC1=CC(F)=C(C=C1Br)C#N | 10 | I |
| CC1=CC=C(C)N1C1=CC(Br)=CC=N1 | 10 | I |
| ClS(=O)(=O)C1=C(Br)C=CS1 | 10 | I |
| FC1=C(F)C(F)=C(N2C(=O)C=CC2=O)C(F)=C1F | 10 | I |
| CN1N=CN(C1=S)C1=CC=CC(=C1)C(F)(F)F | 10 | I |
| ClCC1=CC=C(C=C1)N1CCS(=O)(=O)CC1 | 10 | I |
| FC1=C(CN2C(=O)CCC2=O)C=C(Br)C=C1 | 10 | I |
| BrC1=CC=C2OCC(=O)N(CC#C)C2=C1 | 10 | I |
| ClC1=C(Cl)C(=O)N(CBr)C1=O | 10 | I |
| C[C@H](C#N)N(C)C(=O)C1=CC=C(Br)C=C1 | 10 | I |
| CC[C@H](Br)C(=O)N1C[C@@H](C)OC[C@@H]1C | 10 | I |
| FC1=C(C=CC(CCl)=C1)N1CCN2C=CN=C2C1 | 10 | I |
| CC1=NN(C(C)=N1)C1=C(F)C=C(CCl)C=C1F | 10 | I |
| CS(=O)(=O)C1=CC=C(C=C1)C1=CC=C[C@H]2N=CC=C12 | 10 | I |
| CC1=NN(C2CCOCC2)C(C)=C1Br | 10 | I |
| Br[C@@H]1CCCN(C[C@@H]2CCCOC2)C1=O | 10 | I |
| FC1=C(C=CC(Br)=C1)N1C[C@@H](CC1=O)C#N | 10 | I |
| O=C1[C@H]2OC3=CC=CC=C3C3(SCCCS3)[C@@H]2C1=O | 10 | I |
| BrC[C@@H](C#N)C1=NN=CC2=CC=CC=C12 | 10 | I |
| Cl[C@H]1CCN(C1)C1=NC=NC=C1Br | 10 | I |
| CC1=CC(Br)=CC(C)=C1N1CC(=O)CC1=O | 10 | I |
| ClC1=NC(=NC=C1Br)[C@@H]1CSCCO1 | 10 | I |
| ClC1=NC(=NC=C1Br)[C@H]1CSCCO1 | 10 | I |
| C[C@H](Cl)C1=C(C)N=C(N=C1)[C@H]1CSCCO1 | 10 | I |
| BrC[C@H]1CCCN1C(=O)C1CCOCC1 | 10 | I |
| BrC[C@@H]1CCN(C1)C(=O)[C@@H]1CCCCO1 | 10 | I |
| CN1N=C(C)C(Cl)=C1CN1CC[C@@H](Br)C1 | 10 | I |
| CN1N=C(C)C=C1CN1CC[C@H](Br)C1 | 10 | I |
| CCC1=CC(=NC(C)=N1)N1CC[C@H](Br)C1 | 10 | I |
| C[C@H]1CC[C@@H](O1)C(=O)N1CCC(Br)CC1 | 10 | I |
| C[C@@H]1CC[C@H](O1)C(=O)N1CCC(Br)CC1 | 10 | I |
| C[C@@H]1CC[C@@H](O1)C(=O)N1CCC[C@@H]1CBr | 10 | I |
| C[C@@H]1CC[C@H](O1)C(=O)N1CCC[C@@H]1CBr | 10 | I |
| C[C@H]1CCC[C@@H](CC(=O)N2CC[S@@](=O)C(C)(C)C2)C1 | 10 | I |
| C[S@](=O)C1=NC2=C(S1)C=C(Br)C=N2 | 10 | I |
| C[C@H]1CC(=O)N(CC2=CSC(Br)=C2)C1=O | 10 | I |
| O=C(CN1CCSSCC1)N1CCCCC1 | 10 | I |
| BrC1=CC(OC2CCOCC2)=NC=C1 | 10 | I |
| O=S1(=O)CCCN1C[C@@H]1CSC2=CC=CC=C12 | 10 | I |
| C[C@@H]1[C@H](C)S(=O)(=O)C2=CC3=C(CCC3)C=C2C1=O | 10 | I |
| CN1CCO[C@@H]2CN(CC3=CC=C(F)C(C)=C3)C[C@H]12 | 10 | I |
| N#CC1CCN(CC1)[C@H]1CCO[C@@]2(CCSC2)C1 | 10 | I |
| CN(C1CCC(=O)CC1)[C@H]1CCO[C@]2(CCSC2)C1 | 10 | I |
| O=C([C@H]1CCCOC1)[C@@H]1CCOC2(CCSCC2)C1 | 10 | I |
| O=C[C@@H]1CCCCN1[C@@H]1CCO[C@]2(CCSC2)C1 | 10 | I |
| CC[C@@H]1CN2CCC[C@@H]2CN1[C@@H]1CCN2CCC[C@H]12 | 10 | I |
| C[C@@H]1C[C@@H](C)[C@H](CN2CCN3CCC[C@@H]3C2)C(=O)C1 | 10 | I |
| CS(=O)(=O)[C@@H]1CCC[C@@H](C1)[C@@H]1CCCC(=O)C1 | 10 | I |
| CS(=O)(=O)[C@H]1CCC[C@H](C1)[C@@H]1CCCC(=O)C1 | 10 | I |
| C[C@@H]1[C@@H](CCC1=O)[C@@H]1CCC[C@@H](C1)S(C)(=O)=O | 10 | I |
| FC1=C(F)C(F)=C(N2C=CN=C2C#N)C(F)=C1F | 10 | I |
| FC(F)(F)C1=CN2C(Br)=CN=C2N=C1 | 10 | I |
| O=C1CCC[C@H]1SC1=CC2=C(OCCCO2)C=C1 | 10 | I |
| CC1=CC=CC(=N1)C#CC=C1CCN(CC1)C1=CC=CC=C1 | 11 | A |
| CC1=CC=CC(=N1)C#CC=C1CCN(CC1)C1=NC=C(C=C1C#N)C1=CC=CC=C1 | 11 | A |
| [O-][N+](=O)C1=CC=CN=C1N1CCC(CC1)=CC#CC1=CC=CC=C1 | 11 | A |
| CC1=CC=CC(=C1)C#CC=C1CCN(CC1)C1=NC=CC=C1[N+]([O-])=O | 11 | A |
| [O-][N+](=O)C1=CC=CN=C1N1CCN(CC1)C(=O)C#CC1=CC=CC=C1 | 11 | A |
| [O-][N+](=O)C1=CC=CN=C1N1CCC(CC1)=CC#CC1=CC=CC(Cl)=C1 | 11 | A |
| CC1=CC=CC(=N1)C#CC=C1CCN(CC1)C1=CC=CC=C1[N+]([O-])=O | 11 | A |
| FC1=CN2C=C(CCC#CC3=CC=CC=N3)N=C2C=C1 | 11 | A |
| [O-][N+](=O)C1=CC=C(C=C)N=C1N1CCN(CC1)C(=O)C#CC1=CC=CC=C1 | 11 | A |
| [O-][N+](=O)C1=CC=CN=C1N1CCC(CC1)=CC#CC1=CC=CC=N1 | 11 | A |
| CC1=CC=CC(=N1)C#CC=C1CCN(CC1)C1=NC=CC=C1[N+]([O-])=O | 11 | A |
| CC1=CC=C(C(=N1)N1CCC(CC1)=CC#CC1=CC=C(C=N1)C#N)[N+]([O-])=O | 11 | A |
| CC1=CN=C(N2CCC\C(C2)=C/C#CC2=CC=CC(C)=N2)C(=C1)[N+]([O-])=O | 11 | A |
| CC1=NN=NN1C1=NC(=CC(=N1)C(F)(F)F)C1=CC=C(Cl)C=C1 | 11 | I |
| CC(=O)C1=CC=C(C=C1)N1N=NC2=C(SC3=C2C(C)=CC(C)=N3)C1=O | 11 | I |
| CC1=NN(CC2=CC=C(O2)C(=O)N2C=C(Cl)C=N2)C(C)=C1Cl | 11 | I |
| CC1=C(SN=N1)C1=NN2C(CC3=CC=C(F)C=C3)=NN=C2S1 | 11 | I |
| CC1=CC(=O)OC2=C1C=CC1=C2CN(CO1)C1=CC=C2OCCOC2=C1 | 11 | I |
| CN1C(=S)S\C(=C\C2=CC=C(O2)C2=CC=C(C=C2)[N+]([O-])=O)C1=O | 11 | I |
| CC1(C)CC2=C(CO1)C=C1C(OC3=C1N=CN=C3N1CCCCC1)=N2 | 11 | I |
| FC1=CC=C(OC2=C(C=C(C#N)C#N)C(=O)N3C=CC=CC3=N2)C=C1 | 11 | I |
| C1CS[C@@H]([C@H](S1)N1N=NC2=CC=CC=C12)N1N=NC2=CC=CC=C12 | 11 | I |
| CCSC1=NN=C2N3N=CN=C3C3=C(SC4=C3CCCC4)N12 | 11 | I |
| CN1C(=S)S\C(=C\C2=CC=C(O2)C2=CC=C(C=C2C)[N+]([O-])=O)C1=O | 11 | I |
| C[C@@H]1CN(C[C@@H](C)O1)C1=NC=NC2=C1C=NN2C1=CC=C(C)C=C1C | 11 | I |
| CS(=O)(=O)N1N=C(C[C@H]1C1=CC=C2OCOC2=C1)C1=CC=CS1 | 11 | I |
| CC1=CC=C(C=C1)C1=NN(CC2=CC(=O)N3C=CC=CC3=N2)C(=O)C=C1 | 11 | I |
| CC1=CC=CN2C(=O)C3=C(N=C12)N(CC1=CC=C(F)C=C1)C(=O)C(=C3)C#N | 11 | I |
| CC1=NN(C(C)=C1)C1=NN=C(C)N1\N=C\C1=CC=C2C=CC=CC2=C1 | 11 | I |
| CC1(C)CC(=O)C2(CC3=CC(=CC=C3N3CCCC[C@@H]23)[N+]([O-])=O)C(=O)C1 | 11 | I |
| C[C@@H]1CC2=CC=CC=C2N1C(=O)CN1N=NC(=N1)C1=CC=CC=C1C | 11 | I |
| O=C(N1CCC[C@@H]1C1=CC=C2OCCCOC2=C1)C1=CN2C=CC=CC2=N1 | 11 | I |
| COC1=CC(\C=C2/N=C(OC2=O)C2=CC=CC=C2C)=CC2=C1OCCO2 | 11 | I |
| CC1=NC2=NC=NN2C(SCC2=CC3=C(OCO3)C=C2Cl)=C1 | 11 | I |
| FC1=CC=CC=C1N1C=CC2=NC3=NC=NN3C(C3=CC=CC=C3)=C2C1=O | 11 | I |
| CC1=C2C=CC=CC2=C(N=N1)N1CCC[C@H](C1)C1=NN=C2CCCCCN12 | 11 | I |
| O=C(\C=C\C1=CC2=CC=CC=C2O1)N1CCC[C@@H]1C1=NN=C2C=CC=CN12 | 11 | I |
| CC1=CC=C(CC2=NN3C(=O)\C(SC3=NC2=O)=C/C2=CC=CO2)C=C1 | 11 | I |
| CN(C[C@H]1COC2=CC=CC=C2O1)C1=NC=NC2=C1OC1=CC=CC=C21 | 11 | I |
| O=C1N([C@@H](C2=C1OC1=CC=CC=C1C2=O)C1=CC=CC=C1)C1=NN=CS1 | 11 | I |
| CN1\C(SC2=CC3=C(OCCO3)C=C12)=N\C(=O)C1=C(C)N=C(C)S1 | 11 | I |
| IC1=CC=CC=C1OC1=NN2N=NN=C2C=C1 | 11 | I |
| FC1=CC=C(C=C1)N1C=CC(=O)C(=N1)C1=NC(=NO1)C1=CC=CC=C1F | 11 | I |
| CC1=NC(=CO1)C1=NN2C(S1)=NN=C2C1=CC=C(Cl)C=C1F | 11 | I |
| CCCN1C(=O)\C(SC1=C(C#N)C#N)=C\C1=CC=CC2=C1OCCCO2 | 11 | I |
| C[C@H](SC1=NC(=O)C2=C3CCCC[C@H]3SC2=N1)C1=NC(C)=NO1 | 11 | I |
| CC1CCN(CC1)S(=O)(=O)C1=CC(=CC=C1C)C1=NOC(C)=N1 | 11 | I |
| O=C(N1CCC2=C1C=CC(=C2)C1=CC=NO1)C1=CC=C2OCOC2=C1 | 11 | I |
| CC1=C(CN2C3=C(SC=C3)C(=O)N(C2=O)C2=CC=CC=C2C)C(C)=NO1 | 11 | I |
| ClC1=CC(=CC=C1C#N)S(=O)(=O)OC1=CC2=C(C=CC(=O)O2)C=C1 | 11 | I |
| CC1=NOC(=N1)[C@H]1CCCN(C1)S(=O)(=O)C1=C(C)C(C)=CC(C)=C1C | 11 | I |
| ClC1=CN2C(=O)C=C(CSC3=NN=C4CCCCCN34)N=C2C=C1 | 11 | I |
| CC(C)(C)C1=NC(CN2C=NC3=C(C4=C(CCCC4)S3)C2=O)=NO1 | 11 | I |
| CC1=NN2C(=N1)C1=CC=CC=C1N=C2SCC1=CN2C(C=CC=C2C)=N1 | 11 | I |
| CC1=NC2=NC(SCC3=NC(C)=C4C=CC=CC4=N3)=NN2C(C)=C1C | 11 | I |
| CN1N=C(C)C2=C1N=CC(=C2)S(=O)(=O)OC1=CC=C(C)C=C1Cl | 11 | I |
| CC(C)C1=NN(C)C(=C1)C1=NN=C2SC(=NN12)C1=CSC=C1 | 11 | I |
| C[C@@H]1CN(C(=O)C2=CC=C(C=C2)N2C=CN=N2)C2=CC(Cl)=CC=C2O1 | 11 | I |
| CC1=C(C=NN1C1=CC=NC=C1)C1=NC(=NO1)C1=CC=C(F)C(C)=C1 | 11 | I |
| CN([C@@H]1CCN(C1=O)C1=CC=CC=C1F)C(=O)C1=CN=C2C=CC(C)=CN12 | 11 | I |
| COC1=CC(\C=C2\N=C(OC2=O)C2=CC=CC=C2C)=CC2=C1OCCO2 | 11 | I |
| FC1=CN=C(N=C1)N1CC[C@]2(C1)CN(CCC2(F)F)C1=NN=CS1 | 11 | I |
| O=C(\C=C/C1=CC=C2OCCC2=C1)N1CCC[C@@H]1C1=NN=C2C=CC=CN12 | 11 | I |
| CC1=CC2=CC3=C(OCO3)C=C2N2C(SCC(=O)N3CCCC3)=NN=C12 | 11 | I |
| O=C(OC1=CC(=O)OC2=C1C(=O)N1CCC3=C1C2=CC=C3)C1=CC=CO1 | 11 | I |
| COC1=CC=C(C=C1Cl)C1=CC=C(O1)C=C1C(=O)N(C)C(=O)N(C)C1=O | 11 | I |
| CN1C2=C(N=C(Cl)N2CC(=O)C2=CC=C(C)C=C2)C(=O)N(C)C1=O | 11 | I |
| O=C1N(CCC2=NN3C=NC4=CC=CC=C4C3=N2)N=CC2=CC=CC=C12 | 11 | I |
| CN1C2=CC=CC=C2C(=O)C2=C1N=C(N(CC1=CC=CO1)C2=O)C1=CC=CO1 | 11 | I |
| CC1=CC(=NO1)N1[C@@H](C2=C(OC3=CC=C(C)C=C3C2=O)C1=O)C1=CC=CN=C1 | 11 | I |
| CC1=CC=C(C=C1)C(=O)N=C(N1N=NC2=CC=CC=C12)N1CCOCC1 | 11 | I |
| CN1C(=O)N(C)C2=C(C1=O)C(SCC#N)=NC(=N2)C1=CC(C)=CC(C)=C1 | 11 | I |
| C(C1=NN2C(S1)=NN=C2C1=CC=CC=N1)C1=CC=C2OCCOC2=C1 | 11 | I |
| O=C1C[C@H](CN1C1=CC=CC=C1)C1=NC(=NO1)C1=CC=C2OCOC2=C1 | 11 | I |
| C[C@@H](N1C=NC2=C1C(=O)N(C)C(=O)N2C)C(=O)C12CC3CC(CC(C3)C1)C2 | 11 | I |
| O=C(\N=C1/SC2=CC3=C(OCCO3)C=C2N1CC#C)C1=CC=NO1 | 11 | I |
| CN1N=CC2=C1N=CN(CC1=CC(=O)OC3=C1C=C(Cl)C(C)=C3)C2=O | 11 | I |
| CC1=NC2=NC=NN2C(=C1)N1CCC[C@@H]1C1=CC=C2OCCCOC2=C1 | 11 | I |
| CS(=O)(=O)C1=NC2=CC=CC=C2N1CC1=CC(=O)N2C=CC=CC2=N1 | 11 | I |
| C[C@H]1CN(C[C@@H](C)O1)C(=O)C1=CC2=C(N=C3N(C=CC=C3C)C2=O)N1C | 11 | I |
| CN1N=C([C@H]2CCCN(C2)S(=O)(=O)C2=CC=C(C)C=C2)C2=NC=CN=C12 | 11 | I |
| CN1C2=C(N(CC(=O)C3=C(C)C=C(C)C=C3C)C=N2)C(=O)N(C)C1=O | 11 | I |
| O=C(N1CCC[C@@H]1C1=NN=C2C=CC=CN12)C1=CSC(=N1)C1=CC=CO1 | 11 | I |
| CC1=NC2=NC(SCC3=CC(=O)N4C(C)=CC=CC4=N3)=NN2C(C)=C1 | 11 | I |
| CC1(C)CC2=C(CO1)C=C1C(OC3=C1N=CN=C3N1CCOCC1)=N2 | 11 | I |
| CN1N=C(C)C=C1CN1CCC2=NN=C([C@@H]2C1)[C@@H]1COC2=CC=CC=C2O1 | 11 | I |
| CN1C(=CC2=C1C=CO2)C(=O)N1CCN(CC2=CC=C3OCOC3=C2)CC1 | 11 | I |
| O=C(N1CC(C1)C1=NC(=NO1)C1=CC=CC=C1)C1=CC=C2OCOC2=C1 | 11 | I |
| CC1=NN(CC2=CC3=C(OCCO3)C=C2Br)C(C)=C1[N+]([O-])=O | 11 | I |
| COC1=CC=C(C=C1OC)C1=C2C(=O)C3=CC=CC=C3C2=NC2=NC=NN12 | 11 | I |
| C[C@H]1CCCN(C1)C(=O)CN1C=NC2=C(N=C3CCCCCN23)C1=O | 11 | I |
| C[C@H]1C[C@@H](C)CN(C1)C(=O)CN1C=NC2=C(N=C3CCCCCN23)C1=O | 11 | I |
| C[C@H](N1C=NC2=C1C=CC=C2)C1=NC(C)=NN1C1=CC=C2OCOC2=C1 | 11 | I |
| O=C1C=C(CN2CCC(CC2)C2=NN=C3C=CC=CN23)N=C2C=CC=CN12 | 11 | I |
| COCC1=NC2=C(C=NN2C)C(=N1)N1CCOC2=CC=C(Cl)C=C2C1 | 11 | I |
| O=C(N1C[C@H]([C@@H]2[C@H]1C1CCN2CC1)C1=CC2=C(OCO2)C=C1)C1=COC=N1 | 11 | I |
| CCN1C2=C(OC(C)=C2)C2=C1C(=O)N(CC(=O)N1[C@@H](C)CCC[C@H]1C)N=C2 | 11 | I |
| CC1=CC=CC2=C1N=C1OCCN(CC3=NN=NN3C(C)(C)C)CC1=C2 | 11 | I |
| COC1=CC=CC=C1N1C=CC2=C(C(C)=NC3=NC(SC)=NN23)C1=O | 11 | I |
| CN1C=C2N=CN(CC3=NC4=CC(=CC=C4N3C)C(F)(F)F)C(=O)C2=N1 | 11 | I |
| CC1=C(N=NN1C1=CC=C(F)C(Cl)=C1)C1=NC(=NO1)C1=CC=NC=C1 | 11 | I |
| CC1=NOC2=NC(C)=NC(N3CCN(CC4=CC(C)=CC(C)=C4)C(=O)C3)=C12 | 11 | I |
| ClC1=CN=C(C=C1)N1CCN(CC1)C(=O)C1=C(OC=N1)[C@H]1CCCO1 | 11 | I |
| [O-][N+](=O)C1=CC2=C(C=C1)N=CN=C2N1C=C(I)C=N1 | 11 | I |
| C[C@H]1CC2=CC(=CC=C2N1C(=O)C1=CN=C(S1)[C@@H]1CCCO1)[N+]([O-])=O | 11 | I |
| CN(C)C(=O)C1=NOC2=C1CN(CC2)C(=O)C1=C(C)C2=CC=C(C)C=C2O1 | 11 | I |
| O=C(CC1=CC2=C(OCO2)C=C1)N1C[C@H]2CC[C@@H]1CN(C2)C1=CN=CC=N1 | 11 | I |
| O=C1C=C(CN2C[C@H]3CC[C@@H]2CN(C3)C2=NC=CC=N2)N=C2C=CC=CN12 | 11 | I |
| CC1=NN=C2C=CC(=NN12)N1CCN(CC2=CC=C3OC(C)(C)CC3=C2)CC1 | 11 | I |
| CC1=NC(=NO1)[C@@H]1COCCN1S(=O)(=O)C1=CC(Cl)=CC(Cl)=C1 | 11 | I |
| C[C@H]1N(CCN(C1=O)C1=CC=C(F)C=C1F)C(=O)C1=CC=C(C=C1)[N+]([O-])=O | 11 | I |
| COC1=CC(=CC2=C1OCCO2)[C@@H]1CC(=O)OC2=C1C1=NSN=C1C=C2 | 11 | I |
| COC1=CC=C(C=C1C)S(=O)(=O)N1CCC2=C(C1)C=NC1=CC(C)=NN21 | 11 | I |
| O=CC1=C(SC2=NN=N[C@@H]2C2=CC=CC=C2)N=C2C=CC=CN2C1=O | 11 | I |
| C(OC1=CC=CC=C1)C1=NN2C(S1)=NN=C2[C@@H]1COC2=CC=CC=C2O1 | 11 | I |
| CC1=NN(CC2=NC(=NO2)C2=CC=C3OCOC3=C2)C(C)=C1Br | 11 | I |
| CCCN1\C(SC2=CC3=C(OCCO3)C=C12)=N\C(=O)C1=COCCO1 | 11 | I |
| CC1=CN2C(=O)C=C(COC(=O)C3=CC=C4OCCOC4=C3)N=C2C=C1 | 11 | I |
| [O-][N+](=O)C1=CC=C2OC(=O)C(=CC2=C1)S(=O)(=O)C1=CC=C(F)C=C1 | 11 | I |
| COC1=CC=C2C(=O)[C@]3(OC)C(=NCCC4=CC5=C(OCO5)C=C34)C2=C1OC | 11 | I |
| CCCCN1C(=O)\C(=C2/SC3=NC(=O)C(C)=NN3C2=O)C2=CC=CC=C12 | 11 | I |
| CC1=NC2=NC(SCC3=NC(=NO3)C3=CC=CS3)=NN2C(C)=C1 | 11 | I |
| CC(=O)N1N=C(COC2=CC=C3OCOC3=C2)O[C@@H]1C1=CC=CC=C1C | 11 | I |
| C\C=C\CN1C(=O)N(C)C2=C(N3C=C(C)N(C3=N2)C2=CC=C(F)C=C2)C1=O | 11 | I |
| COC(=O)CN1\C(SC2=CC3=C(OCCO3)C=C12)=N\C(=O)C(C)C | 11 | I |
| CC1=CC=C(C=C1)C1=NOC2=C1N=CN(CCC(=O)N1CCCCCC1)C2=O | 11 | I |
| C[C@H](SC1=NN2C(=N1)N=C(C)C=C2C)C(=O)N(C)C1(CCCCC1)C#N | 11 | I |
| COC1=CC=C(C=C1OC)C1=NOC(=N1)C1=CC2=CC(F)=CC=C2OC1=O | 11 | I |
| CC1=NC2=C(C3=C(S2)C2=NC(=NN2C=N3)C2=CC=NN2C(F)F)C(C)=C1 | 11 | I |
| CN1N=NN=C1SCC1=NC(=NO1)C1=CC=CC(Br)=C1 | 11 | I |
| ClC1=CC=C(C=C1)C1=NOC2=C1C(=NC=N2)N1CCC2(CC1)OCCO2 | 11 | I |
| ClC1=CC2=C(C=C1)C(SC1=NN=NN1C[C@H]1CCCO1)=NC=N2 | 11 | I |
| CN1C2=NN=C(CSC3=NC=NC4=CC=CC=C34)N2C2=CC=CC=C2C1=O | 11 | I |
| C[C@H]1C[C@H](C)CN(C1)C(=O)\C=C\C1=CC(=CC2=C1OCOC2)[N+]([O-])=O | 11 | I |
| CN1C2=C(N(CC3=CC=C(OC(F)(F)F)C=C3)C=N2)C(=O)N(C)C1=O | 11 | I |
| ClC1=C(Cl)C=C(C=C1)N1N=CC2=C1N=CN1N=C(N=C21)[C@@H]1CCCO1 | 11 | I |
| CN(CC1=NN=C(O1)C1=CC=C(C=C1)C(C)(C)C)C(=O)N1CCOCC1 | 11 | I |
| CC(C)C1=CC(=NO1)[C@@H]1CCCN1S(=O)(=O)C1=CC=C2OCCOC2=C1 | 11 | I |
| CCC1=NOC(=C1)[C@@H]1CCCN1S(=O)(=O)C1=CC=CC2=NSN=C12 | 11 | I |
| CN1C2=C(N(CCOC3=CC=C(Br)C=C3)C=N2)C(=O)N(C)C1=O | 11 | I |
| CC[C@@H]1N(CCN2C=CC=C12)C(=O)[C@H]1C=C(CN2C=NC3=C2C=CC=C3)N=N1 | 11 | I |
| CN(C)C(=O)[C@]12CC[C@](C)(C3=C1N=C1C=C(C=CC1=N3)[N+]([O-])=O)C2(C)C | 11 | I |
| CCC1=CC=C(C=C1)C1=NC(=NO1)[C@@H]1CN(C(=O)C1)C1=CC=C2OCOC2=C1 | 11 | I |
| C[C@@H](SC1=NN=C2C=C(C)N=C(C)N12)C1=NC(=NO1)C1=CC=CC=C1 | 11 | I |
| CN1C(=O)OC2=CC(=CC=C12)[C@H](Br)C1=CC=C(O1)[N+]([O-])=O | 11 | I |
| BrC1=CC=C(CN2CCN(CC2)C2=NN3N=NN=C3C=C2)S1 | 11 | I |
| BrC1=CC(=CC=C1)S(=O)(=O)OC1=CC=CC(=C1)N1C=NN=N1 | 11 | I |
| CC1=CSC2=NC(COC(=O)C3=CN4C=CC(C)=CC4=N3)=CC(=O)N12 | 11 | I |
| C[C@H]1CSC2=NC(CS[C@@H]3N=NC4=NC5=CC=CC=C5N34)=CC(=O)N12 | 11 | I |
| C[C@@H]1CCCN(C1)S(=O)(=O)C1=CC=C(O1)C1=NN=C(O1)C1CCCC1 | 11 | I |
| CC1CCN(CC1)S(=O)(=O)C1=CC(=CO1)C1=NN=C(O1)C1CCCC1 | 11 | I |
| CC1=C(N=NN1C1=CC=C2OCCOC2=C1)C(=O)OC1=CC=CC=C1F | 11 | I |
| CN(CC1=NOC2=C1CCCC2)C1=NC(=NC2=C1C=NN2C)C1CCCC1 | 11 | I |
| CC1=CC=C(S1)C1=NOC(=N1)C1CCN(CC1)C1=C(N=C(C)O1)C#N | 11 | I |
| CCC[C@@H]1CN(CCO1)C(=O)C1=C(OC=N1)C1=CC=C2OCCOC2=C1 | 11 | I |
| C(N1C=NC2=CC=CC=C12)C1=CC=CC(=C1)C1=NOC(=N1)C1=CC=NN=C1 | 11 | I |
| CN(C)C1=NC=CC(=N1)N1CCN(CC2=CC=CC3=C2OCCCO3)CC1 | 11 | I |
| CC(C)C1=NOC(=N1)C1CCN(CC1)C1=NC=NC2=C1C(C)=CC(C)=N2 | 11 | I |
| CCCN1C(=O)C(C)=NC2=CC(=CC=C12)C1=NOC(=N1)C1=C(C)N=C(C)S1 | 11 | I |
| CCN1C(=O)S\C(=C/C2=C(C)N(C(C)=C2)C2=CC=CC(=C2)[N+]([O-])=O)C1=O | 11 | I |
| CC1=NN(C(=O)[C@H]1\C=N\N1C(C)=NC2=CC=CC=C2C1=O)C1=CC=C(C)C=C1 | 11 | I |
| BrC1=CC=CC(=C1)C1=NOC(=N1)C1=NC(=NO1)C1=CC=NC=C1 | 11 | I |
| CCN1C=CC(=N1)C(=O)\N=C1/SC2=CC3=C(OCCO3)C=C2N1CC#C | 11 | I |
| CCC1=NC(C)(C)CC2=C1C=C1OCCOC1=C2 | 11 | I |
| CC1=N\C(=C\C2=CC=C(Cl)C(Cl)=C2)C(=O)O1 | 11 | I |
| FC1=CC(=CC=C1CN1C=NC2=CC=CC=C12)C#N | 11 | I |
| CC1=NN(C(=O)[C@]2(C)CC2(Cl)Cl)C(C)=C1 | 11 | I |
| CN1N=C(C)C2=C(C=C(Cl)N=C12)C(F)(F)F | 11 | I |
| FC1=C(C2=NN=C(CCl)O2)C(Cl)=CC=C1 | 11 | I |
| CN1CCN(CC1)C(=O)[C@H]1[C@@H](C=C(C)C)C1(C)C | 11 | I |
| CC(C)N1CCN(CC1)[C@H]1CCCCC[C@H]1C#N | 11 | I |
| C[C@H]1CCC[C@@H](C)N1CC1=C(Cl)N(C)N=C1C | 11 | I |
| C[C@H]1CCN(C1)C1=C(CCl)N2C=CSC2=N1 | 11 | I |
| ClCC1=NN=C(O1)C1=CC2=CC=CC=C2S1 | 11 | I |
| N#C[C@@H]1CCCCC[C@H]1N1CCO[C@H]2CCC[C@H]12 | 11 | I |
| C[C@@H]1CN(CC(C)(C)O1)C1=CC(CCl)=CC=N1 | 11 | I |
| FC1=CC(=CC=C1CN1N=CC2=CC=CC=C12)C#N | 11 | I |
| C[C@H]1CCN(CC2=C(Cl)SN=N2)[C@H](C)C1 | 11 | I |
| CCN1C(=CC2=C1C=C(Cl)S2)C(=O)N(C)C | 11 | I |
| C[C@H]1CN(C[C@H](C)S1)C(=O)C1(CCCCC1)C#N | 11 | I |
| ClC1=C(CN2C=NC=N2)CCC2=CC=CC=C12 | 11 | I |
| ClC1=CC2=C(C=C1)N(C=C2)\N=C\C1=CN=CC=C1 | 11 | I |
| O=C1C[C@@H]2C=C[C@@H]3C[C@@H](O[C@H]4CCCCO4)[C@H]1[C@H]23 | 11 | I |
| [O-][N+](=O)C1=CC(F)=C(F)C=C1I | 11 | I |
| FC1=CC=C(C=C1)N1N=C(CCC1=O)C(F)(F)F | 11 | I |
| CN(C)C1=NC(=O)\C(S1)=C\C1=CC=C(F)C=C1 | 11 | I |
| ClCC1=CN(N=N1)C1=CC2=C(SC=C2)C=C1 | 11 | I |
| CC1=NN=C(Cl)N1C1=CC=C2SC=CC2=C1 | 11 | I |
| C[C@H]1CC[C@@H](C)N(CC2=C(Cl)SN=N2)C1 | 11 | I |
| C[C@@H](Cl)C1=NN=C(S1)C1=C(C)N=CS1 | 11 | I |
| C[C@@H]1CC[C@@H](C[C@@H]1C)N1C(Cl)=NN=C1C1CC1 | 11 | I |
| FC1=CN=C(Cl)N=C1SC1CCOCC1 | 11 | I |
| CC1=C(SC=N1)C1=C(CBr)N=CO1 | 11 | I |
| CC1=NC(S[C@@H]2CCCC(=O)C2)=NC(C)=C1C | 11 | I |
| FC1=C(Cl)N=C(CC2=CC=NC=C2)N=C1Cl | 11 | I |
| CC1(C)CCCN(CC2=NN=C(Cl)S2)CC1 | 11 | I |
| ClC1=NN=C(CN2CCC[C@H]3CCC[C@@H]23)S1 | 11 | I |
| C[C@H](Cl)C1=NN=C(O1)C1=C(Cl)C=CS1 | 11 | I |
| CC[C@@H]1CCC[C@](CC1)(C#N)N1CCN(C)CC1 | 11 | I |
| FC1=C(F)C=C(C=C1)C1=C(C=O)C(Cl)=NC=N1 | 11 | I |
| C[C@@H](Cl)C1=NC2=CN=CC=C2N1[C@@H]1CC1(C)C | 11 | I |
| C[C@@H]1CC2=CC(=CC=C2O1)C(=O)[C@H]1CCO[C@@H]1C | 11 | I |
| C[C@H]1OCC[C@@H]1C(=O)C1=CC=C2OCCCC2=C1 | 11 | I |
| C[C@@H]1CC[C@H](C#N)[C@@H](C1)N1C[C@@H]2CCCN2C[C@@H]1C | 11 | I |
| N#C[C@@H]1CCCCC[C@@H]1N1CCN2CCC[C@@H]2C1 | 11 | I |
| CC(C)C1=C(C)N=C(N=C1Cl)[C@@]1(C)CCCO1 | 11 | I |
| C[C@@]1(CCCO1)C(=O)[C@@H]1CCOC2(CCCC2)C1 | 11 | I |
| BrC1=C2ON=C(C2=CC=C1)C1=CC=CC=N1 | 11 | I |
| ClC1=C(CN2C=NC=N2)SC2=CC=CC=C12 | 11 | I |
| ClC1=CC=C(CC2=N[C@H]3C=NN=C3S2)C=C1 | 11 | I |
| O=C1\C(CCC\C1=C\C1=COC=C1)=C/C1=COC=C1 | 11 | I |
| CN1C=CS\C1=N/C(=O)CC1=CC=CC=C1F | 11 | I |
| FC1=CC(CN2C=NC(Cl)=CC2=O)=CC(F)=C1 | 11 | I |
| C[C@@H]1CCCCN1C(=O)C1=CC2=C(N=C3C=CC=CN3C2=O)N1C | 11 | I |
| CN1N=CC(Cl)=C1C1=NN2C=NC3=C(C4=C(CCC4)S3)C2=N1 | 11 | I |
| C1CCC2=C(CC1)C1=C(S2)N=CN2N=C(N=C12)C1=NC=CN=C1 | 11 | I |
| C[C@H]1CN(C[C@H](C)O1)C1=NC(=O)\C(S1)=C\C1=CC=C2OCOC2=C1 | 11 | I |
| C[C@H](CC(=O)N1N=NC2=CC=CC=C12)N1N=C(C)C(Cl)=C1C | 11 | I |
| CN1C=CC=CC1=C(C#N)C1=NC(=CS1)C1=CC=CC(=C1)[N+]([O-])=O | 11 | I |
| CC1=NN=C(CSC2=NC3=CC=CC=C3C3=NC4=CC=CC=C4N23)O1 | 11 | I |
| CC1=C(C=CO1)C1=NN=C(O1)SC1=CS(=O)(=O)C2=C1C=CC=C2 | 11 | I |
| FC1=CC=C(C=C1)S(=O)(=O)N1CCC[C@H]1C1=NN=C2C=CC=CN12 | 11 | I |
| C[C@H]1C[C@@H]1C(=O)N1CCC(CC1)C1=NC2=NC=NN2C(=C1)C(F)F | 11 | I |
| CN1C2=C3COCC(C)(C)N3C(=C2C(=O)N(C)C1=O)C1=CC=C(C)C=C1 | 11 | I |
| CCC1=NOC(CN2C=NC3=C(C4=C(C[C@H](C)CC4)S3)C2=O)=N1 | 11 | I |
| O=C1CCCC[C@@H]1SC1=NN=C(O1)C1=CC2=C(OCO2)C=C1 | 11 | I |
| C[C@@H]1C=C(N=N1)C1=NN2C(S1)=NN=C2C1=CC=C(Cl)C=C1 | 11 | I |
| CC(=O)C1=C(C)C2=C(S1)N=CN(CC1=CC=C3OCOC3=C1)C2=O | 11 | I |
| CC(C)N1N=CC2=C1N=C(N=C2C1=CN(C)N=C1)C1=CC=CC=C1 | 11 | I |
| CN(C)C(=O)C1=NN=C2SC(=NN12)C1=CC=C(Br)C=C1 | 11 | I |
| C[C@H]1CC2=CC(=CC=C2N1S(C)(=O)=O)C(=O)\N=C1\SC=CN1C | 11 | I |
| CCC1=NC(CN2C=NC3=C(C=C(Cl)C=C3Cl)C2=O)=NO1 | 11 | I |
| CC1=C(OC2=C1C=CC=C2C)C(=O)[C@@H](C#N)C1=NN=C2CCCCCN12 | 11 | I |
| CC1=CC2=NN=C(SCC3=CC=CC4=C3OCCO4)N2C(C)=N1 | 11 | I |
| CN(C(=O)C1=NN2C(=N1)N=CC=C2C)C1=CC=C(C=C1)C(C)(C)C | 11 | I |
| C[C@H]1CN(C[C@@H](C)O1)C(=O)C1=NC(=CC=C1Cl)N1N=C(C)C=C1C | 11 | I |
| C[C@H]1CN(C[C@@H](C)O1)C(=O)C1=CN=C(S1)C1=CC=C2OCOC2=C1 | 11 | I |
| CC1=CN(C[C@H]2CCCN2C(=O)C2=CC3=C(OCO3)C(Cl)=C2)N=C1 | 11 | I |
| FC(F)(F)C1=CN(N=C1)C1=NC(=NC(Cl)=N1)N1CCCC1 | 11 | I |
| CC(C)C1=CSC(=N1)C1=NN2C(S1)=NN=C2[C@@H]1CCCO1 | 11 | I |
| CC1=C2C(=NO1)C(=O)N(CN1CCCC3=C1C=C(F)C(F)=C3)N=C2C | 11 | I |
| C[C@H](N1CCN(CC1)C1=NN2C(C)=NN=C2C=C1)C1=CC=C(F)C=C1 | 11 | I |
| COCC1=NC(N2C[C@]3(C)C[C@H]2CC(C)(C)C3)=C2C=NN(C)C2=N1 | 11 | I |
| ClC1=CC(CN2N=NC(=N2)C2=CSC=C2)=CC2=C1OCCO2 | 11 | I |
| CC1=CC=CC2=NC(CN3C(=O)OC4=CC(Cl)=CC=C34)=CC(=O)N12 | 11 | I |
| [O-][N+](=O)C1=CC(\C=C2\C(=O)C3=CC=CC=C3S2(=O)=O)=CC=C1 | 11 | I |
| CN1C(=O)N(C(=O)C=C1C(F)(F)F)C1=C2OC(C)(C)C(=O)C2=CC=C1 | 11 | I |
| CCC1=C(SN=N1)C(=O)N1CCC[C@@H]1C1=C(Cl)N(C)N=C1C | 11 | I |
| O=C1C=C(CN2CCC[C@H]2C2=CC=C3OCOC3=C2)N=C2C=CC=CN12 | 11 | I |
| ClC1=CN=C(SC2=NC(=NC(Cl)=N2)N2C=CN=C2)C=C1 | 11 | I |
| CC1=C(C)C(=NN2C1=NN=C2C(F)(F)F)N1CC[C@@]2(CCOC2)C1 | 11 | I |
| C1CO[C@@H](C1)C1=NC=C(S1)C1=NC(=NO1)C1=CC2=C(OCC2)C=C1 | 11 | I |
| CN(C(=O)C1=C2C(C)=NN(C)C2=NC(C)=C1)C1=CC=CC2=C1C=CC=N2 | 11 | I |
| C[C@H](N1CCCC2(C1)OCCO2)C1=NN=C(O1)C1=CC=C(C)C(C)=C1 | 11 | I |
| IC1=C(N=CN=C1)N1N=NC(=N1)C1=CC=CC=C1 | 11 | I |
| C(N1CCCC[C@@H]1C1=NN=C2CCCCN12)C1=C2OCOC2=CC=C1 | 11 | I |
| [O-][N+](=O)C1=NN(C=C1Br)[C@@H]1CCO[C@]2(CCSC2)C1 | 11 | I |
| CC1=NOC(=N1)[C@@H]1CCCN(C1)C1=C2C(SC3=C2CCC3)=NC=N1 | 11 | I |
| C1CCN(C1)C1=NN=C(SC2=NC3=C(C=C2)N=CC=N3)S1 | 11 | I |
| CN(C)[C@H]1CC[C@H]2[C@H](C1)OCCN2C(=O)C1=NOC(=C1)C(C)(C)C | 11 | I |
| CC1(C)O[C@H]2O[C@H]3[C@H]4[C@H](OC5=CC=CC=C5)C(=O)N4CCC[C@H]3[C@H]2O1 | 11 | I |
| CC1(C)O[C@H]2O[C@H]3[C@H]4[C@H](OC5=CC=CC=C5)C(=O)N4CCC[C@@H]3[C@H]2O1 | 11 | I |
| CN1N(C(=O)C(\N=C/C2=CC=C3OCOC3=C2)=C1C)C1=CC=CC=C1 | 11 | I |
| CCOC(=O)C1=NN2C=NC3=C(C4=C(COC(C)(C)C4)S3)C2=N1 | 11 | I |
| C[C@@H]1N(CCN2C=CC=C12)S(=O)(=O)C1=CC=C(C=C1)[N+]([O-])=O | 11 | I |
| C1OC2=CC=CC=C2O[C@H]1C1=NN=C2SC(=NN12)C1=CC=NC=C1 | 11 | I |
| C1OC2=CC=CC=C2O[C@@H]1C1=NN=C2SC(=NN12)C1=CC=NC=C1 | 11 | I |
| CN(C)C(=O)C1=C(C)C2=C(S1)N=CN1N=C(N=C21)C1=CC=NC=C1 | 11 | I |
| C[C@H]1CC2=CC=CC=C2N1C(=O)CN1N=C(OC1=O)C1=C(C)OC=C1 | 11 | I |
| FC(F)(F)C1=NN(CN2N=NC3=CC=CC=C23)C(=O)N2C=CC=C12 | 11 | I |
| CN1N=CC2=C1N=CN=C2N1CCC[C@@H]1C1=CC=C2OCCOC2=C1 | 11 | I |
| CC1=NON=C1[C@H]1CCCN1C(=O)C1=CC=C2N=C(OC2=C1)C1CC1 | 11 | I |
| CN1C(=NC2=CC=CC=C12)[C@H](C#N)C1=C(Cl)N=C(C#N)C(=N1)C#N | 11 | I |
| COC1=CC=C(C=C1)N1C=NC2=C(C)N=C(N=C12)N1N=C(C)C=C1C | 11 | I |
| CN1C2=C(N(CC(=O)C3=CC=C(C)C(C)=C3)C=N2)C(=O)N(C)C1=O | 11 | I |
| C[C@H]1CN(C[C@H](C)O1)C1=CC(C)=NC2=CC(=NN12)C1=NC=CN1C | 11 | I |
| CCC1=CC2=C(S1)N=CN(CN1N=NC3=CC=CC=C3C1=O)C2=O | 11 | I |
| C[C@H]1CN(C[C@@H](C)O1)C(=O)C1=NN(CN2N=C(C)C(Cl)=C2C)C=C1 | 11 | I |
| COC(=O)C1=CC(=NC2=C(Cl)C(C)=NN12)C1=C(C)N(C)N=C1C | 11 | I |
| O=C1OC2=CC=C(C=C2C=C1)S(=O)(=O)N1N=NC2=CC=CC=C12 | 11 | I |
| CC1=NC2=NC(SCC3=CC(=O)N4C=CC=CC4=N3)=NN2C(C)=C1 | 11 | I |
| CN1C(=CC2=C1N=C1C=CC=CN1C2=O)C(=O)N1COCC1(C)C | 11 | I |
| CN1N=C(C)C2=C1C=NN2CC1=CC=C(O1)C(=O)N1CCCCC1 | 11 | I |
| O=C1OC(=NC2=CC3=C(OCCCO3)C=C12)C1=CC2=C(OCCO2)C=C1 | 11 | I |
| ClC1=NC(=NC(=N1)N1CCS(=O)(=O)CC1)N1CCCC1 | 11 | I |
| CN([C@H]1CCS(=O)(=O)C1)C1=NC(=NC(Cl)=N1)N1CCCC1 | 11 | I |
| CC1=C(CN2N=CN3N=C(C=C3C2=O)C2=CC=C(F)C=C2)C(C)=NO1 | 11 | I |
| ClC1=NC(=NC(OC2=CC(Br)=CN=C2)=N1)N1C=CN=C1 | 11 | I |
| ClC1=C(Cl)C=C(C=C1)N1C=CC2=C(N=NC3=NC=NN23)C1=O | 11 | I |
| CC(C)C1=CC(=NC2=NC=NN12)C(=O)N1C[C@H]2[C@@H]3CC[C@@H](O3)[C@H]2C1 | 11 | I |
| CC1=CC(=NO1)[C@H]1CCCN1C1=CC(=NC2=NC=NN12)C(F)(F)F | 11 | I |
| CN([C@@H]1CCCN(C1)C1=CC=C(C)N=N1)C1=NN2C=C(C)N=C2S1 | 11 | I |
| C(N1CCC(CC1)C1=NN=C2CCCCCN12)C1=CC=CC2=NON=C12 | 11 | I |
| CC(C)C1=NC(=NO1)[C@@H]1CCCN(C1)C1=C(C#N)C(C)=C(C)N=N1 | 11 | I |
| C1COC2=CC=C(C=C2OC1)C1=NN2C(=N1)N=CC=C2C1=CC=CC=N1 | 11 | I |
| C[C@@H]1COC2=CC=CC=C2N1C(=O)C1=CC=CC(=C1)N1C=NN=N1 | 11 | I |
| CC1=NC(=NN1C1=CC=CC=C1Cl)C1=NC(=NO1)C1=CC=NC=C1 | 11 | I |
| CN1N=CC2=C(N=CN=C12)N1CCCC[C@@H]1CN1N=C(C)C=C1C | 11 | I |
| CN(C(=O)C1=CN=C(S1)C1=CC=C2OCOC2=C1)C1=CN(C)N=C1 | 11 | I |
| O=C(\C=C\N1C(=O)OC2=CC=CC=C12)N1C(=O)OC2=CC=CC=C12 | 11 | I |
| CN1N=CC(Cl)=C1C1=NN=C2SC(=NN12)C1=CC=NC=C1 | 11 | I |
| CC1=CC=C(O1)[C@H]1COCCN1C(=O)C1=CC=C(C=C1C)[N+]([O-])=O | 11 | I |
| [O-][N+](=O)C1=CN(CC2=CN3C=C(Cl)C=C(Cl)C3=N2)C=CC1=O | 11 | I |
| ClC1=NC(=NC(=N1)N1C=NC=N1)C1=C(Br)C=CO1 | 11 | I |
| CN1C=C(N=C1Cl)S(=O)(=O)N1CCOC2=C1C=C(C)C=N2 | 11 | I |
| CC1=CC=C(N=N1)C1=NOC(=N1)C1(CCCCC1)N1CCOCC1 | 11 | I |
| CN1N=C(C)C(OS(=O)(=O)C2=C(Cl)N=C3C=CC=CN23)=C1C | 11 | I |
| CC1=NN(C(C)=C1)C1=CC=C(C=N1)C1=NOC(=N1)C1=COCCC1 | 11 | I |
| FC1=CN=C(N=C1)N1CCO[C@]2(C1)COCCN(C2)C1=CN=CC=C1 | 11 | I |
| CN(C)C1=CC=C(N=C1)N1CCC[C@]2(C1)CN(CCO2)C1=CN=CC=N1 | 11 | I |
| O=C1\C(COC2=CC3=C(OCO3)C=C12)=C/C1=CC=C(C=C1)N1C=NC=N1 | 11 | I |
| CN1C=C([C@@H]2CC(=O)N(C2=O)C2=CC=CC(=C2)[N+]([O-])=O)C2=C1C=CC=C2 | 11 | I |
| FC1=C(Cl)C=C(C=C1)N1C=C(CN2C=NC(C#N)=C2C#N)N=N1 | 11 | I |
| CN1N=C(\C=C2\C(=O)N(N=C2C2=CC=CO2)C2=CC=CC=C2)C=C1C | 11 | I |
| COC1=CC(\C=C2/N=C(OC2=O)C2=CC=CS2)=CC2=C1OCO2 | 11 | I |
| CC1=NN(C(=O)C(C)(C)C)C(C)=C1S(=O)(=O)N1CCCCC1 | 11 | I |
| CN1N=CC2=C(SCC3=CC(=O)OC4=CC(C)=CC=C34)N=CN=C12 | 11 | I |
| O=S(=O)(N1C=NC2=CC=CC=C12)C1=CC=CC2=NSN=C12 | 11 | I |
| ClC1=CC=C(C=C1)C(=O)CN1N=C2C=CC(Cl)=NN2C1=O | 11 | I |
| FC1=CC=C(C=C1)C1=NN(CC2=C(Cl)SN=N2)C(=O)O1 | 11 | I |
| CN(CC1=CN(C)N=C1)C1=NC2=CC(C)=NN2C2=CC=CC=C12 | 11 | I |
| FC(F)(F)C1=CC=CC(=C1)C1=NN=C2C=CC3=NN=CN3N12 | 11 | I |
| C[C@H]1OC2=CC=CC=C2C=C1\C=C1\SC2=NC(=O)C(C)=NN2C1=O | 11 | I |
| CN1C(=O)N([C@H](N2C=NC=N2)C1(C)C)C1=CC=CC2=C1C=CC=C2 | 11 | I |
| CN1C(=S)S[C@](C)([N+](\[O-])=C\C2=CC=C(C=C2)[N+]([O-])=O)C1(C)C | 11 | I |
| CC1=C[C@H](N=N1)[C@@H]1N=N[C@H](S)N1\N=C\C1=CC=CC=C1F | 11 | I |
| C[C@@H]1CCC2=C3C(S[C@H]2C1)=NC(=O)N1N=C(N=C31)C1=CC=NC=C1 | 11 | I |
| CN1C(=NC2=CC=CC=C12)[C@@H]1CN(C(=O)C1)C1=CC=C2OCOC2=C1 | 11 | I |
| ClC1=CC=CC=C1C1=NN=C2SC(CN3C=CC=N3)=NN12 | 11 | I |
| C=CCN1C(=O)\C(SC1=C(C#N)C#N)=C/C1=CC=CC2=C1OCO2 | 11 | I |
| C=CCN1C(=O)\C(SC1=C(C#N)C#N)=C\C1=CC=CC2=C1OCO2 | 11 | I |
| O=C1OC2=CC(OS(=O)(=O)C3=CC=C(C=C3)C#N)=CC=C2C=C1 | 11 | I |
| ClC1=CC=C2N(CC#C)\C(SC2=C1)=N\C(=O)CN1C=NC=N1 | 11 | I |
| CC(=O)N1N=C(O[C@@H]1C1=CC=C(F)C=C1)C1=CC=C2OCOC2=C1 | 11 | I |
| CC1=CC=C(S1)C1=NC(=NO1)C1=CC=C(N=C1)N1C=CN=C1 | 11 | I |
| ClC1=CC=C(O1)C(=O)N1CCC[C@@H]1C1=NN=C2CCCCCN12 | 11 | I |
| C1CCN(C1)C1=NC(=NC=C1)N1CCCN(CC1)C1=NC=CS1 | 11 | I |
| COC1=NC=C(C2=CC(C)=NO2)C(=N1)C1=CC(Cl)=CC=N1 | 11 | I |
| CN1C=NC2=C1C(=O)N(CC1=CC=C(Cl)S1)C(=O)N2C | 11 | I |
| C[C@H](N(C)C(=O)C1=CN2C=NN=C2C=C1)C1=CC2=CC=CC=C2O1 | 11 | I |
| CC1=CC=CN2C(=O)C=C(CN3N=CC(Cl)=C(Cl)C3=O)N=C12 | 11 | I |
| CC1=CC=NC2=NC(=NN12)C(=O)OC1=CC=C(Br)C=C1 | 11 | I |
| CN1N=CC(Cl)=C1C1=NN2C(S1)=NN=C2C1=CC=CS1 | 11 | I |
| CC1=NC2=NC=NN2C2=C1C(=O)N(C=C2)C1=CC=C(F)C(Cl)=C1 | 11 | I |
| C1CCC2=C(C1)C=NC1=NC(=NN21)C1=CC2=C(OCCO2)C=C1 | 11 | I |
| COC1=CC=C2OC(C)=C(C3=NC(=NO3)C3=CC=NC=C3)C2=C1 | 11 | I |
| CN(C(=O)C1=CN(N=N1)C1=CC=C(F)C=C1)C1=CC=C(Cl)C=N1 | 11 | I |
| C[C@H](SC1=NN2C(=N1)N=C(C)C(C)=C2C)C(=O)N1CCCCC1 | 11 | I |
| CN(CC1=CC=CC2=C1C=CC=N2)C1=C2C(C)=NOC2=NC(C)=N1 | 11 | I |
| C[C@@H]1CCN([C@@H](C)C1)C(=O)C1=CC(=CC=C1N(C)C)[N+]([O-])=O | 11 | I |
| FC1=CC=C2C(=O)N(CC3=NOC(=N3)C3=CC=CC=C3)C=NC2=C1 | 11 | I |
| ClC1=NC(=NC(=N1)N1C=CN=C1)C1=C(Br)C=CO1 | 11 | I |
| C[C@H]1CN(CC2CCN(CC2)C2=NN3C=CN=C3C=C2)C[C@H](C)O1 | 11 | I |
| CC(C)C1=NOC(=N1)C1CCN(CC1)C1=NC(C)=NC(C)=C1C | 11 | I |
| CC1=C(CN2N=C3C=CC=CN3C2=O)N=C(O1)C1=CC=CC=C1 | 11 | I |
| CN(CC1=C(C)ON=C1C)C1=C2CC3=CC=CC=C3OCC2=NC=N1 | 11 | I |
| CN(C)C(=O)N1C(=CC2=C1C1=CC=CC=C1N2C)C(=O)N(C)C | 11 | I |
| CN1N=C(C)C2=CC(=CN=C12)C(=O)O[C@H]1[C@@H]2CCO[C@@H]2C1(C)C | 11 | I |
| CN1N=C(C)C2=CC(=CN=C12)C(=O)O[C@H]1[C@H]2CCO[C@@H]2C1(C)C | 11 | I |
| CN(C(=O)C1=CC=CC=C1N1C=CN=N1)C1=CC=C2OCCC2=C1 | 11 | I |
| O=C1SCC(=N1)N1N=C(C[C@@H]1C1=CC=CO1)C1=CC=CO1 | 11 | I |
| CC1=CC=C(C=C1)N1C(=O)[C@@H]2N=NN(CC3=CC=CC=C3F)[C@H]2C1=O | 11 | I |
| CC1=CC=C(C=C1C)N1N=N[C@@H]2[C@H]1C(=O)N(C2=O)C1=CC=C(F)C=C1 | 11 | I |
| CC1=CC(C)=C2S\C(=N/C(=O)C3=COCCO3)N(CC=C)C2=C1 | 11 | I |
| BrC1=CC=C(O1)C=C1C(=O)OC2(CCCCC2)OC1=O | 11 | I |
| CN(C)C1=C2N=CN=C2N(CC2=C(Cl)C=CC=C2Cl)C=N1 | 11 | I |
| COC1=CC=C(C=C1OC)C1=CC2=CC3=C(OC(C)=C3C)C=C2OC1=O | 11 | I |
| CC1=CC=C2C(COC3=CC=C4C=CC(=O)OC4=C3)=CC(=O)OC2=C1C | 11 | I |
| CC1=C(C=CO1)C1=NN=C2SC(=NN12)C1=CC=C2C=CC=CC2=C1 | 11 | I |
| CC1=C(C=CO1)C1=NN=C2SC(CC3=CC=C(C)C(C)=C3)=NN12 | 11 | I |
| CC1=C(C=CO1)C1=NN=C2SC(CC3=CC=CC4=C3C=CC=C4)=NN12 | 11 | I |
| COC1=CC=C(C=C1)C1=NN2C(SC1)=NN=C2C1=CC=CC=C1F | 11 | I |
| CC1=CC=C(\C=C\C2=NN3C(S2)=NN=C3C2=CC=CO2)C=C1C | 11 | I |
| CC1=CC=CC=C1OCC1=NN2C=NC3=C(C4=C(CCCC4)S3)C2=N1 | 11 | I |
| CC1=CC(=NO1)C(=O)\N=C1\SC2=CC(Cl)=CC=C2N1CC#C | 11 | I |
| CC1=C(N=NN1C1=CC(C)=CC(C)=C1)S(=O)(=O)C1=CC=C(C)C=C1 | 11 | I |
| CC1=CC=C(OC2=C3C=NN(C3=NC=N2)C2=CC=C(F)C=C2)C=C1 | 11 | I |
| CCN1\C(SC2=C1C(Cl)=CC=C2)=N\C(=O)C1=COCCO1 | 11 | I |
| CCN1\C(SC2=CC=CC=C12)=C\C=C1C(=O)OC(C)(C)OC1=O | 11 | I |
| CN1\C(SC2=CC(Br)=CC=C12)=N/C(=O)C1=CC(C)=NO1 | 11 | I |
| CC1=C(C)C2=C(N=C(C)N3C4=CC=CC=C4N=C23)N1CC1=CC=CO1 | 11 | I |
| CC(=C)CSC1=C2OC3=NC4=C(COC(C)(C)C4)C=C3C2=NC=N1 | 11 | I |
| CCN1\C(SC2=C(C)C=C(C)C=C12)=N\C(=O)C1=COCCO1 | 11 | I |
| CCN1\C(SC2=CC(Cl)=CC=C12)=N\C(=O)C1=NOC(C)=C1 | 11 | I |
| COC1=CC=CC=C1\C=C1\SC2=NN=C(N2C1=O)C1=CC=C(C)C=C1 | 11 | I |
| CCN1N=CC=C1C(=O)\N=C1/SC2=CC(Cl)=CC=C2N1CC | 11 | I |
| CCN1N=C(C=C1C)C(=O)\N=C1\SC2=CC(F)=CC=C2N1C | 11 | I |
| O=C(\N=C1/SC2=CC=CC=C2N1CC#C)C1=CC=C2OCOC2=C1 | 11 | I |
| C[C@@H]1C[C@@H](C)CN(C1)C1=NC=NC2=C1C=NN2C1=CC(C)=CC(C)=C1 | 11 | I |
| FC(F)(F)C1=CC(=CC=C1)N1N=NN=C1C1=NC2=CC=CC=C2C=C1 | 11 | I |
| ClC1=CC=C(C=C1)C1=NN2C(=NN=C2C2=CC=CC=C12)[C@@H]1CCCO1 | 11 | I |
| CC1=CC(=O)OC2=C1C=C1C(OC=C1C1=CC=C3OCCCOC3=C1)=C2 | 11 | I |
| CC1=NC2=NC(CSC3=NC4=CC(Cl)=CC=C4O3)=CN2C(C)=C1 | 11 | I |
| CN1N=CC=C1C(=O)\N=C1\SC2=C(N1C)C(C)=CC=C2Cl | 11 | I |
| CN1N=CC(C)=C1C(=O)\N=C1\SC2=CC(C)=CC(C)=C2N1C | 11 | I |
| CC1=NN=C2SC(=NN12)C1=CC=C(O1)C1=CC=CC(Cl)=C1Cl | 11 | I |
| COC1=CC=C(Br)C=C1N1N=NN=C1C1CCCCC1 | 11 | I |
| COC1=CC=CC=C1C1=NN=C2SCC(=NN12)C1=CC=CC=C1 | 11 | I |
| CC1=C2OC(=O)C3=C(CCCC3)C2=CC2=C1OCN(CC1=CC=CO1)C2 | 11 | I |
| CC1=CC=C(C=C1C)C1=NC2=NN=NN2[C@@H](C1)C1=CC=C(Cl)C=C1 | 11 | I |
| CC1=NOC(CN2C=NC3=C(C(=CS3)C3=CC=C(C)C(C)=C3)C2=O)=C1 | 11 | I |
| ClC1=NC(=NC(=N1)N1CCCSCC1)N1CCCCC1 | 11 | I |
| C[C@H]1CCC[C@@H](C1)SC1=NC(=NC(Cl)=N1)N1CCOCC1 | 11 | I |
| CCC1=NC2=CC(=CC=C2O1)C1=NOC(=N1)C1=CC(F)=CC=C1F | 11 | I |
| C[C@@H]1CCN([C@@H](C)C1)C(=O)C1=CSC(=N1)C1=CC=C2OCOC2=C1 | 11 | I |
| CCC[C@H]1[C@H](C)CCCN1C1=C2C(Br)=NN(C)C2=NC=N1 | 11 | I |
| CCC1=CC=C(C=C1)[C@H]1CC(=NC2=NN=NN12)C1=CC=C(C)C=C1 | 11 | I |
| CC[C@H](C)[C@@H]1CCCCN1C1=C2C(Br)=NN(C)C2=NC=N1 | 11 | I |
| C\C(\C=C1/N=C(OC1=O)C1=CC=C2OCOC2=C1)=C\C1=CC=CC=C1 | 11 | I |
| CC(C)C1=CC(C(=O)N(C)C2=CC=C(F)C(F)=C2)=C2C(C)=NOC2=N1 | 11 | I |
| CC1=CC=C(C=C1C)C1=NC2=NC=NN2\C1=N/CC1=CC=CC=C1 | 11 | I |
| CN1C=CC(=N1)C(=O)\N=C1/SC2=CC(Cl)=CC=C2N1CC#C | 11 | I |
| CCN1C=CC(=N1)C(=O)\N=C1/SC2=CC(C)=C(C)C=C2N1C | 11 | I |
| CCN1C(=O)C2=CC=CC=C2N=C1\C=C\C1=CC=C2OCOC2=C1 | 11 | I |
| CC1=NN2C(=N1)C1=CC=CC=C1N=C2SCC1=CC=C(C=C1)C#N | 11 | I |
| CC1=NC2=NC(SCC3=NC4=CC=CC=C4S3)=NN2C(C)=C1 | 11 | I |
| BrC1=CC=C2N(C(=O)OC3=CC=CC=C3)C(=O)OC2=C1 | 11 | I |
| CC1=CC=C(O1)C1=NN2[C@H](C1)C1=CC=CC=C1O[C@@H]2C1=CC=CC=N1 | 11 | I |
| CC1=NC2=C(C3=C(CCC3)S2)C(SC2=NN=C3C=CC=CN23)=N1 | 11 | I |
| CC1=CC(=O)OC2=C1C=CC(OS(=O)(=O)C1=CC=CC=C1)=C2 | 11 | I |
| COC1=CC=C2C=C(C(=O)OC2=C1)S(=O)(=O)C1=CC=C(C)C=C1 | 11 | I |
| FC1=CC=C(C=C1)N1N=CC2=C(OC3=CC(F)=CC=C3)N=CN=C12 | 11 | I |
| O=C(CN1N=NC2=CC=CC=C2C1=O)C1=CC=CC2=CC=CC=C12 | 11 | I |
| FC1=CC=CC=C1OCC1=NN=C2S[C@H]3CCCCC3=NN12 | 11 | I |
| CSC1=NC2=CCCC(=O)[C@@H]2[C@@H](C1C#N)C1=CC=C2OCOC2=C1 | 11 | I |
| C[C@@H]1CSC(=N1)N(C(=O)C1=NOC(C)=C1)C1=CC=C(Cl)C=C1 | 11 | I |
| C[C@@H]1CCCN(C1)C1=NC=C(C2=CC=NC=C2)C(=N1)C1=C(C)OC=C1 | 11 | I |
| CC1=C(C)C2=C(N=C(C)N3C4=CC=CC=C4N=C23)N1C[C@@H]1CCCO1 | 11 | I |
| C[C@H]1CN(CC2=CSC(=N2)C2=CC=C3OCOC3=C2)C[C@H](C)O1 | 11 | I |
| FC1=CC=C(C=C1)S(=O)(=O)OC1=CC2=C(C=CC(=O)O2)C=C1 | 11 | I |
| COC1=CC=C(C=C1)C1=NN2C(SC1)=NN=C2C1CCCCC1 | 11 | I |
| COC1=C2[C@H](N3C=CC4=C3C=CC=C4)N(C)CCC2=CC2=C1OCO2 | 11 | I |
| CSC1=NC2=C(N=N1)C1=CC(Br)=CC=C1N=CO2 | 11 | I |
| O=C1[C@@H](CCC#N)C(=NC2=NC3=CC=CC=C3N12)C1=CC=CC=C1 | 11 | I |
| CC1=NC2=NC=NN2C(OC2=CC=C(I)C=C2)=C1 | 11 | I |
| CC(C)(C)N1C=C(\C=C2/N=C(OC2=O)C2=CC=C(F)C=C2F)C=N1 | 11 | I |
| CC1=C(C)C2=C(S1)N=CN(\N=C\C1=CC3=CC=CC=C3OC1)C2=O | 11 | I |
| COC1=C2OC[C@H](CC2=CC=C1)N1C=C(N=N1)C1=C(C)C=CS1 | 11 | I |
| FC1=CC=C(CN2N=CC3=C2CN(C3)C2=NC=C(Cl)C=N2)C=C1 | 11 | I |
| CC(=O)N(C(C)=O)C1=C(C2=CC=CC=C2N1C(C)=O)C1=CC=CC=C1 | 11 | I |
| BrC1=CC=CC=C1C1=NC2=CC=C(C=C2O1)N1C=NN=C1 | 11 | I |
| [O-][N+](=O)C1=CC=C(O1)C(=O)C1=CC(F)=C(Br)C=C1F | 11 | I |
| CN1C=NC2=C1C=CC(OS(=O)(=O)C1=CC=C(Cl)S1)=C2 | 11 | I |
| CC(C)[C@@H]1CCCN(CC1)S(=O)(=O)C1=C(Cl)N(C)C=N1 | 11 | I |
| CN1CCCN(CC1)C1=C(N=C(O1)C12CC3CC(CC(C3)C1)C2)C#N | 11 | I |
| C[C@H]1C[C@@H]2[C@@H](N(N=C2\C(C1)=C/C1=CC=CO1)C(C)=O)C1=CC=CO1 | 11 | I |
| CC1=CC(=CC=C1F)S(=O)(=O)OC1=CC=C2C=CC(=O)OC2=C1 | 11 | I |
| CN(C1=CC=C(Cl)C=N1)S(=O)(=O)C1=CN=C(Cl)C(C)=C1 | 11 | I |
| C[C@H]1C[C@H]2CCCC[C@@H]2N1C(=O)C1=C2C(C)=NN(C)C2=NC(=C1)C1CC1 | 11 | I |
| COC1=CC=C(C=C1Br)C1=NN=C2N1C=CN=C2Cl | 11 | I |
| [O-][N+](=O)C1=CC(Br)=C(CN2CCCC[C@H]2C#N)C=C1 | 11 | I |
| CN(C(=O)C1=CC=C2C=CC=CC2=N1)C1=CC=C(N=N1)C1=CC=CC=C1 | 11 | I |
| [O-][N+](=O)C1=CC(Br)=C(C[C@@]2(C[C@H]3CC[C@@H]2O3)C#N)C=C1 | 11 | I |
| C[C@@H]1CCCCN1C(=O)[C@H]1CSC2=NC(=CC(=O)N2C1)C(C)(C)C | 11 | I |
| FC1=CC=C(CC2CCN(CC2)C(=O)C2=CC3=NON=C3C=C2)C=C1 | 11 | I |
| BrC1=CN2C=CN=C2C(SC2=NC3=CC=CC=C3O2)=N1 | 11 | I |
| COC1[C@H]2CC[C@@H]1CN(C2)C1=C2CC3=CC=CC=C3OCC2=NC=N1 | 11 | I |
| BrC1=CC2=C(C=C1)N(CC1=NOC3=C1CCCC3)C(=O)O2 | 11 | I |
| COC1=CC=C2SC(C(=O)N(C)C3=C(C)ON=C3C)=C(C)C2=C1 | 11 | I |
| CN1N=CC(C(=O)\N=C2/SC=CN2CC2=CC=CC=C2F)=C1C | 11 | I |
| CC1=C(C(=O)N=S(C)(=O)C2=CC=CC=C2)C(=NO1)C1=CC=CC=C1 | 11 | I |
| CN1C(=NC2=CC=CC=C12)[C@H](C#N)C(=O)C1=C(C)C2=CC=CC=C2O1 | 11 | I |
| CC1=NOC(=C1)C(=O)\N=C1\SC2=CC(F)=CC=C2N1CC#C | 11 | I |
| BrC1=CC=C(\C=C2/N=C(OC2=O)C2=CC=C3OCOC3=C2)C=C1 | 11 | I |
| ClC1=CC=C(C=C1)C1=C2C=C(C=CC2=NO1)N1C=CC=C1C1OCCO1 | 11 | I |
| CC1=C(C2=N\C(=C/C3=CC=C(Cl)C=C3)C(=O)O2)C(=NO1)C1=CC=CC=C1 | 11 | I |
| CC1=C(C=CO1)C1=NN=C2SC(=NN12)C1=CC=CC=C1Br | 11 | I |
| CN1\C(=C/C=C(\C#N)C2=NN=C3CCCCCN23)C(C)(C)C2=CC=CC=C12 | 11 | I |
| CC1=CC=CC=C1C1=NC2=C(N=C3C=CC=CC3=N2)N1C[C@@H]1CCCO1 | 11 | I |
| COC1=NC2=CC=CC=C2C2=NN=C(N12)C1=CC=C(Br)C=C1 | 11 | I |
| BrC1=CC=CC(\C=C2/N=C(OC2=O)C2=CC=C3OCOC3=C2)=C1 | 11 | I |
| S(C1=NC2=CC=CC=C2S1)C1=CC(=NC2=NC=NN12)C1=CC=CC=C1 | 11 | I |
| CC1=CC=C(C=C1)S(=O)(=O)OC1=CC=C2C(OC(=O)C3=CC=CC=C23)=C1 | 11 | I |
| CCOC(=O)C1[C@H]2C3=CC(Cl)=CC(Cl)=C3OC(=O)C2=C(C)N=C1C | 11 | I |
| CC1=CC=C(C=C1)N1N=NC2=C(SC3=NC(C)=C(Cl)C(C)=C23)C1=O | 11 | I |
| C1CCN2C(CC1)=NN=C2C1=CC(=NN1C1=CC=CC=C1)C1=CC=CC=C1 | 11 | I |
| C1SC2=NN=C(N2N=C1C1=CC=C(C=C1)C1=CC=CC=C1)C1=CC=NC=C1 | 11 | I |
| C[C@@H]1CCC2=C(C1)SC1=C2C(OC2=CC=C(C=C2)N2C=CN=C2)=NC=N1 | 11 | I |
| CC1=NN(C(C)=C1\C=C1/N=C(OC1=O)C1=CC=CC=C1)C1=CC=C(C)C=C1C | 11 | I |
| CN1N=C(C=C1C)C(=O)\N=C1/SC2=C(N1C)C(Br)=CC=C2 | 11 | I |
| CN(C)C1=CC=C(C=C1)[C@@H]1CC(=O)C2=C(O1)C1=C(OC(=O)C=C1C)C=C2C | 11 | I |
| CCN1N=CC=C1C(=O)\N=C1\SC2=C(N1C)C(Cl)=C(Cl)C=C2 | 11 | I |
| CCN1N=C(C)C=C1C(=O)\N=C1\SC2=C(C)C=C(C)C=C2N1CC#C | 11 | I |
| ClC1=CC(Cl)=C(CN2C=CS\C2=N/C(=O)C2=CN=CC=N2)C=C1 | 11 | I |
| ClC1=CC=C(Cl)C(=C1)S(=O)(=O)OC1=CC2=C(C=CC(=O)O2)C=C1 | 11 | I |
| CCN1\C(SC2=CC(F)=CC=C12)=N\C(=O)C1=COC2=CC=CC=C2C1=O | 11 | I |
| ClC1=CC=CC(C2=NN=C3SC(=NN23)C2=CC=CO2)=C1Cl | 11 | I |
| CC1=CC(=O)N2N=C(OC3=CC(Cl)=C(Cl)C=C3Cl)SC2=N1 | 11 | I |
| ClC1=CC2=C(OC(=O)C3=C2CCCC3)C=C1OC(=O)C1=CC=CO1 | 11 | I |
| O=C1OC2=C(C=C1C1=NN=C(O1)C1=CC=CS1)C1=CC=CC=C1C=C2 | 11 | I |
| [O-][N+](=O)C1=CC=C(OC2=C(Br)C=C(F)C=C2)C2=C1C=NC=C2 | 11 | I |
| C[C@H]1CC2=CC(=CC=C2O1)C1=CC(CC(=O)N2C[C@@H](C)C[C@@H](C)C2)=NO1 | 11 | I |
| CC(C)N1N=C(C=C1C)C(=O)\N=C1/SC2=CC(C)=CC=C2N1CC#C | 11 | I |
| CN1C=C(C)C(=N1)C(=O)\N=C1\SC2=C(Cl)C=CC(Cl)=C2N1C | 11 | I |
| CC1=CC=C2N(N=NC2=C1)S(=O)(=O)C1=CC=C(Cl)C(C)=C1Cl | 11 | I |
| COC1=CC=C(C=C1)C1=CN2N=C3C(=NC2=N1)[C@@H]1CC[C@@]3(C)C1(C)C | 11 | I |
| CC(C)C1=CC=C(C=C1)C1=NC2=NN=NN2[C@@H](C1)C1=CC=C(F)C=C1 | 11 | I |
| CC1=NN(CC(=O)\N=C2/SC3=CC(Cl)=CC=C3N2CC#C)C(C)=C1 | 11 | I |
| COC1=NC(OC2=CC(Cl)=C(Cl)C=C2Cl)=NC(Cl)=N1 | 11 | I |
| FC(F)(Cl)C1=NN=C2SC(=NN12)C1=CC=C2C=CC=C(Cl)C2=N1 | 11 | I |
| CC1=CC=C2N(CC#C)\C(SC2=C1)=N/C(=O)C1=CC=C2N=CC=NC2=C1 | 11 | I |
| CCOC1=CC=C2C(=O)\C(OC2=C1C)=C\C1=CC(Cl)=CC2=C1OCOC2 | 11 | I |
| ClC1=CC=C(C=C1)C1=C2N=CC=C(N2N=C1)C1=CC=C2OCOC2=C1 | 11 | I |
| CC(C)(C)N1N=CC(O\N=C2\C[C@@H]3CC[C@@]2(C)C3(C)C)=C(Cl)C1=O | 11 | I |
| CC1=CC=CN2C(=O)C=C(OC(=O)C34CC5CC(CC(C5)C3)C4)N=C12 | 11 | I |
| ClC1=C(N2C=CC=CC2=N1)C(=O)\C=C\C1=CC=CC2=C1OCCCO2 | 11 | I |
| CC(C)C1=CC=C(C)C(OC2=C3C(Br)=NN(C)C3=NC=N2)=C1 | 11 | I |
| [O-][N+](=O)C1=CC=CC(\C=C2/C=C(OC2=O)C2=CC=C(Br)C=C2)=C1 | 11 | I |
| CN(C(=O)C1=CC(Br)=CC=C1C)C1=CC=C(C=C1)[N+]([O-])=O | 11 | I |
| CC(C)OC1=CC=C(C=C1)[C@@H]1CC(=O)OC2=C1C1=C(C=C2)C(C)=CC(=O)O1 | 11 | I |
| ClC1=CC(\C=C/C(=O)C2=CC3=C(OCO3)C=C2)=CC2=C1OCCO2 | 11 | I |
| COC1=CC=C(C=C1)[C@@H]1CC(=NC2=NC(C)=NN12)C1=CC=C2C=CC=CC2=C1 | 11 | I |
| CN1N=C(C=C1C)C(=O)\N=C1/SC2=C(N1C)C(Cl)=C(Cl)C=C2 | 11 | I |
| BrC1=CC=C(CN2C=NC3=CC=CC=C23)C=C1 | 11 | I |
| BrC1=CC=C(\C=N\C2=C(CCC2)C#N)C=C1 | 11 | I |
| C[C@@H]1C[C@@]2(C)[C@@H](CC[C@@H]3[C@@H]4CCC(=O)[C@@]4(C)CC[C@@H]23)CC1=O | 11 | I |
| FC1=CC=C(C=C1F)[C@@H](Cl)[C@@H]1COC2=CC=CC=C2O1 | 11 | I |
| CC1=C(C)C2=C(S1)N=C(CC(F)(F)F)N=C2Cl | 11 | I |
| Cl[C@H]([C@H]1CCS(=O)(=O)C1)C1=CC2=CC=CC=C2C=C1 | 11 | I |
| CC1=CC=C(O1)C(=O)C1=CC(Br)=CC=C1F | 11 | I |
| FC1=CC=C2SC\C(=C\C=C\C3=CC=CO3)C(=O)C2=C1 | 11 | I |
| BrC1=C(CN2N=CC3=CC=CC=C23)SC=C1 | 11 | I |
| CC1(C)C[C@H]1N1C(CCl)=NC2=C1C(F)=C(F)C=C2 | 11 | I |
| CN1C=C(C(=O)C2=CC=CC(F)=C2F)C2=C1C=CC=C2 | 11 | I |
| C[C@@H](Cl)C1=C(C)N=C(N=C1)C1=CC(F)=C(F)C(F)=C1 | 11 | I |
| FC1=C(Br)C=C(C=C1)C1=CN=C(CCl)O1 | 11 | I |
| FC1=CC(=CC(F)=C1F)C(=O)C1=CC2=C(COC2)C=C1 | 11 | I |
| ClC1=C(Br)C=C2C(Cl)=C(C=NC2=C1)C#N | 11 | I |
| COC1=CC=CC=C1C1=C(Br)N=CS1 | 11 | I |
| CCC1=NC2=CC(Br)=C(F)C=C2N1C(C)C | 11 | I |
| CC1=C(Cl)C=NN1CC1=CC=C(Br)C=C1 | 11 | I |
| FC1=CC(Br)=C(C=C1)C1=CN=C(CCl)O1 | 11 | I |
| ClC1=NC(CC2=CC(Br)=CC=C2)=NS1 | 11 | I |
| CC1=NC(=C(C)C(Cl)=N1)C1=CC=C(Br)C=C1 | 11 | I |
| CN1N=C(C)C=C1C[C@@H]1CCC[C@H](Br)C1 | 11 | I |
| CN1N=C(C)C=C1C[C@H]1CCC[C@H](Br)C1 | 11 | I |
| CN1N=C(C)C=C1C[C@H]1CCC[C@@H](Br)C1 | 11 | I |
| CC(C)C1=C(Br)C(Cl)=NC(=N1)C1CC1 | 11 | I |
| CC1=CC(=C(C)S1)C1=NC=C(Br)C(Cl)=N1 | 11 | I |
| C[C@H](Br)[C@@H](C)C1=CC=C2OCCOC2=C1 | 11 | I |
| ClCC1=C(OC=N1)C1=CC(Br)=CC=C1 | 11 | I |
| CC1=CC=C(C=C1F)C1=C(CBr)N=CO1 | 11 | I |
| FC1=CC(=CC(F)=C1)C1=C(CBr)N=CO1 | 11 | I |
| BrC1=C(ON=C1)C1=CC=C(Br)C=C1 | 11 | I |
| ClC1=C(\C=C\CBr)N2C=CSC2=N1 | 11 | I |
| CCC1=C(Cl)N=C(N=C1Cl)[C@@H]1CSCCS1 | 11 | I |
| CC1=CC=C(C2=C(CBr)N=CO2)C(F)=C1 | 11 | I |
| CC(C)C1=C(Cl)N=C(N=C1Cl)[C@H]1CCCS1 | 11 | I |
| ClC1=CC(=CC2=C1OCO2)[C@H]1C[C@H]1CBr | 11 | I |
| CN1C=C(C[C@@H]2CCCCC[C@H]2Br)C=N1 | 11 | I |
| C[C@H]1CC[C@@H](C#N)[C@@H](C1)N1CCC[C@@H](C1)C(F)(F)F | 11 | I |
| C[C@H]1CC[C@H](C#N)[C@@H](C1)N1CCC(CC1)C(F)(F)F | 11 | I |
| CC1=C(C)N=C(SC2=CC=C(F)C=C2)C(Cl)=N1 | 11 | I |
| CC(C)C1=C(N=CN=C1Cl)C1=CC(F)=CC(F)=C1 | 11 | I |
| CC(C)C1=C(N=CN=C1Cl)C1=CC=C(F)C(F)=C1 | 11 | I |
| CN1N=C(C[C@@H]2CC[C@@H](Br)C2)C2=CC=CC=C12 | 11 | I |
| CCC1=C(Br)C=C2SC(SC)=NC2=N1 | 11 | I |
| CC1=CC=C(SC2=C(Br)C=NC=N2)C=C1 | 11 | I |
| FC1=CC=C(C(=O)C2=CC3=C(OCC3)C=C2)C(F)=C1F | 11 | I |
| CSC1=NC=CC(=N1)C1=CC=C(Br)C=C1 | 11 | I |
| O=C\C=C/C1=C2O[C@H]3C=CC=CC3=C[C@H]2C2=CC=CC=C12 | 11 | I |
| C[C@@H](Cl)C1=NC2=CC(Cl)=C(F)C=C2N1C1CC1 | 11 | I |
| C[C@@]12CC[C@H]3[C@@H](CC[C@H]4CCCC[C@]34C)C1=CC(=O)C2=O | 11 | I |
| CC1(C)OC2=CC(I)=C(C=C2O1)[N+]([O-])=O | 11 | I |
| CN1C=NN=C1SCC(=O)N1C2=C(CCCC2)C2=CC=CC=C12 | 11 | I |
| CSC1=CC=C(C=C1)C1OC(=O)C(=CC2=CC=C(C)O2)C(=O)O1 | 11 | I |
| CC\N=C1\S\C(=C\C2=CC=C3OCOC3=C2)C(=O)N1CC | 11 | I |
| O=C(C1=CC2=CC=CC=C2OC1=O)C1=CC2=CC=CC=C2OC1=O | 11 | I |
| CC1=C(C=CO1)C1=NN=C2SC(=NN12)C1=CC=CC=C1F | 11 | I |
| CC1=NC2=C(C#N)C(=NN2C(C)=C1)C(=C\C1=CC=C(Cl)C=C1)\C#N | 11 | I |
| FC1=CC=C(\C=C\C2=NN3C(S2)=NN=C3C2=CC=CO2)C=C1 | 11 | I |
| FC1=CC=C(C=C1)C1=N\C(=C\C2=CC=C3OCOC3=C2)C(=O)O1 | 11 | I |
| CN1\C(SC2=C1C=C(C)C(C)=C2)=N/C(=O)C1=NOC(C)=C1 | 11 | I |
| CC(C)CN1C(C)=C(C)C2=C1N=CN1N=C(N=C21)C(C)(C)C | 11 | I |
| CSC1=CC=C2N(CC#C)\C(SC2=C1)=N\C(=O)C1=CC=NO1 | 11 | I |
| COC1=CC=CC=C1\N=C1/SC=C(C)N1N1CCOCC1 | 11 | I |
| CN1\C(SC2=C1C(Cl)=CC=C2)=N\C(=O)C1=COCCO1 | 11 | I |
| CCN1\C(SC2=CC=CC(OC)=C12)=N\C(=O)C1=CC=CO1 | 11 | I |
| CN(C1CCCCCC1)S(=O)(=O)C1=CC=C2OC(=O)C=CC2=C1 | 11 | I |
| CC1=CC=C(C=C1)C1=N\C(=C/C2=CN(N=C2)C2=CC=CC=C2)C(=O)O1 | 11 | I |
| CN1\C(SC2=CC(C)=CC(C)=C12)=N/C(=O)C1=CC(C)=NO1 | 11 | I |
| CC(C)C1=C2CCCC2=C2C(OC3=C2N=CN(CC(C)=C)C3=O)=N1 | 11 | I |
| C[C@H](N(C)C1=C2C=NN(C)C2=NC=N1)C1=CC=C(Cl)C=C1Cl | 11 | I |
| CC1=C(C)C2=C(N=CN3C4=CC=CC=C4N=C23)N1CC1=CC=CO1 | 11 | I |
| CC1=CC2=NC(COC3=CC=C(Cl)C=C3Cl)=CC(=O)N2O1 | 11 | I |
| C#CCSC1=NC2=CC=CC=C2C2=NC(=NN12)C1=CC=CO1 | 11 | I |
| CN1N=CC=C1C(=O)\N=C1/SC2=CC(Cl)=CC=C2N1C | 11 | I |
| CN1N=CC=C1C(=O)\N=C1\SC2=CC(C)=CC(C)=C2N1C | 11 | I |
| CC(=O)OC1=CC=C2C(CC3=C2C=CC(=C3)\N=C2\OC(=O)C=C2)=C1 | 11 | I |
| [O-][N+](=O)C1=CC=C(OC2=C(Br)C=C(F)C=C2)N=C1 | 11 | I |
| FC(F)(F)C1=NN(C(=O)C2=CC3=C(OCO3)C=C2)C(=C1)C1CC1 | 11 | I |
| COC1=CC=CC=C1C1=NN2C(S1)=NN=C2C1CCCCC1 | 11 | I |
| ClC1=CC=CC=C1C1=NN=C2SC(=NN12)C1=CC=NC=C1 | 11 | I |
| C[C@H]1[C@H](OC2=C1C=C(C)C=C2Cl)C(=O)N(C)C[C@@H]1C=C(C)N=N1 | 11 | I |
| COC1=CC=C2[C@H]3CC[C@]4(C)[C@@H](CCC4=O)[C@H]3CCC2=C1[N+]([O-])=O | 11 | I |
| CC1=CC=C2N(N=NC2=C1)S(=O)(=O)C1=CC=C(C)C(C)=C1 | 11 | I |
| C[C@H]1S\C(=N\N=C\C2=CC=CO2)N(C1=O)C1=CC=C(Cl)C=C1 | 11 | I |
| O=C1OC(=N\C1=C\C1=CSC(=N1)C1=CC=CO1)C1=CC=CC=C1 | 11 | I |
| CC1=CC(OS(=O)(=O)C2=CC=CC3=NSN=C23)=CC(C)=C1 | 11 | I |
| O1C2=CC(=CC=C2N=C1C1=CC=CC2=CC=CC=C12)N1C=NN=C1 | 11 | I |
| O=C1OC2=C(C=C1)C=C1C(OC=C1C1=CC=C3OCCOC3=C1)=C2 | 11 | I |
| CC1=C2OC(=NC2=CC(=C1)N1C=NN=C1)C1=CC=C(C)C(C)=C1 | 11 | I |
| C[C@H]1CCCC[C@H]1N(C)S(=O)(=O)C1=CC=C2OC(=O)C=CC2=C1 | 11 | I |
| CC(C)(C)C1CCC(CC1)\N=C\C1C(=O)OC(C)(C)OC1=O | 11 | I |
| CCN1N=NC2=CC(=CC=C12)C(=O)N(C)C1=CC=C(C)C=C1C | 11 | I |
| CN(C)C1=NC(=NC(Cl)=N1)N1CCC[C@H](C1)C(F)(F)F | 11 | I |
| CCOC1=CC=CC=C1C1=NN=C2N1C(C)=C(C)N=C2Cl | 11 | I |
| CC(C)OC1=NC(=NC(Cl)=N1)C1=CC=CC2=C1C=CN=C2 | 11 | I |
| [O-][N+](=O)C1=C(Cl)N(N=C1C(F)(F)F)C1=C(F)C=C(F)C=C1 | 11 | I |
| CCC1=NN(C(Cl)=C1[N+]([O-])=O)C1=CC=C(Cl)C(F)=C1 | 11 | I |
| C[C@H]1C[C@@H]2CCCC[C@@H]2N1CC1=CN=C(N=C1)N1CCOCC1 | 11 | I |
| CC(C)(C)C1=N\C(=C/C2=C(Cl)N=C3SC=CN23)C(=O)O1 | 11 | I |
| COC1=CC=C(C=C1)C1=N\C(=C\C2=CN(C)C3=C2C=CC=C3)C(=O)O1 | 11 | I |
| ClC1=CC=C(C=C1)N1CSC2=C(C#N)[C@H](CC(=O)N2C1)C1=CC=CC=C1 | 11 | I |
| ClC1=CC=C(C=C1)N1CSC2=C(C#N)[C@@H](CC(=O)N2C1)C1=CC=CC=C1 | 11 | I |
| COC1=CC=C(\C=C2/N=C(OC2=O)C2=CC=CC=C2F)C=C1Br | 11 | I |
| COC1=CC=C(C=C1Br)C1=N\C(=C\C2=CC=CC=C2Cl)C(=O)O1 | 11 | I |
| COC1=CC=C(C=C1OC)C1=C2C=C(I)C=CC2=NO1 | 11 | I |
| CSC1=NC2=C(C3=C(CO[C@@H](C3)C(C)C)S2)C(=O)N1C1=CC=C(C)C=C1 | 11 | I |
| CSC1=NC2=C(C3=C(CO[C@H](C3)C(C)C)S2)C(=O)N1C1=CC=C(C)C=C1 | 11 | I |
| COC1=CC=CC=C1N1C(=O)[C@H]2[C@@H](C1=O)C1(C)C3=CC=CC=C3C2C2=C1C=CC=C2 | 11 | I |
| COC1=CC(=CC=C1)C1=NN(C2=C1C=NC1=CC=C(F)C=C21)C1=CC=C(C)C=C1 | 11 | I |
| [O-][N+](=O)C1=CC2=C(C=C1)C1=C(C2=O)C(Br)=CC(Br)=C1 | 11 | I |
| FC1=CC=C(C=C1)[C@H]1N(CCN2C=CC=C12)S(=O)(=O)C1=CC=C(Cl)C=C1 | 11 | I |
| ClC1=CC=C(C=C1)S(=O)(=O)N1CCN2C=CC=C2[C@@H]1C1=CC=CC=C1 | 11 | I |
| COC1=CC=C(\C=C2\N=C(OC2=O)C2=CC=C(C)C=C2)C=C1Br | 11 | I |
| CC(=O)N1N=C(O[C@@H]1C1=CC=CC=C1Cl)C1=CC=C(Br)C=C1 | 11 | I |
| CC1=NN2C(=N1)C1=CC=CC=C1N=C2SCC1=CC=C(Br)C=C1 | 11 | I |
| FC1=CC=C(C=C1)C1=NN2[C@H](C1)C1=CC(Cl)=CC=C1O[C@@H]2C1=CC=NC=C1 | 11 | I |
| CC1=CC=C(OC2=CC=C(C=C2)N2C(=O)[C@H]3[C@H]([C@H]4C=C[C@@H]3[C@H]3C[C@@H]43)C2=O)C=C1 | 11 | I |
| CC1=CC=C(C=C1)N1C(=O)CS[C@@]11C(=O)N2C3=C(C=C(C)C=C13)C(C)=CC2(C)C | 11 | I |
| COC1=C(Br)C=C(C)C(=C1)S(=O)(=O)N1C[C@H](C)C[C@@H](C)C1 | 11 | I |
| CC1=C(CN2C[C@H](OC3=CC=C(C)C=C3C2)C2=CSC=C2)N2C=CSC2=N1 | 11 | I |
| CC(C)C1=C(C=C(C)C(Br)=C1)S(=O)(=O)N1C=NC2=C1C=CC=C2 | 11 | I |
| FC1=CC=C(C=C1)[C@H]1CN(CCO1)S(=O)(=O)C1=CC(Cl)=CC(Cl)=C1 | 11 | I |
| FC1=CC=C2C(=C1)N(C=C(C(=O)C1=CC=CC=C1)S2(=O)=O)C1=CC=CC=C1 | 11 | I |
| CC1=CC=C(C=C1)N1C=C(C(=O)C2=CC=CC=C2)S(=O)(=O)C2=C1C=C(F)C=C2 | 11 | I |
| FC1=CC=C(C=C1)N1C=C(C(=O)C2=CC=CC=C2)S(=O)(=O)C2=CC=CC=C12 | 11 | I |
| CC(=O)O[C@]1(C(C)=O)C(=C)C[C@@H]2[C@@H]3C=C(C)C4=CC(=O)CC[C@]4(C)[C@H]3CC[C@]12C | 11 | I |
| C[C@H]1C[C@@H]2[C@H]3CC[C@](OC(C)=O)(C(C)=O)[C@]3(C)CC[C@@H]2[C@@]2(C)C=CC(=O)C=C12 | 11 | I |
| IC1=CC=CC(=C1)C(=O)[C@H](C#N)C1=CC=C2OCOC2=C1 | 11 | I |
| FC(F)(F)C1=CC=C(C=C1)[C@@H]1SC[C@@H]2N1C(=O)N(C2=O)C1=CC=CC=C1 | 11 | I |
| C[C@H]1C[C@@H]2[C@H]3CC[C@](OC(C)=O)(C(C)=O)[C@@]3(C)CC[C@@H]2[C@@]2(C)CCC(=O)C=C12 | 11 | I |
| C[C@@H]1C[C@H]2[C@H]3C[C@H](OC(C)=O)[C@H](C(C)=O)[C@]3(C)CC[C@H]2[C@@]2(C)CCC(=O)C=C12 | 11 | I |
| COC1=CC=C(C=C1)N1C(=O)S\C(=C(\C)C2=CC=C(Br)C=C2)C1=O | 11 | I |
| CC1=CC=C(C=C1)N1[C@@H](C2=C(OC3=CC=C(F)C=C3C2=O)C1=O)C1=CC=C(F)C=C1 | 11 | I |
| CCC1=CC=C(C=C1)[C@@H]1N(C(=O)C2=C1C(=O)C1=CC(F)=CC=C1O2)C1=CC=CC=C1 | 11 | I |
| CN(C)\C=C\C1=C2C(=O)C(=O)N3C2=C(C=C1)[C@](C)(CC3(C)C)C1=CC=CC=C1 | 11 | I |
| CC1=CC=C([C@@H]2CC(=NC3=NC=NN23)C2=CC=C(Br)C=C2)C(C)=C1 | 11 | I |
| CC1=CC=CC2=NC(COC3=CC=C(Br)C=C3Cl)=CC(=O)N12 | 11 | I |
| CC1=NN(CC2=CC=C(F)C=C2)C(Cl)=C1C=C1C(=O)C2=CC=CC=C2C1=O | 11 | I |
| ClC1=C(CON=C2CCC3(CC2)OCCO3)C=CC(Br)=C1 | 11 | I |
| FC1=CC=C(C=C1)C1=NC2=NC(=NN2[C@H](C1)C1=CC=C(F)C(F)=C1)C(F)(F)F | 11 | I |
| CN(C)\N=C1/C(=O)N(CC2=CC=C(F)C=C2)C2=CC=C(Br)C=C12 | 11 | I |
| FC1=C(C=CC(Br)=C1)C1=NN=C2SC(=NN12)C1=CSC=C1 | 11 | I |
| CC(C)C(=O)N1CCC2=CC(=CC=C12)C1=CC=C(C=C1)C(=O)N1CCC(C)CC1 | 11 | I |
| ClC1=CC=CC=C1[C@@H]1CC(=NC2=NC=NN12)C1=CC=C(Br)C=C1 | 11 | I |
| C[C@@H]1C[C@H]2[C@@H](CN3CC[C@@H](C)CC(C)(C)C3)C(=O)O[C@@H]2[C@@H]2[C@@H]3O[C@]3(C)CC[C@@H]12 | 11 | I |
| FC1=CC=CC(CN2C[C@H]([C@@H]3[C@H]2C2CCN3CC2)C2=CC3=C(OCO3)C=C2)=C1F | 11 | I |
| COC1=CC=CC2=C1N(C=C2I)C(=O)OC(C)(C)C | 11 | I |
| CC(=O)N1N=C(O[C@@]1(C)\C=C/C1=CC=CC=C1)C1=CC=CC(Br)=C1 | 11 | I |
| CN1C(=O)\C(S\C1=C(\C#N)C(=O)C1=C(C)C=CS1)=C\C1=CC=C(C)C=C1 | 11 | I |
| C[C@@H](Cl)C1=NC2=C(N(C)N=C2C)N1C1=CC=C(Br)C=C1Cl | 11 | I |
| CC1=CC=CC(=C1)C#CC1(O)CC2CCC(C1)N2C1=NC=CC=C1C#N | 12 | A |
| COC(=O)N1C2CCC1CC(O)(C2)C#CC1=CC=CC(C)=N1 | 12 | A |
| COC(=O)N1CCC2(CC(O)(C2)C#CC2=CC=CC(C)=C2)CC1 | 12 | A |
| COC(=O)N1C2CCC1CC(C2)C#CC1=CC=CC(C)=C1 | 12 | A |
| CC(=O)N1C2CCC1CC(O)(C2)C#CC1=CC=CC(C)=C1 | 12 | A |
| COC(=O)N1C2CCC1CC(O)(C2)C#CC1=CC=CC=C1 | 12 | A |
| CC1=CC=CC(=C1)C#CC1(O)CC2CCC(C1)N2S(C)(=O)=O | 12 | A |
| CC1=CC=CC(=C1)C#CC1(O)CC2CCC(C1)N2C(=O)C(C)(C)C | 12 | A |
| COC(=O)N1C2CCC1CC(O)(C2)C#CC1=CC=CC(C)=C1 | 12 | A |
| COC(=O)N1C2CCC1CC(O)(C2)C#CC1=CC=CC(C)=C1 | 12 | A |
| CN(C)C(=O)N1C2CCC1CC(O)(C2)C#CC1=CC=CC(C)=C1 | 12 | A |
| COC(=O)N1CC2CCC(O)(CC2C1)C#CC1=CC=CC(C)=C1 | 12 | A |
| COC(=O)N1C2CCC1CC(O)(C2)C#CC1=CC=CC(F)=C1 | 12 | A |
| COC(=O)N1C2CCC1CC(O)(C2)C#CC1=CC=CC(Cl)=C1 | 12 | A |
| CCOC(=O)N1C2CCC1CC(O)(C2)C#CC1=CC=CC(C)=C1 | 12 | A |
| COC(=O)N1C2CCC1CC(O)(C2)C#CC1=CC=CC(=C1)C#N | 12 | A |
| COC(=O)N1CCC(O)(CC11CCC1)C#CC1=CC=CC(C)=C1 | 12 | A |
| COC(=O)N1CCC2CC(O)(C#CC3=CC=CC(C)=C3)C2C1 | 12 | A |
| COC(=O)N1CCC(O)(C#CC2=CC=CC(C)=C2)C2CCCCC12 | 12 | A |
| COC(=O)N1CCC(O)(C#CC2=CC=CC(C)=C2)C2CCCC12 | 12 | A |
| COC(=O)N1CCC(O)(C#CC2=CC=CC(C)=C2)C2CCCC12 | 12 | A |
| COC(=O)N1C2CCC1CC(C2)(OC)C#CC1=CC=CC(C)=C1 | 12 | A |
| COC(=O)N1C2CCC1CC(O)(C2)C#CC1=CC=CC(OC)=C1 | 12 | A |
| COC(=O)N1CCCCC11CCCC(O)(C1)C#CC1=CC=CC(C)=C1 | 12 | A |
| COC(=O)N1CC2CCC1CC2(O)C#CC1=CC=CC(C)=C1 | 12 | A |
| CN(C)C(=N/C#N)\N1C2CCC1CC(O)(C2)C#CC1=CC=CC(C)=C1 | 12 | A |
| CC1=CC=CC(=C1)C#CC1(O)CC2CCC(C1)N2C(=O)N1CCC1 | 12 | A |
| COC(=O)N1C2CCC1CC(O)(C2)C#CC1=CC=CC(OC(F)F)=C1 | 12 | A |
| COC(=O)N1CC2CCC(O)(C#CC3=CC=CC(C)=C3)C2C1 | 12 | A |
| COC(=O)N1CCC2(CC(O)(CO2)C#CC2=CC=CC(C)=C2)CC1 | 12 | A |
| COC(=O)N1CCC2C1CCCC2(O)C#CC1=CC=CC(C)=C1 | 12 | A |
| COC(=O)N1CC(F)(F)C(O)(C#CC2=CC=CC(C)=C2)C2CCCC12 | 12 | A |
| CC(=O)NC1=CC=C2OCN(CC2=C1)C1=CC=C(F)C=C1 | 12 | I |
| FC(Cl)(Cl)C1=NC2=NC(Cl)=C(Cl)NC2=N1 | 12 | I |
| CC1=CC(=O)OC2=C1C=CC(NC(=O)C1=CSC=C1)=C2 | 12 | I |
| CC(=O)NC1=CC=C(\C=C2/COC3=CC=CC=C3C2=O)C=C1 | 12 | I |
| C\C(NC1=CC=CC2=C1C=CC=C2)=C1\C(=O)OC(C)=CC1=O | 12 | I |
| CSC1=NC2=C(SC3=C2COC(C)(C)C3)C(=O)N1 | 12 | I |
| CN1C=CC=C1C1=NN\C(SC1)=N/C1=CC=CC=C1C | 12 | I |
| S=C1NC=NN1C1C2=CC=CC=C2OC2=CC=CC=C12 | 12 | I |
| C[C@@H]1C[C@@H](C(=O)N2C[C@H](C)OC[C@@H]2C)C2=CC=CC=C2N1 | 12 | I |
| CC1=NSC(NC(=O)C2=CC=C(Br)O2)=C1 | 12 | I |
| C[C@@H]1NCCN2C1=NN=C2C1=CC=C(Br)C=C1F | 12 | I |
| N=C1S[C@H]2CCCCC2=C1C1=NN=CN1C1CCCC1 | 12 | I |
| C[C@@H]1CSCCN1C1=[NH+]C(=CC2=CC=CC=C12)C([O-])=O | 12 | I |
| CC(C)[C@H]1C[C@](O)(CCO1)C1=CC=C2OCCCOC2=C1 | 12 | I |
| FC1=C(NC2=CN=C(C=N2)C#N)C=C(Br)C=C1 | 12 | I |
| C1CC1[C@H]1CN\C(S1)=N/C1=CC2=C(OCCCO2)C=C1 | 12 | I |
| CC[C@]1(C)CS\C(N1)=N/C1=CC=C2OCCCOC2=C1 | 12 | I |
| O=S(=O)(\N=C1/NC2=CC=CC=C2C=C1)C1=CC=CC=C1 | 12 | I |
| CC(C)CN1C(=O)C2(CCCCC2)NC(=O)C11CCCC1 | 12 | I |
| CN1C[C@@H](O)C2=CC3=C(OCO3)C3=C2[C@@H]1CC1=C3C=CC=C1 | 12 | I |
| CC[C@H]1N(C(=O)C2(CCCC2)NC1=O)C1=CC=CC=C1F | 12 | I |
| C[C@@H]1N(C(=O)[C@H](NC1=O)C(C)(C)C)C1=CC=C(C)C(C)=C1 | 12 | I |
| C[C@@H]1N(C2=CC=C(C)C=C2)C(=O)C2(CCCCC2)NC1=O | 12 | I |
| CC[C@H]1NC(=O)[C@H](C)N(C1=O)C1=CC(Cl)=CC=C1C | 12 | I |
| CC[C@@H]1NC(=O)[C@@H](C)N(C1=O)C1=CC=C(C)C(Cl)=C1 | 12 | I |
| C[C@@H]1N(C2=CC=C(C)C(Cl)=C2)C(=O)C(C)(C)NC1=O | 12 | I |
| C[C@@H]1NC(=O)CN(C1=O)C1=CC=C(Br)C=C1Cl | 12 | I |
| CC1(CCCCC1)N1CCC(=O)NC2(CCCCC2)C1=O | 12 | I |
| CCC1=C(Br)C(Cl)=NC(=N1)C1=NC=CN1 | 12 | I |
| C[C@H]1CC[C@H](C1)C1=NOC(=N1)[C@H]1NCCC2=C1C=CS2 | 12 | I |
| C[C@H]1CC[C@H](C1)C1=NOC(=N1)C1=C2CCC[C@@H]2SC1=N | 12 | I |
| C[C@@H]1CN(C)C2=CC=CC=C2N(C1)C(=O)CC1=CC=CN1 | 12 | I |
| CCC1=C(OC2=CC=CC=C12)[C@@H]1CS(=O)(=O)C[C@@H](C)N1 | 12 | I |
| CN1C=C(NC(=O)C2=C(C)C=C(C)C=C2C)C(C)=CC1=O | 12 | I |
| FC1=CC(Br)=CC(=C1)C1=NNC(CC#N)=N1 | 12 | I |
| O=C(N1CC[C@]2(C1)CCCNC2=O)C1=CC2=C(CCCC2)S1 | 12 | I |
| CC1=C(CN2C(=S)NC3=CC=CC(Cl)=C23)C(C)=NO1 | 12 | I |
| CN1N=C(C)C2=C1N(C(=S)N2)C1=CC=C(C)C(Cl)=C1 | 12 | I |
| CCC1=NN(C)C2=C1NC(=S)N2[C@H]1CCC[C@H](C)[C@@H]1C | 12 | I |
| CCC1=NN(C)C2=C1NC(=S)N2C1=CC=C(F)C(F)=C1 | 12 | I |
| O=C1CN(CC2=COC3=CC=CC=C23)[C@H]2CCCC[C@@H]2N1 | 12 | I |
| CCC1=C(C)N=C(NC1=O)C1=C(Cl)C=C(Cl)C=N1 | 12 | I |
| C[C@@H]1CN(C(=O)NC1=O)C1=CC=C(Br)C=C1F | 12 | I |
| CC(C)(C)[C@@H]1NC(=O)CCN([C@@H]2CCCC2(C)C)C1=O | 12 | I |
| C[C@@H]1N([C@@H]2CCCCC2(C)C)C(=O)[C@](C)(NC1=O)C1CC1 | 12 | I |
| [O-]C(=O)[C@@H]1CCCCC[C@@H]1[NH+]1CCN2CCCC[C@@H]2C1 | 12 | I |
| CC1=CC(C)=C2C(=O)C(=O)N(CC3=CC=C(O)C=C3)C2=C1 | 12 | I |
| C[C@@H]1SC(=N)C(=C1C)C1=NC(=NO1)C1=CSC=C1C | 12 | I |
| OC1=CC2=C(OC=C2C2=CC3=C(OCCCO3)C=C2)C=C1 | 12 | I |
| CC(C)N1N=CC2=C1N=C(O)C[C@H]2C1=CC=CC=C1Cl | 12 | I |
| CC1=C(Br)C(=O)OC2=C3NC(=O)COC3=CC=C12 | 12 | I |
| CCCN1CC(=O)N2[C@@H](C)C3=C(C[C@H]2C1=O)C1=CC=CC=C1N3 | 12 | I |
| CN1C(=O)C=C(NC2=CC=C(Br)C=C2)N(C)C1=O | 12 | I |
| CSC1=CC=C(C=C1)[C@@H]1CC(=O)NC2=C1C(=O)N=C1SC=CN21 | 12 | I |
| C[C@@H]1CCC2=C(C1)SC(NC(=O)C1=C(C)ON=C1C)=C2C#N | 12 | I |
| ClC1=C(Cl)C(\C=N\N2C(=S)NN=CC2=O)=C(Cl)C=C1 | 12 | I |
| CN1C=C(Br)C(=N1)C(=O)NC1=NC2=CC=CC=C2S1 | 12 | I |
| OC1=C(\C=N/N2C=NN=C2)C=C(Br)C=C1Br | 12 | I |
| CC[C@H](C)N1CC(=O)N2[C@H](C)C3=C(C[C@@H]2C1=O)C1=CC=CC=C1N3 | 12 | I |
| O=C1CC2=CC(=CC=C2N1)S(=O)(=O)N1CCC2=CC=CC=C12 | 12 | I |
| C[C@@H]1CCC2=C(C1)SC(NC(=O)C1=C(C)N(C)N=C1C)=C2C#N | 12 | I |
| C[C@H]1CCC2=C(C1)SC(NC(=O)C1=C(C)N(C)N=C1C)=C2C#N | 12 | I |
| BrC1=CC=CC(=C1)N1NC(=O)\C(=C\C2=CC=CO2)C1=O | 12 | I |
| CNC(=O)[C@@H]1CC2=CC=CC=C2N1C(=O)C1=CC2=CC=CC=C2O1 | 12 | I |
| CN1N=CC2=C1CCC1=C2N=C(NC(=O)[C@H]2CCC=CC2)S1 | 12 | I |
| C[C@@H](N1CCOCC1)C1=NC2=C(C3=C(C[C@@H](C)CC3)S2)C(=O)N1 | 12 | I |
| [O-][N+](=O)[C@@H]1CCC(=O)N[C@@H]1C1=CC(Br)=C(F)C=C1 | 12 | I |
| CC1=C(SC(=O)N1)S(=O)(=O)N1CCC[C@H]2CCCC[C@H]12 | 12 | I |
| ClC1=CC=C(C=C1)C(=O)NC1=CC2=C(CCCC2=O)OC1=O | 12 | I |
| CC1=C(OC2=C1C=C(C)C(C)=C2)C(=O)N[C@@H]1CCS(=O)(=O)C1 | 12 | I |
| CC1=C(C)C(=O)NC(=N1)C1=CC2=C(OCCO2)C(Br)=C1 | 12 | I |
| CN1N=C(C)C(C(=O)N[C@@H]2CC(C)(C)CC3=C2C=C(C)O3)=C1Cl | 12 | I |
| CN1C=CC(NC(=O)C2=CC(Br)=CN=C2Cl)=N1 | 12 | I |
| CC1=NN(CC2=NC3=C(SC=C3)C(=O)N2)C(C)=C1Br | 12 | I |
| CN1C(=O)N(C)C2=CC([C@H](O)C3=CC=CO3)=C(Br)C=C12 | 12 | I |
| BrC1=CC2=C(NC(=O)\C2=N\N2CCOCC2)C=C1 | 12 | I |
| CN1CCCC2=CC(NC(=O)[C@@H]3CS[C@@]4(C)CCC(=O)N34)=CC=C12 | 12 | I |
| O=C1N[C@@]2(CCCCC3=CC=CC=C23)C(=O)N1CC1=CC=CN=C1 | 12 | I |
| O=C(N1CCC2(CCCCC2)CC1)C1=CC2=C(CCCC2=O)NC1=O | 12 | I |
| O=C(N1CCCS1(=O)=O)C1=CC2=C(NC3=C2CCCC3)C=C1 | 12 | I |
| C[C@H]1CN([C@@H](C)CO1)C(=O)C1=NNC2=CC=C(Br)C=C12 | 12 | I |
| C[C@H]1CN(C[C@@H](C)O1)S(=O)(=O)C1=CC=C2CCCNC2=C1 | 12 | I |
| CC1=C(Br)C=NC2=C1NC(=N2)[C@H]1COC(C)(C)O1 | 12 | I |
| CC1=C(Br)C(C(=O)NC2=CN3C=CN=C3C=C2)=C(C)O1 | 12 | I |
| O[C@H]1CCCN(C1)C(=O)\C=C\C1=CC2=C(OCO2)C(Cl)=C1 | 12 | I |
| C[C@@H](NC(=O)N1CCS[C@@H](C)[C@@H]1C)C1=C(C)N(C)N=C1C | 12 | I |
| BrC1=CC(=CC2=C1OCO2)[C@@H]1NC(=O)[C@@H]2CCCCN12 | 12 | I |
| FC1=CC=C(CN2C(=O)NC3=CC=CC=C3S2(=O)=O)C=C1F | 12 | I |
| FC1=CN\C(C=C1)=N/S(=O)(=O)C1=C(C#N)C(Cl)=CC=C1 | 12 | I |
| C[C@@H]1CC[C@H](C)N(C1)C(=O)NC[C@@H]1CN2CCCC[C@@H]2CO1 | 12 | I |
| C[C@H]1CN(C[C@@H](C)S1)C(=O)NC[C@@H]1CN2CCCC[C@@H]2CO1 | 12 | I |
| C[C@@H]1CCN([C@@H](C)C1)C(=O)NC[C@@H]1CN2CCCC[C@@H]2CO1 | 12 | I |
| CC1=CN=C2SC[C@@H](CN2C1=O)C(=O)NC1=CC=C(F)C(F)=C1 | 12 | I |
| C[C@@H]1CC[C@H](C)N(C1)C(=O)NC1=CC(Br)=CN(C)C1=O | 12 | I |
| C[C@H]1CN(C[C@@H](O1)C1=CSC=C1)C(=O)NC1=CC=C2COCC2=C1 | 12 | I |
| CC(C)(C)C1=C(Br)C([O-])=NC(=N1)[C@@H]1C[NH+]2CCN1CC2 | 12 | I |
| CN1N=CC(N[C@H]2[C@@H]3CCO[C@@H]3C2(C)C)=C(Br)C1=O | 12 | I |
| O=C1SC2=CC=CC=C2N1CC1=NC(=O)C2=C(N1)C=CS2 | 12 | I |
| CC(=O)NC1=CC=C(\C=C2\CS(=O)(=O)C3=CC=CC=C3C2=O)C=C1 | 12 | I |
| C[C@@H]1CN([C@@H]2CCCC[C@H]12)C(=O)NC[C@@H]1CCCS1(=O)=O | 12 | I |
| O=C1NC(=O)[C@H]2[C@H]1ON([C@H]2C1=CC=CS1)C1=CC=CC=C1 | 12 | I |
| CNC(=O)OC1=CC2=C(OC(=O)S2)C=C1Br | 12 | I |
| CC1=CC=C(O1)\C=C1/SC(=O)N(C1=O)C1=CC=CC(O)=C1 | 12 | I |
| CC1=NNC2=C1[C@@H](N(C1=NC=CS1)C2=O)C1=CC=CC=C1 | 12 | I |
| CC1(C)NS(=O)(=O)N(CC2=CC=CC3=C2C=CC=C3)C1=O | 12 | I |
| CNC(=O)C1=CN=C2SC=C(N2C1=O)C1=CC=C(C)C=C1 | 12 | I |
| CC1=C(C)N=C(NC(=O)C2=C(C)C3=C(CCCC3=O)O2)S1 | 12 | I |
| BrC1=CC=C(C=C1)C1=CN=C2C(=O)NC=NC2=N1 | 12 | I |
| CC1=NC=C(S1)C1=NC2=C(N1)C=NN2CC1=CC=CC=C1 | 12 | I |
| CC1=CC(C)=C(NC(=O)C2=C3N=C(C)C=C(C)N3N=C2)C(C)=C1 | 12 | I |
| CC1=NC2=CC(=CC=C2O1)\N=C\C1=CC2=CC=CC=C2NC1=O | 12 | I |
| CC1=NN2C(=C1)N=CC(C(=O)NC1=CC=C(C)C=C1F)=C2C | 12 | I |
| CN1C=C(Br)C=C1C(=O)NC1=NC(C)=CC(C)=N1 | 12 | I |
| O=C1N(C[C@H]2C=CN=N2)C(=S)NC2=C1C1=C(CCCC1)S2 | 12 | I |
| CN1N=C(C)C=C1NC(=O)[C@H]1COC2=CC=C(Cl)C=C2C1 | 12 | I |
| CC1=NOC(CN2C(=S)NC3=C(C(C)=C(C)S3)C2=O)=C1 | 12 | I |
| CN1C=C(C=N1)C1=NC2=CC=C(Br)C=C2C(=O)N1 | 12 | I |
| O=C1N[C@@]2(CCCC3=CC=CC=C23)C(=O)N1C[C@H]1CCCO1 | 12 | I |
| CN1C(=O)N(C)C2=CC(NC(=O)C3=CC=C(F)C=C3F)=CC=C12 | 12 | I |
| CSC1=NC(=O)[C@H]2[C@@H](CC(=O)NC2=N1)C1=CC=C(C)C=C1C | 12 | I |
| CSC1=NC(=O)[C@H]2[C@H](CC(=O)NC2=N1)C1=CC=C(C)C=C1C | 12 | I |
| CC1=C2C(S[C@H]1C(=O)NC1=CC=C(C)C=C1)=NC=NC2=O | 12 | I |
| CC1=C2[C@H](NC(=O)C[C@@H]2C(=O)C2=CC=C(Br)C=C2)N=N1 | 12 | I |
| O=C1C[C@H]([C@@H]2C(N1)=NN=C2C1CCC1)C1=CC=C[C@H]2N=CC=C12 | 12 | I |
| O=C(NC1(CCCCCCC1)C#N)[C@@H]1CCS(=O)(=O)C1 | 12 | I |
| CN1C=NC2=CC(NC(=O)C3=CN(C)C4=C3C=CC=C4)=CC=C12 | 12 | I |
| FC(F)(F)[C@@H]1CCC[C@@H](C1)NC(=O)N1CCS(=O)(=O)CC1 | 12 | I |
| CN1C(=O)OC2=CC(NC(=O)C3=CC=CC(F)=C3F)=CC=C12 | 12 | I |
| CC1=CC=C(C=C1)N1C(=O)[C@H]2ONC(=C2C1=O)C1=CC=CC=C1 | 12 | I |
| OC(=O)[C@@H]1CCO[C@@H]1C1=CC2=C(OCCCO2)C(Cl)=C1 | 12 | I |
| OC(=O)[C@@H]1CCCO[C@@H]1C1=CC2=C(OCCO2)C(Cl)=C1 | 12 | I |
| ClC1=CC(=CNC1=O)S(=O)(=O)N1CCC[C@@H]2CCCC[C@H]12 | 12 | I |
| FC1=CC=CC2=C1C(=O)NC(CN1C=C(Br)C=N1)=N2 | 12 | I |
| CC1=NN=C2N1C=CN=C2N[C@H]1[C@@H](Cl)CC2=CC=CC=C12 | 12 | I |
| CC1=NC2=NC=NN2C(N[C@H]2[C@H](Cl)CC3=CC=CC=C23)=C1 | 12 | I |
| N=C1S[C@H]2CCCCC2=C1C1=NC(=NO1)C1CCOCC1 | 12 | I |
| CCN1N=C(C)C=C1C1=NC2=C(N1)N=CC(Br)=C2 | 12 | I |
| ClC1=C(Cl)C=C(C=C1)N1C(=O)N[C@H]2COC[C@H]2C1=O | 12 | I |
| CC(C)C1=C(Br)C(=O)N=C(N1)C1=C(C)N(C)N=C1C | 12 | I |
| OC1=C(N=C(Br)C=C1Br)[N+]([O-])=O | 12 | I |
| CC[C@@H]1N(CCNC1=O)C1=C(Br)C=NC(Cl)=N1 | 12 | I |
| CCCN1[C@H]2COC[C@H]2C(=O)NC2=C1N=CC(Br)=C2 | 12 | I |
| CC1=CN2C(S1)=NC(C)=C2CN1CC(=O)N[C@H]2CCCC[C@H]12 | 12 | I |
| C1CNC2=CC=CC(C3=NC(=NO3)[C@H]3CSCCO3)=C2C1 | 12 | I |
| C\C(=N/O)C1=CC=C(C=C1)N1C(=O)[C@H]2[C@H]3C[C@H](C=C3)[C@H]2C1=O | 12 | I |
| ClC1=CC(C2=NC(=NO2)[C@@H]2COCCN2)=C(Cl)C=C1 | 12 | I |
| CN1C=C(NC(=O)C2=CC=C(Br)O2)C=C(Cl)C1=O | 12 | I |
| CC1=CC(=CC(C)=C1F)C(=O)N1CC(=O)NC2=C1C=CN=C2 | 12 | I |
| CN1N=CC2=C1N=CN=C2N[C@@H]1CCSC2=C1C=CC=C2 | 12 | I |
| CN1CC2=CC=C(NC(=O)C3=CC4=C(S3)N(C)N=C4C)C=C2C1 | 12 | I |
| C[C@]12CC[C@H]3[C@@H](CC[C@H]4CC(C[N+]([O-])=O)=CC[C@]34C)[C@@H]1CC[C@@H]2O | 12 | I |
| CN1N=C(C)C(Br)=C1C(=O)NC12CC3CC(CC(C3)C1)C2 | 12 | I |
| OC1=CC2=C(C=C1)C(=O)C(OC1=CC(Cl)=CC=C1Cl)=CO2 | 12 | I |
| FC1=CC=CC=C1NC(=O)N1CCN2C=CC=C2[C@H]1C1=CC=CC=C1F | 12 | I |
| CC1=NC2=C(S1)C=CC1=C2SC(NC(=O)C2=CC=C(Cl)C=C2)=N1 | 12 | I |
| CC1=C(C)C2=C(S1)N=C(NC2=O)C(=C\C1=C(C)C=C(C)C=C1C)\C#N | 12 | I |
| ClC1=CC=C(C=C1)[C@H]1[C@@H]2CCCC=C2[C@H](C#N)C(=N)C1(C#N)C#N | 12 | I |
| N=C1[C@@H](C#N)C2=CCCC[C@H]2[C@@H](C2=CC=CC3=C2C=CC=C3)C1(C#N)C#N | 12 | I |
| OC1=CC2=C(C=C1)C(=O)\C(O2)=C\C1=CC2=CC(Br)=CC=C2O1 | 12 | I |
| OC1=CC(=CC=C1)C1=NN2[C@H](C1)C1=CC=CC=C1O[C@@H]2C1=CC=CS1 | 12 | I |
| CC1=NN2C(NC(=CC2=O)C(F)(F)F)=C1C1=CC=C(Cl)C=C1 | 12 | I |
| [S-]C1=[NH+][C@@H](C2CCC3=CC=CC=C3C2=N1)C1=CC=C2OCOC2=C1 | 12 | I |
| CC1=CC=CC=C1NC(=O)C1=CC2=C(Cl)C=CC3=C2N1CCO3 | 12 | I |
| ClC1=CC=C(NC(=O)C2=CC3=C(S2)N=C2SC=CN32)C=C1 | 12 | I |
| CN1C(C)=C(C)C2=C1C=CC(=C2)C(=O)NC1=NC2=CC=CC=C2S1 | 12 | I |
| S=C1NN=C(N1C1=CC=CC2=CC=CC=C12)C1=CC=C2C=CC=CC2=N1 | 12 | I |
| CC1=CC(Br)=CC=C1C1=NC(=NO1)C1=CC=CC(O)=C1 | 12 | I |
| CC1=CC(Br)=CC=C1C1=NC2=CC3=C(OCCO3)C=C2N1 | 12 | I |
| CC1=CC2=C(C=C1C)N(C=[NH+]2)C1=CC(Br)=CC=C1C([O-])=O | 12 | I |
| O=C(NCC1=CC=CO1)N1C2=CC=CC=C2SC2=CC=CC=C12 | 12 | I |
| CC1=CC=C2N=C(NC(=O)C3=CC=C4N=CSC4=C3)SC2=C1 | 12 | I |
| CC1=CC=CC2=C1C=NN2C(=O)NC1=CC=C(Br)C=C1F | 12 | I |
| ClC1=CC=C(C=C1)[C@@H]1N2N=CN=C2NC2=C1CCC1=CC=CC=C21 | 12 | I |
| CC1=CC=CC2=C1OC(=CC2=O)C(=O)NC1=CC=C(Br)C=C1 | 12 | I |
| CC1=CC2=C(C=C1Cl)C(=O)C1=C(O2)C(=O)N[C@H]1C1=CC=CC=C1F | 12 | I |
| CCC1=CC=C(C=C1)[C@@H]1NC(=O)C2=C1C(=O)C1=C(C)C=C(C)C=C1O2 | 12 | I |
| CN(C(=O)C1=C(C)N=C2C=CC(Cl)=CC2=C1)C1=CC=C(O)C=C1 | 12 | I |
| BrC1=CC=CC=C1C1=NOC(=N1)[C@H]1CC2=CC=CC=C2N1 | 12 | I |
| COC1=CC(=CC(Br)=C1O)C1=NC2=C(CCC2)C(Cl)=N1 | 12 | I |
| ClC1=CC=C(C=C1)[C@H]1C[C@H](N2N=CN=C2N1)C1=CC=C(Cl)C=C1 | 12 | I |
| FC1=CC2=C(CCC(=O)N2)C=C1C(=O)C1=CC2=C(O1)C=CC(Cl)=C2 | 12 | I |
| O[C@@H]1CCCCC[C@H]1C1=NC(=NO1)C1=CC(Br)=CS1 | 12 | I |
| OC1=CC2=C(C=C1)C(=O)\C(CC2)=C\C1=CC2=C(OCCO2)C(Cl)=C1 | 12 | I |
| CC1=NC(Cl)=C(NC(=O)C2=C(Cl)C=CC(Cl)=N2)C(C)=C1 | 12 | I |
| C\C(=C\C1=CC(Br)=CC(Br)=C1O)[N+]([O-])=O | 12 | I |
| C[C@H]1NC(=S)N(C1=O)C1=CC=C(C=C1)C1=CC2=CC=CC=C2O1 | 12 | I |
| CN1\C(NC2=CC(Cl)=CC=C12)=N\C(=O)CC1=CC=C(F)C(F)=C1 | 12 | I |
| C[C@@H]1CN[C@@H](CN1C1=C(Cl)C=CC2=NSN=C12)C(C)(C)C | 12 | I |
| CC1=CC(Br)=CC(C)=C1N1CC(=O)N[C@H](C1=O)C(C)(C)C | 12 | I |
| COC1=CC=C2NC(=NC2=N1)C1=CC=C(I)C=C1 | 12 | I |
| COC1=CC=C2NC(=NC2=N1)C1=CC=C(Br)C=C1F | 12 | I |
| CC1=CC(C(=O)NC2=CC=C(Cl)N=N2)=C(C)C=C1Br | 12 | I |
| CC1=C(Br)SC(=C1)C(=O)NC1=CC=CC2=NSN=C12 | 12 | I |
| C[C@H]1CC[C@@H](NC2=NN3C=CC=C(Br)C3=N2)[C@H](C)C1 | 12 | I |
| OC1=CC=CC2=CC=C(\C=C/C3=C(Cl)N=C4SC=CN34)N=C12 | 12 | I |
| CC1=NN(C2=C1[C@H](CC(O)=N2)C1=CSC=C1)C1=CC(C)=CC(C)=C1 | 12 | I |
| COC1=CC=C(\C=C2\COC3=CC=C(Br)C=C3C2=O)C=C1O | 12 | I |
| CC1=CC=C(C=C1F)C1=NC(=O)C2=C(N1)SC=C2C1=CC=CO1 | 12 | I |
| [O-]C(=O)C1=C([NH+]=C2C=CC(Br)=CN12)C1=CC=C(F)C=C1 | 12 | I |
| CC1=CC=CN\C1=N/S(=O)(=O)C1=CC(C)=C(C)C=C1Br | 12 | I |
| COC1=CC(=CC(=O)C2=C(C)OC(C)=C12)C1=CC=C(O)C=C1 | 12 | I |
| OC1=CC=C(C=C1)N1C(=O)C2=CC=C(Br)C=C2C1=O | 12 | I |
| CCC1=CC=C(C=C1)[C@@H]1CC(=O)NC2=C1C=C1OCCOC1=C2 | 12 | I |
| BrC1=CC=C(NC(=O)C2=NOC3=C2CCCC3)C=C1 | 12 | I |
| C1COC2=CC(=CC=C2O1)[C@H]1NC2=CC=CC=C2C2=CC=CN12 | 12 | I |
| FC1=CC(Cl)=CC=C1NC(=O)C1=CC2=CC=CC=C2OC1=O | 12 | I |
| BrC1=CC=CC(\C=C2/N=C(NC2=O)C2=CC=CO2)=C1 | 12 | I |
| CC1=C(SN=N1)C(=O)NC1=CC=C(Br)C(C)=C1 | 12 | I |
| CC1=CC=C(NC(=O)C2=CC3=CC=CC=C3OC2=O)C=C1F | 12 | I |
| FC1=CC=CC=C1[C@@H]1[C@@H]2CCCC=C2[C@@H](C#N)C(=N)C1(C#N)C#N | 12 | I |
| CC1=CC=C(NC(=O)C2=CC3=CC=C(F)C=C3N=C2C)N=C1 | 12 | I |
| FC1=CC=C(F)C(NC(=O)C2=CC3=CC=CC=C3OC2=O)=C1 | 12 | I |
| FC1=CC=C(C=C1)C(=O)NC1=CC=C2OC(F)(F)OC2=C1 | 12 | I |
| CC1=C(C)C2=C(OC1=O)C(C[NH+]1CCCC1)=C([O-])C(Cl)=C2 | 12 | I |
| [O-]C1=NC(C[NH+]2CC[C@H]3CCCC[C@@H]3C2)=NC2=C1SC=C2 | 12 | I |
| BrC1=NC(NC(=O)[C@H]2CC3=CC=CC=C3O2)=CC=C1 | 12 | I |
| ClC1=CC2=NC=CC(NC3CCS(=O)(=O)CC3)=C2C=C1 | 12 | I |
| OC1=CC=C(Br)C=C1\C=N/N1C=NC2=CC=CC=C12 | 12 | I |
| FC1=CC=CC=C1[C@@H]1C[C@H](N=C2N=CNN12)C1=CC=CS1 | 12 | I |
| CC1=C(OC=C1)[C@H](O)C1=CC2=C(OCCCO2)C=C1Cl | 12 | I |
| CN1C2=CC=CC=C2N=C1\C(=C/C1=CNC2=C1C=CC=C2)C#N | 12 | I |
| COC1=CC(Cl)=C(OC)C2=C1NC1=C(CCCC1)C2=O | 12 | I |
| CC1(CCCC1)NC(=O)C1=CC2=C(S1)N=C1SC=CN21 | 12 | I |
| CN1C=C(\C=C\C2=NC3=CC=CC=C3C(=O)N2)C2=C1C=CC=C2 | 12 | I |
| CC1=C([NH+]=C(O1)C1=CC(Br)=CC=C1F)C([O-])=O | 12 | I |
| CC1=C(SC=C1)C1=NN=C2[C@@H](NCCN12)C1=CC=CC=C1 | 12 | I |
| C1CC2=CC=CC=C2C2=NC3=[NH+]C=NN3[C@H]([C@H]12)C1=CC=CS1 | 12 | I |
| C[C@@H]1SC(=N)C(=C1C)C1=NC(=NO1)C1=CC=C(F)C(F)=C1 | 12 | I |
| CC1=CC(O)=C(\C=N\C2=CC=CC=C2Br)C(=O)O1 | 12 | I |
| CC1=CN\C(C=C1)=N/S(=O)(=O)C1=CC=C(C)C(Cl)=C1 | 12 | I |
| CCC1=C2NC(=CC(=O)N2N=C1)C1=CC=C(Br)C=C1 | 12 | I |
| [O-]C1=N[C@@H]([NH+]2CCCC[C@@H]12)C1=CC=C(C=C1)N1CCCCCC1 | 12 | I |
| ClC1=CC=C(C=C1)[C@H]1CC(=O)NC2=C1C=NN2C1CCCC1 | 12 | I |
| CC1=CC(NC(=O)C2=CC(C)=C(Br)S2)=NO1 | 12 | I |
| CCN1[C@H](NC2(CCCCC2)C1=O)C1=CC2=CC=CC=C2OC1 | 12 | I |
| CC(=O)C1=C2C3=C(C=CC=C3)C(=O)C3=C2C(NC1=O)=C(Cl)C=C3 | 12 | I |
| CC(C)[C@@H]1CN([C@@H](C)CN1)C1=CC=C2OC(F)(F)OC2=C1 | 12 | I |
| CC1=CC=C(C=C1Cl)N1CC(=O)N[C@H](C1=O)C(C)(C)C | 12 | I |
| CC(C)(C)[C@@H]1NC(=O)CN(C1=O)C1=CC(F)=C(F)C=C1F | 12 | I |
| BrC1=CN2C(C=C1)=NN=C2[C@@H]1NCCC2=CC=CC=C12 | 12 | I |
| C[C@@H]1CN[C@@H](CN1C1=C(Cl)C=CC2=NSN=C12)C1CC1 | 12 | I |
| CC1=CC(Cl)=CC=C1S(=O)(=O)\N=C1/NC=CC(Cl)=C1 | 12 | I |
| BrC1=C(NC2=CC=CC3=CC=CN=C23)N=CN=C1 | 12 | I |
| CC1(CC2=CC=CC=C2C1)NC(=O)C1=CC=CC2=C1OCCO2 | 12 | I |
| O[C@H]([C@@H]1CCOC2(CCC2)C1)C1=CC2=C(OCO2)C(Cl)=C1 | 12 | I |
| OC1=C(C=CC(Cl)=C1)C1=NC(=NO1)[C@@H]1CSCCS1 | 12 | I |
| C[C@H]1CCC[C@@H]1NC1=NN2C=CC=C(Br)C2=N1 | 12 | I |
| FC1=NC(F)=C(F)C(NC2=CC3=C(OCCO3)C=C2)=C1F | 12 | I |
| CN1N=C(C)C([C@@H](O)[C@@H]2CCOC3(CCCCC3)C2)=C1Cl | 12 | I |
| CC(C)N1N=CC2=C1N=C(O)C[C@H]2C1=C(F)C=CC=C1Cl | 12 | I |
| FC1=CC(CN2C=NC3=C(OC4=CC=CC=C34)C2=O)=CC=C1 | 12 | I |
| FC1=CC=C2O[C@H](CC(=O)C2=C1)C1=CC2=C(OCCCO2)C=C1 | 12 | I |
| CC1=C(C2=NCCO2)C(=NO1)C1=C(Cl)C=CC=C1Cl | 12 | I |
| CC(=C)CN1C(=O)C2(OCCO2)C2=CC(Br)=CC=C12 | 12 | I |
| ClC1=CC=C2N(CC3=CC=CC=C3)C(=O)C3(OCCCO3)C2=C1 | 12 | I |
| CC1(C)CN(C(=S)N2CC(C)(C)SC2=O)C(=O)S1 | 12 | I |
| FC1=CC=CC=C1C(=O)N1N2C(SC3=CC=CC=C23)=NC1=S | 12 | I |
| CC1=C(C(=O)C2=CC=C3OCCOC3=C2O1)C1=CC=C(F)C=C1 | 12 | I |
| C[C@H]1CN(C[C@H](C)O1)S(=O)(=O)C1=C(C)C=C(Cl)C(C)=C1 | 12 | I |
| CN1N=C(C(=O)N2CCCCCC2)C(Cl)=C1C(F)(F)F | 12 | I |
| ClC1=C2N(CC=C)C(=O)C3(OCCO3)C2=C(Cl)C=C1 | 12 | I |
| ClC1=C(Cl)C2=C(C=C1)C1(OCCO1)C(=O)N2CC=C | 12 | I |
| FC1=CC=C(C=C1)N1N=C(C(=O)N2CCCC2)C2=C1CCCC2 | 12 | I |
| C1[C@@H]2CC3(COC(OC3)C3=CC=C(C=C3)N3CCOCC3)[C@H]1C=C2 | 12 | I |
| C[C@@H]1CCC2=CC=CC=C2N1CC1=CC(=O)N2C=CC=CC2=N1 | 12 | I |
| CN1C(=NC2=CC=CC=C12)C(\C#N)=C1/C=CC(=CN1C)C(F)(F)F | 12 | I |
| CC1=NN=C(SC2=C3C4=C(CCCC4)SC3=NC=N2)S1 | 12 | I |
| C[C@H]1CN(CC2=CC(=O)OC3=C2C=C(Cl)C(C)=C3)C[C@@H](C)O1 | 12 | I |
| C[C@H]1CN(C[C@@H](C)O1)C(=O)CC1=COC2=C1C=C(C)C(C)=C2 | 12 | I |
| FC1=CC=C(C=C1Cl)N1C(=O)S\C(=C\C2=CC=CO2)C1=O | 12 | I |
| BrC1=C(OC2=C3C=CSC3=NC=N2)C=CC=N1 | 12 | I |
| CC(C)C1=NC=NC(=C1)N1N=C(C)C(Br)=C1C | 12 | I |
| C[C@H]1C[C@H]1C(=O)C1=CC2=C(C=C1Br)N(C)C(=O)N2C | 12 | I |
| N#CC(C#N)=C1CCCC[C@@H]1S[C@H]1CCCCC1=C(C#N)C#N | 12 | I |
| CC1=C(C)\C(C=CC1=O)=N\S(=O)(=O)C1=CC=C(Cl)C=C1 | 12 | I |
| BrC1=CC=C(S1)C1=NN=C(O1)C1=CC=CO1 | 12 | I |
| CC1=CC=C(C=C1)N1C=C(C#N)S(=O)(=O)C2=C1C=C(Cl)C=C2 | 12 | I |
| ClC1=NC(=NC2=C1C1=C(CCCC1)S2)C1=NC=NC=C1 | 12 | I |
| FC1=CC=C(C=C1)[C@@H]1CC(=NC2=NC=NN12)C1=CC=C(F)C(F)=C1 | 12 | I |
| CN([C@@H]1CCC2=C1C=CC=C2)C(=O)[C@H]1CC2=CC=CC=C2C(=O)O1 | 12 | I |
| CC(C)N1C(=O)[C@@H]2[C@@H]3C[C@H]4[C@H](O[C@]1([C@H]24)C1CCCCC1)C3=O | 12 | I |
| CC1=NN(C2=C1[C@H]1[C@H](CO2)COC2=CC=CC=C12)C1=CC=CC=C1 | 12 | I |
| C[C@@H]1CCN([C@H](C)C1)C(=O)C1=C(C)C2=C(CC(C)(C)CC2=O)O1 | 12 | I |
| C[C@@H](N(C)C(=O)N1C=CN=C1)C1=CC=C(Cl)C=C1Cl | 12 | I |
| BrC1=CC(N2C=C(C=O)N=N2)=C(Br)C=C1 | 12 | I |
| C[C@H]1O[C@H](C)[C@H]([C@H](Cl)C2=CC=C3N(C)C(=O)OC3=C2)[C@H]1C | 12 | I |
| FC1=C(Br)C=CC(=C1)N1N=NN=C1C1CCCC1 | 12 | I |
| CC(C)C1=C(Br)C(Cl)=NC(=N1)C1=NN(C)C=C1 | 12 | I |
| CN1CCN(CC1)C1=NC(C2CC2)=C(Br)C(Cl)=N1 | 12 | I |
| O=CC1=CC2=C(C=C1)N=CC=C2C1=C2OCCOC2=CS1 | 12 | I |
| CC[C@H]1CO[C@H](C)CN1C1=C(Br)C=NC(Cl)=N1 | 12 | I |
| ClCC1=NC2=C(N=CC=C2)N1C1=CC=CC2=C1C=CC=N2 | 12 | I |
| C[C@@H](Cl)C1=NC2=C(N(C)N=C2C)N1C1=CC=C(F)C=C1F | 12 | I |
| CN1N=C(C)C2=C1N(C(CCl)=N2)C1=CC(F)=CC=C1F | 12 | I |
| CN1N=C(C)C2=C1N(C(CCl)=N2)C1=CC(Cl)=CC=C1F | 12 | I |
| C[C@H](Cl)C1=NC2=CC=CC(Cl)=C2N1C1=CN(C)N=C1 | 12 | I |
| C[C@H]1N(C(=O)[C@H]2CCCCN2C1=O)C1=CC=C(Cl)C(Cl)=C1 | 12 | I |
| C[C@@H]1CN2CCCC[C@@H]2CN1C1=C(CCl)N2C=CSC2=N1 | 12 | I |
| CC1=CC(C)=C(\C=C(\C#N)C2=NN=C3CCCCCN23)C=C1C | 12 | I |
| CC1=CC=CC=C1S(=O)(=O)\N=C1/C=CC(=O)C(Cl)=C1 | 12 | I |
| C[C@H]1C[C@@H]2[C@@H]3CC[C@H](C(C)=O)[C@]3(C)C[C@H](O)[C@@H]2[C@@]2(C)CCC(=O)C=C12 | 12 | I |
| CC1=CC=C(S1)C1=CSC2=C1C(=O)NC(CCl)=N2 | 12 | I |
| OC1=C(Br)C=C2OC(=O)SC2=C1Br | 12 | I |
| C[C@@]12CC[C@H]3[C@@H](CC=C4C[C@H](O)CC[C@]34C)[C@H]1C[C@@H](C=O)C2=O | 12 | I |
| C\C=C\C(=O)NC1=NC2=CC=C(Br)C=C2S1 | 12 | I |
| CO\C=C1/C[C@@]2(C)[C@@H](CC[C@@H]3[C@@H]4CC[C@H](O)[C@@]4(C)CC[C@H]23)CC1=O | 12 | I |
| ClC1=CC2=C(N[C@H]([C@@H]3CCCO[C@H]23)C2=CC=NC=C2)C=C1 | 12 | I |
| C[C@@]12CCC(=O)[C@H](O)[C@@H]1CC[C@@H]1[C@@H]2CC[C@@]2(C)[C@H]1CCCC2=O | 12 | I |
| [O-]C1=C(C[NH+]2CCOCC2)C(Cl)=C(Cl)C=C1Cl | 12 | I |
| C[C@]12CC[C@@H]3[C@H](CC[C@H]4C(=O)C(O)=CC[C@]34C)[C@H]1CCC2=O | 12 | I |
| CN[C@@H]1[C@H](C)CS(=O)(=O)C2=CC=C(Br)C=C12 | 12 | I |
| CN[C@H]1[C@H](C)CS(=O)(=O)C2=CC=C(Br)C=C12 | 12 | I |
| O[C@@H]1CS(=O)(=O)C[C@H]1SC1=CC2=CC=CC=C2C=C1 | 12 | I |
| CC1=C2OC(=O)SC2=C(Br)C(O)=C1Br | 12 | I |
| FC1=CC=C(C=C1)[C@H]1SC2=CC=CC=C2NC2=C1C(=O)OC2 | 12 | I |
| O[C@@H](C1=CSC=C1)C1=CC2=C(OCCCO2)C=C1Cl | 12 | I |
| FC1=CC=CC=C1[C@@H](Cl)C1=CC=C2OCC(=O)NC2=C1 | 12 | I |
| C[C@@H]1CC[C@@]23CCC(=O)[C@@H]2[C@]1(C)[C@H](O)C[C@@](C)(C=C)C(=O)[C@@H]3C | 12 | I |
| C[C@H](N1C(=S)NC2=C(F)C=CC=C12)C1=C(C)OC(C)=C1 | 12 | I |
| C[C@H](N1C(=S)NC2=CC=C(F)C(F)=C12)C1=CC=C(C)O1 | 12 | I |
| ClC1=CC=C2N(C3CC3)C(=NC2=C1)[C@@H]1SC(=N)C=C1 | 12 | I |
| C[C@@H](Br)C(=O)C1=CC=C2NC(=O)C(C)(C)C2=C1 | 12 | I |
| FC1=C(F)C=C2N=C(NC2=C1)C1=NC=C(Br)C=C1 | 12 | I |
| CC(C)NC(=O)C1=CC2=CC(Br)=CC=C2OC1 | 12 | I |
| C[C@@H]1CCC[C@H]1N1C(=S)NC2=C(C3=C(CCCC3)S2)C1=O | 12 | I |
| O[C@@H]1CC2=CC=CC=C2[C@H]1C1=CC=C2OC(=NC2=C1)C1CC1 | 12 | I |
| CC1=NNC(=S)N1C1=C(F)C=C(Br)C=C1F | 12 | I |
| BrC1=CC(=CC2=C1OCO2)[C@H]1NCCCS1 | 12 | I |
| C[NH+](C)C1=CC=C(I)C=C1C([O-])=O | 12 | I |
| CC1(C)CNC[C@@H](O1)C1=CC2=CC(Br)=CC=C2O1 | 12 | I |
| FC1=CC=CC(N2C(=S)NC3=CC=C(Br)C=C23)=C1C#N | 12 | I |
| C[C@H]1CN[C@@H](CS1(=O)=O)C1=CC(F)=C(Cl)C=C1Cl | 12 | I |
| C[C@H]1CCS(=O)(=O)C[C@H](N1)C1=CC=C(F)C(Br)=C1 | 12 | I |
| C[C@H]1CCS(=O)(=O)C[C@@H](N1)C1=CC=C(Br)C=C1 | 12 | I |
| C[C@H]1CN[C@H](CS(=O)(=O)C1)C1=CC=C(C=C1)C(C)(C)C | 12 | I |
| CC1=CC=C(C=C1F)C1=NC(C)=C(Br)C(=O)N1 | 12 | I |
| C[C@@H]1CC[C@@H](C[C@@H]1C)[NH+]1[C@@H]2CCCC[C@@H]2CC[C@@H]1C([O-])=O | 12 | I |
| FC1=CC(=CC(F)=C1)N1C(=S)NC2=CC(Br)=CN=C12 | 12 | I |
| CN[C@@H]1[C@H](C)[C@@H](C)S(=O)(=O)C2=CC=C(C=C12)C(C)(C)C | 12 | I |
| CNC1=NC(C)=NC(=C1)C1=CC(Br)=CC=C1F | 12 | I |
| C[C@@H]1CC(C)(C)C[C@](O)(C1)[C@@H]1CCOC2(CCOCC2)C1 | 12 | I |
| O[C@H]([C@H]1CCOC2(CCCCC2)C1)C1=NC=C(Cl)C=C1 | 12 | I |
| C[C@H]1CN(CC2=NC(C)=C(C)S2)C2(CCCCC2)CN1 | 12 | I |
| C[C@@H](Cl)C1=NC2=C(C3=C(S2)[C@@H](C)C[C@H](C)C3)C(=O)N1 | 12 | I |
| O[C@H]1C[C@H](OC2=C1C=C(Br)C=C2)C1=CSC=N1 | 12 | I |
| O[C@@H]1[C@@H](CC2=CC=CC=C12)N1CCO[C@@H](C1)C1=CC=CC=C1 | 12 | I |
| FC1=C(C=CC(Br)=C1)C1=CC2=C(S1)C(=O)N=CN2 | 12 | I |
| C[C@H](N1C(=S)NC2=CC(Cl)=C(F)C=C12)C1=CC=CO1 | 12 | I |
| C[C@@H](N1C(=S)NC2=CC(Cl)=C(F)C=C12)C1=CC=CO1 | 12 | I |
| OC1=NC2=C(SC=C2)[C@@H](SC1)C1=CC2=CC=CC=C2OC1 | 12 | I |
| CC1=CSC2=NC=C(C(=O)NC3=CC=CC4=C3C=CC=C4)C(=O)N12 | 12 | I |
| FC1=CC(F)=C2N=C(NC(=O)[C@H]3COC4=CC=CC=C4O3)SC2=C1 | 12 | I |
| CC1=CC=C(O1)\C=C1/C(=O)NC(=S)N(C1=O)C1=CC=CC(Cl)=C1C | 12 | I |
| CC1=NC2=C(C3=C(CCCC3)S2)C(=O)N1\N=C\C1=CC=C(O)C=C1 | 12 | I |
| C[C@@H]1CC2=CC=CC=C2N1C(=O)N[C@@H]1CN(C(=O)C1)C1=CC=C(C)C=C1 | 12 | I |
| CC(=O)NC1=C(Cl)C=C(C=C1Cl)N1C(=O)[C@H]2[C@H]3C[C@H](C=C3)[C@H]2C1=O | 12 | I |
| CN1C(=O)C=C(NC2=CC=C(Cl)C(=C2)C(F)(F)F)N(C)C1=O | 12 | I |
| CC1=C(SC=C1)C(=O)NC1=NC2=CC3=C(OCCO3)C=C2S1 | 12 | I |
| CN1C(=O)C2=CC=CC(NC(=O)C3=CC=C(Br)S3)=C2C1=O | 12 | I |
| CC(C)[C@@H]1N(CCN2C(C)=CC=C12)C(=O)C1=CC2=C(CCCCC2)NC1=O | 12 | I |
| CC1=NOC(=C1)C(=O)NC1=NC2=CC=C(Br)C=C2S1 | 12 | I |
| CC(=O)N1CCC2=C1C=CC(NC(=O)C1=CC3=CC(Cl)=CC=C3OC1)=C2 | 12 | I |
| FC1=CC=CC(F)=C1NC(=O)N1CCN2C=CC=C2[C@H]1C1=CC=CN=C1 | 12 | I |
| FC1=CC=C(\C=C2\C(=O)NC(=O)N(C2=O)C2=CC=CC3=CC=CC=C23)C=C1 | 12 | I |
| O=C1N[C@]2(CCCC3=C2C=CC=C3)C(=O)N1CN1CCC2=CC=CC=C2C1 | 12 | I |
| ClC1=CC(Cl)=C(C=C1)C(=O)NC1=CC=C(N=C1)N1C=CN=C1 | 12 | I |
| CC1CCC(CC1)NS(=O)(=O)C1=C(Cl)N=C2SC=CN12 | 12 | I |
| CC1=CC(=CC=C1Br)S(=O)(=O)NC1=NN=CS1 | 12 | I |
| CC1=NN(C2=C1C(=O)NC(=N2)C(Cl)(Cl)Cl)C1=CC=CC=C1 | 12 | I |
| CN1N=CC(C)=C1C(=O)NC1=NC2=C(S1)C=CC=C2Br | 12 | I |
| CN(C)C1=CC=C(C=C1)C(=O)NC1=CC2=C3N(CCC2)C(=O)CC3=C1 | 12 | I |
| FC1=CC=C(C=C1)C1=C(NC(=O)[C@H]2CCCO2)N2C=CSC2=N1 | 12 | I |
| CC1=CN\C(C=C1)=N/S(=O)(=O)C1=CC(Br)=CN=C1Cl | 12 | I |
| FC1=CC=CC=C1NC(=O)CN1C(=O)COC2=CC(Cl)=CC=C12 | 12 | I |
| C[C@@H]1COCCN1C(=O)NCC1=CC=C(C=C1)N1CCSCC1 | 12 | I |
| CNCC1=C(N=C2C=CC=CN12)N1N=C(C)C(Br)=C1C | 12 | I |
| CN(C)C(=O)C1=C(C)NC2=NC3=CC=CC=C3N2[C@H]1C1=CC=CC=C1F | 12 | I |
| C[C@@H]1CN(C[C@@H](C)O1)C(=O)NC1=CC=C(C=C1C)N1CCSCC1 | 12 | I |
| C[C@H]1C[C@@H](C)CN(C1)C(=O)N[C@H]1CN(C(=O)C1)C1=CC=C(F)C=C1 | 12 | I |
| CN1C(=O)OC2=CC(NC(=O)C3=CC=C(Br)C=C3)=CC=C12 | 12 | I |
| C[C@H]1CN([C@H](C)CO1)C(=O)C1=C(Br)C(=NN1)C1=CC=CC=C1 | 12 | I |
| COC1=C(F)C=C(C=C1F)[C@@H]1CC(=O)NCC2=C1N1C=CC(C)=CC1=N2 | 12 | I |
| CC1=CC(C)=C2C(=C1)N=C(C)C=C2C(=O)NC1=CN=C2CCCCN12 | 12 | I |
| C[C@@H]1CN([C@@H](C)CO1)C(=O)C1=C(Br)C(=NN1)C1=CC=CC=C1 | 12 | I |
| CC1=CC(=O)NC(SCC(=O)N2C[C@]3(C)C[C@H]2CC(C)(C)C3)=N1 | 12 | I |
| FC1=CC(NC(=O)CN2C(=O)C=NC3=CC=CC=C23)=CC=C1Cl | 12 | I |
| C[C@@]12C[C@@H](CC(C)(C)C1)N(C2)C1=C2CNCC2=NC(=N1)C1=CC=NC=C1 | 12 | I |
| C[C@H]1CC2=C(O1)C(Cl)=CC(=C2)S(=O)(=O)\N=C1/C=CNC(C)=C1 | 12 | I |
| CC1=CC=C(C(F)=C1)S(=O)(=O)OC1=CC=C2NC(=O)CCC2=C1 | 12 | I |
| CN1C(=O)C2=CC=C(NC(=O)C3=C(F)C(F)=C(F)C(F)=C3F)C=C2C1=O | 12 | I |
| CC1=NNC(CCl)=C1S(=O)(=O)N1CC[C@H]2CCCC[C@@H]2C1 | 12 | I |
| CC1=CC=C(C=C1NC(=O)C1=CC2=CC(F)=CC=C2O1)C1=NC=CO1 | 12 | I |
| ClC1=CC=C(NC(=O)[C@H]2COC3=C2C=C(Br)C=C3)N=N1 | 12 | I |
| O=C(NC[C@H]1CN2CCCC[C@H]2CO1)N1CCC2(CCCC2)CC1 | 12 | I |
| COCC1=C(Br)C(=O)NC(=N1)C1=CC2=CC=CC=C2O1 | 12 | I |
| ClC1=CC2=C(NC3=C2CN(CC2=CN=C4C=NC=CN24)CC3)C=C1 | 12 | I |
| CN1N=NC2=CC(=CC=C12)C(=O)NC[C@H]1C[C@]11CCC2=C1C=CC=C2 | 12 | I |
| ClC1=CC(N2CC[C@H](C(=O)\N=C3/NCCS3)C2=O)=C(Cl)C=C1 | 12 | I |
| CC1=C(Br)C=C\C(N1)=N\S(=O)(=O)C1=CC=CN=C1Cl | 12 | I |
| O=C(NC[C@@H]1CC2=CC=CC=C2CO1)C1=CC2=CC=CC=C2OC1=O | 12 | I |
| FC1=CC=C(NC(=O)C2=CC3=CC=CC=C3C(=O)O2)C=C1 | 12 | I |
| CC1=CC=C(C(=O)NC2=C3C=CSC3=NC=N2)C(C)=C1 | 12 | I |
| OC1=CC=C2C=C(C(=O)C3=CC=C(F)C=C3)C(=O)OC2=C1 | 12 | I |
| [O-][N+](=O)C1=CC2=C(NC=C2I)C=C1 | 12 | I |
| C[C@@H]1SC(=N)C(=C1C)C1=NC(=NO1)[C@H]1CCC[C@H](C)C1 | 12 | I |
| CC1=NC2=CC(NC(=O)C3=CC=CC(F)=C3F)=CC=C2O1 | 12 | I |
| N=C1S[C@H]2CCCC2=C1C1=NN=CN1C1CCCCC1 | 12 | I |
| C[C@@H]1SC(=N)C(=C1C)C1=NC(=NO1)[C@@H]1CCC[C@@H](C)C1 | 12 | I |
| CC(C)N1C=C(C=N1)N1C(C)=CC2=C1CC(C)(C)C[C@H]2O | 12 | I |
| CC1=CS\C(N1)=N/S(=O)(=O)C1=CC=CC(F)=C1F | 12 | I |
| CC1=C\C(NC=C1)=N\S(=O)(=O)C1=CC=C(F)C=C1F | 12 | I |
| CC[C@]1(C)C[C@](O)(CCO1)C1=CC=C2OCCCOC2=C1 | 12 | I |
| C[C@@H]1CCCN[C@H]1CN1C(=O)CC2(CCCCCC2)C1=O | 12 | I |
| CC1=C\C(NC=C1)=N\S(=O)(=O)C1=C(F)C=CC=C1F | 12 | I |
| CCC1=C([C@@H]2N=C([O-])[C@H]3CCCC[NH+]23)C2=CC=CC=C2O1 | 12 | I |
| OC1=CC=C2C(=O)\C(OC2=C1)=C\C1=CC2=C(OCC2)C=C1 | 12 | I |
| BrC1=CC=C2N=C(NC(=O)C2=C1)C1=CC=CO1 | 12 | I |
| COC1=CC(\C=C2\COC3=CC=CC=C3C2=O)=CC=C1O | 12 | I |
| IC1=CC=C(CC2=NN=NN2)C=C1 | 12 | I |
| CC1=CC=C(CN2CC(=O)NC3(CCCCC3)C2=O)C=C1 | 12 | I |
| FC1(F)OC2=C(O1)C=C(C=C2)N1CCN[C@H](C1)C1CC1 | 12 | I |
| CC(C)(C)[C@@H]1CN(CCCN1)C1=CC=C2OCCOC2=C1 | 12 | I |
| CC[C@H]1NC(=O)CCN(C1=O)C12CC3CC(CC(C3)C1)C2 | 12 | I |
| C[C@H]1CCC[C@@H]([C@@H]1C)N1[C@H](C)C(=O)N[C@@](C)(C2CC2)C1=O | 12 | I |
| C[C@H]1CCC[C@@H]([C@@H]1C)N1CCC(=O)N[C@@](C)(C2CC2)C1=O | 12 | I |
| C[C@H]1CCC[C@@H](C1)N1[C@H](C)C(=O)NC2(CCCCC2)C1=O | 12 | I |
| CC[C@H]1NC(=O)[C@@H](C)N(C1=O)C1=CC=C(C)C(Cl)=C1 | 12 | I |
| CC[C@H]1NC(=O)[C@@H](C)N(C1=O)C1=CC=CC(Cl)=C1C | 12 | I |
| CCC1CCC(CC1)N1[C@@H](C)C(=O)NC2(CCCC2)C1=O | 12 | I |
| C[C@H]1CC[C@@H]([C@H](C)C1)N1[C@H](C)C(=O)N[C@@](C)(C2CC2)C1=O | 12 | I |
| CC[C@@H]1CC[C@@H]([C@@H]1C)N1[C@H](C)C(=O)NC2(CCCC2)C1=O | 12 | I |
| CC[C@H]1CC[C@@H]([C@@H]1C)N1[C@H](C)C(=O)NC2(CCCC2)C1=O | 12 | I |
| C[C@H]1CN(C(=O)NC1=O)C1=CC=C(Br)C=C1 | 12 | I |
| C[C@@H]1O[C@H](C)[C@H]([C@H](O)C2=CC=C3OCCCOC3=C2)[C@@H]1C | 12 | I |
| C[C@@H]1CS\C(N1)=N/C1=C(Cl)C=CC2=NSN=C12 | 12 | I |
| CC1=C(C)N=C(N[C@@H]2CCN3CCCC[C@H]23)C(Cl)=N1 | 12 | I |
| C[C@H]1N([C@H]2CCCCC[C@H]2C)C(=O)[C@@](C)(NC1=O)C1CC1 | 12 | I |
| CC(C)[C@H]1NC(=O)[C@@H](C)N([C@@H]2CCCCC[C@H]2C)C1=O | 12 | I |
| CSC1=NC(SC)=C2C(Br)=NNC2=N1 | 12 | I |
| CC1=NN=C([C@@H]2CC[C@H]3CCCC[C@@H]3N2)N1C1CCCC1 | 12 | I |
| CC1=CC2=C(CC(C)(C)C[C@@H]2O)N1CC1=C(C)ON=C1C | 12 | I |
| C[C@@H](N1C(=S)NC2=C1N=CC(C)=C2)C1=C(C)OC(C)=C1 | 12 | I |
| CCC1=NN(C)C2=C1NC(=S)N2C1=CC=C(C)C(C)=C1 | 12 | I |
| CCC1=NN(C)C2=C1NC(=S)N2C1=CC=CC(C)=C1C | 12 | I |
| CCC1=NN(C)C2=C1NC(=S)N2C1=CC(C)=CC(F)=C1 | 12 | I |
| CC(C)[C@H]1NC(=O)[C@@H](C)N([C@@H]2CCCCC2(C)C)C1=O | 12 | I |
| C[C@H](O)C1=C(C)N(N=C1C)[C@@H]1CCOC2(CCCCC2)C1 | 12 | I |
| CN(C)C1=CC=C(C=C1)[C@H]1NCCC2=NC3=CC=CC=C3N12 | 12 | I |
| OC1=CC=CC=C1N1C(=O)CS\C1=N\C1=CC=CC=C1 | 12 | I |
| CC(C)N1N=CC2=C1N=C(O)C[C@@H]2C1=CC=CC=C1Cl | 12 | I |
| CC1=CC(=CC(C)=C1)N1C(=O)NC(=O)\C(=C/C2=CSC=C2)C1=O | 12 | I |
| CC(=O)NC1=CC=C(C=C1)[C@H]1[C@H]2[C@@H](CCCC2=O)OC2=C1C(=O)CCC2 | 12 | I |
| CC(=O)NC1=CC=C(C=C1)[C@@H]1[C@H]2[C@H](CCCC2=O)OC2=C1C(=O)CCC2 | 12 | I |
| C[C@H](N1C(=O)OC2=CC=CC=C12)C(=O)NC1=CC=C(F)C=C1F | 12 | I |
| BrC1=CC=CC=C1C(=O)NC1=NN=C2SCCCN12 | 12 | I |
| CC1=C(C2=CN3C(=N2)N=NC2=C3CCCC2)C2=CC(F)=CC=C2N1 | 12 | I |
| C[C@@H](NC(=O)C1=C(C)C2=C(C)N=C(C)N=C2S1)[C@@H]1CCCO1 | 12 | I |
| CC1=NN2C=NC(=CC2=C1Br)C(=O)NC1=CC=CC=C1F | 12 | I |
| ClC1=CC2=C(OCCO2)C=C1NC1CCS(=O)(=O)CC1 | 12 | I |
| CC1=C(Br)C=CC(NC(=O)C2=CC=C3OCCOC3=C2)=N1 | 12 | I |
| CN1C(=O)[C@]2(C)CC(C)(C[C@@](C)(C2)C1=O)C(=O)NC1=CC=C(C)C=C1 | 12 | I |
| CSC1=NC(=O)[C@@H]2[C@H](CC(=O)NC2=N1)C1=CC=C(Cl)C=C1Cl | 12 | I |
| CSC1=NC(=O)[C@H]2[C@H](CC(=O)NC2=N1)C1=CC=C(Cl)C=C1Cl | 12 | I |
| CNC1=C2C(C)=NN(C2=NC=N1)C1=CC=C(Br)C=C1 | 12 | I |
| CC1=CN\C(S1)=N/S(=O)(=O)C1=CC(Br)=CN=C1 | 12 | I |
| CC1=NC2=NC=NN2C(NCC2=CC=C(Br)C=C2)=C1 | 12 | I |
| C[C@@H](NC(=O)C1=CC(Cl)=CC=C1F)C1=NN=C2C=CC=CN12 | 12 | I |
| O=C1N[C@H]2[C@H]3[C@@H]([C@@H](SC2=N1)C1=CC=CC=C1)C(=O)OC1=CC=CC=C31 | 12 | I |
| CC1=C(OC2=CC=C(Br)C=C12)C(=O)N1CCNC(=O)CC1 | 12 | I |
| C[C@@H](NC(=O)C1=CC(F)=C(Cl)C=C1Cl)C1=NC(C)=NO1 | 12 | I |
| O=C1NC2=CC=C(C=C2S1)S(=O)(=O)N1CCC2=CC=CC=C12 | 12 | I |
| CC1=NN=C(O1)C1=CC(NC(=O)C2=C(C)C=CS2)=C(C)S1 | 12 | I |
| CNC1=C2C=NN(CC3=CC=CC(Br)=C3)C2=NC=N1 | 12 | I |
| CC1=CC=C(CN2C=CC=C\C2=N/C(=O)C2=CNC(=O)C=C2)C=C1 | 12 | I |
| FC1=CC=C(NC(=O)C2=CSC(=N2)C2=NC=CC=N2)C(F)=C1 | 12 | I |
| O=C1NC2=CC=CC=C2[C@]11CCCN(CC2=CC=C3OCCOC3=C2)C1 | 12 | I |
| C[C@@H]1CN(CCO1)C(=O)NC1=CC=C(C=C1)N1CCSCC1 | 12 | I |
| CC1=C(N=C(S1)C1=CC=NC=C1)C1=CC=C2OCC(=O)NC2=C1 | 12 | I |
| FC1=CC(Br)=C(C=C1)C(=O)NC1=CC=NC2=CC=NN12 | 12 | I |
| CN(C)C(=O)C1=CC=C(C=C1)C(=O)NC1=CC(Cl)=CC=C1[O-] | 12 | I |
| CN(CC1=CC=C(Cl)N=C1)C(=O)NC1=CC=C(F)C(=C1)C#N | 12 | I |
| CS(=O)(=O)C1=CC=C2N(CCCC2=C1)C(=O)C1=CC=C2C=CNC2=C1 | 12 | I |
| FC1=CC=C(C=C1)N1N=CC(=N1)C(=O)NC1=CC(F)=CC(F)=C1 | 12 | I |
| CC1(C)[C@@H](NC(=O)C2=CSC(=N2)C2=COC=C2)[C@H]2CCO[C@H]12 | 12 | I |
| CC1=C(ON=C1)C(=O)NC1=CC=C(I)C=N1 | 12 | I |
| CN1C(C)=[NH+]C2=CC([N-]S(=O)(=O)C3=CC=CC=C3F)=CC=C12 | 12 | I |
| CCC1=C(I)C(Cl)=NC(=N1)C1=NNC=N1 | 12 | I |
| C[C@@H]1CN(CC2=CC(=O)N3C=C(Br)C=CC3=N2)[C@H](C)CN1 | 12 | I |
| C[C@H]1CN(CC2=CC(=O)N3C=C(Br)C=CC3=N2)[C@@H](C)CN1 | 12 | I |
| FC1=C(C#N)C(=CC=C1)S(=O)(=O)\N=C1/NC=CC=C1Br | 12 | I |
| CN1C=CC=C1[C@@H]1CCCN1C(=O)C1=NNC(C2CC2)=C1Cl | 12 | I |
| OC[C@H]1CCCCN1S(=O)(=O)N1CC[C@@H]2CCCC[C@H]2C1 | 12 | I |
| ClCC1=C(NN=C1)S(=O)(=O)N1CCC[C@@H]2CCCC[C@H]12 | 12 | I |
| CC1=NNC(=C1CCl)S(=O)(=O)N1CCC[C@H]2CCC[C@H]12 | 12 | I |
| FC1=CC=C2OCC(=CC2=C1)C(=O)N1CC(=O)NC2=CC=CC=C12 | 12 | I |
| C[C@H](NC(=O)[C@H]1COC2=CC=C(F)C=C2C1)C1=C(C)ON=C1C | 12 | I |
| ClC1=CC(=CC2=C1OCCO2)C1=NC(=O)C(Br)=CN1 | 12 | I |
| COC1=CC=C(C=C1)N1C=NC2=C1N=C(O)C[C@@H]2C1=CC=CC=C1 | 12 | I |
| CC1=CN2C(S1)=NC(C)=C2\C=C/C(=O)NC1=NC(C)=CS1 | 12 | I |
| FC1=CC=C(C=C1)N1C(=O)[C@H]2ONC(=C2C1=O)C1=CC=C(Cl)C=C1 | 12 | I |
| CC1=CC(C=C2C(=O)C3=CC=CC=C3C2=O)=C(C)N1C1=CC=C(O)C=C1 | 12 | I |
| O=C1CS[C@@]2(N1C1=CC=CC3=CC=CC=C13)C(=O)NC1=CC=CC=C21 | 12 | I |
| BrC1=CC(=COC1=O)C(=O)NC1=CC=CC2=CC=CC=C12 | 12 | I |
| CC1=NC=C(N=C1)C(=O)NC1=CC=C(I)C=C1C | 12 | I |
| BrC1=CC2=C(OCCO2)C=C1NC(=O)[C@H]1CCC=CC1 | 12 | I |
| O=C1N[C@]2(CCC3=C2C=CC=C3)C(=O)N1CC1=CC=CC2=CC=CC=C12 | 12 | I |
| CC1=CC(C(=O)NC2=C(Cl)C=C(Cl)C=N2)=C2C=C(F)C=CC2=N1 | 12 | I |
| FC1=CC=CC=C1\C=C1\C(=O)NN(C1=O)C1=CC=C(Br)C=C1 | 12 | I |
| CN1C=C(Br)C=C1C(=O)NC1=CC=C2N=C(C)SC2=C1 | 12 | I |
| BrC1=CC=C(N\C=C2/C(=O)OC3=CC=CC=C3C2=O)C=C1 | 12 | I |
| CC1=CC(NC(=O)C2=CC3=C(CS2)C2=C(CCCC2)S3)=NO1 | 12 | I |
| C[C@H]1N(CCN2C=CC=C12)C(=O)NC1=CC=C(Br)C=C1 | 12 | I |
| O=C(NC1=CC=CC2=CC=CC=C12)C1=CN2CCC3=C2C(=CC=C3)C1=O | 12 | I |
| CC1=C\C(NC=C1)=N\S(=O)(=O)C1=CC(Br)=CC=C1C | 12 | I |
| CN1C=C(Br)C=C1C(=O)NC1=C(C#N)C2=C(CCC2)S1 | 12 | I |
| CN1C=CC(=N1)C(=O)NC1=CC=C(Br)C=C1Br | 12 | I |
| [O-]C1=NC(=S)S\C1=C/C1=CC2=CC=CC=C2[NH+]=C1N1CCCCC1 | 12 | I |
| FC1=CC=C(C=C1)C1=NC(=CS1)C1=CC=C2OCC(=O)NC2=C1 | 12 | I |
| OC1=CC=C(C=C1)N1C(=O)S\C(=C/C2=CC=CC3=CC=CC=C23)C1=O | 12 | I |
| O[C@@H](C1=CC=C(Br)O1)C1=CC2=C(OCCO2)C=C1Cl | 12 | I |
| ClC1=CC=C(C=C1Cl)N1C(=N)S\C(=C/C2=CC=CO2)C1=O | 12 | I |
| O=C1N(CC2=CC=CO2)[C@@]2(CCCC3=CC=CC=C23)NC2=CC=CC=C12 | 12 | I |
| CC1=NC2=CC=C(NC(=O)C3=CC=CC(Br)=C3)C=C2O1 | 12 | I |
| CC1=C\C(C=CN1)=N\S(=O)(=O)C1=CC=C(Br)S1 | 12 | I |
| CC1=CC(=CC=C1I)C(=O)NC1=NN=CS1 | 12 | I |
| FC1=C(C=C(Br)C=C1)N1CC(=O)NC2(CCCC2)C1=O | 12 | I |
| CC[C@@]1(C)NC(=O)[C@H](C)N(C2=CC=C(Br)C(C)=C2)C1=O | 12 | I |
| CC(C)[C@H]1N(C2=CC=C(Br)C=C2)C(=O)C(C)(C)NC1=O | 12 | I |
| CC(C)[C@@H]1N(C(=O)C(C)(C)NC1=O)C1=CC=C(F)C=C1Br | 12 | I |
| CC[C@H]1N(C(=O)[C@@H](C)NC1=O)C1=CC=C(Br)C=C1Cl | 12 | I |
| CC[C@H]1NC(=O)[C@@H](C)N(C1=O)C1=CC(Br)=CC=C1Cl | 12 | I |
| C[C@H]1CN(CCN1)S(=O)(=O)C1=C(Cl)C=C(Cl)C=C1Cl | 12 | I |
| FC1=C(Br)C=CC(NC(=O)C2=CC=C(Cl)N=N2)=C1 | 12 | I |
| C[C@@H]1CCC2=C(C1)SC=C2C(=O)NN1C=C(Br)C=N1 | 12 | I |
| C[C@@H]1CCN[C@@H](CS1(=O)=O)C1=CC2=CC(Br)=CC=C2O1 | 12 | I |
| IC1=C(NC2=CC=CC3=C2C=CC=N3)N=CN=C1 | 12 | I |
| CC1=C(Br)C=CC(NC(=O)C2=CC=C3OCCC3=C2)=N1 | 12 | I |
| C[C@@H](N1C(=S)NC2=CC(Br)=CC=C12)C1=C(C)ON=C1C | 12 | I |
| CN1C=CC(C)=C(NC(=O)C2=CC(C)=C(Br)C=C2C)C1=O | 12 | I |
| FC1=CC(Br)=CC(CN2C(=O)NC(=O)C22CCCC2)=C1 | 12 | I |
| O[C@H]([C@H]1CCOC2(CCC2)C1)C1=CC2=C(OCO2)C(Br)=C1 | 12 | I |
| ClC1=CC=C(N=N1)C(=O)NC1=CC(Br)=C(Cl)C=C1 | 12 | I |
| OC1=NC2=C(N=CN2C2=CC=CC=C2F)[C@H](C1)C1=CC=C(F)C=C1F | 12 | I |
| OC1=NC2=C(N=CN2C2=CC=C(F)C=C2)[C@@H](C1)C1=CC(F)=CC=C1F | 12 | I |
| CN(C(=O)C1=CC=CC2=C1NC1=C(C=CC=C1F)C2=O)C1=CC=CC=C1 | 12 | I |
| BrC1=CN=C2N([C@H]3CCOC4=CC=CC=C34)C(=S)NC2=C1 | 12 | I |
| CC1=NN(C(C)=C1[C@H]1SCC(O)=NC2=C1SC=C2)C1=CC=CC=C1 | 12 | I |
| CN1N=C(C)C2=C1N=C(O)CS[C@H]2C1=CC(=CC=C1F)C(F)(F)F | 12 | I |
| OC1=NCC2=NC3=CC=C(Br)C=C3[C@H](N2C1)C1=CC=CC=C1 | 12 | I |
| ClC1=CN\C(C=C1)=N/S(=O)(=O)C1=CC=C(Br)C=C1 | 12 | I |
| FC1=NC=C2N=CN=C(NC3=CC(Br)=CC=C3)C2=C1 | 12 | I |
| BrC1=CC=C(NC(=O)C2=CC3=CC=CC=C3OC2=O)C=C1 | 12 | I |
| CN1N=CC(Br)=C1C(=O)NC1=CC=CC2=C1C=CC=C2 | 12 | I |
| CC1=CC=C(S1)C1=N\C(=C\C2=C(C)NC3=C2C=CC=C3)C(=O)O1 | 12 | I |
| FC1=CC=C([C@@H]2[C@@H]3CCCC=C3[C@@H](C#N)C(=N)C2(C#N)C#N)C(F)=C1 | 12 | I |
| CC1=CC2=C([C@H](SC3=CC=CC=C3N2)C2=CC=CO2)C(=O)O1 | 12 | I |
| CC1=CC=C(NC(=O)C2=CC(=O)C3=CC=CC=C3O2)C(Cl)=C1 | 12 | I |
| FC1=CC=C([C@H]2[C@@H]3CCCC=C3[C@H](C#N)C(=N)C2(C#N)C#N)C(F)=C1 | 12 | I |
| CCN1C=C(\C=C2/OC3=CC(O)=CC(C)=C3C2=O)C2=C1C=CC=C2 | 12 | I |
| CC1=CC=C2OC(=CC(=O)C2=C1)C(=O)NC1=CC(F)=CC=C1C | 12 | I |
| COC1=CC(=CC=C1O)C1=CC(=O)C2=C(O1)C=C(C)C(Cl)=C2 | 12 | I |
| C[C@H]1N(CCN2C=CC=C12)C(=O)NC1=CC=CC=C1Br | 12 | I |
| CSC1=CC=CC(NC(=O)N2CC3=CC=CN3C3=C2C=CC=C3)=C1 | 12 | I |
| CN1C(=CC2=C1C=CO2)C(=O)NC1=CC=C(Cl)C=C1Cl | 12 | I |
| ClC1=CC=C(Cl)C(=C1)C(=O)C1=CC2=C(NC(=O)O2)C=C1 | 12 | I |
| CN1C=C(Br)C=C1C(=O)NC1=CC=C(C)C2=C1C=CC=N2 | 12 | I |
| CC[C@]1(C)OC(=O)N2CCC3=C(C)NC4=C3C(=CC=C4C)[C@]12C | 12 | I |
| CC(C)(C)NC(=O)N1CCN2C=CC=C2[C@@H]1C1=CC=CC=C1F | 12 | I |
| CC1=CC=C(C=C1)C(=O)NC1=CC2=CC(Cl)=CC=C2OC1=O | 12 | I |
| Cl[C@H](C1=CC=C(Br)O1)C1=CC2=C(NC(=O)O2)C=C1 | 12 | I |
| Cl[C@@H](C1=CC=C(Br)O1)C1=CC2=C(NC(=O)O2)C=C1 | 12 | I |
| CC1=CS\C(N1)=N/S(=O)(=O)C1=CC=C(Br)S1 | 12 | I |
| BrC1=CN=C2N=C(NC2=C1)C1=CC2=CC=CC=C2O1 | 12 | I |
| C[C@@H]1C=C(C)[C@@H]2[C@H](C)[C@@]1(CO)CO[C@@H]2C1=CC2=C(OCO2)C=C1 | 12 | I |
| CC1=NN(C2=C1CN[C@H](C1=CC=CN21)C1=CC=CC=C1)C1=CC=CC=C1 | 12 | I |
| CN(C(=O)C1=C(Br)C(C)=NN1)C1=CC(C)=CC(C)=C1 | 12 | I |
| CN(C)C1=CC=C(C=C1)[C@H]1NC2=CC=C(C)C=C2C2=CC(C)=NN12 | 12 | I |
| CN(C)C1=CC=C(C=C1)[C@@H]1NC2=C(C=CC=C2C)C2=CC(C)=NN12 | 12 | I |
| FC1=CC=C(C=C1)N1N=CC2=C1NC(=O)C[C@H]2C1=CC=C(Cl)C=C1 | 12 | I |
| CC1=CN\C(C=C1)=N/S(=O)(=O)C1=CC(Cl)=CC(Cl)=C1 | 12 | I |
| OC1=CC=CC(=C1)C1=NC(=NO1)C1=CC=C(Br)S1 | 12 | I |
| OC1=CC=C(C=C1Cl)C1=NC=CN1C1=CC=CC2=CN=CC=C12 | 12 | I |
| O=C(NC1=CC2=CC=CC=C2C=C1)C1=CC(=O)C2=CC=CC=C2O1 | 12 | I |
| FC1=CC=C(NC(=O)C2=CC(=O)C3=CC(Cl)=CC=C3O2)C=C1 | 12 | I |
| CC1=C\C(NC=C1Br)=N\S(=O)(=O)C1=CC=C(F)C=C1 | 12 | I |
| FC1=CC=C(C=C1)[C@H]1C[C@@H](N=C2NC=NN12)C1=CC=C(Cl)C=C1 | 12 | I |
| ClC1=CC(NC2=NN=C(Br)S2)=C(C=C1)C#N | 12 | I |
| CC1=CS\C(N1)=N/S(=O)(=O)C1=C(C)SC2=C1C=CC=C2 | 12 | I |
| CC[C@@H]1N(C(=O)[C@@H](C)NC1=O)C1=CC(Cl)=C(Cl)C=C1Cl | 12 | I |
| ClC1=NC(N[C@@H]2COC3=CC=CC=C23)=C(Br)C=N1 | 12 | I |
| CCN1\C(NC2=CC(Br)=CC=C12)=N\C(=O)[C@@H]1C[C@@H]1C | 12 | I |
| CCC1=C(SN=N1)C1=NC2=CC(Br)=C(F)C=C2N1 | 12 | I |
| CC1=CC=C(NC2=NC(Br)=CN3C=CN=C23)C(F)=C1 | 12 | I |
| C[C@H]1CC[C@@H](NC2=NC(Br)=CN3C=CN=C23)[C@H](C)C1 | 12 | I |
| COC1=CC=C2NC(=NC2=N1)C1=CC(C)=C(Br)S1 | 12 | I |
| C[C@@H]1CC[C@@H](NC2=NN3C=CC=C(Br)C3=N2)[C@H](C)C1 | 12 | I |
| C[C@H]1CC[C@H](C[C@@H]1C)NC1=NN2C=C(Br)C=CC2=N1 | 12 | I |
| CC1(C)CCC[C@@H]1NC1=NN2C=C(Br)C=CC2=N1 | 12 | I |
| O=C(NC[C@H]1COC2=CC=CC=C2C1)N1C[C@H]2CC=CC[C@H]2C1 | 12 | I |
| OC1=NC2=C(N=CN2C2=CC=CC=C2Cl)[C@H](C1)C1=CC=CC=C1 | 12 | I |
| COC1=C(Cl)C=C2C(=O)C(Cl)(Cl)C(=O)NC2=C1Cl | 12 | I |
| CSC1=CC=C(C=C1)[C@@H]1CC(=O)NC2=C1C=C1OCOC1=C2 | 12 | I |
| CC1=C(C2=C(S1)N=CN1C(=S)NN=C21)C1=CC=C(F)C=C1 | 12 | I |
| FC1=CC=C(C=C1)[C@@H]1C=C(C2CC2)[C@H](C#N)C(=N)C1(C#N)C#N | 12 | I |
| CC1=NC(C)=C(S1)C(=O)NC1=NC2=C(C)C=C(C)C=C2S1 | 12 | I |
| CN1N=C(C)C(Cl)=C1C(=O)NC1=CC(Cl)=CC(Cl)=C1 | 12 | I |
| O\C(=C1/[C@H](OC(=O)C1=O)C1=CC=CC=C1F)C1=CC=CC=C1 | 12 | I |
| FC1=CC=C(NC(=O)[C@H]2CC3=CC=C(Cl)C=C3C(=O)O2)C=C1 | 12 | I |
| CC1=C2OC(=O)C3=C(CCCC3)C2=CC2=C1OC(C)(C)C[C@@H]2O | 12 | I |
| C[C@@H]1OC2=CC=CC=C2O[C@H]1C(=O)NC1=CC=C(F)C(F)=C1 | 12 | I |
| CC1=CC(=O)OC2=C1C=C(Cl)C([O-])=C2C[NH+]1CCCC1 | 12 | I |
| C[C@@H]1CCCC[C@@]11NC(=O)N(C[C@@H]2CC2(Cl)Cl)C1=O | 12 | I |
| CC1=C(C(=O)NC2=CC(Cl)=C(Cl)C=C2Cl)C(C)=NO1 | 12 | I |
| CC1=C(OC2=C1C(=O)CCC2)C(=O)NC1=CC=C(C)C=C1F | 12 | I |
| CCCN1C[C@@H]2CCC[C@@]3(C1)C(=O)NC1(CCCCC1)N=C23 | 12 | I |
| FC1=CC(F)=C(C=C1)[C@@H]1CC(=O)NC2=C1C=C1OCOC1=C2 | 12 | I |
| ClC1=CC=CC=C1[C@@H]1C[C@H](NC2=NC=NN12)C1=CC=CC=C1 | 12 | I |
| CC1=CC=C(O1)[C@@H](Br)C1=CC=C2NC(=O)OC2=C1 | 12 | I |
| ClC1=CC2=C(OCC(=O)N2)C=C1C(=O)[C@H]1C[C@H]2CC[C@@H]1C2 | 12 | I |
| COC1=CC(OC)=C(Cl)C2=C1NC1=C(CCCC1)C2=O | 12 | I |
| [O-]C(=O)C1=COC(=[NH+]1)C1=CC=C(I)C=C1 | 12 | I |
| C1CC2=CC=CC=C2C2=NC3=[NH+]C=NN3[C@@H]([C@H]12)C1=CC=CS1 | 12 | I |
| ClC1=C(N[C@H]2CCS(=O)(=O)C2)C2=C(SC=N2)C=C1 | 12 | I |
| CN(C(=O)C1=CC2=C(CCCC2)NC1=O)C1=CC=C(C)C=C1 | 12 | I |
| CC1=C(C)C2=C(S1)N=CN=C2N[C@@H]1COC2=C1C=CC=C2 | 12 | I |
| C[C@H]1CCC[C@]2(C1)N(CC1=CC=C(Cl)S1)C(=O)NC2=O | 12 | I |
| CC1=CC2=C3N(C4=NC(=S)NN=C4C3=C1C)C(C)(C)C=C2C | 12 | I |
| FC1=CC=C(C=C1)[C@H]1C[C@H]1C(=O)N1CC(=O)NC2=CC=CC=C12 | 12 | I |
| CN1C2=CC=C(F)C=C2N=C1C1=CC=C(C)C(NC(C)=O)=C1 | 12 | I |
| CC1=CC2=CC=C3C([N-]S(=O)(=O)C4=C3C=CC=C4)=C2[NH+]=C1 | 12 | I |
| CC1=CC=CC=C1[C@@H]1C[C@H](NC2=NC=NN12)C1=CC=C(F)C=C1 | 12 | I |
| CC(C)C1=C(I)C(=O)NC(=N1)N(C)C | 12 | I |
| C[C@]1(CN(CCN1)C1=CC=C2OC(F)(F)OC2=C1)C1CC1 | 12 | I |
| BrC1=CC=CC=C1N1CC(=O)NC2(CCCC2)C1=O | 12 | I |
| C[C@@H]1O[C@H](C)[C@H]([C@H](O)C2=CC3=C(OCCO3)C=C2Cl)[C@@H]1C | 12 | I |
| CC1=CC(C)=NC(NC(=O)C2=CC(Br)=CC=C2C)=N1 | 12 | I |
| BrC1=CN2C(C=C1)=NN=C2[C@@H]1C[C@@H]2CCCC[C@@H]2N1 | 12 | I |
| C[C@H]1CN[C@H](CN1C1=C(Cl)C=CC2=NSN=C12)C1CC1 | 12 | I |
| CC1=CC(=O)OC2=C1C=CC(NC(=O)[C@H]1C[C@H]3C[C@@H]1C=C3)=C2 | 12 | I |
| C[C@@H]1C[C@@H]1C1=CC=C(O1)C1=NC(C)=C(Br)C(=O)N1 | 12 | I |
| OC1=CC(=NN1C1=NC=C(Br)C=C1)C(F)(F)F | 12 | I |
| C[C@@H](NC(=O)C1=CC2=C(O1)C(C)=CC(Br)=C2)C#N | 12 | I |
| CC(C)C1=C(Br)C(=O)NC(=N1)C1=CC=C(F)C=N1 | 12 | I |
| CC1=C(N=C(S1)C1=NNC=N1)C1=CC=CC(Br)=C1 | 12 | I |
| C[C@@H]1CC[C@H](NC2=NN3C=CC=C(Br)C3=N2)[C@@H]1C | 12 | I |
| BrC1=CN=C(N[C@@H]2CCC[C@H]3OCC[C@H]23)N=C1 | 12 | I |
| [O-]C1=C(C(=O)OC(=[NH+]1)C#CC1=CC=CC=C1F)C1=CC=CC=C1 | 12 | I |
| FC1=C(Cl)C=C(\C=C\C2=NC3=CN=CC=C3C(=O)N2)C=C1 | 12 | I |
| CN1\C(SC2=CC=CC=C12)=N\C(=O)C1=CC=CNC1=S | 12 | I |
| CC1=NC2=C([C@H](S1)C(F)(F)F)C(O)=NN2C1CCCC1 | 12 | I |
| C[C@@]12CC[C@H]3[C@H]([C@H]1CCC2=O)C(=O)C[C@H]1C[C@@H](O)CC[C@@]31C | 12 | I |
| C[C@]12CC[C@H]3[C@@H](C=CC4=C[C@@H](O)CC[C@]34C)[C@H]1CC[C@@]21CCC(=O)O1 | 12 | I |
| C[C@]12CC[C@H]3[C@@H](CC(=O)[C@H]4C[C@@H](O)CC[C@]34C)[C@H]1CCC2=O | 12 | I |
| C[C@]1(O)CC[C@@H]2[C@H]3CC(=O)C4=CC(=O)CC[C@]4(C)[C@H]3CC[C@]12C | 12 | I |
| OC1=C(CC=C(Cl)Cl)C(=O)C(=O)C2=CC=CC=C12 | 12 | I |
| CC1=C(Br)C=C2N=C(NC2=N1)C(F)(F)F | 12 | I |
| FC(F)(F)C1=C(C=CC(Cl)=C1)N1C=NNC1=S | 12 | I |
| CN[C@H]1C[C@H](C)S(=O)(=O)C2=CC=C(C=C12)C(C)(C)C | 12 | I |
| CN1C=C(N[C@H]2CCSC3=C2C=C(Cl)C=C3)C=N1 | 12 | I |
| O[C@H](C1=CSC=C1)C1=CC2=C(OCCO2)C=C1Cl | 12 | I |
| CNS(=O)(=O)C1=C(C)C=C(Br)C(C)=C1 | 12 | I |
| O[C@@H]1[C@@H](CCC2=CC=CC=C12)N1C=CC(=N1)C(F)(F)F | 12 | I |
| IC1=CC=CC(=C1)N1C=CC(=O)NC1=S | 12 | I |
| CN1C=CN=C1C(C)(O)C1=CC=C(Br)C=C1 | 12 | I |
| BrC1=CC=C(N[C@@]2(CCCOC2)C#N)C=C1 | 12 | I |
| O[C@@H]1CCC[C@H]1N1C=C(I)C=N1 | 12 | I |
| C[C@@]12CC[C@H]3[C@H](CCC4=CC(=O)CC[C@@]34C)[C@H]1CCC(=O)N2 | 12 | I |
| C[C@@]12CC[C@H]3[C@H](CCC4=CC(=O)CC[C@]34C)[C@H]1CCC(=O)N2 | 12 | I |
| C[C@@H]1CCC[C@H](C1)C1=NC(C)=C(Br)C(=O)N1 | 12 | I |
| BrC1=CC2=C(N=C1)N(CC1CC1)C(=S)N2 | 12 | I |
| C[C@H]1CNC2(CCCCC2)CN1CC1=C(C)N=CS1 | 12 | I |
| ClCC1=NC(=NN1)C1=CC=C(Br)S1 | 12 | I |
| [O-]C(=O)C1=C[NH+]=CC(=C1)C1=CSC=C1Br | 12 | I |
| CNC1=CC(=NC=N1)C1=CC(Br)=CC=C1F | 12 | I |
| CN1[C@H]2COC[C@H]2CNC2=CC(Br)=CC=C12 | 12 | I |
| BrC1=CC2=C(O[C@H](CN2)C2=COC=C2)C=C1 | 12 | I |
| CC1=CC(Br)=CN=C1N[C@H]1C[C@H]2CC[C@@H]1O2 | 12 | I |
| FC1=CC2=NNC(I)=C2C=C1C#N | 12 | I |
| C[C@@H]1CN[C@@H](S1)C1=CC2=C(OCCO2)C(Br)=C1 | 12 | I |
| C[C@@H]1CN[C@@H](C[S@@]1=O)C1=CC2=CC(Br)=CC=C2O1 | 12 | I |
| BrC1=CC(=CC2=C1OCCCO2)[C@@H]1NCCCS1 | 12 | I |
| C[C@H]1CCS[C@@H](N1)C1=CC2=C(OCCO2)C(Cl)=C1 | 12 | I |
| C[C@H]1CCS(=O)(=O)C[C@H](N1)C12CC3CC(CC(C3)C1)C2 | 12 | I |
| CC1=C(Br)C=CC(=N1)N1C=CC2=C1CCC[C@H]2O | 12 | I |
| O[C@@H](C1=CC=CC=C1F)[C@@]1(CCOC2=C1C=CC=C2)C#N | 12 | I |
| FC1=C(C=C(Br)C=C1)N1C(=S)NC2=CN=CC=C12 | 12 | I |
| CC1=CC=NC2=C1NC(=S)N2C1=C(F)C=C(F)C=C1F | 12 | I |
| BrC1=CC=C(C=C1)[C@@H]1CNCC[C@@]11CCS(=O)(=O)C1 | 12 | I |
| O[C@@H]([C@H]1CCO[C@]2(CCSC2)C1)C1=NC=C(F)C=C1 | 12 | I |
| C[C@H]1CC[C@H](O)[C@H](CN2C[C@@H]3CCCCN3C[C@@H]2C)C1 | 12 | I |
| CC(C)(C)[C@H]1CC[C@H](O)[C@@H](C1)N1CCN2CCC[C@@H]2C1 | 12 | I |
| CNC1=C2CCCCC2=NC(=N1)[C@@H]1CSCCS1 | 12 | I |
| CN[C@H]1CC(C)(C)CC2=C1SC(=N2)[C@]1(C)CCCO1 | 12 | I |
| ClC1=NC(I)=NC2=C1C=CN2 | 12 | I |
| CC1=CC(\C=C2\SC(=S)NC2=O)=C(C)N1C1CC1 | 12 | I |
| O[C@@H]1C[C@@H](OC2=C1C=C(Br)C=C2)C1=CN=CS1 | 12 | I |
| CC1=NC(=NN1)C1=CC=C(I)C=C1 | 12 | I |
| C[C@]12C3=CC(=O)C=C1C=C(O)C1=CC(=O)C=C(C=C3)[C@]21C | 12 | I |
| OC1\C=C/[C@H](Br)C2(OCCO2)[C@@H](Br)\C=C/1 | 12 | I |
| C[C@]12CC(=O)[C@@H]3[C@H](CC[C@H]4C=C(O)C=C[C@]34C)[C@@H]1CCC2=O | 12 | I |
| O=C1NC(=S)C2=CC=CC=C2\C1=C\C1=CC=C2OCOC2=C1 | 12 | I |
| CC1=CC=C(C=C1)N1C(=O)S\C(=C/C2=CC=C(O)C=C2)C1=O | 12 | I |
| CC1=CC(C)=NC(NC(=O)C2=C(F)C(F)=C(F)C(F)=C2F)=N1 | 12 | I |
| CC1=NC(C)=C(S1)C(=O)NC1=NC2=CC=C(C)C=C2S1 | 12 | I |
| O=C1COC2=C(N1)C=C(C=C2)C1=CSC(=N1)C1=CSC=C1 | 12 | I |
| OC1=CC=C(C=C1)N1C(S)=NC2=C(C3=C(CCC3)S2)C1=O | 12 | I |
| CC1=CC=C(N2C(=S)NN=C2C2=CC=NC=C2)C(Cl)=C1 | 12 | I |
| COC1=CC=C(C=C1Br)C1=NC(C)=CC(=O)N1 | 12 | I |
| C[C@@H]1C[C@H](CC(C)(C)C1)O[C@@H]1CC2(CC[C@@H]1O)OCCO2 | 12 | I |
| C[C@H]1CN([C@H](C)CO1)C1=NC2=C(S1)[C@H](O)CC(C)(C)C2 | 12 | I |
| CC1(C)C[C@@H](O)C2=C(C1)N=C(S2)N1CCOCC1(C)C | 12 | I |
| FC(F)(F)C(F)(F)C1=NC2=NC(Cl)=C(Cl)N=C2N1 | 12 | I |
| FC1=CC=C(F)C(NC(=O)C2=CC(=O)C3=CC=CC=C3O2)=C1 | 12 | I |
| O[C@@H]1CCC[C@H]1C1=NC(=NO1)C1=CC(Br)=CS1 | 12 | I |
| CN1C=C(C=CC1=O)C(=O)NC1=CC=C(Cl)C(Cl)=C1 | 12 | I |
| CN1C=C(C=CC1=O)C(=O)NC1=CC(Cl)=CC(Cl)=C1 | 12 | I |
| CN1C(C)=NC2=CC(=CC=C12)C(=O)NC1=CC=C(F)C(F)=C1F | 12 | I |
| CC1=C(OC=C1)C(=O)NC1=CC=C(Br)C(C)=N1 | 12 | I |
| C[C@H]1CCN(CC2=NC3=C(C4=C(CCC4)S3)C(=O)N2)[C@H](C)C1 | 12 | I |
| CC1=C\C(C=CN1)=N\S(=O)(=O)C1=CC=C(F)C=C1Cl | 12 | I |
| CC1=CC=C(\C=C2\SC(=O)N(C2=O)C2=CC=C(O)C=C2)C=C1 | 12 | I |
| C[C@H]1N(C2=CC=C(Cl)C(Cl)=C2)C(=O)C(C)(C)NC1=O | 12 | I |
| CC[C@@H]1N(C(=O)[C@@H](C)NC1=O)C1=CC=C(Cl)C(Cl)=C1 | 12 | I |
| CC[C@H]1N(C(=O)[C@H](C)NC1=O)C1=CC=C(Cl)C(Cl)=C1 | 12 | I |
| C[C@H]1N(C2=CC(Cl)=CC(Cl)=C2)C(=O)C2(CCCC2)NC1=O | 12 | I |
| CC[C@]1(C)NC(=O)[C@H](C)N(C1=O)C1=CC=CC(Cl)=C1Cl | 12 | I |
| C[C@@H]1N(C2=CC=C(F)C(Cl)=C2)C(=O)C2(CCCC2)NC1=O | 12 | I |
| ClC1=CC(Cl)=C(N=C1)C1=NC(=O)C(Br)=CN1 | 12 | I |
| C[C@@H]1NCCN2C1=NN=C2C1=CC(Br)=CC=C1C | 12 | I |
| C[C@H]1C[C@@H]1C1=NOC(=N1)C1=CC=C(Br)C=C1O | 12 | I |
| CC1=C(SN=N1)C(=O)NC1=CC=C(Br)C(F)=C1 | 12 | I |
| ClC1=C(N2CCCN[C@H](C2)C2CC2)C2=NSN=C2C=C1 | 12 | I |
| [O-]C(=O)[C@H]1COCC[NH+]1C1CCCCCCCCCCC1 | 12 | I |
| CNC1=C2C=CC=CC2=C(N=N1)C1=C(Br)C=CO1 | 12 | I |
| CC[C@]1(C)NC(=O)[C@@H](C)N(C1=O)C1=C(C)C=CC=C1Cl | 12 | I |
| COC1=CC=C(C=C1O)C1=NC=C(Br)C(Cl)=N1 | 12 | I |
| CC1=C(Br)C=C\C(N1)=N\S(=O)(=O)C(C)(C)C | 12 | I |
| BrC1=CSC(NC2=CC3=C(OCCO3)C=C2)=N1 | 12 | I |
| ClC1=CC=C(C=C1)[C@H]1[C@@H](CC(=O)NC1=O)[C@@H]1CCCOC1 | 12 | I |
| CN1N=CC(=C1C)C1=NC2=CC(Br)=C(F)C=C2N1 | 12 | I |
| C[C@@H]1CN(C(=O)NC1=O)C1=CC=C(Br)C(C)=C1 | 12 | I |
| CC1(C)OC(C)(C)[C@H](CN2CCO[C@H]3CCCC[C@H]23)[C@@H]1O | 12 | I |
| CC1(C)OC(C)(C)[C@@H](CN2CCO[C@H]3CCCC[C@H]23)[C@H]1O | 12 | I |
| C[C@@H](N1C(=S)NC2=C1N(C)N=C2C)C1=CC=C(C)C(F)=C1 | 12 | I |
| BrC1=CN=C(N[C@H]2CCC[C@@H]3OCC[C@H]23)N=C1 | 12 | I |
| CC[C@H]1NC(=O)[C@@H](C)N(C1=O)C1=CC(Cl)=CC(Cl)=C1 | 12 | I |
| CC1=C(C)C=C(S1)C(=O)NC1=CC=C2OC(=O)C=CC2=C1 | 12 | I |
| CN1N=CC2=C1N=C(O)C[C@@H]2C1=CC=C(Cl)C=C1Cl | 12 | I |
| OC1=NC2=C(SC=C2)[C@H](SC1)C1=CC2=C(OCO2)C=C1 | 12 | I |
| CN1N=CC2=C1N=C(O)CS[C@@H]2C1=CC=C(Cl)C=C1Cl | 12 | I |
| BrC1=CC=CC(NC(=O)C2=COC(=O)C=C2)=C1 | 12 | I |
| CN1C=C(Br)C(=N1)C(=O)NC1=CC=C(F)C=C1 | 12 | I |
| CC1=CC(NC2=N[C@@H]3CS(=O)(=O)C[C@H]3S2)=CC(C)=C1 | 12 | I |
| BrC1=CC=C(O1)C(=O)NC1=C(C=CS1)C#N | 12 | I |
| CC1=CC=C(NC(=O)[C@H]2[C@@H]3C[C@@H]4OC(=O)[C@@H]2[C@@H]4C3)C=C1C | 12 | I |
| CC1=CC(NC(=O)C2=CC=CO2)=C2C=C(F)C=C(F)C2=N1 | 12 | I |
| CC1=CC(CN2C(=O)N[C@@]3(CCSC3)C2=O)=CC(C)=C1 | 12 | I |
| CC1=NOC(NC(=O)C2=CSC(Br)=C2)=C1 | 12 | I |
| C[NH+]1CCC(CC1)N1C(=S)N=C2SC3=C(CCC3)C2=C1[O-] | 12 | I |
| O=C1C[C@H](C2=CC=CS2)C2=C(N1)C=C1OCCOC1=C2 | 12 | I |
| CC1=CC(C)=C(C(=O)N1)S(=O)(=O)C1=CC(F)=CC=C1C | 12 | I |
| O=C(N[C@H]1CS(=O)(=O)C=C1)C12CC3CC(CC(C3)C1)C2 | 12 | I |
| BrC1=CC=C(C=C1)C1=NC(=S)NC(=O)[C@@H]1C#N | 12 | I |
| FC1=CC=CC(N2C(=S)NN=C2C2=CC=NC=C2)=C1F | 12 | I |
| CC1=NSC(NC(=O)C2=CC(Br)=CN=C2)=C1 | 12 | I |
| BrC1=CN=C(SC2=NC=CC(=O)N2)C=C1 | 12 | I |
| O[C@H]1[C@@H](COC2=CC=CC=C12)N1C=CC(=N1)C(F)(F)F | 12 | I |
| O[C@H]1[C@H](COC2=CC=CC=C12)N1C=CC(=N1)C(F)(F)F | 12 | I |
| C[C@@H]1NCCN2C1=NN=C2C1=C(F)C(F)=C(F)C(F)=C1F | 12 | I |
| CN[C@H]1COCCC2=C1C1=C(OCCCO1)C=C2Cl | 12 | I |
| O[C@@H]([C@H]1COC2=CC=CC=C2O1)C1=CC=C2COCC2=C1 | 12 | I |
| CN1C=C(NC(=O)C2=CC=C(Br)C=C2C)C=N1 | 12 | I |
| O[C@H](C1CCOCC1)C1=CC2=C(OCCO2)C(Cl)=C1 | 12 | I |
| C[C@H]1CCCN([C@@H]1C)S(=O)(=O)C1=CC=C2CCNC2=C1 | 12 | I |
| O[C@@H]1CN(CC2=CC3=NSN=C3C=C2)CC2=CC=CC=C12 | 12 | I |
| C[C@H]1NCCN2C1=NN=C2C1=C(Br)C=CS1 | 12 | I |
| FC(F)(F)C1=CC(Cl)=C(C=C1)N1CC(=O)NCC1=O | 12 | I |
| CC1(C)NC(=O)CCN(C2=CC=C(Br)C=C2)C1=O | 12 | I |
| FC1=CC(F)=C(F)C=C1N1CC(=O)N[C@H](C2CC2)C1=O | 12 | I |
| C[C@H]1NC(=O)CN(C1=O)C1=CC(=CC=C1F)C(F)(F)F | 12 | I |
| CNC1=NC(=NC(C)=C1Br)[C@]1(C)CCCO1 | 12 | I |
| BrC1=C(N=C(NC1=O)C1=CN=CS1)C1CC1 | 12 | I |
| C([C@@H]1CN2CCC[C@@H]2CO1)N1CCCNC2(CCCC2)C1 | 12 | I |
| CN1N=C(C)C(CN2C=CC3=C2CC(C)(C)C[C@H]3O)=C1C | 12 | I |
| CN1[C@H]2COC[C@H]2C(=O)NC2=CC(Br)=CC=C12 | 12 | I |
| CN1N=C(C)C2=C1N(CC1=CC=C(F)C=C1F)C(=S)N2 | 12 | I |
| CN([C@H]1CCSC1)C1=CC2=C(C=C1Cl)C(=O)C(=O)N2 | 12 | I |
| ClC1=CC=C(C=C1)[C@@H]1C(=O)NC(=O)C[C@]11CCCOC1 | 12 | I |
| CC1=NNC=C1CN1CCO[C@@]2(CCC3=C2C=CC=C3)C1 | 12 | I |
| C[C@H]1OCC[C@H]1[C@H](O)C1=CC2=C(OCCO2)C=C1Cl | 12 | I |
| C[C@@H]1OCC[C@H]1[C@H](O)C1=CC2=C(OCCO2)C=C1Cl | 12 | I |
| CC(C)[C@H]1C[C@](O)(CCO1)[C@@H]1CCOC2(CCOCC2)C1 | 12 | I |
| FC1=NC(F)=C(F)C(NC2CCS(=O)(=O)CC2)=C1F | 12 | I |
| FC1=C(Cl)C=C2NCCN([C@H]3CCS(=O)(=O)C3)C2=C1 | 12 | I |
| C[C@@H]1N(CC2=CC(C)=C(F)C(C)=C2)C(=O)C(C)(C)NC1=O | 12 | I |
| ClCC1=NC(=O)C2=C(N1)SC=C2C1=NC=CS1 | 12 | I |
| ClC1=CC=C(CN2CC3(CCNCC3)OC2=O)S1 | 12 | I |
| ClC1=C(CN2CC[C@H]3CC[C@@H](C2)N3)N2C=CSC2=N1 | 12 | I |
| CC1=C(C)C(C#N)=C(NC(=O)C2=CC=NC(F)=C2F)S1 | 12 | I |
| OC[C@H]1CCC2=NN=C([C@H]3CCCC4=CC=CC=C34)N2C1 | 12 | I |
| C[C@]12CC[C@@H]3[C@H](CC[C@]45O[C@H]4C(=O)[C@@H](C[C@]35C)C#N)[C@@H]1CC[C@@H]2O | 12 | I |
| C[C@@]12CC[C@H]3[C@H](CC[C@]45O[C@H]4C(=O)[C@@H](C[C@]35C)C#N)[C@@H]1CC[C@@H]2O | 12 | I |
| FC1=CC=CC=C1CN1C(=O)N[C@@]2(CCCC3=CC=CC=C23)C1=O | 12 | I |
| C[C@@H]1N(O)[C@@](C)(C2=CC=C(C)C=C2)[N+]([O-])=C1C1=CC=CS1 | 12 | I |
| BrC1=CC=CC(CN2C(=O)NC3(CCCC3)C2=O)=C1 | 12 | I |
| OC1=CC=C(C=C1)N1C(=O)S\C(=C\C2=CC=C(F)C=C2)C1=O | 12 | I |
| CC1=CC(\C=C2/SC(=O)NC2=O)=C(C)N1C1=CC=C(C)C=C1C | 12 | I |
| [O-]C(=O)C1=C2C=CC(Br)=CC2=NC(=C1)C1=CC=[NH+]C=C1 | 12 | I |
| CC(C)CN1C(=S)NC2=C(C3=C(COC(C)(C)C3)S2)C1=O | 12 | I |
| CC1=CC(C)=NC(NC(=O)C2=CC(F)=CC=C2Br)=N1 | 12 | I |
| CC[C@]12CCC[NH+]3CCC4=C([C@H]13)N(C1=CC=CC=C41)C(=C2)C([O-])=O | 12 | I |
| CC1=C(C)C2=C(S1)N=C(C)N=C2S[C@H]1CCCCNC1=O | 12 | I |
| CN1C=C(N[C@@H]2CCCOC3=C2C=CC(Br)=C3)C=N1 | 12 | I |
| CC1=C\C(NC=C1)=N\S(=O)(=O)C1=CC=CC(Br)=C1 | 12 | I |
| CN1C=CC(N[C@H]2CCCOC3=C2C=CC(Br)=C3)=N1 | 12 | I |
| [O-]C1=N\C(=C/[NH+]2CCCC2)N=C2[C@@H]1C1(CCCC1)CC1=CC=CC=C21 | 12 | I |
| CC1=CC(C)=NC(NC(=O)C2=CC=C(Br)C(F)=C2)=N1 | 12 | I |
| CN1N=C(C)C(NC(=O)C2=CC=C(Br)C(F)=C2)=C1C | 12 | I |
| CC[NH+]1CCC[C@@H](C1)N1C(=S)N=C2SC(C)=C(C)C2=C1[O-] | 12 | I |
| OCC1=C(N=CC(Br)=C1)N1CCO[C@H]2CCCC[C@H]12 | 12 | I |
| CS(=O)(=O)C1=CC=C(C=C1)\N=C1\NC2(CS1)CCCCC2 | 12 | I |
| CC1=CC2=C(C(=O)O\C2=C/C2=CC=C(Br)O2)C(=S)N1 | 12 | I |
| O[C@H](C1=CC(F)=CN=C1)C1=CC2=C(OCCCO2)C(Cl)=C1 | 12 | I |
| O[C@H](C1=COC=C1)C1=CC2=C(OCCCO2)C(Br)=C1 | 12 | I |
| O[C@H](C1=CC=CO1)C1=CC2=C(OCCCO2)C(Br)=C1 | 12 | I |
| OC1=C(CN2CCCN3C=NC=C3C2)C=C(Cl)C=C1Cl | 12 | I |
| C[C@@H]1C[C@H](CN1C(=O)C1=C(C)C=C(C)NC1=O)C1=CC=C(F)C=C1 | 12 | I |
| C[C@H]1C[C@H](CN1C(=O)C1=CC=C2NC=NC2=C1)C1=CC=C(F)C=C1 | 12 | I |
| CN1N=C(C)C=C1NC(=O)C1=CC(Cl)=CC=C1Br | 12 | I |
| CC1=NC(C)=C(O1)C(=O)NC1=CC(Br)=CC=C1Cl | 12 | I |
| C[C@H]1NC(=O)C2(CCCCC2)N(CC2=CC=C(Cl)S2)C1=O | 12 | I |
| C[C@@H]1N(CC2=CC=C(Cl)S2)C(=O)C2(CCCC2)NC1=O | 12 | I |
| C[C@H]1CC(=O)NCC(=O)N1CC1=C(Br)C=CS1 | 12 | I |
| C[C@H]1N(C2=CC=C(Br)C=C2)C(=O)C2(CCCC2)NC1=O | 12 | I |
| C[C@H]1CN(CC2=CC(Br)=CS2)C(=O)NC1=O | 12 | I |
| ClC1=CC(CN2C(=O)CNC(=O)C22CCCC2)=C(Cl)C=C1 | 12 | I |
| BrC1=CN2C(C=C1)=NN=C2C1=C2CCC[C@@H]2SC1=N | 12 | I |
| C[C@H]1NC(=O)[C@H](C)N(C1=O)C1=C(C)C=C(Br)C=C1C | 12 | I |
| O[C@H](C1=CC2=C(OCO2)C(Br)=C1)C1(CCCC1)C#N | 12 | I |
| C[C@@H](N1C(=S)NC2=CC(Cl)=C(F)C=C12)C1=C(C)ON=C1C | 12 | I |
| CN1N=C(C)C2=C1N(C(=S)N2)C1=CC=C(C)C(Br)=C1 | 12 | I |
| O=C1NC2(CCCC2)C(=O)N1C[C@H]1CSC2=CC=CC=C12 | 12 | I |
| C[C@H]1CC(C)(C)CC[C@H]1NC(=O)N(C)C1CCS(=O)CC1 | 12 | I |
| ClC1=C(Br)C=C(NC(=O)C2=COC(=O)C=C2)C=C1 | 12 | I |
| CC1=C(Br)C(=O)N(N1)C1=CC=C(C=N1)C(F)(F)F | 12 | I |
| OC1=NC2=C(N=CN2C2=CC=C(Cl)C=C2)[C@@H](C1)C1=CSC=C1 | 12 | I |
| C[C@@H]1CCC[C@H](N1)C1=CN=CN1C1=CC=C(Br)C=N1 | 12 | I |
| CC1=NC(C)=C(NC(=O)C2=CC(Br)=CN=C2)C(C)=C1 | 12 | I |
| C[C@H]1CCC[C@@H](C)N1C1=C(I)C(=O)NC=N1 | 12 | I |
| CNC1=NC(=NC=C1Br)C1=CC2=CC=C(C)N=C2C=C1 | 12 | I |
| O=C1N(CC2=CC=CO2)C(=S)NC2=CC3=CC=CC=C3C=C12 | 12 | I |
| ClC1=CC=C2OC(=O)C(=CC2=C1)C(=O)NC1=CC=CC=C1 | 12 | I |
| CC1=CC=C(C=C1C)N1C(=O)CS[C@@]11C(=O)NC2=CC=CC=C12 | 12 | I |
| ClC1=CC=C(C=C1)[C@H]1CC(=O)NC2=C1C=C1OCOC1=C2 | 12 | I |
| CC1=NC(C)=C(O1)C(=O)NC1=CC=C(Br)C(C)=C1 | 12 | I |
| FC1=CC=C(CN2C=NC3=C(NC4=CC=C(F)C=C34)C2=O)C=C1 | 12 | I |
| CS(=O)(=O)NC1=CC=C2SC3=CC=CC=C3C(=O)C2=C1 | 12 | I |
| C[C@@H]1CCCN(C1)C1=NC(=O)\C(S1)=C\C1=CC=C(O)C=C1 | 12 | I |
| CC1=CC2=C(S1)C1=C(N2CC2=CC=C(F)C=C2)C(=O)NN=C1 | 12 | I |
| C[C@H]1OC2=CC=CC=C2O[C@@H]1C(=O)NC1=CC=C(F)C(F)=C1 | 12 | I |
| O=C1C[C@H](C2=C(N1)SC1=C2CCCC1)C1=CC2=C(OCO2)C=C1 | 12 | I |
| OC1=NC2=CC=CC=C2C(=O)N1C1=CC=CC(Br)=C1 | 12 | I |
| CC1=CC(C)=C\C(N1)=N\S(=O)(=O)C1=CC=C(Cl)C=C1 | 12 | I |
| CN1C(=O)SC2=CC(NC(=O)C3=C(C)C=CS3)=CC=C12 | 12 | I |
| CC1=C(C)N=C2C=C(C=CC2=N1)C(=O)NC1=CC=C(F)C=C1F | 12 | I |
| CC(C)N1C[C@H]2CCC[C@]3(C1)C(=O)NC1(CCCCC1)N=C23 | 12 | I |
| C[C@@H]1CCC2=C(C1)SC1=C2C2=NNC(=S)N2C(=N1)C1CC1 | 12 | I |
| CC(C)(C)C(=O)C1=CC2=C(NC(=O)CO2)C=C1Br | 12 | I |
| FC1=CC=CC=C1\C=C\C(=O)N1CC(=O)NC2=C1C=CC=C2 | 12 | I |
| CC1=CC=C2C(=O)C3=C(OC2=C1C)C(=O)N[C@H]3C1=CC=C(F)C=C1 | 12 | I |
| CC1=CC(C)=C2C(=O)C3=C(OC2=C1)C(=O)N[C@@H]3C1=CC=C(F)C=C1 | 12 | I |
| CC1=CC=C(C=C1)C1=NN=C2[C@@H]1[C@@H](NC2=O)C1=CC=C(F)C=C1 | 12 | I |
| CC1=CC=C(NC2=C(Br)C=NC(Cl)=N2)N=C1 | 12 | I |
| OCC1(CCCCC1)N1C(=O)CC2(CCCCCC2)C1=O | 12 | I |
| C1S\C(NC11CCCCC1)=N/C1=CC2=C(OCCO2)C=C1 | 12 | I |
| CN[C@@H]1CC(C)(C)CC2=C1SC(=N2)N1C[C@H](C)O[C@H](C)C1 | 12 | I |
| CC1=CC=C(C=C1)[C@H]1C[C@H](N2N=CN=C2N1)C1=CC=CC=C1F | 12 | I |
| ClC1=CC(C2=CSC(=N2)C2=NNC=N2)=C(Cl)C=C1 | 12 | I |
| FC1=CC=C(C=C1)[C@H]1C[C@H](N2N=CN=C2N1)C1=CC=CC=C1 | 12 | I |
| CC1=CC2=C(OCCCO2)C=C1C(=O)NC1=CC=C(F)C(F)=C1 | 12 | I |
| ClC1=CC=C(S1)S(=O)(=O)\N=C1/NC=C(Cl)C=C1 | 12 | I |
| C[C@H]1CN(C[C@@H](C)S1)C1=C(C=[NH+]C2=CC=CC=C12)C([O-])=O | 12 | I |
| OC1=CC=C(C=C1)C1=NOC(=N1)[C@H]1CSC2=CC=CC=C12 | 12 | I |
| CC(C)C1=C(N2C=C(Br)C=C(C)C2=[NH+]1)C([O-])=O | 12 | I |
| CN1N=C(C)C(=C1C)C1=NC2=CC(Br)=CC=C2N1 | 12 | I |
| N=C1S[C@H](C=C1)C1=NC(=NO1)[C@H]1CCC2=CC=CC=C2C1 | 12 | I |
| CC[C@]1(C)NC(=O)CCN(C2=CC=C3SC=CC3=C2)C1=O | 12 | I |
| S=C1NN=C(N1C1=CC=C2SC=CC2=C1)C1=CC=CC=N1 | 12 | I |
| [O-]C(=O)[C@@H]1COCC[NH+]1C1CCCCCCCCCCC1 | 12 | I |
| C[C@@H]1CC[C@@H](C)N(C1)C1=C(C=C(Br)C=[NH+]1)C([O-])=O | 12 | I |
| FC1=CC2=C(OC[C@H](C2)C(=O)NC2=CC=C(Cl)C=N2)C=C1 | 12 | I |
| CC1CCC(CC1)(C#N)[C@@H](O)C1=CC2=C(OCO2)C(Cl)=C1 | 12 | I |
| C[C@@H]1CN(C[C@H]1C)C1=C(C=C(Br)C=[NH+]1)C([O-])=O | 12 | I |
| O[C@@H](C1=CC2=C(OCCO2)C=C1)C1=C(Cl)C=C(Cl)C=N1 | 12 | I |
| C[C@H]1CCC[C@H]1NC1=NC(Br)=CN2C=CN=C12 | 12 | I |
| BrC1=CN2C=CN=C2C(N[C@@H]2C[C@H]3CC[C@@H]2C3)=N1 | 12 | I |
| CC1=C(C=NO1)C(=O)NC1=CC=C(Cl)C(Br)=C1 | 12 | I |
| CNC1=C2C3=C(CCCC3)SC2=NC(=N1)[C@@]1(C)CCCO1 | 12 | I |
| CC1=CSC=C1C1=NOC(=N1)[C@@H]1NCCC2=C1C=CS2 | 12 | I |
| BrC1=CN\C(S1)=N\S(=O)(=O)C1CCCC1 | 12 | I |
| CCC1(CC)[C@H](O)N=C(Br)C(C#N)C1(C#N)C#N | 12 | I |
| [O-][N+]1=CC2=C(N(C3=CC=CC=C3)C(=O)C1)C1=CC=CC=C1N2 | 12 | I |
| CC1=CN(C(=O)N1)S(=O)(=O)C1=CC=C(Br)C=C1 | 12 | I |
| CNC(=O)C1=CN=C2SC=C(N2C1=O)C1=CC=C(F)C=C1 | 12 | I |
| O=C(NC1=C(C#N)C2=C(CCCC2)S1)C1=CSN=N1 | 12 | I |
| O=C(NC1=CC2=C(OCCO2)C=C1)C1=CC2=CC=N[C@H]2C=C1 | 12 | I |
| [O-]C(=O)C1=NN(C=CC1=[OH+])C1=CC=C(Br)C=C1 | 12 | I |
| CC1CCC2(CC1)NC(=O)N(CN1CCC[C@H](C)C1)C2=O | 12 | I |
| C[C@]1(CCS(=O)(=O)C1)NC(=O)N1C=CC2=C1C=CC=C2 | 12 | I |
| FC(F)(F)[C@@H]1CCCN(C1)C(=O)N[C@@H]1CCS(=O)(=O)C1 | 12 | I |
| FC(F)(F)[C@H]1CCCN(C1)C(=O)N[C@H]1CCS(=O)(=O)C1 | 12 | I |
| CN1C=C(NC(=O)[C@@H]2COC3=CC=C(Cl)C=C3C2)C=N1 | 12 | I |
| CC1=NOC(NC(=O)C2=CC=C(Br)C(F)=C2)=N1 | 12 | I |
| O=C1C[C@H](\C=C\C2=CC=CO2)C2=C(CN1)N=C1C=CC=CN21 | 12 | I |
| C[C@H]1N(CCN2C=CC=C12)C(=O)[C@@H]1CC(=O)NC2=C1C=CC=C2 | 12 | I |
| O[C@@H](C1=CC2=C(OCCO2)C(Br)=C1)C1=NC=CN=C1 | 12 | I |
| C[C@]12C[C@H](CC(C)(C)C1)N(C2)S(=O)(=O)C1=CNC=CC1=O | 12 | I |
| O=C1C=CNC=C1S(=O)(=O)N1CCC2(CCCCC2)CC1 | 12 | I |
| CN(C)CC1=C2C(=O)C(=O)N3C2=C(C=C1O)C(C)=CC3(C)C | 12 | I |
| BrC1=CC2=C(CCN2C(=O)C2=NNN=C2)C=C1 | 12 | I |
| CS(=O)(=O)C1=CC=C2C(NC3=CC=CC=C3S2(=O)=O)=C1 | 12 | I |
| CC1=NN(C=C1[C@@H]1N=C([O-])[C@H]2CCCC[NH+]12)C1=CC=CC=C1 | 12 | I |
| FC1=CC=C(C=C1)N1C=C(C=N1)[C@@H]1NC(=O)[C@H]2CCCCN12 | 12 | I |
| C[C@H]1CN(C[C@@H](C)S1)S(=O)(=O)C1=CNC(=O)C(Cl)=C1 | 12 | I |
| CC(=O)NC1=C(Br)C=C(F)C(F)=C1[N+]([O-])=O | 12 | I |
| C[C@H]1NC(=O)C2(CCCCC2)N(C[C@@H]2CCCCO2)C1=O | 12 | I |
| CC1(C)N(C[C@H]2CCOC2)C(=O)C2(CCCCC2)NC1=O | 12 | I |
| C[C@H](N1[C@H](C)C(=O)NC2(CCCC2)C1=O)C1=CC=C(C)O1 | 12 | I |
| C[C@@H]1N(C[C@H]2CCCOC2)C(=O)C2(CCCCC2)NC1=O | 12 | I |
| CC(C)[C@H]1N([C@H]2CCCOC2)C(=O)C2(CCCC2)NC1=O | 12 | I |
| C[C@H]1O[C@H](C)[C@H]([C@H](O)C2=CC=C3N(C)C(=O)OC3=C2)[C@@H]1C | 12 | I |
| N=C1S[C@H]2CCCCC2=C1C1=NC(=NO1)[C@H]1CCOC1 | 12 | I |
| C1CC2=C(C=CS2)[C@H](N1)C1=NC(=NO1)C1CCOCC1 | 12 | I |
| CC1=CC(Br)=CC(C)=C1N1C(=O)NC(=O)C1=O | 12 | I |
| BrC1=CC(=CC2=C1OCCCO2)[C@@H]1COC(=O)N1 | 12 | I |
| C[C@@H]1CC[C@H](C)N(C1)S(=O)(=O)C1=C(C)NC(=O)S1 | 12 | I |
| CN1N=C(C)C=C1C1=NC2=C(N1)N=CC(Br)=C2 | 12 | I |
| FC1=CC=CC=C1NC(=O)[C@@H]1CSC2=NC=CC(=O)N2C1 | 12 | I |
| CC1=CC(NC(=O)[C@H]2CSC3=NC=CC(=O)N3C2)=CC(C)=C1 | 12 | I |
| CC1=CC(C)=C(C)C(=C1C)S(=O)(=O)N1CCNC[C@@H]1C#N | 12 | I |
| O[C@@H](C1=CC2=C(OCO2)C(Br)=C1)[C@]1(CCOC1)C#N | 12 | I |
| C[C@H]1CN(C[C@@H]1C)S(=O)(=O)C1=CNC(=O)C(Cl)=C1 | 12 | I |
| O[C@@H]([C@@H]1CCO[C@]2(CCOC2)C1)C1=CC2=C(OCO2)C=C1 | 12 | I |
| CN1CCC[C@H](C1)NC1=NC(Br)=CN2C=CN=C12 | 12 | I |
| CCC1=NN(C(O)=C1Br)[C@@]1(C)CCS(=O)(=O)C1 | 12 | I |
| C[C@H]1CC(=O)NC(C)(C)C(=O)N1CC1=NC(C)=C(C)S1 | 12 | I |
| CC1=CC(=NO1)C(=O)NC1=NC=C(Br)C=C1C | 12 | I |
| O=C1NC2=CC=CC=C2[C@]11COC2=C1C=C1OCCOC1=C2 | 12 | I |
| FC1=C(C=CC(=C1)[C@H]1NC(=O)[C@H]2CCCCN12)N1CCOCC1 | 12 | I |
| CN1N=CC2=C1N=C(O)CS[C@@H]2C1=CC=C2N=CC=CC2=C1 | 12 | I |
| FC1=C(F)C=C(C=C1)[C@@H]1CC(=O)NC2=C1C=C1OCOC1=C2 | 12 | I |
| FC(F)(F)C(=O)NC1=C2C3=C(CCCC3)SC2=NC=N1 | 12 | I |
| C[C@H]1CC=C2[C@@H](C1)[C@H]([C@@H](C#N)C(=N)C2(C#N)C#N)C1=CC=CC=C1F | 12 | I |
| CC1(C)CC2=C(CO1)C=C1C(SC3=C1N=CNC3=S)=N2 | 12 | I |
| O=C(NC1=CC=C2OCCOC2=C1)[C@H]1CC2=CC=CC=C2S1 | 12 | I |
| CC1=CC(Br)=CC=C1NC(=O)C1=COC(=O)C=C1 | 12 | I |
| CN1N=CC(C(=O)NC2=CC(Br)=CC=C2C)=C1C | 12 | I |
| CC1=CC=C(C=C1)[C@@H]1CC(=O)NC2=C1C=C1OCCOC1=C2 | 12 | I |
| OC1=CC=C(\C=C2/SC(=O)N(C2=O)C2=CC=CC=C2F)C=C1 | 12 | I |
| IC1=CC=C(NC(=O)C2=NOC=C2)C=C1 | 12 | I |
| FC1=CC=C(C=C1C#N)N1C(=S)NN=C1C1=CC=CS1 | 12 | I |
| CN[C@@H]1CC(C)(C)CC2=C1SC(=N2)N1CCN(C)CC1 | 12 | I |
| C[C@@H]1CN(CCCO1)C1=NC2=C(S1)[C@H](O)CC(C)(C)C2 | 12 | I |
| CC1=C[C@@H](N=N1)C(=O)NC1=CC=C(Br)C(C)=C1 | 12 | I |
| CC1=C2C=C3CCCC3=CC2=NC(=N1)N1[C@@H]2CC[C@H]1CNCC2 | 12 | I |
| O[C@@H](C1=CC=CO1)C1=CC2=C(OCO2)C(Br)=C1 | 12 | I |
| BrC1=CC2=C(OCCO2)C=C1N[C@H]1CCCOC1 | 12 | I |
| FC1=CC(NC(=O)C2=CC3=C(OCCO3)C=C2)=CC(F)=C1F | 12 | I |
| CCN1C=C(\C=C2/C(=O)NC3=CC=C(Br)C=C23)C=N1 | 12 | I |
| C[C@]1(CCCN1)C1=NN=C2N1C=C(C=C2Cl)C(F)(F)F | 12 | I |
| FC(F)CN1C(=O)C2(CCCCC2)NC(=O)C11CCCC1 | 12 | I |
| CC[C@]1(C)NC(=O)[C@H](C)N(C2=CC(Cl)=CC(Cl)=C2)C1=O | 12 | I |
| C[C@H]1N(C(=O)C(C)(C)NC1=O)C1=CC=C(Br)C=C1C | 12 | I |
| C[C@H]1N(C(=O)C(C)(C)NC1=O)C1=CC(Br)=CC=C1C | 12 | I |
| CC1(C)NC(=O)CN(C2=CC=C(F)C(=C2)C(F)(F)F)C1=O | 12 | I |
| C[C@@H]1N(C2=CC=C(Cl)C(F)=C2)C(=O)[C@@](C)(NC1=O)C1CC1 | 12 | I |
| FC1=CC(=CC(F)=C1F)N1CC(=O)NC2(CCCC2)C1=O | 12 | I |
| C[C@@H]1N(C2CCC(C)(C)CC2)C(=O)[C@H](NC1=O)C(C)(C)C | 12 | I |
| CC(C)[C@H]1N([C@@H]2CC[C@H](C)[C@@H](C)C2)C(=O)C(C)(C)NC1=O | 12 | I |
| C[C@@H]1CN(CC2=CC3=C(OCCCO3)C(Cl)=C2)[C@H](C)CN1 | 12 | I |
| CC1=CC(=NO1)C(=O)NC1=CC=C(Br)C(F)=C1 | 12 | I |
| S=C1NN=C(N1C1=CC2=C(SC=C2)C=C1)C1=CC=NC=C1 | 12 | I |
| CCN1N=C(C)C=C1C1=NC2=CC=C(Br)C=C2N1 | 12 | I |
| BrC1=C(N[C@H]2CCOC3=CC=CC=C23)N=CN=C1 | 12 | I |
| BrC1=C(OC2=CC=CC3=C2NCCC3)N=CN=C1 | 12 | I |
| CN(C)[C@@H]1CCCN(C1)C1=NC2=C(S1)[C@H](O)CC(C)(C)C2 | 12 | I |
| FC1=CC=C2OC[C@H](CC2=C1)C(=O)NC1=CC2=C(COC2)C=C1 | 12 | I |
| FC1=CC=CC2=C1O[C@H](C2)C(=O)NC1=CC=C2COCC2=C1 | 12 | I |
| CN1N=C(C)C2=C1N(C(=S)N2)C1=CC=CC(=C1)C(F)(F)F | 12 | I |
| CCN1N=C(C)C2=C1N(C(=S)N2)C1=CC(F)=CC(F)=C1F | 12 | I |
| O[C@@H]([C@@H]1CCO[C@]2(CCOC2)C1)C1=C(Cl)C=C(Cl)C=N1 | 12 | I |
| OC1=C(Cl)C=C(C=C1)C1=NC(=NO1)[C@@H]1CSCCS1 | 12 | I |
| C[C@H]1NCCN2C1=NN=C2C1=CC(C)=C(Br)S1 | 12 | I |
| CC1CCC(CC1)NC1=NN2C=C(Br)C=CC2=N1 | 12 | I |
| OC1=CC=CC(CN2C(=O)C(=O)C3=C(Cl)C=CC(F)=C23)=C1 | 12 | I |
| C[C@@]1(CCCO1)[C@H](Br)C1=CC=C2NC(=O)OC2=C1 | 12 | I |
| C[C@@H]1N(CC2=C(C)C=CS2)C(=O)C2(CCCCC2)NC1=O | 12 | I |
| FC1=C(Br)C=C2C(=O)N=C(NC2=C1)C1=COC=C1 | 12 | I |
| CCN1N=C(C)C2=C1N(C(=S)N2)C1=CC=C(C)C(Cl)=C1 | 12 | I |
| CC1=C(C(=NO1)C1=CC=C(Cl)C=C1)C1=NC(=S)NC=C1 | 12 | I |
| CC1=NN(C(C)=C1Br)C1=CC=C(C=C1)C([O-])=O | 12 | I |
| CC1=NN(C(C)=C1)C1=CC=C(Br)C=C1C([O-])=O | 12 | I |
| CC1=CC(=C(C)O1)C1=CSC2=C1C([O-])=NC(CCl)=N2 | 12 | I |
| CC1=C(C=NC(=N1)C1=CC=C(Cl)C(Cl)=C1)C([O-])=O | 12 | I |
| [O-]C1=NC(N=C2SC=CN12)(C(F)(F)F)C(F)(F)F | 12 | I |
| [O-]C1=C2OC3=CC=CC=C3C2=NC(=S)N1C1=CC=CC=C1 | 12 | I |
| [O-]C(=O)C1=CC(F)=C(C=C1)N1C=C(Br)C=N1 | 12 | I |
| [O-]C1=NC2=C(O1)C=C(C=C2)[C@@H](Br)[C@H]1CCCO1 | 12 | I |
| CN1N=C(C=C1C([O-])=O)C1=CC=C(Br)S1 | 12 | I |
| CC1=C(C)C2=C([O-])N(C[C@H]3CCCCO3)C(=S)N=C2S1 | 12 | I |
| CC1=C(C)C2=C([O-])N(C[C@@H]3CCCOC3)C(=S)N=C2S1 | 12 | I |
| C[C@H](N1N=NN=C1[S-])C1=CC=C(Br)C=C1 | 12 | I |
| COC1=CC=C(Br)C=C1N1C=NN=C1[S-] | 12 | I |
| [O-]C1=NN(CC2=C(F)C=C(Br)C=C2)C(=O)C=C1 | 12 | I |
| CC1=C(Br)C(=NN1C1=CC=C(F)C=C1)C([O-])=O | 12 | I |
| CC1=CC(=CC=C1Br)N1C=NC(=C1)C([O-])=O | 12 | I |
| [O-]C1=NC2=C(O1)C=C(C=C2)[C@H](Br)[C@@H]1CCCCO1 | 12 | I |
| [O-]C(=O)C1=COC(=N1)C1=CC=C(I)C=C1 | 12 | I |
| CC(=O)N1C(=S)N=C2C(Cl)=CC(Cl)=CC2=C1[O-] | 12 | I |
| FC1=CC(F)=C(F)C(=C1)N1C([S-])=NN=C1C1=CC=NC=C1 | 12 | I |
| [O-]C(=O)C1=C(Cl)C=C(C=C1)N1C=C(Br)C=N1 | 12 | I |
| FC(F)(F)C(F)(F)C1=NC2=C([N-]1)C=CC1=NSN=C21 | 12 | I |
| CC(C)C1=CC=C(C=C1)[C@H]1SCC([O-])=NC2=NN=C[C@H]12 | 12 | I |
| CC1=CC=C(C=C1)C1=NN=C2[C@@H]1[C@@H](N=C2[O-])C1=CC=CS1 | 12 | I |
| [O-]C(=O)C1=CC=C(C=C1)N1C=C(I)C=N1 | 12 | I |
| CC1=C(C)C(=O)N(CC2=CC=C(Br)C=C2)N=C1[O-] | 12 | I |
| [O-]C1=CC(=NC2=C(Br)C=NN12)C(F)(F)F | 12 | I |
| [O-]C1=NC=NC2=CC(=CC=C12)C1=C(C=CC=N1)C(F)(F)F | 12 | I |
| [O-]C1=C(CN2CCCN3C=NC=C3C2)C=C(Cl)C=C1Cl | 12 | I |
| FC(F)(F)C1=NC(=N[N-]1)C1=CC=C(Br)O1 | 12 | I |
| CC1=CN(CC2=CC=C(Cl)C(Cl)=C2)C([O-])=NC1=O | 12 | I |
| CC1=C[N-]C=C\C1=N/S(=O)(=O)C1=CC=C(Cl)C=C1C | 12 | I |
| COC1=CC=C(C=C1Br)N1C(C)=NN=C1[S-] | 12 | I |
| [O-]C1=NC(=NC2=C1SC=C2)C1=CC(Br)=CN=C1 | 12 | I |
| CCC1=C(I)C([O-])=NC(=N1)C1=CC=CO1 | 12 | I |
| [O-]C(=O)[C@H]1C[C@H]1C1=NC2=C(O1)C=CC(Br)=C2 | 12 | I |
| [S-]C1=NN=NN1C1=C(Br)C=CC(Cl)=C1 | 12 | I |
| CC1=NN(C(C)=C1[O-])C1=C(Cl)C=C(C=N1)C(F)(F)F | 12 | I |
| CC(C)(C)C1=C(Br)C([O-])=NC(=N1)[C@H]1CCOC1 | 12 | I |
| CN(C)C1=CC=C(C=C1)C1=NC=C(Br)C([O-])=N1 | 12 | I |
| C[C@@H]1OCC[C@H]1[C@H](Br)C1=CC=C2N=C([O-])OC2=C1 | 12 | I |
| [O-]C(=O)C1(CCOCC1)[C@@H]1CCOC2(CCCCC2)C1 | 12 | I |
| CC1(C)CCC(CC1)([C@@H]1CCO[C@@]2(CCOC2)C1)C([O-])=O | 12 | I |
| CC1(C)[C@H]([C@H]1C1=NC2=CC(Br)=CC=C2O1)C([O-])=O | 12 | I |
| C[C@@]1(CCCO1)[C@H](Br)C1=CC=C2N=C([O-])OC2=C1 | 12 | I |
| CC1=CC(Br)=CN=C1N(C([O-])=O)C(C)(C)C | 12 | I |
| FC(F)(F)C1=CC2=C([N-]C(Cl)=NS2(=O)=O)C(Cl)=C1 | 12 | I |
| [O-]C(=O)C1=CC(=O)C2=C(O1)C=CC(I)=C2 | 12 | I |
| CC(C)N1N=CC2=C(C=C(Br)C=C12)C([O-])=O | 12 | I |
| [O-]\C(=N/C1=C(C=CC(Cl)=C1)C#N)C1=CC=NC(F)=C1F | 12 | I |
| CCC1=CC=C(C=C1)[C@H]1CC(=O)NC2=C1C=C1OCCOC1=C2 | 12 | I |
| CC(=O)NC1=CC=C(C=C1)C1=CN2C(C=CC3=CC=CC=C23)=N1 | 12 | I |
| FC1=CC([C@H]2CC(=O)NC3=C2C=C2OCOC2=C3)=C(F)C=C1 | 12 | I |
| CC1=CC=C(C=C1)[C@H]1[C@@H]2CSCC=C2[C@@H](C#N)C(=N)C1(C#N)C#N | 12 | I |
| FC1=CC=CC=C1[C@H]1[C@@H]2CCCC=C2[C@H](C#N)C(=N)C1(C#N)C#N | 12 | I |
| CN1N=C(C)C2=C1SC(=C2)C(=O)NC1=CC=C(C)C(F)=C1 | 12 | I |
| C1C[C@H](NC2=CC=C3OCCCOC3=C2)C2=CC=CC=C2O1 | 12 | I |
| C1OC2=CC=C(C=C2O1)\N=C1/NC[C@@H](S1)C1=CC=CC=C1 | 12 | I |
| ClC1=C(SC=C1)C(=O)NC1=CC2=C(COC2=O)C=C1 | 12 | I |
| BrC1=CC=C(C=C1)C1=N\C(=C\C2=CC=CO2)C(=O)N1 | 12 | I |
| CCCN1C[C@H]2CCC[C@]3(C1)C(=O)NC1(CCCCC1)N=C23 | 12 | I |
| ClC1=CC=C(C=C1)[C@H]1C[C@H](NC2=NC=NN12)C1=CC=CC=C1 | 12 | I |
| FC1=CC=C(C=C1)N1C(=S)NC2=C(OC3=CC=CC=C23)C1=O | 12 | I |
| CC1=CC=C(NC(=O)C2=CC3=C(OCCO3)C=C2Cl)C=C1 | 12 | I |
| O=C(C1=CSC2=CC=CC=C12)C1=CC=C2NC(=O)OC2=C1 | 12 | I |
| FC1=CC=CC=C1[C@@H]1C[C@@H](NC2=NC=NN12)C1=CC=CC=C1 | 12 | I |
| [O-]C(=O)C1=COC(=[NH+]1)C1=C(Cl)C=C(Br)C=C1 | 12 | I |
| N=C1S[C@H]2CCCCC2=C1C1=NN=CN1C1CCCCC1 | 12 | I |
| CC[C@H]1CN(CCO1)C1=NC2=C(S1)[C@H](O)CC(C)(C)C2 | 12 | I |
| O=C(NC1=C2OCCOC2=CC=C1)C1=CSC2=C1CCCC2 | 12 | I |
| CC1=CC=C2N(CC3=CC=C4OCOC4=C3)C(=S)NC2=C1 | 12 | I |
| C[C@@H]1CCC[NH+](CC2=C([O-])C=CC3=C2OC(=C(C)C)C3=O)C1 | 12 | I |
| CC1=C(CN2CCC[C@]3(CC2)OC2=CC=CC=C2C=C3)N=CN1 | 12 | I |
| BrC1=NC(NC(=O)C2=COC3=CC=CC=C23)=CC=C1 | 12 | I |
| C[C@H]1CN(C[C@H](C)S1)C1=CC(C([O-])=O)=C2C=CC=CC2=[NH+]1 | 12 | I |
| BrC1=CC2=C(OCCO2)C=C1N[C@@H]1CCCOC1 | 12 | I |
| ClC1=CC=CC(C2=CC(=O)N3N=CC(C4CC4)=C3N2)=C1Cl | 12 | I |
| CC1=C(NC=N1)C1=NC=CN1C1C2=CC=CC=C2C2=C1C=CC=C2 | 12 | I |
| FC1=CC=C(C=C1)C1=NNC=C1C=C1C(=O)C2=CC=CC=C2C1=O | 12 | I |
| CSC1=N[P@](=S)(NC2=C1N1CCCC1=N2)SC | 12 | I |
| O=C1N=C(N\C1=C/C1=CSC2=CC=CC=C12)N1CCCCC1 | 12 | I |
| ClC1=C(Br)C=C(NC(=O)C2=CSN=N2)C=C1 | 12 | I |
| CC1=CC=C(CN2C=C3C(O)=NN=C3C3=C2C=CC(F)=C3)C=C1 | 12 | I |
| CC[C@@H]1N(C2=CC(Cl)=CC(Cl)=C2)C(=O)C(C)(C)NC1=O | 12 | I |
| CC[C@@]1(C)NC(=O)[C@H](C)N(C2=CC=C(C)C(Cl)=C2)C1=O | 12 | I |
| [S-]C1=NN=C(N1C1=CC=CC=C1)[NH+]1CCC[C@@H]2CCC[C@H]12 | 12 | I |
| ClC1=C(\N=C2/NC[C@H](S2)C2CC2)C2=NSN=C2C=C1 | 12 | I |
| O[C@H]1CCCC2=C1C=CN2C1=C(Cl)C=CC2=NSN=C12 | 12 | I |
| ClC1=CC=C(S1)S(=O)(=O)\N=C1/NC=CC(Cl)=C1 | 12 | I |
| C[C@]1(COC(=O)N1)C1=CC2=CC(Br)=CC=C2O1 | 12 | I |
| CC(C)(C)[C@@H]1NC(=O)CN(C1=O)C1=C(F)C=C(F)C=C1F | 12 | I |
| C[C@H]1CCC[C@H](C)N(CC2=NC3=C(C(C)=C(C)S3)C(=O)N2)C1 | 12 | I |
| CCC1=C(OC2=CC=CC=C12)[C@@H]1CS(=O)(=O)CC[C@H](C)N1 | 12 | I |
| CN1C(Cl)=C(Cl)C=C1C(=O)NC1=CC=C2COCC2=C1 | 12 | I |
| C[C@@H]1CCCC[C@@H]1N(C)C1=CC2=C(C=C1Cl)C(=O)C(=O)N2 | 12 | I |
| CC1(CCCCC1)NC1=NC(Br)=CN2C=CN=C12 | 12 | I |
| CC1=C(Br)SC(=C1)C(=O)NC1=C(C)C(C)=NO1 | 12 | I |
| CC1=NN(C(O)=C1Br)C1=CC=C(Br)C=N1 | 12 | I |
| OC1=CC=C2C(O\C(=C\C3=CC=C(C=C3)N3CCCC3)C2=O)=C1 | 12 | I |
| CN1C(Cl)=C(Cl)C=C1C(=O)NC1=C(C)N=C(C)C=C1C | 12 | I |
| CN1N=C(C(=O)NC2C3CC4CC(C3)CC2C4)C(Br)=C1C | 12 | I |
| ClC1=CC=C(C=C1Cl)N1C(=O)CS[C@]11C(=O)NC2=CC=CC=C12 | 12 | I |
| CC1=CC=C(C=C1Br)[C@@H]1CC(=O)NC2=C1C=C1OCOC1=C2 | 12 | I |
| CC(C)CC1=C2COC(C)(C)CC2=C2C(SC3=C2NC=NC3=S)=N1 | 12 | I |
| ClC1=CC=CC=C1[C@@H]1[C@@H]2CCCC=C2[C@@H](C#N)C(=N)C1(C#N)C#N | 12 | I |
| CC1=CC=C(C=C1)N1NC(=O)\C(=C/C2=CC=C(Br)C=C2)C1=O | 12 | I |
| C[C@H]1CCC[C@@]2(C1)C[C@H](O)C1=C(O2)C(C)=C2OC(=O)C3=C(CCC3)C2=C1 | 12 | I |
| ClC1=CC=C(C=C1)C(=O)NC1=CC2=C(OCCO2)C=C1Br | 12 | I |
| CN1N=C(C(=O)NC2=CC=C(F)C=C2)C2=C1C1=CC(C)=CC=C1SC2 | 12 | I |
| CC1=NC2=CC(NC(=O)C3=CC=C(Br)C=C3F)=CC=C2O1 | 12 | I |
| BrC1=CC=CC(=C1)C1=CC=C(O1)\C=C1/SC(=O)NC1=O | 12 | I |
| OC1=NC(=NC(=N1)C(Cl)(Cl)Cl)C(Cl)(Cl)Cl | 12 | I |
| FC1=CC=C(Br)C=C1C(=O)N1CC(=O)NC2=CC=CC=C12 | 12 | I |
| BrC1=CC=C(C=C1)C1=CC=C(O1)\C=C1/SC(=O)NC1=O | 12 | I |
| C[C@H]1CC=C2[C@@H](C1)[C@H]([C@@H](C#N)C(=N)C2(C#N)C#N)C1=CC=CC=C1Cl | 12 | I |
| IC1=CC=CC(=C1)C(=O)C1=CC=C2NC(=O)OC2=C1 | 12 | I |
| CC1=CN\C(C=C1)=N/S(=O)(=O)C1=C(F)C=C(F)C=C1Br | 12 | I |
| CSC1=CC=C(C=C1)[C@@H]1NC(=O)C2=C1C(=O)C1=CC=C(C)C(C)=C1O2 | 12 | I |
| CC1=NC(C)=C(S1)C(=O)NC1=NC2=C(S1)C=CC(Cl)=C2Cl | 12 | I |
| COC1=C(O)C(Br)=CC(\C=C2/OC3=CC=C(C)C=C3C2=O)=C1 | 12 | I |
| C[C@]12C[C@H]3OC(C)(C)O[C@H]4C[C@@H](O)C[C@@H]5CC[C@H]([C@@H]1CCC2=O)[C@@H]3[C@@]45C | 12 | I |
| C[C@@]12C[C@H]3OC(C)(C)O[C@H]4C[C@@H](O)C[C@@H]5CC[C@H]([C@H]1CCC2=O)[C@@H]3[C@@]45C | 12 | I |
| FC1=CC=CC2=C(NC(=O)C3=CC=C(Br)O3)C=CN=C12 | 12 | I |
| CC1=C(C)S\C(N1)=N\S(=O)(=O)C1=C(Br)C=CS1 | 12 | I |
| FC1(F)OC2=C(O1)C=C(NC(=O)C1=C(Br)C=CS1)C=C2 | 12 | I |
| BrC1=CC(Br)=C(NC2=C(N=CC=N2)C#N)C=C1 | 12 | I |
| ClC1=CC=C(N2C(=O)CS[C@]22C(=O)NC3=CC=CC=C23)C(Cl)=C1 | 12 | I |
| CC1=NC(C)=C(S1)C(=O)NC1=NC2=CC=C(Br)C=C2S1 | 12 | I |
| FC1=CN\C(C=C1)=N/S(=O)(=O)C1=C(Cl)C=CC(Br)=C1 | 12 | I |
| FC1=C([C@H]2CC(=O)NC3=C2C=NN3C2CCCC2)C(Cl)=CC=C1 | 12 | I |
| CC1=CC(NC(=O)C2=CC=C(Br)C=C2)=CC2=C1OCCO2 | 12 | I |
| BrC1=CC=C2N=C(NC2=C1)C1=CC2=C(OCCO2)C=C1 | 12 | I |
| CC[C@H]1N(C(=O)[C@H](C)NC1=O)C1=CC(Cl)=C(Cl)C=C1Cl | 12 | I |
| CC(C)(C)[C@@H]1NC(=O)CN(C1=O)C1=C(F)C=C(F)C=C1Br | 12 | I |
| C[C@@H]1N(C(=O)[C@H](NC1=O)C(C)(C)C)C1=CC(Br)=CC=C1C | 12 | I |
| CC(C)C1=C(I)C(Cl)=NC(=N1)C1=NC=CN1 | 12 | I |
| CC1=[NH+]C2=CC(=CC=C2N1C1=CC=C(Br)C(F)=C1)C([O-])=O | 12 | I |
| CC1=CN2C(C=C1)=NC=C2C(=O)NC1=CC=C(F)C=C1Br | 12 | I |
| CNC1=NC(=NC(C)=C1F)C1=CC(Br)=C(Br)O1 | 12 | I |
| CCN1\C(NC2=CC(Br)=CC=C12)=N\C(=O)C1CC=CC1 | 12 | I |
| CCC1=NN(C)C2=C1NC(=S)N2C1=CC=C(Br)C=C1C | 12 | I |
| CC1=C\C(NC=C1Br)=N\S(=O)(=O)C1=CC(F)=CC=C1C | 12 | I |
| C[C@H]1C[C@H]2CNC[C@@H]2N1S(=O)(=O)C1=CC(C)=C(Br)S1 | 12 | I |
| BrC1=CC=C(C=C1)C1=CN=C(N=C1)N1CCCNCC1 | 12 | I |
| BrC1=CSC(=C1)C1=NOC(=N1)C1=C2CCC[C@@H]2SC1=N | 12 | I |
| O[C@H]1C[C@H](OC2=CC=C(Br)C=C12)C1=CC2=C(OCO2)C=C1 | 12 | I |
| O[C@@H]1C[C@H](OC2=CC=C(Br)C=C12)C1=CC2=C(OCO2)C=C1 | 12 | I |
| O=C1C[C@@H](C(=O)N1C1=CC2=CC=CC=C2C=C1)C1=CNC2=C1C=CC=C2 | 12 | I |
| CC1=C(Br)C=C\C(N1)=N\S(=O)(=O)C1=CC=C(F)C=C1F | 12 | I |
| CC(C)(C)N1N=CC2=C1N=C(O)C[C@@H]2C1=CC=CC(Br)=C1 | 12 | I |
| CN1N=C(C)C2=C1SC(=C2)C(=O)NC1=CC=CC=C1F | 12 | I |
| CN(C)C1=CC=C(\C=C2/C(=O)NC(=O)C3=CC=CC=C23)C=C1 | 12 | I |
| CN1C=CC(=N1)C(=O)NC1=CC=C(Br)C=C1 | 12 | I |
| CC1=CN=C(C=N1)C(=O)NC1=CC(Cl)=CC(Cl)=C1 | 12 | I |
| CN1N=C(C)C2=C1SC(=C2)C(=O)NC1=CC=C(F)C=C1 | 12 | I |
| C[NH+](C)CC1=C([O-])C(Cl)=CC2=C1OC(=O)C(C)=C2C | 12 | I |
| C[C@@H](NC(=O)N1C=CN=C1)C1=CC=C(Cl)C=C1Cl | 12 | I |
| C[C@@H]1OC2=CC=C(C=C2NC1=O)C(=O)C1=CC=C(C)S1 | 12 | I |
| C[C@H]1C[C@H](C(=O)N2C[C@H](C)OC[C@@H]2C)C2=CC=CC=C2N1 | 12 | I |
| CN1C(=NC2=C(F)C=CC=C12)[C@@H]1CNC2=CC=CC=C2O1 | 12 | I |
| CO[C@@H]1OC(=O)C2=C1[C@@]1(C)CCCC(C)(C)[C@H]1C[C@@H]2O | 12 | I |
| O[C@@H]1CCCC2=C1C=CN2C1=CC2=C(OC(F)(F)O2)C=C1 | 12 | I |
| ClC1=CC(C(=O)NC2=NN=CS2)=C(Cl)S1 | 12 | I |
| BrC1=CC2=C(C=C1)N(CC2)C(=O)C1=CNC(=O)C=C1 | 12 | I |
| FC1=CC=C2NC(=S)N(C2=C1)C1=CC2=C(OCO2)C=C1 | 12 | I |
| COC1=CC=C2C=CC=CC2=C1\C=C1/SC(=O)NC1=O | 12 | I |
| CC1=CC(=CC=C1F)N1C(=S)NN=C1C1=CC=NC=C1 | 12 | I |
| CC1=C2[C@H](NC(=O)CS[C@H]2C2=CC(F)=CC=C2F)N=N1 | 12 | I |
| CC1=CC(C)=C(C=C1C)C(=O)NC1=CN=C2CCCCN12 | 12 | I |
| CC1=C\C(NC=C1)=N\S(=O)(=O)C1=CC(F)=CC=C1C | 12 | I |
| OC1=CC(Cl)=C(C=C1)N1C(=O)CC2(CCCCC2)C1=O | 12 | I |
| BrC1=CC=C(O1)C1=NC(=CC(=O)N1)C1CC1 | 12 | I |
| FC1=C(C=CC=C1Cl)S(=O)(=O)N=C1C=CNC=C1 | 12 | I |
| CCC1=C([C@@H]2N=C([O-])[C@@H]3CCCC[NH+]23)C2=CC=CC=C2O1 | 12 | I |
| BrC1=CC2=C(NC(=N2)C2=CSN=N2)C=C1 | 12 | I |
| C[C@@H]1CC(=O)N[C@H](C)C(=O)N1CC1=CC=C(Cl)S1 | 12 | I |
| FC1=CC(N2CCC(=O)NC3(CCCC3)C2=O)=C(F)C=C1 | 12 | I |
| FC1=CC=C(C=C1)N1CCC(=O)NC2(CCCCC2)C1=O | 12 | I |
| CC(C)[C@H]1N([C@H]2CCCC[C@H]2C)C(=O)C(C)(C)NC1=O | 12 | I |
| FC1=CC(=CC(F)=C1)N1CCC(=O)NC2(CCCC2)C1=O | 12 | I |
| FC1=CC(=CC(F)=C1)N1CC(=O)NC2(CCCC2)C1=O | 12 | I |
| C[C@@H]1NC(=O)[C@@H](C)N(C1=O)C1=CC(Cl)=CC(Cl)=C1 | 12 | I |
| CC[C@H]1N(C(=O)[C@H](C)NC1=O)C1=CC=C(C)C(Cl)=C1 | 12 | I |
| FC1=C(Cl)C=C(C=C1)N1CC(=O)N[C@@H](C2CC2)C1=O | 12 | I |
| FC1=C(Cl)C=CC(=C1)N1CC(=O)N[C@@H](C2CC2)C1=O | 12 | I |
| CC[C@@H]1CO[C@H](C)CN1C1=NC2=C(CCC[C@@H]2O)S1 | 12 | I |
| C[C@@H]1SC(=N)C(=C1)C1=NC(=NO1)[C@@H]1CC2=C1C=CC=C2 | 12 | I |
| OC1(CN2C(=O)CC3(CCCCCC3)C2=O)CCCC1 | 12 | I |
| C[C@@H]1CC[C@H](C1)C1=NOC(=N1)C1=C2CCC[C@@H]2SC1=N | 12 | I |
| C[C@@H]1CC[C@H](C1)C1=NOC(=N1)C1=C2CCC[C@@H]2SC1=N | 12 | I |
| OCC1=CN(N=N1)C1=C(Br)C=C(Cl)C=C1 | 12 | I |
| CC1=CC=NC=C1NC(=O)[C@@H]1COC2=CC=C(F)C=C2C1 | 12 | I |
| BrC1=CN=C(NC1=O)C1=CC2=C(OCC2)C=C1 | 12 | I |
| O[C@@H](C1=CC2=C(OCCO2)C=C1)C1(CCCCCC1)C#N | 12 | I |
| C[C@H]1CN(C[C@H]1C)S(=O)(=O)C1=CC=C2NCCC2=C1 | 12 | I |
| CCN1[C@H]2COC[C@@H]2C(=O)NC2=CC=C(Br)C=C12 | 12 | I |
| O[C@@H]([C@H]1CCCC2=C1N=CC=C2)C1=CC=C2OCOC2=C1 | 12 | I |
| CC(C)[C@@H]1N([C@@H]2CCCCC2(C)C)C(=O)[C@@H](C)NC1=O | 12 | I |
| C[C@@H]1[C@H](CC[NH+]1[C@@H]1CCOC2(CCCCC2)C1)C([O-])=O | 12 | I |
| O[C@H]1C[C@@H](OC2=CC(F)=CC=C12)C1=CC2=C(OCO2)C=C1 | 12 | I |
| O=C1NC2=C(C=CC=C2)C11OCC2(C[C@@H]3C[C@H]2C=C3)CO1 | 12 | I |
| CC(C)=C1C(=O)NN(C1=O)C1=CC=C(Cl)C(Cl)=C1 | 12 | I |
| CNC(=O)C1=CC2=C(OC1=O)C(Br)=CC(C)=C2 | 12 | I |
| FC1=CC=C(C=C1F)C(=O)N1CC(=O)NC2=CC=CC=C12 | 12 | I |
| CCCN1C(=O)NC2=CC=C(Br)C=C2C1=O | 12 | I |
| C[NH+](C)CC1=CC2=C(OC(=O)C3=C2CCCC3)C(C)=C1[O-] | 12 | I |
| CC1=CC=C(CC2=C([O-])[NH+]=C3SCCCN3C2=O)C=C1 | 12 | I |
| C[C@@H]1CCC2=CC=CC(O[C@@H]3CCS(=O)(=O)C3)=C2N1 | 12 | I |
| CC1=CC(CN2C(=S)NC3=CC(Cl)=CC=C23)=NO1 | 12 | I |
| Cl[C@@H]([C@H]1CCCCO1)C1=CC2=C(OCC(=O)N2)C=C1 | 12 | I |
| CN1CCCN(CC1)C1=NC2=C(S1)[C@H](O)CC(C)(C)C2 | 12 | I |
| COC1=C[C@]23C[C@H](O)CC=C2O[C@@H]2C[C@H](C)CC(C=N1)=C32 | 12 | I |
| CC1=CC=CN\C1=N/S(=O)(=O)C1=CC=C(F)C=C1F | 12 | I |
| ClC1=CC=C(CN2C(=O)NC(=O)C22CCCC2)S1 | 12 | I |
| C[C@H]1COC2=CC=C(C=C2OC1)[C@H](O)C1=C(C)OC(C)=C1 | 12 | I |
| CS(=O)(=O)C1=CC=C2C(NC3=CC=CC=C3[S@@]2=O)=C1 | 12 | I |
| CC1=CN(CC2=CC=C(Cl)C(Cl)=C2)C(=O)NC1=O | 12 | I |
| BrC1=CC=C2NC(=NC(=O)C2=C1)C1=CC=CO1 | 12 | I |
| CC(C)[C@H]1N([C@@H]2CCCC[C@@H]2C)C(=O)C(C)(C)NC1=O | 12 | I |
| CC[C@H]1NC(=O)[C@@H](C)N(C1=O)C1=CC=C(Cl)C(F)=C1 | 12 | I |
| CC[C@H]1N(C(=O)[C@@H](C)NC1=O)C1=CC=C(Cl)C(F)=C1 | 12 | I |
| CC[C@H]1N(C(=O)[C@H](C)NC1=O)C1=CC=C(Cl)C(F)=C1 | 12 | I |
| CC[C@@]1(C)NC(=O)CCN([C@H]2CC[C@H](C)C[C@@H]2C)C1=O | 12 | I |
| CC[C@H]1N(C2C[C@@H](C)C[C@@H](C)C2)C(=O)C(C)(C)NC1=O | 12 | I |
| C[C@H]1CC[C@@H](C[C@@H]1C)N1[C@H](C)C(=O)NC2(CCCC2)C1=O | 12 | I |
| CC(C)[C@H]1N([C@H]2CC[C@H](C)[C@H]2C)C(=O)C(C)(C)NC1=O | 12 | I |
| CC[C@H]1CO[C@@H](C)CN1C1=NC2=C(CCC[C@@H]2O)S1 | 12 | I |
| C[C@@H]1SC(=N)C(=C1)C1=NC(=NO1)[C@H]1CC2=C1C=CC=C2 | 12 | I |
| CNC1=NC(=NC=C1Br)[C@]1(C)CCCCO1 | 12 | I |
| C[C@H]1CC[C@@H](C1)C1=NOC(=N1)[C@H]1NCCC2=CC=CC=C12 | 12 | I |
| BrC1=CNC(=NC1=O)C1=CC2=C(OCC2)C=C1 | 12 | I |
| O[C@H](C1=CC2=C(OCO2)C(Cl)=C1)C1(CCCC1)C#N | 12 | I |
| O[C@H](C1=CC2=C(OCCCO2)C=C1)C1(CCCCC1)C#N | 12 | I |
| OC[C@H]1CCCO[C@H]1C1=CC2=C(OCCO2)C(Cl)=C1 | 12 | I |
| OC1=C(CN2CCC3(C2)OCCO3)C2=CC=CC=C2C=C1 | 12 | I |
| ClC1=CC=C(C=C1)[C@H]1C(=O)NC(=O)C[C@]11CCCOCC1 | 12 | I |
| CN1C=C(C=N1)N1C(=S)NC2=CC(Br)=CC=C12 | 12 | I |
| C[C@H]1CN(C(=O)NC1=O)C1=CC(Br)=CC=C1F | 12 | I |
| O[C@H]1COC2=C1C=CC(O[C@H]1CCOC3(CCCC3)C1)=C2 | 12 | I |
| [O-]C(=O)[C@H]1CCC[NH+](C1)[C@H]1CCOC2(CCCCC2)C1 | 12 | I |
| C[C@H]1[C@@H](CC[NH+]1[C@@H]1CCOC2(CCCCC2)C1)C([O-])=O | 12 | I |
| OC(=O)C1(CCCCCC1)[C@H]1CCO[C@@]2(CCOC2)C1 | 12 | I |
| C[C@H]1CN2CCCC[C@@H]2C[NH+]1[C@@H]1CCCC[C@@H]1C([O-])=O | 12 | I |
| CN1N=C(C)C([C@@H](O)C2=CN=CC3=C2C=CC=C3)=C1Cl | 12 | I |
| C[C@H]1CN(CC(C)(C)O1)C(=O)NCC(Br)=C | 12 | I |
| C[C@@H]1CS(=O)(=O)C2=CC(Cl)=C(Cl)C=C2NC1=O | 12 | I |
| CC1=CC=C2C=C(C=CC2=N1)C1=NC(=O)C2=C(CSC2)N1 | 12 | I |
| OC[C@@H]1CCC2=NN=C([C@@H]3CC[C@@H]4CCCC[C@H]4C3)N2C1 | 12 | I |
| OC[C@@H]1CCC2=NN=C(N2C1)C12CC3CC(CC(C3)C1)C2 | 12 | I |
| CC1=NOC(=O)\C1=C\NC1=CC=C(Br)C=C1 | 12 | I |
| C\C(=N/N=C1\NC(=O)CS1)C1=CC=C(Br)C=C1 | 12 | I |
| FC1=CC=C(C=C1)S(=O)(=O)C1=CNC2=CC=CC=C2C1=O | 12 | I |
| CC1=CC(\C=C2\SC(=O)NC2=O)=C(C)N1C1=CC=C(F)C=C1 | 12 | I |
| CC1=CC=C(O1)\C=C1/SC(=S)N(C1=O)C1=CC=CC(O)=C1 | 12 | I |
| CC(=O)NC1=NC(=O)\C(S1)=C/C1=CC=C(Br)C=C1 | 12 | I |
| CS(=O)(=O)NC1=CC=C(Br)C2=C1N=CC=C2 | 12 | I |
| O[C@@H]1CS(=O)(=O)C[C@H]1OC1=CC=C(Cl)C2=CC=CC=C12 | 12 | I |
| CC1=CC=C(C=C1)N1C(=O)CCS[C@]11C(=O)NC2=CC=CC=C12 | 12 | I |
| O[C@H](C1=CC=CO1)C1=CC2=C(OCCO2)C=C1Br | 12 | I |
| Br[C@@H]([C@H]1CCCO1)C1=CC2=C(OCC(=O)N2)C=C1 | 12 | I |
| CCN1C(=NC2=CC(Br)=CC=C12)[C@H]1CNCCO1 | 12 | I |
| O=S(=O)(N1CCCSCC1)C1=CC2=C(NCCC2)C=C1 | 12 | I |
| CC1=C(C)C2=C(S1)N=C(CN1CCC[C@H](O)C1)N=C2Cl | 12 | I |
| CC1=NN(C(C)=C1Br)C1=C(Cl)C=C(CO)C=N1 | 12 | I |
| CS[C@H]1N(C)C(=O)C2=CC(=O)NC(=C12)C1=CC=C(F)C=C1 | 12 | I |
| CN1N=CC=C1[C@H](O)C1=CC(Br)=C(Br)O1 | 12 | I |
| C[C@H]1CCC2=C(C1)SC=C2C(=O)N[C@]1(C)CCS(=O)(=O)C1 | 12 | I |
| CC1(C)N(CC2=CC(Br)=CS2)C(=O)NC1=O | 12 | I |
| CCN1C=C(C(C)=N1)C1=C(I)C=NN1 | 12 | I |
| FC1=C(CC(=O)N2[C@@H]3CSC[C@@H]3OC2=N)C(Cl)=CC=C1 | 12 | I |
| CC1=C(C(=O)NCC#C)C(=O)OC2=CC=C(Br)C=C12 | 12 | I |
| CC1(C)CNCCN1S(=O)(=O)C1=CC=C(Br)C=C1 | 12 | I |
| CNC1=C(I)C=NC(=N1)[C@@H]1C[C@H]2CC[C@@H]1O2 | 12 | I |
| C[C@@H]1NC(=O)[C@@H](C)N(C1=O)C1=CC=C(Br)C=C1F | 12 | I |
| CC[C@@H]1NC(=O)[C@@H](C)N(C1=O)C1=CC=C(Br)C=C1 | 12 | I |
| C[C@H](N1[C@@H](C)C(=O)N[C@H](C)C1=O)C1=CC=C(Br)C=C1 | 12 | I |
| CC[C@@]1(C)NC(=O)[C@@H](C)N(C1=O)C1=CC(F)=C(F)C=C1F | 12 | I |
| C[C@@H]1CN([C@H](C)CN1)C(=O)C1=CC(Br)=CN1C1CC1 | 12 | I |
| C[C@H]1CN([C@@H](C)CN1)C(=O)C1=CC(Br)=CN1C1CC1 | 12 | I |
| C[C@@H]1CN([C@H](C)CN1)S(=O)(=O)C1=CC=C(Br)C=C1 | 12 | I |
| CC[C@H]1NC(=O)CCN(C2=CC=C(Br)C(F)=C2)C1=O | 12 | I |
| CC[C@@H]1NC(=O)[C@@H](C)N(C1=O)C1=CC=C(Br)C(F)=C1 | 12 | I |
| O[C@H]1CCC2(C[C@H]1N1CCC[C@H](C1)C(F)(F)F)OCCO2 | 12 | I |
| C[C@@H]1C[C@](O)(CC2=C(Br)C(C)=NN2C)CCO1 | 12 | I |
| CC(C)C1=C(Br)C(=O)NC(=N1)[C@H]1CCCOC1 | 12 | I |
| OC1CC(CN2C(=O)C3=C(C=C(Br)C=C3)C2=O)C1 | 12 | I |
| ClC1=CC2=C(C=C1)N(C[C@@H]1CN3CCC[C@@H]3CO1)C(=S)N2 | 12 | I |
| ClC1=CN=CC(Cl)=C1CN1CC[C@@]2(C1)CCCNC2=O | 12 | I |
| C[C@H]1CC[C@@H](NC(=O)N(C)C2CCS(=O)CC2)[C@H](C)C1 | 12 | I |
| [O-]C(=O)[C@@H]1[NH+](CCC2=C1C=CS2)[C@@H]1CCOC2(CCC2)C1 | 12 | I |
| CC(C)C1=C(Br)C(=O)NC(=N1)[C@@H]1CSCCO1 | 12 | I |
| C[C@@H]1N(CC2=CSC=C2C)C(=O)C2(CCCCC2)NC1=O | 12 | I |
| O[C@@H]1CCCOC11CCN(CC2=CC3=C(OC=C3)C=C2)CC1 | 12 | I |
| COC1=C(\C=C2\SC(=S)NC2=O)C=C2O[C@@H](C)CC2=C1 | 12 | I |
| CC1=C(I)C=C\C(N1)=N\S(C)(=O)=O | 12 | I |
| CC1=C(\C=C2\SC(=S)N(C2=O)C2=CC=C(C)C=C2)C(C)=NN1 | 12 | I |
| CN1C=C(Br)C(=O)C(NC(=O)C2=CSC=C2C)=C1 | 12 | I |
| CN1CC2=CC=C(NC(=O)C3=CC4=CC=CC=C4OC3)C=C2C1 | 12 | I |
| CC(C)N1N=CC(Br)=C1[C@]1(O)CCN2CC[C@@H]1C2 | 12 | I |
| C\C(=C/C1=CC=C(F)C=C1)C(=O)N1CCC[C@@]2(CNC(=O)C2)C1 | 12 | I |
| BrC1=CC=C(NN2C(=O)C=CC3=CC=CC=C23)C=C1 | 12 | I |
| CC1=CC=C(C=C1C)N1C(=S)S\C(=C/C2=CC=C(O)C=C2)C1=O | 12 | I |
| FC1=CC(NC(=O)C2=CC3=CC(Cl)=CC=C3OC2)=C(F)C=C1 | 12 | I |
| C[C@@H](Cl)C(=O)NC1=NC2=C(S1)C=C(C)C(Br)=C2 | 12 | I |
| C[C@H](Cl)C(=O)NC1=NC2=C(S1)C=C(C)C(Br)=C2 | 12 | I |
| FC(F)(F)[C@H]1C[C@@H](NC2=CC=NN12)C1=CC2=CC=CC=C2C=C1 | 12 | I |
| OC1=CC=C2C(=O)\C(OC2=C1)=C\C1=CC=C(C=C1)C(F)(F)F | 12 | I |
| CN1N=CC2=C1CCC[C@H]2NC1=C(F)C=C(F)C=C1Br | 12 | I |
| ClC1=C(C=C(Br)C=C1)C1=CC2=C(S1)C(=O)NC=N2 | 12 | I |
| C[C@@]12CN3C[C@@](C)(C[NH+](C1)[C@@H]3C1=CC=C(Br)C=C1[O-])C2 | 12 | I |
| ClC1=CC=C(C=C1)\N=C1\N[C@H]2[C@@H](CS1)OC1=CC=CC=C21 | 12 | I |
| CC1(C)CCC[C@@]2(C)[C@H]1[C@H](O)C[C@@]1(C)O[C@](C)(CC(=O)[C@H]21)C=C | 12 | I |
| Br[C@@H](C1CCCCC1)C1=CC2=C(OCC(=O)N2)C=C1 | 12 | I |
| Cl[C@H](C1=CC=C(Br)C=C1)C1=CC=C2NC(=O)OC2=C1 | 12 | I |
| FC1=CC=C(Cl)C=C1[C@@H](Cl)C1=CC2=C(NC(=O)O2)C=C1 | 12 | I |
| CC1=CC=C(O1)[C@H](Br)C1=CC2=C(NC(=O)C2)C=C1Cl | 12 | I |
| CC1=C(SC=N1)C(=O)NC1=CC(Cl)=C(Cl)C=C1Cl | 12 | I |
| CC1(CCCCC1)N1C(=S)NC2=C(C3=C(CCC3)S2)C1=O | 12 | I |
| CC1=CC2=C(C(=O)O\C2=C/C2=CC=C(Cl)C=C2Cl)C(=S)N1 | 12 | I |
| FC1=CC=CC([C@H](Br)C2=CC=C3NC(=O)OC3=C2)=C1F | 12 | I |
| CC1=CC=C(C=C1Cl)[C@H](O)C1=CC2=C(OCCO2)C=C1Cl | 12 | I |
| O[C@H](C1=CC=C(F)C=C1)C1=CC2=C(OCO2)C(Br)=C1 | 12 | I |
| O[C@@H](C1=CC2=C(OCO2)C(Br)=C1)C1=C(F)C=CC(F)=C1 | 12 | I |
| O[C@H](C1=CC2=C(OCO2)C(Br)=C1)C1=CC(F)=CC(F)=C1 | 12 | I |
| O[C@H](C1=CC=C(Cl)C=C1)C1=CC2=C(OCCO2)C(Cl)=C1 | 12 | I |
| CC1=CC(Br)=CC=C1N[C@@H]1CCCS(=O)(=O)C1 | 12 | I |
| C[C@H]1COC2=CC=C(C=C2OC1)[C@H](O)C1=CC=C(Cl)S1 | 12 | I |
| C[C@H]1CCC[C@@H](C1)[C@H](O)C1=CC2=C(OCCO2)C(Br)=C1 | 12 | I |
| CC1=C(Br)C=CC(NC(=O)C2=CC=C(F)C=C2Cl)=N1 | 12 | I |
| C[C@H]1C[C@@H]1C1=NC(=C(I)C(=O)N1)C(C)(C)C | 12 | I |
| CNC1=NC(=NC(C)=C1I)[C@@H]1CCC[C@H](C)C1 | 12 | I |
| FC1=CC=CC(F)=C1NC1=C(I)C=NC=N1 | 12 | I |
| CC1=CC=C(NC2=C(I)C=NC=N2)C(C)=C1 | 12 | I |
| CC1=CC=C(NC2=C(I)C=NC=N2)C=C1 | 12 | I |
| CNC1=NC(=NC(C)=C1C)C1=CC=C(Br)C(C)=C1 | 12 | I |
| C[C@H]1CC[C@H](NC2=C(Br)C=NC(Cl)=N2)[C@H](C)C1 | 12 | I |
| O[C@@H](C1=CC=C(Br)O1)[C@@]1(CCCC2=CC=CC=C12)C#N | 12 | I |
| O[C@H](C1=CC=CC(Br)=C1)[C@]1(CCOC2=CC=CC=C12)C#N | 12 | I |
| C[C@H]1CCCC[C@@H]1N1C(=S)NC2=CC(Br)=CN=C12 | 12 | I |
| CC[C@@H]1CCCC[C@H]1N1C(=S)NC2=CC(Br)=CN=C12 | 12 | I |
| CC[C@H]1CCCC[C@H]1N1C(=S)NC2=CC(Br)=CN=C12 | 12 | I |
| C[C@H]1CC[C@H](C[C@@H]1C)N1C(=S)NC2=CC(Br)=CN=C12 | 12 | I |
| CC1=CC(=CC(F)=C1)[C@H](O)C1=CC2=C(OCCO2)C(Cl)=C1 | 12 | I |
| BrC1=CC=CC=C1N[C@H]1CCO[C@]2(CCOC2)C1 | 12 | I |
| CC1=CC=C(C=C1Cl)N1N=C(C2CC2)C(Br)=C1O | 12 | I |
| CC1=CC=C([C@H](O)C2=CC3=C(OCCO3)C=C2Cl)C(F)=C1 | 12 | I |
| CC[C@H](Cl)C1=NC(=O)C2=C(N1)SC1=C2C[C@@H](C)C[C@H]1C | 12 | I |
| COC1=CC=C(C=C1)N1C(=S)NC2=CC(Cl)=C(F)C=C12 | 12 | I |
| CC1=CC=C(CN2C(=S)NC3=CC(Br)=C(F)C=C23)O1 | 12 | I |
| CN1N=C(C2=C1\C(CNC2)=C\C1=CC=C(Cl)C=C1)C(F)(F)F | 12 | I |
| ClC1=CC=CC=C1C[C@@H]1OC(=O)C2=C1C=CNC2=O | 12 | I |
| OC1=CC=CC=C1P1(=O)COC2=CC=CC=C2OC1 | 12 | I |
| CC1=C(C(=O)NC2=CC=CC(Cl)=C2F)C(C)=NO1 | 12 | I |
| COC(=O)C1=CC(Br)=CC2=C1NC(=O)C2 | 12 | I |
| ClC1=CC2=C(NC(=O)OC2=O)C(Br)=C1 | 12 | I |
| CC1=C(C2=CSC=N2)C(=O)C2=C(O1)C=C(O)C=C2C | 12 | I |
| [O-]C(=O)\C=C\C1=C[NH+]=C2C=CC(Br)=CN12 | 12 | I |
| [O-]C1=C2C=CSC2=NC(=S)N1[C@@H]1C[NH+]2CCC1CC2 | 12 | I |
| CN1N=CC2=C1CCC[C@H]2NC1=CC=C2OCCC2=C1 | 12 | I |
| CCN1C(=O)S\C(=C\C2=CNC3=C2C=CC=C3)C1=O | 12 | I |
| CC1=CC(CN2C(C)=CC3=C2CC(C)(C)C[C@H]3O)=NO1 | 12 | I |
| Cl[C@@H]([C@H]1CCCCO1)C1=CC2=C(NC(=O)O2)C=C1 | 12 | I |
| C[C@H]1NCCN2C1=NN=C2C12CC3CC(CC(C3)C1)C2 | 12 | I |
| CC[C@@H]1CN(CCO1)C1=NC2=C(S1)[C@H](O)CCC2 | 12 | I |
| C[C@H]1CC[C@@H]([C@@H](C1)[NH+]1CCO[C@H]2CCC[C@H]12)C([O-])=O | 12 | I |
| C[C@@H]1COC2=CC=C(C=C2OC1)[C@@H](O)C1=C(C)OC=C1 | 12 | I |
| [O-]C1=N[C@@H]([NH+]2CCCC[C@H]12)C1=CC2=CC=CC=C2OC1 | 12 | I |
| ClC1=CNC(=O)C2=C1C=CC(=C2)S(Cl)(=O)=O | 12 | I |
| O=C1NC(=NC2=C1C(=S)SS2)N1CCCC1 | 12 | I |
| OC1=CC(OC2=NC=C(Br)C=N2)=CC=C1 | 12 | I |
| FC1=C(C=CC(Cl)=C1)N1N=CC2=C1N=CNC2=S | 12 | I |
| CNC1=NC(=NC(C)=C1Br)N1CCCC1 | 12 | I |
| O[C@@H]1CCCN(C1)C1=NC(Cl)=NC=C1Br | 12 | I |
| CC[C@@H]1N(C)C2=NC=C(Br)C=C2NC1=O | 12 | I |
| CC(C)[C@H]1N([C@@H]2CCCC[C@H]2C)C(=O)[C@@H](C)NC1=O | 12 | I |
| CC(C)[C@H]1NC(=O)[C@@H](C)N([C@H]2CCC[C@H](C)C2)C1=O | 12 | I |
| CCC1CCC(CC1)N1[C@H](C)C(=O)NC(C)(C)C1=O | 12 | I |
| C[C@@H]1CC[C@H]([C@H](C)C1)N1[C@H](C)C(=O)NC(C)(C)C1=O | 12 | I |
| CC(C)[C@H]1NC(=O)CN([C@@H]2CC[C@H](C)C[C@H]2C)C1=O | 12 | I |
| C[C@H]1CC[C@@H]([C@H](C)C1)N1CCC(=O)NC(C)(C)C1=O | 12 | I |
| C[C@H]1CC[C@H]([C@H](C)C1)N1CCC(=O)NC(C)(C)C1=O | 12 | I |
| C[C@@H]1CC[C@@H](C[C@@H]1C)N1[C@H](C)C(=O)NC(C)(C)C1=O | 12 | I |
| CC[C@@]1(C)NC(=O)[C@@H](C)N([C@@H]2CC[C@H](C)[C@@H]2C)C1=O | 12 | I |
| CS(=O)(=O)[C@@H]1CCC[C@]11CNCC2(CCCCC2)O1 | 12 | I |
| CC(C)[C@H]1N([C@@H]2CCCCC[C@@H]2C)C(=O)CNC1=O | 12 | I |
| ClC1=C(NN=C1C(=O)N1C[C@H]2CC[C@@H]1C2)C1CC1 | 12 | I |
| CSC1=NC=NC2=NNC(I)=C12 | 12 | I |
| C[C@H]1NCCC[C@@H]1N1C(=O)CC2(CCCCCC2)C1=O | 12 | I |
| CC1=CC=C(O1)[C@H](O)[C@]1(CCOC2=C1C=CC=C2)C#N | 12 | I |
| FC1=CC=C(C=C1)[C@H]1C(=O)NC(=O)C[C@]11CCCOCC1 | 12 | I |
| CC(C)[C@H]1N([C@@H]2CCCC2(C)C)C(=O)[C@H](C)NC1=O | 12 | I |
| CC1(C)C[C@@](O)(CCO1)[C@@H]1CCO[C@]2(CCOC2)C1 | 12 | I |
| CCC[C@@H]1C(=O)NC(=O)C[C@@]11CC(C)(C)OC1(C)C | 12 | I |
| C[C@H]1CN(C[C@@H]2[C@H](O)C(C)(C)OC2(C)C)[C@H](C)CO1 | 12 | I |
| C[C@@]1(CCCO1)[C@@H](Cl)C1=CC=C2NC(=O)OC2=C1 | 12 | I |
| FC(F)(F)C1=CC(Br)=CN2C(=O)NN=C12 | 12 | I |
| C[C@H]1N(C(=O)CNC1=O)C1=CC(Cl)=CC(Cl)=C1 | 12 | I |
| C[C@H]1CN([C@@H](C)CO1)C(=O)C1=CC(Cl)=C(Cl)N1 | 12 | I |
| CSC1=CC=C(C=C1)[C@H]1CC(O)=NC2=C1C=NN2C | 12 | I |
| C[C@@]12[C@H]3C[C@H](C=C3)[C@H]1C(=O)N(C2=O)C1=CC=C(O)C=C1 | 12 | I |
| C[C@]12C[C@@H](O)[C@@H]3[C@H](CCC4=CC(=O)C=C[C@@]34C)[C@@H]1CCC2=O | 12 | I |
| O=C1C[C@H](C2=C(N1)SC1=C2CCCC1)C1=CC=NC=C1 | 12 | I |
| ClC1=NC=NC2=C1C(I)=CN2 | 12 | I |
| CN[C@@H]1[C@@H](C)CS(=O)(=O)C2=CC3=C(CCC3)C=C12 | 12 | I |
| CN[C@H]1[C@@H](C)CS(=O)(=O)C2=CC3=C(CCC3)C=C12 | 12 | I |
| CC1=CC=C(Br)C=C1N1C=NNC1=S | 12 | I |
| CN1C(=NC2=CC(Br)=CC=C12)[C@@H]1CCCN1 | 12 | I |
| O[C@H]1N2CCCC2=NC2=C1C=C(Br)C=C2 | 12 | I |
| C[C@@H]1CCC2=C(C1)SC1=C2C(=O)N(C)C(=S)N1 | 12 | I |
| CN1C=C(\C=C2/SC(=O)NC2=S)C2=C1C=CC=C2 | 12 | I |
| C[C@@H](O)C1=CC2=C(OCCCO2)C=C1Br | 12 | I |
| C[C@H]1CCC2=C(C1)C(=S)SC(NC(C)=O)=C2C#N | 12 | I |
| O[C@@H]1CCCC2=C1C=CN2C1=NC=C(Br)S1 | 12 | I |
| CN[C@H]1[C@H](C)S(=O)(=O)C2=C(Cl)C=CC(Cl)=C12 | 12 | I |
| CN[C@@H]1[C@@H](C)S(=O)(=O)C2=C(Cl)C=CC(Cl)=C12 | 12 | I |
| COC1=CC2=C(C=N1)C(I)=CN2 | 12 | I |
| C[C@H]1C[C@H]1N1C(=S)NC2=C(C(C)=C(C)S2)C1=O | 12 | I |
| C[C@H]1C[C@@H]1N1C(=S)NC2=C(C3=C(CCC3)S2)C1=O | 12 | I |
| CC1=CC(=CC=C1Br)N1C=CC(=O)NC1=S | 12 | I |
| O[C@]1(CC2=CC=CC=C2C2=NCCN12)C1=CC=C(F)C=C1 | 12 | I |
| O[C@@]1(CCC2=CC=CC=C12)C1=CC=C2OCCOC2=C1 | 12 | I |
| O[C@]1(CCC2=CC=CC=C12)C1=CC=C2OCCOC2=C1 | 12 | I |
| O=C1C[C@H]2CC[C@@H](CN1)N2C1CSCCSC1 | 12 | I |
| O[C@H]1CN(C(=O)C1)C1=C(Cl)C=C(Br)C=C1 | 12 | I |
| BrC1=CC2=C(N=C(N2)[C@@H]2CCCS2)N=C1 | 12 | I |
| C[C@@]12CCC(=O)[C@H]3O[C@@]13C[C@]1(C)CCCC(=C)[C@]1(O)C2 | 12 | I |
| IC1=CC2=C(NC(=O)CO2)C=C1 | 12 | I |
| CN1C(=S)NN=C1C1=CC=C(Br)S1 | 12 | I |
| FC1=C(Br)C=CC(=C1)N1C=CC(=O)NC1=S | 12 | I |
| BrC1=CC=CC(=C1)C1=CN=C2CNCCN12 | 12 | I |
| C[C@@H]1CC[C@@H](C1)C1=NC(C)=C(Br)C(=O)N1 | 12 | I |
| C[C@H]1C[C@H]1C1=NC(C)=C(I)C(=O)N1 | 12 | I |
| CC1=C(Br)C(=O)N=C(N1)C1=CSC=C1 | 12 | I |
| CC1=NC2=C(CCNC2)N1C1=CC(F)=C(F)C=C1F | 12 | I |
| CC1=NNC(=N1)C1=CSC(I)=C1 | 12 | I |
| C[C@H]1CCC[C@]2(CNC[C@H](O2)C2=C(C)OC(C)=C2)C1 | 12 | I |
| C1CC2=C(C=CS2)[C@@H](N1)C1=NC2=C(S1)C=CN=C2 | 12 | I |
| CN(C)C1=CC2=C(C(=O)NC2)C(I)=C1 | 12 | I |
| C[C@@H]1CS(=O)(=O)C[C@@H](N1)C1=CC=C(F)C=C1Cl | 12 | I |
| C[C@@H]1CCN[C@@H]2CC[C@@H](C[C@@H]2S1(=O)=O)C(F)(F)F | 12 | I |
| O[C@H]1[C@@H](CC2=CC=CC=C12)[C@@H]1CS(=O)(=O)C2=C1C=CC=C2 | 12 | I |
| CC1(C)C[C@@](O)(CCO1)[C@@H]1CCO[C@]2(CCSC2)C1 | 12 | I |
| C[C@@H]1C[C@@H](C)[C@@H]([C@H](O)C1)N1CCN2CCCC[C@@H]2C1 | 12 | I |
| C[C@@H]1C[C@@](O)(CS1)C1=CC=C2OCCCOC2=C1 | 12 | I |
| C[C@H]1CNCCN1C(=O)C12CC3C[C@](C)(C[C@](C)(C3)C1)C2 | 12 | I |
| C[C@H]1CS(=O)(=O)C[C@@H](N1)C1=C(F)C=C(C)C(F)=C1 | 12 | I |
| IC1=CNC2=CN=CC(C#N)=C12 | 12 | I |
| ClC1=C2C(I)=NNC2=NC=C1 | 12 | I |
| O[C@H]1C[C@@H](OC2=C1C=C(Br)C=C2)[C@H]1CCOC1 | 12 | I |
| FC(F)(F)C1=CC2=C(C=C1)S(=O)(=O)CCCN2 | 12 | I |
| COC1=C2O[C@H]3[C@H](C)[C@H](OC4=CC=CC=C34)C2=CC=C1 | 12 | I |
| [O-][N+](=O)C1=CC(Br)=C(Br)C=C1 | 12 | I |
| O=C1\C(CCCC\C1=C/C1=CC=CO1)=C\C1=CC=CO1 | 12 | I |
| CC1=NN(C(=O)\C=C\C2=C(F)C=CC=C2Cl)C(C)=C1 | 12 | I |
| CC1=NC2=C(C#N)C(=CN2C(C)=C1)C1=C(Cl)C=CS1 | 12 | I |
| FC1=CC=C(\C=C2/N=C3SC4=CC=CC=C4N3C2=O)C=C1 | 12 | I |
| C[C@]12CC[C@@H]3[C@H](CCC4=CC(=O)CC[C@]34C)[C@@H]1CCC(=O)O2 | 12 | I |
| N#CCSC1=NC=NC2=C1C1=C(CCCCC1)S2 | 12 | I |
| CON(C)C(=O)C1=CC2=C(S1)C1=CC=CC=C1S2 | 12 | I |
| CC1=NC2=CC(=NN2C(C)=C1)C1=CC=C(Br)C=C1 | 12 | I |
| ClC1=CC2=C(OCC3=NC(=CN23)C2=CC=CC=C2)C=C1 | 12 | I |
| CC[C@@]12CCCN3CCC4=C([C@H]13)N(C1=CC=CC=C41)C(=O)C2 | 12 | I |
| CC1=C(C)C(Cl)=C2C=C3OCCCOC3=CC2=N1 | 12 | I |
| FC1=CC(Cl)=C(OC2=NSN=C2Cl)C=C1 | 12 | I |
| ClCC1=CC2=CC=CC=C2N=C1N1C=C(Cl)C=N1 | 12 | I |
| CC1=NN2C(N=C(C)C=C2C)=C1C1=CC=C(Cl)C=C1 | 12 | I |
| Cl\C(=C(\C#N)C1=CC=CC=C1)C1=CC=C2OCOC2=C1 | 12 | I |
| C[C@@H]1CCC[C@H](C1)N(C)C(=O)C1(CCCCCC1)C#N | 12 | I |
| CC1=CC(C=O)=NC(=N1)C1=CC=C(Br)C=C1 | 12 | I |
| C[C@H]1CN(C[C@@H](C)O1)[C@@H]1C[C@@H](CC[C@@H]1C#N)C(C)(C)C | 12 | I |
| CN1C2=CC=CC=C2C2=C1C=CC(=C2)S(Cl)(=O)=O | 12 | I |
| CN1CCN(CC1)\N=C(\C)C1=CC=C(Cl)C(Cl)=C1 | 12 | I |
| C[C@@H](Cl)C1=C(C)N=C(N=C1)C1=CC=C2C=CC=CC2=N1 | 12 | I |
| CC1=NN(CC2=C(Cl)C=CC(Cl)=N2)C=C1Cl | 12 | I |
| CC1=NN=C(Cl)N1C1=CC=C(C)C(Br)=C1 | 12 | I |
| FC(F)(F)[S@@](=O)C1=CN=C(Br)C=N1 | 12 | I |
| CCCN1C2=CC=CC=C2S(=O)(=O)C2=C1C=CC=C2 | 12 | I |
| C[C@H]1CN(C(=O)\C=C\C2=CC=CO2)C2=CC=CC=C2C1 | 12 | I |
| CC(C)N(C)C(=O)C1=C(C)C2=CC=CC(Cl)=C2O1 | 12 | I |
| CC[C@H]1CCCCN1C1=NN=C(Br)S1 | 12 | I |
| BrC1=NN=C(S1)N1CCC[C@@H]2CCCC[C@H]12 | 12 | I |
| BrC1=NN=C(S1)C1=CN=CC2=CC=CC=C12 | 12 | I |
| CC1=CC=C(C=C1)C1=C(Br)N2C=CC=NC2=N1 | 12 | I |
| FC1=C(Cl)N=C(N=C1Cl)C1=NC=C(Cl)C=C1 | 12 | I |
| ClC1=CC(Cl)=C(N2C=CC=C2)C2=NSN=C12 | 12 | I |
| C[C@@H]1CC[C@@H](C)N(C1)C1=C(CCl)N2C=CSC2=N1 | 12 | I |
| CN1C=CN=C1C1=NC2=CC(Br)=CC=C2S1 | 12 | I |
| C[C@H]1CN(C[C@@H]1C)C1=C(CCl)N2C=CC=CC2=N1 | 12 | I |
| C[C@@H]1CC[C@H]([C@H](C)C1)N1C(CCl)=NC2=CN=CC=C12 | 12 | I |
| C[C@H]1CC[C@H](C[C@@H]1C)N1C(CCl)=NC2=CN=CC=C12 | 12 | I |
| C[C@@H](Cl)C1=NC2=CN=CC=C2N1[C@@H]1CC[C@@H](C)[C@H]1C | 12 | I |
| C[C@@H](Cl)C1=NC2=C(C)C=CN=C2N1C1=CC=C(C)C=C1 | 12 | I |
| CC1=CC=NC2=C1N=C(CCl)N2C1(C)CCCC1 | 12 | I |
| CC1=CC2=C(N=C1)N(C1C(C)(C)C1(C)C)C(CCl)=N2 | 12 | I |
| FC(F)(F)C1=NC2=C(C=C1)N=C(Cl)N=C2Cl | 12 | I |
| COC1=CC=C2C[C@H]3N(C)CC[C@@]45C(OC1=C24)=C[C@@H](C)C=C35 | 12 | I |
| BrC1=CC=C(\C=C/C(=O)N2C=CC=N2)C=C1 | 12 | I |
| C[C@H]1CCC=C(C)[C@H]1CN1CCOC2(CCOCC2)C1 | 12 | I |
| CC1=NN(C=C1Cl)C1=C(Br)C=C(C)C=N1 | 12 | I |
| ClS(=O)(=O)C1=CC=C2C(OC3=CC=CC=C23)=C1 | 12 | I |
| BrC1=CC=C(C=C1)S(=O)(=O)[N-]C1=CC=C(C=C1)C#C | 12 | I |
| CC1=CC(C([O-])=O)=C(C)N1C1=CC=C(Br)C=C1 | 12 | I |
| COC1=CC=C2C=CC=CC2=C1\C=C1\SC(=S)N=C1[O-] | 12 | I |
| CC1=CC(\C=C\C([O-])=O)=C(C)N1C1=CC=C(Br)C=C1 | 12 | I |
| [S-]C1=NN=C(C2CC2)N1C1=C(Cl)C(Cl)=CC=C1 | 12 | I |
| [S-]C1=NN=C(C2CC2)N1C1=CC=C(Br)C=C1 | 12 | I |
| CSC1=CC=CC2=C1N=CC(C([O-])=O)=C2C(F)(F)F | 12 | I |
| C[C@@H]([C@@H]1CCCO1)N1C(=S)N=C2SC=CC2=C1[O-] | 12 | I |
| [S-]C1=NN=C(C2CC2)N1C1=C(Cl)C=C(Br)C=C1 | 12 | I |
| CCC1=NN=C([S-])N1C1=C(F)C=C(F)C=C1Br | 12 | I |
| CN(C)C1=CC=C(C=C1I)C([O-])=O | 12 | I |
| [O-]C(=O)CSC1=C2C=CC(Br)=CC2=NC=C1 | 12 | I |
| CC1=C(C=CC=C1F)N1C(=S)N=C2C=CSC2=C1[O-] | 12 | I |
| [O-]C1=C2SC=CC2=NC(=S)N1[C@@H]1C[C@@H]1C1=CC=CC=C1 | 12 | I |
| CC1(C)C[C@@H]1N1C(=S)N=C2SC3=C(CCCC3)C2=C1[O-] | 12 | I |
| CC1=CC(Br)=CC=C1\C([O-])=N\C1=NCCS1 | 12 | I |
| [O-]C1=C2SC=CC2=NC(=S)N1CCC1=CSC=C1 | 12 | I |
| [O-]\C(=N/C1=NCCS1)C1=CC(=C(F)C=C1)C(F)(F)F | 12 | I |
| [O-]C1=C2C=CC=CC2=NC(=S)N1CC1=CC=C(F)C(F)=C1 | 12 | I |
| COCC1=CC([O-])=C2C=C(Br)C=C(F)C2=N1 | 12 | I |
| C[C@H]1C[C@H]1N1C(=S)N=C2SC3=C(CCC3)C2=C1[O-] | 12 | I |
| COCC1=NN(C([S-])=C1)C1=CC=C(Br)C=C1 | 12 | I |
| CC[C@@H](C)CN1C(=S)N=C2SC(C)=C(C)C2=C1[O-] | 12 | I |
| C[C@@H]1OC2=CC=CC=C2C=C1\C=C1\SC([O-])=NC1=S | 12 | I |
| FC1=CC=C(C=C1)S(=O)(=O)[N-]C1=CC(F)=CC(F)=C1F | 12 | I |
| [O-]C1=NC(=O)C2(CCCC2)N1CC1=CC=C(Cl)S1 | 12 | I |
| [O-]C(=O)C1=NC(Br)=CC(Br)=C1 | 12 | I |
| [O-]C(=O)C1=CC(Br)=CC2=C1O[C@@H]1CC=C[C@@H]21 | 12 | I |
| [O-]C(=O)CN1C(I)=CC2=CC=CC=C12 | 12 | I |
| C[C@H]1C[C@H](C)CC(C1)N1C=C(C([O-])=O)C(=C1)C(F)(F)F | 12 | I |
| C[C@H]1C[C@H](C)CC(C1)N1C(=S)N=C2SC=CC2=C1[O-] | 12 | I |
| CCC1=CC=C2N=CC(C([O-])=O)=C(Br)C2=C1 | 12 | I |
| CC1=CC(=CC=C1F)C1=NC=C(Br)C([O-])=N1 | 12 | I |
| [O-]C1=NC(CC(F)(F)F)=NC(C2CC2)=C1Br | 12 | I |
| C[C@@H]1CC[C@@H](C)[C@@H](C1)N1CC[C@@](C1)(C([O-])=O)C(F)(F)F | 12 | I |
| [S-]C1=NN=CN1C1=CC(Br)=C(Cl)C=C1 | 12 | I |
| CC1=C(F)C([O-])=NC(=N1)C1=CC(Br)=CN=C1 | 12 | I |
| [O-]C1=NC(=NC=C1I)[C@@H]1CCCS1 | 12 | I |
| CCCC1=C(Br)C([O-])=NC(=N1)[C@@H](C)Cl | 12 | I |
| CC(C)C1=C(I)C([O-])=NC(=N1)[C@@H](C)Cl | 12 | I |
| COC1=C([O-])C(Br)=CC(CN2C[C@H](C)S[C@@H](C)C2)=C1 | 12 | I |
| [O-]C1=NC2=C(O1)C=C(C=C2)[C@@H](Br)C(F)(F)C(F)F | 12 | I |
| CC1=C(Br)C([O-])=NC(=N1)C1=C(Cl)C=C(Cl)C=N1 | 12 | I |
| CC1(C)CCC[C@@H]1N1C(=S)N=C2SC=CC2=C1[O-] | 12 | I |
| C[C@]1(CN2C(=S)N=C3SC=CC3=C2[O-])CCCO1 | 12 | I |
| CS(=O)(=O)C1=CC=C(C=C1)C1=CC(=CC([O-])=C1)C(F)(F)F | 12 | I |
| [O-]C(=O)C1=CN=CC(=C1)C1=CC(=C(F)C=C1)C(F)(F)F | 12 | I |
| [O-]C(=O)C1=CC(Cl)=NC(I)=C1 | 12 | I |
| CSC1=CC=C(CN2C(=S)N=C3C=CC=CC3=C2[O-])C=C1 | 12 | I |
| [O-]C1=NC2=CN=C(Cl)C=C2C1(Br)Br | 12 | I |
| FC1=CC=C(NC(=O)[C@@H]2CC(=O)N3CCN=C3S2)C=C1 | 12 | I |
| C[NH+]1CCN(CC1)C(=O)[C@@H]1CC[C@](C)(C([O-])=O)C1(C)C | 12 | I |
| CSC1=CC=C(\C=C2\C(=O)N=C3SC=NN3C2=N)C=C1 | 12 | I |
| CN1[C@H](CC2=CC=NC=C2)[C@H](O)C2=CC=CC=C2S1(=O)=O | 12 | I |
| O=S1(=O)CC[C@@H](C1)NC1=C2C(SC3=C2CCC3)=NC=N1 | 12 | I |
| CC(=O)O[C@@H]1[C@@H](O)C2=C(C(=O)OC2)[C@@]2(C)CCCC(C)(C)[C@H]12 | 12 | I |
| CNC(=O)CN1C(=O)C(=O)C2=CC(Br)=CC=C12 | 12 | I |
| FC1=CC=C(C=C1)N1C(=S)NC(=O)\C(=C\C2=CC=CO2)C1=O | 12 | I |
| O=C([C@H]1C[C@H]2CCCC[C@@H]2N1)N1CCS(=O)(=O)CC1 | 12 | I |
| O=S(=O)(N1CCC[C@@H]2CCCC[C@@H]12)N1CCCNCC1 | 12 | I |
| CC1=C(C=NO1)C(=O)NC1=NC=C(Br)S1 | 12 | I |
| N=C1N2C(SC3=CC=CC=C23)=NC(=O)\C1=C\[C@@H]1C=CC=N1 | 12 | I |
| BrC1=CC=C(CN2C(=O)NC(=O)C2=O)C=C1 | 12 | I |
| CNC(=O)CN1C(=O)C(=O)C2=C1C(C)=CC(Br)=C2 | 12 | I |
| O[C@H]([C@H]1CCS(=O)(=O)C1)C1=CC2=C(OCCO2)C(Cl)=C1 | 12 | I |
| O[C@H]1CCC2(C[C@H]1N1CCO[C@H]3CCC[C@H]13)OCCO2 | 12 | I |
| O[C@H]1CCC2(C[C@H]1N1C=CC(=N1)C(F)(F)F)OCCO2 | 12 | I |
| C[C@@H]1CCC2=C(C1)C=C(C(=O)N1CCN(C)CC1)C(=O)N2 | 12 | I |
| CNC1=NC(Cl)=CC(=C1)C(=O)N1C[C@@H](C)OC[C@H]1C | 12 | I |
| OC[C@H]1CCN(C1)C(=O)C1=CC2=C(OCCO2)C(Cl)=C1 | 12 | I |
| CC1=C(C)N=C(NC(=O)[C@@H]2CS[C@]3(C)CCC(=O)N23)S1 | 12 | I |
| FC1=CC(F)=C(C(F)=C1)S(=O)(=O)N1CCNC(=O)CC1 | 12 | I |
| BrC1=CC=C(O1)\C=N\N=C1/NC(=O)CS1 | 12 | I |
| ClC1=CN(C2CC2)C(=C1)C(=O)N1CCNC(=O)CC1 | 12 | I |
| C1COC[C@H](C1)C1=NN=C2[C@H](NCCN12)C1=CC=CC=C1 | 12 | I |
| C[C@H]1CSCCN1S(=O)(=O)C1=C(C)NC(=O)S1 | 12 | I |
| OCC1=NN=C(C=C1)N1C=C(I)C=N1 | 12 | I |
| CN1C=CN=C1CN1C(=S)NC2=C(C3=C(CCC3)S2)C1=O | 12 | I |
| ClC1=CC(CN2CCNC(=O)CC2)=CC2=C1OCCO2 | 12 | I |
| IC1=CNC(=NC1=O)C1=NC=CN=C1 | 12 | I |
| C[C@H]1N(C[C@@H]2CCOC2)C(=O)C2(CCCCC2)NC1=O | 12 | I |
| C[C@@H]([C@@H]1CCCO1)N1[C@@H](C)C(=O)NC2(CCCC2)C1=O | 12 | I |
| C[C@H](N1[C@@H](C)C(=O)NC(C)(C)C1=O)C1=C(C)N=C(C)S1 | 12 | I |
| C[C@@H]1N([C@H]2CCN(C2)C2CC2)C(=O)C2(CCCC2)NC1=O | 12 | I |
| CC[C@@H]1N([C@@H]2CCCOC2)C(=O)C2(CCCC2)NC1=O | 12 | I |
| O=C1C=CNC=C1S(=O)(=O)N1CCC[C@@H]2CCC[C@H]12 | 12 | I |
| CC1(C)CN(CC[S@@]1=O)C(=O)C1=NNC2=C1CCCC2 | 12 | I |
| C[C@H]1CCS(=O)(=O)C[C@@H](N1)C1=CC=C2OCCCOC2=C1 | 12 | I |
| C[C@@H]1CN[C@H](CS(=O)(=O)C1)C1=CC=C2OCCOC2=C1 | 12 | I |
| CC1=C(O)N(N=C1C(F)(F)F)[C@H]1CCS(=O)(=O)C1 | 12 | I |
| FC1=C(Cl)C=C(C=C1)S(=O)(=O)N1CCNC[C@@H]1C#N | 12 | I |
| CC1=C(CN2CCC3(CC2)OCCC[C@H]3O)C(C)=NO1 | 12 | I |
| O[C@@H](C1=CC2=C(OCCCO2)C=C1)C1(CCOCC1)C#N | 12 | I |
| CN1N=C(C)C(CN2C(=S)NC3=C2N=CC(C)=C3)=C1C | 12 | I |
| CC(C)[C@H]1C(=O)NC(=O)C[C@]11CCC[C@@H]1S(C)(=O)=O | 12 | I |
| CN1C=C(C=N1)[C@H]1CC(=O)NC(=O)[C@@H]1C1=CC=C(F)C=C1 | 12 | I |
| CC1=CC(NC(=O)[C@@H]2COCCO2)=NC=C1Br | 12 | I |
| C[C@@H]1SC(=N)C(=C1)C1=NC(=NO1)[C@H]1CSCCO1 | 12 | I |
| FC(F)(F)C1=CC=C(C=C1)C(=O)N1CC(=O)NC(=O)C1 | 12 | I |
| C[C@H]1N(CC(=O)NC1=O)C(=O)C1=CC=C(Br)S1 | 12 | I |

Table S2 Fenobam analogs used in the virtual screening

| Fenobam analogs | | | |
| --- | --- | --- | --- |
| Structure | Cluster Number | Activite / Inactive |
| CN1N=C(C=C1C1=CC=CC=C1)C(=O)NC1=NC(C)=CC=C1 | 2 | A |
| CN1N=C(C(=O)NC2=CC=NC(C)=C2)C(C)=C1C1=CC=C(F)C=C1 | 2 | A |
| CN1N=C(C(=O)NC2=CC=NC=C2)C(C)=C1C1=CC=C(F)C=C1 | 2 | A |
| CN1N=C(C(=O)NC2=CC=CC(C)=N2)C(C)=C1C1=CC=C(C)C=C1 | 2 | A |
| CN1N=C(C(=O)NC2=NC(C)=CC=C2)C(C)=C1C1=CC=C(C=C1)C(F)(F)F | 2 | A |
| CN1N=C(C(=O)NC2=NC(C)=CC=C2)C(C)=C1C1=CC=CC=C1 | 2 | A |
| CN1N=C(C(=O)NC2=CC=NC(F)=C2)C(C)=C1C1=CC=C(F)C=C1 | 2 | A |
| CN1N=C(C(=O)NC2=CC=NC(Cl)=C2)C(C)=C1C1=CC=C(F)C=C1 | 2 | A |
| CN1N=C(C(=O)NC2=NC(C)=CC=C2)C(C)=C1C1=CC=C(F)C=C1 | 2 | A |
| CN1N=C(C(=O)NC2=NC(C)=CC=C2)C(C)=C1C1=CC=C(Cl)C=C1 | 2 | A |
| CN(C)C1=CC=C(C=C1)C1=C(C)C(=NN1C)C(=O)NC1=CC=CC(C)=N1 | 2 | A |
| CN1N=C(C(=O)NC2=NC(C)=CC=C2)C(C)=C1C1=CC=CC(Cl)=C1 | 2 | A |
| CCC1=C(N(C)N=C1C(=O)NC1=CC=CC(C)=N1)C1=CC=CC=C1 | 2 | A |
| CN1N=C(C(=O)NC2=NC(C)=CC=C2)C(C)=C1C1=CC(F)=CC(F)=C1 | 2 | A |
| COC1=CC=C(C=C1)C1=C(C)C(=NN1C)C(=O)NC1=CC=CC(C)=N1 | 2 | A |
| CN1N=C(C(=O)NC2=CC=C(C)C=N2)C(C)=C1C1=CC=C(F)C=C1 | 2 | A |
| CN1N=C(C(=O)NC2=NC(C)=CC=C2)C(C)=C1C1=CC=C(F)C(F)=C1 | 2 | A |
| CN1N=C(C(=O)NC2=NC(C)=CC=C2)C(C)=C1C1=CC=C(Cl)C(Cl)=C1 | 2 | A |
| CN1N=C(C(=O)NC2=CC=C(F)C(C)=N2)C(C)=C1C1=CC=C(F)C=C1 | 2 | A |
| CN1N=C(C(=O)NC2=NC(C)=CC=C2)C(C)=C1C1=CC=C(F)C=C1F | 2 | A |
| CN1N=C(C(=O)NC2=CC=CC(=N2)C(F)(F)F)C(C)=C1C1=CC=C(F)C=C1 | 2 | A |
| CN1N=C(C(=O)NC2=CC(C)=CC=N2)C(C)=C1C1=CC=C(F)C=C1 | 2 | A |
| CN1N=C(C(=O)NC2=CC=C(F)C=N2)C(C)=C1C1=CC=C(F)C=C1 | 2 | A |
| CN1N=C(C(=O)NC2=CC(Cl)=CC=N2)C(C)=C1C1=CC=C(F)C=C1 | 2 | A |
| CN1N=C(C(=O)NC2=CC=C(Cl)C=N2)C(C)=C1C1=CC=C(F)C=C1 | 2 | A |
| CN1N=C(C(=O)NC2=CC=CC(Cl)=N2)C(C)=C1C1=CC=C(F)C=C1 | 2 | A |
| ClC1=CC(OC2=CN=CN=C2)=CC(=C1)C(=O)NC1=CC=CC(Cl)=N1 | 2 | A |
| FC1=CC(OC2=CN=CN=C2)=CC(=C1)C(=O)NC1=CC(Cl)=CC=N1 | 2 | A |
| FC1=CC=NC(NC(=O)C2=CC(Cl)=CC(OC3=CN=CN=C3)=C2)=C1 | 2 | A |
| FC1=CC=CC(NC(=O)C2=CC(Cl)=CC(OC3=CN=CN=C3)=C2)=N1 | 2 | A |
| FC1=CC=C(NC(=O)C2=CC(Cl)=CC(OC3=CN=CN=C3)=C2)N=C1 | 2 | A |
| CN(C1=CN=CN=C1)C1=CC(=CC=C1F)C(=O)NC1=CC=CC(Cl)=N1 | 2 | A |
| CN(C1=CN=CN=C1)C1=CC(Cl)=CC(=C1)C(=O)NC1=CC=CC(F)=N1 | 2 | A |
| CN1N=C(C(=O)NC2=CC=NC(Cl)=N2)C(C)=C1C1=CC=C(F)C=C1 | 2 | A |
| COC1=CC=CC(NC(=O)C2=CC(Cl)=CC(OC3=CN=CN=C3)=C2)=N1 | 2 | A |
| CN1N=C(C(=O)NC2=NC(C)=CC=C2)C(C)=C1C1=CC=NC=C1 | 2 | A |
| CN1N=C(C(=O)NC2=NC(C)=CS2)C(C)=C1C1=CC=C(F)C=C1 | 2 | A |
| CC1=NC(C(=O)NC2=NC(C)=CC=C2)=C(C)N1C1=CC=C(C=C1)C(F)(F)F | 2 | A |
| CN1N=C(C(=O)NC2=NC(C)=CC=C2)C2=C1C=CC=C2 | 2 | A |
| ClC1=CC=NC(=C1)C(=O)NC1=CN=CC(OC2=CN=CN=C2)=N1 | 2 | A |
| CC1=NC(C(=O)NC2=NC(C)=CC=C2)=C(C)N1C1=CC=C(F)C=C1 | 2 | A |
| CC1=NC(C(=O)NC2=NC(C)=CC=C2)=C(C)N1C1=CC=C(Cl)C=C1 | 2 | A |
| COC1=CC=C(C=C1)N1C(C)=NC(C(=O)NC2=CC=CC(C)=N2)=C1C | 2 | A |
| CC1=NC(C(=O)NC2=NC(C)=CC=C2)=C(C)N1C1=CC=C(Cl)C(Cl)=C1 | 2 | A |
| CC1=NC(C(=O)NC2=CC=C(F)C(C)=N2)=C(C)N1C1=CC=C(F)C=C1 | 2 | A |
| CC1=CSC(NC(=O)C2=CC(F)=CC(OC3=CN=CN=C3)=C2)=N1 | 2 | A |
| CC1=CSC(NC(=O)C2=CC(Cl)=CC(OC3=CN=CN=C3)=C2)=N1 | 2 | A |
| CC1=NC(C(=O)NC2=CC=CC(=N2)C#N)=C(C)N1C1=CC=C(F)C=C1 | 2 | A |
| CC1=NC(C(=O)NC2=CC=CC(=N2)C(F)(F)F)=C(C)N1C1=CC=C(F)C=C1 | 2 | A |
| CC1=NC(C(=O)NC2=NC(C)=CC=C2)=C(C)N1C1=CC=C(F)C(Cl)=C1 | 2 | A |
| CC1=NC(C(=O)NC2=CC=NC(Cl)=C2)=C(C)N1C1=CC=C(F)C=C1 | 2 | A |
| CN1N=C(C(=O)NC2=NC(C)=CC=C2)C(C)=C1OCC1=CC=C(F)C=C1 | 2 | A |
| CN(C1=CN=CN=C1)C1=CC(F)=CC(=C1)C(=O)NC1=NC(C)=CS1 | 2 | A |
| CC1=NC(C(=O)NC2=CC=CC(F)=N2)=C(C)N1C1=CC=C(F)C=C1 | 2 | A |
| CC1=NC(C(=O)NC2=CC=C(F)C=N2)=C(C)N1C1=CC=C(F)C=C1 | 2 | A |
| CN1N=C(C(=O)NC2=NC(C)=CC=C2)C(C)=C1C1CCCCC1 | 2 | A |
| CN1N=C(C(=O)NC2=NC(C)=CC=C2)C(C)=C1OC1CCCCC1 | 2 | A |
| CN1N=C(C(=O)NC2=NC(C)=CC=C2)C2=C1CCCC2 | 2 | A |
| CN1N=C2CCCCC2=C1C(=O)NC1=NC(C)=CC=C1 | 2 | A |
| CC1=NC(C(=O)NC2=CC=NC(Cl)=N2)=C(C)N1C1=CC=C(F)C=C1 | 2 | A |
| CC1(C)O[C@]1([C@H](O)C1=CC=CC=C1)N1N=NC2=CC=CC=C12 | 2 | I |
| CN1C(Cl)=C[P@](=O)(NC2=CC=C(C)C=C2)N(C)C1=O | 2 | I |
| CC[C@@]1(C)NC(=O)N(\N=C\C2=CC=CC3=C2C=CC=C3)C1=O | 2 | I |
| CC1=C(C)C2=C(S1)N=C(N=C2O)C(=C\C1=CC=CO1)\C#N | 2 | I |
| C1COC2=C(OC1)C=C(NC1=C3C=CSC3=NC=N1)C=C2 | 2 | I |
| COC1=CC=C(C=C1OC)[C@H]1CC2=CC=CC(O)=C2C(=O)O1 | 2 | I |
| C[C@@H](NC1=CC=C2OCCOC2=C1)C1=CC=C2OCOC2=C1 | 2 | I |
| ClC1=CC=C(CSC2=NN3C(=O)NN=C3C=C2)C=C1 | 2 | I |
| CC(C)N1N=C(C)C(NC2CCC3(CC2)OCCO3)=C1C | 2 | I |
| CC1CCN(CC1)C1=NC(=O)C(C#N)=C(N1)C1=CC=CC=C1 | 2 | I |
| O=S(=O)(NC1(CCCCCC1)C#N)N1CCCCCC1 | 2 | I |
| C[C@@H]1CCC[C@@H](NC(=O)N(C)CC2=C(C)ON=C2C)[C@H]1C | 2 | I |
| OC1=CC=CC(\C=C(\C#N)C(=O)C2=CC=C3OCOC3=C2)=C1 | 2 | I |
| C[C@H]1CN(C[C@H](C)O1)C1=[NH+]C(=C(S1)C([O-])=O)C(C)(C)C | 2 | I |
| C[C@@H]1CN(C[C@@H](C)O1)C1=[NH+]C(=C(S1)C([O-])=O)C(C)(C)C | 2 | I |
| C[C@@H](NC1=CC=C2OC(F)(F)OC2=C1)C(=O)N1CCCC1 | 2 | I |
| CCC1=C(OC2=CC=CC=C12)C1=CSC(=N1)C1=NNC=N1 | 2 | I |
| N1C=C(C2=NC(=NO2)C2=CC=CS2)C(=N1)C1=CC=CC=C1 | 2 | I |
| CC(C)C1=NC2=CC(N[C@H]3CCS(=O)(=O)C3)=CC=C2O1 | 2 | I |
| N#CC1=CC=C(C=C1)[C@H]1N[C@@H]2N(N=CC2=N1)C1CCCCC1 | 2 | I |
| COC1=CC=CC=C1N1C(=O)N[C@@](C)(C1=O)C1=CC=CC=C1 | 2 | I |
| CC1=CC(=O)NC(SC[C@H]2COC3(CCCCC3)O2)=N1 | 2 | I |
| CCCN1C(=O)C=C2NN(C(=O)C2=C1C)C1=CC=CC(C)=C1 | 2 | I |
| COC1=CC=CC=C1\N=C/C1=C(O)OC2=CC=CC=C2C1=O | 2 | I |
| CC(=O)OCC1=CC=C2C(=O)C3=CC=CC=C3C(=O)C2=C1O | 2 | I |
| N1C=CC(=N1)C1=CC(=CC=C1)C1=NOC(=N1)C1=CC=CS1 | 2 | I |
| CC[C@@H]1CCC[C@@H](C1)NC(=O)N1CCN(CC1)C(=O)CC | 2 | I |
| FC1=CC=CC(F)=C1\C=C\C(=O)\N=C1/NN=C2C=CC=CN12 | 2 | I |
| O=C(CCNC(=O)N1CCCCCCC1)N1CCCCC1 | 2 | I |
| CC1=CC(NS(=O)(=O)C2=C(C)C(C)=CC(C)=C2C)=NO1 | 2 | I |
| [O-][N+](=O)C1=CC(Br)=C(N[C@@H]2CCC=CC2)N=C1 | 2 | I |
| [O-][N+](=O)C1=CC=C(N[C@@H]2CSC3=CC=CC=C23)C(=C1)C#N | 2 | I |
| O=C(NC[C@H]1COCCO1)N1CCC[C@@H]1C1CCCCC1 | 2 | I |
| C[C@H]1CC(=O)N[C@H](C2CCCCC2)C(=O)N1[C@@H]1CCOC1 | 2 | I |
| CCCC1=NN=C(NC(=O)N2CCC[C@H]3CCC[C@H]23)S1 | 2 | I |
| CN1CC[C@H](C1)NC1=CC(Br)=CC=C1[N+]([O-])=O | 2 | I |
| CC(C)C1=C(SC=C1)C(=O)\N=C1/NN=C2C=C(C)C=CN12 | 2 | I |
| [O-][N+](=O)C1=C(C=C(F)C=C1)N1CCNCC11CCCCC1 | 2 | I |
| CC(C)[C@@H]1CCCN(CC1)C(=O)N1CC[C@H](CC(O)=O)C1 | 2 | I |
| C[C@@H]1CCC[C@@H](C)N1C(=O)NCC(C)(C)N1CCOCC1 | 2 | I |
| CC(C)C1=C(SC(=N1)N1CC[NH+](C)C(C)(C)C1)C([O-])=O | 2 | I |
| CC1=CC=C(O)C(CN2CCC3=CC=C(C=C3C2)[N+]([O-])=O)=C1 | 2 | I |
| CC1(C)CCC[C@H]1NC(=O)C1=CC(F)=C(F)C=C1[N+]([O-])=O | 2 | I |
| CC1=C(Cl)C(=O)C2=CC(=CC(=C2N1)[N+]([O-])=O)C(C)(C)C | 2 | I |
| O=C1OC(=N\C1=C/C1=NNC=C1)C12CC3CC(CC(C3)C1)C2 | 2 | I |
| CC1=CC(\C=N/N2C(=O)C3=CC=CC=C3C2=O)=CC(C)=C1O | 2 | I |
| CNC1=CC=C(\C=C2\CC3=CC=CC=C3C2=O)C=C1[N+]([O-])=O | 2 | I |
| FC1=CC=CC(=C1)C1=NC2=C(CN(CC2)C2=NC=CC=N2)N1 | 2 | I |
| C[C@H]1COCCN1C(=O)NC[C@@H]1CCCO[C@@H]1C(C)(C)C | 2 | I |
| C[C@H]1CCC[C@H](CNC(=O)N2CCC3(CC2)OCCO3)C1 | 2 | I |
| O=C1CN(CC2=CC=CC=C2)C(=O)[C@@H]2CC3=C(CN12)NC1=CC=CC=C31 | 2 | I |
| O=C1NC(=S)C2=C(CCCC2)N1CC1=CC2=C(OCO2)C=C1 | 2 | I |
| O=C1O[C@H]2C=CC(=C[C@H]2C=C1)S(=O)(=O)NC1CCCCC1 | 2 | I |
| ClC1=CC=C(C=C1)C1=NN(CN2CCOCC2)C(=S)N1 | 2 | I |
| COC1=CC=C2[C@@H]3[C@H]4CCCC[C@]4(O)CCN3C(=O)C2=C1OC | 2 | I |
| COC1=CC=C(C=N1)N1C(=S)NC2=C(C(C)=C(C)S2)C1=O | 2 | I |
| CN1C=C(NCC2=CC3=C(OCO3)C=C2Br)C=N1 | 2 | I |
| CN1C=C(NCC2=CC3=C(OCO3)C(Br)=C2)C=N1 | 2 | I |
| CN1C(=S)NN=C1N1CCN(CC1)C1=CC=CC=C1Cl | 2 | I |
| ClC1=CC(=CC=C1C#N)N1C(=S)NN=C1C1=CN=CC=C1 | 2 | I |
| ClC1=C(C=CC(=C1)N1C(=S)NN=C1C1=CC=NC=C1)C#N | 2 | I |
| C[C@H]1CC2=CC=CC=C2N1S(=O)(=O)C1=C(C)NC(=O)S1 | 2 | I |
| O=C1CCCN1C1=CC=C(C=C1)C1=NC2=C(SC=C2)C(=O)N1 | 2 | I |
| COC1=CC=C2[C@@H]3[C@@H]4CCCC[C@]4(O)CCN3C(=O)C2=C1OC | 2 | I |
| O=C(N[C@H]1CCCC[C@H]1N1C(=O)CSC1=O)C1CCCC1 | 2 | I |
| CCN1C=C(C=N1)C1=CSC2=C1C(=O)NC(=N2)[C@H](C)Cl | 2 | I |
| O=C1C=CN=C2SC(N[C@H]3CCSC4=CC=CC=C34)=NN12 | 2 | I |
| FC(F)(F)CNC(=O)N1CCCN(CC1)C1=NC=CS1 | 2 | I |
| C[C@@H]1CN([C@H](C)CO1)C1=C(C=C(Br)C=[NH+]1)C([O-])=O | 2 | I |
| CN([C@H]1CCCN(C)C1)C1=NNC(=S)N1C1CCCCC1 | 2 | I |
| C[C@H]1CCC[C@H](C)N1CC(=O)NC1CCC2(CC1)OCCO2 | 2 | I |
| O=C1NC(=O)C2=CC=CC=C2\C1=C/C1=CC=C(C=C1)N1C=CC=N1 | 2 | I |
| CC[C@@H]1CN(CCO1)S(=O)(=O)C1=CC=C2CCCNC2=C1 | 2 | I |
| C[C@@H]1C[C@H]2CNC[C@@H]2N1S(=O)(=O)C1=CC=C(F)C(=C1)C#N | 2 | I |
| CCN1CCN(CC1)C(=O)N[C@@H]1CC(C)(C)CC2=C1C=C(C)O2 | 2 | I |
| C1NCC2=C1N=C(N=C2N1C[C@H]2CC=CC[C@H]2C1)C1=CC=NC=C1 | 2 | I |
| O=C1NC2(CCN(CC3=CC=CC=N3)CC2)OC2=CC=CC=C12 | 2 | I |
| O[C@H]1CCCN(C1)C(=O)\C=C\C1=CC2=C(OCO2)C(Cl)=C1 | 2 | I |
| CC1=CC(C)=C(N1)C(=O)N1CCC[C@@H](C1)C(=O)N1CCCCC1 | 2 | I |
| C[C@@H](NC(=O)C1CCN(CC1)C(=O)N(C)C)C1CCCCC1 | 2 | I |
| C[C@H]1CCN([C@H](C)C1)C(=O)NCC1(CCCC1)C(=O)N(C)C | 2 | I |
| C[C@@H]1CCN([C@@H](C)C1)C(=O)NCC1(CCCC1)C(=O)N(C)C | 2 | I |
| C[C@@H]1N(CCNC1=O)S(=O)(=O)C1=C(C)C(C)=CC(C)=C1C | 2 | I |
| O=C1NC2=CC=CC=C2C(=O)N1CC1=NC2=CC=CC=C2S1 | 2 | I |
| C[C@@H]1CN(CCO1)C1CCN(CC1)C(=O)NCC(F)(F)F | 2 | I |
| C[C@H]1N(C2=CC=CC=C2NC1=O)S(=O)(=O)C1=CC=C(F)C=C1 | 2 | I |
| CC[C@H]1NC(=O)CCN(C2=CC(C)=C(Cl)C=C2OC)C1=O | 2 | I |
| C[C@@H]1N(CC2=CC=CC=C2NC1=O)S(=O)(=O)CC(C)(C)C | 2 | I |
| O=C(NCC1=CC=NC2=C1C=CC=C2)N1CCN(CC#C)CC1 | 2 | I |
| O=C(N[C@H]1[C@@H]2CCO[C@@H]2C11CCCC1)N1CCN(CC#C)CC1 | 2 | I |
| C[C@@H](N1CCCC2(C1)OCCO2)C(=O)N[C@H]1CCCC[C@@H]1C | 2 | I |
| CNC1=NC(=NC(C(C)C)=C1Br)C1=CN=CN1C | 2 | I |
| CC1=NOC(CN2C(=S)NC3=CC(Br)=CN=C23)=C1 | 2 | I |
| BrC1=C(NN(C1=O)C1=CC=NC=N1)C1=CC=CC=C1 | 2 | I |
| CC(C)(C)C1=CC(=NN1)C(=O)N1CCS[C@@H]2COCC[C@H]12 | 2 | I |
| [O-]C(=O)C1=C[NH+]=CC(=C1)C1=CC=C(C=C1)C(=O)N1CCCCC1 | 2 | I |
| CC[C@@H]1CO[C@@]2(C1)CCCN(C2)C(=O)C1=CC=C2NC=NC2=C1 | 2 | I |
| BrC1=CN2C=C(CSC3=NNN=C3)N=C2C=C1 | 2 | I |
| CCCC1=C(C)SC(=C1)C(=O)N1CC(=O)NC(=O)C1(C)C | 2 | I |
| FC(F)(F)C1=CC(=NN1)C(=O)N1CCC[C@@]2(CCCO2)CC1 | 2 | I |
| BrC1=CC=C(C=C1)N1NC(=O)\C(=C/C2=CC=CO2)C1=O | 2 | I |
| CN(C)N\C=C1/C(=O)N(C(=O)C2=CC=CC=C12)C1=CC=C(C)C=C1 | 2 | I |
| O=C1NC(=S)N(C2CCCCC2)C(=O)[C@@H]1\C=N\C1CCCC1 | 2 | I |
| O=C(N1CCC[C@H]1C1=CC=C2OCCOC2=C1)C1=CNC2=CC=CC=C12 | 2 | I |
| O=C1S\C(=C/C2=NC3=CC=CC=C3N2)C(=O)N1C1=CC=CC=C1 | 2 | I |
| CC[C@@H]1N(CCC2=C1C=CS2)C(=O)C1=CC=C2NC(=O)COC2=C1 | 2 | I |
| CC1(C)[C@@H]2CCC11[C@@H](C2)N([C@@H]2[C@H](NC2=O)C2=CC=CC=C2)S1(=O)=O | 2 | I |
| OC[C@]12O[C@H](C=C1)[C@@H]1[C@@H]2C(=O)N(C1=O)C1=CC(Cl)=C(Cl)C=C1 | 2 | I |
| [O-]C1=NC(=S)S\C1=C/C1=CC2=CC=CC=C2[NH+]=C1N1CCOCC1 | 2 | I |
| O=C(NC1CCN(CC1)C(=O)C1CC1)N1CCC2=C(C1)C=CS2 | 2 | I |
| O=C1NC2=CC=CC=C2C(=O)N1CC1=CC=C(C=C1)N1CCCC1 | 2 | I |
| CC1(C)[C@H]2CC[C@]1(C)[C@@H](C2)N1C[C@]23O[C@H](C=C2)[C@@H]([C@@H]3C1=O)C(O)=O | 2 | I |
| CN1C(=O)N(C)C2=C1C=C(Br)C(=C2)[C@@H](O)C1=C(C)C=CO1 | 2 | I |
| [O-]\C=C1\CCCC[NH+]1CC1=CC(=O)N2C=C(Br)C=CC2=N1 | 2 | I |
| CC1=CC(=C(C)S1)S(=O)(=O)N1CC(=O)NC2=C1C=CC=C2 | 2 | I |
| C[C@H]1CC[C@@H]2[C@H](CCCN2C(=O)C2=CC=CC(=C2)N2CCNC2=O)C1 | 2 | I |
| C[C@@H]1CN(C[C@@H](O1)C1=CC=CC=C1)C(=O)C1=CC=C2NC=NC2=C1 | 2 | I |
| O=C(N1CCC(CC1)N1CCNC1=O)C1=CC2=CC=CC=C2S1 | 2 | I |
| CCN1C=C(C=N1)[C@H](O)C1=CC2=C(OCO2)C(Br)=C1 | 2 | I |
| C[C@H](N1C[C@@H](C)O[C@H](C)C1)C1=NC2=C(C(C)=C(C)S2)C(=O)N1 | 2 | I |
| C[C@@H]1CN(CC2=NC3=NC=C(Br)C=C3N2)CC(C)(C)O1 | 2 | I |
| [O-]C1=NC(=NC2=C1C[NH+](CC1=CSC=C1)CC2)C1=CN=CC=C1 | 2 | I |
| O=C(OC1=CC=C2NC(=O)CCC2=C1)C1=CC2=CC=CC=C2OC1 | 2 | I |
| C[C@@H]1CSC2=CC=CC=C2N1S(=O)(=O)C1=C(C)NN=C1C | 2 | I |
| C[C@@H]1CCN([C@H](C)C1)C(=O)N[C@@H]1CCN(CC(F)(F)F)C1=O | 2 | I |
| C[C@H]1CCN([C@H](C)C1)C(=O)N[C@@H]1CCN(CC(F)(F)F)C1=O | 2 | I |
| FC(F)(F)[C@@H]1CCC[C@@H](C1)NC(=O)N1CCS(=O)(=O)CC1 | 2 | I |
| CCC1=NO[C@H](C1)C1=NC2=C(C3=C(CSCC3)S2)C(=O)N1 | 2 | I |
| O=C(CN1C(=O)NC2=CC=CC=C12)N1CCC[C@H]1C1=CC=CC=C1 | 2 | I |
| COC1=CC(\C=C/[N+]([O-])=O)=CC(I)=C1O | 2 | I |
| CC(C)N1C(=O)NN=C1SC1=NC(Br)=CS1 | 2 | I |
| CN1CCN(C2=C1C=CC=C2)S(=O)(=O)C1=CC=C(CO)S1 | 2 | I |
| O=C([C@H]1CCCN1C1CCOCC1)N1CCC2=C(C1)NC1=CC=CC=C21 | 2 | I |
| CN[C@@H]1COC[C@@H]1C1=NC(=NO1)C1=CC=C(Br)C=C1 | 2 | I |
| BrC1=CC=CC=C1C1=NOC(C[C@@H]2CNCCO2)=N1 | 2 | I |
| C[C@@H]1CN(CC[C@]1(C)O)C(=O)C1(CCCCC1)N1CCOCC1 | 2 | I |
| CN(CC1=C(C)N=CS1)C1=CC2=C(C=C1Cl)C(=O)C(=O)N2 | 2 | I |
| C[C@H](CN(C)C1=CC2=C(C=C1Br)C(=O)C(=O)N2)C#N | 2 | I |
| C[NH+](C)C1CCN(CC1)C1=CC2=C(C=C1Br)C(=O)C([O-])=N2 | 2 | I |
| C[C@@H](NC1=NC(Br)=CN2C=CN=C12)C1=CC=C(C)O1 | 2 | I |
| CN1CCCC[C@H]1CNC1=NC(Br)=CN2C=CN=C12 | 2 | I |
| C1CS[C@@H](CS1)C1=NOC(=N1)[C@H]1CNC2=CC=CC=C2O1 | 2 | I |
| O=C1CN(CC2=CC=CC=C2)[C@H](N1)C1=CC=CC2=C1OCCCO2 | 2 | I |
| CC(C)NC(=O)N1CC[C@]2(C1)CN(C(=O)C2)C1=CC=C(C)S1 | 2 | I |
| CN1C=C(C=N1)N1CC[C@H](C1)NC1=CC(Br)=CN=C1 | 2 | I |
| OC1=NC2=C(SC(=N2)N2CCOCC2)[C@@H](C1)C1=CC=CS1 | 2 | I |
| OC1=NC2=C(N=CN2C2=CC=CN=C2)[C@@H](C1)C1=CC=CC=C1Cl | 2 | I |
| C[C@]1(NC(=O)N(CC2=CC=C(Cl)N=C2)C1=O)C1=CC=CS1 | 2 | I |
| COC1=CC=C(Br)C=C1[C@@H]1CC(O)=NC2=C1C=NN2C | 2 | I |
| COC1=CC(=CC2=C1OCCO2)[C@H]1SCC(O)=NC2=C1SC=C2 | 2 | I |
| CC1=CC=C(C=C1)[C@@]1(C)NC(=O)N(CC(=O)N2CCCC2)C1=O | 2 | I |
| CN1C(=O)NC(=O)\C(=C\C2=C(C)N(C(C)=C2)C2=CC=CC=C2C)C1=O | 2 | I |
| CC1CCN(CC1)C(=O)CN1C(=O)N[C@]2(CCC3=C2C=CC=C3)C1=O | 2 | I |
| O=C(CN1C(=O)NC2(CCCC2)C1=O)N1CCC[C@H]2CCCC[C@@H]12 | 2 | I |
| C[C@@H]1CCCCN1C1=C(C(=O)C1=O)C1=CC=C2NC(=O)COC2=C1 | 2 | I |
| O=C(CC1=NC2=C(C3=C(CCCCC3)S2)C(=O)N1)N1CCOCC1 | 2 | I |
| C[C@@H](SC1=C(C#N)C(C)=C(C)C(C)=N1)C(=O)N1CCNC1=O | 2 | I |
| O=C1N(NC2=CC(=O)N(CC3=CC=CN=C3)C=C12)C1=CC=CC=C1 | 2 | I |
| COC(=O)[C@]1(C)N[C@@H]([C@@H]2[C@@H]1C(=O)N(C)C2=O)C1=CC=CC=C1C | 2 | I |
| CC1=NC2=C(C[C@@H]3CC[C@H](C2)N3CC2=CC=C3OCCOC3=C2)C(=O)N1 | 2 | I |
| COC1=CC(=CC=C1O)[C@H]1SC=C2SC3=NN=C(C)N3N12 | 2 | I |
| O=C1N[C@]2(CCOC3=CC=CC=C23)C(=O)N1C[C@@H]1CCCCO1 | 2 | I |
| O=C1N(CC2=CC=CC=C2)C2=NC=CC=C2N=C1N1CCNCC1 | 2 | I |
| O=C1N[C@]2(CCCCC3=CC=CC=C23)C(=O)N1CN1CCOCC1 | 2 | I |
| CCN1C(=O)C2=CC=CC3=CC(=CC(C1=O)=C23)S(=O)(=O)NC | 2 | I |
| C[C@@H]1N(CCN2C(C)=CC=C12)S(=O)(=O)C1=C(C)NC(=O)S1 | 2 | I |
| C[C@H]1CCCCN1C(=O)CN1C(=O)N[C@@]2(CCCC[C@H]2C)C1=O | 2 | I |
| CC1=C(N2CCN(CC2)C(=O)C2=CC=C3N=CSC3=C2)C(C)=NN1 | 2 | I |
| C[C@@H]1CN(C[C@@H](C)O1)S(=O)(=O)C1=CC=C2NC(=O)CCC2=C1 | 2 | I |
| O=C1N[C@@]2(CCOC3=CC=CC=C23)C(=O)N1CCC1=CC=NC=C1 | 2 | I |
| C[C@@H](N1C(=O)NC2(CCCC2)C1=O)C(=O)N1C[C@H](C)C[C@@H](C)C1 | 2 | I |
| CC1=NN2C(S1)=NC(CN1CCC3=CC=C(O)C=C3CC1)=CC2=O | 2 | I |
| CCC1=CC=C(O1)[C@H]1CC(=O)NC2=C1C=C1N(C)C(=O)COC1=C2 | 2 | I |
| O=C(N1CCC[C@@H](C1)C1=CC(=O)NC=N1)C1=CC2=C(OCCC2)C=C1 | 2 | I |
| CC1=CN\C(C=C1)=N/S(=O)(=O)[C@@H]1CS(=O)(=O)C2=C1C=CC=C2 | 2 | I |
| C1CCC(C1)C1=NC(=NC=C1)N1CCO[C@]2(C1)CNCCOC2 | 2 | I |
| CN1C(Cl)=C(Cl)C=C1C(=O)N1CCC(CC1)N1CCNC1=O | 2 | I |
| C[C@H](N1C=NC2=C(C3=C(CCCCC3)S2)C1=O)C1=NC=NN1 | 2 | I |
| O[C@@H]1CCCN(C1)C1=NC2=CC=CC=C2N1C1=C(C=CC=N1)C#N | 2 | I |
| CC1=CC(=O)NC(SCC(=O)N2C(=O)CC3=CC=CC=C23)=N1 | 2 | I |
| CC1=C2C(NN(C2=O)C2=CC=CC=C2)=CC(=O)N1C1=CC=CC=N1 | 2 | I |
| O[C@H]1COCCN(C1)C1CCN(CC1)C1=CC2=C(OCO2)C=C1 | 2 | I |
| C[C@H]1CC2=CC(=CC=C2N1C(C)=O)S(=O)(=O)\N=C1/NC(C)=CC=C1 | 2 | I |
| C[C@H]1CCC[C@@H]1NC(=O)N1CCC(CC1)C(=O)N1CCOCC1 | 2 | I |
| [O-]\C(=N/S(=O)(=O)C1=CC=C[NH+]=C1)C1=CC2=CC=CC=C2OC1 | 2 | I |
| C[C@@H]1CCC[C@H](C1)NC(=O)N1CCN(CC1)C(=O)[C@@H]1CCCO1 | 2 | I |
| ClC1=CC(=CNC1=O)S(=O)(=O)N1CCOC2=CC=CC=C12 | 2 | I |
| C[C@@H]1CC(=O)NCC(=O)N1CC1=C(Br)C(C)=NN1C | 2 | I |
| C[C@H](NC(=O)N1CCN(CC#C)CC1)C(=O)N1CCCC[C@H]1C | 2 | I |
| C[C@H]1CN(C[C@@H]1N(C)C)C(=O)C1=CC=CC2=C1O[C@H](C)C(=O)N2 | 2 | I |
| COC1=CC=C2NC(=O)C[C@H](C(=O)N3CCC4(CCCO4)CC3)C2=C1 | 2 | I |
| CN1CCC2=NNC(=C2C1)C1=NC=NN1C1=CC=C(F)C(F)=C1 | 2 | I |
| O=C(N1C[C@@H](N2C=CC=N2)C2=CC=CC=C2C1)C1=CC=C2N=CNC2=C1 | 2 | I |
| ClC1=CC2=C(OCO2)C=C1CN1CC2(CCNCC2)OC1=O | 2 | I |
| O=C(N1CCO[C@]2(CCCN(C2)C2=CC=CC=C2)C1)C1=CN=CN1 | 2 | I |
| CC1=CC=C(Br)C=C1S(=O)(=O)N1CC(=O)NC(=O)C1 | 2 | I |
| C[C@@H]1N(CC(=O)NC1=O)S(=O)(=O)C1=C(Cl)SC(Cl)=C1 | 2 | I |
| OC1=CC=CC(\C=C2/CC(=O)N(C2=O)C2=CC=C3OCOC3=C2)=C1 | 2 | I |
| CC1=C([C@H]2SCC(O)=NC3=C2C(C)=NN3C2=CC=CC=C2)C(C)=NO1 | 2 | I |
| O=C1N(NC2=NC=C3C(=O)C[C@H](CC3=C12)C1=CC=CO1)C1=CC=CC=C1 | 2 | I |
| CCN1N=CC([C@@H]2C[C@H](N3N=CC(C#N)=C3N2)C(F)(F)F)=C1C | 2 | I |
| COC1=CC=C(C=C1)N1C(=O)NC(=O)\C(=C/C2=CC=CS2)C1=O | 2 | I |
| C[C@@H](NC1=CC=C2OCCOC2=C1)C(=O)N1C[C@@H](C)O[C@H](C)C1 | 2 | I |
| O=C(CN1C(=CC2=C1N=CC=C2)C1=CC=CS1)N1CCNC(=O)C1 | 2 | I |
| COC1=CC2=C(C=C1OC)C1=NC(=O)[C@H](C(C)C)N1C(=S)N2 | 2 | I |
| CN1CCC2=C(C1)C1=NN=CN1C(NCC1=CC=CC=C1)=C2C#N | 2 | I |
| CN1C(=O)N(C)C(N[C@@H]2CCC[C@H](C2)C(F)(F)F)=C(C#N)C1=O | 2 | I |
| CN1C(=O)N(C)C(N[C@H]2CCC[C@H](C2)C(F)(F)F)=C(C#N)C1=O | 2 | I |
| CC(C)NC(=O)[C@H]1CCCN(C1)C1=NC=NC2=C1C(C)=C(C)O2 | 2 | I |
| O=C1N(CC2=NNC(=S)N2C2=CC=CC=C2)N=CC2=CC=CC=C12 | 2 | I |
| COC1=CC=CC(=C1)[C@H]1CC(=O)NC2=NC(SC)=NC(=O)[C@@H]12 | 2 | I |
| COC1=CC=CC(=C1)[C@@H]1CC(=O)NC2=NC(SC)=NC(=O)[C@H]12 | 2 | I |
| COC1=CC=CC(=C1)[C@H]1CC(=O)NC2=NC(SC)=NC(=O)[C@H]12 | 2 | I |
| CC[C@@H]1CN(CCO1)S(=O)(=O)C1=CC=C2NC(=O)CCC2=C1 | 2 | I |
| CC1=C(OC2=C1C(=O)CC(C)(C)C2)C(=O)NCC(=O)N1CCCC1 | 2 | I |
| C[C@@H]1N(CCN(CC2=CC(C)=CC=C2)C1=O)C(=O)C1=C([O-])C=CC=[NH+]1 | 2 | I |
| CC1=NN(C[C@@H](O)C2=CC=C3OCCOC3=C2)C(=O)C2=CC=CC=C12 | 2 | I |
| ClC1=CC=C(C=C1)C1=C[C@@H](N=N1)[C@H]1NC(=NO1)C1=CN=CC=C1 | 2 | I |
| CC1=CN2C(=O)C=C(CN3CCC(CC3)C3=NNC=C3)N=C2C=C1 | 2 | I |
| O[C@@H]1CN(CC[C@H]1C1=CC=C2OCOC2=C1)C(=O)CC1=CN=CC=C1 | 2 | I |
| C1CN(CCN1)C1=NC(=NC(=C1)C1=CC=CC=C1)C1=CN=CC=N1 | 2 | I |
| O=C(C[C@H]1CCCO1)N1CCC(CC1)N1C(=O)NC2=CC=CC=C12 | 2 | I |
| CC1(CCN(CC1)C1=CC=CC=N1)C1=NNC=C1S(C)(=O)=O | 2 | I |
| CC[C@H]1CN2CCC[C@@H]2CN1S(=O)(=O)C1=CNC2=C1C=CC=N2 | 2 | I |
| CN1CCCN(CCN2C(=O)NC(C)(C)C2=O)C2=C1C=CC(F)=C2 | 2 | I |
| COC1=CC=C(C=C1O)C(=O)N1CCN(C(=O)C1)C1=CC=C(C)C=C1 | 2 | I |
| C[C@@H]1CN(CCO1)C(=O)N[C@@H]1CN(CC2=CC=CC=C2)C(=O)C1 | 2 | I |
| C[C@H](NC(=O)N1CCC(CC1)N1CCO[C@@H](C)C1)[C@@H]1CCCO1 | 2 | I |
| CC[C@H]1CO[C@@H](C)CN1S(=O)(=O)C1=CNC(=O)C(Cl)=C1 | 2 | I |
| CNC1=C(Cl)C=C(C=N1)S(=O)(=O)N1CCO[C@@H]2CCC[C@H]12 | 2 | I |
| CC1=CC(=NN1)[C@@H]1CCCN(CC2=CC(=O)N3C=CSC3=N2)C1 | 2 | I |
| C[C@H]1CN(CC(C)(C)O1)S(=O)(=O)C1=C(C)NN=C1CCl | 2 | I |
| ClCC1=CNN=C1S(=O)(=O)N1CCO[C@@H]2CCCC[C@H]12 | 2 | I |
| CN1N=C(C)C(Br)=C1CSC1=NC=NC2=C1NC=N2 | 2 | I |
| CCC1=NNC(=N1)[C@H]1CN(CCO1)C(=O)C1=CC2=C(CCC2)S1 | 2 | I |
| C[C@H]1CC(=O)N(CC(=O)N\N=C2\C[C@H]3CC[C@]2(C)C3(C)C)C1=O | 2 | I |
| O=C1N(CC2CCC2)CCC[C@@]11CCN(C1)C1=NC=NC2=C1C=CN2 | 2 | I |
| OC1=CC=CC(=C1)C(=O)N1CCN(CC2=CC=CC3=C2OCO3)CC1 | 2 | I |
| CN1CCO[C@H](CNC2=NN3C=C(Br)C=CC3=N2)C1 | 2 | I |
| CN[C@H]1COC[C@@H]1C1=NC(=NO1)C1=CC(Br)=CN=C1 | 2 | I |
| O=S1(=O)C[C@H](NC2CCN(CC2)C2=NC3=CC=CC=C3O2)C=C1 | 2 | I |
| CCC1=NNC(=O)C(C(=O)N2CCOC3(CCCC3)C2)=C1CC | 2 | I |
| C[C@@H]1CN(CCN1C1=CC=C(C=[NH+]1)C([O-])=O)C(=O)[C@@H]1CC1(C)C | 2 | I |
| O=C1NC2(CCCC2)C(=O)N1CCN1CCO[C@@H]2CCCC[C@@H]12 | 2 | I |
| CN1C(=O)[C@](C)(OC2=C1N=CC=C2)C(=O)NCC1=CC=CC=C1C | 2 | I |
| CC1=CC=C(N=N1)N1CC[C@]2(C[C@@H](CO2)NC2=C(F)C=CC=N2)C1 | 2 | I |
| C\C(=C/C(=O)N1CCN(CC1)C(=O)C1=CC=C2N=CNC2=C1)C1CC1 | 2 | I |
| CC1=NNC(=N1)[C@H]1CCCN1S(=O)(=O)C1=CC=C(Cl)S1 | 2 | I |
| CC1=CC(=CC=C1[N+]([O-])=O)C(=O)N1CC[C@@](O)(C1)C(F)(F)F | 2 | I |
| N#CC1=C(N=CC=C1)N1CCN(CC2=CC3=C(N2)N=CC=C3)CC1 | 2 | I |
| C[C@H]([C@@H]1SC2=CC=CC=C2NC1=O)C(=O)N1CCC2(CC1)OCCO2 | 2 | I |
| C[C@@]12C[C@@H]([C@H](C3=NN=C(O3)C3=CC=CC=C3)C(=O)N1)C1=CC=CC=C1O2 | 2 | I |
| COC1=CC=CC=C1[C@@H]1CC(=O)NC2=C1C(=O)N=C1N2C=CC=C1C | 2 | I |
| C\N=C\[C@H]1C(=O)NC(=O)N(C1=O)C1=C(Br)C=C(C)C(C)=C1 | 2 | I |
| CC1CCC2(CC1)NC(=O)N(CC(=O)N1CCC[C@H]3CCCC[C@H]13)C2=O | 2 | I |
| ClC1=C(CN2C(=O)N[C@]3(CCCC4=C3C=CS4)C2=O)N=NS1 | 2 | I |
| FC1=CC=CC=C1N1CCN(CC1)C(=O)N[C@@H]1CCS(=O)(=O)C1 | 2 | I |
| CC1=C(C)C2=C(S1)N=C(N=C2N1CCNC(=O)CC1)C1=CC=CN=C1 | 2 | I |
| FC1=CC=CC(=C1)[C@@H]1N2N=CN=C2NC2=C1C(=O)C[C@@H](C2)C1=CC=CO1 | 2 | I |
| CC1CCN(CC1)C(=O)C1=CC=C2C(=O)N(C(=O)NC2=C1)C1=CC=CC=C1 | 2 | I |
| O=C([C@@H]1CCC2=CC=CC=C2O1)N1CCC2=C(NC=N2)[C@H]1C1=CC=CC=N1 | 2 | I |
| O=C(CN1C(=O)N[C@]2(CCCCC3=CC=CC=C23)C1=O)N1CCCCC1 | 2 | I |
| O=C(CN1C(=O)CSC2=CC=CC=C12)N1CC(=O)NC2=CC=CC=C12 | 2 | I |
| O=C(N1CCC[C@@H]1C1=CC2=C(OCCCO2)C=C1)C1=CC=C2NC=NC2=C1 | 2 | I |
| C[C@H]1CC2=CC=CC=C2N1C(=O)C1=NN(C(=O)CN1)C1=CC=CC=C1 | 2 | I |
| COC(=O)[C@@H]1SC2=C(SC(=O)N2)[C@H]2[C@H]1C(=O)OC1=CC=CC=C21 | 2 | I |
| CN(C)C1=NC2=CC(C)=CC=C2C=C1C=C1N(C(C)=O)C(=S)NC1=O | 2 | I |
| OC1=CC=CC=C1N1CCN(CC1)C(=O)[C@@H]1COC2=CC=CC=C2O1 | 2 | I |
| BrC1=CN=N[C@@H]1[C@@H]1NN2C(S1)=NN=C2C1=CC=CS1 | 2 | I |
| CC1CCC2(CC1)NC(=O)N(CC(=O)N1C[C@@H](C)C[C@H](C)C1)C2=O | 2 | I |
| C[C@@H]1C[C@@H](C)CN(C1)C(=O)NC1CCN(CC1)C(=O)C1=COC=C1 | 2 | I |
| [O-]C1=C2[C@H](C3=CC=CC=C3)C(C#N)=C([O-])N=C2[N-][NH+]1C1=CC=CC=C1 | 2 | I |
| C[C@H]1C[C@@H](C)CN(C1)C(=O)CN1C(=O)N[C@]2(CCC3=C2C=CC=C3)C1=O | 2 | I |
| CN1C(=O)N=C2N(C3=CC=C(C=C3)[NH+](C)C)C3=CC=CC=C3C=C2C1=O | 2 | I |
| CN1CCN(CC1)C1=NC2=NC=CC(C3=CSC=C3)=C2C(=O)N1 | 2 | I |
| O=C(N1CCC[C@H](C1)[C@@H]1NN=C2C=CC=CN12)C1=CC2=CC=N[C@@H]2C=C1 | 2 | I |
| ClC1=CC=CC=C1C1=C2C(SC1)=NC(=NC2=O)C1=NNC=N1 | 2 | I |
| CC1=NC=C(CC(=O)N2CCC[C@@]3(CC2)OC2=CC=CC=C2C=C3)C(=O)N1 | 2 | I |
| CC(C)C(=O)N1CCN(CC1)C(=O)C1=CC=C2CC(C)(C)OC2=C1O | 2 | I |
| OC1=CC2=C(C=C1)C(=O)\C(CC2)=C\C1=CN=C(N=C1)N1CCOCC1 | 2 | I |
| CC(=O)N1CCCC2=CC(=CC=C12)S(=O)(=O)\N=C1/NC=C(C)C=C1 | 2 | I |
| CS(=O)(=O)\N=C1/NC2=CC=C(C=C2S1)C(=O)N1CCSCC1 | 2 | I |
| C[C@H]1CCC[C@H](C)N1C(=O)CC1=C(C)C2=C(N=C1C)N(C)NC2=O | 2 | I |
| COC1=C2C(=O)[C@]3(OC2=C(Cl)C(OC)=C1)[C@H](C)CC(O)=CC3=O | 2 | I |
| C[C@H]1CN(C(=O)NC2CCN(CC2)C(=O)C2=COC=C2)C2=C1C=CC=C2 | 2 | I |
| O=C1N(CC2=CN3C=CC=CC3=N2)C=NC2=C1C1=C(CNCC1)S2 | 2 | I |
| IC1=CC(=CS1)C1=NC(=NO1)C1=NC=NN1 | 2 | I |
| CN1CCN(CC1)C1=NC=C(\C=C2/CCC3=CC(O)=CC=C3C2=O)C=N1 | 2 | I |
| CN1N=C(C)C(Br)=C1CN1C(=O)NC2(CCCC2)C1=O | 2 | I |
| C[C@@H]1CC(=O)NC(C)(C)C(=O)N1CC1=C(Br)C(C)=NN1C | 2 | I |
| C[C@@]12C[C@@H](CC(C)(C)C1)N(C2)C(=O)C1=CN=C(NC1=O)C1=CC=CC=N1 | 2 | I |
| O=C([C@@H]1CCC(=O)N(C1)C1CCCCCC1)N1CCC2=NNC=C2C1 | 2 | I |
| CN1C[C@@]2(CCCN(CC3=CC(=O)C4=CC(C)=CC=C4N3)CC2)OC1=O | 2 | I |
| CS(=O)(=O)N1CCC2=C(N=CN2)C11CCN(CC1)C1CCCC1 | 2 | I |
| O=C(N1CCO[C@@]2(CCCN(C2)C2=CC=CC(=C2)C#N)C1)C1=CC=CN1 | 2 | I |
| ClC1=C(C=NNC1=O)N1CCC[C@H](C1)C1=NC2=CC=CC=C2O1 | 2 | I |
| CC1=NN2C(S1)=NC(=O)\C(=C/C1=CC=CN1C1=CC(C)=CC(C)=C1)C2=N | 2 | I |
| CN1C=CC=C1\C=C1/C(=O)NC(=O)N(C2=CC=C3C=CC=CC3=C2)C1=O | 2 | I |
| C[C@H]1CCCN(C1)C(=O)CN1C(=O)N[C@]2(CCC3=CC=CC=C3C2)C1=O | 2 | I |
| CC(C)(C)N1N=CC2=C1N=C(O)C[C@@]21C(=O)N(CC#C)C2=CC=CC=C12 | 2 | I |
| COC1=CC=C(C=C1O)[C@H]1SC[C@@H]2N1C(=S)N(C2=O)C1=CC=CC=C1 | 2 | I |
| COC1=CC=C(C=C1)[C@@H]1C[C@@H]([C@@H]2[C@H](O)CCCC2=O)C2=C(CCCC2=O)O1 | 2 | I |
| FC(F)(F)[C@@]1(NC2=C(C#N)C3=C(CCCC3)S2)N=C2SCCN2C1=O | 2 | I |
| COC1=CC=C(Br)C=C1\C=C1\NC(=O)N(C1=O)C1=CC=CC=C1 | 2 | I |
| O=C(CN1[C@H](C2=CNC3=CC=CC=C23)C2=CC=CC=C2C1=O)N1CCCC1 | 2 | I |
| COC1=CC([C@@H]2CC(=O)NC3=C2C(=O)CCC3)=C(OC)C=C1Br | 2 | I |
| FC1=C(F)C=C(NS(=O)(=O)C2=CC3=C4N(CCC3)C(=O)CCC4=C2)C=C1 | 2 | I |
| CC1=CC=C(C=C1)[C@H]1NC(=S)N(C2CC2)C(C)=C1C(=O)N1CCOCC1 | 2 | I |
| O=C1CSC2=CC=C(C=C2N1)S(=O)(=O)N1CCC(=CC1)C1=CC=CC=C1 | 2 | I |
| CC(=O)C1=CC=C(C=C1)C(=O)N1CCC2=C(C1)C(=NN2)C1=CC=CC=C1F | 2 | I |
| COC1=CC=C2NC(=O)\C(=C3/SC(=S)N(CC4=CC=CC=C4)C3=O)C2=C1 | 2 | I |
| ClC1=CC=CC=C1N1C(=S)NC(=O)\C(=C/C=C/C2=CC=CO2)C1=O | 2 | I |
| O=C1N(C[C@H]2CCCO2)[C@@H](NC2=CC=CC=C12)C1=CC2=CC=CC=C2OC1 | 2 | I |
| C[C@H]1CC(=O)NC2=CC(=CC=C2S1)S(=O)(=O)N(C)C1CCCCC1 | 2 | I |
| CN1C(=O)CC2=CC(NS(=O)(=O)C3=CC=C(Br)C=C3)=CC=C12 | 2 | I |
| CN1CCN(CC2=C(O)C=CC3=C2O\C(=C\C2=CC=C(F)C=C2)C3=O)CC1 | 2 | I |
| N=C1S[C@H]2CS(=O)(=O)C[C@H]2N1\N=C\C1=CC=C(C=C1)C1=CC=CC=C1 | 2 | I |
| C[C@@H]1C[C@@H](CC(C)(C)C1)NC(=O)C[C@@H]1CSC2=NC3=C(CCC3)C(=O)N12 | 2 | I |
| CN1[C@@H](C(C(=O)C2=CC=C(C)O2)=C(O)C1=O)C1=CC=CC(Br)=C1 | 2 | I |
| CC1=CN2C(S1)=NC(=O)\C(=C/C1=CC=C(O1)SC1CCCCC1)C2=N | 2 | I |
| CC1=CSC2=NC(CN3CCC(=CC3)C3=CNC4=C3C=CC=C4)=CC(=O)N12 | 2 | I |
| BrC1=C(C=C2OCCOC2=C1)S(=O)(=O)NC1CCCCC1 | 2 | I |
| BrC1=CC(=C(Br)C=C1)S(=O)(=O)NC1=NOC=C1 | 2 | I |
| C[C@H]1OC2=CC=CC=C2O[C@@H]1C(=O)N1CCC(CC1)[C@H](O)C1=CC=CC=C1 | 2 | I |
| C[C@@H]1CCC2=C(C1)SC(NC(=O)[C@H]1CN(C(=O)C1)C1=CC=CC=C1)=C2C#N | 2 | I |
| CC1=CC=C(O1)[C@@]1(C)NC(=O)N(CC2=CC=CC=C2Br)C1=O | 2 | I |
| O=C1NC(CSC2=CC3=C(OCCCO3)C=C2)=NC2=C1C1=C(CCC1)S2 | 2 | I |
| CC1=C(C)C2=C(S1)N=C(C)N=C2SCC1=NC2=C(SC=C2)C(=O)N1 | 2 | I |
| C[C@]1(NC(=O)N(CC2=CC=C(C=C2)C#N)C1=O)C1=CC=C(Br)C=C1 | 2 | I |
| C[C@H](NC(=O)N1[C@H](C)CC2=CC=CC=C12)C1=CC=C(C=C1)S(C)(=O)=O | 2 | I |
| C(N1CCN(CC1)[C@@H]1CCC2=CC=CC=C2C1)C1=NN2CCCNCC2=C1 | 2 | I |
| O[C@@H]1CC2=CC=CC=C2C11CCN(CC2=CN(N=N2)C2=CC=CC=C2)CC1 | 2 | I |
| C[C@H]1OC2=CC=CC=C2O[C@@H]1C(=O)NCC#CCN1CCC2=CC=CC=C2C1 | 2 | I |
| OC1=CC=C(C=C1)N1CCN(CC(=O)N2CCSC3=CC=CC=C23)CC1 | 2 | I |
| CN1C=[NH+]C2=C1C=CC([N-]S(=O)(=O)C1=CC=C(Br)C=C1)=C2 | 2 | I |
| O=C(CSC1=NC2=CC=CC=C2CS1)N1CC(=O)NC2=C1C=CC=C2 | 2 | I |
| CC1=CC=C(C=C1N1CCNC1=O)C(=O)N1CCC[C@@](C)(C1)C1=CC=CC=C1 | 2 | I |
| CCC1=NNC(=C1)C(=O)N1C[C@H]2CC[C@@H]1CN(C2)C1CC2=CC=CC=C2C1 | 2 | I |
| C[C@@H]1CCC2=C(C1)C=C(S2)C(=O)N1CCC[C@@H]1C(=O)NC1=NC=CS1 | 2 | I |
| FC1=CC=CC=C1CN1CCC2(CC1)N(CCC1=C2N=CN1)C(=O)C1CCC1 | 2 | I |
| O=S1(=O)[N-]CC2(CC[NH+](CCC3=CC=CC=C3)CC2)COC2=CC=CC=C12 | 2 | I |
| CC1=CC(=NN1)C1=CC(=C(C)S1)S(=O)(=O)N1CCC2=CC=CC=C2C1 | 2 | I |
| CC(=O)O[C@@H]1CC[C@@]2(\C=N/O)[C@@H]3CC[C@]4(C)[C@H](CCC4=O)[C@@H]3CC=C2C1 | 2 | I |
| CCN1C(=O)N\C(=C/C2=C(C)N(C(C)=C2)C2=CC=CC=C2Br)C1=O | 2 | I |
| O=C(N[C@H]1C[C@@H]2C[C@H]1CN2C(=O)C1=NC=CC2=CC=CC=C12)C1=CC=CS1 | 2 | I |
| CCC1(O)CN(C1)C1=CC(F)=C(I)C=C1[N+]([O-])=O | 2 | I |
| O=C(\C=C/C1=CC=CC=C1)N1CCN(CC1)C(=O)C1=CNC2=C1C=CC=C2 | 2 | I |
| CC1=CC=C(C(=O)N2CCN(CC2)C2=CC(=NN2)C2=CC=CS2)C(C)=C1 | 2 | I |
| C[C@@H]1C(=O)N2CCCC3=C2C1=CC(=C3)S(=O)(=O)NC1=CC=C(F)C(F)=C1 | 2 | I |
| O=C(N1CCS[C@H](CC1)C1=CC=CO1)C1=CC(=NN1)C1=CC=CS1 | 2 | I |
| CC[C@@H](C)NC(=O)CN1N=NC2=C(C3=C(C[C@@H](C)CC3)S2)C1=O | 2 | I |
| CN1C(=O)N(CC(=O)NCC2=CC=C(C)C=C2)C(=O)C11CCCCC1 | 2 | I |
| O=C1N[C@]2(CCOC3=C2C=CC=C3)C(=O)N1CCOC1=CC=CC=C1 | 2 | I |
| C[C@@H](SC1=NN=C2CCCCCN12)C(=O)NC1(CCCCC1)C#N | 2 | I |
| CCC1=CC=C(\C=C2\C(=O)NC(=O)N(C2=O)C2=CC=C(OC)C=C2)C=C1 | 2 | I |
| [O-]C1=NC(=NC(=C1)[C@@H]1CCC[NH+](CC2CCCC2)C1)N1CCOCC1 | 2 | I |
| C[C@@]1(NC(=O)N(CC2=CC(=NO2)C2=CC=CC=C2)C1=O)C1=CC=CC=C1 | 2 | I |
| CC1=NN(C(=[SH+])\C1=C/NC1=CC=C(C=C1)[N+]([O-])=O)C1=CC=CC=C1 | 2 | I |
| C[C@H](NC(=O)[C@@H]1CCCN(C1)C1=CC=C(Cl)N=N1)C1=CC=C(C)O1 | 2 | I |
| CC1=CC=C(C)C(NS(=O)(=O)C2=CC=C(C=C2)N2CCOC2=O)=C1 | 2 | I |
| CCCS(=O)(=O)N1CCC(CC1)NC(=O)N1C[C@H](C)C[C@@H](C)C1 | 2 | I |
| COC(=O)C1=C(C)C2=C([O-])N=C(C[NH+]3CCC[C@@H](C)C3)N=C2S1 | 2 | I |
| O=S(=O)(CC1=NOC(=C1)C1=CC=CS1)C1=NC2=CC=CC=C2N1 | 2 | I |
| C[C@H]1C[C@@H](C)CN(C1)C(=O)NC[C@@H](N1CCOCC1)C1=CC=C(C)O1 | 2 | I |
| C[C@H](NC(=O)CC1=COC2=C(C)C(C)=CC=C12)C1=NN=C2C=CC=CN12 | 2 | I |
| CC1=CC=C(O1)[C@@H](CNC(=O)N1CCCCCC1)N1CCOCC1 | 2 | I |
| CCCN1\C(NC2=CC=CC=C12)=N\C(=O)[C@@H]1CCCN(C1)C(=O)CC | 2 | I |
| O=C(CCSC1=NC2=C(N=N1)C1=CC=CC=C1N2)N1CCCCC1 | 2 | I |
| C[C@H](NC(=O)N1CCN(CC1)C1=CC=C(C=C1)C(C)=O)[C@H]1CCCO1 | 2 | I |
| CC1=C(C(C)=NO1)S(=O)(=O)N1CCC(CC1)NC1=CC=CC=C1C | 2 | I |
| CC1=NN=C(O1)C1=CSC(=C1)S(=O)(=O)NC1=CC=C(C)C(C)=C1 | 2 | I |
| CCC1=CC=C([N-]S(=O)(=O)C2=C(C)[NH+]=C3SC(C)=NN23)C=C1 | 2 | I |
| CCN([C@@H]1CCCC[C@H]1C)C(=O)CC1=C(C)N2NC(=O)C=C2N=C1C | 2 | I |
| CN(CC1=CC=CC(=C1)C#N)C(=O)N[C@@H]1CCN(C1=O)C1=CC=CC=C1 | 2 | I |
| [O-][N+](=O)C1=CC(C#N)=C(NCC2=CC(Br)=CS2)N=C1 | 2 | I |
| C[C@@H]1CCC[C@H](C1)OC(=O)CN1C(=O)N[C@]2(CCCC[C@@H]2C)C1=O | 2 | I |
| COC1=CC(=CC=C1O)C1=NC=CN1CC1=CC2=NSN=C2C=C1 | 2 | I |
| CC1=NN(C=C1)C1=CC=C(N=N1)N1CCC(CC1)[C@H](O)C1=CC=CC=C1 | 2 | I |
| COC1=CC=CC(\C=C2/C(=O)OCC3=CC(OC)=C(OC)C=C23)=C1O | 2 | I |
| COC1=CC2=C(C=C1OC)C(=O)N(CC1=CC=C(Cl)C=C1)C(=O)N2 | 2 | I |
| CCCN1CCC[NH+](CC2=NC3=C(OC4=CC=CC=C34)C([O-])=N2)CC1 | 2 | I |
| CC(C)N1N=CC=C1NC(=O)N1CCN(CC2=CC=CC=C2)C[C@H]1C | 2 | I |
| CCC1=CC(=NC(=N1)N1CCCCC1)N1CCC[C@@H](C1)C1=NC=CN1 | 2 | I |
| C[C@H](NC1=CC=C(C=C1Br)[N+]([O-])=O)C1=C(C)ON=C1C | 2 | I |
| CCOC1=CC2=C(O[C@H](C)C2)C=C1CN1C(=O)NC2(CCCC2)C1=O | 2 | I |
| CCC1(CC)NC(=O)N(CC(=O)N2CCCCC3=C2C=CC=C3)C1=O | 2 | I |
| C[NH+](CC1=NC2=CC=NN2C([O-])=C1)[C@H]1CCCN(C1)C1=CC=CC=C1 | 2 | I |
| CC(C)(C)C1=NN=C([N-]S(=O)(=O)C2=CC=CC3=C2[NH+]=CC=C3)S1 | 2 | I |
| CC(C)CNC(=O)N1CCC[C@@H](C1)C(=O)N1CC[C@@H](C1)C1=CC=CO1 | 2 | I |
| CC[C@H](C)C(=O)N1CCC2=C(C1)C(=NN2)C(=O)N(C)C1CCCCC1 | 2 | I |
| FC1=CC=CC(F)=C1NCC1=NOC(=N1)[C@@H]1COC2=CC=CC=C2O1 | 2 | I |
| COC1=CC=C(F)C(NS(=O)(=O)C2=CC=C3OC(=O)C=CC3=C2)=C1 | 2 | I |
| CC1(C)CCN(C1)C(=O)NCCCN1C(=O)[C@H]2[C@H]3C[C@H](C=C3)[C@@H]2C1=O | 2 | I |
| O=C(NC(C1CC1)C1CC1)N1CCC[C@H]1C1=NN=C2CCCCCN12 | 2 | I |
| CCOC1=CC2=C(O[C@H](C)C2)C=C1CNC(=O)N1CCCN(C)CC1 | 2 | I |
| CC1=CC=C(C2=NN=C([N-]S(=O)(=O)C3=CC=C[NH+]=C3)S2)C(C)=C1 | 2 | I |
| CC(C)CC(=O)N1CC[C@@]2(C[C@@H]2C(=O)NC2=CC=C3OCOC3=C2)C1 | 2 | I |
| COC1=CC=NC(=N1)N1CCN(CC2=CC3=C(CCC3)C=C2O)CC1 | 2 | I |
| COC1=CC=C(C(C)=C1)S(=O)(=O)NC1=CC=C2OC(=O)C=CC2=C1 | 2 | I |
| [O-][N+](=O)C1=CC=C(S1)S(=O)(=O)N[C@H]1CCC2=C1C=CC=C2F | 2 | I |
| CN1C2=C([C@H](NC1=O)C1=CC=CC=C1)C(=O)N(C[C@@H]1CCCO1)C2 | 2 | I |
| C[C@@H]1CCC[C@@H](C)N1C(=O)CN1C(=O)N[C@@](C)(C1=O)C1=CC=CC=C1 | 2 | I |
| COC(=O)[C@@]1(O)CC(C)=NN1C(=O)C1=CC=C(Br)C=C1 | 2 | I |
| O=C(NC1=CC=CC=C1N1CCOCC1)[C@H]1COC2=CC=CC=C2O1 | 2 | I |
| CC1=NC(NCC2=NN=C3CCCN23)=C2C(SC3=C2CCCC3)=N1 | 2 | I |
| CC1=CC(CN2C(=O)N[C@@](C)(C2=O)C2=CC=C3CCCC3=C2)=NO1 | 2 | I |
| CCN1[C@@H]2[C@@H](NC3=CC=CC=C13)N(C(=O)[C@@H]2C(C)=O)C1=CC=CC=N1 | 2 | I |
| O=C([C@H]1CCC=CC1)N1CCC[C@@H]1C1=NC(=NN1)C1=CC=NC=C1 | 2 | I |
| CC[C@H]1CCCCN1S(=O)(=O)C1=CC=C2OCC(=O)NC2=C1 | 2 | I |
| OC1=CC=C2C(=O)\C(OC2=C1CN1CCOCC1)=C/C1=CC=CO1 | 2 | I |
| OC[C@@H]1CCCCN1C(=O)C1=CC(=C(Br)C=C1)[N+]([O-])=O | 2 | I |
| C[C@H](N1CCN(CC1)C1=NC=CS1)C1=NC2=CC=CC=C2C(=O)N1 | 2 | I |
| C[C@]1(NC(=O)N(CC2=CC=C(C=C2)C#N)C1=O)C1=CC=C(C=C1)C#N | 2 | I |
| C[C@@]1(NC(=O)N(CC(=O)N2CCCCCC2)C1=O)C1=CC=CC=C1 | 2 | I |
| O=C(CC1=CN(N=C1)C1=CC=CC=C1)N1CC(=O)NC2=C1C=CC=C2 | 2 | I |
| CCOC(=O)N1CCC(CC1)N1C(=S)NC2=CC=CC=C2C1=O | 2 | I |
| C[C@H]1CN(C[C@H](C)O1)C1=CC(=O)N(CC2=CC=C(F)C=C2)C(=O)N1 | 2 | I |
| ClC1=NC=C(S1)S(=O)(=O)N1CCC[C@@H](C1)C1=CC=NN1 | 2 | I |
| O=C(N1CCC[C@H]1C1=CC2=C(OCCO2)C=C1)C1=NNC(=C1)C1CC1 | 2 | I |
| O=C(N[C@@H]1CCCC[C@H]1N1C(=O)CSC1=O)C1=CC=C(C=C1)C#N | 2 | I |
| CNC1=C(N2C=CSC2=N1)S(=O)(=O)N1[C@H](C)CCC[C@H]1C | 2 | I |
| CCN(C)S(=O)(=O)C1=CC=C2OC3=CC=CC=C3NC(=O)C2=C1 | 2 | I |
| CC1=CC=C2O[C@@H](CCN(C2=C1)S(C)(=O)=O)C(=O)NC(C)(C)C | 2 | I |
| CN1NC(C)=C([C@H]2N=CN=C2C2=CC=CC=C2)[C@H]1N1CCOCC1 | 2 | I |
| O=C(N1CCC[C@@H](C1)N1C=CC=N1)C1=CC(=NN1)C1=CC=CS1 | 2 | I |
| C[C@H]1CN(CC2=CC(=O)N3N=CC=C3N2)C[C@@H](O1)C1=CC=CC=C1 | 2 | I |
| CC1CCN(CC1)C(=O)C1CCN(CC1)C(=O)NC[C@@H]1CCCO1 | 2 | I |
| COC1=CC=CC(=C1)C1=NN2C=CN=C(N3CCC[C@@H](O)C3)C2=C1 | 2 | I |
| O=C(N1CCN(CC2=CC=CO2)CC1)C1=CC2=CC=CC=C2C(=O)N1 | 2 | I |
| O=C(CC1=CSC(=N1)N1CCNC1=O)N1CC[C@@H]2CCCC[C@H]12 | 2 | I |
| COCC1=CC=C(O1)C(=O)N1CCC[C@]2(C1)C(=O)NC1=CC=CC=C21 | 2 | I |
| CC1=NC(=CC(=O)N1)C1CCN(CC2=CC3=NSN=C3C=C2)CC1 | 2 | I |
| CC(C)C1=NNC2=C1CN(CC2)C(=O)CC1=CN2C=CSC2=N1 | 2 | I |
| COC1=CC2=C(C=C1OC)C(=O)N([C@H]1CCC3=C1C=CC=C3)C(=O)N2 | 2 | I |
| C[C@@]12[C@@H]3C[C@@H](C=C3)[C@H]1C(=O)N(CC1=NC(=NN1)C1=CC=CC=C1)C2=O | 2 | I |
| O=C1NC2(CCSCC2)C(=O)N1CC1=COC(=N1)C1=CC=CC=C1 | 2 | I |
| CC1(C)[C@H](NC(=O)CN2C(=O)C3=CC=CC=C3C2=O)[C@H]2CCO[C@H]12 | 2 | I |
| C[C@@H]1C[C@H](CC[NH+]1C)N(C)C(=O)C1=CC=C(C=C1Cl)[N+]([O-])=O | 2 | I |
| FC(F)(F)C1=NNC(=C1)[C@@H]1CCCN(C1)C(=O)C1=CSN=N1 | 2 | I |
| CCN1C=NN=C1CNC(=O)N1CCSC2(CCCCC2)C1 | 2 | I |
| C[NH+](CC1=NC2=CC=CC=C2C([O-])=N1)[C@@H]1CCOC2(CCOCC2)C1 | 2 | I |
| C[C@H]1COCCN1C(=O)N[C@H]1CCCN(CC2=CC=CC=C2)C1=O | 2 | I |
| CC(C)=CCN1CC2(CC1=O)CCN(CC2)C(=O)C1=C(O)C=CC=N1 | 2 | I |
| O=C(N1C[C@@H]2CC[C@H](C1)[NH+](CC1CCC1)C2)C1=CC2=C([N-]N=N2)C=C1 | 2 | I |
| CC1=C(CN2CCN(CC2)C2=C(C=NC3=CC=CC=C23)C#N)C=NN1 | 2 | I |
| O=C(NC1CCCC1)[C@H]1CO[C@H]2CCN(C[C@H]2C1)C1=NC=CC=N1 | 2 | I |
| [O-]C(=O)C1=C([NH+]=C2CCN(CCC2=C1)C1CCOCC1)N1CCCC1 | 2 | I |
| CC1=NN=C2CC[C@@H](CN12)NC(=O)CC1=COC2=C1C=C(C)C(C)=C2 | 2 | I |
| CN1C(=O)OC2=C(C=CC=C12)C(=O)NC[C@H]1OCCC2=C1C=CS2 | 2 | I |
| [O-][N+](=O)C1=CC=C(C=C1)[C@H](Br)C(=O)\N=C1/NCCS1 | 2 | I |
| COC(=O)C1=C(C=C2C(=O)C3=CC=CC=C3C2=O)C2=CC=CC=C2N1 | 2 | I |
| O[C@]1(N(C(=O)C2=CC=CC=C12)C1=CC=C2OCCOC2=C1)C1=CC=CC=C1 | 2 | I |
| CC1=CC=C(C=C1C)N1C[C@@H](CC1=O)NC(=O)N1CCC2=CC=CC=C2C1 | 2 | I |
| CSC1=NC=C(C#N)C2=N[C@@H](NN12)C1=C(Cl)C=CC=C1Cl | 2 | I |
| OC1=CC(=CC=C1)C1=NN2[C@@H](C1)C1=CC=CC=C1O[C@H]2C1=CC=CC=N1 | 2 | I |
| OC1=CC(=CC=C1)C1=NN2[C@H](C1)C1=CC=CC=C1O[C@@H]2C1=CC=CN=C1 | 2 | I |
| CCCN1C(=O)NC(=O)C2=C1N=CC=C2C1=CC=CC2=C1C=CC=C2 | 2 | I |
| CC(C)N1C(=O)NC(=O)C2=C1N=CC=C2C1=CC=CC2=C1C=CC=C2 | 2 | I |
| COC1=CC(\C=C2/OC3=CC(O)=CC=C3C2=O)=CC(Cl)=C1OC | 2 | I |
| O=C(N1CCCCC1)C1=CC=C(NC2=NN=CC3=CC=CC=C23)C=C1 | 2 | I |
| FC1=CC(=CC=C1)[C@@H]1CC(=O)NC2=C1C=NN2C1=NC2=CC=CC=C2S1 | 2 | I |
| CSC1=NN2[C@@H](C3=CC(C)=CC=C3)C3=C(C[C@@H](C)CC3=O)NC2=N1 | 2 | I |
| C[C@@H]1CCCN(C1)S(=O)(=O)\N=C1/NC=C(Br)S1 | 2 | I |
| C[C@H]1CN(CC2=C(O)C(Cl)=CC3=C2OC(=O)C=C3C)C[C@@H](C)O1 | 2 | I |
| C[C@@H]1C[C@@]2(CC(C)(C)C1)NC(=O)N(CN1CCC3=CC=CC=C3C1)C2=O | 2 | I |
| O=C1C[C@H](CC2=C1[C@@H](N1N=CN=C1N2)C1=CC=CC=C1)C1=CC=CC=C1 | 2 | I |
| O=C1N=C([N-]C2=C[NH+]=CC=C2)S\C1=C/C1=CC=C2N=CC=CC2=C1 | 2 | I |
| FC1=CC2=C(N(CC3=NS(=O)(=O)C4=CC=CC=C4N3)CCC2)C(F)=C1 | 2 | I |
| C[C@H]1CCCC[C@@]11NC(=O)N(CC2=CC=C(Cl)C3=C2N=CC=C3)C1=O | 2 | I |
| OC1=CC=C(C=C1)C1=NN2[C@H](C1)C1=CC=CC=C1O[C@H]2C1=CC=CN=C1 | 2 | I |
| CC1=CC=C(C=C1)C1=NO[C@H](N1)[C@H]1C=C(N=N1)C1=CC=C(C)C=C1C | 2 | I |
| CC1=CC=CC(=C1)C1=NO[C@H](N1)[C@@H]1C=C(N=N1)C1=CC(C)=CC=C1C | 2 | I |
| C[C@@H](NC(=O)N1CCCCCC1)C1=CC=C(C=C1)N1CCOCC1 | 2 | I |
| C[C@@H]1CC2=CC=CC=C2N1C(=O)N[C@@H]1CCN(C1=O)C1=CC=CC=C1 | 2 | I |
| [O-]C1=N[C@H]([NH+]2CCCC[C@@H]12)C1=CC=C(O1)C1=NC2=CC=CC=C2S1 | 2 | I |
| C[C@H]1CCN(CC2=NC3=C(OC4=CC=CC=C34)C(=O)N2)C2=C1C=CC=C2 | 2 | I |
| C[C@@H]1CCC[C@@H](CCNC(=O)N2CCS(=O)(=O)[C@H](C)[C@@H]2C)C1 | 2 | I |
| O=C(NC1=CC=C(C=C1)N1C(=O)C2=CC=CC=C2C1=O)C1CC=CC1 | 2 | I |
| O=C(N1CCOC2=C1C=CC=C2)C1=CC=C(NC1=O)C1=CC=CC=C1 | 2 | I |
| FC1=CC=C(C=C1)[C@@H]1CCCN1C(=O)NC1CCC2(CC1)OCCO2 | 2 | I |
| C[C@@H]1CN(CCO1)C1CCN(CC1)C(=O)NC1=CC(C)=CC(C)=C1 | 2 | I |
| C[C@H]1CCC[C@H](C1)NC(=O)N1CCC(CC1)C(=O)N1CCC[C@H](C)C1 | 2 | I |
| C[C@@H]1CCC[C@H](NS(=O)(=O)C2=CC=CC3=NSN=C23)[C@@H]1C | 2 | I |
| OC1=CC(=CC=C1)N1C(\C=C/C2=CC=CO2)=NC2=CC=CC=C2C1=O | 2 | I |
| CC(C)C1=C(I)C(Cl)=NC(=N1)C1=NC=NN1 | 2 | I |
| COC1=C\C(NC=C1)=N\S(=O)(=O)C1=CC=C(Cl)C(Cl)=C1 | 2 | I |
| CC1=CN=C2SC[C@@H](CN2C1=O)C(=O)NC12CC3CC(CC(C3)C1)C2 | 2 | I |
| CC1(OC(=O)C(NC2=CC=CC=C2Br)C(=O)O1)C=C | 2 | I |
| CCN1\C(NC2=CC(Br)=CC=C12)=N\C(=O)[C@H]1CC[C@H](C)O1 | 2 | I |
| ClC1=C(N[C@@H]2CCO[C@]3(CCOC3)C2)C2=NSN=C2C=C1 | 2 | I |
| BrC1=CN2C=CN=C2C(NC2=CC=C3N=CC=CC3=C2)=N1 | 2 | I |
| BrC1=CN=CC(=C1)C1=NOC(=N1)[C@@H]1CC2=CC=CC=C2N1 | 2 | I |
| CC(C)(C)OC(=O)N1C[C@@H](C(O)=O)[C@]2(C1)CCCC1=C2C=CC=C1 | 2 | I |
| CC1=C(C)S\C(N1)=N\S(=O)(=O)C1=CC=CC(Cl)=C1C#N | 2 | I |
| CC1=CN=C(C)C(=N1)N1CCC2=NNC(\C=C/C3=CC=CC=C3)=C2C1 | 2 | I |
| COC1=C(F)C(C)=C(C=C1)S(=O)(=O)\N=C1/NC=CC=C1Cl | 2 | I |
| CN(C(=O)N1CCC2(C[C@@H](C(=O)N2)C2=CC=CC=C2)CC1)C1=CC=CC=C1 | 2 | I |
| CC1=CC=C(C=C1NC(=O)N1C[C@]2(C)C[C@H]1CC(C)(C)C2)S(C)(=O)=O | 2 | I |
| CC1=NC2=C(C3=C(CCCC3)S2)C(=O)N1\N=C/C1=CNC2=C1C=CC=C2 | 2 | I |
| CC1=CC=C2C(NC=C2C(=O)C(=C/C2=CC=CC3=C2OCO3)\C#N)=C1 | 2 | I |
| CC1=CC=C2NC3=C(N=CN(\N=C\C4=CC=CC=C4Cl)C3=O)C2=C1 | 2 | I |
| CN(C=C1C(C)=NN(C1=O)C1=CC=C(C)C(C)=C1)C1=CC=C(O)C=C1 | 2 | I |
| CC1=CC=C(NS(=O)(=O)C2=CC=C3OCCCOC3=C2)C(C)=C1 | 2 | I |
| COC1=CC(=CC=C1)[C@@H]1NC2=C(C=C(C=C2C)[N+]([O-])=O)[C@H]2C=CC[C@H]12 | 2 | I |
| O=C1OC2=CC=CC=C2C=C1C1=NN=C(NC2=CC=CC=C2)SC1 | 2 | I |
| COC(=O)C1[C@H]([C@H]2C(=O)CCCC2=NC1=C)C1=CNC2=C1C=CC=C2 | 2 | I |
| C(SC1=NN=C2C=CC3=CC=CC=C3N12)C1=NC2=CC=CC=C2N1 | 2 | I |
| C[C@H](C(=O)N1CCC[C@]2(C1)CNC(=O)C1=CC=CC=C1O2)C1=CC=CC=C1 | 2 | I |
| CC(=O)C1=CC=C2N[C@@H]([C@@H]3CC=C[C@H]3C2=C1)C1=CC=CC=C1[N+]([O-])=O | 2 | I |
| COC1=CC=C(C=C1[N+]([O-])=O)[C@H]1NC2=C(C=CC=C2C)[C@@H]2C=CC[C@@H]12 | 2 | I |
| CC1=C2C=CC=CC2=[NH+]C(C2=CC=C3OCCOC3=C2)=C1CC([O-])=O | 2 | I |
| COC1=CC=CC=C1[C@H]1NC2=C(C=C(C=C2C)[N+]([O-])=O)[C@@H]2C=CC[C@@H]12 | 2 | I |
| COC1=CC(=CC=C1)[C@H]1NC2=C(C=CC(=C2C)[N+]([O-])=O)[C@@H]2C=CC[C@@H]12 | 2 | I |
| CC1=CC=CC(=C1)N1CCN(CC1)C(=O)C1=CC2=CC=CC=C2C(=O)N1 | 2 | I |
| O=C1NC2=CC=C(C=C2C2=CC=CC=C12)\N=C\C1=CC=C2OCOC2=C1 | 2 | I |
| COC1=CC=C(Cl)C=C1CN1C(=O)NC2(CCCCCC2)C1=O | 2 | I |
| FC(F)(F)[C@@H]1[N-]C2=CC=C\C(=[NH+]/CC3=CC4=C(OCO4)C=C3)C2=N1 | 2 | I |
| CCN1\C(S\C(=C/C2=CC=CC=C2O)C1=O)=N\C1=CC=CN=C1 | 2 | I |
| CC(C)(C)OC(=O)N1CCC[C@@]2(CC[C@@H](O2)C2=CC=C(O)C=C2)C1 | 2 | I |
| CCN1C(=O)\C(S\C1=N/C1=CC=CN=C1Cl)=C/C1=CC=CN1 | 2 | I |
| C[C@H]1C[C@@H](C)CN(C1)C(=O)NCCC1=CC(F)=CC2=C1OCOC2 | 2 | I |
| ClC1=NC=C(S1)S(=O)(=O)\N=C1/NC2=C(CCCC2)S1 | 2 | I |
| CC(C)[C@@H]1CC[C@@H](C)C[C@H]1NC(=O)C1CCN(CC1)C(=O)N(C)C | 2 | I |
| CC(C)(C)[C@@H]1CCC2=C(C1)SC1=C2N=C(NC1=O)C1=NC=CC=N1 | 2 | I |
| COC1=CC=C(C=C1OC)[C@@H]1NC2=CC=C(C)C=C2C2=CC(C)=NN12 | 2 | I |
| C[C@@H]1CCC[C@@H](C)C11NC(=O)N(CN(C)C2CCCCCC2)C1=O | 2 | I |
| CC1=CC=C(C=C1)[C@H]1C[C@@H](N2N=NN=C2N1)C1=CC=C(Cl)C=C1 | 2 | I |
| COC1=CC(=CC=C1)[C@H]1NC2=C([C@@H]3C=CC[C@H]13)C(=CC=C2C)[N+]([O-])=O | 2 | I |
| CC1=CC=C(C)N1C1=CC=C(C=C1)S(=O)(=O)\N=C1/NC=CS1 | 2 | I |
| C(C1=NN2C(S1)=NN=C2C1=CC2=CC=CC=C2N1)C1=CC=CC=C1 | 2 | I |
| C[C@@H]1CC[C@@H](NC2=C(C=C(Br)C=N2)[N+]([O-])=O)[C@H](C)C1 | 2 | I |
| CC(C)C1=CS\C(N1)=N/S(=O)(=O)C1=CC=CC(F)=C1C#N | 2 | I |
| C[C@H](NC(=O)N1C[C@H](C)C[C@@H](C)C1)C(=O)N1CCC2=C1C=CC=C2 | 2 | I |
| O=C(COC1=C(C#N)C2=CC=CC=C2N1)N1CCC[C@@H]2CCCC[C@@H]12 | 2 | I |
| CN1C(=O)C(CNC2=CC=C3OCOC3=C2)=CC2=C1C=C(C)C(C)=C2 | 2 | I |
| CC[C@H]1CCC[C@H](C1)NC(=O)N1CCC(CC1)N1CCO[C@H](C)C1 | 2 | I |
| C[C@H](N1CCOCC1)C1=CC=C(NC(=O)N2CCCCCC2)C=C1 | 2 | I |
| C[C@H](CC1=C(C)ON=C1C)NC(=O)N1CCC2(CCCCC2)CC1 | 2 | I |
| O=C(NCC1=CC=CC=C1)N1CCCN(CC1)C1=CC=CC=C1C#N | 2 | I |
| O=C1N=C(NC2=CC=CC=N2)S\C1=C\C1=CC2=CC=CC=C2OC1 | 2 | I |
| C[C@@H]1CCC[C@H](C1)NC(=O)N1CCN(CC1)C(=O)C1=CC=C(C)S1 | 2 | I |
| CC[C@@H]1CCCC[C@H]1NC(=O)N1CCC(CC1)N1CCO[C@H](C)C1 | 2 | I |
| CN(CC1=NC2=C(OC3=CC=CC=C23)C(=O)N1)C1=CC=C(C)C=C1C | 2 | I |
| O=C(OCC1C2=CC=CC=C2C2=CC=CC=C12)N1CCCNC(=O)C1 | 2 | I |
| COC1=CC(OC)=C(F)C=C1NC(=O)N1CCC2(CCCC2)CC1 | 2 | I |
| [O-][N+](=O)C1=C(C=C(Br)C=C1)N1CCNCC11CCCCC1 | 2 | I |
| CCOC1=CC=C(Cl)C=C1\C=C1\S\C(NC1=O)=N\C(C)=O | 2 | I |
| O=C1NC(\C=C/C2=CC=C(C=C2)N2CCOCC2)=NC2=CC=CC=C12 | 2 | I |
| CC[C@@H]1CCCN(CN2C(=O)N[C@]3(C[C@H](C)CC(C)(C)C3)C2=O)C1 | 2 | I |
| COC1=CC=C([C@H]2CC(O)=NC3=C2C=NN3C(C)C)C2=C1C=CC=C2 | 2 | I |
| C[C@@H]1NC2=CC=CC=C2C2=C(O1)N=C(SCC1=CC=CC=C1)N=N2 | 2 | I |
| CSC1=NC2=C(N=N1)C1=CC=CC=C1N[C@H](O2)C1=CC=CC=C1C | 2 | I |
| C[C@@H]1CC2=CC=CC=C2N1S(=O)(=O)C1=CC=C2NC(=O)C3=CC=CC1=C23 | 2 | I |
| COC(=O)C1[C@@H](C2=CC=C(O)C=C2)C2=C(CC(C)(C)CC2=O)N=C1C | 2 | I |
| CC1=CC=C(C)C(CSC2=NN=C3NC(=O)C4=C(CCCC4)N23)=C1 | 2 | I |
| CN(C)C1=CC=C(C=C1)[C@H]1NC2=C(C=CC=C2[C@H]2C=CC[C@@H]12)[N+]([O-])=O | 2 | I |
| COC1=CC=CC(=C1)[C@H]1NC2=C(F)C=CC(=C2[C@H]2C=CC[C@H]12)[N+]([O-])=O | 2 | I |
| CC(C)C1=CC2=C(C(I)=NN2)C(=C1)[N+]([O-])=O | 2 | I |
| CC1=CC=C(C=C1C)C(=O)CN1C(=O)NC2(CCCCCCC2)C1=O | 2 | I |
| CCC1=CC=C(C=C1)C1=NNC2=C1CN(CC2)C1=CC=C(Cl)N=N1 | 2 | I |
| COC1=CC=CC(=C1)[C@H]1NC2=C(F)C=CC(=C2[C@@H]2C=CC[C@@H]12)[N+]([O-])=O | 2 | I |
| C[C@@H]1C[C@@H](C)C[NH+](C1)[C@H](C1=C([O-])N2N=CN=C2S1)C1=CC=CC=C1 | 2 | I |
| CN(CN1C(=O)NC2(CCCCCC2)C1=O)[C@@H]1CCC2=C1C=CC=C2 | 2 | I |
| ClC1=NC=C(S1)S(=O)(=O)\N=C1\NC2=CC=CC=C2S1 | 2 | I |
| CC(C)C1=C(C)[C@H](N2NN=NC2=N1)C1=CC=C(Br)C=C1 | 2 | I |
| CC1=CC(C)=C(SCC(=O)\[NH+]=C2/C=CC3=NC([O-])=NC3=C2)C(C)=C1 | 2 | I |
| CN1\C(S\C(=C(\C)C2=CC=C(O)C=C2)C1=O)=N/C1=CC=NC=C1Cl | 2 | I |
| CN1\C(S\C(=C/C2=CNC3=C2C=CC=C3)C1=O)=N/C1=NC(C)=CS1 | 2 | I |
| C[C@H]1CCC[C@H](C)N1C1=CC(=O)N(C(=O)N1)C12CC3CC(CC(C3)C1)C2 | 2 | I |
| [O-][N+](=O)C1=CC=C(C=C1)C1=C[C@@H](CC(=O)N1)C1=CC=C(Cl)C=C1 | 2 | I |
| FC1=CC2=C(OCOC2)C(CCNC(=O)N2CCCCCCC2)=C1 | 2 | I |
| CC1=CC(C)=C(C)C(OC(=O)C2=CC3=C(CCCC3=O)NC2=O)=C1 | 2 | I |
| COC1=CC=CC=C1[C@@H]1C[C@@H](NC2=NC=NN12)C1=CC=CC=C1Cl | 2 | I |
| CCC1=CC=C(C=C1)[C@H]1N2N=CN=C2NC2=C1C(=O)C1=CC=CC=C21 | 2 | I |
| FC1=CC=CC(=C1)[C@@H]1C[C@H](NC2=NN=NN12)C1=CC=C(Cl)C=C1Cl | 2 | I |
| FC1=CC=CC=C1N1CCN(CC1)C1=NC(=CC(=O)N1)C(F)(F)F | 2 | I |
| ClC1=CC=C(C=C1)[C@H]1C[C@H](N2N=NN=C2N1)C1=CC=C(Cl)C=C1 | 2 | I |
| FC1=CC=C(C=C1)[C@H]1C[C@@H](N2N=NN=C2N1)C1=CC=CC2=CC=CC=C12 | 2 | I |
| FC1=CC=C(C=C1)[C@@H]1C[C@@H](N2N=NN=C2N1)C1=CC=CC2=CC=CC=C12 | 2 | I |
| O=C1N(N=C2N[C@H](N3CCCCCC3=C12)C1=CC=CC=C1)C1=CC=CC=C1 | 2 | I |
| O=C(\N=C1/NC2=CC=CC=C2N1C1=CC=CC=C1)[C@H]1CC2=CC=CC=C2O1 | 2 | I |
| COC1=CC=C2N3C(SC2=C1)=NC(O)=C(CC1=CC=CC=C1)C3=O | 2 | I |
| CC1=CC=C(O)C(=C1)C1=NN2[C@H](C1)C1=CC=CC=C1O[C@@H]2C1=CC=CN=C1 | 2 | I |
| COC1=CC(\C=C2/COC3=CC(C)=CC(C)=C3C2=O)=CC(OC)=C1O | 2 | I |
| O=C(NCCC1=CC=CC2=CC=CC=C12)N1CCC2(CC1)OCCO2 | 2 | I |
| CC(C)(CNC(=O)N1CCCCCC1)C1=CC=C2OCCOC2=C1 | 2 | I |
| CC(C)(C)C1=NN2C(NC3=C(CCN(CC4=CC=CS4)C3)C2=O)=C1 | 2 | I |
| OC(=O)C1(CC(=O)N2CCOCC2)CCCCCCCCCCC1 | 2 | I |
| CC(C)(C)C(=O)N1CCOC2=C1C=C(NC(=O)[C@@H]1CCC=CC1)C=C2 | 2 | I |
| FC1=CC(=CC(F)=C1)S(=O)(=O)\N=C1\NC(=CS1)C1=CC=CO1 | 2 | I |
| CC1=CC(F)=CC=C1S(=O)(=O)\N=C1\NC(=CS1)C1=CC=CO1 | 2 | I |
| COC1=CC=C(C=C1)[C@@H]1C[C@H](N=C2NC=NN12)C1=CC=C(Cl)C=C1 | 2 | I |
| CC1(C)[C@H]2CN(C[C@@H]12)C(=O)NC1CCN(CC1)C(=O)C1CCCCC1 | 2 | I |
| CC(C)N1C=[NH+]C=C1C1=NC(=C(Br)C([O-])=N1)C(C)(C)C | 2 | I |
| O=C(N1CCC[C@H]1CC1CCCCC1)C1=CC2=C(C=C1)C(=O)NC2=O | 2 | I |
| C1CCC2(C1)C[C@@H](CO2)NC1CCN(CC1)C1=NC2=CC=CC=C2O1 | 2 | I |
| CC(C)CCN1C(=O)[C@]2(NC(=O)C3=CC=CC=C3O2)C2=CC=CC=C12 | 2 | I |
| C[C@H](NC(=O)N1CCC[C@@H](C1)C1(C)OCCO1)C1=CC=C(C)S1 | 2 | I |
| CN1C=CS\C1=N\C(=O)C1=C(C)C=C(NC(=O)C(C)(C)C)S1 | 2 | I |
| C[C@@H]1CCC[C@@H](CNC(=O)N2CCC(CC2)N2CCO[C@H](C)C2)C1 | 2 | I |
| CO[C@]1(C[C@@H](NO1)C(Cl)(Cl)Cl)C1=CC=C(C=C1)[N+]([O-])=O | 2 | I |
| CN1C2=C([C@H](NC1=O)C1=C(C)C=C(C)C(C)=C1)C(=O)N(CC1=CC=CO1)C2 | 2 | I |
| COC1=CC=CC=C1[C@@H]1NC2=CC=CC=C2C2=C(O1)N=C(SC)N=N2 | 2 | I |
| O[C@@]1(N(C(=O)C2=CC=CC=C12)C1=CC=C(C=C1)[N+]([O-])=O)C1=CC=CC=C1 | 2 | I |
| C[C@@H]1CC(=O)NC2=CC=CC=C2N1C(=O)COC(=O)[C@H]1CCC=CC1 | 2 | I |
| C1CN(CCO1)C1=NC2=C(S1)C=C(NC1=C3C=CSC3=NC=N1)C=C2 | 2 | I |
| COC1=CC=C2C(NC3=C2N=CN(\N=C\C2=CC=C(Cl)C=C2)C3=O)=C1 | 2 | I |
| CC1=CC2=NC(=NC(C)=C2C=C1C)N1CCC(CC1)C(=O)N[C@@H]1CCOC1 | 2 | I |
| CC(C)CN1C(O)=CC2=NN(C3=NC4=CC=CC=C4S3)C(=O)C2=C1C | 2 | I |
| C[C@@H]1CCC[C@H](NC(=O)CN2C(=O)C3=CC=CN3C3=CC=CN=C23)[C@@H]1C | 2 | I |
| C[C@H](SC1=C(C#N)C(C)=C(C)C(C)=N1)C(=O)N1CC(=O)NC2=C1C=CC=C2 | 2 | I |
| C[C@@H]1C[C@@H](C)CN(C1)S(=O)(=O)C1=C(C)C(C)=CC(=C1)C1=NNC(=O)C=C1 | 2 | I |
| CC(=O)C1=C(C)NC2=C([C@@H]1C1=CC=CC(=C1)[N+]([O-])=O)C(=O)CC(C)(C)C2 | 2 | I |
| CC[C@@H]1N(CCN2C(C)=CC=C12)C(=O)NC1=CC=C(C=C1)C(=O)OC | 2 | I |
| CC1=CC=C(C=C1)N1[C@H](NC2=CC=C3OCOC3=C2)C2=NC=CC=C2C1=O | 2 | I |
| CC1=CC=CC(C)=C1NS(=O)(=O)C1=CC=C2OCC3=C(ON=C3)C2=C1 | 2 | I |
| CC[C@@]1(C)NC(=O)N(\N=C\C2=CC3=CC(Cl)=CC(Cl)=C3OC2)C1=O | 2 | I |
| OC1=C(C=C(\C=C2/SC(=S)N(C3CCCCC3)C2=O)C=C1)[N+]([O-])=O | 2 | I |
| CC1=CC(O)=C([C@H](C[N+]([O-])=O)C2=CC=C(Cl)C=C2Cl)C(=O)O1 | 2 | I |
| CC1=CC=C(C)C(=C1)S(=O)(=O)N1CCN(CC1)C1=NC2=CC=CC=C2N1 | 2 | I |
| FC1=CC=C(C=C1)C1=NN2[C@H](NN=C2SC1)[C@@H]1C=C(N=N1)C1=CC=CC=C1 | 2 | I |
| O=C(N[C@@H]1CCC2=CC=CC=C12)N1CCN(CC2=CN3C=CC=CC3=N2)CC1 | 2 | I |
| COC1=CC=CC=C1[C@H]1N2N=CN=C2NC2=C1C(=O)C[C@H](C2)C1=CC=CC=C1 | 2 | I |
| CC[C@H]1N(CCC2=C1C=CS2)C(=O)NCC1=NN=C2C=CC=CN12 | 2 | I |
| CCOC1=CC=C(C=C1)[C@@H]1N2N=CN=C2NC2=C1C(=O)C1=CC=CC=C21 | 2 | I |
| O=C1N=C(NC(C2=CC=CC=C2)=C1C#N)N1CCN(CC1)C1=CC=CC=C1 | 2 | I |
| C[C@@H]1CC2=CC=CC=C2N1C(=O)CSC1=NC2=C(C(C)=CC(C)=N2)C(=O)N1 | 2 | I |
| CC(C)CC1=C[C@@H](N=N1)C1=NN=C2S[C@H](NN12)C1=CC=CC=C1F | 2 | I |
| O=C(N1CCC(CC1)[C@H]1NC2=CC=CC=C2O1)C1=NN=C(C1)C1=CC=CC=C1 | 2 | I |
| CC1(C)[C@H]2CC[C@]1(C)[C@@H](C2)NS(=O)(=O)C1=CC=C(S1)[N+]([O-])=O | 2 | I |
| C[C@@H]1CC2=CC=CC=C2N1C(=O)NCC1=CC=C(N=C1)N1C[C@@H](C)O[C@H](C)C1 | 2 | I |
| COC1=CC(=CC=C1O)C1=NC=NN1C1=C(C#N)C2=C(CCCC2)S1 | 2 | I |
| CC1=CC=C2OCCN(C(=O)CSC3=NC4=C(CCCC4)C(=O)N3)C2=C1 | 2 | I |
| CSC1=CC=C(C=C1)C1=CC=NC2=C1C([O-])=NC(=N2)N1CC[NH+](C)CC1 | 2 | I |
| CC1=CC=C(CNC(=O)N2CCN(CC2)C2=C3C=CSC3=NC=N2)C=C1 | 2 | I |
| CC1(C)[C@@H](OC(=O)C2=CC=C(C=C2)N2CCNC2=O)[C@@H]2CCCO[C@H]12 | 2 | I |
| CC1(C)[C@@H](OC(=O)C2=CC=C(C=C2)N2CCNC2=O)[C@@H]2CCCO[C@@H]12 | 2 | I |
| C[C@@H]1C[C@H](CN1C(=O)C1=CNC2=C1C=C(C=C2)[N+]([O-])=O)C1=CC=C(F)C=C1 | 2 | I |
| C[C@H](NC(=O)N1CC[C@H](C)C[C@@H]1C)C1=CC=CC(=C1)N1CCOC1=O | 2 | I |
| COC1=CC=CC=C1N1CCN(CC1=O)C(=O)C1=CC=C2C=CC=CC2=C1O | 2 | I |
| COC1=CC(=CC=C1NC(=O)N1C[C@@H](C)CC[C@@H]1C)N1CCOC(C)(C)C1 | 2 | I |
| COC1=CC=C2OC3(CCN(CC3)C(=O)OC(C)(C)C)CCNC2=C1 | 2 | I |
| O=C(NC1=CC=CC=C1)[C@@H]1OC2=C([C@H]1C1=CC=CO1)C(=O)OC1=CC=CC=C21 | 2 | I |
| FC1=C(C=CC(=C1)C#N)C(=O)N\N=C/C1=CC2=C(OCO2)C=C1Cl | 2 | I |
| FC1=CC=CC(=C1)C1=NC2=C(CCN(C2)S(=O)(=O)N2CCCCCC2)N1 | 2 | I |
| O=C1N[C@]2(CCC3=CC=CC=C3C2)C(=O)N1CC1=COC(=N1)C1=CC=CS1 | 2 | I |
| CCC1=C(I)C(=O)NC(=N1)C1=CC=C(C=C1)[N+]([O-])=O | 2 | I |
| CCC1=C(I)C(=O)NC(=N1)C1=CC=CC=C1[N+]([O-])=O | 2 | I |
| CC[C@@H]1CCCC[C@@H]1NC(=O)N1CCCN(CC1)C1=C(C=CC=N1)C#N | 2 | I |
| C1SC2=NN=C(N2N=C1C1=CC=CC=C1)C1=NNC(=C1)C1=CC=CC=C1 | 2 | I |
| FC1=CC=C2NC(=O)CN([C@@H](C3=CC=CC=C3)C2=C1)C(=O)C1=CC=CO1 | 2 | I |
| CC1=CC=C(C=C1)N1C(=O)NC(=O)\C(=C/C2=CC=C(Cl)C=C2)C1=O | 2 | I |
| FC1=CC=C(C=C1)S(=O)(=O)NC1=CC=C2OC(=O)SC2=C1 | 2 | I |
| FC1=C(CN2C(=O)N[C@@]3(CCOC4=CC=CC=C34)C2=O)C(Cl)=CC=C1 | 2 | I |
| O=C1N(NC2=NC3=C(C=C12)C(=O)C[C@@H](C3)C1=CC=CS1)C1=CC=CC=C1 | 2 | I |
| C[C@@H]1N2[C@H](CC3=C1NC1=CC=CC=C31)C(=O)N(CC1=CC=CO1)C2=S | 2 | I |
| C[C@@H]1CCCC[C@@]11NC(=O)N(CN2CCC3=C(C2)C=CS3)C1=O | 2 | I |
| C[C@H]1CCCC[C@]11NC(=O)N(CN2CCC3=CC=CC=C3C2)C1=O | 2 | I |
| CC1=CC(=[OH+])C(=NN1C1=CC=C(Br)C(C)=C1)C([O-])=O | 2 | I |
| ClC1=CC=C(C=C1)N1C(=O)NC(=CC1=O)N1CCC2=CC=CC=C12 | 2 | I |
| BrC1=C\C(=C\NN2C(=O)C3=CC=CC=C3C2=O)C(=O)C=C1 | 2 | I |
| CC1=C(C)C(=NC(=N1)N1CCC2=CC=CC=C2C1)N1CCNCC1 | 2 | I |
| FC1=CC2=C(OCOC2)C(CCNC(=O)N2CCSCC2)=C1 | 2 | I |
| C[C@]12CO[C@@]3(C[C@H]1CC[C@]23C)C(=O)NC1=CC=C2OCCOC2=C1 | 2 | I |
| CC1=CC=CC=C1[C@H]1CC(=O)C2=C(C1)NC1=NC=NN1[C@@H]2C1=CC=CC=C1 | 2 | I |
| O=C1NC(=NC2=C1CCCC2)N1CCN(CC2=CC=CC=C2)CC1 | 2 | I |
| C[C@@]1(NC(=O)N(CC2=CC=C(F)C=C2Cl)C1=O)C1=CC=CO1 | 2 | I |
| ClC1=CC=CC=C1C1=NO[C@H](N1)[C@H]1C=C(N=N1)C1=CC=CC=C1 | 2 | I |
| C[C@@H]1NC(=S)N(C1=O)C1=CC=C(OC2=CC=C(C=C2)C#N)C=C1 | 2 | I |
| COC1=CC=C(C=C1C)[C@H]1CC(=O)NCC2=C1N1C=C(C)C=CC1=N2 | 2 | I |
| CC1=NN(C2=C1[C@@H](CC(=O)N2)C1=CC=NC=C1Cl)C1=CC=C(C)C=C1 | 2 | I |
| O[C@H]1CCCN(C1)C1=C(C=C(C(Cl)=C1)C(F)(F)F)[N+]([O-])=O | 2 | I |
| [O-]C(=O)C1=CC=CC=C1[C@H]1CCN(C1)C1=NC=[NH+]C2=C1SC=C2 | 2 | I |
| [O-]C1=NC(=NC2=C1C[NH+](CC1CCCCC1)CC2)C1=CC=NC=C1 | 2 | I |
| O=C(C1CCN(CC1)C1=CC=C2C=CNC2=N1)N1CCC2=CC=CC=C2C1 | 2 | I |
| O=C1NC2=CC=CC=C2C(=O)N1CCC1=NC2=CC=CC=C2S1 | 2 | I |
| O=C(N1CCC[C@@H]1C1=CC=C2OCCOC2=C1)C1=CC2=C(C=CN2)C=C1 | 2 | I |
| BrC1=CC=C(CNC(=O)N2CCC3(C2)OCCO3)C=C1 | 2 | I |
| CC1=CC=CC=C1S(=O)(=O)\N=C(/[O-])C1=CC=C2C=CC=CC2=[NH+]1 | 2 | I |
| CC1=CN2C=C(CN3CCN(CC3)C3=CC=C(O)C=C3)N=C2C=C1 | 2 | I |
| ClC1=C(Br)C=C(C=N1)S(=O)(=O)\N=C1/NC=CS1 | 2 | I |
| ClC1=C(N2CCC[C@H](NC3CC3)C2=O)C2=NSN=C2C=C1 | 2 | I |
| CC1=C(C(CCl)=NN1)S(=O)(=O)N1CCCC2=C1C=CC=C2 | 2 | I |
| CC(C)(C)C1=C(I)C(=O)NC(=N1)C1=CN=CC=N1 | 2 | I |
| CNC1=NC(=NC(C)=C1Br)C1=CC=C2OCCOC2=C1 | 2 | I |
| CNC1=C(I)C=NC(=N1)C1=CN2C=CC=CC2=N1 | 2 | I |
| C[NH+]1CCO[C@@H](C1)C1=NC(=C(Br)C([O-])=N1)C(C)(C)C | 2 | I |
| C[C@@H](N1CCC2=CC(F)=CC=C2C1)C(=O)N1CC(=O)NC2=C1C=CC=C2 | 2 | I |
| CC[C@@H]1CO[C@@H](C)CN1C1=CC2=C(C=C1Br)C(=O)C(=O)N2 | 2 | I |
| CC1=C(CN2CCN(CC2)C(=O)C2=CC3=CC=CC=C3N2)OC=C1 | 2 | I |
| CC1=CC=CN2C(CN3CCO[C@@H](C3)C3=CC=CC(O)=C3)=CN=C12 | 2 | I |
| O[C@@H]1CCCC[C@H]1C1=NC(=NO1)C1=CC(Br)=CN=C1 | 2 | I |
| [O-]C1=CN=CC=C1C(=O)N1C[C@H]([C@@H]2[C@H]1C1CC[NH+]2CC1)C1=CC=CC=C1 | 2 | I |
| CSC1=CC=C(NC(=O)N2C[C@H]3C[C@@H](C2)[C@@H]2CCCC(=O)N2C3)C=C1 | 2 | I |
| OC1=NC2=CC=CC=C2CC[C@H]1N1N=C(C=CC1=O)C1=CC=CC=C1 | 2 | I |
| CN1N\C(C=C1C)=N/S(=O)(=O)C1=CC=C(Br)C=C1F | 2 | I |
| CC1(C)CNCCN1C1=CC=C(I)C=C1[N+]([O-])=O | 2 | I |
| CC1=NN(C2=C1[C@H](SCC(O)=N2)C1=CC=CC=C1F)C1=CC=CC=N1 | 2 | I |
| CC(C)(C)N1N=CC2=C1N=C(O)CS[C@@H]2C1=CC=CC2=C1N=CC=C2 | 2 | I |
| CN1C(=O)C[C@@H](N2CCC3=C(NC4=CC=CC=C34)[C@@H]2C2=CC=CC=C2)C1=O | 2 | I |
| OC1=CC=C(C=C1)[C@H]1N(CC2=CC=CC=C2)C(=O)C2=C1C(=O)C1=CC=CC=C1O2 | 2 | I |
| O=C(CSC1=NC2=C(C3=C(CCCC3)S2)C(=O)N1)N1CCCCCC1 | 2 | I |
| C[C@@H](SC1=NC(C)=NC2=C1C(C)=C(C)S2)C1=NC2=CC=CC=C2C(=O)N1 | 2 | I |
| [O-][N+](=O)[C@@H]1[C@@H](SC(=N)C(C#N)[C@H]1C1=CC=CC=C1Cl)C1=CC=CS1 | 2 | I |
| COC1=CC=C2SC=C(N3CCN(CC3)C3=CC=CC=C3O)C(=O)C2=C1 | 2 | I |
| OC1=CC=CC=C1\C=C1/SC(=NC1=O)N1CCN(CC1)C1=CC=CC=C1F | 2 | I |
| O=C(CC1=CN2C=CSC2=N1)NN=C1C2=CC=CC=C2C2=CC=CC=C12 | 2 | I |
| CC1=C(CCC(=O)N2C[C@]3(C)C[C@H]2CC(C)(C)C3)C(=O)OC2=CC(O)=CC=C12 | 2 | I |
| [O-][N+](=O)[C@@H]1[C@H](SC(=N)C(C#N)[C@@H]1C1=CC=CC=C1Cl)C1=CC=CS1 | 2 | I |
| CC[C@]1(C)CC2=CC=CC=C2C2=C1C(=O)N(C(=O)N2)C1=CC=CC=C1OC | 2 | I |
| CN1C(=O)C(C)(C)C2=CC(=CC=C12)S(=O)(=O)NC1=CC(F)=CC=C1C | 2 | I |
| CN1CC[NH+](CC2=C([O-])C=CC3=C2O\C(=C/C2=CC=CC=C2Cl)C3=O)CC1 | 2 | I |
| OC1=C(SC2=NC=NN12)[C@@H](N1CCC2=CC=CC=C2C1)C1=CC=CC(F)=C1 | 2 | I |
| C[C@H]1C[C@@H](C)C[NH+](C1)[C@H](C1=C([O-])N2N=CN=C2S1)C1=CC=C(F)C=C1 | 2 | I |
| CC1=CC=C(C=C1)[C@@H](N1CCC2=CC=CC=C2C1)C1=C(O)N2N=CN=C2S1 | 2 | I |
| CN1C(=O)CC2=CC(NS(=O)(=O)C3=C(Cl)C=C(Cl)C(C)=C3)=CC=C12 | 2 | I |
| CCSC1=NC(=O)[C@@H]2[C@H](C3=CC=CC=C3)C3=C(CC(C)(C)CC3=O)NC2=N1 | 2 | I |
| CCSC1=NC(=O)[C@H]2[C@H](C3=CC=CC=C3)C3=C(CC(C)(C)CC3=O)NC2=N1 | 2 | I |
| CC1(CCCC1)NC(=O)C1=CC(=CC=C1I)[N+]([O-])=O | 2 | I |
| CN1C=[NH+]C(=C1Cl)S(=O)(=O)[N-]C1=CC=C2C(CC3=C2C=CC=C3)=C1 | 2 | I |
| O=C(NC1=CC2=C(OCC[C@@H]3CCCCN3C2=O)C=C1)\C=C\C1=CSC=C1 | 2 | I |
| C[NH+]1CCN(CC2=C([O-])C=CC3=C2O\C(=C/C2=CC=CC=C2Cl)C3=O)CC1 | 2 | I |
| CC1=CSC2=NC(CSC3=NC4=CC(Cl)=CC=C4N3)=CC(=O)N12 | 2 | I |
| CC1=CC(C)=C(C=C1C)C(=O)CN1C(=O)N[C@]2(CCCC3=C2C=CS3)C1=O | 2 | I |
| CC1=CC=CC(=C1)[C@H]1NC2=CC=CC=C2C(=O)N1CC1=CC=C2OCOC2=C1 | 2 | I |
| O=C(NC1CCN(CC1)C1=NC=CC=N1)C1=CC2=C(CCCCCC2)S1 | 2 | I |
| C[C@H](SC1=NC=CN1C)C1=NC2=C(C3=C(C[C@@H](C)CC3)S2)C(=O)N1 | 2 | I |
| COC1=CC(=CC=C1O)C1=NN2[C@@H](C1)C1=CC=CC=C1O[C@@H]2C1=CSC=C1 | 2 | I |
| C[C@H]1CCCC[NH+]1CC1=C2O\C(=C/C3=CC=C(C)O3)C(=O)C2=C(C)C=C1[O-] | 2 | I |
| CC1CC[NH+](CC2=C3O\C(=C/C4=CC=C(C)O4)C(=O)C3=C(C)C=C2[O-])CC1 | 2 | I |
| C[C@@H]1C[C@@H](C)C[NH+](CC2=C([O-])C=C(C)C3=C2O\C(=C\C2=CC=CO2)C3=O)C1 | 2 | I |
| CC1=CC=CC=C1C1=NO[C@H](N1)[C@@H]1C=C(N=N1)C1=CC=CC(Br)=C1 | 2 | I |
| CC(=O)N1CC(C)(C)C2=C1C=CC(=C2)S(=O)(=O)NC1=CC=CC=C1Cl | 2 | I |
| O[C@@H]1CN(CC[C@H]1C1=CC2=C(OCO2)C=C1)C1=CC=C(C=N1)C(F)(F)F | 2 | I |
| CC1=CC(C)=C(C(C)=C1)S(=O)(=O)NC1=CC2=C(OCCO2)C=C1Cl | 2 | I |
| COC1=CC2=C(CN(CC2)C(=O)N[C@H]2CCC3=C2C=CC(F)=C3)C=C1OC | 2 | I |
| CC1=NN(C(O)=C1CC1=CC=C(Br)C=C1)C1=NC(C)=CC(C)=N1 | 2 | I |
| O[C@]1(CCC[C@H]2CN(C[C@H]12)S(=O)(=O)C1=CC=C(Cl)C=C1)C1=CC=CO1 | 2 | I |
| O[C@@]1(CCC[C@H]2CN(C[C@H]12)S(=O)(=O)C1=CC=C(Cl)C=C1)C1=CC=CO1 | 2 | I |
| CC1(C)CC(O)=C(\C=N\C2=CC=C(C=C2)C2=CN3C=CSC3=N2)C(=O)C1 | 2 | I |
| CN1C2=CC=C(C=C2C(C)(C)C1=O)S(=O)(=O)NC1=CC(C)=CC(C)=C1 | 2 | I |
| O=C(NCC1=CC=CC=C1)N1CCN(CC1)C(=O)C1=CC=C2C=CC=CC2=C1 | 2 | I |
| C[C@H]1N(CCC2=C1C=CS2)C(=O)NCCC(=O)N1CCCC2=C1C=CC=C2 | 2 | I |
| CCC1=C(C)SC(=C1)C(=O)N1CCC(CC1)N1C(=O)NC2=CC=CC=C12 | 2 | I |
| CC1=C(C(=O)C2=CC=C3OCN(CCO)CC3=C2O1)C1=CC=C(Cl)C=C1 | 2 | I |
| O=C1NC(=NC2=C1C1=C(CSCC1)S2)C1=CC=C(CN2C=CC=N2)C=C1 | 2 | I |
| ClC1=CC=C(S1)C(=O)N1CCN(CC1)C1=NNC(=C1)C1=CC=CS1 | 2 | I |
| BrC1=CC=CC(NC2=NC=NC3=CC=C(C=C23)N2CCCC2=O)=C1 | 2 | I |
| CN1N(C(=O)C(\N=C/C2=CC(Cl)=CC(Cl)=C2O)=C1C)C1=CC=CC=C1 | 2 | I |
| IC1=CC=C(N\C=C2\N=C(OC2=O)C2=CC=CO2)C=C1 | 2 | I |
| CC1=CC2=C(C=C1C)N=C(CC1=CC=C3OCOC3=C1)C(=O)N2 | 2 | I |
| [O-][N+](=O)C1=CC=C(C=C1)[C@H]1CC(=O)NC2=C1C=C1CCCC1=C2 | 2 | I |
| CC(C)(C)OC(=O)N1N=C2CCCC[C@H]2[C@@]1(O)C(F)(F)F | 2 | I |
| CC1=NC2=C(NC3=CC=CC=C23)C(=O)N1\N=C\C1=CC=CC=C1F | 2 | I |
| OC1=CC=CC=C1[C@H]1CC(=NO1)C1=CC2=CC=CC=C2OC1=O | 2 | I |
| C[C@@H]1C[C@@H](C)CN(C1)C1=CC(=O)N(C(=O)N1)C1=CC=C(C)C=C1 | 2 | I |
| CC1(C)[C@H]2C[C@@H]1C(\C=N\N1C(=O)NC3(CCCCC3)C1=O)=CC2 | 2 | I |
| COC1=CC=C(\C=C2/OC3=C(C=CC(O)=C3C)C2=O)C=C1OC | 2 | I |
| CC1=CC(OCC(=O)N2CC(=O)NC3=C2C=CC=C3)=CC(C)=C1 | 2 | I |
| ClC1=CC=C(C=C1)[C@H]1C[C@@H](NC2=NN=NN12)C1=CC=CS1 | 2 | I |
| FC1=CC=CC=C1[C@H]1C[C@H](NC2=NN=NN12)C1=CC=C(Cl)C=C1 | 2 | I |
| CN1C=CC=C1\C=C1/S\C(=N/C2=CC=C(O)C=C2)N(C)C1=O | 2 | I |
| COC(=O)C1=C(C)NC(C)=C2[C@@H]1C1=C3C=CC=CC3=CC=C1OC2=O | 2 | I |
| CN1CCN(CC2=CC3=C(OC(=O)C4=C3CCCC4)C(C)=C2O)CC1 | 2 | I |
| CN1CCN(CC2=C(O)C=C(C)C3=C2OC(=O)C2=CC=CC=C32)CC1 | 2 | I |
| COC1=CC2=C(CN(CC2)C(=O)NC2CCCC2)C=C1OC | 2 | I |
| CC(C)C1=NC2=CC(NC3CCS(=O)(=O)CC3)=CC=C2O1 | 2 | I |
| CC1=C(C)C2=C(S1)N=C(C)N=C2NC1=CC=C2OCCOC2=C1 | 2 | I |
| CC1=CC=CC(=C1)N1C=C(NC1=S)C1=CC=C(C=C1)[N+]([O-])=O | 2 | I |
| O=C(NC1CCCCCCCCCCC1)C(=O)N1CCOCC1 | 2 | I |
| C[C@@H]1CCC2=C(C1)[C@@H](N=N2)[C@@H]1NC(=NO1)C1=CC=CC=C1Cl | 2 | I |
| CN1\C(S\C(=C/C2=CC=CC=C2O)C1=O)=N\C1=CC=CN=C1 | 2 | I |
| O=S(=O)(\N=C1/NC2=C(CCC2)S1)C1=CC=CC=C1C#N | 2 | I |
| ClC1=CC=C(C=C1)[C@@H]1C[C@@H](NC2=NN=NN12)C1=CC=CC=C1 | 2 | I |
| C1COC2=CC(\C=N/NC3=NC4=CC=CC=C4C=C3)=CC=C2O1 | 2 | I |
| COC1=CC=CC=C1[C@H]1C[C@H](NC2=NC=NN12)C1=CC=CC=C1 | 2 | I |
| CC1=CC(C)=NC(=N1)N1CCC(=CC1)C1=CNC2=C1C=CC=N2 | 2 | I |
| CCC1=CC(=O)NC(=N1)C1=CC=C(N=C1)N1C[C@@H](C)C[C@@H](C)C1 | 2 | I |
| CN1CCN(CC1)C1=NC=NC2=CC=C(C=C12)C1=CC=CC2=C1C=CN2 | 2 | I |
| C[C@H]1C[C@@H](C)CN(C1)C(=O)NC[C@@H]1COC2=CC=CC=C2O1 | 2 | I |
| C[C@@H]1C[C@@H](C)CN(C1)C(=O)NC[C@@H]1COC2=CC=CC=C2O1 | 2 | I |
| O=C(N[C@@H]1CCC[C@H](C1)C1CC1)N1CCC2(CC1)OCCO2 | 2 | I |
| ClC1=CNC(=C1)C(=O)N1CCC[C@@H]1C1=CC2=C(OCO2)C=C1 | 2 | I |
| CC[C@@H]1CCC[C@H](C1)NC(=O)N1CCCN(CC1)C(=O)CC | 2 | I |
| C[C@H]1CCC[C@@H]1NC(=O)N(C)CC1=CC=C2OCCOC2=C1 | 2 | I |
| C[C@@H]1CCC[C@H]1NC(=O)N(C)CC1=CC=C2OCCOC2=C1 | 2 | I |
| CCC1=NO[C@H](C1)C1=NC2=C(C3=C(S2)SCCC3)C(=O)N1 | 2 | I |
| C[C@H](N(C)C(=O)N[C@@H]1[C@@H]2CCCO[C@@H]2C1(C)C)C1=CC=CO1 | 2 | I |
| CC1=CC=CC2=C1N\C(S2)=N\S(=O)(=O)C1=CC=CN=C1 | 2 | I |
| COC1=CC=CC(=C1)[C@H]1C[C@@H](N=C2NC=NN12)C1=CC=CC=C1 | 2 | I |
| O=C(NC[C@@H]1CN2CCCC[C@@H]2CO1)N1CCCCCCC1 | 2 | I |
| CC1(CNC(=O)N2CCC3(C2)OCCO3)CC2=CC=CC=C2C1 | 2 | I |
| COC1=C(OC)C=C(NC(=O)N2C[C@@H](C)CC[C@@H]2C)C(F)=C1 | 2 | I |
| CCOC1=CC=C2N=C(NC2=N1)C1=CC=C(Br)O1 | 2 | I |
| CCCC1=NN(C(O)=C1Br)C1=NC(C)=CC(C)=N1 | 2 | I |
| OC[C@H]1CCCCN1C1=CC(Cl)=C(Cl)C=C1[N+]([O-])=O | 2 | I |
| CC[C@H]1COCCN1C(=O)N[C@H](C)[C@@H]1COC2=C1C=CC=C2 | 2 | I |
| CC1=CC=C2NC(=O)C3(CCN(CC3)C(=O)OC(C)(C)C)C2=C1 | 2 | I |
| CS(=O)(=O)[C@@H]1CCN(C1)C(=O)NC12CC3CC(CC(C3)C1)C2 | 2 | I |
| C[C@H]1CN([C@@H](C)CN1)C1=CC(F)=C(Br)C=C1[N+]([O-])=O | 2 | I |
| CC(C)N1CC2=C([C@@H](NC(=O)N2C)C2=CC=C(C)C=C2C)C1=O | 2 | I |
| C[C@@H]1CN(O[C@H](O1)C1=CNC2=C1C=CC=C2)C(=O)C1=CC=CC=C1 | 2 | I |
| CC(=O)NC12C[C@@H]3C[C@H](C1)CC(C3)(C2)C1=CC=C(C=C1)[N+]([O-])=O | 2 | I |
| ClC1=CC=CC=C1\C=N\N1C=NC2=C(NC3=CC=CC=C23)C1=O | 2 | I |
| CC1CCN(CC1)C(=O)N[C@H]1CN(C(=O)C1)C1=CC=C(C)C=C1 | 2 | I |
| C[C@@H]1CCCCN1C(=O)N[C@H]1CN(C(=O)C1)C1=CC=C(C)C(C)=C1 | 2 | I |
| O[C@]1([C@@H](OC2=CC=CC=C12)N1N=NC2=CC=CC=C12)C1=CC=CC=C1 | 2 | I |
| CC1=CC=C(C=C1C)C(=O)CN1C(=O)NC2(CCCCC2)C1=O | 2 | I |
| O=C(\C=C\C1=CC2=CC=CC=C2O1)N1CC(=O)NC2=C1C=CC=C2 | 2 | I |
| O=C1S[C@H](NC2=CC=CC=C2C#N)C(=O)N1C1=CC=CC=C1 | 2 | I |
| O=C1NC2(CCCCCCC2)C(=O)N1CC1=CC=CC=C1C#N | 2 | I |
| ClC1=CC=C(C=C1)[C@H]1C[C@H](NC2=NN=NN12)C1=CC=CS1 | 2 | I |
| COC1=CC2=C(C=C1OC)[C@H](NCC2)C1=CC=C2OCOC2=C1 | 2 | I |
| CC1=C2C=CC=CC2=C(N=N1)N1CCC(=CC1)C1=CNC2=C1C=CC=N2 | 2 | I |
| COC1=CC=C(C)C=C1S(=O)(=O)\N=C1/NC(C)=C(C)S1 | 2 | I |
| O=C1NC2(CCCCC2)N=C2[C@@H]3CCC[C@]12CN(CC1=CC=CO1)C3 | 2 | I |
| CCOC1=CC=CC=C1[C@@H]1CC(=O)NC2=C1C=C1OCOC1=C2 | 2 | I |
| S=C1NN=C(CC2=NOC3=CC=CC=C23)N1C1=CC=CC=C1 | 2 | I |
| [O-]C1=C(SC2=NC=NN12)[C@H]([NH+]1CCCCC1)C1=CC=CC=C1 | 2 | I |
| C\C(=C/C1=CC=C2OCOC2=C1)C(=O)[C@H]1C(=O)NC(=C)C=C1C | 2 | I |
| FC1=CC=CC=C1S(=O)(=O)NC1=CC=CC2=NSN=C12 | 2 | I |
| COC1=CC=C(C(OC)=C1)C1=C(C#N)C(=O)NC2=C1CCCC2 | 2 | I |
| FC1=CC=CC=C1NS(=O)(=O)C1=CC=CC2=NSN=C12 | 2 | I |
| CSC1=CC=C(C=C1)[C@@H]1N2N=CN=C2NC2=C1C(=O)CC(C)(C)C2 | 2 | I |
| COC(=O)[C@@H]1[C@H]2C[C@@H]3N(C\C2=C\C)[C@@H]2C[C@]11C4=C(N[C@]31O2)C=CC=C4 | 2 | I |
| C[C@H]1CN(C[C@@H](C)N1)C1=CC=C(C=C1Br)[N+]([O-])=O | 2 | I |
| C[NH+](C)CC1=C2O\C(=C/C3=CC=C(C)O3)C(=O)C2=C(C)C=C1[O-] | 2 | I |
| C[C@@H]1CCC2=C(C1)[C@@H](N=N2)[C@@H]1NC(=NO1)C1=CC=C(Cl)C=C1 | 2 | I |
| CCC1=CC=C(O1)[C@H]1CC(=O)NCC2=C1N1C=CC=C(C)C1=N2 | 2 | I |
| CCC1=CC=C(C=C1)C1=NN2C=CN=C(N3CC[C@@H](O)C3)C2=C1 | 2 | I |
| CC1=C(NC2=CC=C(C)C=C12)C(=O)N1CCN(C(=O)C1)C1=CC=CC=C1 | 2 | I |
| ClC1=CC=CC=C1CN1N=CC2=C1N=C(N2)C1=CC=CN=C1 | 2 | I |
| C[C@@H]1CCCN(C1)C(=O)NC1=CC(=CC=C1OC(F)F)C#N | 2 | I |
| C[C@H]1CCCN(C1)C(=O)NC1=CC(=CC=C1OC(F)F)C#N | 2 | I |
| C[C@@H]1CCC[C@H](C1)N(C)C(=O)NC1CCC2(CC1)OCCO2 | 2 | I |
| C[C@H]1CN(C(=O)CN1C(=O)C1=CC2=CC=CC=C2N1)C1=CC=C(C)C=C1 | 2 | I |
| C1C[C@@H](CN(C1)C1=NC=CC=N1)NC1=NC2=CC=CC=C2S1 | 2 | I |
| COC1=CC=C(Cl)C=C1CN1[C@H](C)CC(=O)N[C@@H](C)C1=O | 2 | I |
| CC1=CC2=NN\C(=N/C(=O)CC3=CC=C(Br)C=C3)N2C=C1 | 2 | I |
| ClC1=CC=CC(Cl)=C1CC1=NOC(=N1)[C@@H]1COCCN1 | 2 | I |
| C[C@H]1CC2=CC=CC=C2N1CC(=O)N1CC(=O)NC2=C1C=CC=C2 | 2 | I |
| C[C@H](NC(=O)N1[C@@H](C)CCC[C@H]1C)C(=O)N1CCCC[C@@H]1C | 2 | I |
| CC1(C)C(NC2=C(Br)C=C(C=N2)[N+]([O-])=O)C1(C)C | 2 | I |
| CN1C(=O)N[C@@](C)([C@H]2CCCN(C2)[C@@H]2CCCC3=C2C=CC=C3)C1=O | 2 | I |
| C[C@@H](CNC(=O)N1CCCC2(C1)OCCO2)C1=CC=CS1 | 2 | I |
| CC1(OCCO1)[C@@H]1CCCN(C1)C(=O)NC[C@@H]1CCC=CC1 | 2 | I |
| CC1=CC=C2NC(=NC2=C1)C(=C\C1=CC=C2OCCOC2=C1)\C#N | 2 | I |
| CC1=CN2C(S1)=NC(C)=C2\C=C/C1=NC2=CC=CC=C2NC1=O | 2 | I |
| CC1CCC(CC1)NC1=C(C=C(Br)C=N1)[N+]([O-])=O | 2 | I |
| COC1=CC=C(\C=C2/COC3=C(C=CC=C3OC)C2=O)C=C1O | 2 | I |
| C[C@H]1CN(CCN1C1=CC=C(C)C=C1)S(=O)(=O)C1=CNC=N1 | 2 | I |
| CC1=C(C(=O)CN2N=C(C)C(C)=C(C#N)C2=O)C2=C(N1)C=CC=C2 | 2 | I |
| CCS(=O)(=O)NC1=CC=C2N(C)C(=O)C(C)(C)COC2=C1 | 2 | I |
| COC1=C2CN(C[C@H](O)C2=C(OC)C=C1)C1=NC(C)=CC(C)=N1 | 2 | I |
| CC1=CC(CN2C(=O)N[C@@](C)(C2=O)C2=CC=C(F)C(F)=C2)=NO1 | 2 | I |
| COC1=CC2=C(C=C1OC)C(=O)N(C(=O)N2)C1=CC=CC=C1F | 2 | I |
| O=C(CN1CCN(CC1)C(=O)NC1CCCC1)N1CCCCC1 | 2 | I |
| OC1=C(SC2=NC=NN12)[C@H](N1CCOCC1)C1=CC=CC=C1 | 2 | I |
| CCC(=O)N1CCC2=C(C1)C(=NN2)C(=O)N(C)C1CCCCC1 | 2 | I |
| CC1=CC=C(C=C1)N1C(=O)NC(=O)\C(=C/C=C/C2=CC=CO2)C1=O | 2 | I |
| C[C@H]1[N-]C2=C\C(C=CC2=N1)=[NH+]\C(=O)CSC1=C(C=CC=N1)C#N | 2 | I |
| C1CN(CCN1)C1=CC(=CC=N1)C1=NC(=NO1)C1=CC=CS1 | 2 | I |
| CCN1CCN(CC1)C1=CC(=O)N(C(=O)N1)C1=CC=CC(C)=C1 | 2 | I |
| CC[C@@H]1CCCN(C1)C(=O)NC1CCN(CC1)S(C)(=O)=O | 2 | I |
| CC(C)(C)C(=O)N1CCC(CC1)N1C=C(N=N1)C1(O)CCCC1 | 2 | I |
| CNC1=NC=C(C=N1)S(=O)(=O)N1CCC[C@@H]2CCCC[C@H]12 | 2 | I |
| CNC1=C(C=C(C=C1Cl)C(=O)N1CCCC[C@@H]1C)[N+]([O-])=O | 2 | I |
| C[C@H]1C[C@@H](C)CN(C1)C(=O)CN1CCN(CC1)C(=O)NC1CC1 | 2 | I |
| O=C(CN1CCN(CC1)C(=O)NC1CCCCC1)N1CCCC1 | 2 | I |
| CC[C@@]1(C)NC(=O)N(CC(=O)N2[C@H](C)CC3=CC=CC=C23)C1=O | 2 | I |
| C[C@H]1CN(C[C@@H](C)O1)C1=CC=C(CNC(=O)N2CCCC2)C=N1 | 2 | I |
| ClC1=CC=C(C=C1)C1=C[C@@H](N=N1)[C@H]1NC(=NO1)C1=CC=CC=N1 | 2 | I |
| CC1=C[C@H](N=N1)[C@H]1CC(=O)NC2=NN(CC3=CC=CC=C3)C(C)=C12 | 2 | I |
| CCO[C@@H]1CCCN(C1)C(=O)NC1CCC2(CC1)OCCO2 | 2 | I |
| [O-][N+](=O)C1=CC(Cl)=C(C=C1)C(=O)N[C@@]1(CCSC1)C#N | 2 | I |
| CC(C)C[C@H]1CN(CC[C@@]1(C)O)C(=O)C1=NN2C=CC=NC2=C1 | 2 | I |
| CC1(CCOCC1)C1=NN=C(O1)C1=CC(=NN1)C1=CC=CC=C1 | 2 | I |
| CC1(CCOCC1)C1=NN=C(O1)C1=NNC(=C1)C1=CC=CC=C1 | 2 | I |
| CC1=C(NN=C1)C1(C)CCN(CC1)S(=O)(=O)C1=CC=CN=C1 | 2 | I |
| O=C(CC1CCCC1)N1CCC(CC1)C1=NNC2=NC=CN=C12 | 2 | I |
| O[C@@H]1CCCN(C1)C1=NC=C(C2=CC=CC=N2)C(=N1)C1=CC=CO1 | 2 | I |
| COC1=CC(=CC=N1)C1=NOC(=N1)C1=CC(Br)=CN1 | 2 | I |
| COC1=C(OC)C=C2C(NC(=O)N(C3CCC(C)CC3)C2=O)=C1 | 2 | I |
| CC1=NOC(CSC2=NC3=NC=C(Br)C=C3N2)=N1 | 2 | I |
| C[C@@]12[C@@H]3C[C@@H](C=C3)[C@@H]1C(=O)N(CC1=NC(=NN1)C1=CC=CC=C1)C2=O | 2 | I |
| COC[C@H]1CN(CC11CCOCC1)C(=O)C1=NNC2=CC=CC=C12 | 2 | I |
| CC(C)N1C(=O)N[C@]2(CCN(C2)[C@@H](C)C2=CC=C(C=C2)C#N)C1=O | 2 | I |
| CN1N=CN=C1CNC1=C(Cl)C=C(Cl)C2=NSN=C12 | 2 | I |
| CC1=C(C(CCl)=NN1)S(=O)(=O)N1CCO[C@H]2CCCC[C@H]12 | 2 | I |
| CC1=C(CCl)C(=NN1)S(=O)(=O)N1CCO[C@@H]2CCCC[C@H]12 | 2 | I |
| CC[C@@H]1NC(=O)C[C@@H](C)N(CC2=C(Cl)C(C)=NN2C)C1=O | 2 | I |
| CC(C)(C)OC(=O)N1[C@H]2CC[C@@H]1CN(CC2)C(=O)C1=CC=CN1 | 2 | I |
| CNC1=C(N2C=CSC2=N1)S(=O)(=O)N1C[C@H](C)[C@H](C)C1 | 2 | I |
| N#CC1=C(N2CCN(CC3=CC=NN3)CC2)C2=CC=CC=C2N=C1 | 2 | I |
| CC1=NC2=NC=C(CN3CCCC[C@@H]3C3=CNN=C3)N2C(C)=C1 | 2 | I |
| CC1=NC2=NC=C(CN3CCCC[C@H]3C3=CNN=C3)N2C(C)=C1 | 2 | I |
| CC(C)(O)C1=CN(N=N1)C1CN(C1)C1=NC2=CC=CC=C2S1 | 2 | I |
| CC(C)C1=NC(=NO1)N1C[C@@H]2C3=CC=CC=C3OC[C@]2(CO)C1 | 2 | I |
| CC(C)[C@@H]1N(CCNC1=O)C(=O)\C=C/C1=CC=C2OCOC2=C1 | 2 | I |
| CCC1=CSC(=N1)[C@H]1CCCN(C1)C1=NC=NC2=C1N=CN2 | 2 | I |
| COC1=CC=C2C(NC3=C2N=CN(\N=C\C2=CC=C(F)C=C2)C3=O)=C1 | 2 | I |
| BrC1=CC=C(O1)\C=N/NC(=O)[C@@H]1COC2=CC=CC=C2O1 | 2 | I |
| CCOC1=CC=CC=C1[C@H]1N2N=C(C)N=C2NC2=C1C(=O)C[C@@H](C)C2 | 2 | I |
| COC1=CC2=C(C[C@H](O)[C@H](O2)C2=CC=C3OCOC3=C2)C(OC)=C1 | 2 | I |
| C[C@H]1CCC2=C(C1)SC1=C2C2=NC=NN2C(NCC2=CC=CN=C2)=N1 | 2 | I |
| CC1=NOC(=C1C)C1=CNN=C1[C@@H]1CCCN1C(=O)C1CCCC1 | 2 | I |
| CN1C(=O)N(C)C2=CC(\C=C\C3=CC(=NC(=O)N3)C(F)(F)F)=CC=C12 | 2 | I |
| O=C(N1CCO[C@H](CC2CCCCC2)C1)C1=CC2=C(NN=N2)C=C1 | 2 | I |
| O=C1NC2(CCCCCC2)C(=O)N1CN1CCC[C@@H]1C1=CN=CC=C1 | 2 | I |
| O=C1NC(=CC(=O)N1C1=CC=CC=C1)N1CCN(CC1)C1=CC=CC=C1 | 2 | I |
| [O-]C1=NC(=O)N(CC2=CC=CC=C2)C(=O)C1=CC1=C2C=CC=CC2=[NH+]C1 | 2 | I |
| O=C(N[C@@H]1CCCN(C1)C1=NC=CC=N1)[C@H]1COC2=CC=CC=C2C1 | 2 | I |
| O=C(N[C@H]1CCCN(C1)C1=NC=CC=N1)[C@H]1COC2=CC=CC=C2C1 | 2 | I |
| CC1=NN2C(SC([C@H]([NH+]3CCOCC3)C3=C(C)C=CS3)=C2[O-])=N1 | 2 | I |
| C[C@]1(CCS(=O)(=O)C1)NC1=CC=C(Br)C=C1[N+]([O-])=O | 2 | I |
| O=C1C[C@H](CN1C1=CC=CC=C1)C1=NOC(=N1)C1=CC=C2C=CNC2=C1 | 2 | I |
| O=C(N1CC(C1)C1=NC(=NO1)C1=CC=CS1)C1=CNC2=CC=CC=C12 | 2 | I |
| C[C@H]1C[C@@H](C)CN(C1)C(=O)CN1CCN(CC1)C(=O)NC1CCCC1 | 2 | I |
| CC1=CS\C(N1)=N/S(=O)(=O)C1=CC=C(C=C1)N1CCCCC1=O | 2 | I |
| CCN1[C@H](NC2=CC(Cl)=CC=C2C1=O)C1=CC=CC=C1[N+]([O-])=O | 2 | I |
| C[C@H](NC(=O)N1CCN(CC1)C(=O)C1=CC=C(C)S1)[C@H]1CCCO1 | 2 | I |
| CC1=CC=C2C(=O)N(CC(=O)N3CCC[C@H]3C3=CC=CN3)C(=O)C2=C1 | 2 | I |
| CC1=NN2C(S1)=[NH+]C(C)=C2S(=O)(=O)[N-]C1=CC(C)=CC=C1C | 2 | I |
| C[C@@H](N1C(=O)NC2=C(SN=C2C2=CC=CC=N2)C1=O)C1=CC=CC=C1 | 2 | I |
| CCOC1=CC=C(C=N1)[C@@H]1CC(=O)NCC2=C1N1C=CC(C)=CC1=N2 | 2 | I |
| O[C@H]1CN(C[C@@H]1N1CCCC1)C1=NC2=C(C=N1)N(CC2)C1=CC=CC=C1 | 2 | I |
| ClC1=C(CN2CCCN(CC2)C2=NC3=CC=CC=C3N2)N=NS1 | 2 | I |
| CC1=C(SC(=N1)C1=NNC=N1)C(=O)N1CCC[C@@H]1C1=CC=CS1 | 2 | I |
| O[C@@H]1CCCN(C1)C1=C(C=CC=N1)C1=NC(=NO1)C1=CC=CS1 | 2 | I |
| CC(C)C[C@@H]1COCCN1C(=O)C1=C2O[C@H](C)C(=O)NC2=CC=C1 | 2 | I |
| CC(C)(C)C(=O)N1CCC(CC1)C(=O)C1=CC=C2OCC(=O)NC2=C1 | 2 | I |
| C[C@@H](C(=O)N1CCC[C@H](C1)C1=CC=NN1)C1=CC=C2OCCOC2=C1 | 2 | I |
| C[C@H]1CCCN(C1)C1=NC2=C(CC[NH+](CC3=CC=CO3)C2)C([O-])=N1 | 2 | I |
| CC1=CC=C2OC(=O)N(CN3CCN(CC3)C3=CC=CC=C3O)C2=C1 | 2 | I |
| CC[C@]1(C)NC(=O)N(CC(=O)N2CCCC3=C2C(F)=CC(C)=C3)C1=O | 2 | I |
| CC1(C)[C@H](NC(=O)C2=CC=C(C=C2)N2CCOC2=O)[C@H]2CCO[C@H]12 | 2 | I |
| CC1=CC=C(C)C([N-]S(=O)(=O)C2=C(C)[NH+]=C3SC=CN3C2=O)=C1 | 2 | I |
| O=C(NC1CCCCCC1)[C@H]1CN(C(=O)C1)C1=CC2=C(OCO2)C=C1 | 2 | I |
| CN1C[C@H](CCC1=O)NC1=CC(Br)=CC=C1[N+]([O-])=O | 2 | I |
| C1CC1C1=NSC(=N1)N1CCCN(CC1)C1=NC2=CC=CC=C2N1 | 2 | I |
| O=C(N[C@H]1CCN(C1=O)C1=CC=CC=C1)N1CCOC[C@@H]1C1CC1 | 2 | I |
| CN1N=CC=C1CN1C(=O)N[C@@](C)(C1=O)C1=CC=C2C=CC=CC2=C1 | 2 | I |
| C[C@@H]1[C@@H](CC[NH+]1C1=CC(F)=C(Br)C=C1[N+]([O-])=O)C([O-])=O | 2 | I |
| C[C@@H](NC1=NC=NC2=C1C(C)=C(C)S2)C1=CN2N=CSC2=N1 | 2 | I |
| CC(C)[C@@H](NC(=O)N1C[C@H](C)S[C@H](C)C1)C1=NN=C2CCCN12 | 2 | I |
| CC1=CC=C(C=C1C)N1C(=O)NC(=O)\C(=C\C=C/C2=CC=CO2)C1=O | 2 | I |
| COC1=CC=CC(=C1)[C@@H]1CC(O)=NC2=C1SC(=N2)N1CCOCC1 | 2 | I |
| CN(\N=C/C1=CC=C(Cl)C(Cl)=C1)C1=CC(=O)N(C)C(=O)N1 | 2 | I |
| C[C@H]1CN(C[C@@H](C)O1)C(=O)NCC(C)(C)N1C[C@H](C)O[C@H](C)C1 | 2 | I |
| [O-][N+](=O)C1=CC(Br)=C(F)C=C1N[C@H]1CS(=O)(=O)C=C1 | 2 | I |
| COC1=CC=CC=C1N1C(=S)S\C(=C/C2=NC3=CC=CC=C3N2)C1=O | 2 | I |
| C[C@@]12N(CCC3=C1NC1=CC=CC=C31)C(=O)CN(CC1=CC=CC=C1Cl)C2=O | 2 | I |
| CC1=CC(C)=C2C(OC3=C([C@H](N(CCO)C3=O)C3=CC=CC=C3F)C2=O)=C1 | 2 | I |
| C[C@H]1CCCC[C@@]11NC(=O)N(CC2=CSC(=N2)C2=CC=CS2)C1=O | 2 | I |
| O=C(N1CCC2=C(C=CS2)[C@@H]1C1=CC=CC=C1)C1=CC=C2NC(=O)COC2=C1 | 2 | I |
| CC1=C(\C=C2\SC(=S)N(C2=O)C2=CC=C3OCOC3=C2)C2=CC=CC=C2N1 | 2 | I |
| CN1C(=O)C(C)(C)C2=CC(=CC=C12)S(=O)(=O)NC1=CC=C(C)C(F)=C1 | 2 | I |
| C[C@H]1CCCC[NH+]1CC1=C([O-])C=C(C)C2=C1O\C(=C/C1=CC=CN=C1)C2=O | 2 | I |
| COC1=CC=C(C=C1)[C@@H]1N2[C@@H](CC3=C1NC1=CC=CC=C31)C(=O)N(C)C2=S | 2 | I |
| COC1=CC=C(C=C1)[C@@H]1N2[C@H](CC3=C1NC1=CC=CC=C31)C(=O)N(C)C2=S | 2 | I |
| O=C1CCC2=CC(=CC=C2N1)S(=O)(=O)N1CCCSC2=CC=CC=C12 | 2 | I |
| OC1=CC=CC=C1C(=O)N1CCN(CC1)C(=O)C1=CC2=C(F)C=CC=C2S1 | 2 | I |
| BrC1=CC=C(C=C1)C1=CC=C(O1)\C=N/N=C1\NC(=O)CS1 | 2 | I |
| COC1=CC=CC=C1N1CCN(CC1)[C@@H]1SC2=CC=CC=C2NC1=O | 2 | I |
| CC1=C(SC(Cl)=N1)S(=O)(=O)\N=C1/NC=C(Br)C=C1 | 2 | I |
| BrC1=NC([N-]S(=O)(=O)C2=CC=CC3=C[NH+]=CC=C23)=CC=C1 | 2 | I |
| COC1=CC=C(Br)C=C1S(=O)(=O)\N=C1/NC=CC(C)=C1 | 2 | I |
| ClC1=CC=C(CN2C(=O)N[C@@]3(CCOC4=CC=CC=C34)C2=O)C=C1Cl | 2 | I |
| BrC1=CC=C(C=C1)S(=O)(=O)N1CC(=O)NC2=CC=CC=C12 | 2 | I |
| CN(C)C(=O)[C@@H]1[C@H]2NC(=S)N(C3=CC(C)=CC=C3)[C@]1(C)OC1=CC=CC=C21 | 2 | I |
| CN(C)C(=O)[C@@H]1[C@H]2NC(=S)N(C3=CC=CC=C3C)[C@]1(C)OC1=CC=CC=C21 | 2 | I |
| CC[C@]1(C)CC2=C(CO1)C=C1C(=O)N(C(=S)NC1=N2)C1=CC=C(C)C=C1 | 2 | I |
| COC1=CC=C(C=C1)N1C(=O)[C@@H]2[C@@H]3C[C@H]4[C@H](O[C@]1([C@H]24)C1=CC=CC=C1)[C@@H]3O | 2 | I |
| C[C@@H]1CCCC[C@@H]1NC(=O)CN1C(=O)OC2=CC(Br)=CC=C12 | 2 | I |
| CC1=NN(C2=C1[C@@H](CC(=O)N2)C(=O)C1=CC=CS1)C1=CC=C(C)C(C)=C1 | 2 | I |
| C[C@H]1CC2=CC=CC=C2N1C(=O)C1CCN(CC1)C(=O)NC1=CC=CS1 | 2 | I |
| C[NH+]1CCN(CC2=C([O-])C=C(C)C3=C2O\C(=C/C2=CC=CC=C2)C3=O)CC1 | 2 | I |
| COC1=C(OC)C=C(NC(=O)N2CCC[C@@H](C)C2)C(Br)=C1 | 2 | I |
| C[C@@H]1CN(C[C@@H](C)O1)C(=O)C1CCN(CC2=C(O)C=C(C)C(C)=C2)CC1 | 2 | I |
| C[C@@H]1CCC[NH+](CC2=C([O-])C=C(C)C3=C2O\C(=C\C2=CC=NC=C2)C3=O)C1 | 2 | I |
| FC1=CC=CC(=C1)[C@H]1C[C@H](NC2=NN=NN12)C1=CC=CC(Br)=C1 | 2 | I |
| [O-]C1=N[C@H]([NH+]2CCCC[C@@H]12)C1=CN(N=C1C1=CC=CS1)C1=CC=CC=C1 | 2 | I |
| C[C@@H]1CCCN(C1)S(=O)(=O)N1CCC(=CC1)C1=CNC2=C1C=CC=C2 | 2 | I |
| C[C@@H]1SC2=CC=C(C=C2NC1=O)S(=O)(=O)N(C)C1=CC=C(C)C=C1 | 2 | I |
| CN1C2=C([C@H](N=C1O)C1=CC=CC=C1)C(=O)N(C2)C1=CC=C2C=CC=CC2=C1 | 2 | I |
| C[C@@H](N1CCOCC1)C1=CC=C(NC(=O)N2CCC[C@H]3CCC[C@H]23)C=C1 | 2 | I |
| CCN1CCC2=C(NC=N2)C11CCN(CC1)C(=O)C1=C2CCCCC2=CS1 | 2 | I |
| CCN1\C(NC2=CC(Br)=CC=C12)=N\C(=O)C1=C(C)N=C(C)S1 | 2 | I |
| CCN1\C(NC2=CC(Br)=CC=C12)=N\C(=O)[C@@H]1CC11CCOCC1 | 2 | I |
| CCN1\C(NC2=CC(Br)=CC=C12)=N\C(=O)[C@@H]1CCCN(C)C1 | 2 | I |
| CC1=CC(C)=C2C=C(NC2=C1)C(=O)N1CC[C@@H]2[C@H](CCC(=O)N2C2CC2)C1 | 2 | I |
| CN1C=C(\C=C2\SC(=O)N\C\2=N/C2=CC=CC(Br)=C2)C=N1 | 2 | I |
| O=C(NC1CC1)N1CCC2(CC1)C(=O)N(C1=CSC=C1)C1=CC=CC=C21 | 2 | I |
| CC1=CC=C(C)C(=C1)N1CCN(CC1)C(=O)\C=C1\SC2=CC=CC=C2NC1=O | 2 | I |
| OC1=NC2=C(N=CN2C2=CC=CC=C2Br)[C@@H](C1)C1=CC=C(C=C1)C#N | 2 | I |
| COC1=CC=CC=C1[C@H]1CC(O)=NC2=C1N=CN2C1=CC(F)=CC(F)=C1 | 2 | I |
| CC(C)C1(O)CN(C1)C1=CC=C(I)C=C1[N+]([O-])=O | 2 | I |
| C[C@H]1OC2=CC=CC=C2C=C1\C=C1\OC2=C(CN(C)C)C(O)=CC=C2C1=O | 2 | I |
| BrC1=CC(OCC(=O)N2CC(=O)NC3=CC=CC=C23)=CC=C1 | 2 | I |
| CCC1=C(I)C(=O)NC(=N1)C1=CC=C2N=CC=NC2=C1 | 2 | I |
| COC1=CC=CC=C1N1C(=O)S\C(=C\C2=CC=C(O)C=C2)C1=O | 2 | I |
| CC[C@@]12C[C@]1(C(=O)OC)C(=O)N1CCC3=C(NC4=CC=CC=C34)[C@H]21 | 2 | I |
| CC1=CC=C(O1)\C=C1\C(=O)NC(=S)N(C1=O)C1=CC=CC=C1Cl | 2 | I |
| CC1=C(\C=N/N2C(=O)[C@@H]3[C@H]([C@@H]4C=C[C@H]3[C@H]3C[C@@H]43)C2=O)C2=CC=CC=C2N1 | 2 | I |
| CC(C)(C)N1C(=O)NC(=O)\C(=C/C2=CC=C(Br)S2)C1=O | 2 | I |
| C[C@@H]1CC2=CC(C[NH+]3CCC4=C(CC3)C([O-])=NC(C)=N4)=CC=C2O1 | 2 | I |
| CC1=CC(=C(C)S1)S(=O)(=O)NC1=CC=C2OCCOC2=C1 | 2 | I |
| CN1C=[NH+]C(=C1Cl)S(=O)(=O)[N-]C1=CC=C(F)C(F)=C1F | 2 | I |
| CN1N=CC2=C1N=CN=C2NCC1=CC=C(Br)S1 | 2 | I |
| O=C(N1C[C@H](C2=CC=CC=C2)C2=C(C1)C=CC=C2)C1=NNC(=O)C=C1 | 2 | I |
| FC(F)(F)C(=O)NN1C(=O)C2=CC=C3CCC4=C3C2=C(C=C4)C1=O | 2 | I |
| COC(=O)[C@H]1[C@@H](O)CC[C@H]2CN3CCC4=C(N=C5C=CC=C[C@H]45)[C@@H]3C[C@H]12 | 2 | I |
| CC1(C)[C@@H]2CC[C@@]1(C)[C@@H](C2)N1C[C@]23O[C@H](C=C2)[C@@H]([C@@H]3C1=O)C(O)=O | 2 | I |
| CSC1=NC(=O)[C@@H]2[C@H](CC(=O)NC2=N1)C1=C(Cl)C=CC=C1Cl | 2 | I |
| O=C([C@H]1CC=CC[C@@H]1C1=NC2=CC=CC=C2S1)N1CCNC(=O)C1 | 2 | I |
| CC1=C(SC(=O)N1)S(=O)(=O)N1CC[C@@H](C1)C1=CC=CC=C1 | 2 | I |
| COC1=CC=C(C=C1Br)C1=NN=C2[C@H](C)NCCN12 | 2 | I |
| [O-]\C=C1/CCCC[NH+]1CC1=CC(=O)N2C=C(Br)C=CC2=N1 | 2 | I |
| [O-][N+](=O)C1=CC=C(\C=C(\Cl)/C=C2\SC(=O)NC2=S)C=C1 | 2 | I |
| O=C(N1CCC[C@H]1C1CCCCC1)C1=CC2=C(CCCC2=O)NC1=O | 2 | I |
| CC1=CC(=CC(C)=C1)N1CCC(CC1)N1CCN2CCNC(=O)[C@@H]2C1 | 2 | I |
| C[C@H]1C[C@@H]1C(=O)N1CCN(CC1)C(=O)CC1=CNC2=C1C=CC=C2 | 2 | I |
| CC1=NC=C(N1)S(=O)(=O)N1CCC[C@H]1C1=CC=CC=C1Cl | 2 | I |
| O=C1NC(CN2CCO[C@@H]3CCCC[C@H]23)=NC2=C1C1=C(CCC1)S2 | 2 | I |
| C[C@@H]1CN(C[C@@H](O1)C1=CC=CC=C1)C(=O)C1=CC=C2NC(=S)OC2=C1 | 2 | I |
| CC1=C[C@@H]2[C@H]3[C@@H]([C@@H](N2C2=C1C=CC=C2)C(=O)C(C)(C)C)C(=O)NC3=O | 2 | I |
| CC1=C[C@H]2[C@H]3[C@@H]([C@@H](N2C2=C1C=CC=C2)C(=O)C(C)(C)C)C(=O)NC3=O | 2 | I |
| FC(F)(F)CN1CCCN(CC1)C(=O)NC1=CC=C(C=C1)C#N | 2 | I |
| C[C@H]1SCCN([C@@H]1C)C(=O)C1=CC(=O)N(N1)C1=CC=C(C)C=C1 | 2 | I |
| O=C([C@@H]1CCS(=O)(=O)C1)N1CCCC[C@@H]1C1=CC2=CC=CC=C2N1 | 2 | I |
| CN1C(Cl)=[NH+]C=C1S(=O)(=O)[N-]C1=CC=C(F)C(F)=C1F | 2 | I |
| CC(C)(C)C1=CC=C(C=C1)N1CC(=O)N[C@H]2CS(=O)(=O)C[C@H]12 | 2 | I |
| C[C@H]1CCCC[C@H]1NS(=O)(=O)C1=CC=C2N(C)C(=O)CC2=C1 | 2 | I |
| ClC1=CC(=CNC1=O)S(=O)(=O)N1CCC2(CCCC2)CC1 | 2 | I |
| BrC1=CC=C(O1)\C=C1\C(=O)NN(C1=O)C1=CC=CC=C1 | 2 | I |
| CCOC1=CC(\C=C2/N=C(C)OC2=O)=CC(Br)=C1O | 2 | I |
| COC1=CC=C(C=C1Br)N1[C@H](C)C(=O)NC(C)(C)C1=O | 2 | I |
| ClCC1=CNN=C1S(=O)(=O)N1CCC2=CC=CC=C2CC1 | 2 | I |
| FC1=CC=C2OC[C@H](CC2=C1)C(=O)N1CCNC(=O)[C@H]1C1=CC=CC=C1 | 2 | I |
| CCC1=C(Br)C(=O)NC(=N1)[C@@H]1COC2=CC=CC=C2O1 | 2 | I |
| CC1=CC=C2NC3=C(CN(CC3)C(=O)[C@H]3CN(C4CC4)C(=O)C3)C2=C1 | 2 | I |
| ClC1=CC(Cl)=C(C(Cl)=C1)S(=O)(=O)N1CCNC[C@@H]1C#N | 2 | I |
| CC(C)N1CCN(CC1)C1=CC2=C(C=C1Br)C(=O)C(=O)N2 | 2 | I |
| CC(C)C1=C(Br)C([O-])=NC(=N1)[C@@H]1CN(C)CC[NH+]1C | 2 | I |
| CC(C)C1=C(Br)C([O-])=NC(=N1)[C@H]1CN(C)CC[NH+]1C | 2 | I |
| CC1=C(Br)SC(=C1)S(=O)(=O)N1CCCNC(=O)C1 | 2 | I |
| C1CC2=C(C1)C1=C(N=CN=C1S2)N1CCC[C@H](C1)C1=NC=CN1 | 2 | I |
| O=C(C1CCOCC1)N1CCC2(C[C@@H](C(=O)N2)C2=CC=CC=C2)CC1 | 2 | I |
| FC1=CC=C(\C=C2\C(=O)N=C3SC(=NN3C2=N)C2=CC=CC=C2)C=C1 | 2 | I |
| O[C@@H]1[C@@H](CC2=CC=CC=C12)N1CCN2C(C1)=NN=C2C1=CC=CC=C1 | 2 | I |
| OC1=CC=CC=C1N1CCN(CC1)C1=CC(=O)N(C1=O)C1=CC=CC=C1 | 2 | I |
| COC1=CC=C2NC3=C(N=C(C)N(\N=C\C4=CC=CC=C4)C3=O)C2=C1 | 2 | I |
| COC1=CC=C(C=C1OC)[C@@H]1NC(=O)C2=C1C(=O)C1=CC=CC=C1O2 | 2 | I |
| CCC1CCC2(CC1)NC(=O)N(CC1=CN3C=CC=CC3=N1)C2=O | 2 | I |
| CC1=CC=C(C[NH+]2CCC3=C(C2)C([O-])=NC(=N3)N2CCOCC2)C=C1 | 2 | I |
| COC(=O)C1=C(C)NC2=C([C@H]1C1=CN(C)N=C1C)C(=O)CC(C)(C)C2 | 2 | I |
| COC1=C(O)C(\C=N\N2C=NC3=C(C(C)=C(C)S3)C2=O)=CC=C1 | 2 | I |
| O=C1N=C(NN2C(=O)C3=CC=CC=C3N=C12)SCC1=CC=CC=C1 | 2 | I |
| CC1=CC=C2NC3=C(N=NC(SCC(=O)N4CCCC4)=N3)C2=C1 | 2 | I |
| CC1=CC=C(NC(=O)[C@@H]2CCN(C2=O)C2=CC=C3OCOC3=C2)C=C1 | 2 | I |
| COC1=CC=C([C@@H]2CC(=O)NC3=C2C=C2OCOC2=C3)C(OC)=C1 | 2 | I |
| COC1=CC=C(C=C1OC)N1C(=O)N[C@H]2C[C@]1(C)OC1=CC=CC=C21 | 2 | I |
| C[C@@H]1NC2=CC=CC=C2C(=O)N1\N=C\[C@@H]1C=NN=C1C1=CC=CC=C1 | 2 | I |
| CC1=CC=CC(N2CN(CCO)CN3C(=O)C(C)=C(C)N=C23)=C1C | 2 | I |
| CNC(=O)[C@H]1CCCCN1C1=CC=C(Br)C=C1[N+]([O-])=O | 2 | I |
| CNS(=O)(=O)C1=CC=C(O1)C(=O)N1CC[C@H]2CCCC[C@@H]2C1 | 2 | I |
| C[C@H](NC(=O)N1CCN(CC1)C(=O)C1=CC=CO1)C1=CC=CC=C1 | 2 | I |
| COC1=CC=C(NC(=O)N2CCC[C@H](C)C2)C=C1N1CCCCC1=O | 2 | I |
| C[C@@H](Br)[C@H](C)C1=CC2=C(C=C1[N+]([O-])=O)C(=O)NC=N2 | 2 | I |
| C1CCC(C1)N1N=CC2=N[C@@H](N[C@@H]12)C1=CC=C(O1)[C@H]1CCCCO1 | 2 | I |
| O=C1C[C@@H](C2=CC3=CC=CC=C3O2)C2=C(N1)N(CC1=CC=CN=C1)N=C2 | 2 | I |
| CC1=C(OC2=C1C=CC=C2C)C(\[O-])=N\S(=O)(=O)C1=CC=C[NH+]=C1 | 2 | I |
| COC1=CC2=NC(=NC(C)=C2C=C1)N1C[C@H](O)[C@H](C1)N1CCCCC1 | 2 | I |
| COC(=O)[C@@H]1CC2=CC=CC=C2N1C(=O)C1=CC(=O)NC2=C1C=CC=C2 | 2 | I |
| O=C(CSC1=NC=C2C=CC=CN12)N1CC(=O)NC2=CC=CC=C12 | 2 | I |
| CN(CC1=C(C)ON=C1C)C(=O)C1=CC=C2CC(C)(C)OC2=C1O | 2 | I |
| CC(=O)C1=CNC(=C1)C(=O)N1CCC[C@@H]1C1=CC=C2OCOC2=C1 | 2 | I |
| O[C@H]1CCN(C1)C1=NC=CC=C1C1=NC(=NO1)C1=CC(F)=CC=C1 | 2 | I |
| C[C@@H]1CCCC[C@H]1NC(=O)C1CN(C1)C(=O)C1=CC=C2OCOC2=C1 | 2 | I |
| [O-]C1=CC(C[NH+]2CCCC[C@@H]2C2=CC(F)=CC=C2)=NC2=NC=NN12 | 2 | I |
| C(N1C=CN=C1C1=NN2CCNCC2=C1)C1=CC2=C(OCCC2)C=C1 | 2 | I |
| CC1=NNC2=C1N=CN=C2N1CCN(CC2=CC=C(Cl)C=C2)CC1 | 2 | I |
| C[C@H]1CN(C(=O)CNC(=O)C2=CC=C3OCOC3=C2)C2=C1C=CC=C2 | 2 | I |
| CN(C)S(=O)(=O)C1=CC=C(C=C1)C1=NC2=C(C(C)=CS2)C(=O)N1 | 2 | I |
| C[C@@H]1CN(CCO1)C1=CC=C(CNC(=O)N2CCCC2(C)C)C=N1 | 2 | I |
| CC[C@@H]1CCCN(C1)C(=O)NC1CCN(CC1)C(=O)C1=CC=CO1 | 2 | I |
| COC1=CC=CC([C@@H]2CC(=O)C3=CC4=C(OCO4)C=C3N2)=C1OC | 2 | I |
| C[C@H]1CN(CCO1)C1CCN(CC1)C(=O)NCC1=CC=C(C=C1)C#N | 2 | I |
| COC1=CC=C(C=C1)[C@@H]1C[C@@H](N2N=NN=C2N1)C1=CC=C(F)C=C1 | 2 | I |
| CC1=CC=C(C=C1)N1C(=O)C[C@H](NC2=CC=C3OCCOC3=C2)C1=O | 2 | I |
| CC[C@H]1CN(C)C2=CC=CC=C2C[NH+]1CC1=NC2=CC=NN2C([O-])=C1 | 2 | I |
| CC1=CC=C(C=C1)N1N=NN=C1N[C@H]1CCO[C@]2(CCSC2)C1 | 2 | I |
| CN(C)C(=O)N1C[C@@H]2CC[C@H](C1)N(CC1=NNC(=C1)C(C)(C)C)C2 | 2 | I |
| C[C@H]1OCCN([C@@H]1C)C(=O)NCC1=CC=CC=C1N1CCN(C)CC1 | 2 | I |
| CC1=CC=C(NC2=C3CCN(CCC3=NC=N2)S(C)(=O)=O)C=C1 | 2 | I |
| CC1=CN=C(N=C1)N1CC[C@]2(C[C@@H](CO2)NC2=C(F)C=CC=N2)C1 | 2 | I |
| O=C(NC1=CN(C[C@@H]2CCCO2)N=C1)N1CCC2(CCCC2)CC1 | 2 | I |
| CC1=CC=C(C=C1C)N1C(=O)NC(=O)\C(=C\C=C\C2=CC=CO2)C1=O | 2 | I |
| CCNC(=O)N1CCN(CC(=O)N2CCC[C@H]3CCCC[C@H]23)CC1 | 2 | I |
| O=C(\N=C1/NN=C2C=CC=CN12)[C@@H]1CCCN(CC2=CC=CC=C2)C1 | 2 | I |
| CC1(C)OC2=CC=C(C=C2N(CC#N)C1=O)C(=O)NC1CCCC1 | 2 | I |
| C[C@@]1(NC(=O)N(CC2=CC=CC=C2F)C1=O)C1=CC=C2OCOC2=C1 | 2 | I |
| C[C@@H](N1C(=O)NC2(CCCCC2)C1=O)C(=O)N(C)C1=CC=CC=C1 | 2 | I |
| O=C1NC2(CCCCC2)C(=O)N1CC1=NC=C(O1)C1=CC=CC=C1 | 2 | I |
| CC1=CC(CN2C(=O)N[C@](C)(C2=O)C2=CC=CC3=C2C=CC=C3)=NO1 | 2 | I |
| CN1CCOC2=CC=C(NS(=O)(=O)C3=CC=C(C)C=C3)C=C2C1=O | 2 | I |
| CC1=CC(O)=C([C@@H]2OC(=O)C3=CC=CC=C23)C(=O)N1C[C@H]1CCCO1 | 2 | I |
| CC[C@@H](C)N1C(S)=NC(=O)\C(=C\C2=NC3=CC=CC=C3N2)C1=O | 2 | I |
| O=C1CN(CCN1)C1=NC=C(C2=CSC=C2)C(=N1)C1=CN=CC=C1 | 2 | I |
| CCOC1=CC=CC=C1[C@@H]1N2N=C(C)N=C2NC2=C1C(=O)C[C@H](C)C2 | 2 | I |
| COC1=CC=C2NC(=O)C(\C=C3\N=C(OC3=O)C3=CC=CC=C3)=CC2=C1 | 2 | I |
| O=C(CN1C(=O)[C@H]2CCCN2C1=O)NC12CC3CC(CC(C3)C1)C2 | 2 | I |
| CC[C@@H](N1C(=O)NC2=CC=CC=C2C1=O)C(=O)N1CCCCCC1 | 2 | I |
| COC1=CC=CC=C1\C=C1\C(=O)NC(=O)N(C1=O)C1=CC=CC=C1C | 2 | I |
| CN1CCN(CC1)[C@H](C1=C(O)N2N=CN=C2S1)C1=CC=C(C)C=C1 | 2 | I |
| OC1=C(SC2=NC=NN12)[C@@H](N1CCOCC1)C1=CC=C(F)C=C1 | 2 | I |
| C[C@H]1CCCC[C@@]11NC(=O)N(CC(=O)C2=CC=C3OCCC3=C2)C1=O | 2 | I |
| CSC1=NN=C(C)C(=O)N1\N=C\C1=C(O)C=CC2=CC=CC=C12 | 2 | I |
| O=C(NN1C(=O)C2=CC=CC=C2N=C1C1=CC=CS1)C1=CC=CO1 | 2 | I |
| C[C@H]1C[C@@H](C)CN(C1)C(=O)NCC1=CC=CN=C1N1CCOCC1 | 2 | I |
| C[C@@H]1C[C@@H](C)CN(C1)C(=O)NCC1=CC=CN=C1N1CCOCC1 | 2 | I |
| O=C1C[C@H](CN1C1=CC=CC=C1)C1=NOC(=N1)C1=CC2=CC=CC=C2N1 | 2 | I |
| CC1=CS\C(N1)=N/S(=O)(=O)C1=CC=C(C=C1)N1CCCC1=O | 2 | I |
| [O-][N+](=O)C1=CC(C(=O)N[C@H]2CCC[C@@H]2C#N)=C(Br)C=C1 | 2 | I |
| N1[C@H](ON=C1C1=CC=CN=C1)[C@@H]1C=C(N=N1)C1=CC2=CC=CC=C2C=C1 | 2 | I |
| C1CCC(C1)N1N=CC2=N[C@H](N[C@@H]12)C1=CC=C(O1)[C@@H]1CCCCO1 | 2 | I |
| CC1=CC=CC=C1N1NC2=CC(=O)N(CC3=CC=CC=N3)C(C)=C2C1=O | 2 | I |
| O=C1C[C@H](C2=CC3=CC=CC=C3O2)C2=C(N1)N(CC1=CC=CN=C1)N=C2 | 2 | I |
| COC1=CC=C(C=N1)[C@H]1CC(=O)NC2=C1C(C)=NN2C1=CC=C(C)C=C1 | 2 | I |
| ClC1=CN2C(=O)C=C(CN3CCC(CC3)C3=NNC=C3)N=C2C=C1 | 2 | I |
| CN1C(=O)NN=C1SCC1=CN2C=C(Cl)C=C(Cl)C2=N1 | 2 | I |
| FC1=CC(OCC(=O)N2CC(=O)NC3=CC=CC=C23)=CC=C1C#N | 2 | I |
| FC1=CN=C(N=C1N1CCOCC1)C1=CC=C(C=C1)C1=CC=NN1 | 2 | I |
| [O-]C1=NC2=C(C=C1C#N)C(=O)N(CC1(CCCC1)[NH+]1CCCC1)C=C2 | 2 | I |
| CCC1=CC(=NC=N1)N1CC[C@H]([C@H](O)C1)C1=CC=C2OCOC2=C1 | 2 | I |
| O[C@@H]1CCCN(C1)C1=CC(=CC=N1)C1=NC(=NO1)C1=CC=CS1 | 2 | I |
| O=C(COC1=CC2=C(NC(=O)CC2)C=C1)N1CCO[C@H]2CCCC[C@@H]12 | 2 | I |
| CC(C)(C)C1=NN2C(NC3=C(CCN(CC4=COC=C4)C3)C2=O)=C1 | 2 | I |
| O=C1S\C(=C/C2=CC=[NH+]C=C2)C([N-]C2=CC3=C(OCO3)C=C2)=N1 | 2 | I |
| CC1=C(N=NN1C1=CC=CC=C1)C1=NNC(=S)N1C1=CC=C(C)C=C1 | 2 | I |
| C[C@@]1(NC(=O)N(CC2=NOC=C2)C1=O)C1=CC=C(Cl)C(Cl)=C1 | 2 | I |
| C[C@@H](N1C(=O)C2=CC=CC=C2C1=O)C(=O)N[C@@H]1[C@@H]2CCO[C@@H]2C1(C)C | 2 | I |
| C[C@H]1C[C@@H]2CCCC[C@@H]2N1C(=O)CN1C(=O)N[C@](C)(C2CC2)C1=O | 2 | I |
| O=C(CN1C(=O)C2=CC=CC=C2C1=O)NC1=CC=CC2=C1N=CC=C2 | 2 | I |
| COC1=CC=C(C=C1OC)[C@@H]1CC(=O)C2=CC3=C(OCO3)C=C2N1 | 2 | I |
| C[C@]1(CC[NH+](C1)C1=CC(Br)=CC=C1[N+]([O-])=O)C([O-])=O | 2 | I |
| [O-][N+](=O)C1=CC(Br)=C(N[C@H]2CN3CCC2CC3)N=C1 | 2 | I |
| ClC1=CC=CN\C1=N/S(=O)(=O)C1=CN(N=C1)C1=CC=CC=C1 | 2 | I |
| CNC1=C(Br)C=NC(=N1)N1CCC[C@@H](C1)N1C=CC=N1 | 2 | I |
| CC(C)C1=NN=C2CC[C@@H](CN12)NC(=O)C1=CC=C2N=CSC2=C1 | 2 | I |
| C[C@]1(NC(=O)N(CC(=O)N2CCCC2)C1=O)C1=CC=C2OCOC2=C1 | 2 | I |
| C[C@]12O[C@](CO)(C=C1)[C@@H]1[C@@H]2C(=O)N(C1=O)C1=CC=C(C=C1)[N+]([O-])=O | 2 | I |
| CCN1C2=CC=C(C=C2[C@](O)(CC(=O)C2=CC=CC=N2)C1=O)[N+]([O-])=O | 2 | I |
| CC1=C(C(=O)OCC(=O)N2CC(=O)NC3=C2C=CC=C3)C(C)=NO1 | 2 | I |
| CCCN1N=NN=C1CN1C(=O)N[C@]2(CCC3=C2C=CC=C3)C1=O | 2 | I |
| CC1=CC(CN2C(=O)N[C@](C)(C2=O)C2=CC=C3OCCOC3=C2)=NO1 | 2 | I |
| CNS(=O)(=O)C1=CC=C(N2C[C@H](C)O[C@@H](C)C2)C(=C1)[N+]([O-])=O | 2 | I |
| OC1=NC(=NC(=C1)[C@H]1CCN(C1)C(=O)[C@@H]1CCOC1)N1CCOCC1 | 2 | I |
| CC(C)OC(=O)C1=C(C)NC2=NC=NN2[C@H]1C1=CN(C)N=C1C | 2 | I |
| OC[C@@]12O[C@@H](C=C1)[C@@H]1[C@@H]2C(=O)N(C1=O)C1=CC(=CC=C1)[N+]([O-])=O | 2 | I |
| CCC1=C(N=CO1)C(=O)NC[C@H]1CCN(C1)C1=CC(=O)N(C)N=C1 | 2 | I |
| C[C@H]1CCCC[C@@]11NC(=O)N(CCN2N=C3C=CC=CN3C2=O)C1=O | 2 | I |
| O=C1NC(CCN2C=NC3=C(N=CC=N3)C2=O)=NC2=CC=CC=C12 | 2 | I |
| [O-][N+](=O)C1=CN(CC(=O)C2=CC3=C(NC(=O)CC3)C=C2)C(=O)C=C1 | 2 | I |
| COC1=CC=C(C=N1)C1=NOC(=N1)[C@@H]1CCCN1CC1=NC=CN1 | 2 | I |
| COC1=CC=C2[C@H](N[C@@H]3N=NC(SC)=N3)OC(=O)C2=C1OC | 2 | I |
| CCOC(=O)N1CCN(CC1)C1=NC=NC2=C1C(SC)=NN2 | 2 | I |
| ClC1=NC(=NC(NC[C@@H]2CCS(=O)(=O)C2)=N1)N1C=CC=N1 | 2 | I |
| ClC1=NC(=NC(NC[C@H]2CCS(=O)(=O)C2)=N1)N1C=CN=C1 | 2 | I |
| CC(C)CN1C(=O)NC2=C(SN=C2C1=O)C(=O)N1CCOCC1 | 2 | I |
| COC1=CC(=CC(OC)=C1OC)C1=NO[C@@H](N1)[C@H]1C=C(C)N=N1 | 2 | I |
| COC1=CC(=CC(OC)=C1OC)C1=NO[C@@H](N1)[C@@H]1C=C(C)N=N1 | 2 | I |
| COC[C@]1(C)NC(=O)N(CC2=NC(=NO2)C2=CC=CC=C2C)C1=O | 2 | I |
| C[C@@H](NC(=O)C1=CC(F)=C(F)C=C1[N+]([O-])=O)C(=O)N1CCOCC1 | 2 | I |
| C[C@@H](NS(=O)(=O)C1=CC=C(C=C1)[N+]([O-])=O)C1=CN(C)N=C1C | 2 | I |
| CC(C)C1=NC(CN2C(=O)N[C@@](C)(C2=O)C2=C(C)OC(C)=C2)=NO1 | 2 | I |
| CC1=C(OC=C1)C1=NN=C(CN[C@@H]2CCCN(C2)C2=CC=CN=N2)O1 | 2 | I |
| COC1=CC=C(C(=C1)[N+]([O-])=O)S(=O)(=O)N(C)[C@@H]1CCNC1 | 2 | I |
| O=C(CN1C=NN=N1)\N=C1/NC2=CC=CC=C2N1C1=CC=CC=C1 | 2 | I |
| O=C(N1CCOCC1)C1=NNC(=C1)[C@@H]1CCCCN1C1=NC=CC=N1 | 2 | I |
| CCC[C@H](C)N1CC2(CCN(CC2)C(=O)C2=NC=NN2)OC1=O | 2 | I |
| CC1=C(C=C(C=C1Cl)S(=O)(=O)N1CCNC(=O)CC1)[N+]([O-])=O | 2 | I |
| [O-]C(=O)CN1N=C2C=C(N=C(N2C1=O)C1=CC=[NH+]C=C1)C1=CC=CC=C1 | 2 | I |
| CCCC1=NNC(=N1)[C@H]1CN(CCO1)C(=O)C1=NOC(=C1)C1CC1 | 2 | I |
| CCC1=NOC(C)=C1C(=O)N1CCO[C@@H](C1)C1=NC(=NN1)C(C)(C)C | 2 | I |
| CCCC1=NC(=NN1)[C@H]1CN(CCO1)C(=O)C1=CC(=NO1)C(C)C | 2 | I |
| CNC(=O)[C@@H]1CCCN1S(=O)(=O)C1=CC=C(C=C1C)[N+]([O-])=O | 2 | I |
| C[C@]1(NC(=O)N(CCN2C=NC=N2)C1=O)C1=CC2=CC=CC=C2O1 | 2 | I |
| CCCCC1=NN=C(NC(=O)N2CCN(CC2)C(=O)N(C)C)S1 | 2 | I |
| O=C(CNC(=O)N1CCN(CC1)C(=O)C1=CC=CO1)N1CCCCC1 | 2 | I |
| CCN1C(=O)NC2=CC(=CC=C2C1=O)C1=NC(=NO1)C(C)(C)OC | 2 | I |
| CCC1=NC(=NN1)S(=O)(=O)CC1=NOC(=N1)C1=CSC=C1 | 2 | I |
| OC1=CC=CC(=C1)[C@H]1CN(CCN2C=C(C=CC2=O)[N+]([O-])=O)CCO1 | 2 | I |
| CC(C)[NH+](CC1=CC=C(C)O1)CC1=NC2=C([O-])N(C)C(=O)N(C)C2=N1 | 2 | I |
| CC1=NOC(C[C@@H]2COC[C@@H]2NC2=C3C(C)=NOC3=NC(C)=N2)=C1 | 2 | I |
| CC(C)N1C=NN=C1CNC1=C(C=C(Br)C=N1)[N+]([O-])=O | 2 | I |
| CC(C)C1=C(CNC(=O)[C@H]2CCCCN3N=NN=C23)SN=N1 | 2 | I |
| CCC1=NN=C2CC[C@@H](CN12)NC1=CC(CC)=NC2=NC=NN12 | 2 | I |
| CNC1=NC=C(C=N1)C1=NC(=NN1C1CCN(C)CC1)[C@@H]1CCOC1 | 2 | I |
| O=S(=O)(N1CC[C@H](C1)C1=NC(=NO1)C1=CC=CC=C1)C1=CN=CN1 | 2 | I |
| CC[C@H](C)N1CC(=O)N2CCC3=C(NC4=CC=C(F)C=C34)[C@@]2(C)C1=O | 2 | I |
| CSC1=C(NC(=O)N2CCOCC2)C(SC)=NS1 | 2 | I |
| CCC1(CC)[C@@H](O)N=C(Br)C(C#N)C1(C#N)C#N | 2 | I |
| CN1C=CN=C1SCC1=NC2=C(C(C)=C(C)S2)C(=O)N1 | 2 | I |
| C[C@@H]1CCCCN1C(=O)C1=CC=C(\C=C2/SC(=O)NC2=O)C=C1 | 2 | I |
| C[C@H]1CCCCN1S(=O)(=O)C1=CC=C2SCC(=O)NC2=C1 | 2 | I |
| CC1=CC=C2NC=C(C(=O)C2=C1)S(=O)(=O)C1=CC=C(C=C1)C#N | 2 | I |
| C[C@]1(NC(=O)N(CC#N)C1=O)C1=CC=C(Br)C=C1 | 2 | I |
| C[C@@]1(NC(=O)N(CC#N)C1=O)C1=CC=C(Br)C=C1 | 2 | I |
| CN1C(=S)NC(=O)C2=C1N=C(C=C2C(F)F)C1=CC=CO1 | 2 | I |
| FC1=C2C=CC=NC2=C(CN2C(=O)N[C@@]3(CCSC3)C2=O)C=C1 | 2 | I |
| CN([C@H]1CCC2=C1C=CC=C2)S(=O)(=O)C1=CNC(=O)C=C1 | 2 | I |
| CN([C@@H]1CCS(=O)(=O)C1)C(=O)[C@@H]1CCC2=CC=CC=C2N1 | 2 | I |
| CC(C)C1=NN(C(Cl)=C1CO)[C@@]1(C)CCS(=O)(=O)C1 | 2 | I |
| C[C@@H]1N(CCC2=C1C=CS2)S(=O)(=O)C1=C(C)NN=C1C | 2 | I |
| BrC1=CN(CC2=NC3=C(SC=C3)C(=O)N2)N=C1 | 2 | I |
| ClC1=NC=C(S1)S(=O)(=O)NC1=C(C=CS1)C#N | 2 | I |
| CCN1CCN(CC1)C(=O)[C@]12CC[C@](C)(\C(C1)=N\O)C2(C)C | 2 | I |
| OCC#CC1=CC(=CN=C1)S(=O)(=O)N1CCCCCCC1 | 2 | I |
| C[C@@H](NC1=NN2C(S1)=NC=CC2=O)C1=C(C)SC(C)=C1 | 2 | I |
| C[C@@H]1SC(=N)C(=C1C)C1=NC(CC2=CSC(C)=N2)=NO1 | 2 | I |
| CC1=C(C)C2=C(NC(=S)N(CC3=NC=C(C)N=C3)C2=O)S1 | 2 | I |
| CSC1=NC(=O)[C@H]2[C@H](CC(=O)NC2=N1)C1=CC(C)=CC=C1C | 2 | I |
| O=C1CCCC2=C(N1)C=CC(=C2)S(=O)(=O)N1CCCSCC1 | 2 | I |
| CN1CCN(CC1)[C@@H]1CCN(C[C@H]1O)C1=CC=C2C=CC=CC2=N1 | 2 | I |
| ClC1=CC=CC=C1NCN1C(=O)[C@H]2[C@H]3O[C@H](C=C3)[C@H]2C1=O | 2 | I |
| C[C@H]1CN(CC2=NC3=C(C4=C(CCC4)S3)C(=O)N2)[C@H](C)CO1 | 2 | I |
| C[C@H](SC1=NNC=N1)C(=O)N1CCSC2=C1C=CC=C2 | 2 | I |
| CNC1=C2C=CSC2=NC(CN2CC[C@H](C)CCC2=O)=N1 | 2 | I |
| C[C@@H]1CCC2=C(C1)C=C(S2)C(=O)N(C)CC1=NC(C)=NN1 | 2 | I |
| OC1=CC2=C(CCN(C2)C(=O)[C@H]2CN(C3CCCC3)C(=O)C2)C=C1 | 2 | I |
| CC1(C)CNCCN1CC1=CC(=O)N2C=C(Cl)C=CC2=N1 | 2 | I |
| C[C@H]1C[C@@H](C)CN(C1)S(=O)(=O)C1=NNC(C)=C1CCl | 2 | I |
| C[C@H]1CCN([C@@H](C)C1)S(=O)(=O)C1=C(CCl)NN=C1C | 2 | I |
| C[C@@H]1CCN([C@@H](C)C1)S(=O)(=O)C1=C(CCl)NN=C1C | 2 | I |
| C[C@@H]1CCCCCN1S(=O)(=O)C1=C(CCl)C(C)=NN1 | 2 | I |
| OC(=O)C[C@H]1CCN(C1)C(=O)N1CCC[C@@H](C1)C(F)(F)F | 2 | I |
| O=C(N[C@H]1CCO[C@]2(CCSC2)C1)N1CCN(CC#C)CC1 | 2 | I |
| [O-]C1=NC(=NC(C2CC2)=C1Br)[C@H]1C[NH+]2CCC[C@@H]2CO1 | 2 | I |
| CCCC1=NN(C)C2=C1NC(=S)N2[C@@H]1CCN2CCC[C@H]12 | 2 | I |
| ClC1=C(C=C2NC(=O)C(=O)C2=C1)N1CCN(CC1)C1CC1 | 2 | I |
| CCC1=C(Br)C([O-])=NC(=N1)[C@@H]1CN(C)CC[NH+]1C | 2 | I |
| CNCC1=C(N=C2SC=CN12)N1CCN2CCCC[C@@H]2C1 | 2 | I |
| CN1C=C(C=N1)N1CCC[C@H](C1)N[C@H]1CC(C)(C)OC1(C)C | 2 | I |
| C[C@@H]1CO[C@@H](CO)CN1C1=C2C3=C(CCC3)SC2=NC=N1 | 2 | I |
| O=C(N1CCC2(C[C@@H](C(=O)N2)C2=CC=NC=C2)CC1)C1=CC=CS1 | 2 | I |
| CC(C)NC(=O)N1CC[C@@]2(C1)CN(C(=O)C2)C1=CSC=C1 | 2 | I |
| O=C(N1C[C@H]2CC=CC[C@H]2C1)C1=CC(=CC=C1)N1CCNC1=O | 2 | I |
| COC[C@@]1(C)NC(=O)N(CC#CC2=CC=C(Cl)C=C2)C1=O | 2 | I |
| C[C@H]1N(CC(=O)NC1=O)C1=CC=C(C=C1Br)C(C)=O | 2 | I |
| C\C(=C1/SC(=S)NC1=O)C1=CC=CC(=C1)[N+]([O-])=O | 2 | I |
| OCCN1CCOC2=CC3=NC4=CC=CC=C4N=C3C=C12 | 2 | I |
| CC1=CN\C(C=C1)=N/S(=O)(=O)C1=CC=C(C=C1)C#N | 2 | I |
| O=C1OC2(CCCCC2)C(N[C@H]2CCS(=O)(=O)C2)=C1 | 2 | I |
| CC1=CC=C(CN2C(=S)NC3=C(N=CC=N3)C2=O)C=C1 | 2 | I |
| O=C(C1=CNC2=C1C=CC=N2)C1=CC=C2OCCOC2=C1 | 2 | I |
| CC1=NC2=CC=CC=C2C(=O)N1\N=C\C1=CC=C(O)C=C1 | 2 | I |
| O=C1N(CC2=CC=NC=C2)C=NC2=C1NC1=CC=CC=C21 | 2 | I |
| CC1=CC=C(C=C1)[C@@H]1C[C@H](N2N=CN=C2N1)C1=CC=CO1 | 2 | I |
| ClCCC(=O)NC(=O)N1CCO[C@H]2CCCC[C@@H]12 | 2 | I |
| CCN1C(=O)NC(=CC1=O)N1CCC[C@H]2CCCC[C@H]12 | 2 | I |
| FC1=C(F)C=C2N(CCCC2=C1)S(=O)(=O)C1=CNN=C1 | 2 | I |
| CC1=CN\C(S1)=N/C(=O)N1CCN(CCC#C)CC1 | 2 | I |
| C[C@H]1CCC[C@@H](C)N1C(=O)N1CCC[C@H](CC(O)=O)C1 | 2 | I |
| C[C@@H]1CCN([C@@H](C)C1)C(=O)N1CCC[C@H](CC(O)=O)C1 | 2 | I |
| OC1=CC=C(C=C1)N1CCN(CC1)C1=CC=C(C=N1)C#N | 2 | I |
| C1[C@@H](NC2=NN=NN2[C@H]1C1=CC=CC=C1)C1=CC=CS1 | 2 | I |
| CC1=CC=C(C=C1)[C@@H]1NC(=O)C2=NN=C([C@H]12)C1=CC=CO1 | 2 | I |
| C1CN(CCC1C1=CC=NN1)C1=NC=NC2=C1SC=C2 | 2 | I |
| C[C@@H](NC(=O)N1CCOC(C)(C)C1)C1=C(C)OC(C)=C1 | 2 | I |
| CC[C@@H]1N(CCNC1=O)C1=CC(C#N)=C2C=CC=CC2=N1 | 2 | I |
| C[C@H]1COC2=CC=C(C=C2OC1)C1=CC=C([NH+]=C1)C([O-])=O | 2 | I |
| CC1=C(C)C(N[C@]2(C)CCS(=O)(=O)C2)=NN=C1Cl | 2 | I |
| C1CN(CCC1C1=CC2=C(N1)N=CC=C2)C1=NC=CC=N1 | 2 | I |
| O=C([C@@H]1COC2=CC=CC=C2O1)N1CCC[C@H]1C1=CC=CN1 | 2 | I |
| ClC1=CC(C#N)=C(C=C1)N1CCC[C@H](C1)N1CCNC1=O | 2 | I |
| C[C@@H](NC1=NC=CN2C=NN=C12)C1=CC=CC=C1Cl | 2 | I |
| O=S(=O)(N1CCCCC2=CC=CC=C12)C1=CN=CN1 | 2 | I |
| CC1(C)CCCN(C1)S(=O)(=O)C1=CNC(=O)C(Cl)=C1 | 2 | I |
| [O-][N+](=O)[C@H]1CCC(=O)N[C@@H]1C1=CC=C(Br)C=C1 | 2 | I |
| COC1=CC=CC=C1N1CCC(=O)NC2(CCCC2)C1=O | 2 | I |
| Cl[C@@H]1CC2=CC=CC=C2[C@@H]1NC1=NC=CN2C=NN=C12 | 2 | I |
| CNC1=NC(N2CCO[C@@H](C)C2)=C(Br)C=N1 | 2 | I |
| CC1(C)CCC(CC1)C1=NOC(=N1)C1=C([O-])C=CC=[NH+]1 | 2 | I |
| [O-]C(=O)C1=C([NH+]=C2C=CC=CN12)N1CC2=CC=CC=C2C1 | 2 | I |
| CC1=CC(CC2=NOC(=N2)[C@@H]2COCCN2)=CC(C)=C1 | 2 | I |
| O=C1NC(\C=C\C2=CC3=C(C=CS3)N=C2)=NC2=C1C=CN=C2 | 2 | I |
| O[C@H]1CCCC[C@H]1N1CCC2=C(C1)C=C(C=C2)[N+]([O-])=O | 2 | I |
| C[C@@H]1CN(C[C@@]1(O)C1CCC1)C1=NN=C2C=CC=CN12 | 2 | I |
| CCC1=CC=C(O1)[C@H](O)C1=CC=C2N(C)C(=O)OC2=C1 | 2 | I |
| S=C1NC2=C(N=CC=C2)N1CC1=CC=C2OCOC2=C1 | 2 | I |
| C[C@H]1CCN([C@@H](C)C1)C(=O)N1CCC(CC(O)=O)CC1 | 2 | I |
| C[C@@H]1O[C@H](C)[C@@H]([C@@H]1C)C1=NC2=CC=C(NC2=N1)N(C)C | 2 | I |
| C[C@H]1O[C@H](C)[C@@H]([C@H]1C)C1=NC2=CC=C(NC2=N1)N(C)C | 2 | I |
| N1C2=CN=CC=C2N=C1C1=CN=C(S1)C1=CN=CC=C1 | 2 | I |
| C1COC[C@H](N1)C1=NOC(=N1)C1=CC2=CC=CC=C2C=C1 | 2 | I |
| CC[C@H]1N(CCC[C@]11CCC(=O)N1)C1=NC=C(F)C=N1 | 2 | I |
| OC1=CC=CC(\C=C(\C#N)C2=NN=C3CCCCCN23)=C1 | 2 | I |
| CN1C(=O)NN=C1SC1=C(Br)C=C(C)C=N1 | 2 | I |
| O=C1N(NC2=NC=CC(C3=CC=CO3)=C12)C1=CC=CC=C1 | 2 | I |
| O=S(=O)(NC1=NC=NC2=CC=CC=C12)C1=CC=CS1 | 2 | I |
| C[C@@H](N1C(=O)CC(=O)NC1=O)C12CC3CC(CC(C3)C1)C2 | 2 | I |
| FC1=CC=CC=C1CN1C(=O)NC2=CC=CC=C2S1(=O)=O | 2 | I |
| COC1=CC=CC=C1N1C(=S)NN=C1N1CCCCC1 | 2 | I |
| CC1=C(SC(Cl)=N1)S(=O)(=O)\N=C1/NC=C(C)C=C1 | 2 | I |
| C[C@@H]1CN(C[C@@H](C)O1)C1=NNC(=S)N1C1CCCCC1 | 2 | I |
| O=C(NC[C@@H]1COC2=CC=CC=C2O1)N1CCSCC1 | 2 | I |
| OCC1=C(N=C2SC=CN12)N1CCO[C@H]2CCCC[C@H]12 | 2 | I |
| C[C@@H]1CCCN(C1)C(=O)[C@H]1CCCN(C1)C(=O)NC1CC1 | 2 | I |
| CC1CCN(CC1)C(=O)[C@@H]1CCCN(C1)C(=O)NC1CC1 | 2 | I |
| [S-]C1=NN=C(N1[C@@H]1CC[NH+]2CCCC[C@H]12)C1=CC=NC=C1 | 2 | I |
| C[C@H]1CN([C@H](C)CO1)C1=C(C=C2CCCCC2=[NH+]1)C([O-])=O | 2 | I |
| O=C1N(CC#C)[C@H](NC2=CC=CC=C12)C1=CC=CC2=C1OCO2 | 2 | I |
| [O-]C(=O)C1=C([NH+]=C(S1)N1CCOCC1)C1=CC=CC=C1 | 2 | I |
| N=C1S[C@H]2CCCCC2=C1C1=NC(=NO1)C1=CC=CC=N1 | 2 | I |
| O=C1NC(=NC2=C1CCCC2)N1CCN=C1C1=CC=CC=C1 | 2 | I |
| N=C1S[C@H](C=C1)C1=NC(=NO1)C1=CC=C2N=CC=CC2=C1 | 2 | I |
| O[C@@H]1CN(CC[C@H]1C1=CC2=C(OCO2)C=C1)C(=O)C1CCC1 | 2 | I |
| CC1CCC(CC1)SC1=NN=NN1C1=CC=C(O)C=C1 | 2 | I |
| CC1=CC=CC=C1C1=NN2C=CN=C(N3CC[C@@H](O)C3)C2=C1 | 2 | I |
| CN1C=CS\C1=N/C(=O)CC1=CC2=CC=CC=C2NC1=O | 2 | I |
| CS(=O)(=O)C1=NNC(=N1)C1=CC=C(Br)C=C1 | 2 | I |
| CC1=C(NN=C1)[C@@]1(C)CCCN(C1)C(=O)C1=CSC=N1 | 2 | I |
| CN1C[C@]2(CCCN(CC3=C(C)C=C(O)C=C3C)C2)OC1=O | 2 | I |
| CC1=CC=CC=C1N1NC2=CC(=O)N(C3CC3)C(C)=C2C1=O | 2 | I |
| CC1=C(N2C(=O)C3=C(CCCC3)C2=O)C(=NN1)C(F)(F)F | 2 | I |
| CC(C)(C)C1=NC(=NN1)[C@H]1CN(CCO1)[C@@H]1CCCC=C1 | 2 | I |
| C[C@H]1CC[NH+](CC2=NS(=O)(=O)C3=CC=CC=C3[N-]2)[C@H](C)C1 | 2 | I |
| C[C@H]1CCC[C@@H](C1)NC(=O)N1CCC(CC1)C(=O)N(C)C | 2 | I |
| O=C(NC[C@@H]1CN2CCC[C@@H]2CO1)N1C[C@H]2CCC[C@@H]2C1 | 2 | I |
| CO[C@H]1CCC[C@H](C1)N1CC(=O)NC2(CCCCC2)C1=O | 2 | I |
| CC[C@@H]1N([C@@H](C)C2=CC=CO2)C(=O)C2(CCCC2)NC1=O | 2 | I |
| CC[C@@H]1CN([C@H](CC)CN1)C(=O)C1=CC=C2OCOC2=C1 | 2 | I |
| CC1=C(C)C(=C(C)O1)C1=NC2=CC=C(C=C2N1)S(C)(=O)=O | 2 | I |
| OC(=O)C[C@H]1CCCN(C1)C(=O)N1CCC[C@H]2CCC[C@H]12 | 2 | I |
| N=C1S[C@H](C=C1)C1=NC(=NO1)[C@H]1CCOC2=C1C=CC=C2 | 2 | I |
| C[C@@H]1CCC[C@]2(C1)N(CC1=CC=NC=C1Cl)C(=O)NC2=O | 2 | I |
| C[C@@H]1CCC[C@@]2(C1)N(CC1=CC=NC=C1Cl)C(=O)NC2=O | 2 | I |
| O=C(N1CCCCCCC1)C1=CC=C(C=C1)N1CCNC1=O | 2 | I |
| O=C(N[C@@H]1CCC[C@H]2OCC[C@@H]12)N1CCOC[C@@H]1C1CC1 | 2 | I |
| C[C@H]1CCN([C@@H]1C)C(=O)NC[C@@H]1CN2CCCC[C@@H]2CO1 | 2 | I |
| CCC1=C(Br)C(=O)N=C(N1)C1=C(C)ON=C1C | 2 | I |
| CCOC1=C(C)C(=NC=N1)N1CCNCC11CCCCC1 | 2 | I |
| CCC[C@H]1CC[C@@H](C#N)[C@@H](C1)N1CCN2[C@H](CNC2=O)C1 | 2 | I |
| [O-]C1=CC(C[NH+]2CCC3=C(C2)C=CC(F)=C3)=NC2=CC=NN12 | 2 | I |
| CN1CCO[C@H](COC2=CC=CC3=C2CCC\C3=N/O)C1 | 2 | I |
| CCCC1=NC(=NN1)C(=O)N1CC[C@@H](C)[C@@H]2CCCC[C@H]12 | 2 | I |
| CN1C(=O)\C(=C\NC2=CC=C(C)C(C)=C2)C(C)=C(C#N)C1=O | 2 | I |
| O=C(NC[C@@H]1CCS(=O)(=O)C1)N1CCC[C@H]2CCC[C@@H]12 | 2 | I |
| [O-][N+](=O)C1=CC=C(S1)C1=CC(=NCCN1)C(F)(F)F | 2 | I |
| C[C@]12N(CCC3=C1NC1=CC=CC=C31)C(=O)N(CCCl)C2=O | 2 | I |
| O=C1[C@H]2[C@@H]3C=C[C@H]([C@@H]2C(=O)N1\N=C\C1=CNC2=CC=CC=C12)C31CC1 | 2 | I |
| ClC1=CC=CC=C1N1C(=O)NC(=O)\C(=C/C2=CC=CC=C2)C1=O | 2 | I |
| CC1=CC=C(C=C1)S(=O)(=O)N1CCC2=CC=CC3=C2[C@@H]1CC(=O)N3 | 2 | I |
| CNC(=O)CN(C)C1=NC=NC2=C1C1=C(C[C@H](C)CC1)S2 | 2 | I |
| C[C@@H]1N(CCC2=C1C=CS2)C(=O)CC1=NNC(=O)C2=CC=CC=C12 | 2 | I |
| C\C(=C/C1=CC=CC=C1)[C@@H]1N2N=CN=C2NC2=C1C(=O)CCC2 | 2 | I |
| C[C@@H]1CCC[C@@H]([N-]S(=O)(=O)C2=C(Cl)N(C)C=[NH+]2)[C@@H]1C | 2 | I |
| [O-]C1=CC=C(F)C=C1C1=[NH+]C2=NC=NN2[C@H](C1)C1=CC=CC=C1 | 2 | I |
| CC1=CC=C2NC(=O)C(=CC2=C1)C1=NOC(=N1)C1=CC=CS1 | 2 | I |
| N#CC1=CC(=CC=C1)[C@@H]1N[C@@H]2N(C[C@@H]3CCC=CC3)N=CC2=N1 | 2 | I |
| [O-]\C(=N/N=C\C1=C2C=CC=CC2=[NH+]C1)[C@H]1CC2=CC=CC=C2O1 | 2 | I |
| C[C@@H]1C[C@]2(CC(C)(C)C1)NC(=O)N(CN1CCCCC1)C2=O | 2 | I |
| CN1N=CC(NC[C@H]2COC3=CC=CC=C3C2)=C(Cl)C1=O | 2 | I |
| CN1C(=O)NN=C1SCC1=CC=C(Cl)C2=C1N=CC=C2 | 2 | I |
| C[C@H]1C[C@@H](C)CN(C1)C(=O)C1CCN(CC1)C(=O)NC1CC1 | 2 | I |
| O[C@H]1CCCN(C1)C1=C(Cl)C(=O)N(N=C1)C1=CC=C(Cl)C=C1 | 2 | I |
| O=C1NC(=NC2=C1C(=CS2)C1=CC=CS1)C1=NC=CC=N1 | 2 | I |
| O=C1N[C@]2(CCCC3=CC=CC=C23)C(=O)N1C[C@H]1CCCCO1 | 2 | I |
| FC1=CC=C(C=C1)[C@H]1C[C@H](N2N=NN=C2N1)C1=CC=C(F)C=C1 | 2 | I |
| CC1=CC(=CC(C)=C1)N1C(=O)NC(=O)\C(=C/C2=CC=CC=C2)C1=O | 2 | I |
| C[C@@H]1CCC2=C(C1)[C@H](N=N2)[C@@H]1NC(=NO1)C1=CC=C(Cl)C=C1 | 2 | I |
| COC1=CC=CC(Cl)=C1C1=NC=CC(=N1)N1CC[C@H](O)C1 | 2 | I |
| C1O[C@@H](O[C@@H]2[C@H]1N[C@H]1CO[C@@H](O[C@@H]21)C1=CC=CC=C1)C1=CC=CC=C1 | 2 | I |
| CCC1=NC=C(N1)S(=O)(=O)N1CCCC2=C1C=CC(C)=C2 | 2 | I |
| ClC1=CC=NC=C1S(=O)(=O)\N=C1/C=CC=C2NC=CC=C12 | 2 | I |
| CC1=NN2C(S1)=NC(NC2=O)(C(F)(F)F)C(F)(F)F | 2 | I |
| CC1=CC=C2OC(=CC2=C1)C(=O)N1CCN(CC1)[C@H]1CCC[C@@H]1O | 2 | I |
| CC1(C)O\C(=C/C=C2\SC(=O)NC2=O)C=C(O1)C1=CC=CC=C1 | 2 | I |
| CN1C2=C([C@@H](N=C1O)C1=CC=CC=C1)C(=O)N(C2)C1=CC=CC=C1 | 2 | I |
| CN(C[C@H]1COC2=CC=CC=C2O1)C(=O)NC1=CC=CS1 | 2 | I |
| CC1(C)[C@@H](NC(=O)[C@]2(C)CC3=CC=CC=C3C(=O)O2)[C@@H]2CCO[C@H]12 | 2 | I |
| O=C(N1CCC[C@@H]1C1CCCCC1)C1=CC2=C(OCC(=O)N2)C=C1 | 2 | I |
| ClC1=CC(=CNC1=O)S(=O)(=O)N1CCC[C@H]2CCCC[C@H]12 | 2 | I |
| C[C@H]1C[C@@H](C)CC(C1)SC1=NN=NN1C1=CC=C(O)C=C1 | 2 | I |
| O=C1NC2=C(CN(CC2)S(=O)(=O)C2=CC3=CC=CC=C3C=C2)C=C1 | 2 | I |
| CC(C)C(=O)N1CC[C@H](C1)NC(=O)N1CCC[C@@H]1C1CCC1 | 2 | I |
| CC(C)C(=O)N1CC[C@H](C1)NC(=O)N1CCC[C@H]1C1CCC1 | 2 | I |
| C[C@H]1CN(C[C@H](O1)C1=CSC=C1)C(=O)NCC1=CC=CO1 | 2 | I |
| CC1(C)CN(CCS1)C(=O)NC[C@@H]1CN2CCCC[C@@H]2CO1 | 2 | I |
| CNC1=NC(=NC(C(C)C)=C1Br)C1=NC=CC=N1 | 2 | I |
| C[C@H]1C[C@H]2CNC[C@@H]2N1S(=O)(=O)C1=CC=C(CC#N)C=C1 | 2 | I |
| C[C@@H](NC1=NN2C=CC=C(Br)C2=N1)C1=CC=CO1 | 2 | I |
| O[C@@H]1CCCOC11CCN(CC1)C1=NN=C(S1)C1=CC=CC=C1 | 2 | I |
| O=C(NC1CC1)N1CCC2=NC=NC(C3=CSC=C3)=C2CC1 | 2 | I |
| CC(C)NC1=NC(C)=C2CCN(CCC2=N1)C1CCOCC1 | 2 | I |
| C[C@@H](NC1=CC=C2N(C)C(=O)N(C)C2=C1)C1=CC=C(C=C1)C#N | 2 | I |
| CC1=CC=C(C=C1)N1C(=O)NC(=O)\C(=C\C2=CC=CC=C2C)C1=O | 2 | I |
| C(NC1=NC2=CC=CC=C2S1)[C@@H]1COC2(CCOCC2)O1 | 2 | I |
| CN1C(=O)\C(=C/NC2=CC=C(C)C(Cl)=C2)C(C)=C(C#N)C1=O | 2 | I |
| CSC1=CC=C(C=C1)[C@H]1C[C@@H](NC2=NN=NN12)C1=CC=C(Cl)C=C1 | 2 | I |
| CC1=CC(C)=C(C=C1C)C(=O)CN1C(=O)N[C@@]2(CCC3=C2C=CC=C3)C1=O | 2 | I |
| COC1=CC=C(Br)C=C1\C=N/N=C1\C(=O)NC2=CC=CC=C12 | 2 | I |
| COC1=CC=C(\C=C2\C(C)=NN(C2=O)C2=CC=C3C=CC=CC3=C2)C=C1O | 2 | I |
| BrC1=CC=C(CC(=O)NN2C(=O)C3=CC=CC=C3C2=O)C=C1 | 2 | I |
| C[C@H]1CCC[C@H](C)N1C(=O)CSC1=NC2=C(C(C)=C(C)S2)C(=O)N1 | 2 | I |
| CNC1=NC2=C(C3=C(COC(C)(C)C3)S2)C(=O)N1CC1=CC=CC=C1 | 2 | I |
| CC1=CC(=O)NC(SCC2=C(Br)N3C=CSC3=N2)=N1 | 2 | I |
| C[C@H]1CCC[C@@H](NC(=O)CN2C=NC3=C(C4=C(CCCC4)S3)C2=O)[C@@H]1C | 2 | I |
| CC1=CC(NS(=O)(=O)C2=CC3=C4N(CCC3)C(=O)CCC4=C2)=CC(C)=C1 | 2 | I |
| C[C@@H]1CC2=CC(=CC=C2N1C(C)=O)S(=O)(=O)NC1=CC(C)=CC=C1C | 2 | I |
| CN(C1=CC=C2C=CC=CC2=C1)S(=O)(=O)C1=CC=C2NC(=O)CCC2=C1 | 2 | I |
| BrC1=CC2=C(C=C1)C(=O)N(CC(=O)NC1CCCCC1)C2=O | 2 | I |
| O=C1C[C@@H](CC2=C1[C@@H](N1N=CN=C1N2)C1=CC=CS1)\C=C\C1=CC=CC=C1 | 2 | I |
| FC1=CC=CC=C1C(=O)CN1C(=O)N[C@]2(CCCCC3=CC=CC=C23)C1=O | 2 | I |
| C[C@H](SC1=C(C#N)C(C)=CC(C)=N1)C1=NC2=C(C(C)=C(C)S2)C(=O)N1 | 2 | I |
| O=C([C@H](C#N)C1=NC2=CC=CC=C2N1)C1C2=CC=CC=C2OC2=CC=CC=C12 | 2 | I |
| FC1=CC=C(N[C@@H]2CCCN(C2)C(=O)C2=CC3=CC=CC=C3OC2=O)C=C1 | 2 | I |
| C[C@@H]1C[C@@H](N2N=C(C=C2N1)[C@H]1CCCN1C(=O)C1CCCC1)C(F)(F)F | 2 | I |
| CC(C)SC1=NN2[C@@H](C3=C(C)C=CS3)C3=C(CCCC3=O)NC2=N1 | 2 | I |
| CC(C)SC1=NN2[C@@H](C3=CC=C(C)C=C3)C3=C(CCCC3=O)NC2=N1 | 2 | I |
| CC(C)(C(=O)N1CCN(CC1)C(=O)C1=CC2=CC=CC=C2N1)C1=CC=CC=C1 | 2 | I |
| C[C@H]1CCC2=C(C1)SC1=C2C(=O)N(CC(=O)NC2CCCCC2)C=N1 | 2 | I |
| C[C@@H]1CN(CC[C@H]1O)C1=NC=C(C2=CSC=C2)C(=N1)C1=NC(C)=CS1 | 2 | I |
| CC1=CC=C2C(NC3=C2N=CN=C3N2CCN(CC3=CC=CC=C3)CC2)=C1 | 2 | I |
| CC1=CC=C(C(C)=C1)S(=O)(=O)NC1=CC2=C3N(CCC2)C(=O)CC3=C1 | 2 | I |
| O=C(CC1=CC=CS1)NC1=CC=C2OCC[C@@H]3CCCCN3C(=O)C2=C1 | 2 | I |
| C[C@H]1C[C@H](C)CN(C1)C(=O)C1CCN(CC2=NC3=CC=C(C)C=C3N2)CC1 | 2 | I |
| C[C@H]1OC(=N)C(=C1C)S(=O)(=O)C(=C/C1=CC(Cl)=CC(Cl)=C1)\C#N | 2 | I |
| O=C(N[C@@H]1C=C(N=N1)C1=CC=CC=C1)C1=C[C@@H]2COC3=CC=CC=C3[C@@H]2S1 | 2 | I |
| ClC1=CC(CN2CCC(CC2)N2C(=O)NC3=CC=CC=C23)=CC(Cl)=N1 | 2 | I |
| C[C@@H]1CCCCN1C1CN(C1)C1=C2CCNCC2=NC(=N1)C1=CC=CC=C1 | 2 | I |
| C[C@H](NC(=O)N1[C@H](C)CC2=CC=CC=C12)C1=CC=CC(=C1)N1CCCC1=O | 2 | I |
| C[C@H]1CC[C@H](NC(=O)C2=CC(=CC=C2Br)[N+]([O-])=O)[C@H](C)C1 | 2 | I |
| C[C@@H](N1C(=O)C=C2NN(C(=O)C2=C1C)C1=CC=CC=C1C)C1=CC=CC=C1 | 2 | I |
| CC(C)N1CCN(CC1)[C@@H](C)C1=NC2=C(C3=C(C[C@H](C)CC3)S2)C(=O)N1 | 2 | I |
| CC[C@H]1CCC2=C(C1)SC1=C2C(=O)NC(CC2=NC(C)=CC(C)=N2)=N1 | 2 | I |
| COC1=CC=C(C=C1)[C@@H]1C[C@H](C)N(C1)C(=O)C1=CC2=C(CCCC2)NC1=O | 2 | I |
| OC1=CC2=C(C=C1Cl)C(CN1C=C(C=CC1=O)C(F)(F)F)=CC(=O)O2 | 2 | I |
| BrC1=CC(=CC=C1)C(=O)\N=C1/NN=CN1CC1=CC=CC=C1 | 2 | I |
| O=C(NC1CCC2(CC1)OCCO2)N1CCC(CC2=CC=CC=C2)CC1 | 2 | I |
| FC1=C(C=CC(NC(=O)N2CCN(CC2)C2CCCC2)=C1)N1CCCC1 | 2 | I |
| C[C@H](NC(=O)N1CCC(=CC1)C1=CC=CC=C1Cl)[C@@H]1CN(C)CCO1 | 2 | I |
| CN1C(=O)N\C(=C/C2=CN(CC3=CC=CC=C3Cl)C3=C2C=CC=C3)C1=O | 2 | I |
| ClC1=C(Cl)C=C(C=C1)N1CC[C@@H](C(=O)NC2(CCCC2)C#N)C1=O | 2 | I |
| [O-][N+](=O)C1=CC(I)=C(F)C=C1NC1CCOCC1 | 2 | I |
| [O-][N+](=O)C1=CC(I)=C(N[C@H]2CCN3CCC[C@H]23)C=C1 | 2 | I |
| ClC1=CC=C(C2=C1N=CC=C2)S(=O)(=O)N[C@@H]1CCOC2=CC=CC=C12 | 2 | I |
| CCNC1=C(Br)C=NC(=N1)C1=CC2=C(OCCO2)C(Cl)=C1 | 2 | I |
| CC1=CC=C2C(NC=C2C(=O)C(=C/C2=CC=CC3=C2OCCCO3)\C#N)=C1 | 2 | I |
| O=C1NC(CC2=CC=CC=C2)=NC2=C1C=NN2C1=CC=CC=C1 | 2 | I |
| CN1C(=O)N\C(=C/C2=CC=C(O2)C2=CC=CC(Cl)=C2Cl)C1=O | 2 | I |
| COC1=CC(CN2C(=O)N[C@@]3(CCCC[C@H]3C)C2=O)=CC=C1 | 2 | I |
| [O-][N+](=O)C1=C2[C@@H]3C=CC[C@H]3[C@H](NC2=C(F)C=C1)C1=CN=CC=C1 | 2 | I |
| C[C@H]1CCC2=C(C1)SC1=C2C(SC2=NC=NN2)=NC=N1 | 2 | I |
| COC(=O)C1=C(C#N)C(=O)NC(=C1)C1=CC=CC2=C1C=CC=C2 | 2 | I |
| CC1=CSC2=NC(C)=C(CNC3=CC=C4OCOC4=C3)N12 | 2 | I |
| CCCNC(=O)N1CCN(C(=O)[C@@H]1C)C1=CC=C(C)C(C)=C1 | 2 | I |
| CCCNC(=O)N1CCN(C(=O)[C@H]1C)C1=CC(C)=CC=C1C | 2 | I |
| CC1=CC=CC=C1N1C(=O)NC(=CC1=O)N1CCC2=C1C=CC=C2 | 2 | I |
| ClC1=CC=CC2=C1C1=C(N2)C(=O)N(C[C@@H]2CCCO2)C=N1 | 2 | I |
| CC1=C(C)C(=O)NC(=N1)N1CCN(CC1)C1=CC=CC=C1F | 2 | I |
| C[C@H]1CCC[C@H](NC(=O)[C@@H]2CCCN(C2)C(=O)N2CCCC2)[C@@H]1C | 2 | I |
| O[C@@H](C1=CC=C(O1)[N+]([O-])=O)C1=CC=C(Br)S1 | 2 | I |
| O=S(=O)(NC1(CCCCCC1)C#N)C1=CC=C(C=C1)C#N | 2 | I |
| BrC1=CC=CC(=C1)C1=NOC(=N1)C1=CNC(=O)C=C1 | 2 | I |
| COC1=CC2=C(NC(=C2)C(=O)N2CCCCCC2)C(OC)=C1 | 2 | I |
| CC1=CC=CC=C1NC1=NC(=O)\C(S1)=C/C1=CSN=N1 | 2 | I |
| C[C@@H](NC(=O)N1CCC2(CC1)OCCO2)C1=C(C)SC(C)=C1 | 2 | I |
| ClC1=CC=CC=C1[C@H]1C[C@H](N2N=CN=C2N1)C1=CC=CO1 | 2 | I |
| C[C@H]1CN(C[C@H](C)N1)C1=CC=C(C(=C1)C(F)(F)F)[N+]([O-])=O | 2 | I |
| C[C@@H]1CN(C[C@@H](C)N1)C1=CC=C(C(=C1)C(F)(F)F)[N+]([O-])=O | 2 | I |
| O=C(\C=C\C1=NC2=CC=CC=C2C=C1)N1CC(=O)NC2=C1C=CC=C2 | 2 | I |
| OC1=CC=C(C=C1)C1=NOC(=N1)C1=CC=C(Br)C=N1 | 2 | I |
| CC1=CC=C(C)C(=C1)N1C(=O)NC(=O)\C(=C/C2=CC=CS2)C1=O | 2 | I |
| O=C1NC(CC2=CC=C(C=C2)N2C=CC=N2)=NC2=CC=CC=C12 | 2 | I |
| CCC#CCN1CCOC2=C(C1)C=C(C=C2O)C1=C(C)N=CC(C)=N1 | 2 | I |
| O=C1N(CC2=CC=CC=C2)C(=O)C(=O)C2=C1NC1=CC=CC=C21 | 2 | I |
| O=C(N1CCO[C@H](C1)C1=CC=CC=C1)C1=CC2=CC=CC=C2C(=O)N1 | 2 | I |
| C[C@@H]1CN(CCO1)C(=O)NC[C@H]1CCN(C1)C1=CC=CC=C1 | 2 | I |
| CCC1=NC2=CC=NN2C(=C1)N1CC[C@]2(O)CCCC[C@@H]2C1 | 2 | I |
| O=C(N[C@@H]1CCC[C@H]1N1C=CN=C1)N1C[C@H]2CCCC[C@H]2C1 | 2 | I |
| C[C@@H]1CN([C@H](C)CN1)C1=CC=C(C=C1[N+]([O-])=O)C(F)(F)F | 2 | I |
| C[C@@H]1CN([C@@H](C)CN1)C1=CC=C(C=C1[N+]([O-])=O)C(F)(F)F | 2 | I |
| C[C@@H]1CN([C@@H](C)CN1)C1=CC=C(C(=C1)C(F)(F)F)[N+]([O-])=O | 2 | I |
| C[C@H]1CN([C@@H](C)CN1)C1=CC=C(C=C1C(F)(F)F)[N+]([O-])=O | 2 | I |
| CC(C)C1=C(Br)C(Cl)=NC(=N1)C1=CNN=N1 | 2 | I |
| O[C@@H](C1=CC=C(O1)[N+]([O-])=O)C1=C(Br)C=CS1 | 2 | I |
| C[C@H]1CN([C@@H](C)CN1)C1=CC(Br)=CC=C1[N+]([O-])=O | 2 | I |
| ClC1=C(NCC2=C(N=CC=C2)C#N)C2=NSN=C2C=C1 | 2 | I |
| CC1(C)CNCCN1C1=CC=C(C(=C1)C(F)(F)F)[N+]([O-])=O | 2 | I |
| CC1(C)CNCCN1C1=CC=C(C=C1C(F)(F)F)[N+]([O-])=O | 2 | I |
| CC1=CC=C(C=C1)N1NC(=CC1=O)C(=O)N1CC[C@@H]2CCCC[C@H]12 | 2 | I |
| O=C(NC[C@H]1CC2=CC=CC=C2O1)N1CCOC[C@H]1C1CC1 | 2 | I |
| O=C(NC[C@H]1CN2CCCC[C@H]2CO1)N1CCC[C@H]2CCC[C@H]12 | 2 | I |
| CC1(C)CCCC[C@H]1NS(=O)(=O)C1=CN=C(Cl)N=C1 | 2 | I |
| CC1=CC=C(C=C1N[C@H]1CSCC(C)(C)C1)N1C=NN=N1 | 2 | I |
| CN[C@@H]1CCCN(C1)C1=CC(Cl)=C(Cl)C=C1[N+]([O-])=O | 2 | I |
| O=C1C[C@H]2CC[C@@H](CN1CC1=CC(=NO1)C1CCCCC1)N2 | 2 | I |
| CC1(OCCO1)[C@H]1CCCN(C1)C(=O)N[C@H]1CCC2=C1C=CC=C2 | 2 | I |
| CC(C)[C@@H]1NC(=O)N(OC1=O)C1=CC=C(Br)C=C1 | 2 | I |
| CN1C2=C([C@H](NC1=O)C1=CC=C(C)C=C1C)C(=O)N(CC=C)C2 | 2 | I |
| CCOC1=CC=CC(=C1)[C@@H]1CC(=O)NC2=C1C=C1OCOC1=C2 | 2 | I |
| C[C@H]1CN(O[C@H](O1)C1=CNC2=C1C=CC=C2)C(=O)C1=CC=CC=C1 | 2 | I |
| O=C1[C@H]2[C@@H]3C=C[C@H]([C@H]2C(=O)N1\N=C\C1=CNC2=CC=CC=C12)C31CC1 | 2 | I |
| COC1=CC=C(C=C1OC)C1=CC(=O)C2=C(O1)C=C(O)C=C2C | 2 | I |
| CC(C)OC1=CC=CC=C1\C=C1\OC(=O)C2=C1C=C(C)N=C2O | 2 | I |
| COC1=C2O[C@@]3(C)C[C@H](NC(=O)N3C3=CC=CC=C3)C2=CC=C1 | 2 | I |
| O=C1N(C2CC2)[C@@H](NC2=CC=CC=C12)C1=CN=C2C=CC=CC2=N1 | 2 | I |
| O=C1N(C2CC2)[C@H](NC2=CC=CC=C12)C1=CN=C2C=CC=CC2=N1 | 2 | I |
| CC1=CC2=C(SC3=C2N=CN=C3NCC2=CC=CO2)N=C1C | 2 | I |
| O=S(=O)(N[C@H]1CCOC2=CC=CC=C12)N1CCCCCC1 | 2 | I |
| C1CN(CCO1)NC1=NC=NC2=C1C(=CS2)C1=CC=CC=C1 | 2 | I |
| [O-][N+](=O)C1=C2[C@H]3C=CC[C@@H]3[C@H](NC2=C(F)C=C1)C1=CN=CC=C1 | 2 | I |
| CC1=CC=C(OCC(=O)N2CC(=O)NC3=C2C=CC=C3)C(C)=C1 | 2 | I |
| C[C@@H]1CCCC[C@]11NC(=O)N(CN2CCC3=C(C2)C=CS3)C1=O | 2 | I |
| OC1=CC=C(C=C1)N1CCN(CN2C=NC3=CC=CC=C23)CC1 | 2 | I |
| CC1=C(C)C(C#N)=C(N=N1)N1CCC(CC1)C1=CC=C(O)C=C1 | 2 | I |
| [O-]C1=NC2=C(CC[C@@H]2[NH+]2CCCCC2)C(=O)N1C1CCCCC1 | 2 | I |
| CC1=CC=C(C=C1)[C@H]1[C@H]2[C@H](ON1C1=CC=CC=C1C)C(=O)NC2=O | 2 | I |
| CC1=C(C(C)=NN1)S(=O)(=O)N1CCC[C@@H]1C1CCCCC1 | 2 | I |
| O[C@]12CCCC[C@H]1[C@H](N(CC#N)CC2)C1=CC2=C(OCO2)C=C1 | 2 | I |
| O=C1NC(=NC2=C1CCC2)N1CCN(CC2=CC=CC=C2)CC1 | 2 | I |
| C[C@H]1CCC2=C(C1)[C@H](N=N2)[C@@H]1NC(=NO1)C1=CC=CC=C1Cl | 2 | I |
| CC1=CC=CC=C1C1=NO[C@H](N1)[C@@H]1C=C(N=N1)C1=CC=CS1 | 2 | I |
| CC1=CC=CC=C1C1=NO[C@@H](N1)[C@H]1C=C(N=N1)C1=CC=CS1 | 2 | I |
| C[C@@]1(NC(=O)N(CC2=CC=C(Cl)S2)C1=O)C1=CC=CO1 | 2 | I |
| FC1=CC=C(C=C1)N1[C@H](NC2=CC=CC=C2C1=O)C1=CSN=N1 | 2 | I |
| COC1=C(O)C(\C=N\C2=NOC(C)=C2)=CC(Br)=C1 | 2 | I |
| C[C@H]1[C@@H](OC2=C1[C@H]1N=NC=C1CC2)C(=O)NC1CCC(C)CC1 | 2 | I |
| O=C1C[C@@H](\C=C\C2=CC=CO2)C2=C(N=N[C@H]2N1)C1=CC=CS1 | 2 | I |
| CC1=NSC2=C1[C@H](CC(=O)N2)C1=CN=C(N=C1)C1=CC(C)=CC=C1 | 2 | I |
| CC(C)NC(=O)N1CCC[C@H](C1)C(=O)N1CCC[C@@H](C)CC1 | 2 | I |
| CCC1=NC=C(N1)S(=O)(=O)N1CC[C@H](C)[C@H]2CCCC[C@H]12 | 2 | I |
| O[C@@H]1CCCN(C1)C1=NC(=NC=C1)N1C[C@H]2[C@@H](C1)C1CCC2CC1 | 2 | I |
| C[C@H]1CCCC[C@]11NC(=O)N(CC2=CC(=CC=C2F)C#N)C1=O | 2 | I |
| CCN1C(=O)[C@]2(NC3=CC=CC=C3C(=O)N2C2CC2)C2=CC(C)=CC=C12 | 2 | I |
| CC1=C\C(C=CN1)=N\S(=O)(=O)C1=CC(Cl)=C(Cl)N=C1 | 2 | I |
| CSC1=C(C=C(C(=O)N2CC[C@@H](C)[C@H]3CCCC[C@@H]23)C(=O)N1)C#N | 2 | I |
| CC1(O)CN(C1)C1=CC=C(C=C1I)[N+]([O-])=O | 2 | I |
| O=S(=O)(N1CCCCCC1)N1CCCNC2=CC=CC=C12 | 2 | I |
| FC1=CC=CC(F)=C1N1CC[C@@H](C1)N1C(=O)NC2=CC=CC=C2C1=O | 2 | I |
| O=C(NC[C@H]1CN2CCCC[C@@H]2CO1)N1CCCC2=CC=CC=C2C1 | 2 | I |
| CC[C@H]1CCCCN1C(=O)NC[C@@H]1CN2CCCC[C@@H]2CO1 | 2 | I |
| C[C@H]1CC(=O)N[C@H](C)C(=O)N1CC1=NC(=CS1)C(C)(C)C | 2 | I |
| CC1=CC=C(O1)C(=O)N1CCC2(C[C@@H](C(=O)N2)C2=CC=CC=C2)CC1 | 2 | I |
| C[C@H]1CN(C[C@@H](C)O1)C(C)(C)CNC(=O)N1CCCC[C@@H]1C | 2 | I |
| COC1=CC(\C=C2\C(=O)NC3=CC=CC=C23)=CC2=C1OCCO2 | 2 | I |
| O=C(\N=C1/SC=CN1CC1=CC=CC=C1)C1=CC=CNC1=O | 2 | I |
| CC(C)C[C@@H]1SC(C)=NC2=C1C(O)=NN2C1CCOCC1 | 2 | I |
| CCCN1C(=O)[C@@H]2CC3=C(NC4=CC=CC=C34)[C@@H](C)N2C1=O | 2 | I |
| O=C1N(NC2=CC=CC3=C2N=CC=C3)C(=O)C2=CC=CC=C12 | 2 | I |
| C[C@@H]1CCCC[C@@]11NC(=O)N(CC2=CC=C(C=C2)C#N)C1=O | 2 | I |
| CN1C(=O)N[C@@](N(C)C1=O)(C1=CC=CC=C1)C(F)(F)F | 2 | I |
| O=C(CN1CCC2=CC=CC=C12)N1CC(=O)NC2=C1C=CC=C2 | 2 | I |
| CC1=CN2C(=O)C=C(CNC3=CC=C(C=C3)C#N)N=C2C=C1 | 2 | I |
| COC1=C(O)C(\C=C(/C)[N+]([O-])=O)=CC(Br)=C1 | 2 | I |
| O=C1OC2=CC=CC=C2C(=O)\C1=C/NC1=CC=C(C=C1)C#N | 2 | I |
| O=C1OC(=N\C1=C/C1=CNC2=C1C=CC=N2)C1=CC=CC=C1 | 2 | I |
| [O-][N+](=O)[C@H]1CCC(=O)N[C@H]1C1=C(Cl)C(Cl)=CC=C1 | 2 | I |
| CNC1=CC=C(OC2=NS(=O)(=O)C3=C2C=CC=C3)C=C1 | 2 | I |
| CC1=C2C(=O)CC(C)(C)CC2=NC(NC[C@H]2CCCO2)=N1 | 2 | I |
| CC[C@@H]1CO\C(N1)=C(/C(Cl)=C(Cl)Cl)[N+]([O-])=O | 2 | I |
| ClC1=C(Cl)N(CC2=NC3=CC=CC=C3C(=O)N2)C=N1 | 2 | I |
| CN1\C(S\C(=C\C2=CC=CN2)C1=O)=N\C1=NC=CS1 | 2 | I |
| [O-][N+](=O)C1=C(O[C@H]2CCNC2)C=CC(Br)=C1 | 2 | I |
| O=C(NC1CCN(CC1)C(=O)C1CCCCC1)N1CCC1 | 2 | I |
| O=C(NC[C@H]1CCN(C1)C1=CC=CC=C1)N1CCOCC1 | 2 | I |
| CC1=CS\C(N1)=N/S(=O)(=O)C1=CC=C2OCCC2=C1 | 2 | I |
| C[C@H]1CNCCN1C1=CC=C(C=C1[N+]([O-])=O)C(F)(F)F | 2 | I |
| CC1=CC=CC=C1[C@@H]1C[C@@H](NC2=NN=NN12)C1=CC=CS1 | 2 | I |
| O=C1C[C@H]2CC[C@@H](CN1)N2CC1=CC=CC2=NSN=C12 | 2 | I |
| CC1=N[C@H]2[C@@H](C=NN2C(C)=C1)C1=C(NC=N1)C1=CC=CC=C1 | 2 | I |
| CC1=NC=CN1C1=CC=C(N[C@@H]2CCOC(C)(C)C2)C=N1 | 2 | I |
| CC1=NC=CN1C1=NC=CC=C1N[C@@H]1CCOC(C)(C)C1 | 2 | I |
| N1C=NC(=C1C1=CN=C(N=C1)C1=CC=CO1)C1=CC=CC=C1 | 2 | I |
| ClC1=C(C=CC(=C1)S(=O)(=O)\N=C1/NC=CS1)C#N | 2 | I |
| O=C1C[C@H](C2=C(N1)N(N=C2)C1CCCC1)C1=CC=C(C=C1)C#N | 2 | I |
| CCCN1CC[C@@]2(C\C(=N/O)C3=CC=CC=C3O2)CCC1=O | 2 | I |
| O=C1COC2=C(N1)C=C(C=C2)C1=CSC(=N1)C1=COC=C1 | 2 | I |
| BrC1=CC2=C(OCO2)C=C1C1=NNC=C1C=O | 2 | I |
| C[C@@H]1CC(=O)NC2(CCCCC2)C(=O)N1C[C@@H]1CCOC1 | 2 | I |
| CC[C@@]1(C)CN(CCN1)C1=CC=C2C(=O)N(C)C(=O)C2=C1 | 2 | I |
| CC[C@@H]1CN[C@H](C)CN1C1=CC=C2C(=O)N(C)C(=O)C2=C1 | 2 | I |
| CC[C@@H]1CN[C@@H](C)CN1C1=CC=C2C(=O)N(C)C(=O)C2=C1 | 2 | I |
| COC1=CC=C(C=C1C)N1CC(=O)NC2(CCCC2)C1=O | 2 | I |
| COC1=CC=C(N2CC(=O)N[C@@H](C2=O)C(C)(C)C)C(C)=C1 | 2 | I |
| CC[C@H](C#N)S(=O)(=O)\N=C1/C=C(C)NC2=C1C=CC=C2 | 2 | I |
| C[C@@H]1CC(=O)NCC(=O)N1C[C@H]1CC2=CC(C)=CC=C2O1 | 2 | I |
| CC(C)[C@@H]1[C@H](CC(=O)NC1=O)C1=CC=C2OCCOC2=C1 | 2 | I |
| CN([C@H]1CCC[NH+](C)C1)C1=CC2=C(C=C1Cl)C(=O)C([O-])=N2 | 2 | I |
| OC(=O)CC1CCN(CC1)C(=O)N1CCC[C@@H]2CCC[C@@H]12 | 2 | I |
| ClC1=NC=C(CN2C(=O)NC3(CCCCC3)C2=O)S1 | 2 | I |
| C1C[C@@]2(CO1)C[C@@H](CCO2)NC1=CC2=C(OCCO2)C=C1 | 2 | I |
| CC(C)C1=NNC(=N1)C(=O)N1C[C@]2(C)C[C@H]1CC(C)(C)C2 | 2 | I |
| CC1=C(NC2=C1C(C)=CC=C2C)C1=NC(=NO1)[C@H]1CCOC1 | 2 | I |
| C[C@H]1CN(C[C@@H](C)N1)C1=CC(F)=C(Cl)C=C1[N+]([O-])=O | 2 | I |
| C[C@@H]1CN(C[C@@H](C)N1)C1=CC(F)=C(Cl)C=C1[N+]([O-])=O | 2 | I |
| CC1(C)CNCCN1C1=CC(F)=C(Cl)C=C1[N+]([O-])=O | 2 | I |
| CCN1C(=O)C(=NC2=CC=CC=C12)[C@H]1C(=O)NC2=CC=CC=C12 | 2 | I |
| CC1=CC=C(C=C1)N1C(=S)N\C(=C/C2=CC=C3OCOC3=C2)C1=O | 2 | I |
| CCOC(=O)[C@H]1[C@@H]2NC(=S)N(C)[C@@]1(C)OC1=CC=CC=C21 | 2 | I |
| O=C1N[C@]2(CCCC3=CC=CC=C23)C(=O)N1CC1=CC=C(C=C1)C#N | 2 | I |
| CC1=C(SC=C1)\C=C1\C(=O)NC(=O)N(C1=O)C1=CC=CC=C1C | 2 | I |
| CSC1=CC=C(C=C1)[C@H]1N2N=C(C)N=C2NC2=C1C(=O)C[C@H](C)C2 | 2 | I |
| N#CC1(CC(C#N)(C#N)[C@@H](N[C@H]1C1=CC=CC=C1)C1=CC=CC=C1)C#N | 2 | I |
| [O-]C1=C2SC(=[NH+]C2=NC(=S)N1CC=C)N1CCCCC1 | 2 | I |
| C[C@H]1C[C@@H](C)C[NH+](C1)C1=C(\C=C\C([O-])=O)N2C=CSC2=N1 | 2 | I |
| C[C@H]1CCC[C@@H](NS(=O)(=O)C2=C(Cl)N(C)C=N2)[C@@H]1C | 2 | I |
| COC1=CC=C(Cl)C=C1N1C(=S)NN=C1C1=CC=CO1 | 2 | I |
| C[C@H]1OP2(NC3CCCCC3)(O[C@@H]1C)O[C@H](C)[C@H](C)O2 | 2 | I |
| O=C(CN1C(=O)NC2(CCCCCC2)C1=O)C1=CC=CS1 | 2 | I |
| CN1\C(S\C(=C/C2=CC=CC(O)=C2)C1=O)=N\C1=NC=CS1 | 2 | I |
| COC1=C(C)C=C(C)C(=C1)S(=O)(=O)\N=C1/NC(C)=CC=C1 | 2 | I |
| CC1=C(C)S\C(N1)=N\S(=O)(=O)CC1=CC=CC=C1C#N | 2 | I |
| CC(C)CN1C(=O)NC(=CC1=O)N1CCC[C@H]2CCCC[C@H]12 | 2 | I |
| CCCN1[C@@H](NC2=CC(Cl)=CC=C2C1=O)C1=CSN=N1 | 2 | I |
| O=C1N[C@@]2(CCCC3=CC=CC=C23)C(=O)N1C[C@H]1CCCCO1 | 2 | I |
| [O-][N+](=O)C1=CC=C(C=C1)[C@H]1NC2=C(C=CC=C2[C@@H]2C=CC[C@H]12)C#N | 2 | I |
| S=C1NN=C(N1C1CCCCC1)N1CCO[C@@H]2CCC[C@H]12 | 2 | I |
| CN1CCN(CC1)C1=NC(=CS1)C1=CC=C2NC(=O)CCCC2=C1 | 2 | I |
| OC1=CC2=C(C=C1)C(=O)\C(CC2)=C\C1=CN=C(N=C1)N1CCCC1 | 2 | I |
| CC1=C2[C@@H](NC(=O)C[C@@H]2C(=O)C2=CC=C(Br)C=C2)N=N1 | 2 | I |
| O=C(N1CCC[C@H]2CCCC[C@H]12)C1=CC=C(NC1=O)C1=CC=CO1 | 2 | I |
| O\N=C1/CCN(C2=CC=CC=C12)S(=O)(=O)C1=CC=CS1 | 2 | I |
| O[C@H]1CCN(C1)C1=NC(=NC=C1)N1C[C@H]2[C@@H](C1)C1CCC2CC1 | 2 | I |
| O=C(NC1CCCCC1)N1CCCC[C@@H]1CN1CCCC1=O | 2 | I |
| CC1=CC(C)=C2C(=O)NC(=CC2=C1)[C@@H]1CCCN(C1)S(C)(=O)=O | 2 | I |
| C[C@@H]1CCC[C@H](C)N(C1)C(=O)C1=CC(=O)N(N1)C1=CC=C(C)C=C1 | 2 | I |
| CC(C)C1=C(Br)C(=NN1)C(=O)N1[C@@H](C)COC[C@H]1C | 2 | I |
| C[C@H](NC(=O)N1CC[C@H](C)C[C@H]1C)C(=O)N1CCCC[C@@H]1C | 2 | I |
| C[C@@H]1CC2=CC=CC=C2N1C(=O)CN1C(=O)NC2=CC=CC=C12 | 2 | I |
| CC1=C\C(NC=C1)=N\S(=O)(=O)C1=CC=C(C#N)C(Cl)=C1 | 2 | I |
| C[C@H]1CCC[C@H](C1)NC(=O)N1CCC(CC1)C(=O)N1CCCC1 | 2 | I |
| CC1=CC=CC=C1NS(=O)(=O)C1=CC=CC2=NSN=C12 | 2 | I |
| CC1=CC=CN\C1=N/S(=O)(=O)C1=CC(Cl)=C(Cl)N=C1 | 2 | I |
| C[C@@H]1CN([C@@H](C)CN1)C1=CC=C(Br)C=C1[N+]([O-])=O | 2 | I |
| ClC1=CC=C(S1)S(=O)(=O)N1CC2=CC=CC=C2NC(=O)C1 | 2 | I |
| BrC1=CC=CN\C1=N/S(=O)(=O)C1=CC=C(C=C1)C#N | 2 | I |
| C[C@H]1CN(C2=CC=CC=C2C1)S(=O)(=O)C1=CC=C(CO)O1 | 2 | I |
| CC1(C)CN(CCS1)C(=O)NC[C@H]1CN2CCCC[C@@H]2CO1 | 2 | I |
| CNC1=NC(=NC(C(C)C)=C1Br)C1=CN=CC=N1 | 2 | I |
| CC(C)(C)C1=C(Br)C([O-])=NC(=N1)[C@H]1C[NH+]2CCN1CC2 | 2 | I |
| C[C@@H]1SC(=N)C(=C1)C1=NC(=NO1)C1=CC(Br)=CN=C1 | 2 | I |
| C[C@@H]1N(CCC2=C1C=CS2)C(=O)C1=NC(=NN1)C(C)(C)C | 2 | I |
| C[C@H]1N(CCC2=C1C=CS2)C(=O)C1=NNC(=N1)C(C)(C)C | 2 | I |
| C[C@H]1CO[C@H](CO)CN1C1=CN=C2C=C(Cl)C(Cl)=CC2=N1 | 2 | I |
| C(N1CCOCC11CCCC1)C1=NNC(=N1)C1=CC=CS1 | 2 | I |
| COC1=CC=CC=C1\C=C1\NS(=O)(=O)C2=CC=CC=C2C1=O | 2 | I |
| COC1=CC=CC(\C=C2/NC(=O)[C@@H]3CC4=CC=CC=C4CN3C2=O)=C1 | 2 | I |
| CN1C2=C([C@@H](NC1=O)C1=CC=C(F)C=C1)C(=O)N(C2)C1CCCCC1 | 2 | I |
| O=C1N[C@@]2(CCCC3=CC=CC=C23)C(=O)N1CC1=NC2=CC=CC=C2S1 | 2 | I |
| CN1C(=O)N\C(=C/C2=CC=C(O2)C2=CC=C(Cl)C=C2Cl)C1=O | 2 | I |
| C[C@H]1CCC[C@@H](C)N1C(=O)N[C@H]1CN(C(=O)C1)C1=CC=C(C)C=C1 | 2 | I |
| O=C1N([C@@H](NC2=CC=CC=C12)C1=CC=CS1)C1=CC=C2OCCOC2=C1 | 2 | I |
| O=C1NC2(CCCCCC2)C(=O)N1CC1=NC2=CC=CC=C2S1 | 2 | I |
| CN1CCOC2=CC=C(C=C12)S(=O)(=O)NC1=CC=C(C)C=C1C | 2 | I |
| COC1=CC=CC=C1N1[C@H](NC2=CC=CC=C2C1=O)C1=CC=CC=N1 | 2 | I |
| OC1=CC(=CC=C1)C1=NN2[C@@H](C1)C1=CC=CC=C1O[C@H]2C1=CC=CN=C1 | 2 | I |
| O=C([C@@H]1COC2=CC=CC=C2C1)N1CCC2=C(C1)C(=NN2)C1=CC=CC=C1 | 2 | I |
| COC1=CC=CC(\C=C2/SC(=NC2=O)N2CCCCCC2)=C1O | 2 | I |
| O=C(N1CCC[C@H]1C1=CC=C2OCCCOC2=C1)C1=CC2=CC=CC=C2N1 | 2 | I |
| C[C@H]1CN(C2=CC=CC=C2O1)S(=O)(=O)C1=CC=C2SCCNC2=C1 | 2 | I |
| O=C1NN(C(=O)\C1=C/C1=CC=C(O1)C1=CC=CC=C1)C1=CC=CC=C1 | 2 | I |
| ClC1=CC=C(OC[C@H]2CO[C@]3(O2)C(=O)NC2=CC=CC=C32)C=C1 | 2 | I |
| CCCN1C(=O)\C(=C/C2=NC3=CC=CC=C3C(=O)N2)C2=CC=CC=C12 | 2 | I |
| CC1=C\C(NC=C1)=N\S(=O)(=O)C1=CC(Br)=CN=C1Cl | 2 | I |
| CN(C1=CC=CC=C1)S(=O)(=O)C1=CC=C2NC(=O)C(C)(C)C2=C1 | 2 | I |
| CC1=CC=C(\C=C2/C(=O)N=C3SC(=NN3C2=N)C2=CC=C(C)C=C2)C=C1 | 2 | I |
| FC1=CC=CC=C1C1=N\C(=C\C2=CC3=C(OCO3)C=C2Cl)C(=O)N1 | 2 | I |
| CC1=CC=C(C=C1)C1=NO[C@@H](N1)[C@H]1C=C(N=N1)C1=CC(C)=CC=C1C | 2 | I |
| CC(C)(C)C1=CC=C2C=C(NC2=C1)C(=O)N1CCC2(CC1)OCCO2 | 2 | I |
| ClC1=C(C=NC=C1)S(=O)(=O)\N=C1/NC=C(Br)S1 | 2 | I |
| C1C[C@@H](CO1)C1=NC=CN1C1=CC=C(C=C1)C1=NC2=CC=CC=C2N1 | 2 | I |
| CC1=CC(C)=C(CN2C(=O)N[C@]3(CCOC4=C3C=CC=C4)C2=O)C(C)=C1 | 2 | I |
| COC1=CC=C2NC3=C(CCN4[C@H](N5CCC34CC5)C3=CC=CN=C3)C2=C1 | 2 | I |
| CC1=CC=CC(=C1)N1C(=O)C=C2NN(C(=O)C2=C1C)C1=CC=CC=C1C | 2 | I |
| CC1CCC(CC1)NC(=O)N1CCC(CC1)C(=O)N1CCCCCC1 | 2 | I |
| C[C@H](NC(=O)N1CCS(=O)(=O)[C@H](C)[C@@H]1C)C1=CC=C(Cl)S1 | 2 | I |
| CC1=CC=C(C2=CSC3=C2C(=O)NC(=N3)C2=CN=CC=N2)C(C)=C1 | 2 | I |
| ClC1=CC=C2N(CC(=O)C3=CNC4=CC=CC=C34)C(=O)OC2=C1 | 2 | I |
| CC1=NC2=C(C=NN2C2=CC=CC=C2Cl)[C@@H](S1)C1=CC=CNC1=O | 2 | I |
| C[C@H]1CCC[C@H](NS(=O)(=O)C2=CC=CC3=NSN=C23)[C@@H]1C | 2 | I |
| CN1CCOC2=CC=C(C=C12)S(=O)(=O)NC1=CC=C(C)C(C)=C1 | 2 | I |
| CC1=CC(=CC(C)=C1)N1C(=O)NC(=O)\C(=C\C2=CC=C(Cl)C=C2)C1=O | 2 | I |
| O=C(NC[C@@H]1C[C@@]11CCC2=CC=CC=C12)N1CCC2(CC1)OCCO2 | 2 | I |
| O=C(NC[C@H]1CN2CCCC[C@@H]2CO1)N1CCC2(C1)CCCCC2 | 2 | I |
| OC1=CN=CC(=C1)C1=NC(=NO1)C1=CC(F)=CC(Br)=C1 | 2 | I |
| CCN1\C(NC2=CC(Br)=CC=C12)=N\C(=O)[C@H]1CCCOC1 | 2 | I |
| BrC1=CN2C=CN=C2C(NC2=CC=CC3=CC=CN=C23)=N1 | 2 | I |
| O=C1NC2(C[C@@H]1C1=CC=CC=C1)CCN(CC2)C1=NC=NC2=C1SC=C2 | 2 | I |
| COC1=CC=C2N=C(CN3CCSC4(CCCCC4)C3)NC2=N1 | 2 | I |
| FC1=CC=CC(=C1)\N=C1/NC(=O)S/C/1=C/C1=CC=C2N=CC=NC2=C1 | 2 | I |
| CC1=CC2=C(C=C1C)C(=CC(=O)O2)C1=CC2=C(CO)C=NC(C)=C2O1 | 2 | I |
| FC(F)(F)CN1C=C(NC(=O)N2CCC3(C2)CCCCC3)C=N1 | 2 | I |
| O=C1N=C(NC2CCCCC2)S\C1=C\C1=CC2=C(OCO2)C=C1 | 2 | I |
| CN1\C(NC2=CC=CC=C12)=C(\C#N)C1=CSC(=N1)N1CCCCC1 | 2 | I |
| CC1=C(Br)C=C\C(N1)=N\S(=O)(=O)C1=CC=C(Cl)N=C1 | 2 | I |
| C[C@@H]1COCCN1C(=O)NCC1(CCOCC1)C1=CC=CC=C1C | 2 | I |
| O=C(NC1=CC=C2OCOC2=C1)N1CCS[C@H](CC1)C1=CC=CS1 | 2 | I |
| ClC1=CC=C(C=C1)N1C(=O)NC(=O)\C(=C/C2=CC=CS2)C1=O | 2 | I |
| CCC1=CC=C(C=C1)[C@H]1CC(=O)NC2=C1C(=O)N=C(SC)N2C | 2 | I |
| CC[C@H](C)N1CC(=O)N2[C@@H](C)C3=C(C[C@@H]2C1=O)C1=CC=CC=C1N3 | 2 | I |
| O=C1NC(=S)N(C2CCCCC2)C(=O)\C1=C/C=C\C1=CC=CO1 | 2 | I |
| CC1CCC2(CC1)NC(=O)N(CC(=O)C1=CC=C(C)C(C)=C1)C2=O | 2 | I |
| CCSC1=NC(=O)C2=C(NC(=O)C[C@@H]2C2=CC=CC=C2C)N1C | 2 | I |
| ClC1=CC=C(CN2C=NC3=C(SC4=C3NC(=O)CS4)C2=O)C=C1 | 2 | I |
| OC[C@H]1CCCCN1C(=O)\C=C\C1=C(Cl)N=C2SC=CN12 | 2 | I |
| CN1CCN(CC2=NC3=C(C4=C(CCCCC4)S3)C(=O)N2)CC1 | 2 | I |
| CC1(C)C[C@@H](CCO1)[C@@H]1N[C@H]2N(CC3=CC=CC(F)=C3)N=CC2=N1 | 2 | I |
| CC[C@H]1S\C(N(C1=O)C1=CC=C(C)C(C)=C1)=C(\C#N)C(=O)NC | 2 | I |
| CC1=CC=C\C(N1)=N\S(=O)(=O)C1=CC(Br)=CN=C1Cl | 2 | I |
| CC(=O)N1C[C@H](OC2=C1C=CC=C2)C(=O)N[C@@H]1CCC2=C1C=CC=C2 | 2 | I |
| CN(C)S(=O)(=O)N1CCC2=C(C1)C1=CC=CC(Br)=C1N2 | 2 | I |
| CCN1C2=CC=C(C)C=C2C(=O)NC11CCN(CC1)C(=O)C1=CC=CC=C1 | 2 | I |
| ClC1=CC=C(C=C1)S(=O)(=O)N1CC[C@]2(C1)C(=O)NC1=CC=CC=C21 | 2 | I |
| O=C1C[C@@H](C2=C(CN1)N=C(S2)N1CCCC1)C1=CC=CC2=CN=CC=C12 | 2 | I |
| C[C@H]1CNC[C@@H](C)N1CC1=CC=C(C=C1Br)[N+]([O-])=O | 2 | I |
| O=C1NC2=C(SC=C2)C(=O)N1CC1=CC=C(C=C1)N1CCCC1 | 2 | I |
| C[C@H]1C[C@H](C)CN(C1)C(=O)C1CN(C1)C(=O)C1=CNC2=C1C=CC=C2 | 2 | I |
| CN(C)C(=O)[C@H]1CCCN(C1)C(=O)C1=CC2=C(C)C(C)=CC=C2N1 | 2 | I |
| CC1=CC=C(C=C1)[C@@H]1CN([C@H]2[C@@H]1N1CCC2CC1)C(=O)C1=CNC(=O)C=C1 | 2 | I |
| O=C(N1CCC[C@@H]1C1CCCC1)C1=CC=C(NC1=O)C1=CC=CO1 | 2 | I |
| C[C@H]1CN(CCN1C(=O)C1=CC(C)=C(C)NC1=O)C1=CC=CC=C1C | 2 | I |
| [O-]\C(=N/S(=O)(=O)C1=CC=C(F)C=C1)C1=[NH+]C2=CC=CC=C2C=C1 | 2 | I |
| CC1=CC=C2C(CC(=O)NC3CCC4(CC3)OCCO4)=COC2=C1 | 2 | I |
| O=C1CCN(CCN1)S(=O)(=O)C1=CC=C(C=C1)C1=CC=CC=C1 | 2 | I |
| FC1=CC=CC([C@@H]2CC(=O)NC3=C2C=NN3CC2=CC=CO2)=C1F | 2 | I |
| C[C@@H]1CN(CC2=CC=C(C=C2Br)[N+]([O-])=O)[C@H](C)CN1 | 2 | I |
| CCNC1=C(C)C(=NC=N1)N1C=C(I)C=N1 | 2 | I |
| CC[C@@H]1CN(C)C2=CC=CC=C2CN1C(=O)[C@@H]1CC(=O)NC2=C1C=CC=C2 | 2 | I |
| CN1N=C(C)C(Br)=C1CSC1=NC(C)=CC(=O)N1 | 2 | I |
| CCO[C@H]1C[C@H]([O-])C11CCN(CC1)C1=NC=[NH+]C2=CC=C(C)C=C12 | 2 | I |
| CN1C(=O)NC2(CCN(CC3=C(Cl)C4=CC=CC=C4S3)CC2)C1=O | 2 | I |
| C[C@@H]1CCCN(C1)C(=O)C1=CN=C(NC1=O)C12CC3CC(CC(C3)C1)C2 | 2 | I |
| BrC1=CN2C(CN3CC(=O)N[C@H]4CCCC[C@H]34)=CN=C2C=C1 | 2 | I |
| BrC1=CN2C(CN3CC(=O)N[C@@H]4CCCC[C@H]34)=CN=C2C=C1 | 2 | I |
| CC1=C(CN2CCCN(CC2)C2=NC3=CC=CC=C3N2)SC=N1 | 2 | I |
| CC1=C(C)N=C(S1)N1CCN(CC2=CC=CC3=C2NN=C3)CC1 | 2 | I |
| CN1C(=O)[C@@H](CC11CCN(CC1)C(=O)C1=CC=C(O)C=C1)C1=CC=CC=C1 | 2 | I |
| CC1=C(SC=N1)C(=O)N1CCC2(C[C@H](C(=O)N2)C2=CC=CC=C2)CC1 | 2 | I |
| OC1=CC(=CC(=C1)C#N)C1=CC(=CC=C1)S(=O)(=O)N1CCCC1 | 2 | I |
| O=C1NCC2(CCN(CC2)S(=O)(=O)C2=CC=CS2)C2=CC=CC=C12 | 2 | I |
| CC1=NN(C2=C1[C@H](C1=CSC=C1)C1=C(CCCC1=O)N2)C1=CC=CC=N1 | 2 | I |
| FC1=CC=C(C=C1F)S(=O)(=O)N1CC[C@@]2(C1)C(=O)NC1=CC=CC=C21 | 2 | I |
| N=C1N2N=C(SC2=NC(=O)\C1=C\C1=CC=CC=C1)C1=CC=CC=C1 | 2 | I |
| FC(F)(F)C1=CC=CC=C1[C@H]1CN(CCO1)C(=O)C1=NNC=C1 | 2 | I |
| ClC1=C(Cl)C=C(C=C1)N1CC[C@H](SC2=NC=NN2)C1=O | 2 | I |
| C[C@H]1CN(C[C@@H](C)O1)C(C)(C)CNC(=O)N1CCCSCC1 | 2 | I |
| C[C@@H]1CN(C[C@@H](C)O1)C(C)(C)CNC(=O)N1CCCSCC1 | 2 | I |
| CC1=CC=C(C=C1C)N1NC(=O)\C(=C\C2=CC=CO2)C1=O | 2 | I |
| C[C@@H]1NC(=S)N(C1=O)C1=CC=C2OC(F)(F)OC2=C1 | 2 | I |
| C[C@H]1N(N2CCOCC2)C(=O)NC2=CC=CC=C12 | 2 | I |
| O[C@@H]1[C@H]([C@H](OC2=CC=CC=C12)C1=CC=CC=C1)[N+]([O-])=O | 2 | I |
| CCC1=CC(=O)NC(=N1)C1=CC=C2OCCCOC2=C1 | 2 | I |
| [O-][N+](=O)[C@H]1CCC(=O)N[C@H]1C1=CC=CC2=CC=CC=C12 | 2 | I |
| FC1=CC=CC=C1[C@@H]1NC(=O)C2=NN=C([C@H]12)C1=CC=CO1 | 2 | I |
| CC1CCC2(CC1)NC(=O)N(C2=O)C1=CC=CC(=C1)C#N | 2 | I |
| C[C@H]1COCCN1C1=C(C=[NH+]C2=CC=CC=C12)C([O-])=O | 2 | I |
| CC1(C)CC2=C(SC(=N2)C2=NNC=N2)C(=O)C1 | 2 | I |
| N#CC1=C(NC2=CC=C3N=CC=CC3=C2)N=CC=N1 | 2 | I |
| C[C@H]1CCCC[C@]11NC(=O)N(CN2CCCCC2)C1=O | 2 | I |
| CC1=C(C=NN1)S(=O)(=O)N1CCC2(CCCC2)CC1 | 2 | I |
| CC1(C)CCCN1C(=O)N1CCC[C@H](CC(O)=O)C1 | 2 | I |
| CC(C)(C)N1C(=O)C\C(=C\C2=CNC3=C2C=CC=N3)C1=O | 2 | I |
| CC1=C(C)C2=C(S1)N=C(NC2=O)C1=NOC=C1 | 2 | I |
| C[C@H](NC(=O)N1CCC[C@@H](C)C1)C(=O)N1CCCCC1 | 2 | I |
| CC1=NN=C2N1C=CN=C2SC1=CC=CC=C1O | 2 | I |
| N#CC1=C(N2CC[C@H]3CNC[C@@H]23)C2=CC=CC=C2N=N1 | 2 | I |
| C[C@H](N1CC(=O)NC2(CCCCC2)C1=O)C1=CC=CO1 | 2 | I |
| C[C@@H](N1[C@@H](C)C(=O)NC2(CCCC2)C1=O)C1=CC=CO1 | 2 | I |
| CC1=CC=NC=C1N1CC(=O)N[C@@H](C1=O)C(C)(C)C | 2 | I |
| C[C@@H]1CN(CN2C(=O)C3=CC=CC=C3C2=O)[C@@H](C)CN1 | 2 | I |
| CC1=C(C(C)=NN1)S(=O)(=O)N1CCC[C@H]2CCC[C@H]12 | 2 | I |
| O[C@H]1CCCCC[C@@H]1C1=NC(=NO1)[C@@H]1CCCO1 | 2 | I |
| O=C1CN(CCCN1)C1=C(C=NC2=CC=CC=C12)C#N | 2 | I |
| CC1(C)CC2=C(SC(=N2)C2=NC=NN2)C(=O)C1 | 2 | I |
| CC[C@@H]1CS(=O)(=O)C[C@@H](N1)C1=CC=C2OCOC2=C1 | 2 | I |
| CC1(C)N(C[C@@H]2CC3=CC=CC=C3O2)C(=O)NC1=O | 2 | I |
| CC1=CC(C=O)=CC=C1N1CCN2[C@@H](CNC2=O)C1 | 2 | I |
| CC1CCN(CC1)C(=O)NC1(CCN(C)CC1)C#N | 2 | I |
| C[C@@H]1CCC[C@@H](C)N1C(=O)NCC(=O)N1CCCC1 | 2 | I |
| CC1(C)NC(=O)CN(CC2=CC=NC3=C2C=CC=C3)C1=O | 2 | I |
| C[C@H]1COCCN1C1=CC2=C(C=C1Cl)C(=O)C(=O)N2 | 2 | I |
| O[C@H]1CCCC[C@H]1[C@@H]1CCCN1C(=O)N1CCOCC1 | 2 | I |
| O[C@@H]1CCCC[C@@H]1[C@@H]1CCCN1C(=O)N1CCOCC1 | 2 | I |
| CN1CCN(C)[C@H](C1)C1=NC(=CC(=O)N1)C(C)(C)C | 2 | I |
| COC1=CC=C(C)C=C1N1C[C@@H](C)C(=O)NC1=O | 2 | I |
| C[C@@H]1CN(C[C@H]2CC3=CC=CC=C3O2)C(=O)NC1=O | 2 | I |
| C[C@H](N1C[C@@H](C)C(=O)NC1=O)C1=C(C)OC(C)=C1 | 2 | I |
| C[C@@]1(NC(=O)N(C1=O)C1=CC=C(C=C1)C#N)C(F)(F)F | 2 | I |
| C[C@@H]1CCCC[C@H]1CN1C=C(C#N)C(=O)NC1=O | 2 | I |
| CC[C@H]1CCCC[C@H]1N1C=C(C#N)C(=O)NC1=O | 2 | I |
| C[C@@H]1C[NH+]2CCCC[C@@H]2CN1C1=NC=C(S1)C([O-])=O | 2 | I |
| O=C1NC(=O)C2(CCCCCC2)N1CC1=NC=CS1 | 2 | I |
| CN1CCN(CC1)\N=C1/C(=O)NC2=C1C(C)=CC=C2C | 2 | I |
| ClC(=C)CN1C(=O)NC2=CC=CC=C2S1(=O)=O | 2 | I |
| CC[C@@H]1N(CC(=O)NC1=O)C1=CC=C(C#N)C(Cl)=C1 | 2 | I |
| C[C@H]1C\C(=N/O)[C@H]2O[C@H](OC[C@@H]2O1)C1=CC=CC=C1 | 2 | I |
| OC1=NC2=C(C=NN2C2CCCC2)[C@@H](C1)C1=CN=CC=C1 | 2 | I |
| OC[C@]12O[C@H](C=C1)[C@H]1[C@H]2C(=O)N(C1=O)C1=CC(=C(Cl)C=C1)C(F)(F)F | 2 | I |
| CN1C(=O)CCC2=CC(NS(=O)(=O)C3=CC=C(Cl)S3)=CC=C12 | 2 | I |
| O=C(N1CCN(CC2=CC=CC=C2)CC1)C1=CC2=CC=C3C=CC=NC3=C2N1 | 2 | I |
| C[C@H]1CN(CC2=NC3=C(C(=CS3)C3=CC=CC=C3)C(=O)N2)C[C@H](C)O1 | 2 | I |
| OC1=CC=C2C(=O)\C(OC2=C1CN1CCOCC1)=C\C1=CC=CC=C1F | 2 | I |
| COC1=C(OC)C=C([C@H]2NC(=O)C3=CC=CC=C3N2C)C(Br)=C1 | 2 | I |
| O=C(NC1=CC=CC=C1N1CCC2=CC=CC=C2C1)[C@@H]1CCS(=O)(=O)C1 | 2 | I |
| COC1=C(C=C(Cl)C=C1Br)S(=O)(=O)N1CCC[C@H](O)C1 | 2 | I |
| COC1=C(C=C(Cl)C=C1Br)S(=O)(=O)N1CCC[C@@H](O)C1 | 2 | I |
| CN1CCN(CC2=NC3=C(C(=CS3)C3=CC=CC=C3Cl)C(=O)N2)CC1 | 2 | I |
| C[C@H]1C[C@@H]1C(=O)N1CC[C@@H](C1)C1=NN2[C@H](C[C@@H](C)NC2=C1)C(F)(F)F | 2 | I |
| BrC1=CC=C(S1)\C=N\N1C(=S)NN=C1C1=CC=CO1 | 2 | I |
| N=C1N(CC2=CC=CC=C12)C1=CC=C(C=C1)S(=O)(=O)N1CCCCC1 | 2 | I |
| FC1=C(C=CC(Br)=C1)S(=O)(=O)NC1=CC=C[C@@H]2N=CN=C12 | 2 | I |
| CN1[C@@H](C(C(=O)C2=CC=CO2)=C(O)C1=O)C1=CC=CC(Br)=C1 | 2 | I |
| CC1=C([C@H](NC(=S)N1C1CC1)C1=CC=CS1)C(=O)N1CCOCC1 | 2 | I |
| FC1=CC=C(C=C1)C1=NNC=C1CN1C[C@H]2C[C@@H](C1)C1=CC=CC(=O)N1C2 | 2 | I |
| CC(C)CC1=NN2C(S1)=NC(=O)\C(=C/C1=CC=CC3=C1C=CC=C3)C2=N | 2 | I |
| BrC1=CC=C(NC(=O)CN2C=NC3=C(SC=C3)C2=O)C=C1 | 2 | I |
| C[C@]1(CCS(=O)(=O)C1)NC(=O)CC1=COC2=C1C1=CC=CC=C1C=C2 | 2 | I |
| CC1=CC=CC(=C1)N1CCN(CC1)C(=O)C1=CC=C(NC1=O)C1=CC=CS1 | 2 | I |
| C[C@@H]1CN(CC2=NC3=C(C4=C(CCC4)S3)C(=O)N2)C[C@@H](O1)C1=CC=CC=C1 | 2 | I |
| CC1=CC=C(C=C1)N1[C@@H]([C@H](CC1=O)C(=O)N1CCC[C@H](O)C1)C1=CC=CS1 | 2 | I |
| CS(=O)(=O)C1=CC=C2N(CCCC2=C1)C(=O)CC1=CNC2=C1C=CC=C2 | 2 | I |
| O=C(NC[C@@H]1CC2=CC=CC=C2O1)[C@@]12CCC(=O)N1C1=CC=CC=C1S2 | 2 | I |
| OC1=CC=C(C=C1)C1CCN(CC(=O)N2CCO[C@H]3CCCC[C@H]23)CC1 | 2 | I |
| CC1=CNC(CN2CCC[C@@]3(CCC(=O)N(C3)C3CCCC3)C2)=C(C)C1=O | 2 | I |
| O=C(NC1CCCCC1)[C@@H]1CCCN1C(=O)C1=CSC2=C1CCCO2 | 2 | I |
| COC1=CC=C(Br)C=C1N1[C@H](C(C)C)C(=O)N[C@H](C)C1=O | 2 | I |
| BrC1=CC=C(CN2C=CN\C2=N/C(=O)C2=CN=CC=C2)C=C1 | 2 | I |
| CC[C@@H]1N(C(=O)C(C)(C)NC1=O)C1=CC=C(OC)C(Br)=C1 | 2 | I |
| CC[C@@]1(C)NC(=O)[C@H](C)N(C1=O)C1=CC=C(OC)C(Br)=C1 | 2 | I |
| CCC1=NN(C)C(CC2(O)CCC3(CC2)OCCO3)=C1Br | 2 | I |
| CC(C)C(=O)N1CCCC[C@@H]1C(=O)NC1=C(C#N)C2=C(CCCC2)S1 | 2 | I |
| BrC1=C(SC=C1)S(=O)(=O)N[C@H]1CCCCC11OCCO1 | 2 | I |
| COC1=CC=C2C[C@@H](COC2=C1)C(=O)N1CCC(O)(CC1)C(F)(F)F | 2 | I |
| CCN1CC2(CCN(CC2)C(=O)C2=C(C)NC=C2)C[C@@H](C1=O)C1=CC=CC=C1 | 2 | I |
| CN(C1CCS(=O)CC1)C(=O)N[C@H]1CCCN(C1)C1=CC=C(C)C=C1 | 2 | I |
| FC1=CC2=C(CN(CC2)C(=O)N[C@@H]2CCCN(CC(F)(F)F)C2=O)C=C1 | 2 | I |
| CNC1=NC(=NC(=C1Br)C(C)(C)C)[C@H]1CN(C)CCN1C | 2 | I |
| O=C(NCC#CC1=CC=C(C=C1)N1CCCCC1=O)C1=CC=C(C=C1)C#N | 2 | I |
| O=C(C[C@H]1CCS(=O)(=O)C1)NC1=C(C#N)C2=C(CCCCCC2)S1 | 2 | I |
| C[C@]1(NC(=O)N(CC2=CC=C(Cl)N=C2)C1=O)C1=CC=C2CCCC2=C1 | 2 | I |
| OC[C@H]1CCN(C1)C1=CC(F)=C(I)C=C1[N+]([O-])=O | 2 | I |
| COC1=C(O)C(\C=N/C2=C(C#N)C3=C(CN(C)CC3)S2)=CC(Cl)=C1 | 2 | I |
| CC(C)N1C=[NH+]C=C1C1=NC(C2CC2)=C(I)C([O-])=N1 | 2 | I |
| COCC1=C(Br)C(=O)NC(=N1)C1=CC=C(Br)O1 | 2 | I |
| COC1=CC=C(N2C(=S)NC3=CC(Br)=CN=C23)C(OC)=C1 | 2 | I |
| FC1=CC=CC=C1C(=O)N1CCN(CC1)C1=NNC(=C1)C1=CC=CS1 | 2 | I |
| O=C(N1CCS[C@H](CC1)C1=CC=CO1)C(=O)C1=CNC2=CC=CC=C12 | 2 | I |
| CCN1C(=O)S\C(C1=O)=C1/C(=O)NC2=CC=CC=C12 | 2 | I |
| CSC1=N\C(=C/C2=CC=C3OCOC3=C2)C(=O)N1 | 2 | I |
| ClC1=CC2=C(OCO2)C=C1\C=C1\SC(=O)NC1=O | 2 | I |
| O=C(N1CCCCCCC1)C1=CC2=C(NC(=O)CO2)C=C1 | 2 | I |
| CCOC1=CC=C2N(C)C3=NC(=S)NN=C3C2=C1 | 2 | I |
| COC1=C2[C@@H](C#CCO)N(C)CCC2=CC2=C1OCO2 | 2 | I |
| O=C1N[C@@]2(CCCCC3=CC=CC=C23)C(=O)N1CC#N | 2 | I |
| CCCN1C(=O)NC2=C(SC3=NC=CC=C23)C1=O | 2 | I |
| COC1=C2OCOC2=CC2=C1[C@@H](C#C[C@H](C)O)N(C)CC2 | 2 | I |
| O=C(N1C(=S)NC2=CC=CC=C2C1=O)C1=CC=CO1 | 2 | I |
| CC1(C)CN(CCO1)C(=O)[C@H]1CCCC[C@@H]1C(O)=O | 2 | I |
| CC1(C)CN(CCO1)C(=O)[C@H]1CCCC[C@H]1C(O)=O | 2 | I |
| CC[C@H]1COCCN1C(=O)[C@@H]1CNC2=CC=CC=C2O1 | 2 | I |
| CC(C)C1=NNC(=S)N1C1=CC=C2OCOC2=C1 | 2 | I |
| C1COC2=C(OC1)C=C1N=C(NC1=C2)C1=CC=CC=N1 | 2 | I |
| CN1C([S-])=NN=C1N1CCC(CC1)[NH+]1CCCCC1 | 2 | I |
| [O-]C(=O)[C@@H]1CN(CCO1)C1=[NH+]C2=CC=CC=C2C=C1 | 2 | I |
| C[C@@H](NC(=O)N1CCC2(CC1)OCCO2)C1CC1 | 2 | I |
| C[C@H]1NCCN2C1=NN=C2C1=CC=C2C=CC=CC2=N1 | 2 | I |
| [O-]\C=C1/CCCC[NH+]1CC1=CC(=O)N2C=CC=CC2=N1 | 2 | I |
| C[C@@H]1CN(CC(C)(C)O1)C1=[NH+]C(C)=CC=C1C([O-])=O | 2 | I |
| O=C1NC2(CCCCC2)C(=O)N1CN1CCCC1 | 2 | I |
| [O-][N+](=O)C1=CC2=C(NC(=O)C=C2C(F)(F)F)C=C1 | 2 | I |
| CC1CCC2(CC1)N(CC1CCOCC1)C(=O)NC2=O | 2 | I |
| O=C1N(C(=O)C2=C1C=CC=N2)C1=CC=C2NCCCC2=C1 | 2 | I |
| O=C1NC2=CC=CC=C2CN1\N=C\C1=CC=CN=C1 | 2 | I |
| [O-][N+](=O)C1=CC=CC(\C=C2/SC(=O)NC2=S)=C1 | 2 | I |
| C[C@H]1CCN([C@H](C)C1)C(=O)NCC(=O)N1CCCC1 | 2 | I |
| CCC1=NO[C@H](C1)C1=NC2=C(C(C)=CS2)C(=O)N1 | 2 | I |
| CC(C)N1C=[NH+]C=C1C1=NC=C(Br)C([O-])=N1 | 2 | I |
| CC(C)[C@H]1NC(=O)C[C@@H](C)N([C@@H]2CCOC2)C1=O | 2 | I |
| COC1=C(C=C(C)C(Cl)=C1)N1CC(=O)NCC1=O | 2 | I |
| CO[C@H]1CCC[C@@H]1N1CC(=O)NC2(CCCC2)C1=O | 2 | I |
| O=C1CN(C(=O)[C@@H](N1)C1CC1)C1=CC=C2N=CC=CC2=C1 | 2 | I |
| CC[C@@H]1NC(=O)CN([C@@H]2COC3=C2C=CC=C3)C1=O | 2 | I |
| CNC1=NC(=NO1)C1=CC(Br)=CN=C1 | 2 | I |
| C[C@H](O)C1=NC(=NO1)C1=CC(Br)=CN=C1 | 2 | I |
| O=C1NC=C(C=C1)S(=O)(=O)N1CCC[C@@H]2CCC[C@H]12 | 2 | I |
| C[C@@H]1SC(=N)C(=C1C)C1=NC(=NO1)[C@@H]1CCCO1 | 2 | I |
| C[C@H]1CN(CCN1)C(=O)C1=CC(=O)C2=CC=CC=C2O1 | 2 | I |
| C[C@@H]1C[C@@H](C)CN(C1)C(=O)N1CC[C@H](CC(O)=O)C1 | 2 | I |
| C1CC[C@@H]2N[C@@H](CC[C@H]2C1)C1=NN=C2N=CC=CN12 | 2 | I |
| CC1CCC(CC1)(NC(=O)N1CCOCC1)C#N | 2 | I |
| COC1=C(F)C=C2NC(=S)N(C3=CN(C)N=C3)C2=C1 | 2 | I |
| C[C@]1(CN2CC(=O)NC3(CCCCC3)C2=O)CCCO1 | 2 | I |
| CC(C)C1=NC(=NN1)C(=O)N1CCC[C@H](C)CC1 | 2 | I |
| CCC1=CC(=NC=N1)N1CCC2(CC1)OCCC[C@@H]2O | 2 | I |
| O=C(N1CC(=O)NC(=O)C1)C12CC3CC(CC(C3)C1)C2 | 2 | I |
| CC#CCN1C(=O)N[C@](C)(C1=O)C1=C(C)OC(C)=C1 | 2 | I |
| N#C\C(C1=NN=C2CCCCCN12)=C1/CCCCN1 | 2 | I |
| CC1=NNC(=O)C2=NOC(\C=C\C3=CSC=C3)=C12 | 2 | I |
| C[C@H]1CN(C[C@@H](C)O1)C(=O)C1=C(C)C(C(C)=O)=C(C)N1 | 2 | I |
| COC(=O)C1(C)C[C@]2(C)C[C@](C)(C1)C(=O)NC2=O | 2 | I |
| [O-][N+](=O)C1=C(Br)N=C(Br)N1 | 2 | I |
| CSC1=C(C#N)C(=O)N(C(=O)N1)C1=CC=C(C)C=C1 | 2 | I |
| CCC1(CC)[C@H]2NC(Cl)=C(C#N)[C@]1(C#N)C(=[N-])O2 | 2 | I |
| ClC1=CC(=CC2=C1OCO2)C1=NC=CC(=O)N1 | 2 | I |
| C[C@H](N1C(=O)NC(=O)C1=O)C12CC3CC(CC(C3)C1)C2 | 2 | I |
| OC1=C(\C=N\C2=CC=CN=C2)C(=O)OC2=CC=CC=C12 | 2 | I |
| ON=C1CCN(CC1)S(=O)(=O)C1CCCCC1 | 2 | I |
| CC1=N\C(NC(=C1)N1CCOCC1)=C1\C=CC=CC1=O | 2 | I |
| COCN1C(=O)N[C@]2(C[C@H](C)CC(C)(C)C2)C1=O | 2 | I |
| C[C@H](Cl)C(=O)NC(=O)N1C[C@@H](C)OC(C)(C)C1 | 2 | I |
| CC1(C)COCCN1C1=NNC(=S)N1C1CCCC1 | 2 | I |
| CC1(C)COCCN1C(=O)[C@@H]1[C@H]2C[C@H](C=C2)[C@@H]1C(O)=O | 2 | I |
| CC1=CC(=C(C)O1)C1=CN2C=C(C=CC2=[NH+]1)C([O-])=O | 2 | I |
| FC1(F)OC2=C(O1)C=C(C=C2)N1C=CC(=O)NC1=S | 2 | I |
| C[C@@H]1CCCN(C1)C(=O)N1CCC[C@H](CC(O)=O)C1 | 2 | I |
| CC1(C)CC2=C(SC(=N2)N2CCNC(=O)C2)C(=O)C1 | 2 | I |
| [O-]C(=O)[C@@H]1CCCN(C1)C1=NC=[NH+]C2=C1SC=C2 | 2 | I |
| C1[C@@H](NC2=NN=CN2[C@@H]1C1=CC=CO1)C1=CC=CC=C1 | 2 | I |
| O=C1NC(=O)C2(CCCCCC2)N1CC1=CC=CC=N1 | 2 | I |
| C[C@H]1CCC[C@@]2(C1)N(C[C@@H]1CCCO1)C(=O)NC2=O | 2 | I |
| C1COC2(C1)CCN(CC2)C1=NC=NC2=C1C=CN2 | 2 | I |
| CN1NC2=CC(=O)N(CC3=CC=CC=C3)C(C)=C2C1=O | 2 | I |
| C1C[C@@H]2O[C@H]1[C@@H]1CN(C[C@H]21)C1=NC=NC2=C1C=CN2 | 2 | I |
| C[C@@H]1CCC[C@@H]1NC(=O)N1CCS(=O)(=O)[C@H](C)[C@@H]1C | 2 | I |
| CCCN1C(=O)C2=C(O[C@H](C)C(=O)N2)C2=CC=CC=C12 | 2 | I |
| C1C[C@@H](CN1)N1C(=NC2=C1N=CC=C2)C1=CC=CO1 | 2 | I |
| CC[C@]1(C)NC(=O)C2(CCCC2)N([C@H]2CCOC2)C1=O | 2 | I |
| CC[C@]1(C)N([C@H]2CCOC2)C(=O)C2(CCCC2)NC1=O | 2 | I |
| CO[C@@H]1CCC[C@@H]1N1CC(=O)NC2(CCCCC2)C1=O | 2 | I |
| CC[C@@]1(C)NC(=O)CN([C@@H]2CCN3CCCC[C@H]23)C1=O | 2 | I |
| CC[C@H]1NC(=O)CN([C@H]2COC3=C2C=CC=C3)C1=O | 2 | I |
| CC1(C)N(CCNC1=O)C1=NC2=C(CCCC2=O)S1 | 2 | I |
| ClC1=NC(=NC=C1Br)C1=NNN=C1 | 2 | I |
| Br[C@H]1CCCN(C1)C1=NC=NC2=C1N=CN2 | 2 | I |
| OC[C@@H]1CCCN(C1)C1=C(Cl)N=NC(Cl)=N1 | 2 | I |
| OCC#CC1=CC=CC(CN2C(=O)OC3=CC=CN=C23)=C1 | 2 | I |
| CC1(C)CCN(CC1)C(=O)N1CC[C@H](CC(O)=O)C1 | 2 | I |
| C[C@@H]1CC[C@@H](C)N(C1)C(=O)N1CC[C@H](CC(O)=O)C1 | 2 | I |
| CCNC(=O)N1CCN(CC1)C1C[C@@H](C)O[C@H](C)C1 | 2 | I |
| C[C@H]1CN(C[C@@H]1C)C(=O)N1CCC[C@H](CC(O)=O)C1 | 2 | I |
| C[C@H]1CC(=O)NCC(=O)N1C[C@@H]1COC2=C1C=CC=C2 | 2 | I |
| S=C1NC2=C(N=CC=C2)N1C1=CC=C2OCCOC2=C1 | 2 | I |
| COC1=C2NC(=S)N([C@H]3CCCC3(C)C)C2=NC=N1 | 2 | I |
| CN(C1C2CC3CC(C2)CC1C3)C(=O)C1=NNC=N1 | 2 | I |
| CCC1=C(C)C=C(S1)C(=O)N1CC(=O)NC(=O)C1 | 2 | I |
| C(N1CC[C@H]2CC[C@@H](C1)N2)C1=CC=CC2=NON=C12 | 2 | I |
| CC(C)N1N=CC2=C1N=C(O)C[C@H]2C1=CC=C(C=C1)C#N | 2 | I |
| CC1=CC=C(C=C1)[C@@]1(C)NC(=O)N(CC2=CC=C3OCOC3=C2)C1=O | 2 | I |
| CCC1CCC2(CC1)NC(=O)N(CC1=CC=C3OCOC3=C1)C2=O | 2 | I |
| COC1=CC=C(C=C1)N1C[C@@H](CC1=O)NC(=O)N1CCCC[C@H]1C | 2 | I |
| COC1=CC=C(C=C1)N1C[C@@H](CC1=O)NC(=O)N1CCC[C@@H](C)C1 | 2 | I |
| COC1=CC=C(C=C1)N1C[C@H](CC1=O)NC(=O)N1CCC[C@H](C)C1 | 2 | I |
| COC1=CC=C2NC3=C(CCN4C(=O)CN(CC(C)C)C(=O)[C@@]34C)C2=C1 | 2 | I |
| CC[C@@H](C)N1CC(=O)N2CCC3=C(NC4=CC=C(OC)C=C34)[C@]2(C)C1=O | 2 | I |
| CN1C(=O)OC2=CC(=CC=C12)S(=O)(=O)NC1=C(C)C=C(C)C=C1C | 2 | I |
| C[C@H]1CCCC[C@]11NC(=O)N(CC(=O)C2=CC3=CC=CC=C3O2)C1=O | 2 | I |
| COC1=CC=C2CN(C[C@H](C)OC2=C1)C(=O)C1=CC(=NN1)C(F)(F)F | 2 | I |
| CC1=CC=C(CCC(=O)N2CCC3(CC2)NC(=O)C2=CC=CC=C2O3)O1 | 2 | I |
| CCCN1C2=C(C(=O)\C(N2)=C\C2=C(C)C=C(C)C=C2C)C(=O)N(C)C1=O | 2 | I |
| CCO\C(O)=C1/[C@@H]([C@H]2C(=O)CCCC2=NC1=C)C1=CC=C2OCOC2=C1 | 2 | I |
| O=C(NC1=C(OC2=CC=CC=C12)C(=O)N1CCOCC1)C1=CC=CS1 | 2 | I |
| CC1=CC=C2C(=O)C=C(OC2=C1)C(=O)NCC1=CC=C2OCOC2=C1 | 2 | I |
| COC1=CC=C(Cl)C=C1C1=N\C(=C\N=C2\NC=CC=C2)C(=O)O1 | 2 | I |
| CC(=O)N1CCC(CC1)NC1=CC=C(Br)C=C1[N+]([O-])=O | 2 | I |
| COC1=CC=C(C=C1)C1=NC2=CC=C(C=C2C(=O)N1)N1CCN(C)CC1 | 2 | I |
| COC1=CC=C(C=C1)\N=C1/NN=C(CS1)C1=CC=C2OCCOC2=C1 | 2 | I |
| CN(CC1=CC=C2OCOC2=C1)C(=O)N[C@@H]1CCOC2=C1C=CC=C2 | 2 | I |
| CN1\C(=C/C2=C(NN3CCOCC3)C(=O)C2=O)C(C)(C)C2=CC=CC=C12 | 2 | I |
| CC(C)(O)C1=CN(N=N1)C1CCN(CC1)C(=O)[C@H]1CC11CCCCC1 | 2 | I |
| CC(C)S(=O)(=O)NC1=CC=C2OCC[C@@H]3CCCCN3C(=O)C2=C1 | 2 | I |
| CN(C1CCCCC1)C(=O)CN1C(=O)NC2(CCCCCC2)C1=O | 2 | I |
| C[C@H]1OC2=CC=CC=C2N(CC(=O)NC2=C(C#N)C(C)=C(C)S2)C1=O | 2 | I |
| CCOC(=O)N1CCN(CC1)C(=O)NC1=CC=C(Br)C=C1 | 2 | I |
| ClC1=CC=CC(Cl)=C1CC1=NN=C2S[C@@H](NN12)[C@H]1C=CN=N1 | 2 | I |
| ClC1=C(C=C(C=C1)C1=NNC(=O)C=C1)S(=O)(=O)N1CCCCC1 | 2 | I |
| O=C(NC[C@H]1CCOC1)N1CCC[C@H]1C1=CC2=C(OCCO2)C=C1 | 2 | I |
| C[C@H]([NH+]1CCC(CC1)N1CCO[C@@H](C)C1)C1=NC2=CC=CC=C2C([O-])=N1 | 2 | I |
| CCNC(=O)N1CCN(CC1)C(=O)\C=C\C1=NC2=CC=CC=C2S1 | 2 | I |
| C[C@H]1C[C@@H](C)CN(C1)C(=O)NCC1=CC=C(N=C1)N1CCN(C)CC1 | 2 | I |
| C(N1C[C@@H]([C@@H]2[C@H]1C1CCN2CC1)C1=CC2=C(OCO2)C=C1)C1=CNC=N1 | 2 | I |
| O=C(N[C@H]1CCCN(C1)C1=NC=CC=N1)C1=CC2=CC=CC=C2OC=C1 | 2 | I |
| CN1N=CC(NCC2=CC3=C(OCCCO3)C(Cl)=C2)=C(Cl)C1=O | 2 | I |
| [O-]C1=NC2=C(SN=C2C2=CC=CC=[NH+]2)C(=O)N1CC1=CC=C(F)C=C1 | 2 | I |
| C[C@@H](N1C(=O)C2=CC=C(C)C=C2C1=O)C(=O)N[C@@H]1[C@@H]2CCO[C@@H]2C1(C)C | 2 | I |
| CC(C)(C)OC(=O)N1CCC[C@@H](C1)N1CC2=CC=CC=C2NC1=O | 2 | I |
| O=C(NC1CCN(CC1)S(=O)(=O)C1CC1)N1C[C@@H]2CCCC[C@H]2C1 | 2 | I |
| O=C(NC1CCN(CC1)S(=O)(=O)C1CC1)N1C[C@H]2CCCC[C@@H]2C1 | 2 | I |
| COC1=C(OC)C=C(C(=O)N2C[C@H](C)C(=O)NC3=C2C=CC=C3)C(C)=C1 | 2 | I |
| C[C@H](NC(=O)N1CCO[C@@H](C1)C1=CC=CS1)C1=C(C)ON=C1C | 2 | I |
| CC1=CC=C(C=C1)N1CCC[C@@H](NC(=O)N2CCN(CC#C)CC2)C1=O | 2 | I |
| O=C(N[C@@H]1CCN(C1)C(=O)C1CCCCC1)N1CCOC[C@H]1C1CC1 | 2 | I |
| COCC1=C(Br)C(=O)N=C(N1)C1=CC=C2OCOC2=C1 | 2 | I |
| CCC1=CC(=NC(=N1)N1CCCC1)N1CCC2(CC1)OCCC[C@H]2O | 2 | I |
| C[C@@H](NC(=O)N1CCC(CC1)C1(C)OCCO1)C1=C(C)OC(C)=C1 | 2 | I |
| COC(=O)C1=CC=NC(=C1)C1=NC2=C(C3=CC=CC=C3S2)C(=O)N1 | 2 | I |
| CC(C)CC1=NNC(=C1)C(=O)N1CC[C@@]2(C1)CC(=O)C1=CC=CC=C1O2 | 2 | I |
| CN1N=CC(=CC1=O)N1CCC[C@@H](C1)C(=O)NC1CCCCCCC1 | 2 | I |
| CC1(C)CC2=C(CO1)SC1=C2C(=O)N(NC2=CC=CC=C2)C=N1 | 2 | I |
| CC1=CC=C(C=C1C)[C@@H]1C[C@@H](N2N=NN=C2N1)C1=CC=C(F)C=C1 | 2 | I |
| CC1=NN(C(=O)\C1=C\C1=CC2=CC(C)=CC=C2N=C1O)C1=CC=CC=C1 | 2 | I |
| CCCC1=C2COC(C)(C)CC2=C2C(SC3=C2N=CNC3=O)=N1 | 2 | I |
| CC1(C)CC2=C(CO1)SC1=C2C(=O)NC(=O)N1CC1=CC=CC=C1 | 2 | I |
| CC1=C(SC(Cl)=N1)S(=O)(=O)\N=C1/NC=C(Cl)C=C1 | 2 | I |
| COC1=CC(=CC=C1)[C@@H]1[C@H]2CCCC=C2[C@@H](C#N)C(=N)C1(C#N)C#N | 2 | I |
| [O-][N+](=O)C1=C2[C@H]3C=CC[C@@H]3[C@@H](NC2=C(Cl)C=C1)C1=CC=CC=N1 | 2 | I |
| C[C@@H]1C[C@@]2(CC(C)(C)C1)NC(=O)N(CN(C)C1CCCC1)C2=O | 2 | I |
| CC1=CC=C(C)C(SCC(=O)\[NH+]=C2/C=CC3=NC([O-])=NC3=C2)=C1 | 2 | I |
| C[C@@H]1CCCCN1C1=CC(=O)N(C(=O)N1)C12CC3CC(CC(C3)C1)C2 | 2 | I |
| C[C@@H]1CCCN(C1)C1=CC(=O)N(C(=O)N1)C12CC3CC(CC(C3)C1)C2 | 2 | I |
| O=C1NC(=CC(=O)N1C1CCCCC1)N1CCC[C@H]2CCCC[C@H]12 | 2 | I |
| C[C@H]1CCCN(C1)C(=O)NC1CCN(CC1)C(=O)C1CCCCC1 | 2 | I |
| CC1=CC2=C(C=C1C)N(C=[NH+]2)C1=C(C=C(Br)C=N1)C([O-])=O | 2 | I |
| COC1=CC=C(C=C1NC(=O)N1CCC2=C(C=CS2)[C@@H]1C)C#N | 2 | I |
| O=C(N[C@@H]1CCS(=O)(=O)C1)N1CCC[C@@H]1CC1CCCCC1 | 2 | I |
| CC1=CC=C(C=C1C)[C@@H]1C[C@H](N2N=NN=C2N1)C1=CC=CC=C1F | 2 | I |
| OC1=CC=C(\C=C2/CSC3=C(C=CC=C3F)C2=O)C=C1[N+]([O-])=O | 2 | I |
| C[C@H]1CCCC[C@]11NC(=O)N(CC2=CC=C(Cl)C3=C2N=CC=C3)C1=O | 2 | I |
| CN1C=C(C=N1)C1=NC2=C(C(=CS2)C2=CC=C(C)C(C)=C2)C(=O)N1 | 2 | I |
| CC1=CC=C(\C=C2/C(=O)NC(=O)N(C2=O)C2=CC=CC3=C2C=CC=C3)C=C1 | 2 | I |
| CC1=NC2=CC(=CC=C2O1)\N=C\C1=C(O)CC2(CCCCC2)CC1=O | 2 | I |
| CCNC(=O)N1CCN(CC1)C(=O)C(C)(C)C1=CC=CC=C1F | 2 | I |
| C[C@@H](NC(=O)N1CCC[C@H](C)CC1)C1=CC=C(C=C1)N1C=CN=C1 | 2 | I |
| O=C(NC1CCCCCC1)N1CCC[C@H](CN2CCOCC2)C1 | 2 | I |
| O=C1NC(=NC(=C1)[C@H]1CCCCN1C1CCCC1)C1=CN=CC=C1 | 2 | I |
| CC1CCN(CC1)C(=O)NCC1(CCCCC1)N1CCOCC1 | 2 | I |
| C[C@@H](N1C(=O)C2=CC=C(C)C=C2C1=O)C(=O)N[C@@H]1CCC[C@H](C)C1 | 2 | I |
| C[C@@H]1CN(CCO1)C1=CC=C(NC(=O)C2(CCCCC2)C#N)C=C1 | 2 | I |
| O[C@H]1COCCN(C1)C1=NC(=NC2=CC=CC=C12)C1=CC=CC=C1 | 2 | I |
| CC1(C)CCCN1C(=O)NC1CCN(CC1)C(=O)C1CCCCC1 | 2 | I |
| C[C@H](NC(=O)N(C)[C@@H]1CCC[C@@H](C)C1)C(=O)N1CCCC[C@@H]1C | 2 | I |
| CC[C@@H]1CCC[C@H](C1)NC(=O)N1CCN(CC1)C1=NC=CS1 | 2 | I |
| C[C@@H]1CCN([C@@H](C)C1)C(=O)NC1CCN(CC1)C(=O)C1=CC=C(C)C=C1 | 2 | I |
| C[C@@H]1CN(CCO1)C1CCN(CC1)C(=O)N[C@@H]1CCCC[C@H]1C | 2 | I |
| CC(C)(C)N1\C(NC2=CC=CC=C12)=N\C(=O)C1=CC=C2COCC2=C1 | 2 | I |
| CC(C)SC1=CC=CC=C1C(=O)\N=C1/NN=C2C=C(C)C=CN12 | 2 | I |
| CC1=CC=C2CCN(C2=C1)S(=O)(=O)C1=CC=C2NC(=O)C(C)(C)C2=C1 | 2 | I |
| COC(=O)C1=CC(Br)=CC=C1NC(=O)N1CCCC1 | 2 | I |
| O=C(NC[C@@H]1CN2CCCC[C@@H]2CO1)N1C[C@H]2CCCC[C@H]2C1 | 2 | I |
| CC(C)C1=C(Br)C(=O)NC(=N1)C1=CC=C2OCCOC2=C1 | 2 | I |
| CCN1\C(NC2=CC(Br)=CC=C12)=N\C(=O)C1CCOCC1 | 2 | I |
| CC1=NC(N2CCC3(C[C@@H](C(=O)N3)C3=CC=CC=C3)CC2)=C(C#N)C(C)=C1 | 2 | I |
| C[C@@H]1CN(C2=C(C=CC=C2)C(=O)N1)S(=O)(=O)C1=CC(C)=CC=C1C | 2 | I |
| CC1=C(\C=C2/SC(=O)N/C/2=N\C2=CC=C(Cl)C=C2)C(C)=NO1 | 2 | I |
| CC(C)N1N=CC2=C1NC(=O)C[C@H]2C1=CC=C(O1)C1=CC=CC=C1 | 2 | I |
| C[C@@H]1CC2=CC=C(C)C=C2[C@H]1NC(=O)N1CCC(CC1)N(C)C(C)=O | 2 | I |
| ClC1=CC=C(C=C1)C1=C[C@@H](N2N=CNC2=N1)C1=CC=C(C=C1)C#N | 2 | I |
| CN1C(=O)C2=CC=C(C=C2C1=O)\N=C\C1=CC(Br)=CC=C1O | 2 | I |
[truncated: 176,735 more chars]
